# Global Trends in Ischemic Heart Disease Mortality from 1990 to 2021 and 2036 Projections: Insights from GBD 2021 Data

**DOI:** 10.5334/gh.1486

**Published:** 2025-10-10

**Authors:** Xiaoqin Zhou, Weiqiang Ruan, Hui Jie, Huizhen Liu, Ting Wang, Jing Li, Ke Lin, Jing Lin

**Affiliations:** 1 Center of Biostatistics, Design, Measurement and Evaluation (CBDME), Department of Clinical Research Management, West China Hospital, Sichuan University, Chengdu, PR China; 2 Department of Pediatric Cardiovascular Surgery, Children’s Heart Center, West China Second University Hospital, Sichuan University, Chengdu, PR China; 3 Key Laboratory of Birth Defects and Related Diseases of Women and Children (Sichuan University), Ministry of Education, Sichuan, Chengdu, PR China; 4 Department of Thoracic Surgery and Institute of Thoracic Oncology, West China Hospital, Sichuan University, Chengdu, PR China; 5 Center of Clinical Epidemiology and Evidence-Based Medicine, West China Hospital, Sichuan University, Chengdu, PR China; 6 Department of Cardiovascular Surgery, West China Hospital, Sichuan University, Chengdu, PR China; 7 Department of Anesthesiology, West China Hospital, Sichuan University, Chengdu 610041, People’s Republic of China

**Keywords:** Ischemic heart disease, Global Burden of Disease Study 2021, Age-period-cohort analysis, Mortality trends, Health projections

## Abstract

**Background:**

Ischemic heart disease (IHD) remains a significant global health challenge despite advancements in prevention and treatment. This study aims to comprehensively analyze global IHD mortality trends from 1990 to 2021 and projections for 2036, to support evidence-based decision-making for IHD prevention and management strategies across diverse contexts.

**Methods:**

Using data from the Global Burden of Disease Study 2021, we conducted multi-level analyses of IHD mortality trends: globally, across socio-demographic index (SDI) quintiles, 21 Global Burden of Disease (GBD) regions, and 204 countries. We performed age-period-cohort (APC), decomposition, risk factor, and frontier analysis. Bayesian APC modeling was used for projections to 2036.

**Results:**

The global age-standardized mortality rate (ASMR) for IHD decreased from 1990 to 2021 (EAPC: –1.301%, net drift: –1.1%) despite a 67.5% increase in total deaths. APC analysis revealed exponential growth in IHD mortality rates with age, with the inflection point at 62.5 years in low SDI regions and 77.5 years in high SDI regions. High SDI regions demonstrated significant period and cohort effects. Frontier analysis showed that Nauru and Ukraine were the countries with the highest effective differences. Decomposition analysis identified population growth and aging as primary factors increasing IHD mortality. High systolic blood pressure remained the leading global risk factor, with increasing contributions from high fasting plasma glucose and high body-mass index, especially in high SDI regions. Projections indicate continued global ASMR decrease by 2036, but with concerning increases in Eastern Europe and some African regions.

**Conclusions:**

This study reveals the complex global landscape of IHD, emphasizing that high SDI regions should focus on comprehensive care for older adults and managing metabolic risk factors such as diabetes and obesity, while low and middle SDI regions need to strengthen healthcare systems and implement early prevention strategies. Regions projecting mortality increases require urgent attention and interventions.

**Highlights:**

**What is new?**

- Global IHD age-standardized mortality rates decreased from 1990 to 2021 (EAPC: –1.301%, net drift: –1.1%), despite a 67.5% increase in total deaths, reflecting advancements in care and ongoing challenges of population aging and growth.
- APC analysis showed exponential growth in IHD mortality rates with age across all SDI regions, with low SDI regions’ inflection point 15 years earlier than high SDI regions, suggesting prioritization of comprehensive care for the elderly in high SDI areas.
- Decomposition analysis showed South Asia and East Asia experienced the largest increases in IHD deaths, with epidemiological changes contributing to death increases rather than reductions, underscoring the need for effective policies to address population growth and aging in these rapidly developing regions.
- High systolic blood pressure remains the leading global risk factor for IHD, with increasing contributions from high fasting plasma glucose and high body-mass index, especially in high SDI regions.
- Projections indicate continued global ASMR decrease by 2036, but with concerning increases in Eastern Europe and some African regions, particularly Southern Sub-Saharan Africa, necessitating targeted interventions.

**What are the clinical implications?**

- High SDI regions should focus on comprehensive care for the elderly and managing metabolic risks like hypertension, diabetes, and obesity.
- Low and middle SDI regions need to strengthen healthcare systems and implement early prevention to combat rising IHD mortality.
- Regions with projected increases, such as Eastern Europe and Southern Sub-Saharan Africa, require urgent policy interventions and resource allocation to address disparities and improve preventive care access.

## Introduction

Over the past three decades, the global burden of ischemic heart disease (IHD) has changed significantly. Despite advancements in prevention and treatment strategies and the influence of demographic shifts and socioeconomic development, IHD remains a major health challenge across diverse socioeconomic contexts (1,2). IHD was estimated to account for 9.14 million deaths worldwide in 2019, ranking as the leading cause of mortality among all cardiovascular diseases (1). While high-income countries have seen a dramatic decrease in IHD mortality, low- and middle-income countries face greater challenges due to factors such as rapid urbanization, the adoption of westernized lifestyles, and limitations in chronic disease management (3,4,5). During this epidemiological transition, population risk factor profiles have shifted, with high-income countries showing stabilization or improvement, while many low- and middle-income countries continue to see worsening trends (1). Although previous studies have reported on global IHD epidemiology, there is still a lack of in-depth analysis of long-term mortality trends across different socioeconomic backgrounds (6,7,8).

In this study, we conducted an age-period-cohort (APC) analysis for IHD mortality trends across 204 countries and territories from 1990 to 2021, using the updated data reported in the Global Burden of Disease Study 2021 (9,10). To generate a comprehensive assessment of IHD healthcare burden, we explored the variations in IHD mortality trends by socio-demographic index (SDI) quintile, region, nation, age, and sex. Additionally, we performed a decomposition analysis to evaluate the contributions of aging, population growth, and epidemiological strategies to mortality changes, and a risk factor analysis to assess the shifting risks across different regions. Finally, we employed the Bayesian APC (BAPC) model to project the IHD death burden for the next 15 years. Our research aims to provide a comprehensive analysis of global IHD mortality trends, their potential drivers, and future projections using the latest GBD data. This information is intended to inform targeted public health strategies and resource allocation, supporting evidence-based decision-making for IHD prevention and management strategies across various socioeconomic contexts worldwide.

## Methods

### Data sources and definitions

The GBD study, led by the Institute for Health Metrics and Evaluation (IHME) at the University of Washington, Seattle, is the most comprehensive global observational epidemiological study to date. The GBD 2021 employs a consistent methodology to produce standardized health metrics for 204 countries and territories from 1990 to 2021. It examines descriptive epidemiological data on 371 diseases and injuries and 88 risk factors, enabling comparisons both within and between countries (10,11). Metric and measure definitions can be found in Table S1.

For this study, we focused on IHD, which was defined based on the following International Classification of Diseases (ICD) codes: 410–414 (ICD-9) and I20–I25 (ICD-10). Mortality estimates were derived using vital registration data coded to the ICD system. We obtained cause-specific deaths attributed to IHD from the GBD 2021 database. The epidemiological data of interest includes the absolute death number and age-standardized mortality rates (ASMR) of IHD from 1990 to 2021, categorized by gender, age, region, and country. All estimates from GBD are presented with 95% uncertainty intervals (UIs).

In this study, we utilized two complementary measures—the estimated annual percentage change (EAPC) and the net drift—to describe the trends in ASMR from 1990 to 2021. The EAPC quantifies the average rate of change in ASMR over time by calculating the percentage change using the slope from a logarithmic linear regression model. The net drift value, derived from APC analysis, represents the overall annual percentage change in age-standardized rates over time, adjusted for both period and cohort effects (12).

The analysis was stratified at multiple levels: global, SDI quintiles, GBD regions, and national. The SDI is a composite measure that reflects a country’s development level by combining data on key indicators such as per capita income, education levels, and fertility rates (9,13). Based on 2021 SDI classification criteria and their SDI values (Tables S2, S3), countries were categorized into five SDI regions: low, low-middle, middle, high-middle, and high (9). Additionally, the 204 countries and territories were grouped into 21 GBD regions according to their geographical locations. This multi-layered analysis provided insights into IHD mortality patterns across various geographical and socioeconomic contexts, allowing for the identification of both broad global trends and specific regional variations in IHD mortality.

We also examined the burden of IHD deaths across different age groups. We divided the death counts into five age categories: 15–29 years, 30–44 years, 45–59 years, 60–74 years, and 75 years and above. We calculated the proportions of deaths for each group to analyze the age distribution of IHD mortality.

### Analytical methods and predictive modeling

We employed several analytical methods to examine temporal trends, decompose factors contributing to IHD mortality changes, analyze associated risks, and predict future trends. APC analysis was used to investigate the effects of age, period, and birth cohort on IHD mortality trends from 1992 to 2021 (12,14). To explore the relationship between socioeconomic development and IHD mortality, we conducted a frontier analysis, identifying the lowest potentially achievable IHD mortality rates at various levels of development (15). A three-factor decomposition method was applied to quantify the contributions of demographic and epidemiological factors to changes in IHD mortality (16,17). Additionally, we performed a risk factor analysis to examine the contributions of the top eight global risk factors for IHD mortality across different regions and SDI levels in 1990 and 2021 (9). Finally, we utilized a BAPC model to project IHD death cases and mortality rates from 2022 to 2036, accounting for age, period, and cohort effects (18,19). Detailed methodologies for these analyses are provided in the Supplementary Methods. The GBD 2021 complies with the Guidelines for Accurate and Transparent Health Estimates Reporting (GATHER) statement (20).

### Analysis software

Statistical analyses were performed using R version 4.2.3. The BAPC model was implemented using the R packages BAPC (version 0.0.36) and INLA (version 22.05.07).

## Results

### Global and regional trends

**Figure 1:**
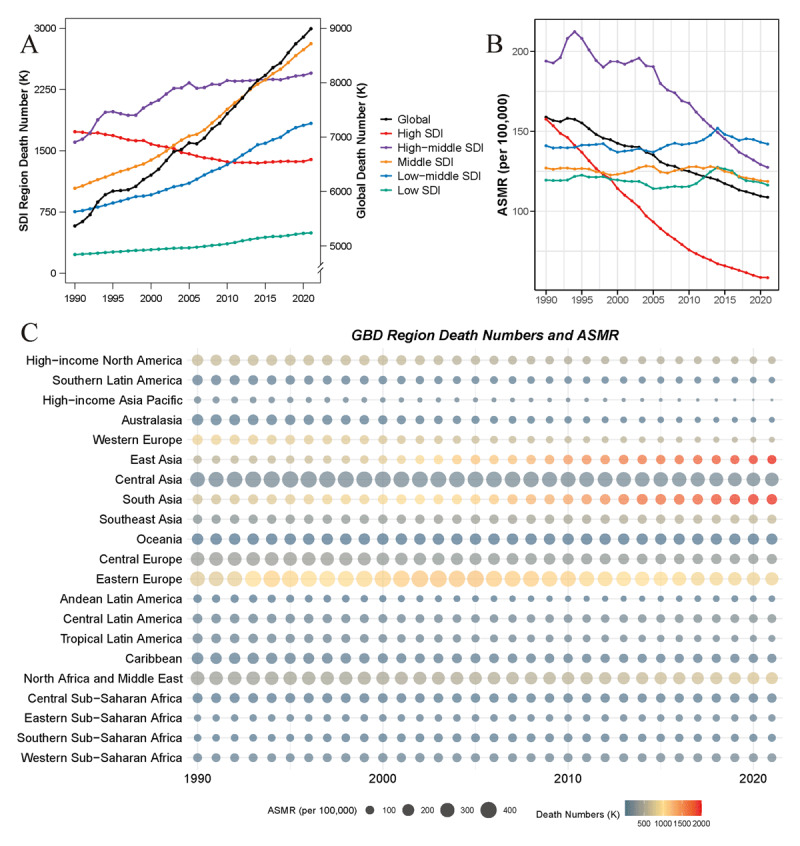
Trends in IHD mortality from 1990 to 2021. **(A)** and **(B)** show the death numbers (in thousands) and age-standardized mortality rates (ASMR, per 100,000 population) across different SDI regions, respectively. **(C)** presents the death numbers and ASMR across different GBD regions. *ASMR, age-standardized mortality rate; GBD, Global Burden of Disease; IHD, Ischemic heart disease; SDI, socio-demographic index*.

Our analysis revealed significant changes in the global burden of IHD from 1990 to 2021. The total number of IHD deaths globally increased substantially over the past three decades, rising from 5.37 million (95% UI: 5.08–5.56) in 1990 to 8.99 million (95% UI: 8.26–9.53) in 2021, representing a 67.5% increase. However, the global ASMR for IHD showed a declining trend during this period. The EAPC in ASMR was –1.301% per year, with a net drift of –1.1% per year. The trends in IHD mortality varied considerably across different SDI regions, with high SDI regions experiencing the largest decline in ASMR (EAPC: –3.428%, net drift: –2.77%) (Figure 1 A, 1B, Table 1, and Table S4).

**Table 1:** Global and Regional ASMR and trends for ischemic heart disease from 1990 to 2021.


LOCATION	1990		2021		1990–2021	
		
	DEATH CASES	ASMR	DEATH CASES	ASMR	EAPC	NET DRIFT
					
	(×10^3^)(95% UI)	(PER 100,000)(95% UI)	(×10^3^)(95% UI)	(PER 100,000)(95% UI)	(95% CI)	(95% CI)

Global	5367.14 (5076.40,5562.77)	158.9 (148.14,165.3)	8991.64 (8264.12,9531.13)	108.73 (99.6,115.38)	–1.301 (–1.341,–1.26)	–1.1 (–1.15,–1.06)

**SDI region**						

High SDI	1732.39 (1591.27,1797.85)	157.59 (144.19,163.92)	1392.37 (1217.17,1489.07)	58.45 (52.18,61.92)	–3.428 (–3.533,–3.324)	–2.77 (–2.92,–2.62)

High-middle SDI	1604.86 (1527.81,1657.94)	193.94 (182.11,200.6)	2450.43 (2218.84,2647.30)	127.5 (115.03,137.72)	–1.564 (–1.771,–1.356)	–2.21 (–2.33,–2.08)

Middle SDI	1038.97 (978.52,1100.28)	127.06 (118.35,134.97)	2811.12 (2564.66,3023.43)	118.71 (107.23,127.8)	–0.115 (–0.186,–0.045)	–0.37 (–0.44,–0.31)

Low-middle SDI	753.13 (698.70,804.55)	140.99 (129.76,151.24)	1834.65 (1699.51,1964.08)	142.1 (131.3,151.87)	0.159 (0.086,0.232)	–0.12 (–0.2,–0.03)

Low SDI	228.81 (202.39,255.18)	119.5 (106.26,132.45)	493.48 (448.13,543.12)	116.41 (105.21,127.69)	0.006 (–0.102,0.113)	–0.46 (–0.57,–0.35)

**GBD region**						

High-income North America	644.90 (580.45,675.67)	177.72 (160.27,186.07)	534.77 (468.40,571.53)	75.85 (67.17,80.6)	–3.09 (–3.238,–2.942)	–2.59 (–2.76,–2.41)

Southern Latin America	62.79 (59.93,64.74)	149.43 (141.12,154.54)	49.10 (45.02,51.73)	54.41 (50.08,57.26)	–2.918 (–3.076,–2.76)	–2.73 (–2.9,–2.56)

High-income Asia Pacific	117.62 (107.06,122.95)	67.04 (59.85,70.5)	152.70 (124.00,168.79)	25.56 (21.78,27.65)	–3.041 (–3.201,–2.881)	–2.74 (–3.16,–2.33)

Australasia	40.16 (37.29,41.66)	176.76 (162.82,184.02)	28.64 (24.37,30.97)	46.67 (40.23,50.21)	–4.524 (–4.622,–4.426)	–4.55 (–4.88,–4.22)

Western Europe	879.29 (811.26,910.87)	148.22 (136.54,153.85)	543.04 (463.99,584.34)	47.27 (41.45,50.42)	–3.894 (–3.991,–3.797)	–4.12 (–4.33,–3.92)

East Asia	570.43 (505.99,639.93)	93.92 (83.87,105.3)	2008.01 (1683.97,2335.23)	108.9 (91.18,125.79)	0.896 (0.586,1.208)	–0.02 (–0.21,0.16)

Central Asia	131.77 (124.69,136.25)	320.47 (299.83,332.36)	175.39 (159.20,192.41)	265.51 (240.67,290.42)	–1.012 (–1.287,–0.736)	–2.31 (–2.46,–2.15)

South Asia	708.22 (643.04,771.05)	136.39 (122.87,149.55)	1990.11 (1824.49,2155.70)	149.14 (136.97,161.16)	0.436 (0.302,0.571)	–0.03 (–0.18,0.13)

Southeast Asia	252.65 (229.37,275.07)	114.72 (103.39,125.65)	638.70 (575.91,694.08)	110.92 (100.18,120.2)	–0.103 (–0.18,–0.025)	–0.21 (–0.25,–0.17)

Oceania	4.53 (3.75,5.49)	182.55 (155.47,217.42)	11.14 (9.33,13.32)	170.89 (145.43,201.15)	–0.182 (–0.224,–0.141)	–0.17 (–0.32,–0.02)

Central Europe	364.09 (349.91,371.87)	272.56 (259.22,279.18)	331.28 (299.88,352.60)	139.98 (126.84,148.91)	–2.455 (–2.557,–2.353)	–3.42 (–3.61,–3.24)

Eastern Europe	786.27 (751.13,803.30)	323.17 (305.26,331.76)	903.62 (811.06,990.58)	252.89 (226.96,277.15)	–1.237 (–1.691,–0.782)	–2.3 (–2.62,–1.97)

Andean Latin America	17.09 (15.55,18.72)	92.95 (84.56,101.24)	32.96 (28.04,39.37)	58.17 (49.56,69.33)	–1.741 (–2.118,–1.363)	–1.72 (–1.82,–1.62)

Central Latin America	88.86 (84.86,90.85)	125.72 (118.69,129.14)	247.05 (221.19,273.15)	103.69 (92.48,114.59)	–0.764 (–0.997,–0.532)	–0.52 (–0.6,–0.43)

Tropical Latin America	107.55 (102.01,110.64)	135.91 (126.39,140.82)	162.30 (149.05,170.32)	64.49 (58.98,67.84)	–2.272 (–2.367,–2.176)	–1.87 (–1.97,–1.78)

Caribbean	44.60 (42.48,46.17)	188.97 (179.08,195.79)	61.45 (54.94,69.04)	112.5 (100.51,126.54)	–1.706 (–1.899,–1.513)	–0.93 (–1.05,–0.8)

North Africa and Middle East	386.04 (357.95,418.21)	275.18 (253.62,299.12)	769.14 (685.36,858.25)	202.85 (180.59,223.68)	–1.026 (–1.075,–0.977)	–1.41 (–1.5,–1.32)

Central Sub-Saharan Africa	23.27 (18.25,29.48)	134.9 (107.86,167.2)	49.85 (38.89,63.65)	119.34 (93.7,150.29)	–0.572 (–0.655,–0.489)	–0.59 (–0.68,–0.5)

Eastern Sub-Saharan Africa	43.99 (39.10,50.52)	69.44 (61.05,78.85)	101.22 (87.73,117.27)	72.16 (62.09,82.99)	–0.055 (–0.14,0.03)	–0.43 (–0.48,–0.38)

Southern Sub-Saharan Africa	17.90 (15.56,19.84)	75.96 (64.95,84.84)	39.82 (36.78,43.19)	83.44 (76.93,90.19)	0.266 (–0.128,0.661)	–0.56 (–0.79,–0.32)

Western Sub-Saharan Africa	75.12 (64.54,87.13)	105.29 (90.24,121.56)	161.33 (140.03,185.44)	105.97 (92.83,120.17)	0.024 (–0.101,0.149)	–0.23 (–0.28,–0.17)


*ASMR, age-standardized mortality rate; CI, confidential interval; EAPC, estimated annual percentage change; SDI, socio-demographic index; UI, uncertainty interval*.

Regionally, East Asia, South Asia, and Eastern Europe were the top three regions for IHD deaths in 2021, collectively accounting for 54.6% of global IHD deaths. Central Asia, Eastern Europe, North Africa and Middle East had the highest ASMR, while high-income Asia Pacific, Western Europe, and Australasia had the lowest. From 1990 to 2021, Australasia (EAPC: –4.524%, net drift: –4.55%), Western Europe (EAPC: –3.894%, net drift: –4.12%), and high-income Asia Pacific (EAPC: –3.041%, net drift: –2.74%) showed the most significant decreases in ASMR. However, some regions experienced minimal changes or even increases in ASMR, such as South Asia (EAPC: 0.436%, net drift: –0.03%) and East Asia (EAPC: 0.896%, net drift: –0.02%) (Figure 1C, Table 1, and Table S4).

### National trends

**Figure 2:**
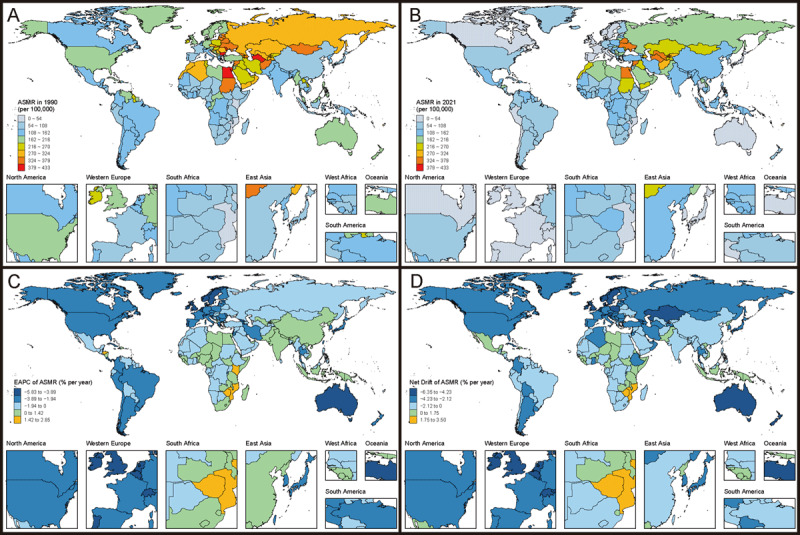
Age-standardized mortality rates (ASMR) and trends for IHD from 1990 to 2021 in 204 countries and territories. **(A)** and **(B)** show the ASMR (per 100,000 population) in 1990 and 2021, respectively. **(C)** presents the estimated annual percentage change (EAPC) of ASMR (% per year) by country, and **(D)** illustrates the net drift of ASMR (% per year) over the study period. *ASMR, age-standardized mortality rate; EAPC, estimated annual percentage change; IHD, Ischemic heart disease*.

Figure 2 and Table S5 reveal significant global disparities and dynamic patterns of change in ASMR for IHD across countries from 1990 to 2021. In 2021, San Marino, Japan, South Korea, and France reported the lowest ASMR. EAPC and net drift data demonstrate significant changes and consistent improvement trends in these developed countries: San Marino (EAPC: –3.28%, net drift: –3.18%), Japan (EAPC: –2.95%, net drift: –2.25%), South Korea (EAPC: –3.03%, net drift: –4.15%), and France (EAPC: –3.48%, net drift: –3.44%). These countries are depicted in deep blue or dark blue in Figures 2C and 2D, indicating substantial downward trends.

In contrast, some countries in Eastern Europe, Central Asia, the Middle East, and North Africa generally show higher ASMR, such as Ukraine, Belarus, Egypt, and Syria. However, most of these countries demonstrate positive trends, as seen in Ukraine (EAPC: –0.30%, net drift: –0.96%) and Syria (EAPC: –0.47%, net drift: –1.34%). Notably, Sub-Saharan African countries face severe challenges, with many showing rising IHD mortality rates. Kenya (EAPC: 1.71%, net drift: 1.39%), Lesotho (EAPC: 2.84%, net drift: 3.49%), and Zimbabwe (EAPC: 1.75%, net drift: 2.24%) all demonstrate significant upward trends. These countries are colored orange in Figures 2C and 2D, reflecting concerning trends.

### Age and sex disparities

The age distribution of IHD deaths exhibited a clear shift towards older age groups from 1990 to 2021 (Figure S1 and Table S6). Globally, the proportion of IHD deaths in the 75+ age group rose from 47.9% in 1990 to 52.0% in 2021. This shift was evident in both high SDI regions (61.4% to 68.5%) and low SDI regions (25.7% to 32.9%). Australasia, high-income Asia Pacific, and Western Europe had the highest proportions of IHD deaths in the 75+ age group in 2021 (75.5%, 76.5%, and 77.0% respectively). In contrast, Oceania, Central Sub-Saharan Africa, and Eastern Sub-Saharan Africa had much lower proportions in this age group (22.6%, 28.8%, and 32.4% respectively), with a higher proportion of deaths in younger age groups. Although ASMR decreased for both sexes, the decline was more pronounced in females. The gender disparity was most notable in high SDI regions, where the ratio of male to female ASMR increased from 1.79 in 1990 to 1.97 in 2021 (Figure S2 and Table S7).

### Age-period-cohort effects

**Figure 3:**
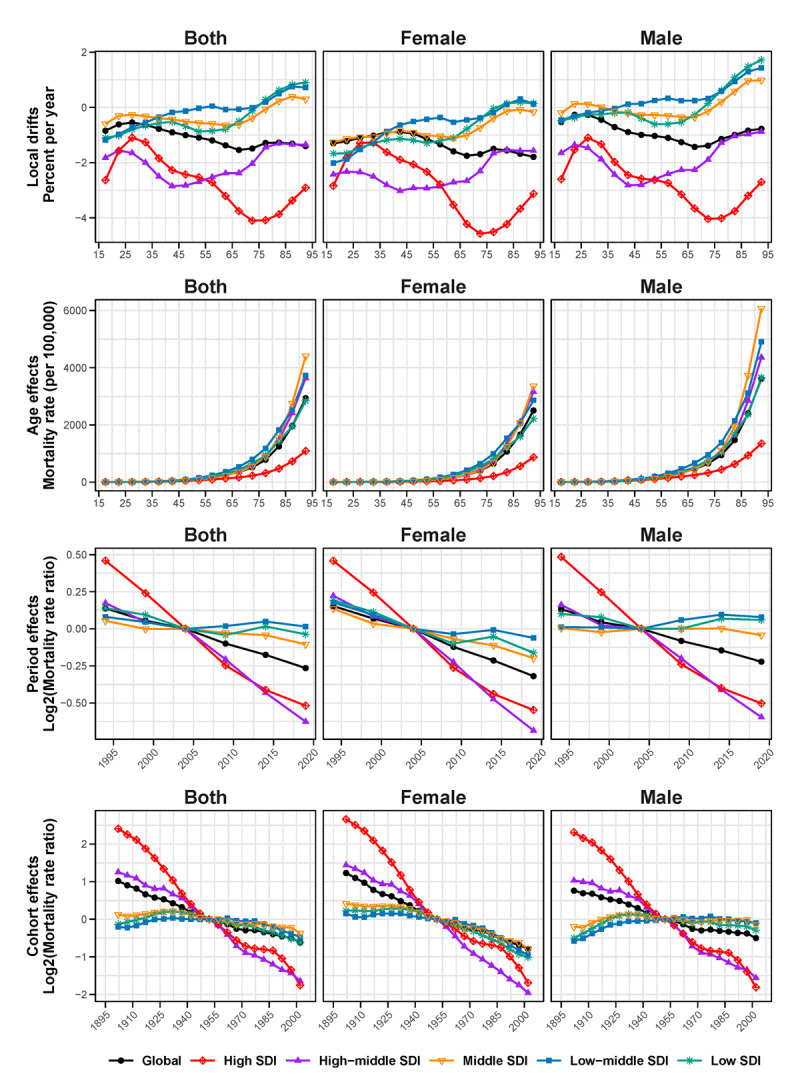
Age, period, and cohort effects on IHD mortality rates from 1990 to 2021 in different SDI regions and across both sexes. **(A)** shows the local drifts (% per year) by age group, **(B)** illustrates the age effects (mortality rates per 100,000 population), **(C)** presents the period effects (log2 mortality rate ratios over time), and **(D)** depicts the cohort effects (log2 mortality rate ratios by birth cohort). Each panel includes data for both sexes, males, and females. *IHD, Ischemic heart disease; SDI, socio-demographic index*.

Figure 3 and Tables S8–S11 present estimates for age, period, cohort effects, and local drift on IHD mortality by SDI quintile. Local drift analysis revealed an overall downward trend in mortality rates across all age groups globally, with significant variations between SDI regions. High SDI regions showed the most pronounced changes, with substantial decreases across all age groups, particularly for those aged 60 and above. The most significant annual percentage decrease of 4.10% was observed in the 72.5 years age group. In contrast, low SDI and low-middle SDI regions exhibit an upward trend among individuals aged 75 and above, with the increase being more pronounced among males.

Age effects analysis demonstrated that IHD mortality rates increased exponentially with age across all SDI regions. Low SDI regions showed the steepest increase, particularly for females aged 60 and above. In contrast, high SDI regions exhibited the lowest IHD mortality rates across all age groups, with rapid increases primarily concentrated after age 75. The age curves showed that the inflection point occurred at 62.5 years in low SDI regions, while it was observed at 77.5 years in high SDI regions.

Period effects analysis indicated an overall declining trend globally, but with significant variations across different SDI regions. High and high-middle SDI regions demonstrated the most pronounced changes, with substantial decreases in IHD mortality risk. In high SDI regions, the relative risk decreased from 1.38 in 1994 to 0.70 in 2019, compared to the reference year 2004. In contrast, middle-low and low SDI regions showed more modest changes. Notably, these regions experienced an upward trend among males during the 2009 and 2014 periods.

Cohort effects analysis also revealed an overall declining trend globally. High and high-middle SDI regions exhibited the most significant downward trends. In high SDI regions, the relative risk decreased dramatically from 5.31 for the 1902 birth cohort to 0.30 for the 2002 cohort, compared to the reference 1952 birth cohort. In comparison, middle, low-middle, and low SDI regions showed more modest changes. Gender disparities were particularly evident in these regions: females showed a slow downward trend, while males exhibited an upward trend. In low SDI regions, the relative risk for female cohorts decreased from 1.17 for the 1902 cohort to 0.50 for the 2002 cohort, while for males, it increased from 0.70 to 0.82 over the same period.

### Frontier analysis

**Figure 4:**
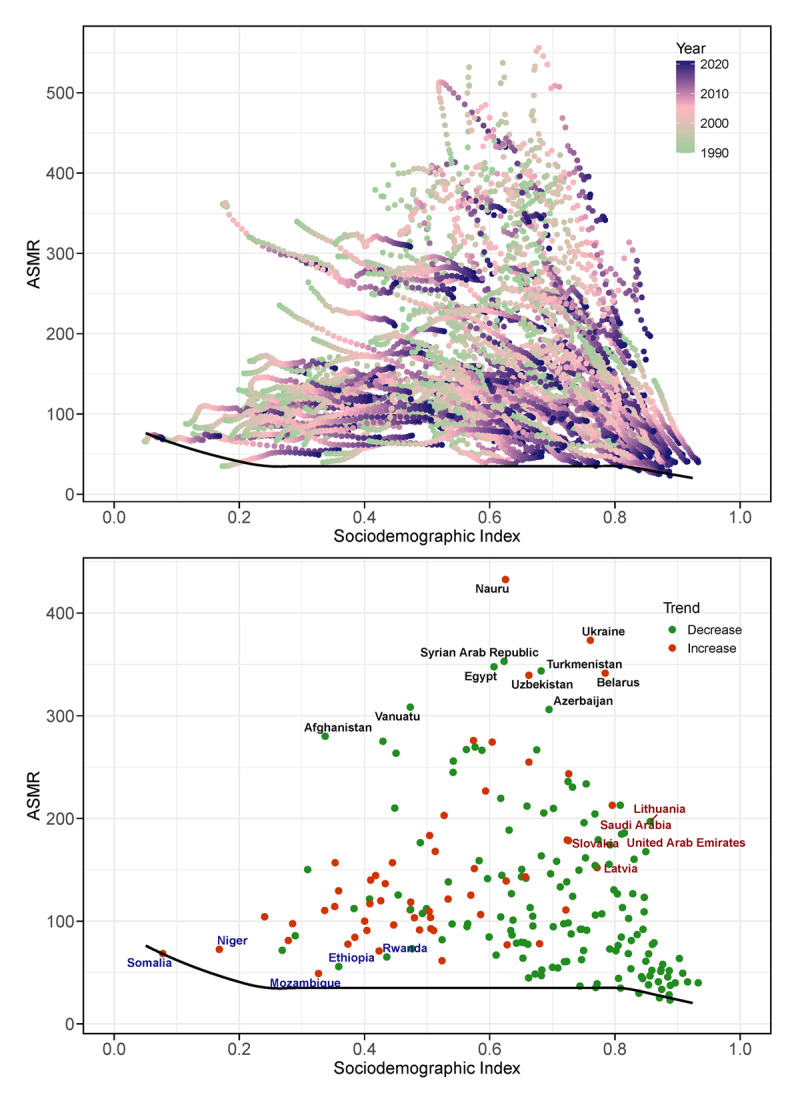
Relationship between the SDI and ASMR for IHD from 1990 to 2021. The top panel shows ASMR (per 100,000 population) against SDI for 204 countries and territories. The bottom panel displays the effective difference trend line, indicating the gap between observed and potentially achievable ASMR in 2021 across the same countries and territories. *ASMR, age-standardized mortality rate; IHD, Ischemic heart disease; SDI, socio-demographic index*.

Figure 4 and Table S12 present the results of the frontier analysis, revealing that many countries demonstrated a general decrease in ASMR from 1990 to 2021. However, some countries, particularly those with lower SDI values, exhibited an increase in ASMR. As SDI values increased, ASMR tended to converge toward lower levels. However, significant variations were observed even among countries with similar SDI levels. The countries with the highest effective differences included Nauru, Ukraine, the Syrian Arab Republic, Egypt, Turkmenistan, Belarus, Uzbekistan, Vanuatu, Azerbaijan, and Afghanistan. These countries exhibit the greatest potential for reducing IHD mortality given their current levels of development. Notably, some higher SDI countries, such as Lithuania, Saudi Arabia, Slovakia, the United Arab Emirates, and Latvia, also displayed substantial distances from the frontier. Conversely, low SDI regions such as Somalia, Niger, Mozambique, Ethiopia, and Rwanda were found to be closest to the frontier, showcasing relatively good performance in IHD mortality control despite socioeconomic constraints.

### Decomposition and risk factor analysis

**Figure 5:**
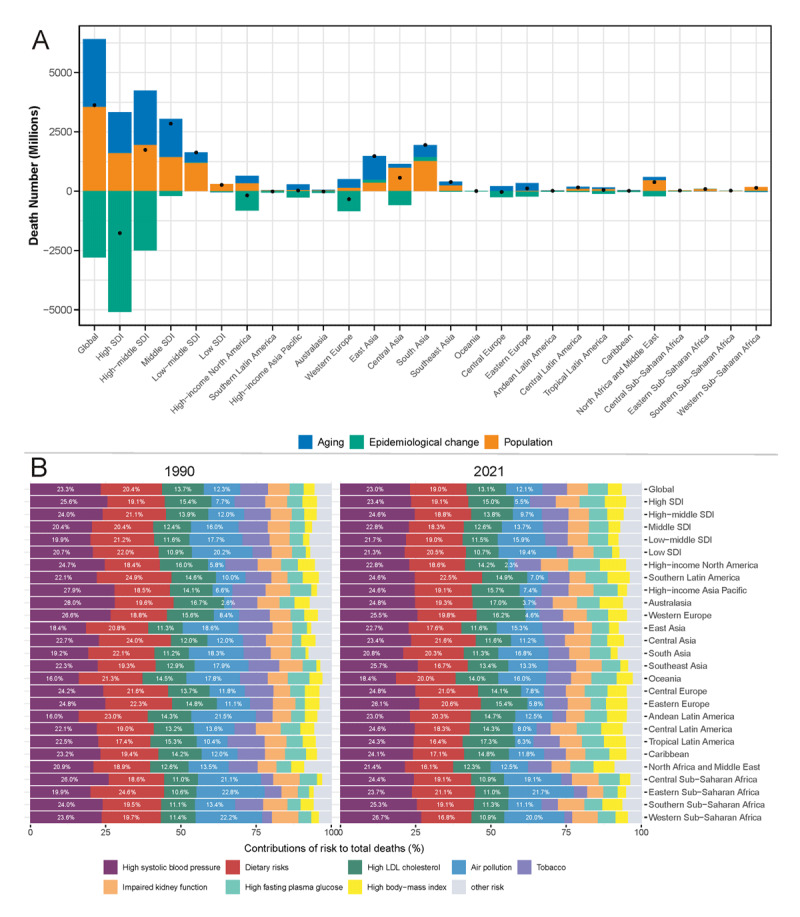
**(A)**. Contributions of aging, epidemiological change, and population growth to changes in IHD mortality from 1990 to 2021 across different SDI and GBD regions. **(B).** Risk factor contributions to total IHD deaths in 2021 across different SDI and GBD regions. *GBD, Global Burden of Disease; IHD, Ischemic heart disease; SDI, socio-demographic index*.

Decomposition analysis provides crucial insights into the driving factors behind the changes in IHD mortality across different SDI regions and GBD regions (Figure 5 A and Table S13). Globally, population growth emerged as the most significant factor contributing to the increase in IHD mortality. High SDI regions demonstrated the most notable epidemiological improvements, with advancements in prevention, treatment, and healthcare systems outweighing the effects of population aging and growth, resulting in a net decrease in deaths. In contrast, middle SDI regions experienced the largest increase in death numbers, primarily due to population aging coupled with less substantial improvements in epidemiological factors.

Regionally, South Asia and East Asia emerged as the two areas with the largest increases in IHD deaths. South Asia’s growth was primarily driven by population increase, while East Asia’s was mainly attributed to population aging. These two regions exhibited a unique pattern where epidemiological changes did not reduce deaths but instead contributed to an increase in IHD mortality. In contrast, high-income North America and Western Europe experienced a decline in IHD deaths, primarily due to substantial epidemiological improvements that surpassed the effects of aging and population growth.

Figure 5B and Table S14 demonstrate that in 2021, high systolic blood pressure was the primary risk factor for IHD mortality both globally and across all SDI regions. From 1990 to 2021, the contribution of high systolic blood pressure to IHD mortality increased in all SDI regions except for the high SDI region. Dietary risks (20.4% to 19.0%) and high low-density lipoprotein (LDL) cholesterol (13.7% to 13.1%) remained the second and third leading factors contributing to IHD mortality globally. However, in low, low-middle, and middle SDI regions, although the contribution of air pollution decreased, it remained the third highest risk factor in these areas. Notably, the contribution of high fasting plasma glucose and high body-mass index increased across all SDI regions. The high SDI region showed the most significant growth, with high fasting plasma glucose increasing from 5.1% to 8.5%, and high body-mass index from 4.9% to 7.2%.

Regionally, except for high-income North America and high-income Asia Pacific, the proportion of high systolic blood pressure risk increased in other GBD regions. Additionally, high fasting plasma glucose and high body-mass index increased in all GBD regions, with high-income North America showing the most significant increase. Regarding air pollution, high-income North America’s air pollution decreased to the lowest level of 2.3%, while in regions like Asia and Africa, air pollution still accounted for over 10% of the risk, ranking third among risk factors. In Eastern Sub-Saharan Africa and Western Sub-Saharan Africa, it exceeded 20%.

### Projection from 2022 to 2036

**Figure 6:**
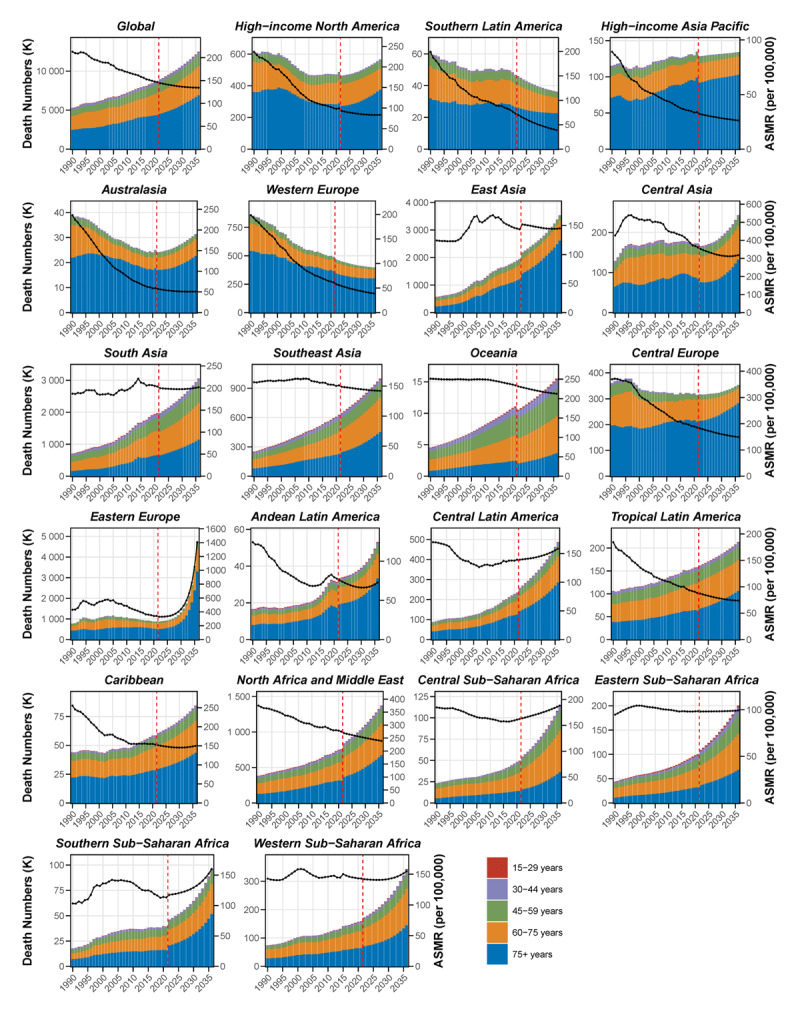
BAPC projections for 2022 to 2036, showing predicted death numbers (in thousands) and ASMR (per 100,000 population) across different GBD regions. ASMR, age-standardized mortality rate; BAPC, Bayesian Age-Period-Cohort; GBD, Global Burden of Disease

The global ASMR of IHD is projected to decrease by 8.5%, from 146.7 per 100,000 in 2021 to 134.3 per 100,000 by 2036, despite a gradual increase in the number of deaths, as shown in Figure 6 and Tables S15-S16. However, trends vary significantly across regions, Western Europe and Southern Latin America are projected to experience continued declines in both death numbers and ASMR. High-income North America, High-income Asia Pacific, North Africa and Middle East, Central Europe, Australasia, and most Asian regions are also expected to see decreases in ASMR, but the number of deaths in these regions will still increase, primarily due to deaths in the 75+ age group. In contrast, over the next 15 years, most regions in Africa are projected to experience increasing trends in both death numbers and ASMR. Southern Sub-Saharan Africa is projected to see a notable increase in ASMR, increasing from 113.3 to 160.0 per 100,000, a 41.2% increase. Notably, Eastern Europe is expected to see the most dramatic increase, with ASMR rising from 340.9 to 1,406.0 per 100,000, a 312.4% increase.

## Discussion

In this study, we conducted a comprehensive analysis of global IHD mortality trends from 1990 to 2021 and projection from 2022 to 2036. The overall decline in global ASMR for IHD is encouraging and reflects advancements in prevention, diagnosis, and treatment across many parts of the world. However, the persistent increase in absolute death numbers underscores the ongoing challenge posed by demographic changes, particularly population aging and growth. This trend aligns with previous studies on cardiovascular disease burden, highlighting the need for health systems to adapt to the growing prevalence of age-related chronic diseases (21,22,23).

The APC analysis revealed several critical findings. The exponential increase in IHD mortality with age underscores the biological vulnerability to cardiovascular disease with advancing age (24). In high SDI regions, both the inflection point of the age curve and the most significant decrease in local drift occurred at 72.5 years. The inflection point in low SDI regions was 15 years earlier than in high SDI regions. This suggests that while mortality rates are declining across all ages, future interventions in high SDI regions should focus increasingly on age groups older than this inflection point age (75 years+). In contrast, low SDI regions should implement early intervention and prevention strategies targeting middle-aged populations (25).

Interestingly, while high SDI regions consistently showed the highest male-to-female ASMR ratio from 1990 to 2021, the period and cohort effects revealed greater gender disparities in ASMR trends in low and low-middle SDI regions. In these areas, females demonstrated more significant improvements in ASMR over time, while males showed relatively small improvement or even worsening trends. Potential explanations include differences in healthcare-seeking behaviors, gender disparities in access to medical services, and variations in risk factor profiles between men and women across different socioeconomic contexts (26). In high SDI regions, despite high medical standards and continuous improvements, the highest male-to-female mortality ratios may reflect multiple sociocultural determinants. Male reluctance to seek healthcare, particularly for preventive services and early symptom recognition, likely contributes to persistently higher male mortality rates. Work-related stress and risk-taking behaviors, including higher rates of smoking and alcohol consumption among men in developed countries, may compound these healthcare-seeking disparities. Occupational factors represent another critical dimension, as men are more likely to work in industries with higher cardiovascular risk exposure, including shift work, physical labor, and environments with air pollution or chemical exposures. These factors partially offset the improvements in healthcare advances and progress in reducing ASMR. Additionally, women’s greater access to education, economic empowerment, and preventive healthcare contributes to the substantial decline in female ASMR observed in high SDI regions.

Conversely, in low and middle SDI regions, barriers to healthcare access for women may include economic constraints, family responsibilities that prioritize children’s health needs, and cultural restrictions that limit women’s ability to seek medical services independently. These barriers result in smaller gender disparities in low SDI regions compared to high SDI regions, where healthcare access is more equitable but behavioral and occupational factors become more prominent determinants. Notably, the decline in female ASMR across all SDI levels may be associated with female underdiagnosis of IHD. Women often present with atypical symptoms that may be misinterpreted by both patients and healthcare providers, leading to delayed diagnosis and treatment. This underdiagnosis problem is particularly pronounced in lower SDI regions, where healthcare providers have limited awareness of gender-specific IHD presentations and diagnostic resources are scarce. These findings suggest a need for further investigation and targeted interventions for males in lower SDI regions, while addressing the persistent underdiagnosis issues affecting women across all development contexts.

The decomposition analysis revealed that South Asia and East Asia had the largest increases in IHD deaths. In these regions, epidemiological changes did not reduce deaths but instead led to an increase. This indicates effective policies should be developed to address the growing population and aging in these rapidly developing regions (5,27,28). Risk factor analysis revealed that high systolic blood pressure is the primary risk factor for IHD mortality globally and across all SDI regions, emphasizing the continued importance of hypertension control (29,30). In low, low-middle, and middle SDI regions, despite some reduction in its contribution over time, air pollution persisted as the third highest risk factor. This highlights the need to address environmental health issues in these regions (31,32). Additionally, the increasing contributions of high fasting plasma glucose and high body-mass index, particularly in high SDI regions, highlight the growing impact of diabetes and obesity on IHD mortality. These trends align with global increases in metabolic risk factors reported in recent studies (9,33,34).

The frontier analysis revealed significant variations in IHD mortality rates even among countries with similar SDI levels, indicating that factors beyond socioeconomic development play crucial roles in determining IHD mortality outcomes. Our analysis revealed that countries such as San Marino, Japan, South Korea, and France achieved the lowest ASMR rates, while Ukraine, Belarus, Egypt, and Syria demonstrated substantially higher rates. Several Sub-Saharan African countries, including Kenya, Lesotho, and Zimbabwe, showed concerning upward trends in IHD mortality. The exceptional performance of Japan and South Korea likely reflects comprehensive healthcare policies emphasizing prevention and early intervention. Japan’s and South Korea’s universal health insurance systems provide accessible cardiovascular screening and management, while cultural dietary patterns rich in fish and vegetables may contribute to lower cardiovascular risk profiles. Additionally, Japan’s and South Korea’s National Health Screening Programs include mandatory cardiovascular risk assessments, facilitating early detection and management of hypertension and dyslipidemia. Both countries have implemented stringent tobacco control policies and maintain relatively low smoking prevalence rates compared to global averages (35,36,37). In contrast, the elevated ASMR rates in Eastern European countries like Ukraine and Belarus may reflect healthcare system disruptions following political transitions, limited access to modern cardiovascular medications, and higher prevalence of behavioral risk factors including smoking and alcohol consumption. The ongoing conflict in Ukraine has further compromised healthcare infrastructure and access to cardiac care services. Similarly, countries in the Middle East and North Africa face challenges related to healthcare system capacity, economic instability, and rapidly changing lifestyle patterns associated with urbanization (38,39).

The rising trends in Sub-Saharan African countries present a complex epidemiological picture. Kenya, Lesotho, and Zimbabwe are experiencing epidemiological transitions characterized by increasing prevalence of cardiovascular risk factors including hypertension, diabetes, and obesity, while healthcare systems remain primarily focused on infectious disease management. Limited availability of cardiovascular specialists, lack of affordable cardiac medications, and inadequate emergency cardiac care infrastructure contribute to poor IHD outcomes. Healthcare system characteristics such as primary care organization, specialist accessibility, emergency response capabilities, and medication availability significantly influence IHD outcomes. Population-level interventions including tobacco control policies, food safety regulations, urban planning approaches that promote physical activity, and air quality management represent critical determinants of national IHD mortality patterns (40,41).

Our projections for 2036 present a complex picture of the future global IHD burden. While ASMR is expected to decline in most regions globally, the significant anticipated increase in deaths among those aged 75 and older, driven by population aging, poses major challenges for healthcare systems worldwide. The rising ASMR trends in Eastern Europe and African regions highlight concerning patterns that demand urgent attention and targeted interventions.

Our study has several limitations. First, our analysis is constrained by the inherent limitations of the GBD database, particularly the incomplete primary data from many low and middle-income countries, which may lead to underestimation of IHD mortality in these regions. The GBD methodology’s reliance on vital registration systems and statistical modeling to address data gaps introduces uncertainty, especially in settings with limited health information infrastructure. This potentially affects the accuracy of temporal trends and cross-regional comparisons in these areas. Second, the GBD methodology’s use of mutually exclusive causes and single underlying cause of death attribution may underestimate actual IHD mortality. This occurs because patients frequently present with multiple cardiovascular complications (e.g., heart failure) and comorbidities (e.g., diabetes, chronic kidney disease), particularly in older populations where multimorbidity is common. Third, long-term predictions inherently carry substantial uncertainty as they assume the continuation of historical age-period-cohort trends observed from 1990–2021. These trends may be disrupted by unforeseen events such as economic crises, pandemics, climate change impacts, or transformative medical innovations and policy interventions. Consequently, our projections should be interpreted as scenario-based estimates contingent upon current patterns persisting, rather than definitive forecasts. Their principal value lies in informing proactive public health strategies designed to mitigate projected risks and alter future trajectories. Fourth, our national-level analysis may mask important within-country inequalities such as urban-rural or ethnic differences in IHD mortality. Additionally, socioeconomic stratification within countries creates differential access to preventive care and treatment, which our aggregate national estimates cannot capture. Finally, our frontier analysis assumes countries can achieve mortality rates similar to their most efficient peers, which may not account for unique contextual constraints including geographic factors, political instability, climate-related health challenges, or governance capacity limitations.

## Conclusions

In conclusion, our study provides a comprehensive analysis of global IHD mortality trends from 1990 to 2021, along with projections to 2036, revealing the complex landscape of cardiovascular health worldwide. Our findings emphasize that the global focus on hypertension control strategies remains essential. In high SDI regions, the priority should be on providing comprehensive care for older adults, particularly those aged 75 and above, with multiple comorbidities, and managing metabolic risk factors such as diabetes and obesity. Conversely, low and middle SDI regions need to strengthen their healthcare systems to address the growing burden of IHD, implement early intervention and prevention strategies targeting middle-aged populations, and increase efforts to address air pollution. By 2036, IHD mortality rates are projected to continue their upward trend in Eastern Europe and African regions, demanding immediate attention and targeted interventions.

## Nonstandard Abbreviations and Acronyms

APC Age-period-cohort

ASMR Age-standardized mortality rates

BAPC Bayesian age-period-cohort

EAPC Estimated annual percentage change

GBD Global Burden of Disease

ICD International Classification of Diseases

IHD Ischemic heart disease

IHME the Institute for Health Metrics and Evaluation

LDL Low-density lipoprotein

SDI Socio-demographic index

UIs Uncertainty intervals

## Data Accessibility Statement

All data are publicly available and can be accessed through the GBD Results Tool website (http://ghdx.healthdata.org/gbd-results-tool).

## Additional File

The additional file for this article can be found as follows:

10.5334/gh.1486.s1 Supplementary files. Tables S1–S16 and Figures S1–S2.
