# Supplementary material for: Global Trends in Ischemic Heart Disease Mortality from 1990 to 2021 and 2036 Projections: Insights from GBD 2021 Data
Source: Glob Heart. 2025 Oct 10;20(1):92. doi: 10.5334/gh.1486 (PMC12513345; doi:10.5334/gh.1486)
Supplement: Supplementary files. — Tables S1–S16 and Figures S1–S2. [file gh-20-1-1486-s1.pdf]

Figure S1

Click here to access/download;Supplementary Files;Figure S1.tif

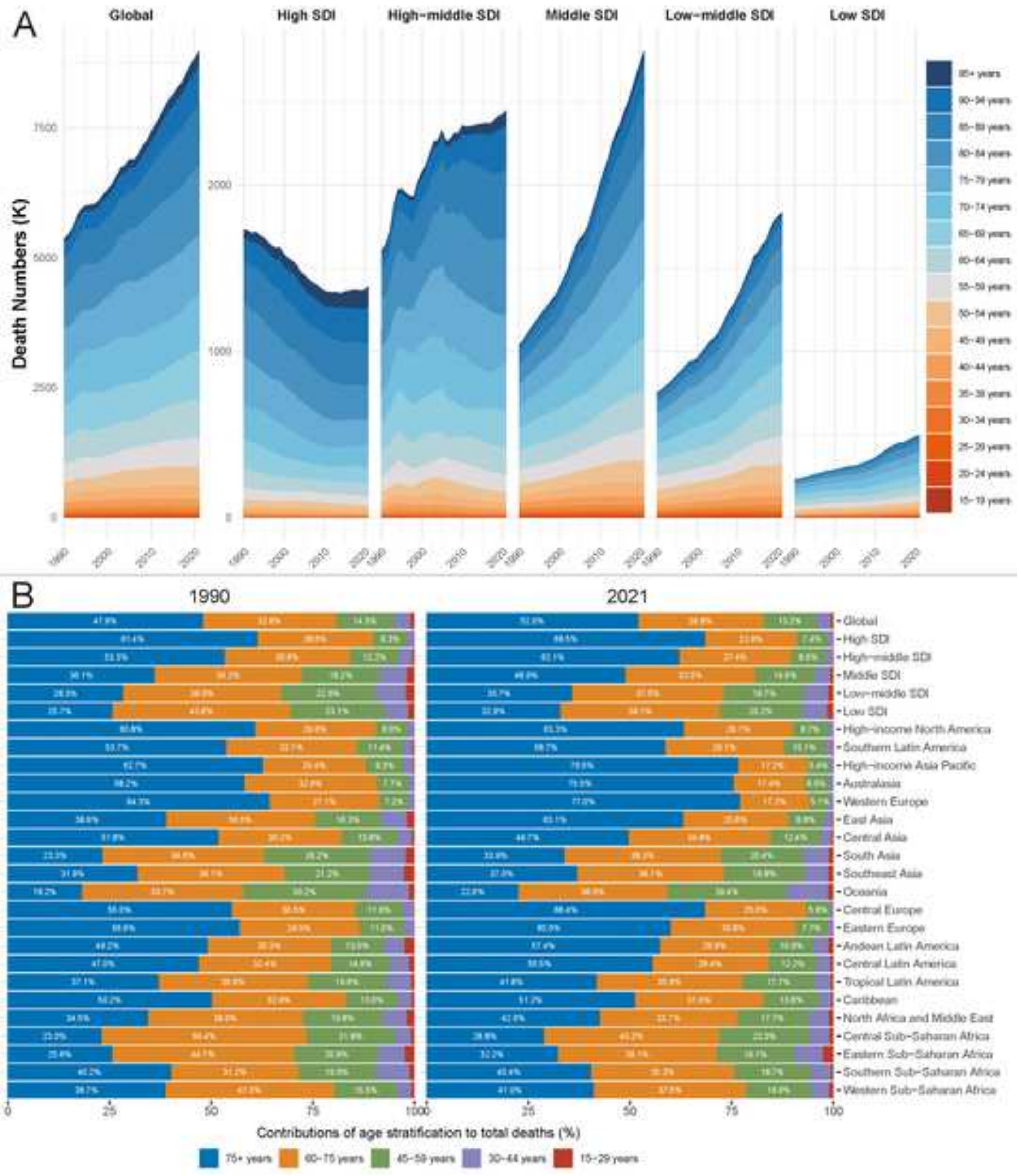

Figure S2

[Click here to access/download;Supplementary Files;Figure S2.tif]

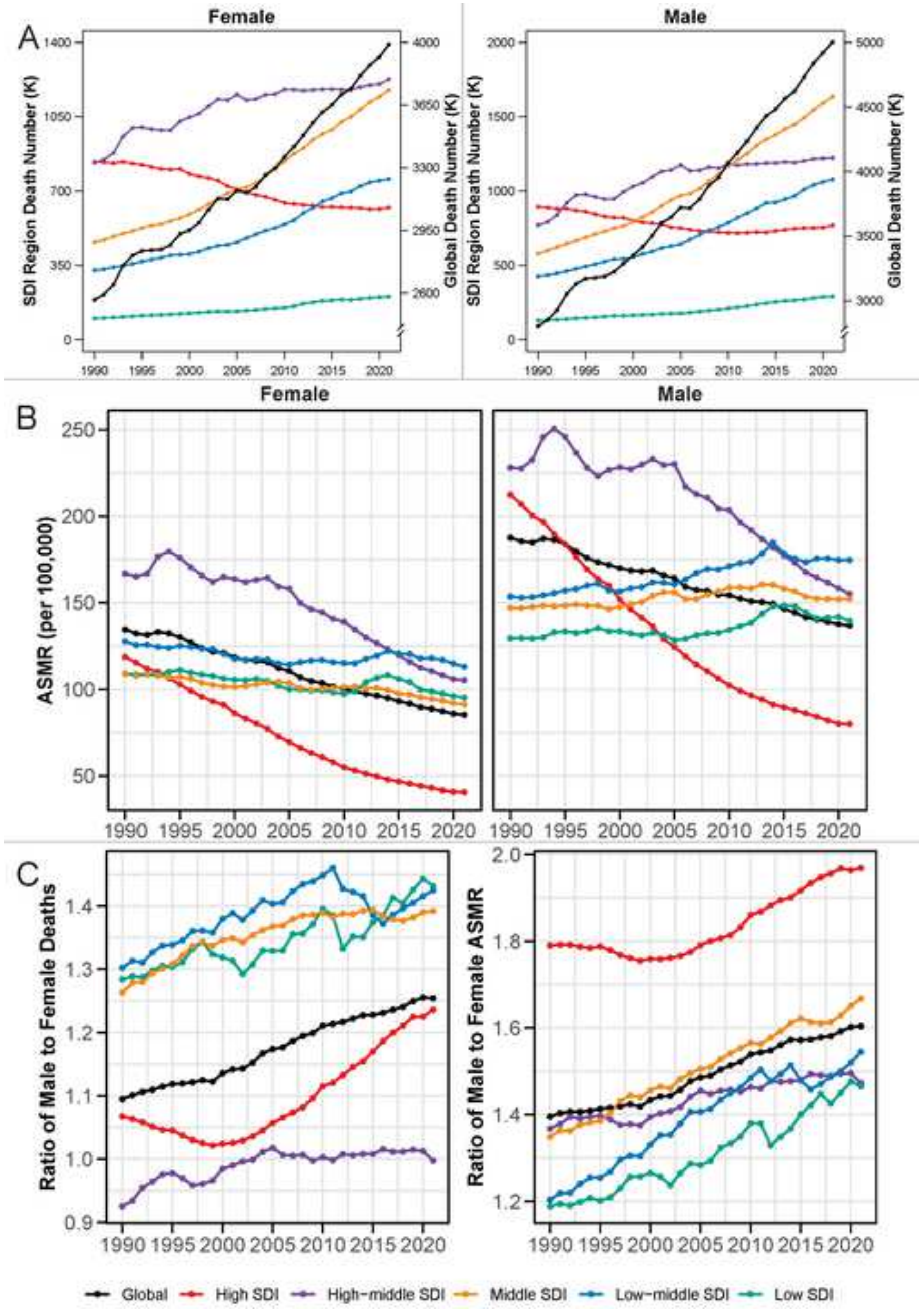

Supplemental Methods

CONTENTS

Age-Period-Cohort Analysis..1

Frontier Analysis 2

Decomposition Analysis 3

Bayesian Age-period-cohort Analysis 4

Supplemental Methods

Age-Period-Cohort Analysis

We employed the Age-Period-Cohort (APC) model to investigate temporal trends in ischemic heart disease (IHD) mortality. The APC model is a multidimensional analytical approach that decomposes disease trends into three key components: age effects, which reflect how the risk of a disease changes with age; period effects, which capture temporal variations that affect all age groups simultaneously; and cohort effects, which represent variations in disease risk associated with different birth cohorts. This approach allows us to address key questions about changes in IHD mortality rates over time, differences across age groups, and variations among birth cohorts. The APC analysis effectively separates the independent effects of these intertwined factors, overcoming limitations of traditional multifactor regression analysis.

We utilized IHD mortality and population data estimates from the Global Burden of Disease (GBD) Study 2021. In this model, the groups younger than 15 years and groups older than 95 years were excluded. The data structure included six 5-year periods (1992-1996 to 2017-2021), sixteen 5-year age groups (15-19 to 90-94 years), and twenty-one 5-year birth cohorts (1900-1904 to 2000-2004). The analysis was conducted using the APC Web Tool provided by the National Cancer Institute (NCI) (https://analysistools.cancer.gov/apc/). This tool was chosen for its ability to address the "identifiability problem" in APC models, which arises from the linear relationship among age, period, and cohort. The APC model could be expressed as follows::

$$\log(E[r_{ij}]) = \mu + \alpha_i + \beta_j + \gamma_k$$

Where:

- r_{ij} is the mortality rate for age group i in period j.
- μ is the intercept.
- α_i is the age effect for age group i.

- β_j is the period effect for period j.
- γ_k is the cohort effect for cohort k (where $k = j - i + 1$).

The APC Web Tool employs several statistical methods to analyze the data. It uses weighted least squares with a Poisson distribution assumption to estimate parameters, effectively handling the characteristics of mortality data, including potential overdispersion. To resolve the inherent identification problem, the tool uses the Intrinsic Estimator (IE) method. This approach imposes additional constraints on the model parameters, such as the sum of coefficients for each of the age, period, and cohort groups equaling zero, to obtain unique estimates.

The APC Web Tool generates several key outputs, providing comprehensive insights into IHD mortality trends.

1. Net Drift: Indicates the overall annual percentage change in mortality rates over time, reflecting long-term trends.
2. Local Drifts: Show the annual percentage change in mortality rates for each specific age group, allowing identification of trend differences across age groups.
3. Age Effect: Represented as the longitudinal age-specific rates in the reference cohort, adjusted for period effects. This reflects the relative risk across different age groups.
4. Period Effect: Expressed as the mortality rate relative risk for each period compared to the reference period (set as 2000-2004 in this study). This reflects factors affecting all age groups at specific time points.
5. Cohort Effect: Presented as the mortality rate relative risk for each cohort compared to the reference cohort (set as the 1946-1954 birth cohort in this study). This reflects risk differences among different birth groups.

Frontier Analysis

To evaluate the relationship between IHD mortality and socio-demographic development, we applied a frontier analysis as a quantitative methodology. This approach aimed to identify the lowest potentially achievable age-standardized IHD mortality rate based on development status, as measured by the Socio-demographic Index (SDI). The IHD mortality frontier pinpoints the minimum mortality rate that could be attained for every country or territory given its SDI. We utilized data from all countries and territories from 1990 to 2021, encompassing a 32-year period.

We employed a data envelope analysis, which allows for the delineation of non-linear frontiers, using the free disposal hull method to produce a frontier for age-adjusted IHD mortality rates by SDI. To ensure robust estimation of the frontier and account for uncertainty, we employed a bootstrap resampling technique with 100 resamples. This involved randomly sampling with replacement from all countries and territories across all years within the study period.

To understand the relationship between age-standardized IHD mortality rates and the frontier in 2021, we calculated the "effective difference" - the absolute distance from the frontier - using 2021 SDI and age-standardized IHD mortality rate data points for each country or territory. Countries or territories with lower mortality rates than the frontier were assigned a zero distance. The effective difference quantifies the gap between observed and potentially achievable IHD mortality burden. A large effective difference from the frontier suggests there may be unrealized opportunities for reduction in IHD mortality that should be possible based on the country or territory's place on the development spectrum. This analysis allows us to identify countries that are performing better or worse than expected given their level of socio-demographic development, and to quantify the potential for improvement in IHD mortality rates across different contexts over the 32-year study period.

Decomposition Analysis

We employed the decomposition methodology of Das Gupta to decompose IHD mortality by population age structure, population growth, and epidemiologic changes. The mortality at each location was obtained from the following formula:

$$\text{Mortality}_{a_y,p_y,e_y} = \sum(a_{i,y} \times p_y \times e_{i,y})$$

Where $\text{Mortality}_{a_y,p_y,e_y}$ represented mortality based on the factors of age structure, population, and mortality rate for specific year y; $a_{i,y}$ represents the proportion of population for the age category i of the 17 age categories (ranging from 15 to 95+ years) in given year y; p_y represents the total population in given year y; and $e_{i,y}$ represents mortality rate given age category i in year y.

The contribution of each factor to the change in mortality from 1990 to 2021 was defined by the effect of one factor changing while the other factors were held constant. For example, the effect of age structure was calculated as:

[(Mortality a2021, p1990, e1990 + Mortality a2021, p2021, e2021)/3 + (Mortality a2021, p1990, e2021 + Mortality a2021, p2021, e1990)/6] - [(Mortality a1990, p2021, e2021 + Mortality a1990, p1990, e1990)/3 + (Mortality a1990, p2021, e1990 + Mortality a1990, p1990, e2021)/6]

This decomposition analysis allows us to quantify the contribution of demographic changes (age structure and population growth) and epidemiological changes to the trends in IHD mortality over the study period, providing insights into the driving factors behind the observed changes in IHD burden across different regions and the 17 age groups from 15 to 95+ years.

Bayesian Age-period-cohort Analysis

The Bayesian age-period-cohort (BAPC) model was employed to project IHD death cases and mortality rates from 2022 to 2036. This statistical approach analyzes and forecasts trends in disease burden by incorporating the effects of age, time period, and birth cohort. The BAPC model assumes that the observed mortality counts y_{ij} in age group i at time point j follow a Poisson distribution with mean $n_{ij}\lambda_{ij}$, where n_{ij} is the corresponding person-time of exposure. The linear predictor $\eta_{ij} = \log(\lambda_{ij})$ is specified as

$\log(\lambda_{ij}) = \mu + \alpha_i + \beta_j + \gamma_k$, where μ represents the general level (intercept), and α_i, β_j, γ_k denote age, period, and cohort effects, respectively.

Smoothing priors are used for age, period, and cohort effects. We employed a second-order random walk (RW2) prior, which assumes independent mean-zero normal distributions with unknown variance on the second differences of all time effects. This approach penalizes deviations from a linear trend and is considered the discrete-time analogue of a cubic smoothing spline. For Bayesian inference, we utilized integrated nested Laplace approximations (INLA), which provide numerical approximations for the posterior marginal distributions. This method offers computational efficiency and avoids issues related to Markov chain Monte Carlo (MCMC) convergence. To project future mortality rates, we extrapolated the period and cohort effects following the structure of the RW2 model. For t periods ahead into the future, the projected rate is given by $\log(\lambda_{iJ+t}) = \mu + \alpha_i + \beta_{J+t} + \gamma_{k+t} + z_{i,J+t}$, where $z_{i,J+t}$ represents additional independent mean-zero Gaussian random effects to adjust for overdispersion.

We implemented the model using the R package BAPC, which is a wrapper of the INLA package specific for APC analysis. This package facilitates model specification and offers specialized functions to visualize and extract output of interest. The BAPC package was used to generate probabilistic forecasts, providing both age-specific and age-standardized projected rates. Poisson noise was automatically added for the predictive distribution, ensuring that the projections account for both the underlying trends and the inherent variability in the data. For age-standardization, we used the global age-standard population from the World Health Organization. Population forecast data was obtained from the GBD Study 2019 Global Fertility, Mortality, Migration, and Population Forecasts 2017-2100. To assess the predictive quality of the model, we employed proper scoring rules, including the continuous ranked probability score (CRPS) and calibration tests. These methods allow for the evaluation of both the accuracy and the reliability of the probabilistic forecasts. The CRPS assesses the sharpness and calibration of the predictive distributions, while the calibration tests examine whether the predicted probabilities match the observed frequencies.

Table S1. Definitions of Metrics and Measures Used in the Global Burden of Disease (GBD) 2021 Study.

Table S2. 2021 Socio-demographic Index (SDI) Classification Criteria and Corresponding Quintiles.

Table S3. Socio-demographic Index values for 204 countries and territories, 1990-2021.

Table S4. Trends in Global, SDI Regions, and GBD Regions Ischemic Heart Disease Mortality: Total Number of Deaths and Age-Standardized Mortality Rate (ASMR), 1990-2021.

Table S5. Trends in Age-Standardized Mortality Rate (ASMR) for Ischemic Heart Disease Across 204 Countries and Territories: EAPC and Net Drift Analysis, 1990-2021.

Table S6. Shifts in Age Distribution of Ischemic Heart Disease (IHD) Deaths by SDI Region: Total Number and Proportion of Deaths Across Age Groups, 1990-2021.

Table S7. Trends in Sex-Specific Age-Standardized Mortality Rates (ASMR) for Ischemic Heart Disease Across Global and SDI Regions: Male-to-Female Ratio Analysis, 1990-2021.

Table S8. Local Drift Analysis of Ischemic Heart Disease Mortality: Age-Specific Percent Changes in Mortality Rates by Sex Across SDI Quintiles, 1990-2021.

Table S9. Age Effects on Ischemic Heart Disease Mortality Rates by Sex Across SDI Regions: Analysis of Rate Increases with Age, 1990-2021.

Table S10. Period Effects on Ischemic Heart Disease Mortality Rates: Rate Ratios by Sex and SDI Regions, 1990-2021.

Table S11. Cohort Effects on Ischemic Heart Disease Mortality Rates by Sex Across SDI Regions: Relative Risks Across Birth Cohorts, 1902-2002.

Table S12. Frontier Analysis of Ischemic Heart Disease Mortality and Socio-demographic Index (SDI): Effective Differences and Trends, 1990-2021.

Table S13. Decomposition Analysis of Ischemic Heart Disease Mortality: Contributions of Aging, Population Growth, and Epidemiological Changes Across SDI and GBD Regions, 1990-2021.

Table S14. Risk Factor Analysis of Ischemic Heart Disease Mortality: Contributions of Top 8 Risk Factors by Region in 1990 and 2021.

Table S15. Bayesian APC Projections of Ischemic Heart Disease Mortality: Projected Number of Deaths Across Regions, 2022-2036.

Table S16. Bayesian APC Projections of Age-Standardized Mortality Rates (ASMR) for Ischemic Heart Disease Across Regions, 2022-2036.

Table S1. Definitions of Metrics and Measures Used in the Global Burden

Measure	Number
**Deaths**	Number of deaths in the population
**Disability adjusted life years (DALYs)**	Number of DALYs in the population
**Years of life lost (YLLs)**	Number of YLLs in the population
**Years lived with disability (YLDs)**	Number of YLDs in the population
**Prevalence**	Total number of cases in the population
**Incidence**	Number new of cases in the population
**Maternal mortality ratio (MMR)**	n/a
**Probability of death**	n/a
**Life expectancy**	n/a
**Healthy life expectancy (HALE)**	n/a
**Summary exposure value (SEV)**	n/a

NOTE: These apply only to measures in the GBD Results Tool and GBD Compa

of Disease (GBD) 2021 Study.

Metric definitions		
**Percent**	**Rate**	**Years**
Proportion of deaths for a particular cause relative to deaths from all causes	Deaths per 100,000 population	n/a
Proportion of DALYs for a particular cause relative to DALYs for all causes	DALYs per 100,000 population	n/a
Proportion of YLLs for a particular cause relative to YLDs for all causes	YLLs per 100,000 population	n/a
Proportion of YLDs for a particular cause relative to YLDs for all causes	YLDs per 100,000 population	n/a
Proportion of total cases of a particular cause relative to cases from all causes	Total cases per 100,000 population	n/a
Proportion of news cases of a particular cause relative to cases from all causes	New cases per 100,000 population	n/a
n/a	Deaths per 100,000 live births	n/a
n/a	n/a	n/a
n/a	n/a	Years lived
n/a	n/a	Years lived
n/a	0 to 100, where 0 is no risk and 100 is the highest level of risk	n/a

ıre

**Probability of death**
n/a
n/a
n/a
n/a
n/a
n/a
n/a
Probability of dying due to a specific cause between a given age start and end, contingent upon being alive at age start
n/a
n/a
n/a

Table S2. 2021 Socio-demographic Index (SDI) Classification Criteria and Corresponding Quintiles.

location_id	location_name	lower_bound	upper_bound
44637	Low SDI	0	0.46581580319161997
44636	Low-middle SDI	0.46581580319161997	0.6188294452454329
44639	Middle SDI	0.6188294452454329	0.7119746219361235
44634	High-middle SDI	0.7119746219361235	0.8102959891918925
44635	High SDI	0.8102959891918925	1

Table S3. Socio-demographic Index values for 204 countries and territories, 1990-2021.

Location	1990	1991	1992	1993	1994	1995
Afghanistan	0.173832165	0.17647255	0.179633724	0.180183713	0.17851866	0.178279128
Albania	0.5577733	0.555629412	0.553107897	0.552453328	0.553763669	0.557040889
Algeria	0.460486906	0.468319197	0.475950937	0.483255591	0.49045066	0.497957543
American Samoa	0.613633924	0.615847612	0.618583654	0.622078598	0.626053241	0.629814054
Andorra	0.76146388	0.764709071	0.767188282	0.768953828	0.770023765	0.770988683
Angola	0.270736935	0.275160625	0.2792315	0.281867703	0.284487594	0.287447813
Antigua and Barbuda	0.612104591	0.618817129	0.624695818	0.630075854	0.634768955	0.638008629
Argentina	0.587397284	0.59170754	0.597996324	0.603739312	0.60978733	0.614473706
Armenia	0.544414535	0.547561863	0.548974724	0.551214674	0.553783844	0.556815513
Australia	0.725982519	0.729759859	0.733901404	0.738374743	0.742846572	0.747426824
Austria	0.749853693	0.751652734	0.754375286	0.758804554	0.764704364	0.769827471
Azerbaijan	0.595986033	0.595807712	0.594651335	0.593542676	0.592088785	0.589441457
Bahamas	0.693509268	0.689126784	0.687343838	0.693816501	0.706819892	0.719381607
Bahrain	0.584578852	0.590596897	0.596040229	0.602165622	0.608133725	0.613850722
Bangladesh	0.228548934	0.237207413	0.245247956	0.25209072	0.258997294	0.265293918
Barbados	0.653582517	0.656654073	0.662446635	0.668815012	0.673409365	0.676012247
Belarus	0.622446576	0.627038267	0.632891737	0.639394169	0.644652781	0.648959504
Belgium	0.737390656	0.742173074	0.748012299	0.753685087	0.758903638	0.763170401
Belize	0.423726992	0.433834392	0.444198507	0.455258577	0.465729308	0.475278511
Benin	0.218907154	0.222539019	0.226323465	0.230312594	0.234032036	0.238009696
Bermuda	0.696451196	0.700276851	0.703984095	0.707678222	0.711068998	0.714468828
Bhutan	0.21503985	0.22131244	0.228148272	0.236004687	0.244331246	0.253291957
Bolivia (Plurinational State	0.423917961	0.429397795	0.434835542	0.440601646	0.4468225	0.453479243
Bosnia and Herzegovina	0.54113254	0.541346251	0.539303227	0.536031882	0.53385339	0.534025536
Botswana	0.418077748	0.429752286	0.440490843	0.450523614	0.459954967	0.469266513
Brazil	0.500070509	0.504784798	0.508539943	0.512081793	0.515760842	0.519685963
Brunei Darussalam	0.666081917	0.671233831	0.676702526	0.68217544	0.687394093	0.692471156
Bulgaria	0.633446498	0.643069488	0.651186876	0.659161569	0.66743046	0.673999977
Burkina Faso	0.129695615	0.133299128	0.136594798	0.139825465	0.142848973	0.146115673
Burundi	0.20586736	0.20849572	0.210864619	0.213249386	0.215222973	0.215631676
Cabo Verde	0.276723367	0.282931722	0.288460371	0.294517286	0.302216794	0.310675634
Cambodia	0.289075059	0.2924792	0.296900955	0.301627202	0.305722592	0.310678881
Cameroon	0.303055334	0.309623458	0.315601269	0.320831654	0.325410123	0.329539318
Canada	0.781977864	0.78415936	0.787030537	0.789716593	0.792598518	0.796521166
Central African Republic	0.216825191	0.220191656	0.223007541	0.225451125	0.228487724	0.231696792
Chad	0.114638829	0.117954634	0.121132832	0.123600072	0.126080572	0.128289492
Chile	0.5864951	0.59300658	0.599106083	0.604828509	0.610673933	0.617234677
China	0.458668935	0.46730197	0.475784031	0.484353831	0.492578645	0.501500908
Colombia	0.480720054	0.482339894	0.484572359	0.488229373	0.493278182	0.499102757
Comoros	0.270048116	0.279145649	0.288506271	0.297607417	0.305706587	0.314220341
Congo	0.420654655	0.426734374	0.431987662	0.436606594	0.440168347	0.443744125
Cook Islands	0.564514854	0.570622776	0.577123468	0.584059175	0.591540868	0.598789968
Costa Rica	0.534125181	0.539342164	0.544624238	0.549946626	0.55517125	0.560547086
Croatia	0.668906358	0.6747246	0.678093133	0.678221047	0.677548133	0.677029465
Cuba	0.558019071	0.563413638	0.566169006	0.565441047	0.563186313	0.560765902
Cyprus	0.648230872	0.655726169	0.666011849	0.677547477	0.688829361	0.699923898
Czechia	0.681848021	0.688002859	0.697577809	0.710853416	0.725969686	0.737981722

Côte d'Ivoire	0.279320353	0.286492681	0.293451293	0.299743215	0.305210653	0.310105508
Democratic People's Repu	0.497780128	0.499120512	0.499629841	0.499700646	0.49953134	0.499080529
Democratic Republic of th	0.28984321	0.290431567	0.290959242	0.28962879	0.286859146	0.283967532
Denmark	0.801154655	0.80454035	0.808164434	0.811585713	0.815138614	0.819104612
Djibouti	0.337781789	0.338770011	0.340986061	0.344904394	0.347981404	0.351245633
Dominica	0.56360259	0.565237843	0.569570684	0.578811811	0.590119147	0.601986932
Dominican Republic	0.442654076	0.446144578	0.449953081	0.454229297	0.458620614	0.463313869
Ecuador	0.518430614	0.517679421	0.51837907	0.520707291	0.524908291	0.529380752
Egypt	0.417182742	0.426909452	0.437744555	0.448490053	0.458727004	0.468035368
El Salvador	0.373057975	0.374855325	0.377076643	0.380307326	0.385149094	0.391780266
Equatorial Guinea	0.268783629	0.275296992	0.283487764	0.292236032	0.30068634	0.310443551
Eritrea	0.216028239	0.222574737	0.230296066	0.239385041	0.250752541	0.261498485
Estonia	0.674967632	0.685223089	0.695586674	0.70369315	0.708456181	0.713172725
Eswatini	0.399420955	0.408269969	0.416683379	0.424802995	0.432503472	0.440127047
Ethiopia	0.148033885	0.14959971	0.150174386	0.151582364	0.152982637	0.155085605
Fiji	0.534648908	0.53877826	0.543188552	0.547776474	0.55284902	0.558530972
Finland	0.756221509	0.758410687	0.7608041	0.763526604	0.767101901	0.771163742
France	0.730747466	0.736452382	0.742871973	0.748582327	0.753176132	0.757125789
Gabon	0.455421187	0.461563098	0.467024048	0.472394463	0.478061947	0.483826218
Gambia	0.238714846	0.245010164	0.251087863	0.256996141	0.262424792	0.267696507
Georgia	0.656136044	0.662113186	0.665021428	0.663466862	0.658134139	0.651363604
Germany	0.817077666	0.823339898	0.828323503	0.832286809	0.835561963	0.837643152
Ghana	0.373112005	0.379239256	0.384856767	0.389968494	0.394953663	0.399930204
Greece	0.674186465	0.68031648	0.687055389	0.693328926	0.699153022	0.704862873
Greenland	0.732258252	0.730626532	0.729781103	0.729894842	0.730875896	0.730831638
Grenada	0.436734419	0.445887953	0.455910058	0.46599489	0.476410341	0.486558999
Guam	0.676220305	0.669974292	0.66635634	0.667318181	0.672111712	0.678120215
Guatemala	0.311792455	0.314329484	0.319165481	0.326840819	0.3334691	0.339642101
Guinea	0.178295421	0.181459391	0.184485016	0.187686242	0.191128837	0.194573245
Guinea-Bissau	0.207614839	0.212505984	0.217026088	0.221678978	0.226434933	0.231223526
Guyana	0.460430129	0.462677069	0.466753989	0.472174229	0.478992924	0.486573457
Haiti	0.31033463	0.315455441	0.320389979	0.324895851	0.328399456	0.332999152
Honduras	0.332042889	0.337034649	0.342305657	0.347838446	0.353164852	0.358787268
Hungary	0.649419991	0.653707155	0.660382905	0.667543556	0.675051406	0.683186412
Iceland	0.764212517	0.769027393	0.772706415	0.776389423	0.779967961	0.782740057
India	0.332593603	0.338591779	0.344591727	0.350685397	0.35699603	0.363418501
Indonesia	0.457134954	0.466505583	0.475753157	0.484775241	0.4936071	0.502008381
Iran (Islamic Republic of)	0.453799944	0.468982858	0.480626697	0.492277025	0.505072857	0.517531494
Iraq	0.412044173	0.415814116	0.420353594	0.424834933	0.428751731	0.432075545
Ireland	0.719891819	0.725195019	0.731918691	0.738384074	0.744011728	0.748921575
Israel	0.709178347	0.713371633	0.717897923	0.722250176	0.726654736	0.731127859
Italy	0.706255224	0.711457349	0.716649746	0.721720992	0.726870913	0.731644082
Jamaica	0.534781234	0.53959448	0.545088753	0.551218492	0.557643773	0.564315038
Japan	0.790253516	0.794616189	0.799156225	0.802972994	0.806689483	0.810510771
Jordan	0.539147468	0.542819411	0.547458846	0.55218235	0.557254335	0.562571536
Kazakhstan	0.589435804	0.590967832	0.596127664	0.603138328	0.6105911	0.618573582
Kenya	0.333850293	0.34047622	0.346298636	0.351611769	0.356779194	0.361535807
Kiribati	0.410389821	0.412884199	0.415577609	0.418481661	0.421716217	0.425024354
Kuwait	0.664517904	0.668809621	0.67015911	0.670036252	0.671393213	0.677215523
Kyrgyzstan	0.519407652	0.523746714	0.527901552	0.532431008	0.53623298	0.53905282

Lao People's Democratic R	0.264283164	0.268787618	0.273435312	0.278140418	0.28327023	0.288795341
Latvia	0.680193638	0.688117297	0.696157036	0.705249855	0.712531353	0.717639459
Lebanon	0.536718969	0.539197492	0.541793516	0.544581306	0.547684033	0.55147172
Lesotho	0.339155125	0.345496571	0.35216963	0.358780457	0.365310801	0.371265315
Liberia	0.235296847	0.238212218	0.238283313	0.235984406	0.232656774	0.228424478
Libya	0.527998168	0.541432399	0.554468227	0.566798415	0.578651416	0.589578634
Lithuania	0.668503938	0.673234121	0.68099288	0.687480621	0.691151653	0.69473438
Luxembourg	0.781051609	0.785961442	0.789053364	0.792843836	0.797824008	0.80268247
Madagascar	0.279889465	0.280429589	0.280501283	0.280204299	0.279615149	0.279164003
Malawi	0.204010245	0.206043786	0.206732359	0.208091205	0.209144566	0.211756232
Malaysia	0.545799405	0.551683548	0.558186752	0.565845656	0.574380446	0.583441103
Maldives	0.331601544	0.344398607	0.35825234	0.372806999	0.388081604	0.403945583
Mali	0.126526428	0.12912815	0.131537449	0.134177054	0.136937829	0.139866225
Malta	0.656504584	0.661112691	0.666324648	0.672120257	0.677847314	0.682617704
Marshall Islands	0.430839288	0.435571412	0.440491018	0.445479084	0.450848221	0.456859674
Mauritania	0.335780942	0.341135175	0.346531288	0.352176572	0.357585912	0.363037695
Mauritius	0.544586533	0.548209121	0.55345463	0.56068864	0.569092171	0.57731939
Mexico	0.504996083	0.50951067	0.514638057	0.520493167	0.526749241	0.531554141
Micronesia (Federated Stat	0.462511617	0.468144831	0.473792936	0.479939595	0.485300006	0.491015185
Monaco	0.845495153	0.84830369	0.85086394	0.853264992	0.855626927	0.857986147
Mongolia	0.466550118	0.472199014	0.477586554	0.482726727	0.488276683	0.495006781
Montenegro	0.67422572	0.674305723	0.672664772	0.668665719	0.665011931	0.662765437
Morocco	0.35807287	0.364984629	0.371187194	0.376778977	0.382849451	0.38816411
Mozambique	0.173064715	0.175550435	0.177285188	0.179851807	0.182236861	0.183427982
Myanmar	0.319219724	0.322034719	0.325628884	0.329707434	0.33429012	0.339590976
Namibia	0.450040233	0.45453444	0.460021314	0.465734135	0.471873217	0.477794329
Nauru	0.539145981	0.538433557	0.537038893	0.535059742	0.533001097	0.530890925
Nepal	0.199560654	0.205687443	0.21211309	0.218843431	0.22607306	0.233253154
Netherlands	0.794612123	0.799285437	0.803965978	0.808278967	0.812530216	0.816662769
New Zealand	0.752321655	0.755616091	0.759461372	0.763634604	0.767118658	0.769998036
Nicaragua	0.346035235	0.351740622	0.357712704	0.363562069	0.369727968	0.376625345
Niger	0.08086848	0.082848208	0.084771578	0.08669649	0.088608733	0.090473708
Nigeria	0.305868047	0.308489225	0.311234052	0.31398306	0.316747447	0.319637439
Niue	0.587532984	0.593882057	0.599881243	0.605514779	0.610650175	0.615374609
North Macedonia	0.609026094	0.611337453	0.612708856	0.613195099	0.614362905	0.616994878
Northern Mariana Islands	0.708593838	0.712223642	0.715787607	0.719179396	0.722510494	0.726219053
Norway	0.795887277	0.800468886	0.805437473	0.810565689	0.815414668	0.820223522
Oman	0.429270949	0.442268122	0.457737972	0.474999033	0.493513107	0.513194359
Pakistan	0.310467621	0.316265667	0.322248051	0.328219252	0.334433128	0.340926296
Palau	0.66290951	0.668230731	0.673302462	0.676648715	0.680195775	0.68483214
Palestine	0.40179221	0.40572715	0.411146869	0.416779649	0.423136673	0.429307027
Panama	0.546048123	0.550484218	0.554972755	0.559168666	0.561714534	0.563353023
Papua New Guinea	0.310668629	0.314967305	0.319530259	0.325005984	0.330664468	0.335421285
Paraguay	0.469527785	0.474352286	0.479314169	0.4846142	0.490226935	0.496336876
Peru	0.510419852	0.512161525	0.513821552	0.515981506	0.51930608	0.523640527
Philippines	0.510011796	0.513707183	0.517109502	0.520600378	0.524613607	0.528827907
Poland	0.627227888	0.632671471	0.640661793	0.6489505	0.658010514	0.666678867
Portugal	0.599777757	0.607269449	0.61480808	0.622101177	0.629306387	0.635498761
Puerto Rico	0.658758146	0.66326172	0.667272192	0.671024105	0.67545081	0.68067395
Qatar	0.651208376	0.655338767	0.660673763	0.666194399	0.672025384	0.678320339

Republic of Korea	0.692329307	0.702631128	0.712440842	0.72228674	0.732197906	0.74194003
Republic of Moldova	0.604251762	0.607270774	0.609664558	0.61415292	0.617603767	0.620951321
Romania	0.619298862	0.626795627	0.630475082	0.633801422	0.638371932	0.643718879
Russian Federation	0.671600578	0.680099211	0.688068022	0.692036194	0.694144966	0.697854214
Rwanda	0.27509719	0.276194698	0.277178171	0.276980133	0.272331258	0.271102227
Saint Kitts and Nevis	0.580685877	0.59025495	0.59918719	0.607235892	0.614514658	0.620716498
Saint Lucia	0.49629657	0.505975555	0.515368261	0.524234949	0.532731083	0.541479794
Saint Vincent and the Gre	0.475930186	0.481075658	0.485977871	0.490998791	0.496379132	0.50298609
Samoa	0.487491428	0.490987146	0.494027448	0.497079264	0.499484836	0.502369735
San Marino	0.813244888	0.818282513	0.822782758	0.827382324	0.831970983	0.836762726
Sao Tome and Principe	0.309542852	0.310601028	0.311515447	0.312743558	0.314261087	0.31584392
Saudi Arabia	0.538954515	0.549907717	0.561144266	0.572186913	0.582878871	0.593251423
Senegal	0.238047613	0.244482178	0.250416532	0.255767964	0.260379997	0.264438773
Serbia	0.63051102	0.638016934	0.641971994	0.642096034	0.642223221	0.642739671
Seychelles	0.575526496	0.582698331	0.590052919	0.598044194	0.606041268	0.613853281
Sierra Leone	0.211569335	0.213013567	0.21422304	0.216089201	0.218277411	0.219822604
Singapore	0.686404444	0.695055558	0.703427834	0.712315204	0.721256797	0.729982231
Slovakia	0.653853505	0.658731402	0.665830111	0.675967937	0.688136477	0.697476536
Slovenia	0.727463928	0.732666986	0.737391552	0.741774172	0.746234861	0.750994975
Solomon Islands	0.301217167	0.305830668	0.311431697	0.317151092	0.323300247	0.329968564
Somalia	0.048848564	0.049422382	0.050115531	0.050797999	0.051471696	0.052148606
South Africa	0.541571435	0.546921136	0.552066899	0.557198439	0.562448636	0.567905304
South Sudan	0.2066565	0.208932716	0.211199401	0.213416471	0.215612089	0.217920463
Spain	0.636673166	0.644130745	0.651597597	0.658637569	0.665321549	0.671576034
Sri Lanka	0.522622553	0.528239203	0.533345535	0.538891102	0.545124551	0.551744058
Sudan	0.292178643	0.296473446	0.300863677	0.305409326	0.31009643	0.315208259
Suriname	0.502054305	0.506655795	0.510959772	0.514419437	0.517650245	0.521002622
Sweden	0.785535792	0.790096441	0.795431458	0.801009522	0.806700264	0.812244654
Switzerland	0.862766844	0.864796867	0.867736079	0.870863558	0.873140871	0.874835331
Syrian Arab Republic	0.430492643	0.437907493	0.445904236	0.45414786	0.462437298	0.470692043
Taiwan (Province of China	0.667633854	0.676537237	0.684803636	0.69292413	0.70107875	0.709626821
Tajikistan	0.466155413	0.472342173	0.475745195	0.478238813	0.478726011	0.477586868
Thailand	0.506644861	0.515911466	0.525161869	0.534303709	0.543325547	0.552452209
Timor-Leste	0.262468083	0.270943349	0.27991676	0.289266629	0.298763189	0.308018684
Togo	0.269692273	0.274530568	0.27878931	0.281224631	0.284653387	0.288536692
Tokelau	0.521942386	0.526628473	0.53130785	0.535935005	0.540523665	0.545295634
Tonga	0.49180684	0.498166205	0.503659848	0.50890445	0.514207835	0.52021502
Trinidad and Tobago	0.62397015	0.628758918	0.634039552	0.639191914	0.64424291	0.649220055
Tunisia	0.471138521	0.479543057	0.488285281	0.496852039	0.505564882	0.51419694
Turkmenistan	0.563126887	0.564939386	0.565587505	0.567174449	0.567288976	0.567198635
Tuvalu	0.406247566	0.414819937	0.42290198	0.43051826	0.438563051	0.444962242
Türkiye	0.461606984	0.469244066	0.476814569	0.48465697	0.49161995	0.49882484
Uganda	0.187001096	0.188924156	0.191003433	0.193557456	0.197293193	0.201835423
Ukraine	0.647461466	0.651950021	0.657600333	0.662867405	0.665506375	0.666950167
United Arab Emirates	0.644412271	0.660739899	0.675926211	0.689463801	0.701656792	0.713138288
United Kingdom	0.744334126	0.749148231	0.754556689	0.76015435	0.7651646	0.768656864
United Republic of Tanzar	0.259306074	0.262416238	0.264989448	0.267254915	0.269417204	0.272078202
United States Virgin Islands	0.655160856	0.664018659	0.671081871	0.676872745	0.681982223	0.686730211
United States of America	0.76364769	0.766044295	0.769595492	0.773103517	0.776368588	0.779410254
Uruguay	0.581921855	0.586156118	0.589822355	0.592295025	0.595188424	0.596825409

Uzbekistan	0.500241735	0.501544609	0.502905194	0.506556615	0.511018525	0.516986264
Vanuatu	0.353100252	0.357005379	0.360893296	0.364444155	0.368501002	0.372479126
Venezuela (Bolivarian Rep	0.516891218	0.522272296	0.529517991	0.536279978	0.541866058	0.546348234
Viet Nam	0.407630048	0.41321725	0.419781657	0.427072185	0.435106272	0.443858785
Yemen	0.215664586	0.222885409	0.230415046	0.238025246	0.245879426	0.253962572
Zambia	0.304008549	0.30584236	0.307316277	0.308780208	0.309223449	0.309995813
Zimbabwe	0.398559341	0.406052877	0.412083826	0.4179742	0.424051564	0.42892904

1996	1997	1998	1999	2000	2001	2002
0.178085404	0.177884124	0.177513403	0.177075239	0.177025772	0.177773144	0.18380843
0.562248886	0.566802804	0.572534102	0.578295462	0.584664188	0.592412947	0.599865978
0.505990967	0.514056566	0.522382419	0.530785583	0.539481508	0.547529277	0.555172222
0.632706983	0.634926685	0.636597976	0.638322193	0.64060955	0.643214421	0.646002062
0.772403471	0.774580971	0.776972578	0.779629634	0.781976506	0.785417035	0.790158759
0.291421279	0.295433993	0.299553353	0.303720042	0.307603312	0.311697534	0.316706571
0.641647192	0.645895591	0.650614287	0.655534994	0.660819064	0.665620632	0.670360801
0.618942624	0.623934409	0.628723436	0.633175129	0.638194472	0.642175705	0.644472507
0.560537778	0.564627462	0.569556663	0.574323064	0.579437569	0.585419295	0.592710614
0.751991736	0.756633901	0.761133265	0.765697506	0.770223843	0.774662787	0.77936094
0.774446565	0.779232856	0.783521016	0.787451584	0.79184768	0.795770118	0.79915412
0.586315946	0.583221813	0.580392893	0.579192506	0.580020573	0.582193708	0.585716143
0.728478844	0.734781215	0.739281293	0.741622848	0.742517946	0.743592882	0.746250322
0.619485376	0.624596062	0.630738301	0.638083187	0.646750697	0.657196064	0.664901201
0.271177781	0.27777328	0.28451594	0.290951431	0.297251976	0.303188546	0.308640093
0.677193054	0.678644826	0.679827167	0.680398459	0.681304199	0.683329389	0.687807576
0.652855221	0.655627296	0.657246467	0.66017101	0.664705822	0.671333269	0.678121782
0.766826075	0.770813953	0.774228268	0.77704104	0.780396331	0.784713043	0.788836054
0.482702165	0.488020129	0.491819335	0.495070196	0.49864373	0.502399434	0.506871655
0.241895323	0.245731534	0.249376451	0.253215341	0.257380681	0.261388011	0.265417458
0.717831671	0.721642813	0.725853528	0.730539823	0.735996502	0.741918908	0.747544503
0.262362746	0.271487422	0.280440384	0.289510488	0.298804875	0.308200652	0.317943536
0.460220669	0.466982897	0.473796201	0.48019431	0.486327903	0.492030764	0.497348837
0.545549215	0.563565415	0.581829515	0.597997469	0.610950685	0.621989464	0.632075518
0.478588352	0.487767857	0.496193622	0.504978826	0.513498223	0.521370182	0.529252964
0.523863329	0.528177889	0.532481169	0.536972035	0.542051497	0.54730443	0.552776035
0.697602233	0.702944386	0.708630119	0.714851237	0.721552493	0.728535069	0.735343916
0.678862584	0.680221466	0.678496877	0.677446543	0.681004524	0.686487366	0.691511674
0.150041686	0.154228401	0.158707048	0.163468067	0.16808434	0.172996395	0.17798199
0.215526325	0.21576182	0.216402603	0.216855767	0.216869858	0.216988726	0.217782477
0.319736229	0.329049585	0.338786069	0.349296918	0.359899017	0.370070276	0.379846674
0.31563097	0.32050375	0.324974299	0.330224246	0.33607503	0.342507911	0.349465712
0.333425982	0.337346001	0.34119709	0.344955364	0.348814789	0.352631873	0.356511817
0.80106497	0.804833356	0.808135441	0.81226594	0.816685059	0.82069625	0.824402272
0.233989591	0.236671999	0.239743529	0.24298853	0.246021132	0.24924674	0.252524369
0.130169484	0.132136021	0.134090304	0.135825261	0.137412374	0.139663692	0.1420417
0.62369446	0.630540085	0.638082758	0.645928084	0.652989028	0.66026551	0.667888408
0.512033996	0.522326866	0.531379484	0.540333688	0.547989193	0.553680374	0.560542936
0.504986969	0.511079502	0.517022505	0.52243594	0.527839094	0.533076501	0.538189342
0.322249069	0.330148125	0.337517914	0.344774694	0.351875506	0.359211999	0.366486127
0.447566642	0.451026961	0.454445045	0.45717654	0.460764814	0.463751682	0.467037268
0.606414151	0.613991564	0.621202228	0.62802903	0.635365607	0.644421442	0.652790488
0.565180862	0.570128417	0.576067051	0.582229492	0.588243191	0.594889115	0.600894433
0.679507318	0.68536501	0.691365519	0.696636411	0.703039899	0.709536841	0.715383422
0.559346329	0.558924568	0.55936322	0.561820953	0.566676902	0.572753861	0.57996829
0.709839946	0.718864244	0.727964382	0.736846135	0.745199301	0.753594717	0.761502465
0.74604138	0.751680344	0.757033755	0.76242888	0.767766159	0.77216724	0.776927934

0.314965385	0.319843979	0.324712873	0.329201009	0.333015393	0.336545511	0.339977638
0.498429014	0.497136869	0.49615921	0.496484754	0.497461995	0.499674043	0.502633569
0.28041316	0.276336503	0.271813823	0.267367662	0.262456747	0.257857505	0.25413636
0.823375243	0.827679979	0.831683237	0.835381543	0.839184298	0.84317775	0.847086357
0.354385634	0.358096401	0.361596764	0.365305211	0.369015095	0.372853155	0.376650004
0.61438205	0.625057353	0.633017994	0.639453915	0.645830937	0.652368567	0.658423554
0.468516938	0.474504389	0.481170939	0.488178541	0.495468586	0.502775415	0.510298019
0.532691399	0.535349652	0.53719633	0.539032308	0.543044779	0.54855215	0.554897325
0.476116627	0.482970886	0.489184071	0.495502619	0.502169886	0.508716824	0.515038129
0.400178468	0.410455826	0.42142119	0.432733148	0.443962666	0.454177659	0.46296886
0.324601221	0.350550963	0.371237163	0.391128023	0.41138345	0.433590057	0.451939
0.272527055	0.283551721	0.293764828	0.30314234	0.310248162	0.317247988	0.323566585
0.718813024	0.72465244	0.729027035	0.733250616	0.739713154	0.746602207	0.752629898
0.447399238	0.454089854	0.460136984	0.465990845	0.471852843	0.477233374	0.482474423
0.158127229	0.161874743	0.16507771	0.168903545	0.17341853	0.178972969	0.184138013
0.565041879	0.571187753	0.577172354	0.583573942	0.589196631	0.594497419	0.599322031
0.774809059	0.778814215	0.782305059	0.785773988	0.78998523	0.794453835	0.798770274
0.760899579	0.764650672	0.767852561	0.770304363	0.772792033	0.776225157	0.780088872
0.48946146	0.495074162	0.50037255	0.50480635	0.509133087	0.513045709	0.516917621
0.27295019	0.278274122	0.283946056	0.290070621	0.296354282	0.302543257	0.307712927
0.64455809	0.638525631	0.633347931	0.630277946	0.630265991	0.6333883	0.638370108
0.839188129	0.841385903	0.843854978	0.846232398	0.848925756	0.852070413	0.855066793
0.405003047	0.410038073	0.414888532	0.419755496	0.424725212	0.429411846	0.434104128
0.710139336	0.715687268	0.721593982	0.727529455	0.73271054	0.737154008	0.741945221
0.730216225	0.730551087	0.733242588	0.73655713	0.74085701	0.746289605	0.752878371
0.496589808	0.506217602	0.516590019	0.527333411	0.538388116	0.54905897	0.559473128
0.684173735	0.691684871	0.701819289	0.713641593	0.724802357	0.734267376	0.741284245
0.346961212	0.355995963	0.366461193	0.374455316	0.379877297	0.386927686	0.396899639
0.198633755	0.203280449	0.208003383	0.212412705	0.216649792	0.221090941	0.225898779
0.236106374	0.241360584	0.244371797	0.248217317	0.252026519	0.255786768	0.259321816
0.495216036	0.504362779	0.513068898	0.521595978	0.528693992	0.534869806	0.540514273
0.338229396	0.343880049	0.349698073	0.355980743	0.362572209	0.368643508	0.374473325
0.364328424	0.369983894	0.375950111	0.381752316	0.387988053	0.394174012	0.400371798
0.690883334	0.697935514	0.70420272	0.709980627	0.715805281	0.722099055	0.728384596
0.78535642	0.788528335	0.792626368	0.797368808	0.80303033	0.808965786	0.813400392
0.370018644	0.376358763	0.382817848	0.389453889	0.395752331	0.401837472	0.407411414
0.510108146	0.518102189	0.524045604	0.52917633	0.533789587	0.537977981	0.542245676
0.52860072	0.537727779	0.546366017	0.555624332	0.565474561	0.574618208	0.584097268
0.436818479	0.443104027	0.452384092	0.462736441	0.472376399	0.48145832	0.488664737
0.754010124	0.759970384	0.766488721	0.773538834	0.780190655	0.786353741	0.793010606
0.734714026	0.738015277	0.74192719	0.745443338	0.749184084	0.752834297	0.755988076
0.73605087	0.739972336	0.743101596	0.746074084	0.749675986	0.753554839	0.757191479
0.570940131	0.577212091	0.583048035	0.588846467	0.594592917	0.600192022	0.605591748
0.813870746	0.816675782	0.819016202	0.821066637	0.822938999	0.824723241	0.826936774
0.567529655	0.572581508	0.57764357	0.582759199	0.587909394	0.593182803	0.599000385
0.626013138	0.631166626	0.635388624	0.640180485	0.644689623	0.648397258	0.651529335
0.366203076	0.370436465	0.374581401	0.378630009	0.382395616	0.386188415	0.389886256
0.428300682	0.431641941	0.435741904	0.439692923	0.444436339	0.448782301	0.453462298
0.686093304	0.695789389	0.704383736	0.711841328	0.718357562	0.722840631	0.726816976
0.541628927	0.54334115	0.543258546	0.542867282	0.543036487	0.543805551	0.54437467

0.294838372	0.301477967	0.308402545	0.315972615	0.323947359	0.332580409	0.341667271
0.721139538	0.72374658	0.725427336	0.727219685	0.730951449	0.736294196	0.74187044
0.556436159	0.562120818	0.56905907	0.576078528	0.582698277	0.589185502	0.595978788
0.377221202	0.383096648	0.388406478	0.393495001	0.398635802	0.403990126	0.409021657
0.222682591	0.219672765	0.219053855	0.222212867	0.231173362	0.239594386	0.248311971
0.600350044	0.610620315	0.619973984	0.628600604	0.637047935	0.644685131	0.651669147
0.698591301	0.70284095	0.707468182	0.712834171	0.71989696	0.727133585	0.733793398
0.806516138	0.810235288	0.813889026	0.817570995	0.822006568	0.825312816	0.828223491
0.279066493	0.27942619	0.280317671	0.281571072	0.283190721	0.28566818	0.287153149
0.215264475	0.219416252	0.22312233	0.227068368	0.23090081	0.234020507	0.237602155
0.593152135	0.603418105	0.612645412	0.621724901	0.630523544	0.638257477	0.645044248
0.420519128	0.437436026	0.453973361	0.469338066	0.483495671	0.495923695	0.50740486
0.143078916	0.146414326	0.14998673	0.153737336	0.157190028	0.161344448	0.165420696
0.686623261	0.691756406	0.698095287	0.704734545	0.711805254	0.717803038	0.722790275
0.46120943	0.464752728	0.468133032	0.471292727	0.474562536	0.478588316	0.482776259
0.368508328	0.373021339	0.37708025	0.381112297	0.38420538	0.386487416	0.388564478
0.584797061	0.591497735	0.597363039	0.602034234	0.607087901	0.612240817	0.617190007
0.536530827	0.541988171	0.547842121	0.55381337	0.559532547	0.564774176	0.570113531
0.495570308	0.499110106	0.502861564	0.506663431	0.511004228	0.515316814	0.519545672
0.860245432	0.86244672	0.864716513	0.866842394	0.868973583	0.871094755	0.873182769
0.501703718	0.508558268	0.514793383	0.520781189	0.526650345	0.532620201	0.538424835
0.663414769	0.665542032	0.669024771	0.672096103	0.67730685	0.683433454	0.689939483
0.394150096	0.399504009	0.405038792	0.410303191	0.415344026	0.420595133	0.42577575
0.185705869	0.188650436	0.191998629	0.195435288	0.198534397	0.202241924	0.20639006
0.345191641	0.350892883	0.356696902	0.363037732	0.370360017	0.37817198	0.386343628
0.483116378	0.488012938	0.492812548	0.497613862	0.502406958	0.506719033	0.510818275
0.528544474	0.52624159	0.523885302	0.521894324	0.520254888	0.519235425	0.518438705
0.240700616	0.248382797	0.256227179	0.26435918	0.272719115	0.281287467	0.289513608
0.81998422	0.82288177	0.825882234	0.828693827	0.831913984	0.835518148	0.838676077
0.773575283	0.778205776	0.781730757	0.784428638	0.787659557	0.791426079	0.794519591
0.384087266	0.391775893	0.399807535	0.408085965	0.416108363	0.423790295	0.430813755
0.092346359	0.094219115	0.096545153	0.098813686	0.100886948	0.103271777	0.105741109
0.323137685	0.326690553	0.330263851	0.3339365	0.338201743	0.343029751	0.349092528
0.619734555	0.623913848	0.627578934	0.630857172	0.63406895	0.637366627	0.641122021
0.620944017	0.626223811	0.63205941	0.636687259	0.641651243	0.648049676	0.65539879
0.730074086	0.733795366	0.737423127	0.740934815	0.744492865	0.747170606	0.748755189
0.825701668	0.83146877	0.836562809	0.841130237	0.846475451	0.852111936	0.856757888
0.533246394	0.554315537	0.574474902	0.59239847	0.608421541	0.622160627	0.635162126
0.347656203	0.354218627	0.360875336	0.367641397	0.374319132	0.380593927	0.386629992
0.690095673	0.695122123	0.69965291	0.703115322	0.705196945	0.707402386	0.709587644
0.434731371	0.440901776	0.447782415	0.454909692	0.460681496	0.465299102	0.468600257
0.56531007	0.569395939	0.574697318	0.580432842	0.587378393	0.594535701	0.600138677
0.340348698	0.344258325	0.347801408	0.351250621	0.354135791	0.356588986	0.358572086
0.502322804	0.508286948	0.513819896	0.518720583	0.523007793	0.526916763	0.531177358
0.528312042	0.533576491	0.538721717	0.543853377	0.548952201	0.553775453	0.558853263
0.532938849	0.536589868	0.53949647	0.542257968	0.544997723	0.547409432	0.549521153
0.674378829	0.682587612	0.690805671	0.698536056	0.706662052	0.714831304	0.722009458
0.640864526	0.646192904	0.651335994	0.656120667	0.661610852	0.667247151	0.67258394
0.685865893	0.691274382	0.697040445	0.702850671	0.710035652	0.718955861	0.725956856
0.684835919	0.693109654	0.701409064	0.708880981	0.714831609	0.720831064	0.727112174

0.75155324	0.760591683	0.768281885	0.776773436	0.785428821	0.793496281	0.801024295
0.623113984	0.624370105	0.624116761	0.622834529	0.622004934	0.62293641	0.625874845
0.648104195	0.651225576	0.654927769	0.659544846	0.665294346	0.672020167	0.678297417
0.701468231	0.703752867	0.705198518	0.706863538	0.708643648	0.710570359	0.713460957
0.271097922	0.272455861	0.274582551	0.277014387	0.280983454	0.286082055	0.29295543
0.626207411	0.63127474	0.635539988	0.639713739	0.644353377	0.649226653	0.654593409
0.550242412	0.558293461	0.566138703	0.573428555	0.579788896	0.585087558	0.590167578
0.509527158	0.515482619	0.521209679	0.527099552	0.53317329	0.539455712	0.546063616
0.505778026	0.509096382	0.512367566	0.51540905	0.518902327	0.522971626	0.52724267
0.841389495	0.845864865	0.850474771	0.855338613	0.859695363	0.863747757	0.865680592
0.317729288	0.31979567	0.322519219	0.325651916	0.329460109	0.334484786	0.340302837
0.603648418	0.613749116	0.623518214	0.632796447	0.642311774	0.651502092	0.660356856
0.268318965	0.272072875	0.275995656	0.280111918	0.283784903	0.287451899	0.291046288
0.644846417	0.647162094	0.650245989	0.655534174	0.6607528	0.665407335	0.671511072
0.62213517	0.630697732	0.638438882	0.645339666	0.651551988	0.656490668	0.66090779
0.21996927	0.219089791	0.218584313	0.217943042	0.218492837	0.220391233	0.224585825
0.739028235	0.747571898	0.754873204	0.760831136	0.76771737	0.774294657	0.77973577
0.704405003	0.71151321	0.719058766	0.726144173	0.73404624	0.741392554	0.746791142
0.756348987	0.762275292	0.767728526	0.773236192	0.77957801	0.786240776	0.791805569
0.336208053	0.341835627	0.34713506	0.351867191	0.354799143	0.356626745	0.357910116
0.052840548	0.053570967	0.054321527	0.055071838	0.055842063	0.056643165	0.057457612
0.573453988	0.579099278	0.584437313	0.589407349	0.594093683	0.597884168	0.600921676
0.220399437	0.223037171	0.225788869	0.228648585	0.231730344	0.235014128	0.238341186
0.677088943	0.682429497	0.687490717	0.692245197	0.697056031	0.70172607	0.706309655
0.558938888	0.566128422	0.572326187	0.577630048	0.582942638	0.587556599	0.592359127
0.320680229	0.326761462	0.333087761	0.339754884	0.346875809	0.354215515	0.361728237
0.525059318	0.529750812	0.534599696	0.538932791	0.543660023	0.548683524	0.553879432
0.817227025	0.821694059	0.825731995	0.829487099	0.833028112	0.835956398	0.838741527
0.876640557	0.878485545	0.880035152	0.882045586	0.885243531	0.888445951	0.890902811
0.478743085	0.48623235	0.493707992	0.500474869	0.507143686	0.513850111	0.521309744
0.719128415	0.729145497	0.738125787	0.745338399	0.752926841	0.760829809	0.769008984
0.474346027	0.470274796	0.465341742	0.460505616	0.457061333	0.456256513	0.45880778
0.561302487	0.568449492	0.573817902	0.578854168	0.583500917	0.587661561	0.591759098
0.31758875	0.326705374	0.334894497	0.33925214	0.343793649	0.34949032	0.35416202
0.292606677	0.296947835	0.300022333	0.303020232	0.305472963	0.307828258	0.31046991
0.550282652	0.555631326	0.561047611	0.566415406	0.57181437	0.576915597	0.581858317
0.525808135	0.531009092	0.536172314	0.54131702	0.546303195	0.551054251	0.556008345
0.654169455	0.659293633	0.664507714	0.670244574	0.676683835	0.682763494	0.688872271
0.523294274	0.532064044	0.540498161	0.548724563	0.55667265	0.56442101	0.571719534
0.567061261	0.565623084	0.564405264	0.564716006	0.567421033	0.571043819	0.575596166
0.44980498	0.455368428	0.462454678	0.468845802	0.474956823	0.480850886	0.487252252
0.506238546	0.513933356	0.521509898	0.528512416	0.535866331	0.54235913	0.549023644
0.207080731	0.213091696	0.219712442	0.226916945	0.234323225	0.242186388	0.250552388
0.668104744	0.669207846	0.670322301	0.670642905	0.671095001	0.67246782	0.674543221
0.724181976	0.734723175	0.744476623	0.753253916	0.760969094	0.767858793	0.774346424
0.771576484	0.775050122	0.779057165	0.784036475	0.78921896	0.793513165	0.79665556
0.275507909	0.279259184	0.28315261	0.287381679	0.291827265	0.296715645	0.301983995
0.691247699	0.695598204	0.699768686	0.703949342	0.708436565	0.713751115	0.724398272
0.78193477	0.783979278	0.786076332	0.788544535	0.79231665	0.796676793	0.800403318
0.599775104	0.605665051	0.612700815	0.619009271	0.623208291	0.625796068	0.6282858

0.52439291	0.532686328	0.541279069	0.549739391	0.557522311	0.564803383	0.571690143
0.376460885	0.380667171	0.384690196	0.388300526	0.392158349	0.395325055	0.397723782
0.548649226	0.552364437	0.557243117	0.560277999	0.56350626	0.565458381	0.562604461
0.45310241	0.462853518	0.472478892	0.481445774	0.489802045	0.497630349	0.505453447
0.262278613	0.270782536	0.279500507	0.288261301	0.297145157	0.306112424	0.315065023
0.311326691	0.31308733	0.314926435	0.317396437	0.320625322	0.324401368	0.328680122
0.434187192	0.438141988	0.441093135	0.443246604	0.444841402	0.446354681	0.446970895

2003	2004	2005	2006	2007	2008	2009
0.190518991	0.196903541	0.203928133	0.211074194	0.219627611	0.228089171	0.237900824
0.606971166	0.613864247	0.620679615	0.627485617	0.634167969	0.640766134	0.64681613
0.562536956	0.569352852	0.576001965	0.582126362	0.587790538	0.593190973	0.597870384
0.648951964	0.651921141	0.655061005	0.657725723	0.660781176	0.664025762	0.667300532
0.797278901	0.80473198	0.81221398	0.81978726	0.826617254	0.831821715	0.835929887
0.321777362	0.327395266	0.333785188	0.341012622	0.348846841	0.357082005	0.364407744
0.67531149	0.680412429	0.685486437	0.690826021	0.696360039	0.702175362	0.707128564
0.645906577	0.648296828	0.65336883	0.6574459	0.660187576	0.663079235	0.665911944
0.60080917	0.609031766	0.617759634	0.626688335	0.635870982	0.644431591	0.649714845
0.783600181	0.787016543	0.789615521	0.791155688	0.792532683	0.7952929	0.799174373
0.802043253	0.804889144	0.808419194	0.812064609	0.815480522	0.818793658	0.821541167
0.590038705	0.594646085	0.602485193	0.613771097	0.626110926	0.636901109	0.645163341
0.749916714	0.753759643	0.756782661	0.759185939	0.761990997	0.765590503	0.769561674
0.669564111	0.674147775	0.679790598	0.686144289	0.693045147	0.699806387	0.70488584
0.31436004	0.320922851	0.32809051	0.335690598	0.343932546	0.352520033	0.361481513
0.693235962	0.698029703	0.701510049	0.703856186	0.705871649	0.708514495	0.712143362
0.684868556	0.692502135	0.698985483	0.705119103	0.711496562	0.719120871	0.727292784
0.792014384	0.795224936	0.798497236	0.801572763	0.804612664	0.807757945	0.811055825
0.512679459	0.519697333	0.527231062	0.534889621	0.541900694	0.548093223	0.553465869
0.269544281	0.273941145	0.27840675	0.282933936	0.287673055	0.292627446	0.297702227
0.753305163	0.759008885	0.764690903	0.77059677	0.776114264	0.780358325	0.784759107
0.327670422	0.337286495	0.346998578	0.356824209	0.367560498	0.378233543	0.388977162
0.502365803	0.507273036	0.512166491	0.517146194	0.52194613	0.52694375	0.53179244
0.640635102	0.647793732	0.653859137	0.659813662	0.665578735	0.671100814	0.6760329
0.537000179	0.544349223	0.55170697	0.55922162	0.566702496	0.573883805	0.579696412
0.55814571	0.563772907	0.569458118	0.575338775	0.581588755	0.588077818	0.594073297
0.741578091	0.747311715	0.752481793	0.757318655	0.761643129	0.76571426	0.769601682
0.695565965	0.699881951	0.704471207	0.708868294	0.713192402	0.71767245	0.723676627
0.183253539	0.188564995	0.194086128	0.199571952	0.204891092	0.210223295	0.215423374
0.218961537	0.220918709	0.223084372	0.22582189	0.228996819	0.232853551	0.23694304
0.389419786	0.398838732	0.408009221	0.417433786	0.427379787	0.43737213	0.446691506
0.35692966	0.364854645	0.373331815	0.381698166	0.38987623	0.39767952	0.404220933
0.360668581	0.36510003	0.369545756	0.374355878	0.379490349	0.384838304	0.390288643
0.827801096	0.83092811	0.833288294	0.83491566	0.836625096	0.838913685	0.841583671
0.255101519	0.257778778	0.260451918	0.263712446	0.267332195	0.271158118	0.275338515
0.145348052	0.150910868	0.157033036	0.162576402	0.168161062	0.173396784	0.178335361
0.673445927	0.678258212	0.682589191	0.685892748	0.688930323	0.692359787	0.696602141
0.569018827	0.578178091	0.588052194	0.59875817	0.609520246	0.619454833	0.629307727
0.543265131	0.548601832	0.554089191	0.560182735	0.566822644	0.573494652	0.579876469
0.373410566	0.380035976	0.386891845	0.393521434	0.399051094	0.404649755	0.410253575
0.470134374	0.473612436	0.478137627	0.483355822	0.48764074	0.492822855	0.498299969
0.661465038	0.668769675	0.674004103	0.680644001	0.687505191	0.695592069	0.70377698
0.605765687	0.610315699	0.614412619	0.617850162	0.621200571	0.62570391	0.631291379
0.721130377	0.726327652	0.73215414	0.738066879	0.743148777	0.748469602	0.754065605
0.587647762	0.594348715	0.599863799	0.604970567	0.607618099	0.609400923	0.611775641
0.767896216	0.773764775	0.779031361	0.784560965	0.792068383	0.799454089	0.805112085
0.782448081	0.787126405	0.791333948	0.794922784	0.798254118	0.801764846	0.804962225

0.343348932	0.346375411	0.348727913	0.350715944	0.352727478	0.355087643	0.357938755
0.506165243	0.510229134	0.515097763	0.51974144	0.524066965	0.528813498	0.533114411
0.252333885	0.252470919	0.254161023	0.257355123	0.262272267	0.268103872	0.274361144
0.850441326	0.853304103	0.855690732	0.85803321	0.859781734	0.861782061	0.86394805
0.380686615	0.384987706	0.389568706	0.395037471	0.400618546	0.406658303	0.411969335
0.663678868	0.668952132	0.674549077	0.680342482	0.685415917	0.690197519	0.694461869
0.517395574	0.524543275	0.532228649	0.54022269	0.548187021	0.555371313	0.561783754
0.560955485	0.566290293	0.569927345	0.572516273	0.574704702	0.57812504	0.582484258
0.520623395	0.525181678	0.528665483	0.530395807	0.529457594	0.524743833	0.516255831
0.470397413	0.476536664	0.482041719	0.486826	0.490809752	0.495077739	0.499489714
0.467846833	0.483879612	0.498683715	0.511579828	0.524076461	0.53701899	0.548514683
0.328593581	0.33287938	0.336682511	0.339835824	0.342843174	0.343427801	0.345147743
0.757815678	0.763223727	0.769112182	0.774451842	0.781074443	0.788598874	0.794728213
0.487608829	0.492669159	0.498043849	0.503658294	0.509260219	0.514730896	0.520427398
0.188694814	0.195030701	0.202868243	0.211524229	0.221819931	0.232872186	0.244265818
0.603489622	0.60754489	0.610812944	0.613932767	0.616582915	0.618958809	0.620883465
0.802325431	0.805694227	0.808936787	0.812081482	0.815597613	0.818875544	0.821457588
0.783178757	0.786084655	0.789042086	0.79232192	0.795557651	0.79817743	0.80050867
0.521051412	0.525479071	0.530488575	0.534797716	0.539441424	0.544110892	0.548591319
0.312930916	0.318615029	0.32356835	0.328151431	0.332640147	0.337315245	0.342389614
0.644065579	0.648920978	0.653616005	0.658403372	0.663697966	0.668442496	0.672449381
0.85767579	0.860240333	0.862894178	0.865873707	0.86914044	0.872552849	0.875087911
0.439118213	0.444236977	0.44936002	0.454692737	0.460606245	0.467259067	0.473848047
0.747185956	0.752418607	0.756384396	0.760209696	0.763927173	0.767527658	0.771247356
0.759444152	0.765401567	0.77209958	0.779141406	0.785321922	0.791441257	0.79702575
0.569718037	0.578487456	0.587158877	0.59405452	0.600766336	0.606879282	0.611805257
0.74591153	0.74901652	0.750865875	0.751176245	0.752625471	0.755152408	0.758252296
0.408234164	0.419777065	0.430327529	0.438856376	0.447245965	0.456541865	0.464003077
0.230522594	0.23513497	0.239691845	0.244010127	0.24847169	0.253167689	0.257552785
0.262747724	0.266271736	0.270056132	0.273803987	0.277805017	0.282151502	0.286423345
0.545516473	0.55032637	0.554844797	0.559465611	0.564541135	0.569595206	0.574769931
0.380067255	0.385061512	0.38984542	0.394322001	0.398973441	0.403285871	0.407944424
0.406668381	0.413061816	0.419612332	0.426352997	0.433240315	0.439998164	0.446151054
0.734450658	0.740099975	0.745356806	0.750515381	0.754942942	0.759317468	0.763563687
0.816597067	0.819528087	0.822766536	0.826244743	0.83028989	0.834622678	0.838121013
0.413115783	0.419184961	0.425913062	0.433321558	0.44135146	0.449235851	0.457672332
0.546550264	0.550902314	0.555424913	0.560265924	0.565811896	0.572225267	0.578866902
0.593963012	0.60371745	0.613291056	0.621268095	0.627749442	0.63286223	0.63739838
0.492174445	0.498933881	0.504719431	0.510918049	0.517698972	0.525242908	0.533200914
0.800005865	0.80685314	0.811713818	0.814505479	0.816931026	0.819932128	0.823929666
0.758875409	0.762213291	0.765543461	0.768733898	0.770756143	0.772011615	0.773303913
0.760093744	0.762787749	0.765689914	0.768402408	0.77069838	0.77306847	0.775499262
0.610877424	0.615836278	0.620400057	0.62501886	0.62954947	0.633967851	0.638110835
0.829392223	0.832037381	0.834288685	0.836222264	0.83829416	0.840301217	0.842133664
0.60494595	0.611934404	0.619909228	0.627803813	0.635670276	0.643892693	0.652196319
0.654835926	0.658733071	0.662943942	0.667048858	0.671367208	0.676352869	0.681606839
0.39368048	0.397743651	0.402444011	0.407745764	0.413736227	0.419738147	0.426123888
0.458208374	0.462336213	0.466722276	0.470557197	0.47422745	0.477201933	0.480035601
0.731778685	0.738153707	0.746384913	0.754928493	0.762411484	0.770002615	0.777546551
0.545649262	0.547262343	0.548260449	0.549383594	0.551146192	0.553206382	0.555123866

0.350658178	0.359681759	0.368728678	0.378122281	0.387723649	0.397011299	0.406139022
0.748168162	0.755263211	0.761929433	0.768459929	0.776481959	0.786324573	0.794504427
0.60296724	0.61084576	0.619391355	0.627791217	0.636938234	0.646667663	0.657433186
0.413982571	0.418670919	0.423211228	0.427806732	0.432745038	0.438340567	0.443580127
0.251595458	0.25517961	0.25896799	0.262897258	0.267239568	0.271853596	0.276804111
0.659345125	0.666913926	0.675322268	0.683702	0.691855134	0.698873253	0.704998471
0.741229601	0.748949762	0.75635236	0.763059124	0.770229639	0.778273712	0.785689739
0.830983946	0.833672567	0.836675264	0.840574453	0.844707006	0.847978883	0.850688725
0.289675457	0.292629885	0.295919327	0.299723392	0.304026245	0.308990265	0.313450891
0.241559228	0.24643454	0.251672318	0.257595649	0.265226928	0.273689856	0.282296509
0.650885678	0.656243326	0.661242241	0.665956287	0.670843726	0.67639991	0.682070654
0.518926864	0.529910191	0.538253332	0.547950412	0.557529247	0.566839634	0.57444206
0.169733502	0.173779552	0.178029844	0.182452468	0.186912995	0.191536746	0.196537651
0.727511133	0.731933853	0.736188747	0.740140181	0.744237812	0.748427095	0.752171059
0.48624862	0.489573077	0.493271391	0.497456175	0.502250042	0.506359588	0.510794536
0.391104879	0.394012934	0.397499105	0.403159467	0.408377122	0.413233621	0.417910609
0.622764533	0.629030193	0.63476658	0.639545183	0.644358656	0.649630852	0.655000295
0.575698215	0.580984982	0.58522408	0.588543226	0.591447408	0.594454558	0.596684358
0.523829599	0.527610965	0.531703321	0.535718682	0.539431464	0.542766354	0.546275496
0.875197652	0.877231549	0.879288089	0.881274148	0.883262291	0.885331394	0.887328279
0.543971651	0.5495515	0.554680284	0.559561138	0.564236795	0.568500599	0.571341686
0.696517506	0.702998276	0.709065465	0.715459074	0.72250394	0.729981756	0.736538926
0.43121781	0.436822352	0.442531339	0.44867616	0.455036325	0.461803552	0.468929893
0.21053449	0.214972457	0.219692372	0.224758087	0.230158227	0.23579017	0.241360656
0.395218766	0.404692358	0.414662082	0.424705071	0.434536927	0.443595531	0.451954884
0.514574645	0.519103605	0.523881736	0.529350118	0.535062126	0.540896995	0.546516477
0.518459986	0.51880954	0.519892863	0.522673179	0.52320135	0.526084096	0.530221092
0.297772595	0.30619653	0.314661796	0.323056346	0.33144561	0.340150706	0.348701893
0.841639477	0.844807348	0.84793419	0.850798808	0.853595391	0.856368467	0.858600459
0.796286883	0.798177477	0.799107294	0.798456866	0.798447616	0.799669514	0.801727535
0.437248152	0.443274511	0.448844739	0.453950499	0.459042798	0.463837699	0.467961839
0.108322891	0.110764495	0.113452023	0.116247074	0.118971626	0.121957786	0.124829381
0.355911392	0.363780814	0.372583553	0.38102604	0.389779649	0.398037706	0.406353307
0.64577954	0.650614832	0.657281265	0.664162983	0.670199299	0.676396859	0.682579173
0.663408891	0.67123752	0.678047273	0.684044336	0.689729481	0.695376955	0.700453052
0.750171533	0.751528841	0.751826692	0.751797809	0.751608416	0.750807192	0.748597173
0.860302576	0.863468908	0.866217488	0.868588435	0.87092967	0.873745121	0.876582817
0.647249093	0.657626118	0.666401231	0.675353885	0.685055583	0.694952263	0.703098571
0.392719263	0.399084167	0.405667437	0.41223483	0.418519756	0.4243482	0.430019833
0.71100022	0.712543811	0.714339831	0.716353431	0.718809542	0.720806623	0.722288267
0.472881732	0.478527322	0.485320586	0.491890407	0.498978426	0.506849479	0.515815836
0.604043365	0.60687703	0.609494125	0.612249713	0.615285417	0.618958907	0.622402806
0.360736816	0.362827542	0.365294965	0.367826759	0.370794147	0.373426209	0.376528285
0.535360126	0.539556655	0.543622697	0.547943305	0.552758676	0.558016903	0.562900059
0.563871072	0.569063024	0.574517001	0.580503365	0.586958489	0.593750811	0.599954993
0.551567968	0.553704287	0.555664103	0.557976265	0.560822024	0.563974721	0.56735582
0.728155254	0.733673192	0.738428767	0.742363709	0.746008817	0.750521426	0.756427378
0.677663412	0.682352926	0.686887105	0.691158795	0.694974853	0.698604599	0.702223399
0.730475138	0.734219546	0.737742266	0.741629431	0.745883807	0.750922167	0.756361695
0.733536599	0.740223208	0.747189359	0.754123028	0.760960134	0.767659233	0.774276849

0.807687786	0.813720927	0.8190934	0.82417982	0.829127953	0.833894627	0.838108331
0.629985045	0.635394822	0.641530965	0.647996264	0.654215851	0.661046597	0.666284394
0.684074929	0.690417055	0.69499895	0.699490766	0.704523727	0.710757128	0.717445122
0.718875116	0.726305319	0.733592777	0.740064664	0.746191512	0.752623243	0.757960405
0.299676918	0.306943916	0.314219578	0.321998519	0.329616591	0.337039226	0.344494633
0.659925312	0.66756813	0.679145457	0.690078667	0.697727628	0.704141494	0.709499498
0.595675146	0.601625482	0.607239866	0.6127553	0.617994714	0.623273001	0.628169849
0.552693724	0.558663543	0.563588116	0.568221748	0.572819052	0.577611145	0.582549107
0.531603736	0.536003276	0.540557908	0.544812068	0.549110752	0.553164596	0.556474015
0.867522009	0.869287134	0.870979596	0.87265215	0.874307253	0.875991881	0.877705228
0.346851981	0.353722384	0.361105881	0.369413007	0.377813517	0.386688944	0.395821238
0.669839577	0.679917856	0.690520852	0.701152613	0.711442141	0.721960205	0.731158488
0.294887172	0.298984521	0.303257209	0.30763391	0.312570298	0.317894089	0.323657726
0.678334929	0.686819842	0.696097568	0.704945018	0.713242305	0.721505306	0.729965596
0.664162027	0.666461353	0.669264742	0.672149914	0.675077438	0.676590946	0.677847028
0.2292359	0.234131674	0.23954994	0.245647886	0.252523077	0.259781562	0.26705838
0.784165555	0.789852774	0.796455751	0.803342694	0.810625304	0.818124162	0.824251516
0.75116727	0.755836694	0.761724185	0.767673036	0.772914153	0.7771974	0.78105226
0.796371531	0.80096224	0.805601248	0.809708595	0.813006319	0.81634725	0.81887856
0.359424359	0.361290558	0.363580019	0.366443698	0.369827951	0.373764884	0.377821028
0.05827215	0.059076945	0.059898539	0.060741484	0.061598151	0.062475387	0.063382225
0.603515602	0.606262514	0.609989979	0.61501469	0.62060815	0.625948031	0.630645626
0.241797191	0.245462499	0.249280904	0.253300508	0.257491764	0.261740458	0.266179235
0.710678584	0.71488623	0.718717289	0.721970128	0.725211443	0.729411041	0.733956055
0.597410284	0.602250062	0.606906656	0.611807083	0.616979265	0.622414317	0.62814963
0.369693653	0.377920656	0.386685712	0.396151374	0.406079992	0.4160876	0.425975917
0.559418373	0.565109708	0.570462186	0.575647048	0.580654574	0.585644825	0.590512805
0.841663138	0.844506882	0.846876344	0.849115871	0.851420847	0.853705851	0.855702748
0.892780807	0.894909812	0.89727263	0.899962305	0.902913413	0.905778454	0.908049998
0.528935756	0.538084469	0.549750449	0.560484761	0.57002812	0.578990493	0.587789118
0.776955803	0.784677978	0.792445129	0.800174675	0.808137694	0.815400691	0.821432279
0.463329706	0.469154214	0.474637786	0.479651831	0.484211156	0.488441136	0.492064041
0.59604892	0.600520665	0.605040775	0.610356678	0.616368591	0.621067543	0.62432907
0.358440025	0.362687998	0.366913584	0.370654092	0.375390768	0.381227528	0.387944823
0.313792064	0.316687944	0.31902558	0.32165939	0.324382497	0.327743136	0.331558542
0.586854346	0.592342442	0.598176117	0.60442939	0.611163237	0.617940247	0.624070078
0.560736066	0.564806379	0.568449169	0.571619868	0.57435151	0.577305415	0.57917075
0.695828341	0.702610809	0.708520985	0.71460021	0.720196417	0.725720702	0.729538892
0.578882669	0.586032594	0.592937759	0.599758513	0.606696784	0.613529322	0.620106833
0.580820126	0.586712349	0.593388895	0.599896069	0.606042198	0.611923309	0.617652905
0.492754711	0.497692152	0.50185131	0.505975411	0.510504301	0.515611601	0.519870566
0.556102527	0.564024	0.572495886	0.581383179	0.590442387	0.599212061	0.607104618
0.259164883	0.268004611	0.277369193	0.287030583	0.29674042	0.306748843	0.316731153
0.678614636	0.685380936	0.692023301	0.698093383	0.704202308	0.710980184	0.716185465
0.781143992	0.788525951	0.796428086	0.805537023	0.814870878	0.822793435	0.828304165
0.799186111	0.80195087	0.804489194	0.806817046	0.809352772	0.812039287	0.814793642
0.307615488	0.313772983	0.320437339	0.327234002	0.334313237	0.34135263	0.348378035
0.73460985	0.744613598	0.754267558	0.763447496	0.772180593	0.780342623	0.787055146
0.803228025	0.805304692	0.80603308	0.806783583	0.80968805	0.814577102	0.820294574
0.631437774	0.634216906	0.63697577	0.640590696	0.646331714	0.653525522	0.660321135

0.578006538	0.583942986	0.589530777	0.594498057	0.599628055	0.604889158	0.610351745
0.400396456	0.403226097	0.406488572	0.410501729	0.414915578	0.419853237	0.42485465
0.555212224	0.550972256	0.553941679	0.56333419	0.575367174	0.586184887	0.593288422
0.513367511	0.52122939	0.528956332	0.536300653	0.543342652	0.550127119	0.556455944
0.324081689	0.333371285	0.343462562	0.353472947	0.363301218	0.373140288	0.382762252
0.333721398	0.339488917	0.346048864	0.353540709	0.362450967	0.371688496	0.381667118
0.446002182	0.443696404	0.439651657	0.434405758	0.42916514	0.422448974	0.418330613

2010	2011	2012	2013	2014	2015	2016
0.247759949	0.257041676	0.266484407	0.275636558	0.284030351	0.291849505	0.299630696
0.652654203	0.658196773	0.66355678	0.668739559	0.674112985	0.679668601	0.685036648
0.602823535	0.607825529	0.612703533	0.617422204	0.622087292	0.626745568	0.631710736
0.671248916	0.675979288	0.680861539	0.685833061	0.691343137	0.697162912	0.702726398
0.839489966	0.842794802	0.845810831	0.84832991	0.851010492	0.853591218	0.856257053
0.372033537	0.380006613	0.387891857	0.395796329	0.403720086	0.411372026	0.418822062
0.711432246	0.715489989	0.719509323	0.722864978	0.725585978	0.728372762	0.731598944
0.66992588	0.675242697	0.67921824	0.681633215	0.684974509	0.691710126	0.698618031
0.654811534	0.659818092	0.664833143	0.669534496	0.67393219	0.678054635	0.681746395
0.803561544	0.80752022	0.811644461	0.816453661	0.82084085	0.824860715	0.828559943
0.824500109	0.828001999	0.831113309	0.83367978	0.835838577	0.837579895	0.83988379
0.652056507	0.657971629	0.662897002	0.667658779	0.672162741	0.676244911	0.679807643
0.77411981	0.778281891	0.781838639	0.784623911	0.787253492	0.789956145	0.792738696
0.70790262	0.708958739	0.710362418	0.713343156	0.71676806	0.719926605	0.723611297
0.370598956	0.380124886	0.390623552	0.401706478	0.413263169	0.425482121	0.437709906
0.716208546	0.720060687	0.724162458	0.72782617	0.730383287	0.732602993	0.734912535
0.734954626	0.739504326	0.743468837	0.748529323	0.75351148	0.758205743	0.764328222
0.815030207	0.819470362	0.824226016	0.828619832	0.832425052	0.8358784	0.83946294
0.558546893	0.563440363	0.568456236	0.573236457	0.578091326	0.583279094	0.588275912
0.302809008	0.307925538	0.313373588	0.319318688	0.325727272	0.33221635	0.338763921
0.789181467	0.793380524	0.79748487	0.801388201	0.804923407	0.807998795	0.810550842
0.399751936	0.410149249	0.419443216	0.427790675	0.43496927	0.441810748	0.448401855
0.537009068	0.542680745	0.548615482	0.554788216	0.560934581	0.567000898	0.573080316
0.680824196	0.685399264	0.689506566	0.693508711	0.697031893	0.700446047	0.704079344
0.58582415	0.591985457	0.597739553	0.604064731	0.610086049	0.615411124	0.620797575
0.600445846	0.606777291	0.612619337	0.618218237	0.623433814	0.628149803	0.632235114
0.773219221	0.776631893	0.780216552	0.784017041	0.787733289	0.791367567	0.794980772
0.730701737	0.735749281	0.739177517	0.742135948	0.745372643	0.748979058	0.752259199
0.22100103	0.226696632	0.232589135	0.238497491	0.244378901	0.250208261	0.256081774
0.241447786	0.246277277	0.251572303	0.257191355	0.262794722	0.267327938	0.271587852
0.455647568	0.464473969	0.472857771	0.480796481	0.488259896	0.495229489	0.501855424
0.410211121	0.415964409	0.421769754	0.42771506	0.433597749	0.439487358	0.445358172
0.396067145	0.402092799	0.408580123	0.415607965	0.42303356	0.430830276	0.438878945
0.844853489	0.848017833	0.850978048	0.853844945	0.856759934	0.859532904	0.862174226
0.279981382	0.285097245	0.290460023	0.29010493	0.289999498	0.290827871	0.2924304
0.184110621	0.189591609	0.195262749	0.200952884	0.206658836	0.212428697	0.21753218
0.702304704	0.708597936	0.714743477	0.720448781	0.727275927	0.736098082	0.744972224
0.641521443	0.651447978	0.657144292	0.663103007	0.668640847	0.671940111	0.676218437
0.586408108	0.593340477	0.600254238	0.607250637	0.614151923	0.620889228	0.627554497
0.415543376	0.421092972	0.426729957	0.432789932	0.438844505	0.444712394	0.450506979
0.505542849	0.513815777	0.522928283	0.531551679	0.540594486	0.54841403	0.555108608
0.711861612	0.719444666	0.726963385	0.733317015	0.739595704	0.746550297	0.752353087
0.636595426	0.641201295	0.646574093	0.651945813	0.656890456	0.662030136	0.667430092
0.759472252	0.763686381	0.767405336	0.77115188	0.774479241	0.777865888	0.781443605
0.615261262	0.62038669	0.626156025	0.631839801	0.637304129	0.642818963	0.647907866
0.809840188	0.813726132	0.816978525	0.819380602	0.821184302	0.822732851	0.824283624
0.808747328	0.812054784	0.814230401	0.815615361	0.816227257	0.816564684	0.817153026

0.361205187	0.36408365	0.367831685	0.37235379	0.377575444	0.38349138	0.389825192
0.537192918	0.54121227	0.545331713	0.549483297	0.55360356	0.557321271	0.560743563
0.282223754	0.290872953	0.300454783	0.310831256	0.321622927	0.332421307	0.341988787
0.866767079	0.869778682	0.87251751	0.874932974	0.876814932	0.878543945	0.880589783
0.41699935	0.42231614	0.42771144	0.433360437	0.439455722	0.445855285	0.453442854
0.698489193	0.70246326	0.706870608	0.712210475	0.718455556	0.724785536	0.731760801
0.568130582	0.573834813	0.578966381	0.583625825	0.588236743	0.592924164	0.597619609
0.588763056	0.596729076	0.605454319	0.614080275	0.622027433	0.62854671	0.634281143
0.507715477	0.504594044	0.509157068	0.519334464	0.531749809	0.544370149	0.556012469
0.504171335	0.508969239	0.513396874	0.518121908	0.523468901	0.52938695	0.535650269
0.559109377	0.570277424	0.581833899	0.592800613	0.603473167	0.613134624	0.621858157
0.348146177	0.354163147	0.360112751	0.364343653	0.372527588	0.376935162	0.382171666
0.800829718	0.806633078	0.811109358	0.814887177	0.818770732	0.823221278	0.82736436
0.526245802	0.53191391	0.537798023	0.543836416	0.549613848	0.555373739	0.561061186
0.25608146	0.267880862	0.279006958	0.289885267	0.300484548	0.310736419	0.320431867
0.623308786	0.626438251	0.629881335	0.634278114	0.639390066	0.645026353	0.65074268
0.824844982	0.828270577	0.831434484	0.834425401	0.837431872	0.840878477	0.844622338
0.803240247	0.806378738	0.809615389	0.812984015	0.816551448	0.820238385	0.823730929
0.554022706	0.560232082	0.566842964	0.57380813	0.580915656	0.588365	0.596062305
0.34799783	0.352869236	0.35837365	0.364255878	0.3699013	0.375840485	0.381569217
0.677034892	0.681567044	0.685519223	0.688603268	0.692155207	0.697316133	0.70373149
0.87794671	0.881027508	0.883656408	0.885926953	0.887651614	0.888867854	0.890896369
0.480777892	0.489020974	0.497513932	0.506004647	0.514119061	0.521539202	0.528629586
0.775155815	0.778525867	0.780789483	0.781949052	0.782442013	0.782489523	0.782798443
0.803003131	0.808105185	0.811684562	0.814368308	0.81652706	0.817719189	0.818961902
0.616486997	0.620978521	0.624977512	0.629048336	0.633734696	0.638936084	0.644335234
0.761484317	0.763987394	0.766845984	0.770224957	0.773441684	0.776723614	0.780511644
0.470477364	0.476909684	0.481862853	0.487286692	0.494027188	0.500655607	0.507149792
0.262405949	0.2676246	0.273428319	0.279600444	0.285961264	0.292363614	0.299112412
0.291141639	0.296682967	0.301562342	0.306602009	0.311602905	0.317090015	0.32297967
0.580083911	0.585688269	0.591336582	0.596826295	0.602136511	0.607605487	0.61391436
0.411840105	0.41617719	0.420021528	0.423985549	0.427940902	0.43175676	0.435288722
0.452382226	0.458700329	0.464925155	0.470766833	0.476587799	0.482294176	0.487705932
0.767328649	0.769701942	0.770784245	0.771561698	0.772575046	0.774047383	0.776150305
0.841384642	0.84461993	0.847299444	0.850173948	0.853558697	0.857724976	0.861844467
0.467018996	0.476832909	0.487210876	0.498317981	0.5097221	0.521242719	0.532220023
0.585982419	0.593652985	0.601185701	0.608509904	0.615499887	0.622222469	0.628666625
0.64255917	0.647899089	0.651500587	0.654475289	0.657961511	0.662057987	0.667652445
0.542343278	0.553048555	0.564745467	0.576968998	0.588317652	0.599494007	0.611508646
0.828103848	0.831804792	0.835325359	0.838644433	0.842082115	0.847437887	0.852642502
0.77525953	0.777649669	0.780403415	0.783267167	0.786151084	0.788874457	0.791772445
0.778109206	0.780703468	0.783090981	0.785356877	0.787533956	0.789902119	0.792477244
0.642129388	0.64639583	0.65058376	0.654573624	0.658505341	0.662465809	0.666469279
0.844361811	0.846750267	0.849061489	0.851356994	0.853692749	0.856097499	0.858698173
0.659949495	0.66739668	0.674568925	0.681119962	0.68713404	0.692952032	0.698473302
0.686996643	0.691758952	0.695312767	0.699053593	0.703511921	0.708229736	0.71256904
0.433305839	0.44089383	0.448666396	0.456666942	0.464724302	0.473084051	0.481709072
0.482447689	0.485028211	0.488175864	0.491880767	0.495317241	0.500059699	0.505207837
0.784873929	0.791812065	0.798356988	0.804583834	0.810743233	0.816998413	0.823026527
0.556416545	0.558923335	0.561541574	0.565776769	0.570569357	0.575463231	0.580388551

0.414865566	0.423354523	0.431149361	0.438691039	0.445869424	0.452749603	0.459583996
0.798171594	0.799080776	0.801026474	0.80266049	0.803721486	0.806985995	0.811463591
0.668804525	0.680920617	0.693910983	0.704811001	0.712423873	0.718697292	0.724583347
0.448966476	0.454530098	0.460419689	0.466692256	0.473188157	0.479754456	0.486144827
0.282235752	0.288015145	0.294854315	0.302985656	0.31095544	0.318485166	0.325085174
0.711362496	0.711466657	0.716700693	0.71766525	0.716042207	0.713651367	0.71076014
0.793364682	0.800924994	0.807827026	0.813219722	0.817886376	0.823112629	0.829303695
0.853258277	0.856216834	0.859789031	0.863368792	0.866693556	0.870112937	0.87342088
0.318353184	0.323801043	0.329947541	0.336519232	0.343352611	0.350443595	0.358026341
0.29117962	0.300448509	0.309197624	0.317936213	0.327042426	0.336140138	0.344731542
0.688258622	0.694083403	0.699664734	0.704930717	0.709953756	0.714659871	0.719223425
0.581980986	0.58968446	0.596948519	0.604238519	0.611578071	0.61845439	0.62487595
0.201803536	0.207097221	0.21246506	0.217993867	0.22372998	0.229826263	0.236186231
0.7562505	0.760310547	0.76453788	0.76861614	0.772728741	0.777276905	0.78167341
0.516085138	0.521177087	0.526042019	0.5314463	0.536640131	0.541793167	0.546782936
0.423202436	0.42886729	0.434724376	0.44106891	0.447653881	0.454523488	0.461269545
0.6604698	0.666497627	0.67312765	0.680114023	0.686616503	0.69223973	0.697020562
0.599527028	0.603646095	0.608914289	0.615089993	0.621857405	0.628896724	0.635925349
0.550122546	0.554240411	0.557965853	0.561082821	0.56384003	0.567151342	0.570458101
0.889262841	0.891237592	0.893073822	0.894864472	0.896547874	0.898356337	0.900051303
0.574406669	0.578600663	0.583255886	0.588080811	0.592867353	0.596859648	0.600260767
0.743347873	0.750080328	0.75557838	0.760886907	0.765776527	0.770561278	0.77520077
0.476269799	0.483922152	0.491603392	0.499528607	0.507443265	0.515582893	0.523518592
0.2467515	0.252232471	0.258315562	0.265280462	0.272892814	0.281032685	0.289152425
0.460180429	0.467850872	0.475336423	0.482821855	0.490255145	0.497374156	0.504115978
0.552741565	0.559618899	0.566978697	0.574590344	0.582321425	0.58982104	0.596283059
0.535689995	0.542342743	0.549890621	0.560112906	0.571424364	0.581283234	0.590102988
0.3568681	0.364642605	0.372437588	0.380022326	0.387689088	0.395021154	0.401628776
0.861255298	0.864246678	0.866950626	0.869402979	0.871848914	0.874402841	0.876784052
0.805133185	0.809760352	0.814957564	0.82014599	0.823908944	0.827462493	0.831843967
0.472172	0.476593466	0.4812969	0.48610547	0.491207346	0.496391663	0.501853345
0.127952996	0.130958067	0.134469718	0.13797817	0.141731738	0.145440503	0.149200109
0.414997748	0.423351915	0.431780376	0.440124664	0.448766823	0.457240991	0.465183781
0.68628063	0.690315578	0.694150949	0.697221078	0.700545924	0.704232979	0.707476099
0.705589324	0.710592521	0.715173148	0.719671149	0.724089589	0.728603773	0.733173624
0.746617262	0.744411908	0.742937351	0.74228436	0.742605449	0.743797398	0.748455466
0.880378381	0.884351667	0.888160475	0.891989618	0.895499546	0.898912644	0.902584187
0.710688695	0.718264528	0.726450074	0.73343249	0.738743772	0.743597182	0.748797193
0.435640657	0.441455585	0.447096599	0.452823095	0.458781424	0.464792668	0.471022168
0.724065956	0.726733446	0.729612899	0.731953475	0.734768297	0.738166425	0.741361911
0.525711905	0.53677763	0.548295425	0.559163481	0.569239882	0.579204261	0.58949277
0.625116323	0.628633942	0.634263435	0.641324353	0.649108174	0.657962047	0.667276758
0.380286778	0.383587539	0.386781028	0.389926437	0.394004571	0.398285864	0.402407267
0.568846562	0.574954463	0.580650186	0.587247042	0.593953153	0.600484125	0.607018145
0.606520932	0.613051221	0.619355264	0.625519024	0.631114354	0.636278271	0.641285279
0.571765888	0.577240283	0.583767625	0.590837549	0.59838452	0.606215624	0.614224961
0.763184974	0.769407164	0.774680767	0.779434257	0.783682369	0.787445343	0.790651015
0.706563555	0.711262893	0.715804084	0.719836991	0.723021282	0.72575622	0.728300849
0.761763253	0.766784882	0.772311804	0.778461162	0.785120345	0.791800167	0.799084083
0.780807074	0.78721245	0.793494901	0.799611555	0.80572571	0.811872852	0.817979841

0.842511429	0.846913229	0.851140263	0.855441	0.859665568	0.863907408	0.868317824
0.671989616	0.678004137	0.682979796	0.688519175	0.694104557	0.699355166	0.705144542
0.723891349	0.729018272	0.733892482	0.737880473	0.740395467	0.74347353	0.74705871
0.763320116	0.767633588	0.771920384	0.776526979	0.780617524	0.784913774	0.790330149
0.351659416	0.35894034	0.366411821	0.373769682	0.381533179	0.389895415	0.398287959
0.714460415	0.719324381	0.723436152	0.726706593	0.729530219	0.732220672	0.735566138
0.632782905	0.637439442	0.641567127	0.645153079	0.648743507	0.65215748	0.65565334
0.587190384	0.590259096	0.592153908	0.594614075	0.598378672	0.603754135	0.61005921
0.559622905	0.562891574	0.565320388	0.567448536	0.569493661	0.571998983	0.575402051
0.879440814	0.881197518	0.882924531	0.884592627	0.885353694	0.884693365	0.884477665
0.405530666	0.415783728	0.425721315	0.43559246	0.445146237	0.454051473	0.463230082
0.740300669	0.749959451	0.759054935	0.767387864	0.775178133	0.782300802	0.788867882
0.329804072	0.336059897	0.342977114	0.350134004	0.357181573	0.364144496	0.371114525
0.740091311	0.750072762	0.757105082	0.762170908	0.766043091	0.769842223	0.773654077
0.68016364	0.683681491	0.687629394	0.692525734	0.697114589	0.70172282	0.706183623
0.274270275	0.281673575	0.290338512	0.300736693	0.310348867	0.31699865	0.324091565
0.83022216	0.833829608	0.837391807	0.840420178	0.842894538	0.845401857	0.847596999
0.786254422	0.791465382	0.795456216	0.797903723	0.799618796	0.80039418	0.80087821
0.821178486	0.823023561	0.824336563	0.825826275	0.827799246	0.829837071	0.831367915
0.38271759	0.388055337	0.39300464	0.39790625	0.402244745	0.406233897	0.410406962
0.064322128	0.065306811	0.066354879	0.067459486	0.068609299	0.069811521	0.071064047
0.635268538	0.639991722	0.644715661	0.649430554	0.653964495	0.658417761	0.662642487
0.270572622	0.274719143	0.275066143	0.275813942	0.276311461	0.275833634	0.274455491
0.738352055	0.74209645	0.745181867	0.747651982	0.749800929	0.752269823	0.754757939
0.634263123	0.640799571	0.648049816	0.655435838	0.662782434	0.669760292	0.676277441
0.436171097	0.447239442	0.456790265	0.466435995	0.476362135	0.486549437	0.496848219
0.595469745	0.600356336	0.60474187	0.608910612	0.612704423	0.616097315	0.618868088
0.858727941	0.861892379	0.864706672	0.867383768	0.869923325	0.872388515	0.874661655
0.910530522	0.913276573	0.915714893	0.917955006	0.919994182	0.921832991	0.923930598
0.595967764	0.601524456	0.602872601	0.602410134	0.60223576	0.602282914	0.602911181
0.827226752	0.832064074	0.836677665	0.841280463	0.84569556	0.850055269	0.854466038
0.495893964	0.499869804	0.503953885	0.507970676	0.511989518	0.515783245	0.519897002
0.62858944	0.633226728	0.638410972	0.643672916	0.648772057	0.654015623	0.659369303
0.394887019	0.401872356	0.4089103	0.415217878	0.421155538	0.42638362	0.431207272
0.335899583	0.34077726	0.346320495	0.352278605	0.358661442	0.365600169	0.372910028
0.63017554	0.636016518	0.641611991	0.647966148	0.653231999	0.658798995	0.663974319
0.580657299	0.582656723	0.58477088	0.587351141	0.590826853	0.594965748	0.600343669
0.733237077	0.736802405	0.739999073	0.743593955	0.747338963	0.751510682	0.754953348
0.626432774	0.63201984	0.637578345	0.643008512	0.648297395	0.653413538	0.658353517
0.623234589	0.629233722	0.635263183	0.641361893	0.647542417	0.65335279	0.658945613
0.52345551	0.527744245	0.531203235	0.535166235	0.539122244	0.54404788	0.549173928
0.615597228	0.624787211	0.634097092	0.643723823	0.653388835	0.662971739	0.671948148
0.326543661	0.336217716	0.345364515	0.354381615	0.363430903	0.372537986	0.381312226
0.72101987	0.724891957	0.7288172	0.734033573	0.739883557	0.743790863	0.746857989
0.831284254	0.832912804	0.83358611	0.833803818	0.833858727	0.834175498	0.83522071
0.818033994	0.821718995	0.826453754	0.831508668	0.835516642	0.838951919	0.842460233
0.355567563	0.36303632	0.370412488	0.377963171	0.385974579	0.394277757	0.402879308
0.793219696	0.798071698	0.801092851	0.803405789	0.805641378	0.807889245	0.810199044
0.825884658	0.830266388	0.833958192	0.837412988	0.840746528	0.844370336	0.848015521
0.666099135	0.667079733	0.665802192	0.668228361	0.67333426	0.680595506	0.689816733

0.616097907	0.621344619	0.626202125	0.630796403	0.63525705	0.639805639	0.644230597
0.429658049	0.434283044	0.438661998	0.442720176	0.446687298	0.450329861	0.454072763
0.599127923	0.604823484	0.610187465	0.614441024	0.614686806	0.610675357	0.606993213
0.562768571	0.568945458	0.575078672	0.581029215	0.586913815	0.592849573	0.598849485
0.392936641	0.401471801	0.409561349	0.417642405	0.425189667	0.430197789	0.434232983
0.392470989	0.403475629	0.414574501	0.425492302	0.436244006	0.446744719	0.457122596
0.417305357	0.419729695	0.42574852	0.432881888	0.439997226	0.446552872	0.452473037

2017	2018	2019	2020	2021
0.307424618	0.314866093	0.322454047	0.329830068	0.337199998
0.690379199	0.695566878	0.700287863	0.703790245	0.706849791
0.636973176	0.642500039	0.648210785	0.653651466	0.659500924
0.707647909	0.712800591	0.717308133	0.7208385	0.723727533
0.85947703	0.862588951	0.865621024	0.867449169	0.869444113
0.426177082	0.433355005	0.440454598	0.447283992	0.453721949
0.734963713	0.738879106	0.74305296	0.74634533	0.749886887
0.705135529	0.712888693	0.719002843	0.721294303	0.723122973
0.685980265	0.690276872	0.694765962	0.698268899	0.701833194
0.831798266	0.835354624	0.839317426	0.842051314	0.844252814
0.843173331	0.84661447	0.849862515	0.852020385	0.853837004
0.683263204	0.686561093	0.689834715	0.692605192	0.694851274
0.795347181	0.798179034	0.80103307	0.802948018	0.805020668
0.729323103	0.736192583	0.742847804	0.748103083	0.753043204
0.449752413	0.461717749	0.473264998	0.483079169	0.492420885
0.737157255	0.739531437	0.742239309	0.744366646	0.746748764
0.771131466	0.776568957	0.780508019	0.782381565	0.784484711
0.843212044	0.846617625	0.849498395	0.851346649	0.853654016
0.59333729	0.598512015	0.603351601	0.607060046	0.610229002
0.345606759	0.352652696	0.360031163	0.366964487	0.373486574
0.812807229	0.814892802	0.817000338	0.81920342	0.821365422
0.454280497	0.459624711	0.464633353	0.468714479	0.473062378
0.579241285	0.585184106	0.590692043	0.594854443	0.599010799
0.708053431	0.712347302	0.716857125	0.720202056	0.723077893
0.625704953	0.630606841	0.635324093	0.639275298	0.642721629
0.636422188	0.640802974	0.645298005	0.649201568	0.653043887
0.798309936	0.801436553	0.804499388	0.807424091	0.810234367
0.755665865	0.75914846	0.762359791	0.765089711	0.768150939
0.262033009	0.268102073	0.274287092	0.279871317	0.285118402
0.275840505	0.27973329	0.283442515	0.286496	0.289374365
0.508435792	0.515285886	0.522488008	0.528461595	0.533534539
0.451221744	0.45734149	0.46364343	0.468876054	0.473621491
0.4471851	0.455687847	0.464246473	0.472285846	0.479691223
0.86505383	0.867905541	0.870554479	0.871992631	0.87317068
0.294752243	0.298011223	0.301953064	0.305426298	0.30916769
0.22220695	0.226913881	0.231704755	0.236103083	0.240436019
0.752058077	0.758625714	0.765120323	0.769213676	0.771514716
0.684619661	0.693717996	0.703686824	0.713364585	0.72162976
0.634130359	0.640255072	0.646038852	0.650855354	0.655442913
0.456280841	0.461951344	0.467388014	0.472003132	0.475978688
0.5618341	0.56779397	0.573373665	0.578451546	0.583075236
0.758233572	0.764041182	0.769795913	0.774700578	0.779109955
0.673621392	0.681296147	0.690049088	0.696194605	0.700340477
0.785074537	0.78867141	0.792475415	0.795462272	0.798341027
0.652309286	0.656858654	0.661674717	0.665210747	0.668729864
0.826139878	0.828343243	0.830980728	0.833059523	0.835630545
0.81906768	0.822034486	0.824784473	0.826626631	0.828450433

0.396693974	0.404091933	0.41172558	0.419040103	0.425941883
0.563388289	0.565161484	0.566765706	0.568188551	0.569854634
0.351102035	0.360287677	0.369340684	0.376712271	0.383179849
0.883688487	0.887529846	0.891624571	0.894368297	0.896424204
0.460440152	0.467705672	0.475263243	0.481879945	0.487958371
0.736062473	0.738935954	0.742132066	0.744419944	0.746967185
0.602140291	0.606960839	0.611913021	0.615635625	0.619388201
0.640011219	0.64588852	0.651787987	0.656714457	0.661017053
0.566914632	0.577442786	0.587736877	0.597363341	0.606787094
0.541998957	0.548143174	0.554031648	0.558919894	0.563775188
0.630234538	0.638022823	0.645303093	0.652124903	0.657857456
0.385936029	0.391154884	0.396330558	0.400459411	0.403863943
0.830975311	0.834566296	0.838573509	0.841817139	0.844917787
0.566698493	0.572154807	0.577206305	0.581653071	0.585459713
0.329469244	0.338050075	0.346422664	0.35321635	0.358823295
0.65646401	0.661991454	0.667060979	0.671431491	0.675051631
0.848586128	0.852202943	0.855365179	0.857655553	0.859831368
0.827098441	0.83044859	0.833772805	0.836049443	0.838364875
0.603816252	0.61172438	0.619904727	0.627609647	0.634691393
0.387273644	0.393155679	0.39918978	0.404524194	0.40971416
0.710874718	0.717993654	0.724536462	0.729125889	0.732473604
0.894050472	0.896975545	0.899703158	0.901438614	0.902957091
0.53616994	0.543840539	0.551632027	0.558462173	0.56493039
0.784108594	0.785999753	0.788138138	0.78963222	0.791854408
0.819781101	0.820815514	0.822339996	0.824359028	0.826210336
0.649831522	0.655372772	0.660808512	0.665086347	0.668993028
0.78555492	0.791294773	0.796775452	0.800969472	0.803982203
0.513735682	0.520995469	0.52863047	0.534571414	0.539972424
0.306881462	0.314839588	0.322839848	0.329877997	0.336401293
0.329326492	0.335667833	0.342156115	0.347798998	0.353109621
0.620602607	0.627167041	0.633640347	0.642284645	0.650812335
0.438541003	0.441678925	0.444328854	0.446390614	0.448278285
0.493130694	0.498657227	0.504156505	0.508669246	0.513037248
0.779116417	0.782083661	0.784910309	0.78762039	0.790754768
0.865702891	0.869219822	0.87242558	0.874323153	0.87636168
0.542388507	0.551930326	0.560809927	0.568138149	0.575401649
0.634895745	0.64094875	0.646865457	0.651926648	0.656868336
0.674343462	0.680909621	0.686741662	0.691918763	0.697207398
0.622674532	0.633232108	0.643635318	0.65330038	0.662626231
0.857816164	0.863925284	0.869412503	0.871958822	0.87375385
0.795173437	0.799124994	0.803232991	0.806351589	0.809011652
0.795311186	0.79836269	0.801530021	0.80363568	0.805773534
0.670291045	0.674075313	0.677778539	0.680567177	0.683263064
0.861393309	0.864237137	0.867148183	0.869106879	0.871241813
0.703923826	0.709412879	0.714998946	0.72006824	0.725307227
0.716026688	0.718531999	0.720797602	0.722788768	0.725144495
0.490296619	0.499101274	0.508004211	0.516136572	0.523768077
0.510019241	0.514810384	0.519441321	0.523454113	0.527186583
0.828886987	0.834371461	0.839688307	0.843459812	0.846651055
0.585766096	0.591237134	0.596501638	0.600499554	0.603979328

0.466168892	0.472618103	0.478811373	0.484192959	0.489136091
0.816923118	0.821419809	0.824781777	0.827900448	0.830663516
0.729963294	0.734746243	0.738644009	0.74200972	0.744746351
0.491978155	0.497381791	0.502457317	0.506746655	0.510393066
0.331527786	0.337784578	0.343403262	0.34813389	0.352442452
0.710666264	0.712720438	0.716157239	0.720270393	0.725771399
0.83587174	0.84191891	0.847906339	0.852752388	0.856484049
0.876347684	0.878637895	0.880593895	0.882495316	0.884428955
0.366235587	0.374969644	0.384209196	0.392778709	0.400246943
0.353121067	0.36147041	0.370114605	0.37787902	0.384553634
0.724235412	0.729388078	0.734587751	0.738743993	0.742523828
0.631083288	0.637205659	0.643082794	0.647608589	0.650886627
0.242726135	0.249419731	0.256250746	0.262629219	0.268579941
0.786410184	0.790965618	0.795197846	0.798375166	0.801585034
0.552065152	0.557599278	0.56342875	0.568837509	0.574091128
0.468452365	0.475839507	0.483767738	0.491365603	0.4989451
0.701618136	0.706266651	0.710935045	0.714629804	0.718260446
0.642667198	0.649053317	0.655095338	0.660119062	0.664575304
0.574039833	0.577555081	0.581195255	0.584527229	0.587534967
0.901752712	0.903388687	0.905030991	0.906685437	0.908262831
0.603721965	0.607501833	0.611250681	0.614629637	0.617621565
0.779764632	0.784408971	0.789026903	0.792554393	0.795800584
0.531610862	0.539729671	0.547837444	0.555246069	0.562698301
0.297164772	0.305226772	0.313174508	0.320291182	0.326462614
0.510823431	0.517557469	0.52410897	0.529434703	0.53390084
0.601754645	0.606618403	0.610921091	0.614435889	0.617564872
0.597619076	0.605311879	0.612697253	0.619330076	0.625177834
0.408463874	0.415236684	0.422007252	0.427417089	0.433174635
0.879300152	0.881900218	0.88464384	0.886558566	0.888464256
0.836301795	0.840597863	0.844533209	0.847398737	0.849442499
0.507482022	0.512338228	0.516597046	0.52029267	0.523958472
0.15295917	0.156875196	0.160889484	0.164539265	0.168072774
0.472972869	0.480844413	0.488787361	0.496204741	0.503390833
0.711220996	0.71510823	0.719078885	0.72279025	0.72622205
0.737339955	0.74133437	0.745193569	0.7478499	0.750629703
0.756495479	0.7612554	0.765770647	0.76942766	0.771535213
0.906044049	0.909261782	0.912278761	0.91452992	0.91613281
0.753786058	0.759179514	0.764527863	0.768854216	0.773391602
0.477544137	0.484326128	0.491022981	0.497364406	0.504028689
0.744224767	0.74776125	0.750715307	0.752780929	0.754046931
0.599157187	0.608124861	0.616466839	0.623810015	0.631011665
0.676783503	0.686719925	0.69667068	0.70378849	0.708864828
0.406153435	0.409165083	0.412311147	0.415186879	0.417797443
0.613581591	0.620032362	0.626070139	0.631057689	0.635718099
0.646180855	0.650977317	0.655433735	0.658672244	0.662054037
0.622315029	0.630464276	0.638547384	0.644867233	0.651219329
0.794530345	0.799478982	0.804791332	0.808795318	0.812042809
0.731225535	0.734402947	0.738090481	0.741037384	0.744151851
0.806648961	0.812906066	0.818843191	0.822918436	0.825525847
0.824016868	0.829962127	0.835805284	0.841467395	0.846860584

0.872617764	0.876823317	0.88073151	0.883717569	0.886675267
0.711206637	0.717244427	0.723011648	0.72771054	0.732214875
0.751117367	0.755533333	0.760283971	0.764276444	0.768453864
0.795669484	0.800108144	0.803658201	0.806011474	0.808536005
0.406411475	0.414657912	0.423086727	0.429896596	0.435588706
0.739470681	0.743736461	0.748152066	0.751568409	0.754987055
0.659341346	0.663086682	0.666840359	0.66972049	0.672509735
0.616438473	0.622634944	0.628325986	0.632860904	0.637195963
0.578925435	0.582320661	0.586256695	0.590075296	0.593392769
0.884557607	0.885133516	0.88620066	0.886867937	0.888005474
0.47217093	0.481098204	0.48983823	0.497861409	0.505413747
0.794886288	0.800656985	0.806087841	0.810541861	0.815143493
0.378351477	0.386079666	0.394012316	0.401197516	0.408054193
0.777460384	0.781494974	0.7857683	0.789183333	0.792416294
0.711027281	0.716407819	0.722073685	0.726663769	0.730150775
0.331213431	0.338341564	0.345770019	0.352412022	0.358665881
0.849051518	0.850762729	0.852497338	0.85429601	0.856097766
0.802277317	0.804357302	0.806448457	0.808305966	0.81061053
0.833038139	0.835472481	0.838266782	0.840373846	0.842430731
0.414676394	0.418889221	0.422736386	0.426112429	0.429360316
0.072334776	0.073642248	0.074979811	0.07636337	0.077688109
0.666622608	0.670335365	0.674041037	0.677166096	0.679626598
0.274387859	0.274987848	0.275706201	0.276787924	0.278371125
0.757663276	0.760659575	0.763942918	0.766506025	0.769283698
0.682405274	0.688125858	0.693516063	0.697648535	0.701534935
0.506883534	0.516214086	0.525009122	0.533455422	0.541949735
0.62168418	0.624708011	0.627896781	0.630683705	0.633665739
0.877120611	0.879888912	0.882984116	0.88506201	0.886880299
0.926202291	0.92855059	0.930682102	0.932027645	0.933059111
0.604896366	0.608507164	0.613336836	0.617727593	0.623004075
0.858888019	0.863158361	0.86741185	0.871247947	0.874747053
0.524492607	0.529153916	0.533769951	0.537690531	0.541511187
0.664804134	0.670225587	0.675273901	0.679119989	0.682547933
0.434519734	0.437018663	0.440195845	0.442349336	0.444667619
0.380336231	0.387921229	0.395667269	0.402356922	0.408533695
0.669054134	0.674179514	0.679247839	0.682937378	0.686425621
0.606243592	0.611953942	0.617324063	0.622104456	0.626349936
0.758226988	0.761241991	0.764169599	0.766422081	0.768763254
0.663222845	0.668218997	0.673170552	0.677615148	0.682432216
0.664284692	0.669433599	0.674303536	0.67829818	0.682160776
0.55446648	0.559859395	0.566045489	0.571666102	0.576620529
0.681096272	0.689895867	0.698263774	0.705799744	0.712692673
0.390082259	0.399117777	0.408431557	0.416846079	0.423261181
0.750593324	0.754451727	0.757980081	0.759929872	0.760773913
0.837213553	0.83990014	0.842985326	0.846151976	0.849317734
0.846263618	0.849969788	0.854130573	0.85692029	0.859000182
0.411824974	0.420985364	0.430225379	0.438747238	0.446568273
0.812405172	0.81482544	0.817429008	0.819601712	0.821830853
0.851630299	0.855049193	0.858578065	0.860792773	0.862448354
0.699189456	0.70690493	0.712536588	0.716169766	0.719283445

0.648119347	0.651918144	0.655836959	0.659124204	0.662621694
0.458016834	0.462080221	0.466276162	0.470038391	0.473100706
0.605160131	0.604068247	0.602313887	0.600034781	0.596513059
0.605019421	0.611351406	0.617733815	0.62309032	0.627933721
0.437549155	0.440727991	0.4440014	0.446893069	0.450376375
0.467532631	0.477887712	0.487911523	0.497485561	0.505948954
0.458424984	0.464379262	0.468384438	0.471573171	0.473819486

**Table S4. Trends in Global, SDI Regions, and GBD Regions Ischemic Heart Dise
Number of Deaths and Age-Standardized Mortality Rate (ASMR), 19**

location_id	location_name	year	Number_UI
1	Global	1990	5367136.58 (5076403.86,5562773.87)
1	Global	1991	5447159.32 (5173861.47,5648148.87)
1	Global	1992	5575981.00 (5296293.50,5782569.21)
1	Global	1993	5806592.83 (5501074.73,6010558.34)
1	Global	1994	5942545.44 (5667880.95,6147185.23)
1	Global	1995	6008444.87 (5703658.27,6200095.63)
1	Global	1996	6018333.12 (5716455.17,6215190.02)
1	Global	1997	6033520.72 (5724244.31,6241350.91)
1	Global	1998	6094290.54 (5762858.12,6296502.71)
1	Global	1999	6221304.70 (5881593.88,6430682.63)
1	Global	2000	6302616.16 (5962705.15,6520776.09)
1	Global	2001	6410928.54 (6063643.60,6619307.08)
1	Global	2002	6569961.07 (6219603.64,6777359.03)
1	Global	2003	6732156.71 (6357694.06,6946606.43)
1	Global	2004	6772362.54 (6380051.77,6998882.88)
1	Global	2005	6893998.67 (6531657.31,7114529.84)
1	Global	2006	6879172.93 (6483862.12,7123566.50)
1	Global	2007	6982528.71 (6593805.66,7241212.23)
1	Global	2008	7154405.45 (6729810.46,7417278.36)
1	Global	2009	7252168.92 (6828088.39,7506660.99)
1	Global	2010	7429319.70 (6960144.82,7690364.06)
1	Global	2011	7566050.62 (7089926.95,7861224.50)
1	Global	2012	7718432.43 (7241311.45,8023194.52)
1	Global	2013	7891277.44 (7353477.94,8231459.69)
1	Global	2014	8041084.30 (7504798.11,8406603.35)
1	Global	2015	8134518.01 (7571341.94,8482075.43)
1	Global	2016	8276731.25 (7682159.03,8658525.50)
1	Global	2017	8362271.07 (7760063.22,8783241.53)
1	Global	2018	8548346.91 (7938485.70,8982674.22)
1	Global	2019	8714714.89 (8104137.84,9159318.56)
1	Global	2020	8839380.68 (8156472.02,9344777.54)
1	Global	2021	8991636.68 (8264123.21,9531130.17)
44635	High SDI	1990	1732392.15 (1591274.95,1797849.33)
44635	High SDI	1991	1726173.44 (1582391.88,1793364.81)
44635	High SDI	1992	1710831.60 (1565311.60,1779232.46)
44635	High SDI	1993	1719086.51 (1569128.67,1787018.21)
44635	High SDI	1994	1698026.35 (1549977.78,1765965.05)
44635	High SDI	1995	1686035.51 (1538090.50,1755445.14)
44635	High SDI	1996	1657891.60 (1506979.36,1727452.29)
44635	High SDI	1997	1633129.99 (1481514.13,1704465.17)
44635	High SDI	1998	1622582.67 (1469977.50,1695795.33)
44635	High SDI	1999	1624903.39 (1470418.48,1698657.81)
44635	High SDI	2000	1581111.49 (1426628.02,1655846.62)
44635	High SDI	2001	1559377.35 (1402215.86,1635809.75)
44635	High SDI	2002	1545829.09 (1392722.21,1623555.78)
44635	High SDI	2003	1526080.03 (1371479.95,1603902.91)

44635	High SDI	2004	1478948.19 (1324740.68,1555086.73)
44635	High SDI	2005	1462180.67 (1309535.26,1539050.69)
44635	High SDI	2006	1434368.41 (1279538.95,1512438.69)
44635	High SDI	2007	1413436.86 (1258815.32,1490291.12)
44635	High SDI	2008	1402300.08 (1243305.05,1478789.67)
44635	High SDI	2009	1382582.87 (1228003.34,1460169.64)
44635	High SDI	2010	1363349.34 (1205918.85,1441727.23)
44635	High SDI	2011	1357371.19 (1202371.25,1438389.49)
44635	High SDI	2012	1354425.08 (1192390.45,1434817.99)
44635	High SDI	2013	1356434.66 (1198322.25,1439855.60)
44635	High SDI	2014	1347609.17 (1186816.98,1430478.20)
44635	High SDI	2015	1357950.04 (1196890.84,1443130.41)
44635	High SDI	2016	1363078.41 (1203569.70,1446982.19)
44635	High SDI	2017	1368261.08 (1202260.22,1455556.01)
44635	High SDI	2018	1370751.10 (1199439.42,1458297.48)
44635	High SDI	2019	1365798.96 (1194200.73,1455885.34)
44635	High SDI	2020	1369467.08 (1195713.99,1460438.76)
44635	High SDI	2021	1392371.05 (1217171.56,1489069.66)
44634	High-middle SDI	1990	1604862.17 (1527808.26,1657939.76)
44634	High-middle SDI	1991	1642157.68 (1564119.01,1695009.71)
44634	High-middle SDI	1992	1718885.04 (1637016.88,1771562.19)
44634	High-middle SDI	1993	1878103.85 (1785271.85,1928785.13)
44634	High-middle SDI	1994	1971068.71 (1887606.39,2021146.68)
44634	High-middle SDI	1995	1978176.64 (1890550.89,2033348.87)
44634	High-middle SDI	1996	1954096.77 (1864242.90,2006800.08)
44634	High-middle SDI	1997	1933750.29 (1836683.46,1987250.14)
44634	High-middle SDI	1998	1935119.46 (1839054.10,1991312.13)
44634	High-middle SDI	1999	2022801.76 (1930257.64,2081136.42)
44634	High-middle SDI	2000	2079213.59 (1984951.07,2140498.49)
44634	High-middle SDI	2001	2119548.07 (2019382.92,2179294.54)
44634	High-middle SDI	2002	2195397.97 (2095154.31,2256907.04)
44634	High-middle SDI	2003	2264310.69 (2154416.37,2329340.05)
44634	High-middle SDI	2004	2269393.73 (2151727.51,2334766.49)
44634	High-middle SDI	2005	2330865.57 (2216016.31,2395085.76)
44634	High-middle SDI	2006	2265046.65 (2147780.01,2332701.20)
44634	High-middle SDI	2007	2274014.03 (2148150.72,2348886.17)
44634	High-middle SDI	2008	2314676.58 (2178068.29,2391650.53)
44634	High-middle SDI	2009	2310473.39 (2183540.82,2389363.60)
44634	High-middle SDI	2010	2359814.95 (2210869.93,2440093.83)
44634	High-middle SDI	2011	2351918.80 (2206466.01,2445968.36)
44634	High-middle SDI	2012	2354757.63 (2191165.04,2464005.45)
44634	High-middle SDI	2013	2359540.42 (2192320.12,2466482.61)
44634	High-middle SDI	2014	2367188.01 (2206300.63,2490446.45)
44634	High-middle SDI	2015	2369233.97 (2195101.25,2487670.15)
44634	High-middle SDI	2016	2374285.71 (2175936.49,2493644.24)
44634	High-middle SDI	2017	2369567.06 (2169635.61,2514222.43)
44634	High-middle SDI	2018	2393198.63 (2172414.33,2549451.26)
44634	High-middle SDI	2019	2414922.79 (2205001.63,2570673.39)
44634	High-middle SDI	2020	2424306.67 (2202858.49,2594658.22)
44634	High-middle SDI	2021	2450426.35 (2218836.45,2647300.49)

44637	Low SDI	1990	228811.49 (202386.13,255182.86)
44637	Low SDI	1991	233499.34 (207570.61,258590.64)
44637	Low SDI	1992	238732.51 (212820.21,264950.61)
44637	Low SDI	1993	244935.35 (218413.37,269854.36)
44637	Low SDI	1994	253711.20 (228795.44,281093.00)
44637	Low SDI	1995	260240.12 (233933.58,285804.54)
44637	Low SDI	1996	265030.77 (237010.67,290943.57)
44637	Low SDI	1997	272206.92 (245425.56,299078.38)
44637	Low SDI	1998	279571.05 (251604.96,305716.98)
44637	Low SDI	1999	282025.76 (255383.69,309900.94)
44637	Low SDI	2000	287688.23 (259269.50,315639.24)
44637	Low SDI	2001	293533.09 (266231.99,319352.96)
44637	Low SDI	2002	299596.44 (269675.07,326765.43)
44637	Low SDI	2003	306098.92 (274834.89,332410.03)
44637	Low SDI	2004	308372.02 (277368.84,332916.75)
44637	Low SDI	2005	310076.43 (282772.14,334774.30)
44637	Low SDI	2006	318404.87 (289024.92,343990.88)
44637	Low SDI	2007	328889.55 (297824.65,354453.12)
44637	Low SDI	2008	339664.52 (309619.25,365466.55)
44637	Low SDI	2009	349006.56 (317273.85,377031.99)
44637	Low SDI	2010	360098.70 (329352.15,389415.01)
44637	Low SDI	2011	375777.48 (341471.06,409391.65)
44637	Low SDI	2012	397063.30 (360403.81,434083.15)
44637	Low SDI	2013	412741.85 (372972.79,447453.93)
44637	Low SDI	2014	428562.16 (388435.55,465903.58)
44637	Low SDI	2015	439598.15 (397079.64,478609.53)
44637	Low SDI	2016	449900.50 (409443.40,488625.45)
44637	Low SDI	2017	451523.66 (411533.54,491455.54)
44637	Low SDI	2018	462276.93 (419043.80,503113.08)
44637	Low SDI	2019	476790.18 (434993.29,518875.69)
44637	Low SDI	2020	488030.25 (445571.95,535812.08)
44637	Low SDI	2021	493475.84 (448128.79,543115.16)
44636	Low-middle SDI	1990	753129.84 (698697.09,804552.24)
44636	Low-middle SDI	1991	766908.88 (710462.34,818634.97)
44636	Low-middle SDI	1992	788543.90 (731695.51,841412.73)
44636	Low-middle SDI	1993	809562.71 (755505.79,861787.66)
44636	Low-middle SDI	1994	833097.19 (779125.52,880245.98)
44636	Low-middle SDI	1995	860120.78 (807270.24,911340.69)
44636	Low-middle SDI	1996	883354.11 (828532.33,938407.59)
44636	Low-middle SDI	1997	911228.64 (850727.32,959105.36)
44636	Low-middle SDI	1998	938824.33 (881034.92,992957.38)
44636	Low-middle SDI	1999	944958.64 (886411.79,1002169.86)
44636	Low-middle SDI	2000	960134.90 (898790.77,1015515.65)
44636	Low-middle SDI	2001	992181.97 (933061.86,1045302.22)
44636	Low-middle SDI	2002	1024263.21 (964915.00,1079758.23)
44636	Low-middle SDI	2003	1059121.43 (997373.67,1111069.70)
44636	Low-middle SDI	2004	1076497.13 (1017201.14,1135461.70)
44636	Low-middle SDI	2005	1102458.86 (1037105.02,1162579.26)
44636	Low-middle SDI	2006	1151780.49 (1083704.58,1213239.93)
44636	Low-middle SDI	2007	1202671.36 (1137908.72,1261611.23)

44636	Low-middle SDI	2008	1249592.50 (1180672.05,1309877.48)
44636	Low-middle SDI	2009	1282969.91 (1213472.60,1346084.73)
44636	Low-middle SDI	2010	1330618.25 (1258117.01,1393364.66)
44636	Low-middle SDI	2011	1383906.12 (1303677.02,1454370.95)
44636	Low-middle SDI	2012	1448040.29 (1365799.48,1524420.91)
44636	Low-middle SDI	2013	1504580.55 (1417359.67,1577989.27)
44636	Low-middle SDI	2014	1572730.42 (1480322.26,1650977.07)
44636	Low-middle SDI	2015	1591168.53 (1493786.32,1673189.38)
44636	Low-middle SDI	2016	1635951.98 (1538193.07,1717292.05)
44636	Low-middle SDI	2017	1665136.58 (1569285.61,1753214.90)
44636	Low-middle SDI	2018	1733992.36 (1634313.02,1825513.45)
44636	Low-middle SDI	2019	1782936.38 (1675958.08,1881446.83)
44636	Low-middle SDI	2020	1811070.36 (1696043.51,1932838.51)
44636	Low-middle SDI	2021	1834652.91 (1699506.18,1964078.89)
44639	Middle SDI	1990	1038968.25 (978516.15,1100277.11)
44639	Middle SDI	1991	1069215.37 (1014355.49,1125461.95)
44639	Middle SDI	1992	1109561.62 (1044988.09,1170866.36)
44639	Middle SDI	1993	1145275.70 (1085622.20,1204511.29)
44639	Middle SDI	1994	1176850.77 (1120489.67,1233936.66)
44639	Middle SDI	1995	1213816.63 (1150755.41,1273305.78)
44639	Middle SDI	1996	1247777.90 (1187441.20,1302326.25)
44639	Middle SDI	1997	1272892.31 (1211163.01,1330902.75)
44639	Middle SDI	1998	1308430.99 (1243235.16,1363235.50)
44639	Middle SDI	1999	1337529.52 (1272690.29,1398472.65)
44639	Middle SDI	2000	1385816.96 (1318552.89,1442280.30)
44639	Middle SDI	2001	1437628.86 (1367754.33,1496990.80)
44639	Middle SDI	2002	1496214.24 (1426270.39,1560478.44)
44639	Middle SDI	2003	1567797.27 (1493751.78,1625371.61)
44639	Middle SDI	2004	1630431.83 (1552384.35,1695706.56)
44639	Middle SDI	2005	1679593.20 (1602913.20,1744361.14)
44639	Middle SDI	2006	1700800.70 (1617041.70,1765995.42)
44639	Middle SDI	2007	1754703.85 (1667323.31,1836001.38)
44639	Middle SDI	2008	1839304.32 (1744160.28,1923437.82)
44639	Middle SDI	2009	1918178.54 (1820928.38,2001815.52)
44639	Middle SDI	2010	2006491.08 (1894602.63,2097067.15)
44639	Middle SDI	2011	2088140.50 (1972762.80,2187784.42)
44639	Middle SDI	2012	2155080.78 (2028655.62,2272719.24)
44639	Middle SDI	2013	2248918.99 (2093356.94,2366942.38)
44639	Middle SDI	2014	2315901.98 (2175905.87,2446785.73)
44639	Middle SDI	2015	2367268.94 (2205689.94,2492971.53)
44639	Middle SDI	2016	2444233.29 (2243739.90,2598447.08)
44639	Middle SDI	2017	2498360.59 (2326590.24,2658528.00)
44639	Middle SDI	2018	2578642.59 (2390586.48,2732360.10)
44639	Middle SDI	2019	2664762.21 (2463091.77,2861617.65)
44639	Middle SDI	2020	2736908.15 (2522721.73,2933664.92)
44639	Middle SDI	2021	2811121.62 (2564657.44,3023433.63)
5	East Asia	1990	570428.71 (505994.31,639930.43)
5	East Asia	1991	587827.92 (529066.05,655880.71)
5	East Asia	1992	605063.97 (540088.93,673915.66)
5	East Asia	1993	623825.54 (558551.38,693188.62)

5	East Asia	1994	639210.19 (582767.37,702175.34)
5	East Asia	1995	658903.03 (603691.29,720513.60)
5	East Asia	1996	678136.30 (622902.74,734571.05)
5	East Asia	1997	698703.11 (642099.52,759581.20)
5	East Asia	1998	730872.52 (672567.52,791310.35)
5	East Asia	1999	770481.54 (708055.18,837915.75)
5	East Asia	2000	831574.35 (763956.41,894749.18)
5	East Asia	2001	888108.95 (815061.94,955875.62)
5	East Asia	2002	952702.99 (886660.89,1023505.61)
5	East Asia	2003	1045425.80 (974709.09,1122477.27)
5	East Asia	2004	1125609.76 (1045763.31,1204008.76)
5	East Asia	2005	1170388.39 (1083792.04,1252292.17)
5	East Asia	2006	1156535.18 (1063038.33,1238637.49)
5	East Asia	2007	1193115.27 (1106075.01,1290089.23)
5	East Asia	2008	1266225.71 (1165466.80,1358093.09)
5	East Asia	2009	1349920.66 (1241640.73,1445650.10)
5	East Asia	2010	1437749.26 (1307248.65,1556888.75)
5	East Asia	2011	1517920.47 (1384037.91,1650860.27)
5	East Asia	2012	1550857.78 (1413558.32,1709570.13)
5	East Asia	2013	1626761.22 (1455974.97,1786205.50)
5	East Asia	2014	1665136.10 (1484752.62,1836610.18)
5	East Asia	2015	1683077.39 (1491705.14,1863282.79)
5	East Asia	2016	1727245.81 (1510474.54,1938472.97)
5	East Asia	2017	1780824.16 (1557696.84,2028388.01)
5	East Asia	2018	1828259.29 (1594138.78,2101329.38)
5	East Asia	2019	1883248.53 (1625625.13,2153760.00)
5	East Asia	2020	1944502.84 (1675539.48,2246590.74)
5	East Asia	2021	2008011.06 (1683969.69,2335232.27)
9	Southeast Asia	1990	252646.98 (229370.47,275073.75)
9	Southeast Asia	1991	258972.59 (236472.74,282994.34)
9	Southeast Asia	1992	268634.74 (246631.70,292223.44)
9	Southeast Asia	1993	277898.26 (256522.67,299525.85)
9	Southeast Asia	1994	287513.22 (265004.67,309516.45)
9	Southeast Asia	1995	297156.62 (275847.49,320340.88)
9	Southeast Asia	1996	306465.91 (284445.96,327401.33)
9	Southeast Asia	1997	313855.73 (292242.56,336389.39)
9	Southeast Asia	1998	323958.24 (301432.28,347290.59)
9	Southeast Asia	1999	336537.81 (315233.47,358759.58)
9	Southeast Asia	2000	345705.35 (324140.15,369376.70)
9	Southeast Asia	2001	354765.90 (333120.84,379236.42)
9	Southeast Asia	2002	367996.75 (342947.64,390813.23)
9	Southeast Asia	2003	380228.74 (356650.05,405205.49)
9	Southeast Asia	2004	392971.40 (367543.54,419656.49)
9	Southeast Asia	2005	407037.10 (383067.35,432270.90)
9	Southeast Asia	2006	419066.97 (390533.35,446237.06)
9	Southeast Asia	2007	429353.76 (402308.70,455770.26)
9	Southeast Asia	2008	444813.56 (417701.99,470962.44)
9	Southeast Asia	2009	459170.71 (429000.63,488064.49)
9	Southeast Asia	2010	471412.00 (441518.48,500541.17)
9	Southeast Asia	2011	477063.29 (443968.15,508634.60)

9	Southeast Asia	2012	491551.05 (453680.50,528826.38)
9	Southeast Asia	2013	506845.88 (470017.57,543388.92)
9	Southeast Asia	2014	518290.08 (475743.15,559567.64)
9	Southeast Asia	2015	538297.26 (494956.81,582961.47)
9	Southeast Asia	2016	556149.38 (510720.07,604372.55)
9	Southeast Asia	2017	565755.71 (520736.37,614355.21)
9	Southeast Asia	2018	583952.08 (534020.98,633701.24)
9	Southeast Asia	2019	604050.99 (548220.58,655137.83)
9	Southeast Asia	2020	615434.88 (551932.71,672789.47)
9	Southeast Asia	2021	638703.89 (575906.56,694079.45)
21	Oceania	1990	4528.19 (3745.94,5489.13)
21	Oceania	1991	4683.31 (3901.70,5654.39)
21	Oceania	1992	4805.59 (4014.86,5787.20)
21	Oceania	1993	4935.03 (4135.49,5880.75)
21	Oceania	1994	5051.72 (4249.36,6022.82)
21	Oceania	1995	5178.55 (4393.93,6191.34)
21	Oceania	1996	5352.24 (4564.89,6336.69)
21	Oceania	1997	5490.99 (4717.51,6444.83)
21	Oceania	1998	5607.60 (4826.64,6583.87)
21	Oceania	1999	5856.40 (5048.05,6826.45)
21	Oceania	2000	6083.98 (5291.64,7067.61)
21	Oceania	2001	6220.58 (5426.99,7168.42)
21	Oceania	2002	6385.69 (5594.82,7315.43)
21	Oceania	2003	6563.75 (5766.53,7543.52)
21	Oceania	2004	6683.80 (5896.68,7652.13)
21	Oceania	2005	6949.01 (6106.36,7943.12)
21	Oceania	2006	7234.03 (6333.98,8280.99)
21	Oceania	2007	7504.44 (6554.33,8630.93)
21	Oceania	2008	7737.80 (6757.93,8895.73)
21	Oceania	2009	7910.55 (6953.74,9117.88)
21	Oceania	2010	8216.56 (7159.89,9508.81)
21	Oceania	2011	8505.46 (7387.03,9842.89)
21	Oceania	2012	8744.78 (7525.25,10170.10)
21	Oceania	2013	8943.45 (7727.28,10430.58)
21	Oceania	2014	9212.50 (7919.66,10815.74)
21	Oceania	2015	9524.84 (8104.50,11197.58)
21	Oceania	2016	9702.38 (8230.81,11386.81)
21	Oceania	2017	10000.05 (8444.23,11807.13)
21	Oceania	2018	10297.72 (8648.33,12175.90)
21	Oceania	2019	10689.31 (8920.67,12639.51)
21	Oceania	2020	10936.82 (9163.35,12974.45)
21	Oceania	2021	11136.86 (9328.75,13321.11)
32	Central Asia	1990	131771.21 (124692.12,136248.92)
32	Central Asia	1991	140915.40 (133489.79,145399.00)
32	Central Asia	1992	151761.70 (143328.70,156399.15)
32	Central Asia	1993	163288.28 (154899.08,168151.52)
32	Central Asia	1994	169130.40 (161032.64,174191.16)
32	Central Asia	1995	173568.22 (165539.57,179006.13)
32	Central Asia	1996	175074.85 (166457.41,180657.48)
32	Central Asia	1997	171805.76 (163088.19,177639.76)

32	Central Asia	1998	171753.14 (162162.30,177713.88)
32	Central Asia	1999	170503.26 (160258.77,175982.31)
32	Central Asia	2000	172955.65 (163784.56,178483.63)
32	Central Asia	2001	173760.31 (164840.04,179496.99)
32	Central Asia	2002	176826.19 (168079.41,182417.77)
32	Central Asia	2003	179183.41 (170308.42,184463.71)
32	Central Asia	2004	176741.36 (168574.13,182194.08)
32	Central Asia	2005	180674.89 (172807.77,187104.34)
32	Central Asia	2006	183144.73 (174735.11,191141.20)
32	Central Asia	2007	184681.96 (175249.18,192490.33)
32	Central Asia	2008	184399.92 (175639.15,191665.25)
32	Central Asia	2009	177054.16 (168597.75,183149.69)
32	Central Asia	2010	175302.62 (167101.85,181300.19)
32	Central Asia	2011	176630.99 (168035.22,182946.02)
32	Central Asia	2012	179376.17 (170814.50,185665.91)
32	Central Asia	2013	179144.37 (170449.05,186179.17)
32	Central Asia	2014	180016.22 (170998.18,186921.21)
32	Central Asia	2015	181246.25 (172068.98,187271.76)
32	Central Asia	2016	181110.68 (171742.03,187718.59)
32	Central Asia	2017	179963.77 (170515.65,186941.79)
32	Central Asia	2018	177283.21 (167851.01,183871.75)
32	Central Asia	2019	174765.14 (164742.87,182545.89)
32	Central Asia	2020	176143.86 (162880.66,187871.32)
32	Central Asia	2021	175394.03 (159202.72,192406.55)
42	Central Europe	1990	364089.93 (349914.69,371867.90)
42	Central Europe	1991	371257.61 (357163.22,379410.28)
42	Central Europe	1992	373811.75 (359286.17,382639.51)
42	Central Europe	1993	373841.22 (358649.01,383373.58)
42	Central Europe	1994	375190.18 (358359.75,384401.88)
42	Central Europe	1995	382168.46 (365656.92,390574.85)
42	Central Europe	1996	381022.90 (365539.44,389291.76)
42	Central Europe	1997	382204.50 (366727.50,390574.47)
42	Central Europe	1998	367152.93 (350800.78,375454.01)
42	Central Europe	1999	359608.42 (343357.43,367627.82)
42	Central Europe	2000	345752.46 (330161.37,354112.21)
42	Central Europe	2001	340966.35 (325153.31,349305.39)
42	Central Europe	2002	338749.97 (322362.60,347127.22)
42	Central Europe	2003	339014.50 (322223.09,347562.54)
42	Central Europe	2004	334026.47 (318145.92,342941.52)
42	Central Europe	2005	336997.57 (319687.16,346133.44)
42	Central Europe	2006	333973.17 (316356.65,343253.51)
42	Central Europe	2007	333708.75 (315301.94,343082.56)
42	Central Europe	2008	333206.91 (313742.24,342897.43)
42	Central Europe	2009	335316.15 (316058.95,344919.08)
42	Central Europe	2010	333061.86 (311984.96,343185.63)
42	Central Europe	2011	330074.94 (308079.78,340969.55)
42	Central Europe	2012	330811.78 (307593.58,341336.07)
42	Central Europe	2013	324334.84 (300952.29,335143.19)
42	Central Europe	2014	325507.50 (301468.21,336705.97)
42	Central Europe	2015	332961.08 (307978.23,344716.55)

42	Central Europe	2016	328377.62 (302878.87,340240.80)
42	Central Europe	2017	334512.13 (307820.21,346318.18)
42	Central Europe	2018	335840.07 (309127.25,348279.57)
42	Central Europe	2019	332633.41 (306266.07,345156.57)
42	Central Europe	2020	334634.59 (307723.42,352156.79)
42	Central Europe	2021	331281.29 (299877.58,352596.61)
56	Eastern Europe	1990	786270.58 (751128.31,803303.11)
56	Eastern Europe	1991	808969.69 (772515.98,826772.12)
56	Eastern Europe	1992	864160.98 (827310.37,881473.09)
56	Eastern Europe	1993	1002451.07 (966458.54,1019554.67)
56	Eastern Europe	1994	1080253.27 (1044709.76,1099968.86)
56	Eastern Europe	1995	1059705.62 (1023689.68,1077885.21)
56	Eastern Europe	1996	1016262.05 (978154.11,1034313.14)
56	Eastern Europe	1997	982665.70 (943631.77,1000905.31)
56	Eastern Europe	1998	970667.85 (930468.95,990020.73)
56	Eastern Europe	1999	1041659.15 (1001539.35,1059751.64)
56	Eastern Europe	2000	1084466.68 (1044329.37,1102623.71)
56	Eastern Europe	2001	1104758.58 (1064337.01,1123348.94)
56	Eastern Europe	2002	1144946.77 (1105178.61,1163330.53)
56	Eastern Europe	2003	1161654.05 (1120174.29,1180404.51)
56	Eastern Europe	2004	1142460.25 (1101539.87,1161656.08)
56	Eastern Europe	2005	1179351.98 (1137124.27,1199494.74)
56	Eastern Europe	2006	1120171.03 (1075378.94,1140383.92)
56	Eastern Europe	2007	1107024.38 (1061982.37,1127829.02)
56	Eastern Europe	2008	1121123.92 (1074433.78,1142041.57)
56	Eastern Europe	2009	1078316.82 (1029141.64,1100199.11)
56	Eastern Europe	2010	1092244.67 (1039918.57,1114719.03)
56	Eastern Europe	2011	1049903.53 (996007.56,1073626.44)
56	Eastern Europe	2012	1035288.64 (979060.05,1058995.14)
56	Eastern Europe	2013	1016687.60 (959985.25,1041421.20)
56	Eastern Europe	2014	1000693.83 (944433.38,1025297.76)
56	Eastern Europe	2015	977379.97 (919360.48,1001938.17)
56	Eastern Europe	2016	964157.59 (907362.38,988889.10)
56	Eastern Europe	2017	930959.02 (874366.93,967185.61)
56	Eastern Europe	2018	933570.45 (867968.21,981859.62)
56	Eastern Europe	2019	924936.62 (857296.30,978574.03)
56	Eastern Europe	2020	901513.53 (836768.43,951338.87)
56	Eastern Europe	2021	903624.61 (811062.83,990580.97)
65	High-income Asia Pacific	1990	117621.58 (107063.63,122952.52)
65	High-income Asia Pacific	1991	118995.06 (107660.73,124460.45)
65	High-income Asia Pacific	1992	121018.68 (109224.01,126786.14)
65	High-income Asia Pacific	1993	122112.55 (109844.45,128025.21)
65	High-income Asia Pacific	1994	119069.31 (106798.22,124974.99)
65	High-income Asia Pacific	1995	116734.51 (104905.81,122516.15)
65	High-income Asia Pacific	1996	114535.48 (102013.89,120543.97)
65	High-income Asia Pacific	1997	114422.48 (101823.93,120599.83)
65	High-income Asia Pacific	1998	116968.53 (103521.49,123489.48)
65	High-income Asia Pacific	1999	119529.10 (105782.57,126313.01)
65	High-income Asia Pacific	2000	117130.58 (103206.41,124064.95)
65	High-income Asia Pacific	2001	116925.24 (102655.26,124342.57)

65	High-income Asia Pacific	2002	118522.04 (103532.73,126384.69)
65	High-income Asia Pacific	2003	121569.79 (106075.09,129889.96)
65	High-income Asia Pacific	2004	123182.69 (107047.00,131825.81)
65	High-income Asia Pacific	2005	127985.93 (111140.41,137159.08)
65	High-income Asia Pacific	2006	129393.33 (112012.88,139188.48)
65	High-income Asia Pacific	2007	130629.78 (112364.95,140604.54)
65	High-income Asia Pacific	2008	132560.30 (113729.67,142940.02)
65	High-income Asia Pacific	2009	133638.29 (114156.78,144457.85)
65	High-income Asia Pacific	2010	136085.78 (116140.63,147407.11)
65	High-income Asia Pacific	2011	139261.98 (118493.02,150842.94)
65	High-income Asia Pacific	2012	140312.71 (119205.21,152327.57)
65	High-income Asia Pacific	2013	139804.66 (118158.58,152392.65)
65	High-income Asia Pacific	2014	139479.03 (117439.37,152020.34)
65	High-income Asia Pacific	2015	138995.81 (116355.87,152035.89)
65	High-income Asia Pacific	2016	140664.32 (117043.05,154136.18)
65	High-income Asia Pacific	2017	142630.90 (117818.59,156658.32)
65	High-income Asia Pacific	2018	145606.28 (119525.26,159778.52)
65	High-income Asia Pacific	2019	146664.07 (120273.68,161210.59)
65	High-income Asia Pacific	2020	145721.10 (118444.64,160826.20)
65	High-income Asia Pacific	2021	152702.31 (123996.01,168788.53)
70	Australasia	1990	40160.88 (37288.99,41659.24)
70	Australasia	1991	39280.68 (36292.84,40798.91)
70	Australasia	1992	39663.22 (36616.46,41234.34)
70	Australasia	1993	39125.21 (35910.41,40732.51)
70	Australasia	1994	39192.57 (35888.72,40873.07)
70	Australasia	1995	38842.23 (35492.22,40529.11)
70	Australasia	1996	38590.81 (35163.08,40334.36)
70	Australasia	1997	37826.49 (34441.62,39578.20)
70	Australasia	1998	37187.37 (33648.90,38989.19)
70	Australasia	1999	36456.45 (32977.62,38340.32)
70	Australasia	2000	35291.29 (31760.99,37149.10)
70	Australasia	2001	34784.28 (31104.94,36698.51)
70	Australasia	2002	34384.78 (30552.98,36241.80)
70	Australasia	2003	33675.80 (29916.16,35616.16)
70	Australasia	2004	32836.04 (29184.65,34709.83)
70	Australasia	2005	31953.28 (28281.46,33861.17)
70	Australasia	2006	31437.34 (27699.33,33378.11)
70	Australasia	2007	31567.78 (27902.79,33455.66)
70	Australasia	2008	31697.27 (28028.36,33612.31)
70	Australasia	2009	31036.19 (27311.66,32971.48)
70	Australasia	2010	30424.91 (26672.15,32357.19)
70	Australasia	2011	29900.55 (26194.57,31851.25)
70	Australasia	2012	28832.73 (25235.38,30752.28)
70	Australasia	2013	28392.11 (24796.85,30330.06)
70	Australasia	2014	28611.36 (24888.88,30577.61)
70	Australasia	2015	28480.22 (24721.78,30421.00)
70	Australasia	2016	27760.98 (23992.33,29683.52)
70	Australasia	2017	27369.34 (23570.95,29303.78)
70	Australasia	2018	26815.42 (22969.02,28780.91)
70	Australasia	2019	27837.59 (23813.88,29875.49)

70	Australasia	2020	26812.33 (22612.47,28986.79)
70	Australasia	2021	28640.38 (24369.92,30972.53)
73	Western Europe	1990	879289.87 (811263.83,910871.04)
73	Western Europe	1991	874875.95 (804418.79,906965.46)
73	Western Europe	1992	863207.37 (791240.27,896032.94)
73	Western Europe	1993	860857.46 (789014.09,894139.35)
73	Western Europe	1994	843329.75 (770343.98,877255.90)
73	Western Europe	1995	835421.88 (761363.25,870051.31)
73	Western Europe	1996	825105.90 (750786.59,859650.79)
73	Western Europe	1997	810770.35 (735463.16,845988.63)
73	Western Europe	1998	807009.67 (730787.29,843544.36)
73	Western Europe	1999	793557.74 (717039.09,830980.76)
73	Western Europe	2000	761428.58 (685797.78,798784.98)
73	Western Europe	2001	743962.03 (667449.82,782222.53)
73	Western Europe	2002	739299.02 (663200.88,777388.38)
73	Western Europe	2003	729957.95 (653406.46,768516.65)
73	Western Europe	2004	694543.62 (618137.34,732756.47)
73	Western Europe	2005	674743.33 (598848.51,712476.54)
73	Western Europe	2006	655935.05 (579786.42,692963.57)
73	Western Europe	2007	647902.06 (571380.11,685697.68)
73	Western Europe	2008	633367.74 (557670.58,671022.26)
73	Western Europe	2009	622101.60 (546157.99,659590.01)
73	Western Europe	2010	606355.90 (531145.46,644376.94)
73	Western Europe	2011	595624.70 (519732.65,633366.75)
73	Western Europe	2012	593733.51 (517717.85,632067.16)
73	Western Europe	2013	585672.98 (510047.73,623394.88)
73	Western Europe	2014	571273.51 (495781.59,609630.15)
73	Western Europe	2015	578169.30 (502439.59,616192.19)
73	Western Europe	2016	569696.60 (494361.91,607829.82)
73	Western Europe	2017	563293.56 (487692.32,601303.26)
73	Western Europe	2018	557386.82 (481158.21,595867.26)
73	Western Europe	2019	548719.93 (471176.20,588187.77)
73	Western Europe	2020	550534.03 (471029.31,590752.02)
73	Western Europe	2021	543038.41 (463990.18,584339.40)
96	Southern Latin America	1990	62788.28 (59932.98,64743.19)
96	Southern Latin America	1991	60385.60 (57430.28,62312.99)
96	Southern Latin America	1992	60353.11 (57187.97,62203.59)
96	Southern Latin America	1993	59716.12 (56580.00,61628.06)
96	Southern Latin America	1994	57470.58 (54147.40,59507.01)
96	Southern Latin America	1995	57872.77 (54508.37,59775.17)
96	Southern Latin America	1996	57144.63 (53716.27,59108.72)
96	Southern Latin America	1997	55708.36 (52205.45,57767.36)
96	Southern Latin America	1998	56762.45 (53336.71,58735.27)
96	Southern Latin America	1999	57280.66 (54078.54,59175.95)
96	Southern Latin America	2000	53850.93 (50586.65,55757.32)
96	Southern Latin America	2001	53201.40 (49961.85,55114.63)
96	Southern Latin America	2002	53387.38 (50180.85,55194.04)
96	Southern Latin America	2003	53152.74 (49865.28,55027.28)
96	Southern Latin America	2004	51577.79 (48266.54,53516.37)
96	Southern Latin America	2005	51017.43 (47544.10,52935.00)

96	Southern Latin America	2006	51194.98 (47586.99,53184.33)
96	Southern Latin America	2007	52659.29 (48901.91,54762.57)
96	Southern Latin America	2008	51017.40 (47151.10,53141.45)
96	Southern Latin America	2009	51398.79 (47536.78,53594.60)
96	Southern Latin America	2010	52714.80 (48887.66,54996.20)
96	Southern Latin America	2011	53151.71 (49167.74,55489.59)
96	Southern Latin America	2012	53249.47 (49267.82,55506.25)
96	Southern Latin America	2013	52749.73 (48766.79,54995.34)
96	Southern Latin America	2014	51648.42 (47787.72,53826.21)
96	Southern Latin America	2015	52628.25 (48958.24,54852.61)
96	Southern Latin America	2016	54196.51 (50534.14,56401.05)
96	Southern Latin America	2017	53014.56 (49491.48,55237.53)
96	Southern Latin America	2018	52899.77 (49361.24,55123.69)
96	Southern Latin America	2019	52840.47 (48907.15,55074.67)
96	Southern Latin America	2020	51121.34 (47180.47,53443.66)
96	Southern Latin America	2021	49098.50 (45022.28,51734.53)
100	High-income North America	1990	644903.98 (580448.05,675667.31)
100	High-income North America	1991	639715.33 (573967.17,670631.58)
100	High-income North America	1992	636105.91 (569953.75,667342.08)
100	High-income North America	1993	648869.26 (581073.39,680498.06)
100	High-income North America	1994	648859.76 (581288.64,680255.69)
100	High-income North America	1995	649856.63 (581941.69,681915.08)
100	High-income North America	1996	641477.13 (572900.89,673178.92)
100	High-income North America	1997	632492.06 (564011.69,664280.00)
100	High-income North America	1998	631286.09 (561811.10,663440.26)
100	High-income North America	1999	643426.80 (573085.66,676424.62)
100	High-income North America	2000	630920.79 (560156.96,663923.62)
100	High-income North America	2001	621057.29 (550683.77,654249.86)
100	High-income North America	2002	612567.50 (543091.22,646127.77)
100	High-income North America	2003	597331.11 (528351.09,630494.14)
100	High-income North America	2004	574124.12 (506249.26,607144.84)
100	High-income North America	2005	565798.03 (498279.69,598586.36)
100	High-income North America	2006	549795.76 (482929.43,582801.33)
100	High-income North America	2007	533107.03 (466921.99,565056.37)
100	High-income North America	2008	527867.29 (461839.29,560425.68)
100	High-income North America	2009	516101.60 (450670.62,548005.02)
100	High-income North America	2010	507018.86 (442304.07,539078.54)
100	High-income North America	2011	506915.00 (442342.04,539555.79)
100	High-income North America	2012	506372.68 (442043.19,539119.94)
100	High-income North America	2013	507877.44 (443484.24,541163.67)
100	High-income North America	2014	508989.11 (444440.39,542257.08)
100	High-income North America	2015	511705.04 (446920.52,545144.15)
100	High-income North America	2016	517227.23 (452489.64,551035.54)
100	High-income North America	2017	520661.61 (455363.42,554214.73)
100	High-income North America	2018	519578.01 (453047.65,553189.48)
100	High-income North America	2019	518460.93 (452028.22,552548.91)
100	High-income North America	2020	520975.52 (451103.35,555569.28)
100	High-income North America	2021	534770.38 (468399.90,571529.32)
104	Caribbean	1990	44603.13 (42479.94,46171.14)
104	Caribbean	1991	44062.27 (41725.86,45889.35)

104	Caribbean	1992	45015.76 (42736.25,47014.42)
104	Caribbean	1993	46338.86 (43989.40,48331.37)
104	Caribbean	1994	46538.30 (44217.15,48568.68)
104	Caribbean	1995	46625.22 (44058.12,48714.23)
104	Caribbean	1996	46869.48 (44490.53,48980.76)
104	Caribbean	1997	46175.23 (43681.69,48254.78)
104	Caribbean	1998	46452.99 (43903.24,48628.95)
104	Caribbean	1999	46273.01 (43732.91,48551.72)
104	Caribbean	2000	45025.60 (42270.57,47394.86)
104	Caribbean	2001	45398.28 (42423.39,47855.84)
104	Caribbean	2002	44517.32 (41885.70,47098.99)
104	Caribbean	2003	46314.44 (43358.13,48949.49)
104	Caribbean	2004	47931.74 (44674.81,50807.67)
104	Caribbean	2005	49297.80 (45977.88,52377.13)
104	Caribbean	2006	48458.81 (45000.01,51886.74)
104	Caribbean	2007	48720.52 (44676.47,52974.81)
104	Caribbean	2008	49446.21 (45398.81,53779.18)
104	Caribbean	2009	50186.16 (46170.27,54473.72)
104	Caribbean	2010	50036.55 (46232.95,54157.91)
104	Caribbean	2011	49465.23 (45581.24,53201.31)
104	Caribbean	2012	50243.16 (46429.06,53836.50)
104	Caribbean	2013	51984.84 (48023.23,55616.93)
104	Caribbean	2014	53188.86 (49198.21,56989.23)
104	Caribbean	2015	54933.76 (50642.16,59187.57)
104	Caribbean	2016	55910.72 (51843.31,60097.71)
104	Caribbean	2017	57737.52 (53510.62,62265.04)
104	Caribbean	2018	58527.63 (53970.75,63634.80)
104	Caribbean	2019	59949.81 (54769.99,65391.54)
104	Caribbean	2020	61570.01 (56071.02,67655.60)
104	Caribbean	2021	61449.99 (54942.17,69041.03)
120	Andean Latin America	1990	17089.42 (15548.22,18721.24)
120	Andean Latin America	1991	16965.63 (15401.27,18625.41)
120	Andean Latin America	1992	17797.31 (16306.83,19438.40)
120	Andean Latin America	1993	18126.43 (16561.15,19782.81)
120	Andean Latin America	1994	18424.43 (16828.30,20075.32)
120	Andean Latin America	1995	18106.73 (16575.82,19906.85)
120	Andean Latin America	1996	17508.75 (16007.52,19053.51)
120	Andean Latin America	1997	17959.92 (16520.87,19659.88)
120	Andean Latin America	1998	18501.56 (17106.81,20228.81)
120	Andean Latin America	1999	17758.05 (16273.62,19392.99)
120	Andean Latin America	2000	17764.90 (16152.04,19391.19)
120	Andean Latin America	2001	17752.12 (16045.29,19699.79)
120	Andean Latin America	2002	18469.63 (16736.52,20429.37)
120	Andean Latin America	2003	18765.50 (16801.92,20762.08)
120	Andean Latin America	2004	18831.55 (17060.74,20791.23)
120	Andean Latin America	2005	19348.18 (17624.37,21190.89)
120	Andean Latin America	2006	19492.61 (17882.22,21569.96)
120	Andean Latin America	2007	19590.46 (17798.84,21526.55)
120	Andean Latin America	2008	19922.29 (17915.94,22113.59)
120	Andean Latin America	2009	20564.43 (18494.54,23073.57)

120	Andean Latin America	2010	20901.04 (18575.77,23541.09)
120	Andean Latin America	2011	21091.93 (18746.11,23684.67)
120	Andean Latin America	2012	21920.54 (19402.38,24543.12)
120	Andean Latin America	2013	22782.86 (20379.88,25550.24)
120	Andean Latin America	2014	23991.35 (21528.33,26829.32)
120	Andean Latin America	2015	25225.23 (22723.51,28083.07)
120	Andean Latin America	2016	27474.89 (24624.16,30243.09)
120	Andean Latin America	2017	30247.72 (26857.29,33913.88)
120	Andean Latin America	2018	32498.21 (28727.14,36700.24)
120	Andean Latin America	2019	34954.54 (30558.09,40000.85)
120	Andean Latin America	2020	33575.57 (29161.44,39110.60)
120	Andean Latin America	2021	32964.66 (28040.24,39366.22)
124	Central Latin America	1990	88861.76 (84857.22,90852.22)
124	Central Latin America	1991	92642.63 (88562.32,94644.94)
124	Central Latin America	1992	96004.89 (91722.32,98061.16)
124	Central Latin America	1993	99242.41 (94851.27,101434.06)
124	Central Latin America	1994	102720.86 (98174.37,105030.50)
124	Central Latin America	1995	105594.44 (100338.27,108541.75)
124	Central Latin America	1996	108139.57 (103094.95,110730.07)
124	Central Latin America	1997	108358.52 (103095.90,111026.02)
124	Central Latin America	1998	109161.72 (103766.71,111935.12)
124	Central Latin America	1999	110012.88 (104495.40,112791.83)
124	Central Latin America	2000	111279.59 (105526.49,114302.90)
124	Central Latin America	2001	113999.85 (107980.05,117056.78)
124	Central Latin America	2002	117472.71 (111256.95,120610.79)
124	Central Latin America	2003	122574.81 (115980.60,125940.92)
124	Central Latin America	2004	124160.00 (117125.50,127832.16)
124	Central Latin America	2005	128020.77 (120790.77,131767.93)
124	Central Latin America	2006	131777.31 (124257.24,135629.40)
124	Central Latin America	2007	134173.75 (125968.17,138209.05)
124	Central Latin America	2008	142599.15 (134403.84,146987.98)
124	Central Latin America	2009	151032.07 (142514.55,155461.15)
124	Central Latin America	2010	157112.71 (147758.02,161988.68)
124	Central Latin America	2011	161174.20 (151178.83,166618.26)
124	Central Latin America	2012	166990.02 (156315.20,172375.28)
124	Central Latin America	2013	177207.84 (165839.92,183048.33)
124	Central Latin America	2014	185008.42 (173135.45,190940.14)
124	Central Latin America	2015	193601.20 (180833.53,200123.95)
124	Central Latin America	2016	208057.35 (194609.58,215128.31)
124	Central Latin America	2017	213564.61 (199465.00,220839.07)
124	Central Latin America	2018	224577.89 (208997.07,233540.03)
124	Central Latin America	2019	233413.38 (216987.04,244617.03)
124	Central Latin America	2020	238372.03 (214812.26,257969.97)
124	Central Latin America	2021	247050.82 (221193.82,273150.30)
134	Tropical Latin America	1990	107545.38 (102011.55,110638.54)
134	Tropical Latin America	1991	105281.58 (99494.72,108575.50)
134	Tropical Latin America	1992	106486.94 (100914.36,109562.84)
134	Tropical Latin America	1993	110464.61 (104557.83,113666.37)
134	Tropical Latin America	1994	111002.00 (105251.56,114151.43)
134	Tropical Latin America	1995	112496.28 (106304.29,115783.24)

134	Tropical Latin America	1996	114655.61 (108157.05,118025.99)
134	Tropical Latin America	1997	114166.28 (107746.11,117734.69)
134	Tropical Latin America	1998	116392.55 (110128.34,119973.72)
134	Tropical Latin America	1999	117244.88 (110766.21,121069.02)
134	Tropical Latin America	2000	117460.21 (110796.69,121261.73)
134	Tropical Latin America	2001	118712.18 (111690.61,122709.52)
134	Tropical Latin America	2002	120731.07 (113499.43,124720.68)
134	Tropical Latin America	2003	123301.82 (115999.25,127345.11)
134	Tropical Latin America	2004	126164.04 (118114.28,130643.52)
134	Tropical Latin America	2005	124041.45 (115718.30,128550.08)
134	Tropical Latin America	2006	127178.71 (118529.44,131818.27)
134	Tropical Latin America	2007	129472.28 (120643.61,134169.06)
134	Tropical Latin America	2008	132113.37 (122919.55,136870.43)
134	Tropical Latin America	2009	134788.51 (125252.68,139821.37)
134	Tropical Latin America	2010	138128.97 (128782.29,143330.71)
134	Tropical Latin America	2011	141829.31 (131713.96,147366.92)
134	Tropical Latin America	2012	142648.36 (132035.01,148151.23)
134	Tropical Latin America	2013	145028.60 (134561.26,150798.84)
134	Tropical Latin America	2014	146911.74 (135766.56,152739.78)
134	Tropical Latin America	2015	150478.47 (138923.02,156315.85)
134	Tropical Latin America	2016	156688.72 (144898.89,162765.71)
134	Tropical Latin America	2017	155022.69 (143203.18,161172.56)
134	Tropical Latin America	2018	155933.31 (143606.15,162389.86)
134	Tropical Latin America	2019	159349.74 (147284.83,165921.98)
134	Tropical Latin America	2020	162131.75 (148445.39,168915.72)
134	Tropical Latin America	2021	162299.20 (149052.81,170324.77)
138	North Africa and Middle East	1990	386036.53 (357949.00,418208.39)
138	North Africa and Middle East	1991	392866.52 (365350.07,425708.62)
138	North Africa and Middle East	1992	400856.74 (373870.42,433997.95)
138	North Africa and Middle East	1993	412628.12 (384078.25,446340.33)
138	North Africa and Middle East	1994	423535.38 (394050.15,456931.49)
138	North Africa and Middle East	1995	431589.30 (400888.80,465519.49)
138	North Africa and Middle East	1996	439939.09 (408765.06,474651.41)
138	North Africa and Middle East	1997	450369.13 (419319.50,485008.88)
138	North Africa and Middle East	1998	458215.66 (426302.89,494215.99)
138	North Africa and Middle East	1999	466203.65 (433319.68,502376.60)
138	North Africa and Middle East	2000	469524.17 (437284.94,506142.55)
138	North Africa and Middle East	2001	483104.17 (452182.48,519139.01)
138	North Africa and Middle East	2002	493927.58 (462555.52,527089.13)
138	North Africa and Middle East	2003	505419.56 (472324.22,541537.64)
138	North Africa and Middle East	2004	513387.65 (478308.23,553814.93)
138	North Africa and Middle East	2005	521011.13 (486023.99,561247.64)
138	North Africa and Middle East	2006	532118.16 (497083.25,575576.11)
138	North Africa and Middle East	2007	542700.92 (504480.43,589009.96)
138	North Africa and Middle East	2008	559136.19 (519668.92,605448.78)
138	North Africa and Middle East	2009	577670.75 (537502.51,622832.67)
138	North Africa and Middle East	2010	588770.37 (547082.13,633687.64)
138	North Africa and Middle East	2011	597920.96 (554615.09,644682.93)
138	North Africa and Middle East	2012	615047.23 (569376.42,662945.30)
138	North Africa and Middle East	2013	632916.97 (585422.80,681975.64)

138	North Africa and Middle East	2014	653881.72 (603610.88,703422.37)
138	North Africa and Middle East	2015	680874.55 (629004.10,732228.06)
138	North Africa and Middle East	2016	690075.70 (633779.26,742191.55)
138	North Africa and Middle East	2017	700112.95 (642009.22,753337.90)
138	North Africa and Middle East	2018	718798.13 (658456.48,776455.51)
138	North Africa and Middle East	2019	744055.08 (681758.91,804129.67)
138	North Africa and Middle East	2020	759732.50 (682027.83,832674.77)
138	North Africa and Middle East	2021	769135.14 (685360.01,858253.16)
159	South Asia	1990	708220.98 (643038.47,771051.83)
159	South Asia	1991	725562.38 (662385.51,787263.28)
159	South Asia	1992	752362.90 (688824.83,811475.90)
159	South Asia	1993	770101.05 (700543.59,841712.65)
159	South Asia	1994	797705.49 (734582.68,858784.34)
159	South Asia	1995	834648.82 (773392.14,897278.85)
159	South Asia	1996	859908.42 (792387.71,929195.98)
159	South Asia	1997	889455.79 (820240.64,954129.10)
159	South Asia	1998	917613.44 (848500.82,985726.72)
159	South Asia	1999	914246.50 (846205.64,983763.56)
159	South Asia	2000	934064.89 (861412.68,1003969.55)
159	South Asia	2001	967198.87 (901001.21,1032338.10)
159	South Asia	2002	998590.91 (932267.24,1071106.14)
159	South Asia	2003	1034172.30 (962276.19,1103647.33)
159	South Asia	2004	1050674.32 (980053.75,1128656.33)
159	South Asia	2005	1080789.43 (1011203.68,1155214.48)
159	South Asia	2006	1139502.84 (1062132.53,1212238.75)
159	South Asia	2007	1210661.33 (1136314.39,1279886.99)
159	South Asia	2008	1264564.76 (1182347.54,1341713.26)
159	South Asia	2009	1296678.47 (1220776.05,1372615.56)
159	South Asia	2010	1358350.73 (1277051.62,1439982.29)
159	South Asia	2011	1436990.87 (1351605.55,1526443.40)
159	South Asia	2012	1522795.44 (1431490.75,1611551.46)
159	South Asia	2013	1601511.98 (1507566.79,1693676.82)
159	South Asia	2014	1689595.49 (1594180.73,1789028.81)
159	South Asia	2015	1690262.59 (1585172.30,1794182.92)
159	South Asia	2016	1749863.69 (1635741.16,1863795.11)
159	South Asia	2017	1778101.10 (1663310.74,1875456.43)
159	South Asia	2018	1858674.31 (1744175.24,1968734.84)
159	South Asia	2019	1921169.50 (1795878.25,2055073.08)
159	South Asia	2020	1959451.78 (1819170.97,2118161.16)
159	South Asia	2021	1990113.47 (1824490.29,2155696.11)
167	Central Sub-Saharan Africa	1990	23271.65 (18250.79,29475.76)
167	Central Sub-Saharan Africa	1991	23893.22 (18876.97,30206.96)
167	Central Sub-Saharan Africa	1992	24420.30 (19441.45,30858.72)
167	Central Sub-Saharan Africa	1993	24954.64 (19773.91,31374.45)
167	Central Sub-Saharan Africa	1994	25767.68 (20385.55,32489.79)
167	Central Sub-Saharan Africa	1995	26616.55 (21037.66,33622.77)
167	Central Sub-Saharan Africa	1996	27655.19 (21819.65,35134.48)
167	Central Sub-Saharan Africa	1997	27584.40 (21915.80,34654.75)
167	Central Sub-Saharan Africa	1998	28162.38 (22570.92,35238.34)
167	Central Sub-Saharan Africa	1999	28611.82 (23078.99,35553.22)

167	Central Sub-Saharan Africa	2000	29115.99 (23433.35,36065.62)
167	Central Sub-Saharan Africa	2001	29377.36 (23627.56,36289.41)
167	Central Sub-Saharan Africa	2002	29388.25 (23778.85,36488.19)
167	Central Sub-Saharan Africa	2003	30127.84 (24487.66,37620.87)
167	Central Sub-Saharan Africa	2004	30484.21 (24859.04,38186.68)
167	Central Sub-Saharan Africa	2005	30651.86 (24922.94,38673.50)
167	Central Sub-Saharan Africa	2006	31361.28 (25492.70,39263.33)
167	Central Sub-Saharan Africa	2007	32101.88 (26051.73,40063.59)
167	Central Sub-Saharan Africa	2008	33373.68 (27009.93,41500.93)
167	Central Sub-Saharan Africa	2009	34005.44 (27180.45,42246.15)
167	Central Sub-Saharan Africa	2010	34710.93 (27711.49,43553.03)
167	Central Sub-Saharan Africa	2011	35696.77 (28592.35,44832.42)
167	Central Sub-Saharan Africa	2012	36670.52 (29295.55,45942.67)
167	Central Sub-Saharan Africa	2013	37340.99 (29824.12,46864.77)
167	Central Sub-Saharan Africa	2014	38132.71 (30409.06,47494.79)
167	Central Sub-Saharan Africa	2015	39908.19 (31779.79,50462.41)
167	Central Sub-Saharan Africa	2016	41099.60 (33012.10,51775.43)
167	Central Sub-Saharan Africa	2017	42407.91 (34210.18,53262.68)
167	Central Sub-Saharan Africa	2018	44424.86 (35397.74,55866.66)
167	Central Sub-Saharan Africa	2019	46396.83 (36701.68,58663.40)
167	Central Sub-Saharan Africa	2020	48355.85 (37870.01,61558.70)
167	Central Sub-Saharan Africa	2021	49849.97 (38888.58,63650.14)
174	Eastern Sub-Saharan Africa	1990	43989.28 (39100.72,50522.16)
174	Eastern Sub-Saharan Africa	1991	45593.42 (40457.49,51921.10)
174	Eastern Sub-Saharan Africa	1992	47303.07 (42365.47,53712.88)
174	Eastern Sub-Saharan Africa	1993	49201.04 (44062.82,55461.04)
174	Eastern Sub-Saharan Africa	1994	50594.63 (45062.67,57215.20)
174	Eastern Sub-Saharan Africa	1995	52087.39 (46727.45,58913.80)
174	Eastern Sub-Saharan Africa	1996	53497.92 (47948.45,60353.88)
174	Eastern Sub-Saharan Africa	1997	55107.95 (49304.22,62106.72)
174	Eastern Sub-Saharan Africa	1998	56754.02 (50946.85,63777.05)
174	Eastern Sub-Saharan Africa	1999	57743.98 (52009.00,65021.01)
174	Eastern Sub-Saharan Africa	2000	58832.62 (52920.15,65703.57)
174	Eastern Sub-Saharan Africa	2001	59388.28 (53597.01,65884.80)
174	Eastern Sub-Saharan Africa	2002	60416.68 (54715.46,66682.15)
174	Eastern Sub-Saharan Africa	2003	61640.54 (55657.94,68394.31)
174	Eastern Sub-Saharan Africa	2004	63201.07 (57640.94,69553.41)
174	Eastern Sub-Saharan Africa	2005	64403.07 (58682.60,70673.25)
174	Eastern Sub-Saharan Africa	2006	65808.19 (59532.60,71652.42)
174	Eastern Sub-Saharan Africa	2007	67089.53 (61180.65,73155.15)
174	Eastern Sub-Saharan Africa	2008	68732.76 (62559.55,75052.21)
174	Eastern Sub-Saharan Africa	2009	70556.56 (63922.79,77472.46)
174	Eastern Sub-Saharan Africa	2010	72544.92 (65523.84,80194.91)
174	Eastern Sub-Saharan Africa	2011	74810.83 (67080.65,82864.36)
174	Eastern Sub-Saharan Africa	2012	76725.13 (68544.73,85892.82)
174	Eastern Sub-Saharan Africa	2013	78697.37 (70421.23,88348.22)
174	Eastern Sub-Saharan Africa	2014	80817.59 (71630.22,90738.03)
174	Eastern Sub-Saharan Africa	2015	84150.14 (74053.73,95097.55)
174	Eastern Sub-Saharan Africa	2016	87214.32 (76373.38,98117.50)
174	Eastern Sub-Saharan Africa	2017	89753.06 (78191.43,101977.88)

174	Eastern Sub-Saharan Africa	2018	92913.51 (80858.47,106151.24)
174	Eastern Sub-Saharan Africa	2019	96068.18 (83591.31,110188.10)
174	Eastern Sub-Saharan Africa	2020	99106.73 (86124.49,113941.79)
174	Eastern Sub-Saharan Africa	2021	101222.41 (87734.69,117265.40)
192	Southern Sub-Saharan Africa	1990	17901.16 (15561.88,19842.89)
192	Southern Sub-Saharan Africa	1991	18481.03 (16003.36,20543.11)
192	Southern Sub-Saharan Africa	1992	19777.34 (17304.52,21830.09)
192	Southern Sub-Saharan Africa	1993	19690.53 (17025.44,21908.12)
192	Southern Sub-Saharan Africa	1994	21186.29 (18602.96,23304.48)
192	Southern Sub-Saharan Africa	1995	22090.37 (19586.12,24254.06)
192	Southern Sub-Saharan Africa	1996	24067.12 (21978.22,26108.40)
192	Southern Sub-Saharan Africa	1997	27450.64 (25768.32,29259.08)
192	Southern Sub-Saharan Africa	1998	28767.85 (26995.21,30419.23)
192	Southern Sub-Saharan Africa	1999	28873.52 (27142.31,30597.98)
192	Southern Sub-Saharan Africa	2000	30565.70 (28768.18,32050.18)
192	Southern Sub-Saharan Africa	2001	31109.79 (29332.96,32769.62)
192	Southern Sub-Saharan Africa	2002	32508.39 (30616.69,34420.94)
192	Southern Sub-Saharan Africa	2003	33930.41 (31913.40,36252.31)
192	Southern Sub-Saharan Africa	2004	34338.04 (32361.40,36686.95)
192	Southern Sub-Saharan Africa	2005	34962.08 (32989.61,37433.80)
192	Southern Sub-Saharan Africa	2006	35952.86 (33832.38,38643.90)
192	Southern Sub-Saharan Africa	2007	36273.15 (34224.48,38898.75)
192	Southern Sub-Saharan Africa	2008	36847.62 (34757.74,39495.43)
192	Southern Sub-Saharan Africa	2009	37353.94 (35226.64,39911.67)
192	Southern Sub-Saharan Africa	2010	37408.69 (35330.58,39971.61)
192	Southern Sub-Saharan Africa	2011	37180.83 (35203.59,39214.43)
192	Southern Sub-Saharan Africa	2012	36928.57 (34886.25,38941.59)
192	Southern Sub-Saharan Africa	2013	36747.97 (34741.48,38618.98)
192	Southern Sub-Saharan Africa	2014	37426.96 (35405.45,39404.63)
192	Southern Sub-Saharan Africa	2015	38540.46 (36405.74,40576.40)
192	Southern Sub-Saharan Africa	2016	39024.38 (36779.64,41129.99)
192	Southern Sub-Saharan Africa	2017	38569.41 (36167.55,40881.84)
192	Southern Sub-Saharan Africa	2018	38886.69 (36453.92,41302.70)
192	Southern Sub-Saharan Africa	2019	38752.11 (36056.54,41424.79)
192	Southern Sub-Saharan Africa	2020	39929.43 (37087.28,42691.87)
192	Southern Sub-Saharan Africa	2021	39816.58 (36778.00,43187.11)
199	Western Sub-Saharan Africa	1990	75117.12 (64540.84,87128.79)
199	Western Sub-Saharan Africa	1991	75931.47 (65847.61,87620.44)
199	Western Sub-Saharan Africa	1992	77368.73 (66846.47,89033.54)
199	Western Sub-Saharan Africa	1993	78925.15 (68604.45,89947.75)
199	Western Sub-Saharan Africa	1994	80799.44 (70215.65,92807.04)
199	Western Sub-Saharan Africa	1995	83181.24 (71321.86,95945.54)
199	Western Sub-Saharan Africa	1996	86923.78 (75285.77,98910.88)
199	Western Sub-Saharan Africa	1997	90947.31 (78391.26,104581.05)
199	Western Sub-Saharan Africa	1998	95042.02 (81431.35,108446.52)
199	Western Sub-Saharan Africa	1999	99439.05 (84929.03,114081.38)
199	Western Sub-Saharan Africa	2000	103821.84 (89234.47,120535.65)
199	Western Sub-Saharan Africa	2001	106376.72 (91241.08,122553.71)
199	Western Sub-Saharan Africa	2002	108169.45 (92149.45,124430.17)
199	Western Sub-Saharan Africa	2003	108151.86 (92155.74,123534.04)

199	Western Sub-Saharan Africa	2004	108432.63 (93227.47,124449.48)
199	Western Sub-Saharan Africa	2005	108575.98 (93822.69,124337.72)
199	Western Sub-Saharan Africa	2006	109640.59 (94324.98,126173.28)
199	Western Sub-Saharan Africa	2007	110490.40 (96333.21,127097.04)
199	Western Sub-Saharan Africa	2008	113651.57 (98146.81,130613.42)
199	Western Sub-Saharan Africa	2009	117367.08 (101669.47,133651.45)
199	Western Sub-Saharan Africa	2010	120767.55 (105315.00,138203.34)
199	Western Sub-Saharan Africa	2011	124937.06 (110056.43,141715.49)
199	Western Sub-Saharan Africa	2012	129332.15 (112328.85,147797.13)
199	Western Sub-Saharan Africa	2013	129843.75 (114356.11,147536.80)
199	Western Sub-Saharan Africa	2014	133271.80 (115644.10,153089.39)
199	Western Sub-Saharan Africa	2015	144078.03 (123686.04,164116.95)
199	Western Sub-Saharan Africa	2016	145032.78 (124433.22,165208.96)
199	Western Sub-Saharan Africa	2017	147769.31 (127965.16,169513.83)
199	Western Sub-Saharan Africa	2018	151623.25 (129082.87,173063.21)
199	Western Sub-Saharan Africa	2019	155758.73 (133101.29,178432.39)
199	Western Sub-Saharan Africa	2020	158824.20 (135952.43,184764.19)
199	Western Sub-Saharan Africa	2021	161332.72 (140025.90,185440.45)

ase Mortality: Total 90-2021.
**ASR_UI**
158.90 (148.14,165.30)
156.77 (146.58,163.35)
156.08 (146.14,162.66)
158.19 (147.82,164.48)
157.50 (147.81,163.74)
155.26 (145.45,160.96)
151.67 (142.38,157.19)
148.11 (138.87,153.91)
145.74 (136.01,151.32)
144.80 (135.28,150.15)
142.54 (133.37,148.15)
140.96 (131.58,146.10)
140.49 (131.67,145.52)
140.12 (131.12,145.13)
136.92 (127.67,141.93)
135.24 (126.52,140.05)
130.91 (122.32,135.92)
128.90 (120.31,134.08)
128.12 (119.16,133.20)
125.93 (117.62,130.75)
125.00 (116.02,129.74)
123.33 (114.56,128.56)
121.82 (113.22,126.95)
120.89 (112.03,126.41)
119.56 (110.92,125.31)
117.28 (108.40,122.56)
115.65 (106.75,121.20)
113.29 (104.37,119.24)
112.24 (103.57,118.12)
110.92 (102.77,116.60)
109.47 (100.66,115.89)
108.73 (99.60,115.38)
157.59 (144.19,163.92)
153.50 (140.29,159.88)
148.74 (135.78,154.94)
146.17 (133.22,152.15)
141.20 (128.80,147.02)
136.92 (124.91,142.64)
131.64 (119.73,137.24)
126.75 (115.13,132.31)
123.02 (111.65,128.54)
120.33 (109.14,125.77)
114.23 (103.37,119.54)
110.04 (99.29,115.32)
106.52 (96.34,111.72)
102.69 (92.68,107.72)

97.04 (87.34,101.88)
93.35 (84.08,98.01)
89.21 (80.13,93.80)
85.56 (76.77,89.94)
82.58 (73.80,86.81)
79.17 (71.00,83.31)
75.80 (67.74,79.82)
73.44 (65.84,77.40)
71.27 (63.55,75.10)
69.44 (62.18,73.29)
67.15 (59.95,70.88)
65.87 (59.06,69.46)
64.48 (57.84,68.01)
63.06 (56.34,66.61)
61.60 (54.78,65.08)
59.90 (53.30,63.32)
58.59 (51.96,61.91)
58.45 (52.18,61.92)
193.94 (182.11,200.60)
192.75 (180.96,199.84)
196.14 (184.63,202.94)
208.12 (195.55,214.62)
212.35 (200.78,218.42)
208.14 (196.66,214.77)
200.98 (188.93,207.11)
194.34 (182.13,200.54)
190.17 (178.49,196.31)
193.67 (182.78,199.97)
193.68 (182.81,200.20)
192.03 (180.81,198.40)
193.96 (182.82,200.15)
195.78 (183.72,202.05)
190.99 (179.22,197.37)
190.47 (179.06,196.49)
179.94 (168.83,186.18)
175.82 (164.29,182.33)
174.08 (162.16,180.51)
169.05 (158.62,175.45)
167.62 (155.64,173.88)
162.05 (150.71,169.02)
157.40 (145.30,165.01)
153.29 (141.39,160.73)
149.36 (138.35,157.45)
145.31 (134.18,152.82)
141.26 (128.79,148.58)
137.01 (124.91,145.72)
134.64 (121.60,143.61)
132.05 (120.36,140.64)
129.24 (117.08,138.37)
127.50 (115.03,137.72)

119.50 (106.26,132.45)
119.29 (106.71,131.22)
119.26 (107.11,130.70)
119.54 (107.01,131.23)
121.79 (109.84,134.21)
122.59 (110.24,134.60)
121.37 (108.67,132.41)
121.32 (108.76,132.31)
121.69 (109.57,132.40)
119.98 (108.41,131.45)
119.59 (107.31,130.72)
118.87 (107.33,129.36)
118.64 (107.41,128.93)
118.72 (106.64,128.30)
116.62 (105.49,125.83)
114.16 (103.83,123.17)
114.42 (103.35,123.80)
115.04 (103.92,123.90)
115.61 (104.57,124.28)
115.21 (104.39,124.76)
115.59 (104.95,125.11)
117.31 (106.07,127.50)
120.94 (109.00,131.52)
124.72 (112.76,135.04)
127.52 (115.24,139.05)
126.45 (113.75,137.91)
125.29 (113.46,136.29)
121.35 (110.91,131.61)
119.15 (108.16,129.37)
118.66 (108.86,129.02)
118.07 (107.52,129.50)
116.41 (105.21,127.69)
140.99 (129.76,151.24)
139.61 (128.30,149.80)
139.86 (128.85,150.01)
139.72 (129.68,149.62)
140.01 (130.04,149.17)
141.39 (131.81,150.20)
141.45 (132.44,150.81)
141.82 (131.99,150.51)
142.27 (132.54,150.89)
138.86 (129.79,147.45)
137.01 (127.53,145.27)
137.65 (129.00,145.57)
138.15 (129.25,146.31)
139.24 (130.03,146.85)
137.84 (129.43,145.72)
137.02 (128.03,144.81)
139.12 (130.11,147.34)
141.32 (132.66,148.90)

142.56 (133.44,150.32)
141.69 (133.52,149.10)
142.28 (133.67,149.55)
142.98 (134.18,150.68)
144.71 (135.74,152.70)
147.68 (138.71,155.15)
151.94 (142.22,159.80)
148.05 (137.55,155.98)
146.63 (137.17,154.51)
144.33 (135.72,152.28)
145.39 (136.16,153.54)
144.88 (135.63,152.96)
143.22 (133.67,152.79)
142.10 (131.30,151.87)
127.06 (118.35,134.97)
126.28 (118.39,133.81)
126.94 (118.64,134.59)
127.03 (118.18,134.49)
126.44 (118.70,133.35)
126.77 (118.70,133.74)
126.43 (118.93,132.69)
124.72 (117.22,131.22)
124.09 (116.07,129.85)
122.65 (114.67,128.88)
123.11 (115.42,128.83)
124.03 (116.32,129.83)
125.08 (117.60,130.74)
127.23 (119.26,132.53)
128.32 (120.34,133.91)
127.90 (120.44,133.32)
124.61 (116.25,129.92)
123.91 (116.01,129.93)
125.43 (116.74,131.63)
126.34 (117.79,132.23)
127.71 (118.82,133.88)
127.95 (119.09,134.56)
126.88 (117.99,134.27)
127.99 (117.91,135.21)
127.11 (118.08,134.70)
124.94 (115.05,132.04)
124.01 (112.77,132.12)
121.85 (112.12,130.27)
120.83 (110.85,128.37)
120.13 (109.79,129.28)
119.15 (108.78,128.20)
118.71 (107.23,127.80)
93.92 (83.87,105.30)
93.51 (84.08,103.85)
93.11 (82.88,103.80)
93.13 (82.74,103.26)

92.77 (84.23,101.38)
92.86 (84.17,101.27)
92.86 (84.74,101.76)
92.84 (84.56,100.61)
94.37 (84.79,102.07)
96.56 (87.44,105.29)
101.52 (92.68,109.82)
105.86 (96.23,113.98)
110.24 (101.08,118.14)
119.11 (109.69,128.01)
124.48 (113.58,133.05)
125.60 (115.17,134.27)
118.60 (107.77,126.97)
117.56 (107.50,127.28)
119.96 (108.74,128.97)
122.82 (112.04,131.64)
125.70 (113.42,135.90)
126.86 (114.80,137.82)
123.19 (110.64,135.49)
123.86 (109.72,136.00)
121.43 (107.98,133.44)
117.31 (102.86,129.46)
115.32 (99.58,129.04)
113.92 (99.67,129.53)
112.17 (97.41,128.89)
110.76 (95.34,125.87)
109.91 (93.33,126.74)
108.90 (91.18,125.79)
114.72 (103.39,125.65)
114.17 (103.29,125.44)
114.94 (104.57,125.72)
115.38 (106.03,125.06)
115.97 (105.88,125.71)
116.47 (107.38,126.06)
116.67 (107.26,125.61)
115.96 (107.52,124.69)
116.33 (107.98,125.17)
117.25 (108.98,125.41)
116.95 (108.85,125.02)
116.66 (108.41,124.78)
117.53 (108.69,125.01)
117.77 (109.64,125.49)
118.20 (109.97,126.36)
119.22 (111.15,126.67)
119.40 (110.73,126.87)
118.79 (110.18,126.55)
119.39 (111.56,126.71)
119.44 (111.00,127.15)
118.89 (110.65,126.47)
116.38 (108.14,124.15)

115.88 (106.99,124.26)
115.54 (106.55,124.04)
114.05 (104.56,123.69)
114.27 (105.05,123.37)
113.99 (103.79,123.02)
111.96 (102.44,120.87)
111.68 (101.82,120.56)
111.66 (101.59,120.98)
109.93 (98.67,120.34)
110.92 (100.18,120.20)
182.55 (155.47,217.42)
184.12 (156.53,218.27)
183.83 (157.01,218.31)
183.45 (156.98,215.85)
182.56 (156.37,212.75)
182.47 (157.47,213.52)
183.49 (159.30,212.41)
182.86 (159.58,210.55)
181.78 (159.21,209.76)
183.70 (161.32,211.08)
184.97 (162.85,211.73)
183.90 (162.02,209.95)
183.08 (161.93,208.08)
182.54 (161.85,207.58)
180.06 (159.46,204.72)
180.60 (159.84,204.43)
182.37 (161.06,206.41)
182.69 (160.76,207.66)
182.28 (160.10,207.21)
180.79 (159.23,206.27)
181.28 (158.63,206.83)
181.79 (158.91,207.75)
180.58 (157.08,207.80)
179.15 (155.82,206.56)
178.54 (155.42,207.36)
178.05 (153.17,207.28)
176.34 (151.36,204.90)
175.70 (150.76,204.68)
174.96 (149.55,203.46)
174.57 (147.79,204.12)
172.09 (145.04,201.94)
170.89 (145.43,201.15)
320.47 (299.83,332.36)
336.35 (315.46,347.80)
358.21 (335.87,369.78)
380.91 (358.61,393.11)
391.34 (369.45,403.20)
398.89 (376.33,412.43)
400.04 (377.01,413.40)
390.45 (367.32,404.44)

386.99 (361.93,401.42)
380.25 (353.76,393.07)
380.06 (356.06,392.98)
375.63 (353.13,389.29)
377.83 (355.62,390.93)
377.96 (355.71,390.40)
367.57 (347.18,379.59)
369.00 (349.01,382.82)
366.54 (347.04,382.78)
363.97 (342.37,379.35)
358.09 (338.02,372.56)
338.67 (319.19,351.19)
329.47 (311.27,341.31)
325.67 (306.88,338.49)
325.61 (307.11,337.87)
320.12 (301.83,333.19)
317.07 (297.47,330.09)
313.58 (294.94,325.16)
306.54 (287.32,318.59)
297.20 (278.59,309.40)
284.48 (265.75,295.97)
273.55 (256.60,286.39)
271.22 (250.52,288.90)
265.51 (240.67,290.42)
272.56 (259.22,279.18)
274.63 (261.53,281.53)
273.81 (260.76,281.12)
271.67 (258.29,279.23)
269.07 (255.06,276.45)
268.98 (255.58,275.71)
264.68 (252.03,271.28)
262.78 (250.40,269.40)
250.77 (237.97,257.31)
242.47 (229.72,248.84)
228.62 (216.35,234.81)
221.64 (209.50,227.98)
216.95 (204.64,223.01)
213.77 (201.31,219.87)
206.02 (194.10,212.11)
202.36 (190.44,208.37)
195.96 (183.91,202.07)
191.57 (179.71,197.65)
187.15 (174.78,193.17)
183.63 (171.58,189.53)
177.09 (164.99,182.82)
170.75 (158.28,176.69)
166.96 (154.60,172.56)
159.83 (147.52,165.36)
156.54 (144.30,162.13)
156.35 (144.30,161.99)

150.74 (139.02,156.22)
150.26 (138.37,155.59)
147.96 (136.46,153.40)
143.76 (132.69,149.13)
142.10 (130.87,149.54)
139.98 (126.84,148.91)
323.17 (305.26,331.76)
325.86 (308.47,334.33)
343.29 (325.84,351.54)
393.03 (375.54,401.09)
419.08 (401.86,428.12)
411.07 (393.85,419.44)
393.29 (375.45,401.57)
379.21 (360.95,387.63)
373.50 (354.48,382.33)
397.38 (378.89,405.74)
411.22 (392.71,419.66)
414.81 (396.00,423.37)
427.20 (408.74,435.62)
430.66 (411.44,439.47)
415.44 (396.86,424.11)
420.06 (401.72,428.79)
393.45 (374.56,402.03)
383.03 (364.17,391.71)
383.14 (364.18,391.92)
362.76 (343.71,371.42)
361.57 (341.99,370.15)
341.12 (321.69,349.95)
329.61 (310.10,338.15)
318.93 (299.96,327.49)
309.43 (291.23,317.77)
297.62 (279.36,305.69)
287.76 (270.37,295.58)
271.78 (254.76,282.65)
268.60 (249.43,282.52)
262.68 (243.24,278.00)
253.72 (235.55,267.70)
252.89 (226.96,277.15)
67.04 (59.85,70.50)
64.90 (57.71,68.35)
63.04 (56.06,66.47)
60.84 (54.04,64.14)
56.75 (50.38,59.89)
53.09 (47.31,56.01)
49.88 (44.12,52.70)
47.64 (42.17,50.40)
46.68 (41.16,49.43)
45.82 (40.46,48.50)
43.03 (37.85,45.62)
41.18 (36.17,43.80)

39.98 (35.04,42.60)
39.35 (34.49,41.96)
38.33 (33.51,40.92)
38.18 (33.46,40.76)
37.08 (32.48,39.70)
35.92 (31.32,38.45)
35.05 (30.55,37.55)
34.08 (29.65,36.52)
33.36 (29.07,35.75)
32.91 (28.66,35.27)
31.90 (27.79,34.27)
30.69 (26.68,33.04)
29.60 (25.67,31.85)
28.44 (24.58,30.64)
27.80 (23.99,29.93)
27.13 (23.27,29.25)
26.78 (22.88,28.84)
26.19 (22.43,28.21)
25.31 (21.52,27.36)
25.56 (21.78,27.65)
176.76 (162.82,184.02)
167.24 (153.34,174.35)
163.99 (150.32,171.05)
157.04 (143.26,163.93)
152.77 (139.08,159.65)
146.88 (133.52,153.55)
141.06 (128.00,147.71)
133.88 (121.44,140.25)
127.38 (114.95,133.74)
120.75 (109.05,127.06)
112.93 (101.57,118.84)
107.33 (96.07,113.14)
102.60 (91.34,108.11)
97.38 (86.78,102.87)
92.04 (82.12,97.24)
86.56 (76.97,91.62)
82.13 (72.77,86.99)
79.91 (71.09,84.52)
77.97 (69.38,82.39)
74.24 (65.85,78.62)
70.55 (62.40,74.79)
67.01 (59.16,71.12)
62.61 (55.24,66.58)
59.84 (52.73,63.68)
58.49 (51.46,62.21)
56.49 (49.61,60.10)
53.25 (46.59,56.68)
50.90 (44.38,54.26)
48.35 (41.97,51.62)
48.71 (42.25,52.02)

45.41 (38.90,48.82)
46.67 (40.23,50.21)
148.22 (136.54,153.85)
144.86 (133.11,150.37)
140.47 (128.83,145.95)
137.66 (126.35,143.04)
132.29 (121.09,137.57)
128.40 (117.34,133.63)
124.45 (113.61,129.58)
120.21 (109.49,125.30)
117.50 (106.88,122.66)
113.18 (102.75,118.34)
106.33 (96.28,111.34)
101.72 (91.81,106.73)
99.18 (89.54,104.04)
96.18 (86.67,100.97)
89.54 (80.25,94.18)
84.92 (75.96,89.37)
80.61 (71.88,84.84)
77.81 (69.28,82.00)
74.29 (66.08,78.37)
71.09 (63.11,75.01)
67.52 (59.91,71.37)
64.64 (57.22,68.34)
62.81 (55.61,66.46)
60.55 (53.60,64.08)
57.62 (50.89,61.10)
56.97 (50.39,60.32)
54.93 (48.57,58.16)
53.21 (46.99,56.38)
51.62 (45.46,54.73)
49.67 (43.67,52.74)
48.54 (42.53,51.67)
47.27 (41.45,50.42)
149.43 (141.12,154.54)
139.71 (131.60,144.55)
135.56 (127.15,140.04)
130.17 (122.01,134.69)
121.40 (113.65,125.86)
118.70 (111.16,123.01)
113.71 (106.28,117.84)
107.59 (100.48,111.73)
106.86 (100.11,110.68)
105.26 (99.06,108.87)
96.58 (90.54,100.08)
93.16 (87.36,96.51)
91.33 (85.64,94.47)
88.94 (83.27,92.08)
84.41 (78.99,87.56)
81.50 (75.98,84.51)

79.77 (74.21,82.86)
80.05 (74.35,83.20)
75.63 (70.00,78.78)
74.20 (68.74,77.32)
74.12 (68.90,77.22)
72.94 (67.67,76.07)
71.40 (66.28,74.35)
69.13 (64.13,71.96)
66.18 (61.42,68.90)
65.97 (61.58,68.66)
66.57 (62.27,69.18)
63.87 (59.75,66.46)
62.42 (58.36,64.99)
60.94 (56.59,63.44)
57.72 (53.44,60.27)
54.41 (50.08,57.26)
177.72 (160.27,186.07)
172.32 (155.08,180.50)
167.64 (150.79,175.63)
167.30 (150.51,175.19)
164.13 (147.82,171.76)
160.96 (145.02,168.49)
155.71 (140.00,163.03)
150.50 (135.16,157.65)
147.14 (131.94,154.21)
147.38 (132.30,154.50)
141.82 (126.95,148.79)
137.13 (122.62,144.02)
132.84 (118.79,139.63)
127.07 (113.38,133.64)
119.83 (106.64,126.25)
115.46 (102.66,121.72)
109.81 (97.44,115.90)
104.03 (92.09,109.79)
100.52 (88.91,106.20)
96.08 (84.86,101.53)
91.91 (81.15,97.22)
89.69 (79.23,94.90)
87.44 (77.31,92.59)
85.58 (75.69,90.64)
83.93 (74.23,88.92)
82.38 (72.89,87.23)
81.48 (72.22,86.26)
80.12 (70.98,84.73)
78.02 (68.91,82.53)
76.13 (67.23,80.57)
74.87 (65.72,79.33)
75.85 (67.17,80.60)
188.97 (179.08,195.79)
179.98 (169.97,187.22)

178.04 (168.08,185.71)
177.73 (167.95,185.63)
173.09 (163.91,180.78)
168.10 (158.24,175.75)
163.81 (154.99,171.18)
156.44 (147.66,163.40)
152.61 (143.99,159.80)
147.46 (139.15,154.69)
139.26 (130.65,146.42)
136.35 (127.49,143.64)
129.91 (122.14,137.42)
131.54 (123.17,139.03)
132.85 (123.76,140.71)
133.45 (124.49,141.73)
127.92 (118.87,136.93)
125.29 (114.93,136.14)
123.96 (113.83,134.76)
122.70 (112.84,133.06)
119.51 (110.44,129.30)
115.20 (106.22,123.88)
113.88 (105.35,122.01)
114.69 (105.91,122.69)
114.25 (105.71,122.38)
115.06 (106.29,123.96)
114.15 (106.21,122.74)
115.06 (106.81,124.13)
113.86 (105.43,123.78)
113.78 (104.19,124.14)
114.18 (103.92,125.53)
112.50 (100.51,126.54)
92.95 (84.56,101.24)
89.16 (81.13,97.39)
90.56 (83.50,98.67)
89.16 (81.12,97.33)
87.33 (79.66,95.31)
83.20 (76.10,91.52)
77.85 (71.19,84.53)
77.19 (70.86,84.15)
76.74 (70.80,83.66)
71.01 (65.03,77.62)
68.32 (62.18,74.44)
65.62 (59.32,72.61)
65.66 (59.41,72.54)
64.03 (57.20,70.72)
61.68 (55.99,67.98)
60.78 (55.24,66.63)
58.69 (53.81,64.73)
56.48 (51.19,61.95)
54.89 (49.30,60.80)
54.23 (48.78,60.79)

53.00 (47.07,59.75)
51.42 (45.76,57.77)
51.40 (45.44,57.59)
51.31 (45.94,57.57)
51.88 (46.56,57.98)
52.40 (47.12,58.31)
54.97 (49.21,60.53)
58.37 (51.83,65.48)
60.50 (53.47,68.37)
62.91 (54.98,72.00)
59.31 (51.48,69.10)
58.17 (49.56,69.33)
125.72 (118.69,129.14)
125.52 (118.57,128.89)
124.51 (117.58,127.79)
123.25 (116.67,126.56)
122.64 (116.20,125.97)
121.70 (114.69,125.60)
120.53 (113.91,123.89)
116.07 (109.50,119.37)
112.09 (105.52,115.35)
108.52 (102.24,111.74)
105.16 (98.94,108.42)
103.22 (97.04,106.34)
102.02 (95.88,105.09)
102.00 (95.87,105.12)
99.07 (92.82,102.30)
97.88 (91.72,101.02)
96.43 (90.31,99.53)
94.07 (87.84,97.15)
95.85 (89.91,99.01)
97.56 (91.54,100.60)
97.73 (91.47,100.98)
96.40 (89.99,99.80)
95.96 (89.45,99.22)
97.94 (91.27,101.31)
98.48 (91.80,101.77)
99.27 (92.39,102.73)
102.68 (95.71,106.26)
101.53 (94.51,105.08)
102.82 (95.37,106.95)
102.99 (95.58,107.90)
102.14 (91.99,110.44)
103.69 (92.48,114.59)
135.91 (126.39,140.82)
127.76 (118.49,132.77)
124.14 (115.51,128.64)
123.92 (115.08,128.40)
119.72 (111.38,123.90)
116.44 (108.16,120.65)

114.02 (105.90,118.07)
109.01 (101.39,113.16)
106.59 (99.35,110.53)
103.15 (96.08,107.12)
99.22 (92.34,102.99)
96.50 (89.55,100.26)
94.57 (87.59,98.22)
93.04 (86.28,96.55)
91.67 (84.74,95.33)
86.81 (79.93,90.35)
85.66 (78.93,89.14)
83.92 (77.46,87.30)
82.39 (75.85,85.65)
81.05 (74.56,84.34)
80.16 (74.07,83.45)
79.44 (73.13,82.79)
77.10 (70.81,80.29)
75.62 (69.62,78.86)
73.96 (67.88,77.08)
73.21 (67.16,76.25)
73.61 (67.67,76.64)
70.35 (64.66,73.30)
68.30 (62.61,71.27)
67.39 (61.99,70.29)
66.37 (60.54,69.28)
64.49 (58.98,67.84)
275.18 (253.62,299.12)
270.84 (249.57,294.28)
267.68 (247.21,290.37)
267.08 (246.85,289.84)
265.95 (246.45,286.76)
263.39 (243.24,283.69)
260.50 (240.92,280.99)
258.49 (238.87,278.75)
255.25 (235.70,274.93)
251.50 (232.20,270.94)
245.74 (226.55,265.53)
244.92 (226.46,264.40)
242.53 (223.86,260.21)
240.31 (222.00,258.45)
235.73 (217.29,253.59)
230.97 (213.10,249.24)
227.89 (209.76,246.87)
224.80 (206.35,243.75)
223.85 (205.84,242.47)
223.39 (205.27,241.12)
219.78 (201.39,237.25)
215.64 (197.07,233.27)
214.17 (196.17,230.89)
213.25 (195.63,230.18)

213.53 (195.65,230.12)
215.50 (197.31,231.86)
211.93 (192.75,228.04)
208.32 (189.21,224.58)
206.46 (187.63,223.76)
206.53 (186.75,223.48)
205.22 (184.81,223.30)
202.85 (180.59,223.68)
136.39 (122.87,149.55)
135.94 (123.38,148.14)
137.74 (124.87,149.55)
137.10 (123.21,149.64)
138.28 (126.44,149.98)
142.30 (130.41,153.86)
142.35 (129.93,154.61)
142.07 (130.03,152.86)
141.96 (129.95,153.82)
136.02 (125.02,147.47)
134.58 (123.76,145.32)
135.36 (124.97,144.86)
135.34 (125.39,145.67)
136.52 (126.34,145.95)
135.00 (124.73,145.31)
134.38 (125.08,144.14)
137.77 (127.35,147.01)
142.22 (132.94,150.94)
143.60 (133.14,152.21)
141.72 (132.31,150.41)
143.28 (133.82,152.65)
145.93 (136.01,155.89)
149.57 (139.66,158.90)
156.17 (146.47,165.62)
163.51 (153.05,173.31)
156.72 (146.08,166.86)
155.15 (144.10,165.63)
151.24 (140.56,160.29)
151.81 (142.22,161.20)
151.64 (141.09,162.42)
150.34 (138.81,162.50)
149.14 (136.97,161.16)
134.90 (107.86,167.20)
134.16 (107.83,166.78)
133.12 (106.79,165.26)
132.20 (106.35,164.58)
132.59 (106.74,165.34)
133.09 (106.34,166.20)
134.52 (107.99,168.03)
131.14 (105.81,163.06)
130.39 (105.28,161.30)
129.08 (105.33,157.82)

128.22 (104.01,156.15)
126.24 (102.20,154.57)
123.46 (100.35,151.89)
123.24 (100.44,153.10)
121.75 (99.27,152.05)
119.62 (97.84,148.78)
119.12 (97.63,147.27)
118.63 (97.02,146.59)
119.86 (97.41,148.85)
118.68 (96.02,147.90)
117.73 (94.74,145.80)
117.29 (94.27,146.13)
116.66 (93.30,144.67)
115.08 (91.97,142.86)
113.75 (90.88,141.26)
115.49 (91.77,143.77)
114.83 (92.54,141.93)
114.44 (92.59,141.37)
115.82 (93.14,143.63)
116.94 (93.52,146.75)
118.53 (93.91,148.47)
119.34 (93.70,150.29)
69.44 (61.05,78.85)
70.31 (61.93,79.80)
71.37 (63.29,81.42)
72.57 (64.71,81.78)
73.28 (65.29,82.39)
74.23 (66.93,83.54)
74.75 (66.89,83.74)
75.37 (67.43,84.30)
76.04 (68.00,84.17)
75.98 (68.61,84.83)
76.05 (68.47,84.43)
75.38 (67.72,83.80)
75.09 (67.59,83.18)
74.79 (67.51,82.51)
74.84 (67.70,82.80)
74.51 (67.29,81.87)
74.21 (66.72,81.63)
73.61 (66.49,81.26)
73.40 (66.09,81.29)
73.25 (65.71,81.50)
73.21 (65.88,81.66)
73.17 (65.12,81.50)
72.65 (64.73,81.96)
72.12 (64.30,81.55)
71.58 (62.99,81.21)
72.15 (63.42,82.28)
72.18 (62.17,81.87)
71.84 (62.28,82.16)

71.94 (62.45,82.46)
71.95 (62.13,82.87)
72.26 (62.60,83.30)
72.16 (62.09,82.99)
75.96 (64.95,84.84)
75.89 (64.88,85.11)
78.88 (68.31,87.77)
76.81 (65.66,85.85)
80.38 (70.15,88.63)
81.47 (71.94,89.78)
86.79 (78.37,94.56)
96.30 (89.41,103.18)
98.91 (92.23,104.98)
97.59 (90.87,103.83)
100.84 (93.68,106.05)
100.64 (94.32,106.24)
102.77 (95.93,108.95)
104.81 (97.70,111.91)
103.75 (97.10,110.49)
103.42 (96.87,110.72)
104.20 (97.72,111.44)
103.49 (96.96,110.53)
103.04 (96.66,109.87)
102.52 (96.38,109.71)
100.95 (94.64,107.79)
98.39 (92.21,104.24)
95.64 (89.54,101.21)
93.06 (87.14,98.30)
92.48 (86.90,97.48)
92.85 (86.93,97.77)
91.54 (85.37,96.40)
88.33 (82.02,93.46)
86.64 (80.50,91.74)
83.97 (77.94,89.66)
84.69 (78.10,90.20)
83.44 (76.93,90.19)
105.29 (90.24,121.56)
103.98 (89.87,119.49)
103.50 (90.35,117.94)
103.13 (90.31,116.52)
103.13 (89.60,117.33)
103.69 (89.96,118.21)
105.93 (92.87,119.74)
108.37 (94.61,122.77)
110.71 (95.38,125.01)
113.22 (98.40,128.77)
115.72 (99.55,132.68)
115.87 (99.91,131.62)
115.57 (100.39,132.06)
112.98 (98.27,127.12)

110.82 (97.02,125.03)
108.60 (95.52,122.37)
107.54 (94.56,121.43)
105.77 (93.53,119.47)
106.44 (93.27,120.83)
107.11 (93.97,120.82)
107.34 (94.65,121.02)
108.22 (95.80,121.55)
108.87 (95.64,123.32)
106.26 (93.75,119.38)
105.79 (92.67,120.35)
111.13 (96.87,124.96)
108.58 (95.48,122.06)
107.40 (94.17,121.64)
106.97 (92.20,120.30)
106.56 (92.93,120.17)
105.96 (92.15,121.37)
105.97 (92.83,120.17)

Table S5. Trends in Age-Standardized Mortality Rate (ASMR) for Ischemic Heart Disease Ac and Net Drift Analysis, 1990-2021		
**location_id**	**location_name**	**ASR_StartYear_UI**
6	People's Republic of China	94.14 (84.01,105.89)
7	Democratic People's Republic of Korea	112.65 (86.11,137.19)
8	Taiwan (Province of China)	73.72 (68.60,76.86)
10	Kingdom of Cambodia	115.73 (97.89,135.07)
11	Republic of Indonesia	102.96 (85.94,119.91)
12	Lao People's Democratic Republic	235.34 (187.60,286.47)
13	Malaysia	177.45 (162.83,189.78)
14	Republic of Maldives	167.99 (147.79,188.24)
15	Republic of the Union of Myanmar	193.33 (157.01,238.03)
16	Republic of the Philippines	174.14 (159.02,186.45)
17	Democratic Socialist Republic of Sri Lanka	138.44 (124.98,151.47)
18	Kingdom of Thailand	84.42 (71.21,97.77)
19	Democratic Republic of Timor-Leste	122.80 (98.09,150.93)
20	Socialist Republic of Viet Nam	66.40 (52.90,83.93)
22	Republic of Fiji	291.09 (256.25,329.57)
23	Republic of Kiribati	189.64 (154.84,225.35)
24	Republic of the Marshall Islands	263.68 (230.16,301.81)
25	Federated States of Micronesia	268.02 (220.53,324.28)
26	Independent State of Papua New Guinea	136.52 (103.02,179.07)
27	Independent State of Samoa	205.73 (170.84,240.01)
28	Solomon Islands	292.82 (239.31,353.68)
29	Kingdom of Tonga	138.69 (116.31,161.28)
30	Republic of Vanuatu	312.35 (259.57,376.09)
33	Republic of Armenia	307.44 (286.83,323.83)
34	Republic of Azerbaijan	362.31 (337.11,388.22)
35	Georgia	358.65 (337.55,374.96)
36	Republic of Kazakhstan	298.65 (276.16,319.98)
37	Kyrgyz Republic	264.38 (242.39,284.65)
38	Mongolia	324.79 (286.63,365.71)
39	Republic of Tajikistan	304.83 (266.01,336.17)
40	Turkmenistan	407.01 (383.66,423.46)
41	Republic of Uzbekistan	318.62 (295.07,333.63)
43	Republic of Albania	174.23 (153.51,192.16)
44	Bosnia and Herzegovina	200.09 (181.52,217.01)
45	Republic of Bulgaria	403.98 (387.80,416.73)
46	Republic of Croatia	287.39 (271.39,300.65)
47	Czech Republic	296.03 (282.63,305.49)
48	Hungary	262.80 (250.24,271.33)
49	North Macedonia	208.57 (187.07,232.69)
50	Montenegro	164.64 (142.76,183.41)
51	Republic of Poland	269.75 (255.72,277.00)
52	Romania	278.03 (264.18,287.37)
53	Republic of Serbia	309.12 (280.44,334.36)
54	Slovak Republic	327.25 (310.38,341.42)
55	Republic of Slovenia	128.30 (119.03,135.28)
57	Republic of Belarus	331.13 (309.29,343.52)

58	Republic of Estonia	368.95 (347.37,383.27)
59	Republic of Latvia	343.06 (322.50,356.66)
60	Republic of Lithuania	365.29 (343.23,379.01)
61	Republic of Moldova	404.17 (382.51,417.02)
62	Russian Federation	311.27 (294.21,318.67)
63	Ukraine	338.06 (318.09,349.50)
66	Brunei Darussalam	149.62 (132.75,166.48)
67	Japan	66.69 (59.50,70.22)
68	Republic of Korea	60.15 (51.83,68.91)
69	Republic of Singapore	148.22 (140.14,153.04)
71	Australia	175.07 (160.93,182.55)
72	New Zealand	185.37 (172.37,193.64)
74	Principality of Andorra	83.57 (62.19,109.06)
75	Republic of Austria	163.50 (150.03,169.73)
76	Kingdom of Belgium	134.49 (123.19,141.36)
77	Republic of Cyprus	271.51 (244.97,299.09)
78	Kingdom of Denmark	212.53 (198.63,220.27)
79	Republic of Finland	215.87 (199.55,224.26)
80	French Republic	84.26 (77.70,87.99)
81	Federal Republic of Germany	192.40 (176.05,200.95)
82	Hellenic Republic	142.04 (132.21,147.90)
83	Republic of Iceland	166.20 (151.65,175.42)
84	Ireland	227.01 (212.63,235.11)
85	State of Israel	177.30 (163.19,184.77)
86	Republic of Italy	108.50 (97.93,113.85)
87	Grand Duchy of Luxembourg	153.83 (144.60,160.39)
88	Republic of Malta	207.71 (192.05,218.97)
89	Kingdom of the Netherlands	141.63 (130.27,147.71)
90	Kingdom of Norway	175.82 (162.81,182.41)
91	Portuguese Republic	116.87 (109.19,121.40)
92	Kingdom of Spain	98.17 (89.76,102.96)
93	Kingdom of Sweden	181.58 (166.78,189.16)
94	Swiss Confederation	141.46 (129.90,148.32)
95	United Kingdom of Great Britain and Northern Ireland	196.49 (184.68,201.92)
97	Argentine Republic	158.80 (149.31,164.64)
98	Republic of Chile	118.48 (111.12,122.93)
99	Eastern Republic of Uruguay	152.28 (143.29,157.84)
101	Canada	156.66 (143.99,162.88)
102	United States of America	179.83 (162.05,188.39)
105	Antigua and Barbuda	127.16 (118.69,134.92)
106	Commonwealth of the Bahamas	130.35 (120.96,139.35)
107	Barbados	120.80 (112.91,126.46)
108	Belize	119.19 (110.42,126.07)
109	Republic of Cuba	214.24 (203.04,220.24)
110	Commonwealth of Dominica	151.50 (140.13,162.34)
111	Dominican Republic	150.36 (133.62,167.93)
112	Grenada	143.97 (131.72,156.44)
113	Republic of Guyana	230.59 (209.18,251.95)
114	Republic of Haiti	264.00 (219.99,304.41)
115	Jamaica	71.38 (66.35,74.66)

116	Saint Lucia	150.23 (143.53,156.46)
117	Saint Vincent and the Grenadines	176.86 (165.16,187.43)
118	Republic of Suriname	172.96 (158.64,185.24)
119	Republic of Trinidad and Tobago	234.72 (225.53,241.92)
121	Plurinational State of Bolivia	142.74 (113.77,184.17)
122	Republic of Ecuador	99.86 (94.74,103.33)
123	Republic of Peru	79.05 (69.19,89.61)
125	Republic of Colombia	143.52 (134.90,148.41)
126	Republic of Costa Rica	124.75 (114.39,131.17)
127	Republic of El Salvador	118.02 (108.48,125.47)
128	Republic of Guatemala	149.15 (142.83,153.93)
129	Republic of Honduras	112.05 (96.50,126.75)
130	United Mexican States	113.39 (108.31,115.87)
131	Republic of Nicaragua	89.16 (80.60,96.17)
132	Republic of Panama	105.33 (97.33,110.85)
133	Bolivarian Republic of Venezuela	169.56 (157.91,175.88)
135	Federative Republic of Brazil	136.61 (127.24,141.52)
136	Republic of Paraguay	109.31 (95.72,122.85)
139	People's Democratic Republic of Algeria	310.63 (267.49,359.89)
140	Kingdom of Bahrain	408.07 (381.77,433.03)
141	Arab Republic of Egypt	379.20 (343.43,420.07)
142	Islamic Republic of Iran	259.88 (238.06,276.23)
143	Republic of Iraq	249.67 (213.86,290.86)
144	Hashemite Kingdom of Jordan	199.16 (171.57,228.44)
145	State of Kuwait	198.32 (179.17,212.96)
146	Lebanese Republic	235.27 (199.02,287.02)
147	State of Libya	162.07 (128.59,199.64)
148	Kingdom of Morocco	296.20 (254.39,342.55)
149	Palestine	288.99 (240.40,346.99)
150	Sultanate of Oman	301.16 (238.04,376.57)
151	State of Qatar	384.95 (334.48,437.71)
152	Kingdom of Saudi Arabia	225.52 (174.28,279.62)
153	Syrian Arab Republic	370.80 (309.81,437.57)
154	Republic of Tunisia	218.26 (180.41,251.41)
155	Republic of Turkey	204.65 (180.71,228.18)
156	United Arab Emirates	266.92 (214.61,326.84)
157	Republic of Yemen	320.35 (252.11,407.07)
160	Islamic Republic of Afghanistan	361.39 (279.98,444.56)
161	People's Republic of Bangladesh	119.12 (101.74,138.51)
162	Kingdom of Bhutan	106.73 (78.16,137.30)
163	Republic of India	137.87 (121.54,151.08)
164	Federal Democratic Republic of Nepal	130.11 (102.83,160.78)
165	Islamic Republic of Pakistan	140.71 (111.59,162.54)
168	Republic of Angola	126.92 (102.51,155.46)
169	Central African Republic	165.96 (127.17,223.28)
170	Republic of the Congo	186.73 (149.01,224.12)
171	Democratic Republic of the Congo	130.63 (98.95,169.53)
172	Republic of Equatorial Guinea	159.29 (124.09,197.59)
173	Gabonese Republic	135.27 (108.08,162.06)
175	Republic of Burundi	104.94 (80.73,131.97)

176	Union of the Comoros	77.26 (60.01,98.64)
177	Republic of Djibouti	69.59 (50.20,92.41)
178	State of Eritrea	83.07 (60.89,105.60)
179	Federal Democratic Republic of Ethiopia	81.27 (68.45,99.83)
180	Republic of Kenya	39.81 (28.88,49.04)
181	Republic of Madagascar	82.43 (70.46,95.52)
182	Republic of Malawi	69.58 (59.67,81.51)
183	Republic of Mauritius	228.18 (214.34,239.27)
184	Republic of Mozambique	34.96 (29.53,41.14)
185	Republic of Rwanda	97.17 (72.20,127.11)
186	Republic of Seychelles	134.13 (123.05,144.58)
187	Federal Republic of Somalia	66.17 (49.89,90.31)
189	United Republic of Tanzania	66.88 (52.40,83.81)
190	Republic of Uganda	70.72 (54.09,87.52)
191	Republic of Zambia	62.20 (49.58,76.43)
193	Republic of Botswana	101.42 (75.69,127.42)
194	Kingdom of Lesotho	50.87 (39.57,66.28)
195	Republic of Namibia	104.53 (84.80,124.55)
196	Republic of South Africa	74.43 (61.29,84.33)
197	Kingdom of Eswatini	90.35 (70.96,112.97)
198	Republic of Zimbabwe	83.54 (68.17,98.94)
200	Republic of Benin	73.34 (62.22,86.00)
201	Burkina Faso	84.03 (68.86,103.12)
202	Republic of Cameroon	79.88 (61.99,104.29)
203	Republic of Cabo Verde	71.83 (59.60,82.89)
204	Republic of Chad	87.87 (70.37,109.87)
205	Republic of Côte d'Ivoire	118.09 (97.26,141.65)
206	Republic of the Gambia	112.57 (90.28,140.89)
207	Republic of Ghana	142.80 (119.35,169.63)
208	Republic of Guinea	92.10 (73.83,112.19)
209	Republic of Guinea-Bissau	150.59 (120.96,186.49)
210	Republic of Liberia	107.55 (91.01,125.79)
211	Republic of Mali	75.19 (57.11,96.95)
212	Islamic Republic of Mauritania	136.72 (109.19,169.77)
213	Republic of the Niger	68.91 (49.35,98.38)
214	Federal Republic of Nigeria	107.45 (85.67,131.35)
215	Democratic Republic of Sao Tome and Principe	80.51 (70.58,90.20)
216	Republic of Senegal	128.22 (105.19,151.19)
217	Republic of Sierra Leone	128.79 (108.47,151.69)
218	Togolese Republic	114.24 (95.44,135.97)
298	American Samoa	166.50 (150.04,183.69)
305	Bermuda	245.20 (230.52,257.73)
320	Cook Islands	160.27 (141.38,183.71)
349	Greenland	210.19 (192.06,230.77)
351	Guam	203.55 (187.20,218.36)
367	Principality of Monaco	103.52 (82.13,122.18)
369	Republic of Nauru	412.25 (337.73,489.41)
374	Republic of Niue	241.28 (204.64,282.86)
376	Northern Mariana Islands	134.08 (113.25,163.61)
380	Republic of Palau	249.87 (210.92,298.11)

385	Puerto Rico	161.56 (152.58,167.32)
393	Saint Kitts and Nevis	220.62 (208.45,231.84)
396	Republic of San Marino	76.92 (65.70,87.83)
413	Tokelau	225.76 (189.40,267.48)
416	Tuvalu	274.89 (236.08,314.05)
422	United States Virgin Islands	213.35 (187.38,239.88)
435	Republic of South Sudan	72.37 (55.04,93.38)
522	Republic of Sudan	339.57 (276.21,415.93)

ross 204 Countries and Territories: EAPC

ASR_EndYear_UI	EAPC_CI	Netdrift_CI
110.91 (92.42,128.56)	0.97 (0.64,1.29)	-0.02 (-0.22,0.18)
125.35 (100.79,153.33)	0.38 (0.19,0.56)	0.44 (0.35,0.54)
33.61 (30.00,36.21)	-2.46 (-2.71,-2.21)	-1.40 (-1.64,-1.17)
111.55 (89.23,134.56)	-0.17 (-0.26,-0.09)	-0.90 (-1.07,-0.74)
143.25 (119.34,163.30)	1.19 (1.09,1.29)	0.65 (0.60,0.70)
176.46 (141.65,213.26)	-1.07 (-1.17,-0.96)	-1.60 (-1.76,-1.43)
149.66 (138.24,159.74)	-0.64 (-0.80,-0.49)	-0.20 (-0.37,-0.04)
79.43 (66.25,93.77)	-2.80 (-2.91,-2.69)	-3.91 (-4.91,-2.90)
138.21 (113.02,171.61)	-1.25 (-1.34,-1.16)	-2.05 (-2.12,-1.97)
150.42 (129.19,171.93)	-0.35 (-0.43,-0.28)	-0.05 (-0.15,0.04)
94.59 (64.82,125.44)	-0.81 (-1.02,-0.59)	-1.24 (-1.40,-1.07)
47.05 (36.80,58.63)	-2.54 (-2.80,-2.27)	-2.42 (-2.60,-2.24)
156.95 (121.11,195.97)	1.12 (0.93,1.32)	1.08 (0.50,1.66)
76.99 (63.45,90.68)	0.96 (0.77,1.16)	0.97 (0.85,1.09)
266.78 (216.97,322.31)	-0.50 (-0.64,-0.35)	-0.64 (-1.05,-0.23)
202.94 (169.31,243.41)	0.24 (0.21,0.28)	0.26 (-1.07,1.61)
275.90 (219.83,337.91)	0.23 (0.12,0.35)	0.34 (-1.36,2.07)
266.43 (213.65,330.40)	0.03 (0.01,0.05)	0.12 (-1.05,1.30)
144.45 (109.76,186.31)	0.26 (0.14,0.37)	0.14 (-0.07,0.35)
226.72 (194.95,270.93)	0.37 (0.33,0.41)	0.70 (-0.38,1.78)
275.17 (228.47,339.14)	-0.20 (-0.32,-0.08)	-0.05 (-0.62,0.51)
139.13 (113.60,166.57)	0.15 (0.03,0.28)	0.25 (-1.60,2.14)
308.39 (260.86,357.06)	-0.10 (-0.14,-0.05)	-0.08 (-0.83,0.68)
209.78 (186.39,233.39)	-1.98 (-2.20,-1.75)	-2.42 (-2.78,-2.06)
306.13 (270.78,343.48)	-0.37 (-0.57,-0.18)	-2.28 (-2.51,-2.05)
124.16 (111.04,136.51)	-4.01 (-4.41,-3.61)	-4.78 (-5.44,-4.11)
235.98 (212.04,258.87)	-1.88 (-2.44,-1.32)	-4.67 (-5.16,-4.17)
274.41 (234.16,313.72)	0.36 (-0.01,0.72)	-1.20 (-1.36,-1.04)
219.59 (190.33,247.40)	-1.73 (-1.98,-1.48)	-2.29 (-2.61,-1.96)
244.92 (206.56,281.62)	-0.97 (-1.31,-0.63)	-2.31 (-2.60,-2.02)
343.68 (280.94,420.00)	-1.50 (-1.90,-1.11)	-2.50 (-2.73,-2.27)
339.48 (295.64,382.01)	0.30 (-0.05,0.65)	-0.53 (-0.72,-0.34)
158.29 (134.20,185.54)	0.09 (-0.12,0.30)	-0.80 (-1.23,-0.38)
138.30 (114.10,162.53)	-1.60 (-1.90,-1.30)	-2.53 (-2.98,-2.08)
204.40 (180.50,230.30)	-2.85 (-3.18,-2.51)	-2.39 (-2.81,-1.97)
130.52 (114.93,143.14)	-2.60 (-2.72,-2.47)	-3.39 (-4.31,-2.46)
126.68 (110.35,139.62)	-2.56 (-2.66,-2.45)	-3.83 (-4.22,-3.44)
155.36 (137.21,170.61)	-1.71 (-1.85,-1.58)	-3.40 (-3.95,-2.84)
195.86 (170.12,224.26)	-0.58 (-1.02,-0.13)	-2.57 (-3.08,-2.05)
212.83 (186.68,239.20)	0.97 (0.73,1.20)	-1.05 (-2.07,-0.02)
113.26 (100.87,122.58)	-3.08 (-3.22,-2.94)	-4.64 (-4.94,-4.34)
154.19 (140.01,169.13)	-2.45 (-2.63,-2.27)	-2.84 (-3.13,-2.56)
174.28 (150.25,200.22)	-2.56 (-2.84,-2.27)	-3.09 (-3.96,-2.21)
184.77 (160.51,206.70)	-1.77 (-1.84,-1.70)	-3.06 (-3.60,-2.53)
45.95 (39.17,51.61)	-3.85 (-4.04,-3.65)	-5.13 (-6.50,-3.74)
341.51 (292.04,394.62)	-0.30 (-0.61,0.02)	-1.58 (-2.36,-0.80)

92.06 (80.72,102.43)	-4.84 (-5.25,-4.43)	-6.35 (-7.30,-5.38)
160.30 (141.76,177.14)	-2.75 (-3.05,-2.45)	-4.21 (-4.94,-3.46)
197.08 (176.61,215.91)	-1.97 (-2.19,-1.74)	-2.99 (-3.68,-2.30)
230.52 (209.69,252.49)	-2.52 (-2.83,-2.21)	-1.78 (-2.16,-1.41)
212.91 (195.61,229.02)	-1.61 (-2.10,-1.12)	-2.75 (-3.10,-2.40)
373.47 (291.58,459.07)	-0.30 (-0.82,0.23)	-0.96 (-1.47,-0.45)
84.50 (74.32,95.88)	-1.42 (-1.66,-1.17)	-1.71 (-2.65,-0.76)
25.36 (21.70,27.29)	-2.95 (-3.18,-2.73)	-2.25 (-2.77,-1.73)
28.27 (22.87,32.57)	-3.03 (-3.30,-2.75)	-4.15 (-4.36,-3.93)
46.74 (41.78,49.85)	-3.96 (-4.20,-3.72)	-4.04 (-4.38,-3.70)
44.08 (37.95,47.53)	-4.68 (-4.79,-4.57)	-4.58 (-4.96,-4.19)
60.98 (52.78,65.35)	-3.81 (-3.89,-3.72)	-4.44 (-5.13,-3.74)
41.02 (30.25,52.92)	-2.06 (-2.29,-1.83)	-2.71 (-8.03,2.93)
68.11 (59.02,73.18)	-3.16 (-3.35,-2.98)	-4.42 (-5.00,-3.84)
38.04 (33.11,40.92)	-4.09 (-4.24,-3.94)	-4.56 (-5.01,-4.10)
86.95 (75.85,98.11)	-4.03 (-4.23,-3.83)	-3.85 (-5.15,-2.53)
39.95 (34.90,42.81)	-5.83 (-5.99,-5.66)	-5.62 (-6.17,-5.06)
77.38 (65.63,83.77)	-3.28 (-3.35,-3.22)	-4.17 (-4.62,-3.71)
29.87 (25.89,32.30)	-3.48 (-3.60,-3.36)	-3.44 (-3.78,-3.10)
63.67 (55.51,68.32)	-3.72 (-3.84,-3.61)	-4.15 (-4.44,-3.85)
72.90 (65.20,76.93)	-2.33 (-2.64,-2.02)	-1.96 (-2.29,-1.63)
57.95 (49.00,64.16)	-3.42 (-3.52,-3.32)	-3.75 (-5.59,-1.87)
52.77 (44.84,57.37)	-4.77 (-4.94,-4.59)	-5.21 (-5.76,-4.66)
34.77 (29.93,37.49)	-5.69 (-5.89,-5.48)	-6.08 (-6.55,-5.60)
44.27 (37.56,47.75)	-3.04 (-3.13,-2.95)	-3.55 (-3.75,-3.34)
45.70 (40.60,50.17)	-4.01 (-4.16,-3.86)	-5.47 (-7.62,-3.27)
71.19 (61.30,78.48)	-3.43 (-3.62,-3.23)	-3.44 (-5.01,-1.83)
37.42 (32.50,40.31)	-4.82 (-5.04,-4.60)	-5.93 (-6.58,-5.27)
40.88 (35.33,43.73)	-4.94 (-5.12,-4.77)	-5.96 (-6.78,-5.14)
36.80 (32.35,39.41)	-4.22 (-4.47,-3.98)	-3.99 (-4.38,-3.60)
35.37 (30.69,38.04)	-3.48 (-3.60,-3.35)	-3.61 (-3.80,-3.42)
51.80 (44.14,57.63)	-4.00 (-4.10,-3.90)	-4.56 (-5.22,-3.89)
39.96 (33.20,43.53)	-4.24 (-4.33,-4.15)	-5.41 (-5.97,-4.84)
52.11 (46.86,54.65)	-4.67 (-4.85,-4.49)	-4.45 (-4.83,-4.08)
60.79 (55.95,64.11)	-2.68 (-2.86,-2.50)	-2.85 (-3.04,-2.67)
39.05 (35.77,41.32)	-3.41 (-3.53,-3.28)	-2.04 (-2.30,-1.78)
60.55 (55.50,63.77)	-3.04 (-3.23,-2.85)	-2.71 (-3.19,-2.23)
51.12 (45.43,54.44)	-3.88 (-4.03,-3.73)	-3.57 (-3.87,-3.27)
78.92 (69.93,83.85)	-3.01 (-3.15,-2.86)	-2.51 (-2.69,-2.33)
71.43 (66.59,77.89)	-2.17 (-2.39,-1.96)	-3.15 (-6.42,0.23)
76.31 (63.63,92.15)	-1.89 (-2.08,-1.70)	-2.15 (-3.41,-0.87)
62.55 (51.40,74.63)	-2.26 (-2.57,-1.95)	-2.83 (-4.82,-0.79)
66.97 (59.34,74.28)	-2.42 (-2.77,-2.06)	-2.93 (-4.32,-1.52)
104.76 (91.34,117.29)	-2.56 (-2.86,-2.27)	-2.61 (-2.86,-2.35)
92.21 (81.49,107.84)	-1.81 (-2.07,-1.55)	-1.30 (-4.95,2.48)
144.66 (116.75,177.94)	0.54 (0.31,0.77)	1.17 (0.77,1.57)
95.36 (83.86,105.78)	-1.84 (-2.14,-1.55)	-2.25 (-4.88,0.45)
143.23 (115.50,177.30)	-1.30 (-1.56,-1.03)	-1.66 (-2.31,-1.01)
210.11 (159.57,271.20)	-0.57 (-0.65,-0.48)	-0.52 (-0.68,-0.36)
52.45 (42.06,66.21)	-0.64 (-1.14,-0.14)	-0.79 (-1.55,-0.02)

48.50 (40.20,56.66)	-4.25 (-4.67,-3.83)	-3.76 (-6.33,-1.13)
100.97 (90.71,111.62)	-2.03 (-2.28,-1.79)	-2.59 (-4.73,-0.41)
90.94 (69.95,113.45)	-1.91 (-2.19,-1.63)	-1.83 (-2.70,-0.96)
105.96 (83.20,131.89)	-3.12 (-3.37,-2.86)	-2.70 (-3.21,-2.19)
84.63 (62.40,121.70)	-1.60 (-1.83,-1.37)	-2.16 (-2.38,-1.94)
77.47 (64.11,93.12)	-0.89 (-1.44,-0.34)	-1.06 (-1.25,-0.88)
44.80 (34.86,55.87)	-2.25 (-2.73,-1.77)	-2.00 (-2.15,-1.86)
79.05 (66.64,93.24)	-2.13 (-2.32,-1.93)	-2.41 (-2.52,-2.30)
55.10 (48.17,61.10)	-2.65 (-2.82,-2.48)	-1.86 (-2.24,-1.48)
94.74 (78.35,113.90)	-0.79 (-0.99,-0.58)	-0.94 (-1.18,-0.69)
97.26 (85.34,109.04)	-1.36 (-1.77,-0.94)	-1.86 (-2.03,-1.69)
167.93 (139.63,201.92)	1.49 (1.25,1.74)	0.08 (-0.27,0.43)
113.21 (100.89,125.20)	-0.10 (-0.36,0.17)	0.52 (0.39,0.65)
81.93 (70.19,95.03)	0.06 (-0.16,0.28)	-0.32 (-0.64,0.01)
54.48 (43.03,64.93)	-1.97 (-2.21,-1.72)	-1.32 (-1.77,-0.87)
141.40 (112.21,177.15)	-0.98 (-1.26,-0.70)	-0.85 (-0.98,-0.73)
64.01 (58.63,67.35)	-2.32 (-2.41,-2.22)	-1.90 (-1.99,-1.80)
86.53 (67.26,106.48)	-0.43 (-0.67,-0.19)	-0.73 (-1.06,-0.40)
212.02 (172.72,251.71)	-1.21 (-1.26,-1.16)	-2.40 (-2.99,-1.81)
161.62 (140.64,184.38)	-3.56 (-3.94,-3.18)	-4.24 (-4.73,-3.74)
347.73 (297.31,402.14)	0.20 (-0.01,0.41)	-0.14 (-0.30,0.03)
146.11 (130.71,156.95)	-2.08 (-2.22,-1.93)	-2.38 (-2.47,-2.28)
254.83 (204.17,296.68)	-0.43 (-0.62,-0.25)	-1.44 (-1.61,-1.28)
98.28 (80.73,117.66)	-2.83 (-3.14,-2.52)	-3.53 (-3.75,-3.31)
109.10 (90.44,131.44)	-1.89 (-2.30,-1.47)	-2.05 (-2.35,-1.74)
92.09 (77.66,107.98)	-3.09 (-3.38,-2.79)	-3.25 (-3.54,-2.96)
178.47 (140.30,228.13)	0.83 (0.59,1.07)	0.75 (0.56,0.93)
267.15 (210.84,310.86)	-0.35 (-0.40,-0.30)	-1.33 (-1.41,-1.26)
188.68 (162.69,211.70)	-1.51 (-1.77,-1.25)	-1.83 (-2.12,-1.53)
179.25 (150.33,211.70)	-1.40 (-1.53,-1.27)	-2.13 (-2.52,-1.75)
123.16 (97.95,147.94)	-4.51 (-5.27,-3.75)	-4.72 (-5.24,-4.20)
185.90 (158.13,219.08)	-0.72 (-0.91,-0.52)	-0.11 (-0.28,0.05)
353.01 (281.64,432.02)	-0.47 (-0.58,-0.36)	-1.34 (-1.47,-1.21)
163.59 (121.84,215.67)	-1.24 (-1.37,-1.12)	-1.60 (-1.77,-1.44)
133.38 (109.89,155.94)	-1.29 (-1.59,-0.98)	-2.55 (-2.79,-2.30)
167.71 (136.64,196.33)	0.13 (-0.41,0.67)	-2.41 (-3.00,-1.81)
263.54 (201.84,344.23)	-0.85 (-0.94,-0.75)	-1.30 (-1.40,-1.20)
280.03 (221.41,346.20)	-0.98 (-1.08,-0.89)	-1.22 (-1.31,-1.13)
107.50 (86.20,131.71)	-0.19 (-0.33,-0.06)	-0.54 (-0.74,-0.34)
110.98 (86.52,133.92)	0.20 (0.17,0.24)	-0.60 (-1.29,0.11)
151.17 (137.46,165.08)	0.48 (0.30,0.65)	-0.07 (-0.27,0.12)
136.53 (111.67,169.87)	0.45 (0.28,0.62)	-0.02 (-0.12,0.08)
183.45 (154.60,226.81)	0.81 (0.69,0.94)	0.59 (0.53,0.66)
125.48 (99.59,156.40)	-0.24 (-0.32,-0.15)	-0.42 (-0.61,-0.23)
150.26 (109.43,210.57)	-0.42 (-0.50,-0.35)	-0.50 (-0.83,-0.16)
158.95 (127.71,189.82)	-0.80 (-0.93,-0.67)	-1.26 (-1.58,-0.94)
112.32 (83.28,149.12)	-0.65 (-0.74,-0.55)	-0.56 (-0.67,-0.45)
141.80 (102.97,188.38)	-0.57 (-0.77,-0.37)	-1.10 (-1.92,-0.27)
126.77 (99.33,155.15)	-0.33 (-0.45,-0.21)	-0.71 (-1.36,-0.05)
85.80 (68.00,108.32)	-1.17 (-1.36,-0.98)	-1.47 (-1.71,-1.23)

73.16 (55.29,95.05)	-0.44 (-0.56,-0.33)	-0.75 (-1.72,0.22)
91.49 (67.70,118.94)	0.80 (0.67,0.94)	0.47 (-0.42,1.38)
90.95 (69.21,117.14)	0.28 (0.20,0.35)	0.05 (-0.30,0.41)
55.81 (45.21,66.72)	-1.51 (-1.62,-1.40)	-2.18 (-2.27,-2.08)
61.50 (46.70,79.16)	1.71 (1.45,1.97)	1.39 (1.21,1.57)
100.02 (74.95,125.89)	0.27 (0.12,0.41)	-0.01 (-0.16,0.15)
84.27 (70.14,99.95)	0.39 (0.13,0.66)	0.12 (-0.08,0.32)
97.49 (90.28,102.00)	-3.21 (-3.48,-2.94)	-2.52 (-2.95,-2.10)
49.06 (36.92,61.05)	1.56 (1.38,1.74)	2.18 (1.90,2.46)
65.09 (46.31,88.10)	-2.22 (-2.59,-1.86)	-3.38 (-3.63,-3.13)
87.60 (78.75,97.97)	-1.27 (-1.40,-1.15)	-1.66 (-3.36,0.07)
68.48 (48.16,95.21)	0.20 (0.07,0.34)	0.06 (-0.20,0.32)
96.28 (72.01,123.39)	1.04 (0.92,1.17)	0.74 (0.62,0.86)
71.03 (56.38,91.34)	-0.61 (-1.00,-0.23)	-1.09 (-1.25,-0.93)
92.70 (71.66,117.34)	1.29 (1.18,1.40)	0.81 (0.57,1.05)
78.60 (62.09,98.66)	-0.86 (-1.12,-0.60)	-1.53 (-2.16,-0.90)
90.90 (63.42,134.25)	2.84 (2.31,3.38)	3.49 (2.66,4.33)
104.17 (81.37,129.06)	-0.17 (-0.46,0.11)	-0.61 (-1.23,0.01)
78.04 (71.05,84.66)	0.00 (-0.39,0.39)	-1.16 (-1.47,-0.85)
106.44 (75.27,145.87)	1.06 (0.55,1.58)	1.19 (0.40,1.99)
118.54 (97.50,145.32)	1.75 (1.21,2.29)	2.24 (1.97,2.51)
77.65 (65.25,93.07)	0.24 (0.08,0.41)	0.18 (-0.20,0.55)
97.57 (75.51,124.07)	0.75 (0.61,0.89)	0.48 (0.20,0.77)
103.29 (79.89,137.12)	0.95 (0.41,1.49)	0.60 (0.40,0.80)
121.51 (100.46,142.26)	1.14 (0.76,1.52)	0.01 (-1.09,1.12)
104.29 (80.34,131.28)	0.47 (0.26,0.68)	0.42 (0.13,0.72)
119.90 (98.75,152.67)	-0.06 (-0.29,0.17)	-0.27 (-0.44,-0.10)
140.02 (108.58,171.76)	0.61 (0.47,0.75)	0.44 (-0.17,1.05)
97.56 (78.35,118.19)	-1.76 (-2.09,-1.43)	-2.15 (-2.30,-2.00)
110.41 (85.87,140.24)	0.84 (0.71,0.98)	0.96 (0.68,1.25)
156.92 (123.29,192.38)	0.20 (0.11,0.29)	0.03 (-0.50,0.57)
114.25 (90.14,145.70)	0.18 (0.05,0.31)	0.40 (-0.05,0.85)
71.65 (56.24,90.99)	-0.04 (-0.14,0.06)	-0.06 (-0.33,0.21)
112.23 (85.32,144.29)	-0.95 (-1.14,-0.76)	-1.38 (-1.88,-0.88)
72.56 (51.59,98.73)	0.24 (0.14,0.33)	0.13 (-0.20,0.47)
109.48 (90.67,129.23)	0.14 (0.03,0.26)	-0.11 (-0.18,-0.04)
103.54 (89.77,120.61)	0.99 (0.86,1.12)	0.40 (-1.66,2.51)
121.62 (95.49,151.29)	-0.39 (-0.49,-0.30)	-0.71 (-0.94,-0.48)
129.53 (101.84,159.08)	0.20 (0.01,0.39)	0.52 (0.20,0.83)
117.00 (89.49,149.18)	-0.17 (-0.38,0.04)	-0.35 (-0.68,-0.03)
178.99 (155.05,207.56)	0.37 (0.32,0.43)	0.52 (-1.68,2.78)
68.35 (59.38,81.13)	-4.11 (-4.44,-3.78)	-4.14 (-8.64,0.57)
107.15 (88.99,128.05)	-1.25 (-1.37,-1.13)	-0.95 (-5.38,3.68)
81.20 (70.57,97.64)	-3.17 (-3.34,-2.99)	-3.49 (-8.39,1.66)
126.60 (113.51,139.39)	-0.74 (-1.07,-0.40)	0.66 (-0.49,1.83)
49.33 (40.10,59.33)	-2.48 (-2.56,-2.40)	-2.64 (-9.27,4.47)
432.64 (361.02,517.42)	0.13 (-0.14,0.40)	0.01 (-2.59,2.67)
243.51 (206.45,279.74)	-0.10 (-0.17,-0.03)	-0.32 (-9.29,9.53)
152.09 (134.52,164.56)	0.67 (0.49,0.85)	0.79 (-2.21,3.89)
233.62 (197.27,272.14)	-0.02 (-0.11,0.07)	0.11 (-2.61,2.90)

53.62 (44.64,61.73)	-3.84 (-4.07,-3.61)	-3.29 (-3.77,-2.80)
91.15 (78.72,103.50)	-2.76 (-2.99,-2.54)	-3.92 (-7.81,0.13)
23.19 (15.63,32.00)	-3.28 (-3.64,-2.92)	-3.18 (-14.10,9.12)
205.41 (164.75,248.55)	-0.31 (-0.39,-0.24)	-0.16 (-11.17,12.20)
269.54 (234.98,314.27)	0.01 (-0.04,0.06)	0.14 (-3.35,3.76)
102.62 (83.61,125.67)	-2.25 (-2.39,-2.11)	-1.97 (-3.85,-0.06)
81.22 (61.25,106.99)	0.27 (0.20,0.34)	0.47 (0.17,0.77)
255.88 (202.40,330.73)	-1.08 (-1.14,-1.02)	-1.47 (-1.54,-1.41)

Table S6. Shifts in Age Distribution of Ischemic Heart Disease (IHD) Deaths by SDI Region: Total Number and Proportion of Deaths Across Age Groups, 1990-2021.				
**location_name**	**age_strat**	**year**	**Number**	**proportion**
Global	15-29 years	1990	49838.97707	0.928595282
Global	15-29 years	2021	55810.20635	0.620690185
Global	30-44 years	1990	220873.0484	4.115286524
Global	30-44 years	2021	309168.8992	3.438405154
Global	45-59 years	1990	764850.6959	14.25062851
Global	45-59 years	2021	1182620.041	13.15244469
Global	60-75 years	1990	1760405.972	32.79972375
Global	60-75 years	2021	2771008.236	30.8176179
Global	75+ years	1990	2571167.888	47.90576593
Global	75+ years	2021	4673029.3	51.97084207
High SDI	15-29 years	1990	2663.840969	0.153766627
High SDI	15-29 years	2021	1648.202132	0.118373772
High SDI	30-44 years	1990	29092.45055	1.679322466
High SDI	30-44 years	2021	19065.63508	1.369292697
High SDI	45-59 years	1990	144133.6053	8.31991795
High SDI	45-59 years	2021	103683.7025	7.446556912
High SDI	60-75 years	1990	493182.3587	28.46828642
High SDI	60-75 years	2021	314589.9875	22.59383287
High SDI	75+ years	1990	1063319.892	61.37870653
High SDI	75+ years	2021	953383.5206	68.47194375
High-middle SDI	15-29 years	1990	8340.040889	0.519673343
High-middle SDI	15-29 years	2021	4782.347682	0.195163902
High-middle SDI	30-44 years	1990	51127.39293	3.185780929
High-middle SDI	30-44 years	2021	43159.17767	1.76129259
High-middle SDI	45-59 years	1990	195006.4752	12.15097963
High-middle SDI	45-59 years	2021	207082.6569	8.450882727
High-middle SDI	60-75 years	1990	494689.9166	30.82444873
High-middle SDI	60-75 years	2021	672606.0422	27.44853127
High-middle SDI	75+ years	1990	855698.3436	53.31911737
High-middle SDI	75+ years	2021	1522796.123	62.14412951
Middle SDI	15-29 years	1990	19088.43478	1.837249102
Middle SDI	15-29 years	2021	19310.85359	0.686944793
Middle SDI	30-44 years	1990	69585.46489	6.697554533
Middle SDI	30-44 years	2021	107629.8835	3.828716719
Middle SDI	45-59 years	1990	198976.3681	19.15134257
Middle SDI	45-59 years	2021	410025.0502	14.58581682
Middle SDI	60-75 years	1990	375999.6508	36.18971533
Middle SDI	60-75 years	2021	899685.4202	32.00450005
Middle SDI	75+ years	1990	375318.3279	36.12413846
Middle SDI	75+ years	2021	1374470.413	48.89402162
Low-middle SDI	15-29 years	1990	16152.33037	2.14469397
Low-middle SDI	15-29 years	2021	22305.58726	1.215793305
Low-middle SDI	30-44 years	1990	57371.36566	7.61772569
Low-middle SDI	30-44 years	2021	108419.4672	5.909535617
Low-middle SDI	45-59 years	1990	172794.3704	22.94350326
Low-middle SDI	45-59 years	2021	361287.4291	19.69241305

Low-middle SDI	60-75 years	1990	293389.9584	38.95609244
Low-middle SDI	60-75 years	2021	688232.3751	37.51294706
Low-middle SDI	75+ years	1990	213421.8198	28.33798465
Low-middle SDI	75+ years	2021	654408.0518	35.66931097
Low SDI	15-29 years	1990	3554.52389	1.553472659
Low SDI	15-29 years	2021	7722.342957	1.564887754
Low SDI	30-44 years	1990	13407.02542	5.859419732
Low SDI	30-44 years	2021	30650.14668	6.211073434
Low SDI	45-59 years	1990	52799.79534	23.0756751
Low SDI	45-59 years	2021	99483.11959	20.15967387
Low SDI	60-75 years	1990	100329.6827	43.84818439
Low SDI	60-75 years	2021	193166.5051	39.14406546
Low SDI	75+ years	1990	58720.45962	25.66324811
Low SDI	75+ years	2021	162453.7237	32.92029948
High-income North America	15-29 years	1990	619.1825783	0.096011593
High-income North America	15-29 years	2021	547.2434254	0.102332411
High-income North America	30-44 years	1990	9872.585027	1.530861245
High-income North America	30-44 years	2021	6523.619014	1.219891602
High-income North America	45-59 years	1990	51575.0333	7.997319796
High-income North America	45-59 years	2021	46267.44336	8.651833512
High-income North America	60-75 years	1990	190999.3827	29.61671659
High-income North America	60-75 years	2021	143031.3624	26.74631327
High-income North America	75+ years	1990	391837.7919	60.75909078
High-income North America	75+ years	2021	338400.7165	63.2796292
Southern Latin America	15-29 years	1990	261.2514774	0.416083183
Southern Latin America	15-29 years	2021	145.484124	0.29631072
Southern Latin America	30-44 years	1990	1494.866612	2.38080513
Southern Latin America	30-44 years	2021	870.1676296	1.77228958
Southern Latin America	45-59 years	1990	7168.49029	11.41692398
Southern Latin America	45-59 years	2021	4980.406887	10.14370442
Southern Latin America	60-75 years	1990	20140.06159	32.07614751
Southern Latin America	60-75 years	2021	14270.53612	29.06511527
Southern Latin America	75+ years	1990	33723.61089	53.7100402
Southern Latin America	75+ years	2021	28831.90695	58.72258001
High-income Asia Pacific	15-29 years	1990	466.4237654	0.396546093
High-income Asia Pacific	15-29 years	2021	128.462778	0.084126283
High-income Asia Pacific	30-44 years	1990	2657.868108	2.259677343
High-income Asia Pacific	30-44 years	2021	1276.857576	0.836174361
High-income Asia Pacific	45-59 years	1990	10925.675	9.288835735
High-income Asia Pacific	45-59 years	2021	8212.790647	5.378301465
High-income Asia Pacific	60-75 years	1990	29821.24041	25.35354598
High-income Asia Pacific	60-75 years	2021	26191.44615	17.15196445
High-income Asia Pacific	75+ years	1990	73750.36892	62.70139484
High-income Asia Pacific	75+ years	2021	116892.7542	76.54943344
Australasia	15-29 years	1990	45.30516458	0.112809195
Australasia	15-29 years	2021	15.3340951	0.053540123
Australasia	30-44 years	1990	564.7485909	1.406215706
Australasia	30-44 years	2021	298.2015685	1.041192758
Australasia	45-59 years	1990	3105.086941	7.731620926
Australasia	45-59 years	2021	1725.881683	6.026043119

Australasia	60-75 years	1990	13084.87317	32.58114223
Australasia	60-75 years	2021	4973.431123	17.36510138
Australasia	75+ years	1990	23360.86533	58.16821195
Australasia	75+ years	2021	21627.53211	75.51412262
Western Europe	15-29 years	1990	1030.603053	0.117208567
Western Europe	15-29 years	2021	233.6510621	0.043026618
Western Europe	30-44 years	1990	10606.20177	1.206223588
Western Europe	30-44 years	2021	3353.356578	0.617517378
Western Europe	45-59 years	1990	63379.12053	7.207989428
Western Europe	45-59 years	2021	27687.34193	5.098597299
Western Europe	60-75 years	1990	238652.9482	27.14155566
Western Europe	60-75 years	2021	93532.98535	17.22400899
Western Europe	75+ years	1990	565620.9917	64.32702276
Western Europe	75+ years	2021	418231.0796	77.01684971
East Asia	15-29 years	1990	10498.39732	1.840439852
East Asia	15-29 years	2021	6032.654843	0.300429364
East Asia	30-44 years	1990	36281.69857	6.360426447
East Asia	30-44 years	2021	41644.63694	2.073924684
East Asia	45-59 years	1990	93222.54781	16.34254133
East Asia	45-59 years	2021	176466.7201	8.788134886
East Asia	60-75 years	1990	208453.0525	36.54322593
East Asia	60-75 years	2021	517731.5865	25.78330358
East Asia	75+ years	1990	221973.0145	38.91336644
East Asia	75+ years	2021	1266135.458	63.05420749
Central Asia	15-29 years	1990	840.229499	0.637642719
Central Asia	15-29 years	2021	715.5832819	0.407986108
Central Asia	30-44 years	1990	4654.427751	3.532203966
Central Asia	30-44 years	2021	4672.296755	2.663885838
Central Asia	45-59 years	1990	18176.37338	13.79388865
Central Asia	45-59 years	2021	21814.46359	12.43740363
Central Asia	60-75 years	1990	39816.85062	30.21665502
Central Asia	60-75 years	2021	61056.18498	34.81086819
Central Asia	75+ years	1990	68283.32438	51.81960964
Central Asia	75+ years	2021	87135.50247	49.67985623
South Asia	15-29 years	1990	15408.1895	2.17561891
South Asia	15-29 years	2021	22288.47927	1.119960225
South Asia	30-44 years	1990	61518.26668	8.686309594
South Asia	30-44 years	2021	123106.9504	6.185926202
South Asia	45-59 years	1990	185728.9467	26.22471696
South Asia	45-59 years	2021	406897.8307	20.44596137
South Asia	60-75 years	1990	280717.812	39.63703718
South Asia	60-75 years	2021	762648.2419	38.32184719
South Asia	75+ years	1990	164847.7621	23.27631735
South Asia	75+ years	2021	675171.9651	33.92630501
Southeast Asia	15-29 years	1990	6385.900792	2.527598262
Southeast Asia	15-29 years	2021	7809.677374	1.222738347
Southeast Asia	30-44 years	1990	20933.45178	8.285652728
Southeast Asia	30-44 years	2021	37286.01118	5.837761727
Southeast Asia	45-59 years	1990	53569.90732	21.20346198
Southeast Asia	45-59 years	2021	126218.0187	19.76158605

Southeast Asia	60-75 years	1990	91177.13943	36.0887503
Southeast Asia	60-75 years	2021	230849.4908	36.14342965
Southeast Asia	75+ years	1990	80580.58533	31.89453674
Southeast Asia	75+ years	2021	236540.691	37.03448422
Oceania	15-29 years	1990	63.07821142	1.393012982
Oceania	15-29 years	2021	148.5526139	1.33388249
Oceania	30-44 years	1990	477.8751472	10.55334748
Oceania	30-44 years	2021	1130.105203	10.14743197
Oceania	45-59 years	1990	1366.048703	30.16768443
Oceania	45-59 years	2021	3275.209612	29.408737
Oceania	60-75 years	1990	1796.221209	39.6675715
Oceania	60-75 years	2021	4069.990171	36.54522449
Oceania	75+ years	1990	824.9621989	18.2183836
Oceania	75+ years	2021	2513.001532	22.56472406
Central Europe	15-29 years	1990	652.3594588	0.179175366
Central Europe	15-29 years	2021	161.9235383	0.04887796
Central Europe	30-44 years	1990	9748.308349	2.677445228
Central Europe	30-44 years	2021	2551.031693	0.770050026
Central Europe	45-59 years	1990	42368.25134	11.63675463
Central Europe	45-59 years	2021	19115.12937	5.770059975
Central Europe	60-75 years	1990	110949.955	30.47322847
Central Europe	60-75 years	2021	82796.43431	24.99278883
Central Europe	75+ years	1990	200371.0519	55.0333963
Central Europe	75+ years	2021	226656.7754	68.41822321
Eastern Europe	15-29 years	1990	1327.503984	0.168835515
Eastern Europe	15-29 years	2021	707.7861215	0.078327451
Eastern Europe	30-44 years	1990	18993.62953	2.415660721
Eastern Europe	30-44 years	2021	12575.28433	1.391649168
Eastern Europe	45-59 years	1990	86609.44084	11.01522086
Eastern Europe	45-59 years	2021	69651.23118	7.707983005
Eastern Europe	60-75 years	1990	231835.5562	29.48546753
Eastern Europe	60-75 years	2021	278257.3138	30.79346351
Eastern Europe	75+ years	1990	447504.4483	56.91481538
Eastern Europe	75+ years	2021	542432.9921	60.02857686
Andean Latin America	15-29 years	1990	419.6899288	2.455846993
Andean Latin America	15-29 years	2021	421.0508776	1.277279495
Andean Latin America	30-44 years	1990	874.0796746	5.11474256
Andean Latin America	30-44 years	2021	1182.568263	3.587381654
Andean Latin America	45-59 years	1990	2218.860049	12.98382545
Andean Latin America	45-59 years	2021	3577.886178	10.85370176
Andean Latin America	60-75 years	1990	5170.56996	30.25597666
Andean Latin America	60-75 years	2021	8868.658651	26.9035322
Andean Latin America	75+ years	1990	8406.217194	49.18960833
Andean Latin America	75+ years	2021	18914.49876	57.37810489
Central Latin America	15-29 years	1990	1183.89205	1.332285174
Central Latin America	15-29 years	2021	1795.502671	0.72677462
Central Latin America	30-44 years	1990	4145.363822	4.664958058
Central Latin America	30-44 years	2021	7782.685014	3.15023644
Central Latin America	45-59 years	1990	12969.36421	14.59498917
Central Latin America	45-59 years	2021	30093.08738	12.18092988

Central Latin America	60-75 years	1990	28817.32538	32.4293886
Central Latin America	60-75 years	2021	70159.24419	28.39870909
Central Latin America	75+ years	1990	41745.81428	46.978379
Central Latin America	75+ years	2021	137220.3026	55.54334997
Tropical Latin America	15-29 years	1990	853.4237263	0.793547572
Tropical Latin America	15-29 years	2021	955.8724481	0.588956955
Tropical Latin America	30-44 years	1990	6351.18537	5.905586609
Tropical Latin America	30-44 years	2021	6541.973412	4.030810543
Tropical Latin America	45-59 years	1990	21101.0055	19.6205603
Tropical Latin America	45-59 years	2021	28744.52362	17.71082234
Tropical Latin America	60-75 years	1990	39320.00551	36.56131643
Tropical Latin America	60-75 years	2021	58249.5279	35.89021177
Tropical Latin America	75+ years	1990	39919.75667	37.11898909
Tropical Latin America	75+ years	2021	67807.30627	41.77919838
Caribbean	15-29 years	1990	306.5133222	0.687201376
Caribbean	15-29 years	2021	311.5873821	0.507058481
Caribbean	30-44 years	1990	1457.479067	3.267660974
Caribbean	30-44 years	2021	1768.479133	2.877916099
Caribbean	45-59 years	1990	5776.11918	12.95003109
Caribbean	45-59 years	2021	8499.334625	13.83130369
Caribbean	60-75 years	1990	14670.29872	32.89073833
Caribbean	60-75 years	2021	19380.65364	31.53890486
Caribbean	75+ years	1990	22392.71955	50.20436824
Caribbean	75+ years	2021	31489.93445	51.24481687
North Africa and Middle East	15-29 years	1990	7460.345462	1.932549076
North Africa and Middle East	15-29 years	2021	8501.453297	1.105326343
North Africa and Middle East	30-44 years	1990	22392.94903	5.800733113
North Africa and Middle East	30-44 years	2021	38012.48117	4.942236972
North Africa and Middle East	45-59 years	1990	76291.17552	19.76268278
North Africa and Middle East	45-59 years	2021	135794.8369	17.65552373
North Africa and Middle East	60-75 years	1990	146778.2137	38.02184535
North Africa and Middle East	60-75 years	2021	259095.2409	33.68656924
North Africa and Middle East	75+ years	1990	133113.8497	34.48218969
North Africa and Middle East	75+ years	2021	327731.1261	42.61034371
Central Sub-Saharan Africa	15-29 years	1990	191.5304124	0.823020206
Central Sub-Saharan Africa	15-29 years	2021	443.0209143	0.888708424
Central Sub-Saharan Africa	30-44 years	1990	915.4187309	3.933621316
Central Sub-Saharan Africa	30-44 years	2021	2425.049006	4.864694672
Central Sub-Saharan Africa	45-59 years	1990	5068.613101	21.78020163
Central Sub-Saharan Africa	45-59 years	2021	11094.65415	22.25608834
Central Sub-Saharan Africa	60-75 years	1990	11733.48194	50.41963104
Central Sub-Saharan Africa	60-75 years	2021	21538.41898	43.20648027
Central Sub-Saharan Africa	75+ years	1990	5362.609528	23.04352581
Central Sub-Saharan Africa	75+ years	2021	14348.83049	28.78402829
Eastern Sub-Saharan Africa	15-29 years	1990	1063.754735	2.418213569
Eastern Sub-Saharan Africa	15-29 years	2021	2570.69261	2.539647821
Eastern Sub-Saharan Africa	30-44 years	1990	2774.573329	6.307385203
Eastern Sub-Saharan Africa	30-44 years	2021	7152.661333	7.066282721
Eastern Sub-Saharan Africa	45-59 years	1990	9200.247991	20.91475018
Eastern Sub-Saharan Africa	45-59 years	2021	19295.9267	19.06290079

Eastern Sub-Saharan Africa	60-75 years	1990	19676.64991	44.73055702
Eastern Sub-Saharan Africa	60-75 years	2021	39565.78006	39.0879667
Eastern Sub-Saharan Africa	75+ years	1990	11274.0539	25.62909403
Eastern Sub-Saharan Africa	75+ years	2021	32637.34456	32.24320196
Southern Sub-Saharan Africa	15-29 years	1990	217.2377504	1.213539754
Southern Sub-Saharan Africa	15-29 years	2021	223.7597643	0.561976412
Southern Sub-Saharan Africa	30-44 years	1990	1495.53848	8.354419962
Southern Sub-Saharan Africa	30-44 years	2021	2006.606992	5.039627214
Southern Sub-Saharan Africa	45-59 years	1990	3401.989866	19.00429339
Southern Sub-Saharan Africa	45-59 years	2021	7449.508545	18.70956602
Southern Sub-Saharan Africa	60-75 years	1990	5590.458609	31.22958027
Southern Sub-Saharan Africa	60-75 years	2021	14050.27428	35.28750019
Southern Sub-Saharan Africa	75+ years	1990	7195.940027	40.19816663
Southern Sub-Saharan Africa	75+ years	2021	16086.42626	40.40133016
Western Sub-Saharan Africa	15-29 years	1990	544.1648827	0.724421916
Western Sub-Saharan Africa	15-29 years	2021	1652.433859	1.024239783
Western Sub-Saharan Africa	30-44 years	1990	2662.533013	3.544508893
Western Sub-Saharan Africa	30-44 years	2021	7007.875944	4.343741382
Western Sub-Saharan Africa	45-59 years	1990	11628.39835	15.48035692
Western Sub-Saharan Africa	45-59 years	2021	25757.81556	15.96564926
Western Sub-Saharan Africa	60-75 years	1990	31203.87484	41.5402969
Western Sub-Saharan Africa	60-75 years	2021	60691.43408	37.61880145
Western Sub-Saharan Africa	75+ years	1990	29078.14932	38.71041537
Western Sub-Saharan Africa	75+ years	2021	66223.15648	41.04756813

Table S7. Trends in Sex-Specific Age-Standardized Mort
to-I

location_name	Mortality_Male_UI
Global	2804950.13 (2673799.04,2921926.62)
Global	2854407.15 (2727046.80,2961989.74)
Global	2928519.94 (2809679.20,3036741.72)
Global	3053991.69 (2932292.29,3152101.49)
Global	3132414.42 (3015737.86,3232024.36)
Global	3172417.33 (3043671.11,3264899.72)
Global	3178877.80 (3056547.28,3284105.74)
Global	3189252.15 (3062732.72,3287774.28)
Global	3225896.99 (3095689.98,3324718.38)
Global	3289853.05 (3151080.51,3390977.86)
Global	3351438.11 (3216186.47,3453559.10)
Global	3417593.58 (3272923.66,3516856.12)
Global	3504175.65 (3370670.39,3605818.46)
Global	3604281.72 (3474236.77,3705637.01)
Global	3647202.21 (3503812.04,3756036.56)
Global	3722419.30 (3582826.11,3827665.29)
Global	3718309.51 (3576426.11,3838356.22)
Global	3789162.32 (3635721.72,3911071.69)
Global	3894381.52 (3741229.20,4027747.91)
Global	3954608.44 (3797835.38,4085585.22)
Global	4068597.00 (3896003.81,4202601.80)
Global	4147954.05 (3955012.50,4316723.18)
Global	4236671.71 (4035497.66,4420161.53)
Global	4339636.54 (4112365.39,4514191.97)
Global	4430652.62 (4198940.52,4639727.48)
Global	4483229.07 (4232433.63,4684373.71)
Global	4566909.97 (4303493.87,4793916.29)
Global	4622499.38 (4362171.83,4864388.77)
Global	4733082.09 (4455361.86,5002294.65)
Global	4840900.55 (4553978.58,5141773.14)
Global	4919960.87 (4596547.59,5250883.84)
Global	5002681.10 (4679269.08,5336245.44)
High SDI	894445.33 (857227.43,912581.58)
High SDI	889562.97 (851129.49,908026.29)
High SDI	879603.05 (839517.77,897972.74)
High SDI	881021.72 (840174.08,899935.25)
High SDI	868214.56 (828779.51,887032.21)
High SDI	861680.43 (820800.60,880802.11)
High SDI	844081.78 (801129.57,862851.99)
High SDI	828648.11 (785565.53,847078.37)
High SDI	821395.64 (778764.26,840470.77)
High SDI	821119.94 (777107.40,839995.91)
High SDI	799958.65 (756933.80,819774.78)
High SDI	789474.72 (747049.39,809451.56)
High SDI	784026.07 (740150.88,804516.30)
High SDI	776482.53 (733764.07,796381.96)

High SDI	755862.61 (711129.21,776195.68)
High SDI	751371.25 (707329.34,772711.96)
High SDI	739967.22 (694218.53,761341.51)
High SDI	731771.21 (686729.94,753799.85)
High SDI	728601.62 (679270.83,750804.91)
High SDI	723092.39 (677731.79,745359.07)
High SDI	718767.79 (671483.35,742238.45)
High SDI	717268.04 (671704.01,740875.94)
High SDI	719314.99 (669878.45,743496.26)
High SDI	724209.48 (673864.02,750442.99)
High SDI	722053.37 (669963.37,749803.78)
High SDI	732110.94 (680916.54,759333.15)
High SDI	739558.25 (684705.12,768859.68)
High SDI	746263.37 (691995.31,775452.04)
High SDI	750734.00 (689787.52,781479.65)
High SDI	751910.12 (689397.92,783392.25)
High SDI	753937.85 (690197.28,786902.32)
High SDI	769662.82 (708119.36,804781.00)
High-middle SDI	771220.46 (740416.85,798682.96)
High-middle SDI	793017.84 (760832.09,819244.34)
High-middle SDI	839360.30 (809914.41,867847.56)
High-middle SDI	921951.77 (891173.01,946530.90)
High-middle SDI	973306.61 (946910.27,997797.15)
High-middle SDI	977888.27 (949392.85,1001232.67)
High-middle SDI	961980.10 (934189.77,986888.61)
High-middle SDI	946394.34 (917943.70,969603.65)
High-middle SDI	948135.22 (913508.54,974266.05)
High-middle SDI	993913.72 (960847.49,1018896.09)
High-middle SDI	1031579.84 (1000966.79,1057385.47)
High-middle SDI	1054533.55 (1023123.53,1083569.75)
High-middle SDI	1095627.41 (1063310.73,1123413.35)
High-middle SDI	1131544.86 (1093870.82,1161026.30)
High-middle SDI	1141038.05 (1101793.48,1174084.20)
High-middle SDI	1175575.53 (1136974.28,1204824.83)
High-middle SDI	1136179.54 (1095344.94,1168850.90)
High-middle SDI	1139936.14 (1097901.97,1171933.84)
High-middle SDI	1161208.29 (1115149.39,1199710.18)
High-middle SDI	1153803.70 (1105337.68,1192375.15)
High-middle SDI	1181681.00 (1130921.12,1228026.68)
High-middle SDI	1174838.66 (1126026.26,1226592.37)
High-middle SDI	1181816.41 (1126386.88,1240227.56)
High-middle SDI	1183225.91 (1117521.87,1243050.15)
High-middle SDI	1188267.64 (1125721.67,1254830.52)
High-middle SDI	1189215.08 (1116790.98,1260677.69)
High-middle SDI	1196363.95 (1124036.29,1277382.27)
High-middle SDI	1191545.73 (1099177.66,1281147.82)
High-middle SDI	1203570.23 (1114729.71,1299107.43)
High-middle SDI	1216246.61 (1112425.33,1325453.05)
High-middle SDI	1219710.55 (1112355.31,1333573.12)
High-middle SDI	1223783.46 (1106841.05,1349448.24)

Low SDI	128634.24 (112623.82,147547.43)
Low SDI	131486.53 (115366.03,148022.36)
Low SDI	134360.89 (118308.67,151178.53)
Low SDI	138337.78 (122823.61,154799.42)
Low SDI	143648.84 (128383.05,161660.53)
Low SDI	147273.14 (131546.46,163515.02)
Low SDI	150344.10 (134145.60,166446.93)
Low SDI	155481.49 (138877.96,172679.18)
Low SDI	160252.51 (143745.37,175807.06)
Low SDI	160654.43 (144971.62,176737.91)
Low SDI	163615.26 (146526.97,180728.19)
Low SDI	166640.90 (150112.56,183498.38)
Low SDI	168923.07 (154179.40,185195.83)
Low SDI	173454.95 (157676.58,187232.48)
Low SDI	175994.16 (158772.83,191644.73)
Low SDI	176932.65 (160822.49,191859.35)
Low SDI	181722.36 (164735.15,197061.86)
Low SDI	189266.01 (172349.96,206537.93)
Low SDI	195524.91 (176883.42,212838.48)
Low SDI	201819.46 (181898.74,220486.59)
Low SDI	209775.73 (189631.08,229546.68)
Low SDI	218212.89 (195850.77,238645.12)
Low SDI	226831.57 (204445.85,251840.77)
Low SDI	237246.40 (215418.46,260833.23)
Low SDI	246250.47 (223342.60,273609.39)
Low SDI	254442.91 (228100.40,283037.18)
Low SDI	261244.44 (235222.84,290102.25)
Low SDI	264375.38 (240861.95,291438.87)
Low SDI	270009.82 (243124.58,299394.33)
Low SDI	280238.16 (254248.31,309652.41)
Low SDI	288276.35 (259127.19,321356.59)
Low SDI	290534.52 (258974.35,323350.32)
Low-middle SDI	425985.40 (383770.79,465066.96)
Low-middle SDI	435383.51 (396920.11,471690.69)
Low-middle SDI	447229.35 (407982.38,485712.11)
Low-middle SDI	461597.49 (422830.12,500890.82)
Low-middle SDI	476685.85 (441171.64,513358.29)
Low-middle SDI	492341.85 (453390.30,528205.94)
Low-middle SDI	506739.66 (466337.36,546278.60)
Low-middle SDI	525159.26 (485632.30,562616.85)
Low-middle SDI	541188.57 (500468.95,576553.27)
Low-middle SDI	544214.69 (503942.54,582971.97)
Low-middle SDI	556599.41 (518721.88,593055.50)
Low-middle SDI	576809.54 (535633.79,613993.92)
Low-middle SDI	593602.05 (555078.08,629962.31)
Low-middle SDI	616441.17 (573951.51,655191.94)
Low-middle SDI	629630.15 (585701.39,670741.80)
Low-middle SDI	643711.25 (601750.04,681955.63)
Low-middle SDI	673024.96 (627145.09,714423.80)
Low-middle SDI	706416.32 (662327.53,746943.37)

Low-middle SDI	736312.12 (687191.66,780398.26)
Low-middle SDI	756937.20 (710167.22,800708.22)
Low-middle SDI	787144.37 (737094.36,831087.44)
Low-middle SDI	821360.77 (771159.63,869540.57)
Low-middle SDI	851439.02 (799119.13,905203.18)
Low-middle SDI	883403.30 (836285.52,939066.74)
Low-middle SDI	921583.15 (864290.11,976274.97)
Low-middle SDI	924186.39 (864375.87,982918.77)
Low-middle SDI	946144.20 (885905.65,1004858.62)
Low-middle SDI	967302.19 (910375.36,1026137.60)
Low-middle SDI	1010218.66 (946818.60,1082774.34)
Low-middle SDI	1041608.41 (970599.93,1106404.70)
Low-middle SDI	1061255.96 (989095.95,1152855.54)
Low-middle SDI	1077885.58 (987649.88,1169582.87)
Middle SDI	579841.48 (539200.13,621212.00)
Middle SDI	600028.22 (558312.48,634916.36)
Middle SDI	622919.49 (579742.82,661501.98)
Middle SDI	645958.53 (608370.94,680153.27)
Middle SDI	665363.58 (629078.49,699276.07)
Middle SDI	687905.62 (649292.84,720426.97)
Middle SDI	710355.45 (672612.00,747273.57)
Middle SDI	728129.90 (693805.94,762669.97)
Middle SDI	749789.29 (716969.62,785904.17)
Middle SDI	765182.72 (727410.44,800383.96)
Middle SDI	795159.33 (755596.99,832968.42)
Middle SDI	825606.42 (785407.34,866231.45)
Middle SDI	857464.86 (818197.67,893948.41)
Middle SDI	901787.24 (862040.27,939161.00)
Middle SDI	940122.59 (894534.22,982312.91)
Middle SDI	970221.50 (927135.95,1008712.95)
Middle SDI	982831.96 (935194.01,1028526.89)
Middle SDI	1017177.53 (968778.22,1062812.58)
Middle SDI	1068125.48 (1018257.56,1118333.73)
Middle SDI	1114311.97 (1062480.31,1164481.75)
Middle SDI	1166581.75 (1108855.34,1221546.59)
Middle SDI	1211642.41 (1142435.69,1278392.17)
Middle SDI	1252568.66 (1182567.62,1330520.17)
Middle SDI	1306842.88 (1226472.93,1381309.81)
Middle SDI	1347774.02 (1270888.33,1437332.31)
Middle SDI	1378439.58 (1288004.94,1466084.66)
Middle SDI	1418755.12 (1323275.24,1520506.84)
Middle SDI	1448105.75 (1346106.78,1559906.63)
Middle SDI	1493596.78 (1377294.06,1618107.98)
Middle SDI	1545919.40 (1424989.47,1679862.24)
Middle SDI	1591742.88 (1457295.68,1739679.54)
Middle SDI	1635811.02 (1499835.51,1799723.34)

ality Rates (ASMR) for Ischemic Heart Disease Across Global and SDI Regions: Male-Female Ratio Analysis, 1990-2021.

Mortality_Female_UI	ASR_Male_UI	ASR_Female_UI
2562186.45 (2357409.87,2702952.82)	187.66 (177.77,195.35)	134.50 (122.46,142.23)
2592752.17 (2405878.88,2724065.98)	185.60 (175.92,192.99)	132.29 (121.19,139.45)
2647461.06 (2442595.49,2782234.53)	184.90 (175.71,192.22)	131.46 (120.29,138.45)
2752601.14 (2544992.00,2887316.92)	187.18 (178.27,193.71)	133.12 (121.45,139.88)
2810131.02 (2597734.48,2945588.77)	186.38 (177.82,192.68)	132.32 (121.30,138.90)
2836027.54 (2626400.44,2976584.54)	183.95 (175.02,189.85)	130.18 (119.39,136.97)
2839455.32 (2619914.73,2976253.76)	179.87 (171.11,186.24)	127.08 (115.94,133.52)
2844268.57 (2630888.38,2987750.27)	175.91 (167.73,181.70)	123.99 (113.71,130.54)
2868393.55 (2631516.33,3009371.00)	173.42 (164.72,179.14)	121.79 (110.85,127.94)
2931451.64 (2712851.31,3072571.82)	171.83 (163.13,177.62)	121.16 (111.08,127.32)
2951178.05 (2711824.20,3098339.71)	170.00 (161.66,175.70)	118.58 (108.22,124.77)
2993334.96 (2756366.54,3141428.60)	168.62 (160.69,174.06)	116.93 (106.76,122.94)
3065785.42 (2826236.02,3210419.53)	168.12 (160.80,173.45)	116.49 (106.70,122.20)
3127875.00 (2870922.63,3282319.04)	168.56 (160.90,173.68)	115.66 (105.52,121.62)
3125160.34 (2870996.83,3277412.70)	165.84 (157.94,171.27)	112.26 (102.38,117.99)
3171579.37 (2928281.58,3328167.04)	164.26 (156.13,169.30)	110.54 (101.41,116.21)
3160863.41 (2903759.15,3313495.25)	159.20 (151.58,164.70)	106.87 (97.55,112.23)
3193366.39 (2915809.74,3355798.91)	157.47 (149.95,162.88)	104.73 (95.18,110.25)
3260023.93 (2959669.00,3427882.97)	156.95 (148.93,162.67)	103.70 (93.60,109.19)
3297560.48 (2997212.96,3478853.14)	154.72 (147.19,160.16)	101.65 (91.99,107.37)
3360722.69 (3060205.61,3535317.67)	154.39 (146.90,159.90)	100.33 (91.01,105.63)
3418096.57 (3091900.84,3615924.57)	152.51 (144.17,158.92)	98.82 (89.20,104.67)
3481760.72 (3142073.93,3681893.12)	150.91 (142.53,157.61)	97.49 (87.81,103.18)
3551640.90 (3200952.26,3767018.08)	150.44 (141.35,156.78)	96.40 (86.75,102.31)
3610431.68 (3259658.95,3834876.04)	149.41 (140.52,156.71)	95.01 (85.64,100.96)
3651288.94 (3265248.68,3872168.83)	146.47 (137.51,153.35)	93.19 (83.29,98.86)
3709821.28 (3339722.03,3944700.99)	144.40 (134.43,151.90)	91.79 (82.64,97.60)
3739771.69 (3376692.07,4006311.50)	141.54 (132.79,149.01)	89.69 (81.03,96.06)
3815264.82 (3451852.96,4074623.15)	140.28 (131.26,148.36)	88.73 (80.37,94.74)
3873814.34 (3466719.13,4158246.92)	139.04 (130.34,147.86)	87.35 (78.22,93.75)
3919419.82 (3476162.56,4242887.10)	137.66 (127.94,146.87)	85.94 (76.34,93.00)
3988955.58 (3540381.49,4317969.43)	136.84 (127.37,145.90)	85.32 (75.90,92.31)
837946.82 (735973.52,885805.82)	212.44 (201.21,217.78)	118.68 (104.55,125.36)
836610.46 (731947.90,885968.87)	206.95 (195.68,212.44)	115.51 (101.47,122.24)
831228.55 (726167.72,881167.58)	200.46 (189.18,205.65)	111.89 (98.29,118.45)
838064.80 (730114.92,888316.81)	196.73 (185.53,201.77)	110.06 (96.48,116.42)
829811.79 (721056.73,880451.33)	189.75 (179.27,194.73)	106.35 (93.08,112.54)
824355.09 (717220.02,876264.05)	184.05 (173.55,188.91)	102.94 (90.30,109.11)
813809.82 (705836.21,865725.52)	176.49 (165.87,181.19)	99.19 (86.80,105.17)
804481.88 (695948.61,856455.43)	169.32 (158.97,173.85)	95.75 (83.64,101.57)
801187.03 (692104.95,854733.16)	163.88 (153.70,168.48)	93.07 (81.24,98.83)
803783.45 (693303.55,858982.04)	159.91 (149.92,164.31)	91.13 (79.48,96.94)
781152.84 (671022.45,836288.31)	151.87 (142.28,156.37)	86.34 (75.04,91.97)
769902.62 (659277.48,827098.36)	146.16 (136.96,150.57)	83.12 (72.03,88.82)
761803.03 (652497.67,818439.41)	141.51 (132.45,145.88)	80.34 (69.68,85.83)
749597.51 (638648.46,807530.09)	136.45 (127.74,140.57)	77.26 (66.67,82.73)

723085.58 (614084.71,779318.25)	129.11 (120.39,133.24)	72.75 (62.64,77.96)
710809.42 (602334.50,766474.56)	124.57 (116.35,128.61)	69.56 (59.83,74.49)
694401.19 (585490.72,750160.90)	119.16 (110.99,123.03)	66.19 (56.79,70.99)
681665.65 (572085.39,737623.48)	114.31 (106.57,118.16)	63.27 (53.98,67.91)
673698.46 (564624.32,730433.06)	110.42 (102.27,114.13)	60.87 (51.89,65.44)
659490.48 (551449.08,715551.25)	106.24 (99.05,109.79)	57.98 (49.35,62.37)
644581.56 (536932.37,701231.11)	102.32 (95.11,105.91)	54.99 (46.69,59.28)
640103.15 (530695.91,696873.62)	99.21 (92.51,102.70)	53.12 (44.96,57.36)
635110.09 (524549.66,691481.51)	96.54 (89.57,99.98)	51.29 (43.29,55.34)
632225.18 (522543.27,690142.48)	94.26 (87.58,97.81)	49.75 (42.04,53.73)
625555.80 (514351.72,682722.07)	91.19 (84.43,94.80)	47.98 (40.43,51.70)
625839.10 (513742.72,684830.86)	89.67 (83.35,93.07)	46.77 (39.53,50.54)
623520.15 (516020.09,681663.45)	87.99 (81.39,91.49)	45.50 (38.78,49.14)
621997.71 (507737.50,682709.62)	86.22 (79.98,89.62)	44.27 (37.17,47.95)
620017.11 (509178.23,681436.34)	84.29 (77.58,87.78)	43.08 (36.35,46.72)
613888.85 (503646.09,675453.61)	82.08 (75.43,85.43)	41.71 (35.34,45.27)
615529.23 (501652.04,677831.42)	80.17 (73.66,83.60)	40.83 (34.42,44.33)
622708.22 (507042.48,687228.73)	80.02 (73.72,83.64)	40.64 (34.19,44.21)
833641.71 (775908.12,870047.90)	228.08 (217.26,235.79)	166.79 (153.25,174.63)
849139.83 (794442.73,885708.18)	227.57 (216.44,235.25)	165.09 (152.50,172.95)
879524.74 (820542.85,917012.67)	232.64 (222.00,240.20)	166.79 (154.21,174.30)
956152.07 (892096.56,993233.12)	245.78 (233.93,252.68)	176.70 (163.18,183.99)
997762.10 (933324.69,1034745.17)	250.67 (241.28,257.86)	179.70 (166.24,186.98)
1000288.37 (935221.39,1038703.27)	245.87 (235.77,252.43)	176.00 (163.25,183.27)
992116.66 (922474.51,1029496.63)	236.80 (227.53,243.34)	170.50 (157.06,177.23)
987355.94 (919100.66,1025240.20)	228.02 (218.27,234.26)	165.71 (153.09,172.57)
986984.24 (915502.87,1026339.44)	223.32 (212.74,230.22)	162.06 (149.05,168.89)
1028888.04 (962894.31,1067710.49)	226.78 (216.96,232.96)	164.94 (153.18,171.66)
1047633.75 (975983.44,1088931.74)	228.33 (219.66,234.93)	163.73 (151.50,170.69)
1065014.52 (993745.56,1107023.45)	227.15 (217.21,234.32)	161.95 (149.85,168.78)
1099770.56 (1025807.41,1144492.88)	229.82 (220.50,235.90)	163.27 (150.94,170.34)
1132765.83 (1048114.03,1182330.97)	233.08 (222.98,239.75)	164.44 (150.91,172.09)
1128355.68 (1048484.62,1177789.59)	229.59 (219.64,236.51)	159.29 (146.96,166.76)
1155290.05 (1070687.44,1203390.03)	230.18 (220.23,236.41)	158.20 (145.37,165.25)
1128867.11 (1048095.80,1179351.61)	217.01 (206.66,223.67)	149.93 (138.39,157.06)
1134077.89 (1043266.55,1187408.53)	212.82 (203.02,219.55)	146.28 (133.80,153.47)
1153468.30 (1054145.68,1207356.42)	210.74 (200.24,218.19)	144.60 (131.35,151.77)
1156669.69 (1056111.70,1213838.20)	204.48 (194.27,211.74)	140.77 (128.08,148.00)
1178133.95 (1073127.91,1240940.46)	203.51 (193.00,211.78)	139.06 (126.21,146.73)
1177080.14 (1065402.95,1244362.58)	196.57 (187.02,205.39)	134.59 (121.44,142.52)
1172941.23 (1058437.59,1239943.81)	192.06 (181.71,201.75)	130.03 (117.09,137.62)
1176314.51 (1056859.40,1255595.87)	186.91 (176.13,196.40)	126.67 (113.48,135.31)
1178920.37 (1060460.97,1251753.43)	182.03 (170.87,192.51)	123.23 (110.67,130.92)
1180018.88 (1050511.79,1259063.85)	177.29 (165.14,187.42)	119.79 (106.62,127.91)
1177921.76 (1055278.76,1266893.89)	173.08 (160.96,184.68)	115.89 (103.80,124.71)
1178021.33 (1056216.44,1277612.70)	167.66 (154.30,179.89)	112.48 (100.79,122.10)
1189628.40 (1045667.61,1290201.93)	164.57 (151.34,177.61)	110.53 (97.36,119.86)
1198676.18 (1059852.47,1303595.55)	161.70 (147.85,176.19)	108.21 (95.89,117.66)
1204596.12 (1045357.59,1335963.55)	158.43 (144.39,172.99)	105.95 (92.12,117.46)
1226642.89 (1051753.17,1359541.30)	155.11 (140.29,170.66)	105.32 (90.67,116.71)

100177.25 (85287.80,116183.90)	129.50 (112.91,148.05)	108.99 (93.07,125.86)
102012.80 (86772.58,118556.43)	129.55 (113.48,146.10)	108.55 (92.53,125.16)
104371.62 (88828.07,121621.64)	129.33 (112.79,146.69)	108.68 (92.68,126.06)
106597.57 (89966.14,123588.24)	130.02 (114.61,145.61)	108.53 (92.20,124.49)
110062.36 (92686.52,128692.08)	132.97 (118.09,149.19)	110.12 (93.09,127.40)
112966.98 (96413.30,131641.56)	133.49 (118.29,148.54)	111.15 (95.03,127.63)
114686.68 (96900.24,133039.70)	132.56 (117.68,146.52)	109.66 (93.35,125.41)
116725.43 (99152.37,135762.51)	133.54 (119.11,148.37)	108.59 (93.60,124.56)
119318.54 (101539.36,138292.30)	135.32 (120.42,148.37)	107.72 (92.85,123.75)
121371.32 (104479.02,141555.92)	133.57 (119.45,146.90)	106.28 (91.86,122.30)
124072.97 (106153.84,143130.49)	133.59 (118.84,147.52)	105.60 (90.81,120.14)
126892.19 (109145.24,145146.93)	132.35 (119.07,146.21)	105.33 (91.03,119.68)
130673.36 (112150.86,149729.25)	131.14 (118.43,143.76)	106.06 (91.71,120.65)
132643.97 (112500.53,152228.46)	132.69 (119.77,143.52)	104.89 (89.82,119.08)
132377.86 (112850.87,150486.35)	131.34 (118.13,142.68)	102.08 (87.87,114.91)
133143.77 (113303.01,151236.33)	128.41 (115.59,139.48)	100.04 (85.82,112.69)
136682.50 (116463.64,154856.37)	129.20 (116.17,140.69)	99.91 (85.88,112.28)
139623.54 (118873.91,158535.02)	131.29 (118.55,143.10)	99.20 (84.98,111.92)
144139.60 (123207.04,162432.79)	132.42 (119.78,145.12)	99.32 (85.87,111.72)
147187.10 (124443.66,165571.98)	132.64 (119.49,145.17)	98.32 (83.57,110.57)
150322.96 (128492.15,170058.34)	134.43 (121.39,147.59)	97.41 (83.55,109.62)
157564.59 (134608.27,176983.54)	136.50 (122.15,149.69)	98.95 (84.99,111.61)
170231.73 (145380.10,191212.75)	138.49 (124.31,153.60)	104.20 (88.83,117.03)
175495.45 (150057.53,197508.24)	143.72 (129.49,158.19)	106.60 (90.90,120.20)
182311.68 (155156.83,206239.92)	148.06 (133.29,163.25)	108.25 (92.15,121.96)
185155.24 (157364.62,208261.99)	148.41 (132.78,164.37)	105.99 (89.89,118.99)
188656.06 (162532.31,211861.84)	148.08 (133.36,164.34)	104.21 (89.98,118.02)
187148.27 (161209.41,211513.52)	144.59 (131.67,158.82)	99.88 (85.82,112.45)
192267.11 (167159.39,216850.09)	140.95 (126.64,155.45)	98.88 (85.92,111.32)
196552.02 (171087.14,224225.68)	141.42 (127.75,156.23)	97.54 (84.75,110.31)
199753.90 (173215.38,227178.24)	141.91 (127.63,158.07)	96.11 (83.73,108.45)
202941.32 (175450.04,231420.17)	139.53 (124.25,154.72)	95.23 (82.22,107.68)
327144.44 (290976.76,361968.31)	153.63 (137.58,167.66)	127.69 (114.15,142.04)
331525.37 (296311.49,364227.09)	153.02 (138.15,166.31)	125.58 (111.59,138.85)
341314.55 (306202.20,377806.50)	153.34 (139.30,166.70)	125.78 (112.42,139.15)
347965.22 (310489.23,381684.31)	154.39 (140.59,167.55)	124.53 (111.21,136.72)
356411.33 (320213.76,390022.34)	155.57 (142.94,168.18)	123.98 (111.49,135.87)
367778.94 (330045.77,405632.50)	157.06 (144.31,169.55)	125.23 (112.64,138.06)
376614.44 (340670.82,411115.57)	157.95 (144.80,170.90)	124.57 (112.65,136.49)
386069.38 (346204.32,421055.74)	160.00 (147.55,172.38)	123.44 (110.59,135.34)
397635.75 (357915.19,435538.69)	161.07 (148.45,172.07)	123.40 (110.97,134.96)
400743.95 (360867.94,436164.79)	157.16 (144.68,168.69)	120.50 (108.39,131.42)
403535.49 (364522.97,438812.21)	156.48 (144.15,167.23)	117.59 (106.03,127.98)
415372.43 (374792.71,450559.45)	158.41 (146.89,168.81)	117.14 (105.23,126.88)
430661.17 (389765.25,470758.39)	159.09 (147.85,170.04)	117.58 (105.99,128.46)
442680.26 (402094.23,478755.97)	161.79 (150.52,172.45)	117.30 (105.88,126.73)
446866.97 (407757.94,484348.42)	161.59 (150.26,172.05)	114.91 (104.22,125.09)
458747.61 (416846.05,497430.62)	160.67 (149.25,170.84)	114.26 (102.96,123.77)
478755.53 (436724.50,517193.26)	163.48 (151.31,173.82)	115.72 (104.88,124.87)
496255.04 (453133.74,534395.85)	167.18 (155.98,177.37)	116.58 (105.85,125.81)

513280.38 (468926.22,552068.59)	169.48 (158.62,180.12)	116.92 (106.44,125.59)
526032.71 (479757.84,564662.06)	169.17 (158.62,179.40)	115.65 (104.97,124.32)
543473.88 (493838.24,581611.08)	171.07 (159.93,181.42)	115.22 (104.24,123.21)
562545.34 (508246.97,601568.44)	172.88 (161.01,183.82)	115.00 (103.86,123.18)
596601.28 (543558.34,640273.86)	173.76 (162.41,185.07)	117.61 (106.51,126.43)
621177.26 (568247.41,669793.51)	178.40 (166.53,189.81)	119.47 (108.35,128.42)
651147.27 (593876.08,701601.26)	184.97 (172.34,196.01)	122.19 (110.96,131.79)
666982.14 (606563.36,717700.15)	178.41 (166.69,189.92)	120.62 (109.94,129.93)
689807.79 (631835.63,743360.78)	175.53 (163.83,186.30)	120.36 (109.93,129.39)
697834.39 (640411.51,752043.13)	173.34 (162.22,183.84)	117.86 (108.43,126.91)
723773.70 (666782.95,779758.77)	175.51 (163.64,187.99)	118.01 (108.67,127.05)
741327.97 (679755.55,806315.14)	175.59 (163.43,186.87)	117.03 (106.54,127.28)
749814.41 (686401.32,813571.31)	174.64 (162.21,188.89)	114.91 (105.06,124.30)
756767.33 (685841.26,822796.50)	174.68 (160.53,188.84)	113.10 (102.58,122.81)
459126.76 (423237.54,497557.13)	147.15 (136.08,157.39)	109.12 (99.40,118.82)
469187.15 (433646.74,508052.76)	146.95 (135.66,155.50)	107.81 (99.29,117.07)
486642.13 (449230.42,526336.98)	147.62 (137.62,156.35)	108.36 (99.55,117.70)
499317.17 (463885.54,537464.68)	148.41 (138.79,155.94)	107.83 (99.09,116.45)
511487.18 (476176.21,550156.63)	147.95 (138.85,155.46)	107.06 (98.15,115.69)
525911.01 (489784.89,568119.85)	148.45 (139.13,155.66)	107.13 (98.56,116.16)
537422.45 (503097.17,577146.64)	149.04 (140.50,156.91)	106.09 (98.17,114.28)
544762.41 (509990.43,586210.72)	148.38 (140.22,155.51)	103.71 (95.68,111.52)
558641.70 (519810.00,596573.81)	148.35 (139.70,155.15)	102.70 (94.08,110.09)
572346.80 (533510.26,608966.86)	146.41 (137.26,153.21)	101.67 (93.56,108.48)
590657.63 (552807.88,628720.71)	147.73 (139.81,154.73)	101.48 (93.29,108.48)
612022.45 (572421.88,651998.07)	149.29 (141.10,156.27)	101.93 (93.82,109.05)
638749.38 (591784.16,676682.66)	150.35 (142.11,157.06)	102.96 (94.18,109.49)
666010.03 (620297.34,705086.44)	154.08 (146.53,160.53)	103.95 (95.22,110.55)
690309.24 (641649.01,728826.76)	156.06 (148.05,162.71)	104.35 (95.55,110.72)
709371.69 (664254.44,751437.08)	156.01 (148.19,162.02)	103.64 (95.77,110.19)
717968.74 (669091.76,754797.86)	152.20 (144.05,159.04)	100.82 (92.85,106.37)
737526.32 (684955.96,782480.95)	152.22 (144.04,159.14)	99.62 (91.41,106.17)
771178.83 (713380.13,820126.89)	154.71 (146.99,162.04)	100.38 (91.54,106.90)
803866.57 (740713.34,862159.25)	156.49 (148.32,163.65)	100.73 (91.24,108.12)
839909.33 (773433.06,894864.78)	158.79 (149.52,166.18)	101.42 (92.13,108.54)
876498.08 (799544.74,936787.46)	158.93 (148.97,168.15)	101.71 (91.63,109.29)
902512.12 (825022.47,968018.38)	158.34 (148.82,168.04)	100.37 (90.94,107.94)
942076.11 (855110.36,1013354.51)	160.56 (149.60,169.98)	100.83 (90.70,108.87)
968127.96 (883837.86,1042879.85)	160.42 (150.31,170.94)	99.56 (90.15,107.62)
988829.36 (894714.08,1070862.42)	158.17 (147.16,167.90)	97.54 (87.41,106.13)
1025478.17 (925088.11,1113949.86)	156.59 (145.03,167.86)	97.06 (86.82,105.66)
1050254.83 (948759.35,1147987.79)	153.64 (141.92,165.69)	95.41 (85.76,104.71)
1085045.81 (966360.44,1176954.92)	152.47 (140.46,165.49)	94.51 (83.45,102.70)
1118842.81 (1000628.22,1229432.81)	152.26 (140.55,165.75)	93.50 (82.83,103.02)
1145165.27 (1024968.61,1273033.75)	152.06 (138.15,166.15)	92.10 (81.58,102.54)
1175310.60 (1033467.06,1302347.30)	152.31 (138.83,166.98)	91.33 (79.72,101.29)

Table S8. Local Drift Analysis of Ischemic Heart Disease Mortality: Age-Specific Percent Changes in Mortality Rates by Sex Across SDI Quintiles, 1990-2021.

location_name	Age	sex	Percent_Change	CILo	CIHi
Global	17.5	Male	-0.533208706	-1.400013374	0.341216149
Global	22.5	Male	-0.268429367	-0.788258781	0.254123744
Global	27.5	Male	-0.270565205	-0.633507843	0.093703105
Global	32.5	Male	-0.446629391	-0.707500275	-0.185073123
Global	37.5	Male	-0.711943456	-0.910517251	-0.512971723
Global	42.5	Male	-0.892963374	-1.046347828	-0.739341164
Global	47.5	Male	-0.996608661	-1.119901096	-0.873162495
Global	52.5	Male	-1.031280884	-1.133549825	-0.928906155
Global	57.5	Male	-1.101650955	-1.189211173	-1.014013145
Global	62.5	Male	-1.25813411	-1.33554067	-1.180666822
Global	67.5	Male	-1.430625602	-1.501997742	-1.359201744
Global	72.5	Male	-1.385332912	-1.455792011	-1.314823434
Global	77.5	Male	-1.144662659	-1.218134415	-1.071136256
Global	82.5	Male	-0.996805928	-1.077969375	-0.915575888
Global	87.5	Male	-0.840315709	-0.947228665	-0.733287357
Global	92.5	Male	-0.777261349	-0.962842761	-0.591332184
Global	17.5	Female	-1.301511534	-1.898121048	-0.701273722
Global	22.5	Female	-1.223572646	-1.617042273	-0.82852939
Global	27.5	Female	-1.098355434	-1.397229026	-0.79857593
Global	32.5	Female	-1.025188669	-1.258960561	-0.790863317
Global	37.5	Female	-0.903301831	-1.091544362	-0.714701037
Global	42.5	Female	-0.88776414	-1.037307097	-0.737995208
Global	47.5	Female	-0.94845362	-1.068473237	-0.828288399
Global	52.5	Female	-1.145356549	-1.242717919	-1.047899193
Global	57.5	Female	-1.334843325	-1.414151556	-1.255471294
Global	62.5	Female	-1.591795323	-1.657740328	-1.525806098
Global	67.5	Female	-1.7509721	-1.806718102	-1.69519445
Global	72.5	Female	-1.692811434	-1.742636297	-1.642961307
Global	77.5	Female	-1.502354699	-1.548244649	-1.45644336
Global	82.5	Female	-1.55714143	-1.601312605	-1.512950426
Global	87.5	Female	-1.681176284	-1.732638863	-1.629686754
Global	92.5	Female	-1.791864171	-1.872153645	-1.711509003
Global	17.5	Both	-0.842986726	-1.369547534	-0.313614754
Global	22.5	Both	-0.614599446	-0.942912858	-0.285197878
Global	27.5	Both	-0.546604586	-0.783092993	-0.309552498
Global	32.5	Both	-0.627491074	-0.802482157	-0.452191294
Global	37.5	Both	-0.774730339	-0.910316845	-0.638958307
Global	42.5	Both	-0.902608354	-1.008200486	-0.796903589
Global	47.5	Both	-1.004143899	-1.088945081	-0.919270014
Global	52.5	Both	-1.093879266	-1.163703698	-1.024005507
Global	57.5	Both	-1.194301716	-1.2531426	-1.135425771
Global	62.5	Both	-1.374323469	-1.42524227	-1.323378366
Global	67.5	Both	-1.541830028	-1.587258526	-1.49638056
Global	72.5	Both	-1.486808702	-1.529779824	-1.443818827
Global	77.5	Both	-1.288108027	-1.330379918	-1.245818025
Global	82.5	Both	-1.272424945	-1.315898685	-1.228932053

Global	87.5	Both	-1.312467088	-1.36582917	-1.259076136
Global	92.5	Both	-1.402000717	-1.488450814	-1.315474755
High-middle SDI	17.5	Male	-1.646899312	-3.563856292	0.308162925
High-middle SDI	22.5	Male	-1.351712323	-2.414575221	-0.277273132
High-middle SDI	27.5	Male	-1.466587334	-2.139758252	-0.78878574
High-middle SDI	32.5	Male	-1.876290748	-2.320698015	-1.42986158
High-middle SDI	37.5	Male	-2.433467427	-2.756146601	-2.109717523
High-middle SDI	42.5	Male	-2.82002324	-3.057169362	-2.582296999
High-middle SDI	47.5	Male	-2.802341627	-2.985212053	-2.619126495
High-middle SDI	52.5	Male	-2.605967713	-2.752380813	-2.459334178
High-middle SDI	57.5	Male	-2.407648787	-2.528409428	-2.286738532
High-middle SDI	62.5	Male	-2.262812101	-2.366587199	-2.158926701
High-middle SDI	67.5	Male	-2.263908553	-2.358001473	-2.169724961
High-middle SDI	72.5	Male	-1.890915379	-1.984785622	-1.796955236
High-middle SDI	77.5	Male	-1.280174934	-1.377841694	-1.182411453
High-middle SDI	82.5	Male	-1.037017961	-1.142499486	-0.931423887
High-middle SDI	87.5	Male	-0.973117328	-1.112713697	-0.833323894
High-middle SDI	92.5	Male	-0.87872077	-1.131509526	-0.62528568
High-middle SDI	17.5	Female	-2.430769185	-4.772274778	-0.031689514
High-middle SDI	22.5	Female	-2.325038055	-3.701359755	-0.929045658
High-middle SDI	27.5	Female	-2.344538155	-3.274913603	-1.405213648
High-middle SDI	32.5	Female	-2.503821204	-3.16311647	-1.840037254
High-middle SDI	37.5	Female	-2.810048313	-3.312050189	-2.305440054
High-middle SDI	42.5	Female	-3.022605545	-3.396072376	-2.647694906
High-middle SDI	47.5	Female	-2.916783988	-3.198464774	-2.634283544
High-middle SDI	52.5	Female	-2.928772294	-3.143673468	-2.713394306
High-middle SDI	57.5	Female	-2.862863876	-3.026988319	-2.698461656
High-middle SDI	62.5	Female	-2.709886497	-2.836148296	-2.583460625
High-middle SDI	67.5	Female	-2.663213374	-2.763141886	-2.563182166
High-middle SDI	72.5	Female	-2.303244639	-2.388974381	-2.217439602
High-middle SDI	77.5	Female	-1.661195129	-1.735724073	-1.586609659
High-middle SDI	82.5	Female	-1.534700624	-1.602842543	-1.466511516
High-middle SDI	87.5	Female	-1.568712364	-1.648806314	-1.488553188
High-middle SDI	92.5	Female	-1.568954915	-1.700043014	-1.437692002
High-middle SDI	17.5	Both	-1.822348231	-3.655801225	0.04599581
High-middle SDI	22.5	Both	-1.561887623	-2.595354909	-0.517455207
High-middle SDI	27.5	Both	-1.647146708	-2.313099675	-0.976653794
High-middle SDI	32.5	Both	-2.000371268	-2.447750058	-1.550940779
High-middle SDI	37.5	Both	-2.499376428	-2.827820126	-2.169822585
High-middle SDI	42.5	Both	-2.850205227	-3.092164654	-2.607641676
High-middle SDI	47.5	Both	-2.824467333	-3.0099324	-2.63864762
High-middle SDI	52.5	Both	-2.694309108	-2.84082882	-2.547568439
High-middle SDI	57.5	Both	-2.523058378	-2.64136843	-2.404604555
High-middle SDI	62.5	Both	-2.381396662	-2.479593226	-2.283101222
High-middle SDI	67.5	Both	-2.3815342	-2.466259794	-2.296735008
High-middle SDI	72.5	Both	-2.035680127	-2.114693159	-1.956603315
High-middle SDI	77.5	Both	-1.447008968	-1.522301949	-1.37165842
High-middle SDI	82.5	Both	-1.300820009	-1.375090121	-1.226493968
High-middle SDI	87.5	Both	-1.339207635	-1.430600976	-1.247729555
High-middle SDI	92.5	Both	-1.348913396	-1.503105466	-1.194479946

Low SDI	17.5	Male	-0.475430246	-1.63707112	0.699929309
Low SDI	22.5	Male	-0.374965258	-1.16929467	0.425748396
Low SDI	27.5	Male	-0.251525091	-0.860441578	0.36113137
Low SDI	32.5	Male	-0.250178931	-0.71191912	0.213708585
Low SDI	37.5	Male	-0.204439764	-0.56409262	0.156513931
Low SDI	42.5	Male	-0.179501685	-0.46158512	0.103381151
Low SDI	47.5	Male	-0.398941371	-0.629988159	-0.167357372
Low SDI	52.5	Male	-0.609881402	-0.805860588	-0.413515018
Low SDI	57.5	Male	-0.601601234	-0.771980927	-0.430928991
Low SDI	62.5	Male	-0.53562153	-0.689664843	-0.381339276
Low SDI	67.5	Male	-0.260851995	-0.407963908	-0.113522775
Low SDI	72.5	Male	0.1426921	-0.010025346	0.295642796
Low SDI	77.5	Male	0.609144486	0.433224649	0.785372467
Low SDI	82.5	Male	1.088685397	0.860506558	1.31738045
Low SDI	87.5	Male	1.481625554	1.129064479	1.835415744
Low SDI	92.5	Male	1.715687792	1.023482368	2.412636156
Low SDI	17.5	Female	-1.669558755	-2.392977239	-0.940778624
Low SDI	22.5	Female	-1.665287733	-2.188155075	-1.139625328
Low SDI	27.5	Female	-1.46518819	-1.900915458	-1.02752555
Low SDI	32.5	Female	-1.296491434	-1.658349372	-0.933302005
Low SDI	37.5	Female	-1.180203154	-1.483300864	-0.87617293
Low SDI	42.5	Female	-1.138338145	-1.386754852	-0.889295652
Low SDI	47.5	Female	-1.18573972	-1.389992331	-0.981064038
Low SDI	52.5	Female	-1.288963166	-1.459570534	-1.118060417
Low SDI	57.5	Female	-1.207232608	-1.350894694	-1.063361307
Low SDI	62.5	Female	-1.111578416	-1.2372678	-0.985729074
Low SDI	67.5	Female	-0.780161254	-0.8946015	-0.665588861
Low SDI	72.5	Female	-0.415689351	-0.527991051	-0.303260865
Low SDI	77.5	Female	-0.042729122	-0.164027418	0.078716547
Low SDI	82.5	Female	0.144050715	-0.006403903	0.294731714
Low SDI	87.5	Female	0.186168177	-0.036437414	0.409269481
Low SDI	92.5	Female	0.145695673	-0.267576682	0.56068055
Low SDI	17.5	Both	-1.10527937	-1.961658717	-0.241419422
Low SDI	22.5	Both	-1.014963719	-1.616351348	-0.4099
Low SDI	27.5	Both	-0.794653446	-1.274242349	-0.312734801
Low SDI	32.5	Both	-0.665349148	-1.044093428	-0.285155261
Low SDI	37.5	Both	-0.5656104	-0.869641395	-0.260646947
Low SDI	42.5	Both	-0.522084651	-0.764643836	-0.278932583
Low SDI	47.5	Both	-0.685129924	-0.884091638	-0.485768822
Low SDI	52.5	Both	-0.872076909	-1.039871827	-0.703997481
Low SDI	57.5	Both	-0.849305929	-0.993456146	-0.704945833
Low SDI	62.5	Both	-0.788201013	-0.916860435	-0.659374526
Low SDI	67.5	Both	-0.506912079	-0.627408017	-0.386270031
Low SDI	72.5	Both	-0.127257052	-0.249296125	-0.005068673
Low SDI	77.5	Both	0.284087074	0.147546309	0.420813998
Low SDI	82.5	Both	0.617083889	0.443782182	0.790684603
Low SDI	87.5	Both	0.827050273	0.565227525	1.08955468
Low SDI	92.5	Both	0.904816658	0.405772945	1.406340752
High SDI	17.5	Male	-2.598125052	-5.373132277	0.258261439
High SDI	22.5	Male	-1.532556562	-3.009760671	-0.032853984

High SDI	27.5	Male	-1.097393681	-1.991052318	-0.195586545
High SDI	32.5	Male	-1.333127736	-1.902726631	-0.760221481
High SDI	37.5	Male	-1.975509959	-2.363151163	-1.586329728
High SDI	42.5	Male	-2.447085061	-2.721003467	-2.172395355
High SDI	47.5	Male	-2.580732495	-2.781678281	-2.379371363
High SDI	52.5	Male	-2.623158292	-2.777441412	-2.46863034
High SDI	57.5	Male	-2.736135262	-2.859708897	-2.612404427
High SDI	62.5	Male	-3.150272691	-3.253678896	-3.046755961
High SDI	67.5	Male	-3.658187956	-3.748223331	-3.568068361
High SDI	72.5	Male	-4.037420803	-4.119355582	-3.955416006
High SDI	77.5	Male	-4.015134795	-4.093458615	-3.93674701
High SDI	82.5	Male	-3.757194019	-3.835842364	-3.678481352
High SDI	87.5	Male	-3.194399363	-3.289305431	-3.099400159
High SDI	92.5	Male	-2.702728123	-2.856275725	-2.54893782
High SDI	17.5	Female	-2.839977846	-5.751964566	0.161980686
High SDI	22.5	Female	-1.825600851	-3.466537524	-0.156770501
High SDI	27.5	Female	-1.287867486	-2.351549111	-0.212599209
High SDI	32.5	Female	-1.268287963	-1.985778371	-0.545545333
High SDI	37.5	Female	-1.623888832	-2.133282718	-1.111843563
High SDI	42.5	Female	-1.892079919	-2.261209768	-1.521555977
High SDI	47.5	Female	-2.064912766	-2.335997574	-1.793075512
High SDI	52.5	Female	-2.331806382	-2.536002493	-2.12718246
High SDI	57.5	Female	-2.784556325	-2.940636701	-2.628224957
High SDI	62.5	Female	-3.526818071	-3.648812654	-3.404669025
High SDI	67.5	Female	-4.225565559	-4.322758886	-4.128273499
High SDI	72.5	Female	-4.571365316	-4.650414659	-4.492250437
High SDI	77.5	Female	-4.511184371	-4.576735429	-4.445588282
High SDI	82.5	Female	-4.229321518	-4.284135147	-4.174476499
High SDI	87.5	Female	-3.671762871	-3.725774738	-3.617720703
High SDI	92.5	Female	-3.128872237	-3.203839797	-3.053846614
High SDI	17.5	Both	-2.635194176	-5.113743442	-0.091902062
High SDI	22.5	Both	-1.571073836	-2.911283102	-0.212364367
High SDI	27.5	Both	-1.098356593	-1.924012477	-0.265749897
High SDI	32.5	Both	-1.262690143	-1.796249237	-0.726232124
High SDI	37.5	Both	-1.842336922	-2.208930395	-1.474369185
High SDI	42.5	Both	-2.271185889	-2.531604376	-2.010071609
High SDI	47.5	Both	-2.429464186	-2.620461724	-2.23809203
High SDI	52.5	Both	-2.529359373	-2.675305854	-2.383194033
High SDI	57.5	Both	-2.722344825	-2.837876619	-2.606675657
High SDI	62.5	Both	-3.208069275	-3.302967869	-3.113077547
High SDI	67.5	Both	-3.752591753	-3.832983101	-3.6721332
High SDI	72.5	Both	-4.100718276	-4.171082701	-4.030302184
High SDI	77.5	Both	-4.08489122	-4.148319474	-4.021420992
High SDI	82.5	Both	-3.86669377	-3.925154166	-3.8081978
High SDI	87.5	Both	-3.369489424	-3.432910955	-3.30602624
High SDI	92.5	Both	-2.906938727	-3.000015992	-2.81377215
Low-middle SDI	17.5	Male	-0.454047132	-1.455135227	0.55721072
Low-middle SDI	22.5	Male	-0.313770553	-0.921536434	0.297723478
Low-middle SDI	27.5	Male	-0.192275086	-0.631662176	0.249054886
Low-middle SDI	32.5	Male	-0.122770503	-0.449519001	0.205050462

Low-middle SDI	37.5	Male	-0.037871507	-0.295375826	0.220297861
Low-middle SDI	42.5	Male	0.11698602	-0.088981396	0.323378038
Low-middle SDI	47.5	Male	0.137150703	-0.034446335	0.309042297
Low-middle SDI	52.5	Male	0.250822455	0.104274057	0.397585393
Low-middle SDI	57.5	Male	0.329323715	0.199735923	0.459079102
Low-middle SDI	62.5	Male	0.242692736	0.123141585	0.362386636
Low-middle SDI	67.5	Male	0.244483274	0.128120814	0.360980962
Low-middle SDI	72.5	Male	0.32457631	0.203150328	0.446149437
Low-middle SDI	77.5	Male	0.586139019	0.449152872	0.72331198
Low-middle SDI	82.5	Male	0.939991929	0.771094425	1.109172514
Low-middle SDI	87.5	Male	1.296388713	1.055472358	1.537879412
Low-middle SDI	92.5	Male	1.429265832	0.996911174	1.863471344
Low-middle SDI	17.5	Female	-2.014064809	-2.801002713	-1.220755736
Low-middle SDI	22.5	Female	-1.871655246	-2.402008989	-1.338419526
Low-middle SDI	27.5	Female	-1.522922564	-1.934555284	-1.109562003
Low-middle SDI	32.5	Female	-1.253018077	-1.583353578	-0.921573805
Low-middle SDI	37.5	Female	-0.876889269	-1.149451404	-0.603575596
Low-middle SDI	42.5	Female	-0.638221076	-0.861962517	-0.413974681
Low-middle SDI	47.5	Female	-0.509655733	-0.696069963	-0.322891564
Low-middle SDI	52.5	Female	-0.432207876	-0.588868025	-0.275300849
Low-middle SDI	57.5	Female	-0.366257205	-0.499206277	-0.23313049
Low-middle SDI	62.5	Female	-0.53587565	-0.653035888	-0.418577245
Low-middle SDI	67.5	Female	-0.453060645	-0.560166989	-0.345838937
Low-middle SDI	72.5	Female	-0.380580005	-0.485125952	-0.275924226
Low-middle SDI	77.5	Female	-0.188879263	-0.298591049	-0.07904675
Low-middle SDI	82.5	Female	0.110141361	-0.020356486	0.240809539
Low-middle SDI	87.5	Female	0.298410621	0.116608723	0.480542653
Low-middle SDI	92.5	Female	0.12750427	-0.189505758	0.44552116
Low-middle SDI	17.5	Both	-1.184353027	-1.969505553	-0.392912003
Low-middle SDI	22.5	Both	-0.973729467	-1.473871897	-0.471048194
Low-middle SDI	27.5	Both	-0.716159226	-1.088652354	-0.342263315
Low-middle SDI	32.5	Both	-0.543033304	-0.828551841	-0.256692748
Low-middle SDI	37.5	Both	-0.3553837	-0.584238313	-0.126002264
Low-middle SDI	42.5	Both	-0.180834605	-0.36555994	0.004233217
Low-middle SDI	47.5	Both	-0.132278435	-0.28615136	0.021831938
Low-middle SDI	52.5	Both	-0.031353632	-0.161969405	0.099433023
Low-middle SDI	57.5	Both	0.046558383	-0.067238096	0.160484445
Low-middle SDI	62.5	Both	-0.080500176	-0.183641859	0.022748086
Low-middle SDI	67.5	Both	-0.070781108	-0.168700983	0.027234811
Low-middle SDI	72.5	Both	-0.011036626	-0.110294571	0.08831995
Low-middle SDI	77.5	Both	0.19074981	0.082352857	0.299264164
Low-middle SDI	82.5	Both	0.497605642	0.366147903	0.629235563
Low-middle SDI	87.5	Both	0.756579484	0.57132	0.942180229
Low-middle SDI	92.5	Both	0.717800732	0.390321512	1.046348209
Middle SDI	17.5	Male	-0.201335648	-0.988165521	0.591747027
Middle SDI	22.5	Male	0.13413467	-0.328708531	0.599127174
Middle SDI	27.5	Male	0.120515893	-0.199654476	0.441713404
Middle SDI	32.5	Male	0.015779706	-0.218253456	0.250361782
Middle SDI	37.5	Male	-0.141310438	-0.323883738	0.041597275
Middle SDI	42.5	Male	-0.216393385	-0.361697684	-0.070877186

Middle SDI	47.5	Male	-0.275970506	-0.395205202	-0.156593077
Middle SDI	52.5	Male	-0.275859308	-0.377120909	-0.17449478
Middle SDI	57.5	Male	-0.298611417	-0.388069783	-0.20907271
Middle SDI	62.5	Male	-0.34884432	-0.42962096	-0.26800215
Middle SDI	67.5	Male	-0.358739665	-0.434900421	-0.282520652
Middle SDI	72.5	Male	-0.139920745	-0.216560836	-0.063221789
Middle SDI	77.5	Male	0.19868397	0.116904613	0.280530128
Middle SDI	82.5	Male	0.582285802	0.485808389	0.678855844
Middle SDI	87.5	Male	0.959465186	0.827029737	1.092074588
Middle SDI	92.5	Male	0.984997119	0.750919281	1.219618797
Middle SDI	17.5	Female	-1.259550172	-2.361602567	-0.145058825
Middle SDI	22.5	Female	-1.139382222	-1.834375371	-0.439468659
Middle SDI	27.5	Female	-1.073117223	-1.586638839	-0.556916047
Middle SDI	32.5	Female	-1.071668623	-1.471803663	-0.669908586
Middle SDI	37.5	Female	-0.937542266	-1.261716487	-0.612303726
Middle SDI	42.5	Female	-0.846543254	-1.105496309	-0.586912135
Middle SDI	47.5	Female	-0.903976916	-1.112401098	-0.695113441
Middle SDI	52.5	Female	-1.021149319	-1.192463034	-0.849538579
Middle SDI	57.5	Female	-1.035706542	-1.178770712	-0.892435258
Middle SDI	62.5	Female	-1.106176846	-1.228286708	-0.983916022
Middle SDI	67.5	Female	-1.013220938	-1.120357696	-0.905968096
Middle SDI	72.5	Female	-0.733099985	-0.832958569	-0.633140846
Middle SDI	77.5	Female	-0.392792718	-0.489866626	-0.295624112
Middle SDI	82.5	Female	-0.134295219	-0.237702213	-0.030781039
Middle SDI	87.5	Female	-0.076633467	-0.209188163	0.056097305
Middle SDI	92.5	Female	-0.165643517	-0.391379066	0.060603599
Middle SDI	17.5	Both	-0.593199306	-1.308600476	0.127387715
Middle SDI	22.5	Both	-0.304073219	-0.735683301	0.129413542
Middle SDI	27.5	Both	-0.266934862	-0.572223751	0.039291405
Middle SDI	32.5	Both	-0.321317858	-0.549093209	-0.093020826
Middle SDI	37.5	Both	-0.390295537	-0.570039014	-0.210227131
Middle SDI	42.5	Both	-0.42913098	-0.572303427	-0.285752369
Middle SDI	47.5	Both	-0.509500634	-0.626254838	-0.392609257
Middle SDI	52.5	Both	-0.564830448	-0.662918656	-0.466645385
Middle SDI	57.5	Both	-0.591282431	-0.67636103	-0.506130955
Middle SDI	62.5	Both	-0.644955151	-0.720253275	-0.569599919
Middle SDI	67.5	Both	-0.623235216	-0.692315283	-0.554107095
Middle SDI	72.5	Both	-0.386095202	-0.453401705	-0.318743191
Middle SDI	77.5	Both	-0.061895795	-0.130723691	0.006979535
Middle SDI	82.5	Both	0.230359056	0.153088415	0.307689314
Middle SDI	87.5	Both	0.393478361	0.291132507	0.495928658
Middle SDI	92.5	Both	0.298151598	0.121042394	0.475574101

Table S9. Age Effects on Ischemic Heart Disease Mortality Rates by Sex Across SDI Regions: Analysis of Rate Increases with Age, 1990-2021.				
**location_name**	**Age**	**Rate**	**CILo**	**CIHi**
Global	17.5	2.280285162	2.015571549	2.579764744
Global	22.5	4.85133261	4.446702907	5.292781771
Global	27.5	8.615294119	8.064810053	9.203352872
Global	32.5	17.47371622	16.64589978	18.34270076
Global	37.5	30.87879934	29.73598875	32.06553032
Global	42.5	56.92064393	55.3120696	58.57599849
Global	47.5	94.22166142	92.02100981	96.47494087
Global	52.5	150.9829597	147.9793621	154.0475226
Global	57.5	225.0531186	221.0259596	229.1536537
Global	62.5	325.9403709	320.4875187	331.4859992
Global	67.5	458.2927898	450.7433219	465.9687033
Global	72.5	650.8167177	638.5008582	663.3701342
Global	77.5	944.9553633	926.4498397	963.8305286
Global	82.5	1473.781042	1443.519602	1504.676873
Global	87.5	2406.784111	2353.196508	2461.592024
Global	92.5	3617.070394	3520.290117	3716.511367
Global	17.5	2.391045577	2.193806095	2.606018355
Global	22.5	3.720007112	3.478306587	3.97850292
Global	27.5	5.048646493	4.774871049	5.338119323
Global	32.5	7.970310028	7.621374646	8.335220992
Global	37.5	12.15428579	11.71645625	12.60847647
Global	42.5	21.33312459	20.73455282	21.94897612
Global	47.5	35.25868828	34.4496815	36.08669356
Global	52.5	57.70518084	56.59962244	58.83233406
Global	57.5	95.99701597	94.4240135	97.59622296
Global	62.5	156.4801886	154.2000685	158.7940244
Global	67.5	248.6526556	245.2242938	252.1289477
Global	72.5	400.5311232	394.3019509	406.8587037
Global	77.5	645.0236345	634.9142458	655.2939894
Global	82.5	1068.662607	1051.649312	1085.951138
Global	87.5	1661.329738	1633.954351	1689.163774
Global	92.5	2506.184014	2461.88488	2551.280266
Global	17.5	2.300140159	2.134475713	2.478662427
Global	22.5	4.272247037	4.04303833	4.514450089
Global	27.5	6.846381816	6.555896346	7.149738417
Global	32.5	12.79230744	12.37879281	13.21963555
Global	37.5	21.66416652	21.10933339	22.23358276
Global	42.5	39.41878092	38.64476851	40.20829595
Global	47.5	65.10848135	64.05612095	66.1781307
Global	52.5	104.4957346	103.0660846	105.9452156
Global	57.5	159.9244655	157.9851482	161.8875885
Global	62.5	239.1341817	236.4663157	241.8321471
Global	67.5	349.0785692	345.2998268	352.8986638
Global	72.5	517.9445148	511.5603413	524.4083615
Global	77.5	782.6444939	772.7981708	792.6162702
Global	82.5	1248.836645	1232.657502	1265.228146

Global	87.5	1965.298165	1938.391134	1992.578696
Global	92.5	2934.143564	2888.983978	2980.00907
High-middle SDI	17.5	3.646145588	2.915799095	4.559428553
High-middle SDI	22.5	7.055090866	6.059166008	8.214712563
High-middle SDI	27.5	12.71190914	11.40517494	14.16836084
High-middle SDI	32.5	25.09865761	23.28229745	27.05672046
High-middle SDI	37.5	45.10833608	42.70881444	47.64267073
High-middle SDI	42.5	80.64289176	77.50354141	83.90940432
High-middle SDI	47.5	124.6464601	120.6624139	128.7620521
High-middle SDI	52.5	186.1069618	181.1002912	191.252046
High-middle SDI	57.5	261.6385713	255.2987313	268.1358489
High-middle SDI	62.5	373.9642547	365.4636577	382.6625735
High-middle SDI	67.5	510.9357182	499.3674878	522.7719356
High-middle SDI	72.5	714.741175	696.5623322	733.3944481
High-middle SDI	77.5	1045.882446	1018.603639	1073.891795
High-middle SDI	82.5	1642.828007	1598.062927	1688.84705
High-middle SDI	87.5	2848.987203	2765.155497	2935.36045
High-middle SDI	92.5	4362.83922	4207.467568	4523.948373
High-middle SDI	17.5	2.350426603	1.802434732	3.065023726
High-middle SDI	22.5	3.83916714	3.169336524	4.650564627
High-middle SDI	27.5	5.081786691	4.368019542	5.912188745
High-middle SDI	32.5	8.103765549	7.238745683	9.072154064
High-middle SDI	37.5	12.98073336	11.92515882	14.12974377
High-middle SDI	42.5	22.16659923	20.83305851	23.58550095
High-middle SDI	47.5	34.00226833	32.36514199	35.72220546
High-middle SDI	52.5	54.29078853	52.20520837	56.4596869
High-middle SDI	57.5	91.0289714	88.13666502	94.01619214
High-middle SDI	62.5	153.6278278	149.3315257	158.0477355
High-middle SDI	67.5	256.5301168	249.7658222	263.4776057
High-middle SDI	72.5	447.9557657	435.0622276	461.2314176
High-middle SDI	77.5	762.3888359	740.4979769	784.9268401
High-middle SDI	82.5	1318.796633	1280.599733	1358.132846
High-middle SDI	87.5	2102.003993	2039.59387	2166.323822
High-middle SDI	92.5	3156.562925	3057.265093	3259.08588
High-middle SDI	17.5	2.928536602	2.372156361	3.61541371
High-middle SDI	22.5	5.375562486	4.644571495	6.221601298
High-middle SDI	27.5	8.901707384	7.994684944	9.911634406
High-middle SDI	32.5	16.70026844	15.48081342	18.0157824
High-middle SDI	37.5	29.23992858	27.66012197	30.90996578
High-middle SDI	42.5	51.63825533	49.5938073	53.7669834
High-middle SDI	47.5	79.36747299	76.80590515	82.01447215
High-middle SDI	52.5	119.4225521	116.2198974	122.7134619
High-middle SDI	57.5	173.9968128	169.8818585	178.2114414
High-middle SDI	62.5	258.7216491	253.0867526	264.4820046
High-middle SDI	67.5	375.0053895	367.0318677	383.1521308
High-middle SDI	72.5	570.682539	557.2669332	584.4211111
High-middle SDI	77.5	894.8789626	873.6701797	916.6025995
High-middle SDI	82.5	1478.290485	1442.517633	1514.950464
High-middle SDI	87.5	2420.037865	2358.738883	2482.929886
High-middle SDI	92.5	3636.96671	3534.341442	3742.571866

Low SDI	17.5	1.62927464	1.340905299	1.979659454
Low SDI	22.5	3.078488954	2.645098105	3.582889506
Low SDI	27.5	5.19027176	4.590253469	5.868721874
Low SDI	32.5	11.7653562	10.73928436	12.88946282
Low SDI	37.5	22.07708615	20.52098912	23.75118128
Low SDI	42.5	44.36928065	41.93771105	46.94183387
Low SDI	47.5	79.2519853	75.65394944	83.02114061
Low SDI	52.5	139.4504367	134.05111	145.0672382
Low SDI	57.5	226.7318802	218.8576947	234.8893677
Low SDI	62.5	349.4638155	338.2366867	361.0636077
Low SDI	67.5	520.7082006	504.3608279	537.5854253
Low SDI	72.5	762.2681839	734.2288561	791.3783002
Low SDI	77.5	1039.95564	998.8517204	1082.751033
Low SDI	82.5	1681.416764	1609.462432	1756.587963
Low SDI	87.5	2370.494989	2248.717471	2498.867272
Low SDI	92.5	3653.770622	3396.523111	3930.501669
Low SDI	17.5	3.008739006	2.657369741	3.406567879
Low SDI	22.5	4.685840621	4.235519739	5.184039664
Low SDI	27.5	5.488588792	5.016024522	6.005673775
Low SDI	32.5	9.100265015	8.444065045	9.807459192
Low SDI	37.5	13.72287337	12.87719915	14.62408489
Low SDI	42.5	25.73986165	24.48446009	27.05963191
Low SDI	47.5	46.59256907	44.74131587	48.52042123
Low SDI	52.5	79.02601868	76.33190337	81.8152221
Low SDI	57.5	136.0041036	131.9943036	140.1357157
Low SDI	62.5	222.0175052	216.1215862	228.0742682
Low SDI	67.5	355.5633378	346.518884	364.8438599
Low SDI	72.5	546.9011103	530.7759596	563.5161484
Low SDI	77.5	817.8792388	792.7092983	843.8483699
Low SDI	82.5	1212.340038	1172.288068	1253.760409
Low SDI	87.5	1596.86371	1535.492142	1660.688218
Low SDI	92.5	2224.18861	2112.186672	2342.129623
Low SDI	17.5	2.158701752	1.869252717	2.492971234
Low SDI	22.5	3.688030772	3.288872239	4.135633733
Low SDI	27.5	5.19334643	4.710221902	5.72602474
Low SDI	32.5	10.30301556	9.54588547	11.12019728
Low SDI	37.5	17.85583205	16.7725789	19.00904686
Low SDI	42.5	35.16276453	33.49455624	36.91405853
Low SDI	47.5	63.15997927	60.69280945	65.72743984
Low SDI	52.5	109.5362551	105.8808218	113.3178888
Low SDI	57.5	181.3645122	176.006886	186.8852238
Low SDI	62.5	285.3811568	277.662391	293.3144975
Low SDI	67.5	437.3570945	425.8926768	449.1301179
Low SDI	72.5	652.406436	632.4653192	672.9762801
Low SDI	77.5	926.750075	896.7390928	957.7654285
Low SDI	82.5	1434.164015	1384.036776	1486.106769
Low SDI	87.5	1949.69914	1868.994048	2033.889162
Low SDI	92.5	2844.88357	2686.577204	3012.518128
High SDI	17.5	1.210394395	0.87615736	1.672136375
High SDI	22.5	2.367903677	1.90673217	2.940616364

High SDI	27.5	4.688858578	4.051545975	5.426421149
High SDI	32.5	10.01875992	9.10480179	11.02446299
High SDI	37.5	20.30397455	19.02645106	21.66727686
High SDI	42.5	40.48636864	38.72424251	42.32867939
High SDI	47.5	68.66236585	66.34269794	71.06314079
High SDI	52.5	105.2834737	102.3132886	108.3398841
High SDI	57.5	145.7977542	142.1139347	149.5770643
High SDI	62.5	193.9633938	189.3583457	198.6804332
High SDI	67.5	248.4162483	242.6286216	254.3419323
High SDI	72.5	323.5091027	315.1905461	332.047204
High SDI	77.5	439.2001585	427.7583021	450.9480664
High SDI	82.5	629.165602	612.4545351	646.332637
High SDI	87.5	937.4154719	911.5343363	964.0314489
High SDI	92.5	1347.741815	1306.567475	1390.213696
High SDI	17.5	0.529659653	0.377197519	0.743746537
High SDI	22.5	0.900529318	0.707022203	1.146997999
High SDI	27.5	1.469384982	1.231323561	1.753472679
High SDI	32.5	2.784873562	2.463464743	3.148216665
High SDI	37.5	5.13041152	4.701993788	5.59786413
High SDI	42.5	9.848789573	9.263870473	10.47064036
High SDI	47.5	16.75428547	15.98986292	17.55525255
High SDI	52.5	26.59327977	25.6104896	27.61378404
High SDI	57.5	40.00833155	38.73735503	41.32100894
High SDI	62.5	61.2672835	59.52031292	63.06552911
High SDI	67.5	89.46207823	87.02979584	91.96233731
High SDI	72.5	136.1291066	132.1510616	140.2268997
High SDI	77.5	211.4586656	205.3216589	217.7791057
High SDI	82.5	343.4637832	333.5173519	353.7068453
High SDI	87.5	557.119021	540.9031378	573.8210447
High SDI	92.5	869.2503198	843.4814984	895.8063928
High SDI	17.5	0.872797238	0.654590664	1.163742565
High SDI	22.5	1.640628235	1.348088485	1.996650098
High SDI	27.5	3.087076742	2.696225993	3.534586062
High SDI	32.5	6.423348079	5.871110125	7.02752966
High SDI	37.5	12.7597863	11.99645825	13.57168449
High SDI	42.5	25.23052898	24.18172401	26.3248225
High SDI	47.5	42.71493416	41.34110172	44.13442129
High SDI	52.5	65.72456176	63.97162938	67.5255275
High SDI	57.5	92.16828869	89.98648439	94.40299282
High SDI	62.5	126.0026847	123.2311271	128.8365767
High SDI	67.5	165.7587377	162.2120391	169.3829834
High SDI	72.5	224.4772593	219.2318938	229.848126
High SDI	77.5	316.735211	309.3136247	324.3348688
High SDI	82.5	473.5509413	462.3858695	484.9856122
High SDI	87.5	727.7670297	710.305706	745.6576021
High SDI	92.5	1088.729867	1061.344093	1116.822273
Low-middle SDI	17.5	2.2590591	1.938669473	2.632397161
Low-middle SDI	22.5	5.346425817	4.802170762	5.952364136
Low-middle SDI	27.5	9.467312677	8.70067982	10.30149496
Low-middle SDI	32.5	20.14632046	18.90278073	21.4716678

Low-middle SDI	37.5	33.83112032	32.11380322	35.64027264
Low-middle SDI	42.5	64.61010177	62.02790357	67.2997959
Low-middle SDI	47.5	114.390999	110.5792105	118.3341841
Low-middle SDI	52.5	195.3567567	189.7321325	201.1481233
Low-middle SDI	57.5	311.6174198	303.5324332	319.9177607
Low-middle SDI	62.5	459.3457881	448.1767289	470.7931927
Low-middle SDI	67.5	663.6805448	647.741566	680.0117341
Low-middle SDI	72.5	955.5335729	928.2281101	983.642274
Low-middle SDI	77.5	1380.437109	1338.393952	1423.800974
Low-middle SDI	82.5	2147.897881	2076.860458	2221.365085
Low-middle SDI	87.5	3109.24421	2989.099549	3234.217998
Low-middle SDI	92.5	4907.058012	4663.931359	5162.858644
Low-middle SDI	17.5	3.908982386	3.470721489	4.402584113
Low-middle SDI	22.5	5.908861127	5.372562621	6.498693879
Low-middle SDI	27.5	8.185740221	7.55348602	8.870916394
Low-middle SDI	32.5	12.79926486	11.97806136	13.67676923
Low-middle SDI	37.5	19.23926473	18.18592139	20.35361858
Low-middle SDI	42.5	34.40919605	32.89796521	35.98984817
Low-middle SDI	47.5	58.19267867	56.06119178	60.40520623
Low-middle SDI	52.5	97.784151	94.70633644	100.9619899
Low-middle SDI	57.5	164.2957003	159.8013431	168.9164598
Low-middle SDI	62.5	264.8294344	258.3591825	271.461725
Low-middle SDI	67.5	415.8003721	406.0737813	425.7599417
Low-middle SDI	72.5	643.7173615	626.0794039	661.8522171
Low-middle SDI	77.5	992.5190951	964.3023705	1021.561477
Low-middle SDI	82.5	1531.581913	1485.553789	1579.036164
Low-middle SDI	87.5	2064.720963	1994.781044	2137.113078
Low-middle SDI	92.5	2864.487217	2744.835645	2989.354584
Low-middle SDI	17.5	2.874526699	2.554230272	3.23498779
Low-middle SDI	22.5	5.484712437	5.019596566	5.99292595
Low-middle SDI	27.5	8.734038971	8.127283416	9.386092849
Low-middle SDI	32.5	16.53888876	15.63348721	17.49672596
Low-middle SDI	37.5	26.7459383	25.52647292	28.02366069
Low-middle SDI	42.5	50.04741941	48.24081823	51.92167716
Low-middle SDI	47.5	87.20027652	84.57709636	89.90481528
Low-middle SDI	52.5	147.520317	143.6942406	151.4482683
Low-middle SDI	57.5	237.9148351	232.4206598	243.5388869
Low-middle SDI	62.5	360.3384862	352.6748567	368.1686465
Low-middle SDI	67.5	536.5363063	525.4053458	547.9030814
Low-middle SDI	72.5	793.8506349	774.3980094	813.791904
Low-middle SDI	77.5	1178.563951	1148.183974	1209.747756
Low-middle SDI	82.5	1821.647616	1771.308682	1873.417135
Low-middle SDI	87.5	2536.408482	2455.783191	2619.680765
Low-middle SDI	92.5	3731.398546	3581.680426	3887.375046
Middle SDI	17.5	2.116059835	1.898669361	2.358340698
Middle SDI	22.5	4.534978217	4.19764354	4.899422077
Middle SDI	27.5	8.177956754	7.711358993	8.672787343
Middle SDI	32.5	15.93560704	15.24009805	16.66285682
Middle SDI	37.5	28.20159807	27.21799575	29.22074574
Middle SDI	42.5	49.80177202	48.42528617	51.2173844

Middle SDI	47.5	80.90119408	79.01027519	82.83736752
Middle SDI	52.5	132.196947	129.5451681	134.9030077
Middle SDI	57.5	203.0293666	199.3617875	206.7644168
Middle SDI	62.5	306.0952478	300.9470331	311.3315315
Middle SDI	67.5	458.7285109	451.196995	466.3857451
Middle SDI	72.5	705.9132295	692.3205625	719.7727679
Middle SDI	77.5	1117.784946	1095.379671	1140.648506
Middle SDI	82.5	1941.407069	1900.250392	1983.45514
Middle SDI	87.5	3730.980727	3644.708173	3819.295407
Middle SDI	92.5	6071.911974	5899.029983	6249.860591
Middle SDI	17.5	2.243549859	1.929294649	2.608992862
Middle SDI	22.5	3.623288263	3.22571546	4.06986233
Middle SDI	27.5	5.110986965	4.649018475	5.61886082
Middle SDI	32.5	8.005457203	7.416356624	8.641351578
Middle SDI	37.5	12.01611887	11.27896792	12.80144724
Middle SDI	42.5	20.503521	19.50298995	21.55538072
Middle SDI	47.5	33.91901212	32.54296731	35.35324151
Middle SDI	52.5	55.85765236	53.950109	57.83264177
Middle SDI	57.5	94.92889618	92.13196339	97.81073798
Middle SDI	62.5	156.0033659	151.904268	160.2130769
Middle SDI	67.5	254.203319	247.9410208	260.6237854
Middle SDI	72.5	415.131749	403.298117	427.3126051
Middle SDI	77.5	693.5651811	673.4574997	714.2732253
Middle SDI	82.5	1239.632588	1202.778927	1277.615461
Middle SDI	87.5	2067.534251	2002.549418	2134.627909
Middle SDI	92.5	3356.027806	3238.213804	3478.128164
Middle SDI	17.5	2.094414851	1.899378831	2.309477972
Middle SDI	22.5	4.012191322	3.733796979	4.311342928
Middle SDI	27.5	6.631195352	6.269408816	7.013859375
Middle SDI	32.5	12.06661994	11.55326717	12.60278281
Middle SDI	37.5	20.3615562	19.66151891	21.08651792
Middle SDI	42.5	35.58682991	34.61719486	36.58362465
Middle SDI	47.5	57.93862018	56.61073545	59.29765232
Middle SDI	52.5	94.44814754	92.60153767	96.33158151
Middle SDI	57.5	148.8667649	146.2797113	151.4995722
Middle SDI	62.5	229.7428948	226.0789742	233.4661943
Middle SDI	67.5	353.2694327	347.8521658	358.7710654
Middle SDI	72.5	553.8692077	543.9682927	563.9503319
Middle SDI	77.5	892.0823449	875.6636686	908.808871
Middle SDI	82.5	1557.21897	1527.384896	1587.635787
Middle SDI	87.5	2747.439146	2690.848938	2805.219482
Middle SDI	92.5	4403.535915	4297.370272	4512.324358

Table S10. Period Effects on Ischemic Heart Disease Mortality Rates: Rate Ratios by Sex and SDI Regions, 1990-2021.

location_name	period	sex	rate.ratio	ci.lo	ci.hi	logratio
Global	1994.5	Male	1.096904118	1.079841703	1.114236133	0.133437423
Global	1999.5	Male	1.03158526	1.017203526	1.046170331	0.044863065
Global	2004.5	Male	1	1	1	0
Global	2009.5	Male	0.944857786	0.93231845	0.957565772	-0.081830894
Global	2014.5	Male	0.904182078	0.891602776	0.916938857	-0.145314772
Global	2019.5	Male	0.857519196	0.844762495	0.870468535	-0.221759128
Global	1994.5	Female	1.112199618	1.099584151	1.124959823	0.153415747
Global	1999.5	Female	1.048912273	1.038863853	1.059057887	0.068894022
Global	2004.5	Female	1	1	1	0
Global	2009.5	Female	0.918988524	0.910487694	0.927568722	-0.12188125
Global	2014.5	Female	0.862492073	0.853632473	0.871443625	-0.213416897
Global	2019.5	Female	0.801510045	0.792154845	0.810975727	-0.319207495
Global	1994.5	Both	1.100515488	1.089912003	1.111222133	0.13817945
Global	1999.5	Both	1.03870979	1.029932685	1.047561694	0.054792629
Global	2004.5	Both	1	1	1	0
Global	2009.5	Both	0.933317487	0.925764124	0.940932479	-0.099560168
Global	2014.5	Both	0.885486719	0.877833099	0.893207068	-0.175457427
Global	2019.5	Both	0.832300297	0.824448572	0.840226798	-0.264823943
High-middle SDI	1994.5	Male	1.11837431	1.094031193	1.143259081	0.161403126
High-middle SDI	1999.5	Male	1.016741316	0.997970393	1.035865303	0.023952668
High-middle SDI	2004.5	Male	1	1	1	0
High-middle SDI	2009.5	Male	0.870546194	0.855106555	0.886264608	-0.200007241
High-middle SDI	2014.5	Male	0.752705484	0.737442761	0.768284097	-0.409842612
High-middle SDI	2019.5	Male	0.661674987	0.6460533	0.677674409	-0.595805352
High-middle SDI	1994.5	Female	1.166854687	1.139396948	1.194974116	0.222624908
High-middle SDI	1999.5	Female	1.059475805	1.041430537	1.077833751	0.083350642
High-middle SDI	2004.5	Female	1	1	1	0
High-middle SDI	2009.5	Female	0.855699152	0.841558362	0.870077552	-0.224824434
High-middle SDI	2014.5	Female	0.719528671	0.703482783	0.735940553	-0.474875919
High-middle SDI	2019.5	Female	0.621591812	0.603419042	0.640311879	-0.685960594
High-middle SDI	1994.5	Both	1.128004863	1.106141254	1.150300622	0.173773288
High-middle SDI	1999.5	Both	1.032112017	1.016051139	1.048426772	0.045599558
High-middle SDI	2004.5	Both	1	1	1	0
High-middle SDI	2009.5	Both	0.866846687	0.853839746	0.880051769	-0.206151238
High-middle SDI	2014.5	Both	0.74166174	0.728231739	0.755339416	-0.431166749
High-middle SDI	2019.5	Both	0.647975548	0.63367216	0.662601794	-0.625988723
Low SDI	1994.5	Male	1.071918258	1.036914086	1.108104102	0.100194894
Low SDI	1999.5	Male	1.055834987	1.024292662	1.088348635	0.078384378
Low SDI	2004.5	Male	1	1	1	0
Low SDI	2009.5	Male	1.000823384	0.972605666	1.029859768	0.001187403
Low SDI	2014.5	Male	1.048388312	1.018832963	1.078801032	0.068173174
Low SDI	2019.5	Male	1.042100508	1.012088315	1.073002675	0.059494429
Low SDI	1994.5	Female	1.142228528	1.113707383	1.171480077	0.191851323
Low SDI	1999.5	Female	1.081722442	1.057051666	1.106969015	0.113330367
Low SDI	2004.5	Female	1	1	1	0
Low SDI	2009.5	Female	0.933429726	0.913161257	0.954148073	-0.099386682

Low SDI	2014.5	Female	0.964571432	0.943587366	0.986022154	-0.052040013
Low SDI	2019.5	Female	0.894093275	0.873908158	0.914744618	-0.161502748
Low SDI	1994.5	Both	1.101655292	1.07236702	1.131743479	0.139672875
Low SDI	1999.5	Both	1.067188512	1.041224458	1.093800007	0.093815041
Low SDI	2004.5	Both	1	1	1	0
Low SDI	2009.5	Both	0.971330764	0.948949644	0.994239747	-0.041965439
Low SDI	2014.5	Both	1.011108825	0.987837384	1.034928494	0.015938282
Low SDI	2019.5	Both	0.974807132	0.951792463	0.998378304	-0.036811288
High SDI	1994.5	Male	1.399256778	1.367447664	1.431805825	0.484660736
High SDI	1999.5	Male	1.186710628	1.166052711	1.207734523	0.246968186
High SDI	2004.5	Male	1	1	1	0
High SDI	2009.5	Male	0.847229848	0.83223943	0.862490276	-0.239174678
High SDI	2014.5	Male	0.758298996	0.74130776	0.775679682	-0.399161281
High SDI	2019.5	Male	0.705789638	0.685965364	0.726186831	-0.502689844
High SDI	1994.5	Female	1.37380099	1.342055875	1.406297007	0.458173029
High SDI	1999.5	Female	1.184439151	1.166575104	1.202576754	0.244204083
High SDI	2004.5	Female	1	1	1	0
High SDI	2009.5	Female	0.833587069	0.820731961	0.846643526	-0.262595197
High SDI	2014.5	Female	0.737530264	0.720683107	0.754771251	-0.439225844
High SDI	2019.5	Female	0.684156474	0.66291478	0.706078813	-0.547601773
High SDI	1994.5	Both	1.37506733	1.348010867	1.402666854	0.459502262
High SDI	1999.5	Both	1.181197239	1.164420758	1.198215428	0.240249889
High SDI	2004.5	Both	1	1	1	0
High SDI	2009.5	Both	0.842961495	0.830734412	0.855368541	-0.246461362
High SDI	2014.5	Both	0.751507704	0.736918613	0.766385621	-0.412140202
High SDI	2019.5	Both	0.698323522	0.680775806	0.716323549	-0.518032526
Low-middle SDI	1994.5	Male	1.008003479	0.982640615	1.034020982	0.011500618
Low-middle SDI	1999.5	Male	1.00617135	0.9829728	1.029917396	0.008876016
Low-middle SDI	2004.5	Male	1	1	1	0
Low-middle SDI	2009.5	Male	1.041697932	1.019546245	1.064330908	0.05893699
Low-middle SDI	2014.5	Male	1.068434204	1.04572314	1.091638508	0.095498067
Low-middle SDI	2019.5	Male	1.056383356	1.033494408	1.079779228	0.079133476
Low-middle SDI	1994.5	Female	1.130837602	1.104644645	1.157651637	0.17739176
Low-middle SDI	1999.5	Female	1.067380732	1.044790254	1.090459661	0.094074873
Low-middle SDI	2004.5	Female	1	1	1	0
Low-middle SDI	2009.5	Female	0.975635793	0.956337212	0.995323814	-0.035585408
Low-middle SDI	2014.5	Female	0.994942599	0.975160675	1.015125816	-0.0073148
Low-middle SDI	2019.5	Female	0.958263223	0.938493837	0.978449051	-0.061506095
Low-middle SDI	1994.5	Both	1.057380468	1.034927699	1.08032035	0.080494582
Low-middle SDI	1999.5	Both	1.031649741	1.011583048	1.052114496	0.04495324
Low-middle SDI	2004.5	Both	1	1	1	0
Low-middle SDI	2009.5	Both	1.012845973	0.994580937	1.031446438	0.018414795
Low-middle SDI	2014.5	Both	1.034707669	1.016048531	1.05370947	0.049223227
Low-middle SDI	2019.5	Both	1.010452817	0.991789564	1.02946727	0.015001957
Middle SDI	1994.5	Male	1.003658913	0.986994556	1.02060463	0.005269062
Middle SDI	1999.5	Male	0.98404448	0.969250718	0.999064041	-0.023204566
Middle SDI	2004.5	Male	1	1	1	0
Middle SDI	2009.5	Male	0.999447293	0.985732351	1.013353058	-0.000797608
Middle SDI	2014.5	Male	1.001774368	0.987826883	1.015918782	0.002557603
Middle SDI	2019.5	Male	0.970682168	0.956582252	0.984989916	-0.042929105

Middle SDI	1994.5	Female	1.098433431	1.073272503	1.124184213	0.135447441
Middle SDI	1999.5	Female	1.024194534	1.003555173	1.04525837	0.034489765
Middle SDI	2004.5	Female	1	1	1	0
Middle SDI	2009.5	Female	0.953963726	0.936215586	0.972048323	-0.067993686
Middle SDI	2014.5	Female	0.925338946	0.907126889	0.94391664	-0.111946182
Middle SDI	2019.5	Female	0.872190826	0.853376286	0.891420174	-0.197284279
Middle SDI	1994.5	Both	1.037998952	1.022453195	1.053781074	0.053804988
Middle SDI	1999.5	Both	0.999369049	0.985947862	1.012972931	-0.000910557
Middle SDI	2004.5	Both	1	1	1	0
Middle SDI	2009.5	Both	0.981179603	0.969102129	0.993407593	-0.027410852
Middle SDI	2014.5	Both	0.970785489	0.958506581	0.983221696	-0.042775551
Middle SDI	2019.5	Both	0.929917095	0.917453211	0.942550305	-0.104825994

Table S11. Cohort Effects on Ischemic Heart Disease Mortality Rates by Sex Across SDI Regions: Relative Risks Across Birth Cohorts, 1902-2002.

Global	1902	Male	1.694752773	1.591659153	1.804523888	0.761074832
Global	1907	Male	1.616097305	1.557604204	1.676787011	0.692514065
Global	1912	Male	1.602672536	1.557914535	1.648716409	0.680479679
Global	1917	Male	1.49832652	1.46139299	1.536193465	0.583352055
Global	1922	Male	1.440029483	1.408072571	1.472711674	0.52609835
Global	1927	Male	1.400890005	1.372554439	1.429810542	0.486343683
Global	1932	Male	1.314342996	1.289100713	1.340079557	0.394341816
Global	1937	Male	1.227719435	1.204882089	1.25098964	0.295980906
Global	1942	Male	1.116125856	1.095477701	1.137163198	0.158499716
Global	1947	Male	1.026740691	1.007792673	1.046044961	0.038071868
Global	1952	Male	1	1	1	0
Global	1957	Male	0.970432228	0.950684848	0.990589796	-0.043300631
Global	1962	Male	0.91087026	0.889578943	0.932671168	-0.134682516
Global	1967	Male	0.839365106	0.815943371	0.863459164	-0.252629606
Global	1972	Male	0.813475483	0.78529076	0.842671777	-0.29782923
Global	1977	Male	0.825525968	0.789267192	0.863450465	-0.276614497
Global	1982	Male	0.806709772	0.760485784	0.855743355	-0.309878364
Global	1987	Male	0.795203582	0.735906824	0.859278261	-0.33060384
Global	1992	Male	0.777482819	0.696596342	0.867761567	-0.363117301
Global	1997	Male	0.769766987	0.656397726	0.902716738	-0.377506296
Global	2002	Male	0.707354066	0.535274677	0.934753308	-0.499495558
Global	1902	Female	2.344611635	2.276712963	2.414535257	1.229348972
Global	1907	Female	2.141993004	2.097628074	2.187296255	1.098953768
Global	1912	Female	1.961269339	1.925451757	1.997753206	0.971787673
Global	1917	Female	1.718312235	1.688221209	1.748939606	0.780992213
Global	1922	Female	1.591106588	1.564445813	1.618221707	0.670030485
Global	1927	Female	1.528236511	1.503845843	1.553022768	0.611867833
Global	1932	Female	1.395693967	1.373932713	1.41779989	0.480982637
Global	1937	Female	1.29322376	1.273334205	1.31342399	0.370971919
Global	1942	Female	1.166546907	1.148391048	1.184989807	0.22224432
Global	1947	Female	1.04515394	1.028526747	1.06204993	0.063715452
Global	1952	Female	1	1	1	0
Global	1957	Female	0.949368029	0.931514464	0.967563777	-0.074960629
Global	1962	Female	0.92240922	0.901952952	0.943329436	-0.116521161
Global	1967	Female	0.848920172	0.825843038	0.872642168	-0.236299198
Global	1972	Female	0.830819051	0.802884539	0.859725478	-0.267393798
Global	1977	Female	0.806162923	0.772086592	0.841743225	-0.310856662
Global	1982	Female	0.752451695	0.712285839	0.794882506	-0.410329127
Global	1987	Female	0.695564146	0.649584588	0.744798276	-0.523744527
Global	1992	Female	0.650549296	0.594789643	0.711536241	-0.620269712
Global	1997	Female	0.623858782	0.552964949	0.703841681	-0.680708601
Global	2002	Female	0.578012422	0.47739192	0.699840836	-0.790827596
Global	1902	Both	2.024464393	1.964623547	2.086127943	1.017540269
Global	1907	Both	1.871009097	1.834833356	1.907898083	0.903816573
Global	1912	Both	1.761265845	1.732999784	1.789992939	0.816612686
Global	1917	Both	1.584457426	1.560901992	1.608368334	0.663988896
Global	1922	Both	1.49128748	1.470657234	1.512207125	0.576558397

Global	1927	Both	1.44541086	1.426755604	1.464310039	0.531479639
Global	1932	Both	1.342827189	1.326129184	1.359735448	0.425273654
Global	1937	Both	1.250263764	1.235073854	1.26564049	0.322232487
Global	1942	Both	1.135382789	1.121555178	1.14938088	0.183178777
Global	1947	Both	1.035089865	1.02239306	1.047944347	0.049756025
Global	1952	Both	1	1	1	0
Global	1957	Both	0.963615547	0.950281665	0.977136524	-0.053470424
Global	1962	Both	0.912242018	0.897624138	0.927097952	-0.132511472
Global	1967	Both	0.839640217	0.823447312	0.856151551	-0.252156825
Global	1972	Both	0.816350028	0.796800589	0.83637911	-0.292740224
Global	1977	Both	0.817706826	0.792905987	0.843283396	-0.290344411
Global	1982	Both	0.788196871	0.757251148	0.820407218	-0.343372072
Global	1987	Both	0.759905987	0.721604214	0.800240768	-0.39610715
Global	1992	Both	0.728856888	0.678695781	0.782725307	-0.456292527
Global	1997	Both	0.710093162	0.642196388	0.785168381	-0.493919782
Global	2002	Both	0.652729227	0.551345521	0.772755789	-0.615443456
High-middle SDI	1902	Male	2.046788795	1.87777343	2.231016961	1.033362241
High-middle SDI	1907	Male	1.990185127	1.895459316	2.089644872	0.992902636
High-middle SDI	1912	Male	1.953082947	1.881510741	2.027377743	0.965753222
High-middle SDI	1917	Male	1.764536762	1.706443248	1.824607991	0.819289488
High-middle SDI	1922	Male	1.667999413	1.618730461	1.718767953	0.738118781
High-middle SDI	1927	Male	1.70510456	1.659654866	1.751798896	0.769860211
High-middle SDI	1932	Male	1.55067583	1.511130571	1.591255962	0.632897122
High-middle SDI	1937	Male	1.458889999	1.422913586	1.495776026	0.544871107
High-middle SDI	1942	Male	1.24146339	1.210368999	1.273356597	0.312041719
High-middle SDI	1947	Male	1.047149465	1.020602889	1.074386535	0.06646738
High-middle SDI	1952	Male	1	1	1	0
High-middle SDI	1957	Male	0.90326248	0.877982742	0.929270096	-0.146782813
High-middle SDI	1962	Male	0.759361533	0.734166035	0.785421703	-0.397141178
High-middle SDI	1967	Male	0.609732451	0.584665622	0.63587399	-0.713751765
High-middle SDI	1972	Male	0.539450059	0.510826294	0.569677736	-0.890438689
High-middle SDI	1977	Male	0.523078865	0.48644936	0.562466561	-0.934899617
High-middle SDI	1982	Male	0.488777173	0.442418358	0.539993696	-1.032751186
High-middle SDI	1987	Male	0.449604799	0.392301833	0.515277926	-1.153270661
High-middle SDI	1992	Male	0.411079774	0.332578469	0.508110404	-1.282509706
High-middle SDI	1997	Male	0.393664427	0.280023008	0.553424813	-1.344961745
High-middle SDI	2002	Male	0.339094657	0.178891701	0.64276423	-1.560240043
High-middle SDI	1902	Female	2.718296474	2.585193596	2.858252369	1.442702814
High-middle SDI	1907	Female	2.534337291	2.444155248	2.627846782	1.341608543
High-middle SDI	1912	Female	2.357753441	2.282363568	2.435633553	1.237412858
High-middle SDI	1917	Female	2.047822923	1.983965996	2.113735182	1.03409097
High-middle SDI	1922	Female	1.915519123	1.857745094	1.975089867	0.937735428
High-middle SDI	1927	Female	1.901551711	1.846399697	1.958351118	0.927177172
High-middle SDI	1932	Female	1.680198403	1.63207195	1.729744007	0.748631601
High-middle SDI	1937	Female	1.547409846	1.50349422	1.592608205	0.629855358
High-middle SDI	1942	Female	1.327464769	1.288726113	1.367367896	0.408673573
High-middle SDI	1947	Female	1.068792832	1.036081363	1.102537077	0.095982237
High-middle SDI	1952	Female	1	1	1	0
High-middle SDI	1957	Female	0.871674874	0.838692758	0.905954032	-0.198137971
High-middle SDI	1962	Female	0.730248169	0.695345499	0.766902769	-0.453541259

High-middle SDI	1967	Female	0.605862699	0.568463357	0.645722553	-0.722937209
High-middle SDI	1972	Female	0.530751488	0.487846922	0.577429372	-0.913891584
High-middle SDI	1977	Female	0.477490944	0.426246322	0.53489635	-1.066454723
High-middle SDI	1982	Female	0.425894613	0.365449098	0.496337855	-1.231431612
High-middle SDI	1987	Female	0.379371537	0.310834125	0.463021114	-1.398316655
High-middle SDI	1992	Female	0.330507668	0.2470899	0.442087348	-1.597244352
High-middle SDI	1997	Female	0.297318981	0.191028253	0.462751319	-1.749916528
High-middle SDI	2002	Female	0.257359575	0.117249574	0.564897159	-1.958142635
High-middle SDI	1902	Both	2.383950484	2.257378145	2.517619798	1.25335427
High-middle SDI	1907	Both	2.247443144	2.16995456	2.327698827	1.168284619
High-middle SDI	1912	Both	2.124779934	2.063274372	2.188118957	1.087313427
High-middle SDI	1917	Both	1.868749519	1.817550622	1.921390646	0.902073208
High-middle SDI	1922	Both	1.753859675	1.708780929	1.800127626	0.810533323
High-middle SDI	1927	Both	1.764540731	1.722055304	1.80807433	0.819292732
High-middle SDI	1932	Both	1.587341211	1.550090146	1.625487477	0.66661228
High-middle SDI	1937	Both	1.480093413	1.44607363	1.514913533	0.565688231
High-middle SDI	1942	Both	1.268849559	1.239024206	1.299392858	0.343521027
High-middle SDI	1947	Both	1.055313461	1.029835424	1.081421824	0.077671589
High-middle SDI	1952	Both	1	1	1	0
High-middle SDI	1957	Both	0.896375835	0.871671135	0.92178071	-0.157824338
High-middle SDI	1962	Both	0.753500816	0.728489356	0.779371003	-0.408319021
High-middle SDI	1967	Both	0.610280054	0.58492367	0.636735636	-0.712456655
High-middle SDI	1972	Both	0.539060862	0.510026311	0.569748278	-0.891479928
High-middle SDI	1977	Both	0.514385095	0.477727163	0.553855939	-0.959079255
High-middle SDI	1982	Both	0.476435803	0.430623984	0.527121299	-1.069646263
High-middle SDI	1987	Both	0.435034347	0.379442248	0.498771247	-1.200798784
High-middle SDI	1992	Both	0.394092576	0.31971985	0.485765768	-1.343393523
High-middle SDI	1997	Both	0.371246822	0.266610428	0.516949784	-1.429549418
High-middle SDI	2002	Both	0.320125916	0.173631476	0.590219036	-1.643288619
Low SDI	1902	Male	0.704760371	0.561438841	0.884668364	-0.504795291
Low SDI	1907	Male	0.767954087	0.684761939	0.861253301	-0.380908034
Low SDI	1912	Male	0.843046722	0.782369689	0.90842959	-0.246315507
Low SDI	1917	Male	0.922704437	0.871061073	0.977409627	-0.116059501
Low SDI	1922	Male	1.00943937	0.961634101	1.05962116	0.013554261
Low SDI	1927	Male	1.065442942	1.020966014	1.111857443	0.091453334
Low SDI	1932	Male	1.096690627	1.054598604	1.140462661	0.133156603
Low SDI	1937	Male	1.098342902	1.058165144	1.140046181	0.135328533
Low SDI	1942	Male	1.07861923	1.040241764	1.11841255	0.109185661
Low SDI	1947	Male	1.052981826	1.015909963	1.091406489	0.074480536
Low SDI	1952	Male	1	1	1	0
Low SDI	1957	Male	0.966009546	0.9278819	1.005703896	-0.04989065
Low SDI	1962	Male	0.959914955	0.917003414	1.004834559	-0.059021501
Low SDI	1967	Male	0.926823538	0.878170911	0.97817163	-0.10963341
Low SDI	1972	Male	0.959424445	0.899499052	1.02334212	-0.059758896
Low SDI	1977	Male	0.949555162	0.876236321	1.029008936	-0.074676283
Low SDI	1982	Male	0.898874227	0.809344427	0.998307825	-0.153808832
Low SDI	1987	Male	0.897499337	0.783795368	1.027698162	-0.156017222
Low SDI	1992	Male	0.893031024	0.745579482	1.069643718	-0.1632178
Low SDI	1997	Male	0.873037572	0.689711753	1.105091509	-0.195884352
Low SDI	2002	Male	0.818679887	0.57006268	1.175724672	-0.288628642

Low SDI	1902	Female	1.170452209	1.020160778	1.342884772	0.227066029
Low SDI	1907	Female	1.179584656	1.095398169	1.270241269	0.238278961
Low SDI	1912	Female	1.163735093	1.105595615	1.224931926	0.218762687
Low SDI	1917	Female	1.160838492	1.113584934	1.210097195	0.215167263
Low SDI	1922	Female	1.203404421	1.160089598	1.24833651	0.267121562
Low SDI	1927	Female	1.217561006	1.177640181	1.258835108	0.283994061
Low SDI	1932	Female	1.216440917	1.179168639	1.254891332	0.282666249
Low SDI	1937	Female	1.187208968	1.15234527	1.223127451	0.247573895
Low SDI	1942	Female	1.153150674	1.119910021	1.187377962	0.205581032
Low SDI	1947	Female	1.079864902	1.04857936	1.112083884	0.110850834
Low SDI	1952	Female	1	1	1	0
Low SDI	1957	Female	0.923849481	0.892606655	0.956185862	-0.114270276
Low SDI	1962	Female	0.900227436	0.86506788	0.936816007	-0.15163856
Low SDI	1967	Female	0.8298051	0.790885908	0.870639491	-0.269155571
Low SDI	1972	Female	0.799687664	0.754933409	0.847095059	-0.322491461
Low SDI	1977	Female	0.742438449	0.691770213	0.796817845	-0.429656669
Low SDI	1982	Female	0.689550249	0.63228122	0.752006435	-0.536272407
Low SDI	1987	Female	0.646682357	0.582788768	0.717580869	-0.628870845
Low SDI	1992	Female	0.570652651	0.501695947	0.649087261	-0.809315233
Low SDI	1997	Female	0.526997245	0.45029191	0.616769012	-0.924132674
Low SDI	2002	Female	0.49620189	0.394475891	0.624160618	-1.011000864
Low SDI	1902	Both	0.910276201	0.771961402	1.073373306	-0.135623733
Low SDI	1907	Both	0.950832706	0.872535967	1.036155378	-0.072736566
Low SDI	1912	Both	0.98635725	0.931118098	1.044873498	-0.019817823
Low SDI	1917	Both	1.029614204	0.983811355	1.077549474	0.042103861
Low SDI	1922	Both	1.096614865	1.054482799	1.140430327	0.133056936
Low SDI	1927	Both	1.135511504	1.096466702	1.175946678	0.183342322
Low SDI	1932	Both	1.151147516	1.1144967	1.189003614	0.203072723
Low SDI	1937	Both	1.136169972	1.101611389	1.17181269	0.18417868
Low SDI	1942	Both	1.11013045	1.077152892	1.144117631	0.150729216
Low SDI	1947	Both	1.065924383	1.03438321	1.098427332	0.092105096
Low SDI	1952	Both	1	1	1	0
Low SDI	1957	Both	0.949823373	0.917819179	0.982943549	-0.074268836
Low SDI	1962	Both	0.937516323	0.901477949	0.974995403	-0.093084285
Low SDI	1967	Both	0.891567493	0.850993496	0.934075992	-0.165584079
Low SDI	1972	Both	0.900049556	0.851251102	0.95164541	-0.151923658
Low SDI	1977	Both	0.868609207	0.810632622	0.930732288	-0.203220849
Low SDI	1982	Both	0.81375652	0.745159375	0.888668513	-0.297330897
Low SDI	1987	Both	0.789568875	0.707182568	0.881553134	-0.340862975
Low SDI	1992	Both	0.739049974	0.641300268	0.851699105	-0.436256174
Low SDI	1997	Both	0.698090646	0.583475752	0.835219885	-0.518513714
Low SDI	2002	Both	0.653997439	0.499838303	0.855702029	-0.61264311
High SDI	1902	Male	4.968880935	4.701381181	5.251600923	2.312920972
High SDI	1907	Male	4.471881436	4.309163047	4.640744237	2.160881938
High SDI	1912	Male	4.114075624	3.987210789	4.244977036	2.040568313
High SDI	1917	Male	3.56433804	3.461305305	3.670437751	1.833634168
High SDI	1922	Male	3.033746907	2.95072833	3.119101208	1.601100733
High SDI	1927	Male	2.473700067	2.409029185	2.540107052	1.306670586
High SDI	1932	Male	2.011695586	1.960259214	2.064481627	1.00841201
High SDI	1937	Male	1.581023705	1.54076434	1.622335026	0.660858999

High SDI	1942	Male	1.30875091	1.275191667	1.343193333	0.38819054
High SDI	1947	Male	1.10246566	1.073941822	1.131747089	0.140733718
High SDI	1952	Male	1	1	1	0
High SDI	1957	Male	0.893856404	0.867140927	0.92139495	-0.16188501
High SDI	1962	Male	0.767482025	0.740244401	0.79572187	-0.381795133
High SDI	1967	Male	0.656040307	0.626499208	0.686974346	-0.608143639
High SDI	1972	Male	0.586160782	0.551234784	0.623299677	-0.770631649
High SDI	1977	Male	0.558453373	0.51295743	0.607984507	-0.840491264
High SDI	1982	Male	0.550313279	0.487955287	0.620640279	-0.861674953
High SDI	1987	Male	0.53834929	0.451574428	0.641798871	-0.893385573
High SDI	1992	Male	0.470596022	0.355439288	0.623061725	-1.08743897
High SDI	1997	Male	0.379990121	0.235608823	0.612848409	-1.395966182
High SDI	2002	Male	0.285120735	0.110769618	0.733900097	-1.810355133
High SDI	1902	Female	6.328362041	6.101744878	6.563395704	2.661832138
High SDI	1907	Female	5.683889453	5.506362674	5.867139748	2.506878495
High SDI	1912	Female	5.099300285	4.946282599	5.25705171	2.350299297
High SDI	1917	Female	4.269116293	4.142119597	4.400006688	2.093937463
High SDI	1922	Female	3.542058572	3.437932906	3.649337923	1.824588069
High SDI	1927	Female	2.862610236	2.779894945	2.947786706	1.517331251
High SDI	1932	Female	2.252617892	2.187707715	2.319453979	1.171602612
High SDI	1937	Female	1.720835317	1.670735462	1.772437501	0.783109039
High SDI	1942	Female	1.372778647	1.331743883	1.415077808	0.457099018
High SDI	1947	Female	1.129528691	1.094571593	1.165602205	0.175720916
High SDI	1952	Female	1	1	1	0
High SDI	1957	Female	0.92321503	0.88829904	0.959503449	-0.115261383
High SDI	1962	Female	0.832590676	0.794226491	0.872807998	-0.264320693
High SDI	1967	Female	0.733465715	0.689826673	0.779865401	-0.447198564
High SDI	1972	Female	0.669090168	0.616354307	0.726338159	-0.579727451
High SDI	1977	Female	0.637593202	0.569474386	0.713860187	-0.649291847
High SDI	1982	Female	0.622232079	0.532532265	0.727040943	-0.684475321
High SDI	1987	Female	0.593553083	0.476886993	0.738760476	-0.752551035
High SDI	1992	Female	0.503131863	0.361077063	0.70107381	-0.990991536
High SDI	1997	Female	0.408544292	0.240993466	0.69258491	-1.291435601
High SDI	2002	Female	0.309452045	0.114986784	0.832796299	-1.692212238
High SDI	1902	Both	5.312887458	5.121098111	5.511859473	2.409496153
High SDI	1907	Both	4.775713721	4.641784199	4.913507514	2.255716357
High SDI	1912	Both	4.324217048	4.213462238	4.437883152	2.112438939
High SDI	1917	Both	3.679224232	3.587729374	3.773052407	1.879401605
High SDI	1922	Both	3.092886899	3.018271365	3.169347025	1.628954078
High SDI	1927	Both	2.536017288	2.476658338	2.596798914	1.34256458
High SDI	1932	Both	2.051962847	2.004498936	2.100550641	1.03700461
High SDI	1937	Both	1.606525671	1.569253766	1.644682835	0.683944035
High SDI	1942	Both	1.320944134	1.289844015	1.352794123	0.401569452
High SDI	1947	Both	1.109477405	1.082906413	1.136700362	0.149880286
High SDI	1952	Both	1	1	1	0
High SDI	1957	Both	0.902039684	0.876728348	0.928081763	-0.148737191
High SDI	1962	Both	0.784810713	0.758505555	0.81202814	-0.34958336
High SDI	1967	Both	0.67707248	0.648151545	0.707283885	-0.562617814
High SDI	1972	Both	0.60951396	0.575019548	0.646077632	-0.71426883
High SDI	1977	Both	0.583127005	0.538021639	0.632013807	-0.778117958

High SDI	1982	Both	0.574254443	0.512756722	0.643127921	-0.800237982
High SDI	1987	Both	0.560238137	0.475469478	0.660119703	-0.835887899
High SDI	1992	Both	0.485351412	0.374756259	0.628584548	-1.042898404
High SDI	1997	Both	0.392871258	0.254955601	0.605390999	-1.347871468
High SDI	2002	Both	0.295943608	0.127492084	0.686965156	-1.756605797
Low-middle SDI	1902	Male	0.668469054	0.580481867	0.769792998	-0.58106732
Low-middle SDI	1907	Male	0.700992097	0.648453671	0.757787244	-0.512529915
Low-middle SDI	1912	Male	0.766301923	0.725392527	0.809518455	-0.384015168
Low-middle SDI	1917	Male	0.831892344	0.795950433	0.869457247	-0.265531255
Low-middle SDI	1922	Male	0.901393844	0.868025779	0.936044623	-0.149770497
Low-middle SDI	1927	Male	0.929269866	0.898972303	0.960588531	-0.10583047
Low-middle SDI	1932	Male	0.964466533	0.935654983	0.994165275	-0.052196917
Low-middle SDI	1937	Male	0.967022859	0.939617317	0.99522773	-0.048378102
Low-middle SDI	1942	Male	0.971313914	0.944756352	0.998618022	-0.041990467
Low-middle SDI	1947	Male	0.982676837	0.956297385	1.009783966	-0.025211044
Low-middle SDI	1952	Male	1	1	1	0
Low-middle SDI	1957	Male	1.026559987	0.996837769	1.057168419	0.037817934
Low-middle SDI	1962	Male	1.045875572	1.011864924	1.081029381	0.064711224
Low-middle SDI	1967	Male	1.016059667	0.977083774	1.056590309	0.022985125
Low-middle SDI	1972	Male	1.017356348	0.970109934	1.066903764	0.024825099
Low-middle SDI	1977	Male	1.053490787	0.993567138	1.117028529	0.075177698
Low-middle SDI	1982	Male	1.008382839	0.935385315	1.087077094	0.012043471
Low-middle SDI	1987	Male	0.999428645	0.907832221	1.100266759	-0.000824527
Low-middle SDI	1992	Male	0.968497533	0.848494403	1.105472786	-0.046179722
Low-middle SDI	1997	Male	0.960280468	0.798081909	1.155443528	-0.058472262
Low-middle SDI	2002	Male	0.930945142	0.675510005	1.282969684	-0.103231938
Low-middle SDI	1902	Female	1.111104824	1.000036992	1.234508263	0.15199493
Low-middle SDI	1907	Female	1.042738874	0.981027046	1.108332705	0.060377919
Low-middle SDI	1912	Female	1.037609559	0.99183158	1.08550042	0.053263676
Low-middle SDI	1917	Female	1.088347371	1.04808401	1.130157497	0.1221391
Low-middle SDI	1922	Female	1.111661974	1.074773246	1.14981681	0.152718171
Low-middle SDI	1927	Female	1.107385169	1.073852175	1.141965293	0.147157106
Low-middle SDI	1932	Female	1.108325853	1.076930802	1.140636144	0.148382103
Low-middle SDI	1937	Female	1.067484129	1.038399916	1.097382953	0.09421462
Low-middle SDI	1942	Female	1.043573877	1.015604352	1.072313676	0.061532735
Low-middle SDI	1947	Female	1.015639469	0.988386784	1.04364359	0.022388367
Low-middle SDI	1952	Female	1	1	1	0
Low-middle SDI	1957	Female	0.960212315	0.930760508	0.990596058	-0.058574656
Low-middle SDI	1962	Female	0.989984687	0.95522563	1.026008567	-0.014521886
Low-middle SDI	1967	Female	0.918072742	0.879325364	0.958527519	-0.123319628
Low-middle SDI	1972	Female	0.888574306	0.843546917	0.936005196	-0.170435669
Low-middle SDI	1977	Female	0.850025451	0.797731531	0.905747409	-0.234422057
Low-middle SDI	1982	Female	0.778115681	0.719109234	0.841963897	-0.361943441
Low-middle SDI	1987	Female	0.709438374	0.64389053	0.781658966	-0.495250727
Low-middle SDI	1992	Female	0.625104155	0.55215117	0.707696056	-0.677831502
Low-middle SDI	1997	Female	0.561841656	0.476766969	0.66209714	-0.831764501
Low-middle SDI	2002	Female	0.520012154	0.403287763	0.670520321	-0.943382752
Low-middle SDI	1902	Both	0.867582384	0.778960103	0.966287221	-0.204927335
Low-middle SDI	1907	Both	0.856391168	0.80585467	0.910096893	-0.223658178
Low-middle SDI	1912	Both	0.889720864	0.851602478	0.92954546	-0.16857531

Low-middle SDI	1917	Both	0.946591365	0.913286599	0.981110653	-0.079186334
Low-middle SDI	1922	Both	0.99332286	0.962741584	1.024875544	-0.009665381
Low-middle SDI	1927	Both	1.006922885	0.97916594	1.03546667	0.009953198
Low-middle SDI	1932	Both	1.025204921	0.99907114	1.052022311	0.035912309
Low-middle SDI	1937	Both	1.006314547	0.981827386	1.031412427	0.009081323
Low-middle SDI	1942	Both	1.00041591	0.976726224	1.02468017	0.000599907
Low-middle SDI	1947	Both	0.997051282	0.973682487	1.020980937	-0.004260386
Low-middle SDI	1952	Both	1	1	1	0
Low-middle SDI	1957	Both	1.001157373	0.97530897	1.02769083	0.00166877
Low-middle SDI	1962	Both	1.021785097	0.991959541	1.052507427	0.031091799
Low-middle SDI	1967	Both	0.974828648	0.941044086	1.009826114	-0.036779446
Low-middle SDI	1972	Both	0.962651396	0.922346815	1.004717201	-0.054914644
Low-middle SDI	1977	Both	0.9700496	0.92042214	1.022352881	-0.043869579
Low-middle SDI	1982	Both	0.914151008	0.855242903	0.977116633	-0.129495592
Low-middle SDI	1987	Both	0.876340996	0.805789577	0.95306959	-0.190435744
Low-middle SDI	1992	Both	0.811541927	0.725409023	0.907901996	-0.301262463
Low-middle SDI	1997	Both	0.76717176	0.658161777	0.894236842	-0.382378481
Low-middle SDI	2002	Both	0.721176755	0.560368241	0.928132385	-0.471575197
Middle SDI	1902	Male	0.87069362	0.805745131	0.940877394	-0.199762943
Middle SDI	1907	Male	0.859858237	0.822604428	0.898799184	-0.217829269
Middle SDI	1912	Male	0.937706826	0.907425467	0.968998693	-0.09279116
Middle SDI	1917	Male	0.997695197	0.9709582	1.025168443	-0.003328966
Middle SDI	1922	Male	1.045556713	1.02083247	1.070879769	0.064271319
Middle SDI	1927	Male	1.077746864	1.054841786	1.101149308	0.108018364
Middle SDI	1932	Male	1.094544808	1.072847671	1.116680744	0.130331016
Middle SDI	1937	Male	1.080300012	1.059733673	1.101265484	0.111432022
Middle SDI	1942	Male	1.045459143	1.025908417	1.065382448	0.064136682
Middle SDI	1947	Male	1.016548194	0.997783133	1.035666165	0.023678615
Middle SDI	1952	Male	1	1	1	0
Middle SDI	1957	Male	1.020182528	0.999613462	1.041174843	0.028827297
Middle SDI	1962	Male	0.990580398	0.967871553	1.013822052	-0.013654023
Middle SDI	1967	Male	0.960118927	0.934538853	0.986399176	-0.058714975
Middle SDI	1972	Male	0.951227137	0.920129619	0.983375655	-0.072138221
Middle SDI	1977	Male	0.97279433	0.932887869	1.014407884	-0.039793274
Middle SDI	1982	Male	0.983359798	0.931959778	1.037594664	-0.024208719
Middle SDI	1987	Male	0.97748093	0.912055048	1.04760011	-0.032859538
Middle SDI	1992	Male	0.983105366	0.892412871	1.083014591	-0.024582047
Middle SDI	1997	Male	0.991819998	0.860283929	1.143467727	-0.01184978
Middle SDI	2002	Male	0.900857179	0.698975866	1.161046749	-0.150629695
Middle SDI	1902	Female	1.336004613	1.236188442	1.443880453	0.41792499
Middle SDI	1907	Female	1.298990556	1.237703063	1.363312828	0.377390942
Middle SDI	1912	Female	1.270239305	1.221458456	1.320968293	0.345100317
Middle SDI	1917	Female	1.265823992	1.222087337	1.311125915	0.340076817
Middle SDI	1922	Female	1.278290254	1.237047771	1.320907737	0.354215457
Middle SDI	1927	Female	1.273787882	1.235433006	1.313333513	0.349125053
Middle SDI	1932	Female	1.260012446	1.223691803	1.297411131	0.333437985
Middle SDI	1937	Female	1.216085493	1.181840028	1.251323269	0.282244657
Middle SDI	1942	Female	1.13892135	1.10676844	1.172008339	0.187668123
Middle SDI	1947	Female	1.064215883	1.033935926	1.095382623	0.089790841
Middle SDI	1952	Female	1	1	1	0

Middle SDI	1957	Female	0.973121198	0.940833919	1.006516503	-0.039308597
Middle SDI	1962	Female	0.93997138	0.903620475	0.97778461	-0.089311265
Middle SDI	1967	Female	0.861377734	0.821287622	0.903424794	-0.215282063
Middle SDI	1972	Female	0.852887822	0.804065737	0.904674337	-0.229572094
Middle SDI	1977	Female	0.817945354	0.758955927	0.881519701	-0.289923634
Middle SDI	1982	Female	0.762219288	0.693226101	0.838079007	-0.39172198
Middle SDI	1987	Female	0.699584721	0.621871747	0.787009193	-0.515429314
Middle SDI	1992	Female	0.674457874	0.577586154	0.787576746	-0.568199759
Middle SDI	1997	Female	0.652255447	0.525926306	0.808929243	-0.616491007
Middle SDI	2002	Female	0.580533475	0.406026756	0.830041643	-0.784548836
Middle SDI	1902	Both	1.090002449	1.026987003	1.156884493	0.124331376
Middle SDI	1907	Both	1.056999224	1.020301017	1.095017393	0.079974318
Middle SDI	1912	Both	1.078033691	1.048982112	1.107889853	0.108402266
Middle SDI	1917	Both	1.104232521	1.078527164	1.130550533	0.143043996
Middle SDI	1922	Both	1.135704006	1.11171401	1.160211689	0.18358688
Middle SDI	1927	Both	1.153134779	1.130840059	1.175869044	0.205561146
Middle SDI	1932	Both	1.158184259	1.137043625	1.179717952	0.211864794
Middle SDI	1937	Both	1.132019977	1.112021186	1.15237843	0.178899418
Middle SDI	1942	Both	1.081723818	1.062784823	1.101000309	0.113332202
Middle SDI	1947	Both	1.03539684	1.017355779	1.053757828	0.05018382
Middle SDI	1952	Both	1	1	1	0
Middle SDI	1957	Both	1.003550784	0.984023514	1.023465559	0.005113625
Middle SDI	1962	Both	0.968898251	0.947293296	0.990995951	-0.045582926
Middle SDI	1967	Both	0.92258496	0.89842669	0.947392836	-0.116246321
Middle SDI	1972	Both	0.915190753	0.885737987	0.945622891	-0.127855619
Middle SDI	1977	Both	0.918124152	0.88096652	0.956849028	-0.123238842
Middle SDI	1982	Both	0.905361797	0.858815316	0.954431026	-0.143433663
Middle SDI	1987	Both	0.874965242	0.818008683	0.93588759	-0.192702388
Middle SDI	1992	Both	0.865576675	0.789296149	0.949229235	-0.208266473
Middle SDI	1997	Both	0.858113907	0.751285777	0.980132327	-0.22075893
Middle SDI	2002	Both	0.771557296	0.612446455	0.97200442	-0.374154799

Table S12. Frontier Analysis of Ischemic Heart Disease Mortality and Socio-demograp[illegible] Differences and Trends, 1990-2021.

location_id	location_name	year	val	SDI
6	China	1990	94.14084669	0.458668935
6	China	1991	93.68220555	0.46730197
6	China	1992	93.23501245	0.475784031
6	China	1993	93.332957	0.484353831
6	China	1994	93.039362	0.492578645
6	China	1995	93.2128336	0.501500908
6	China	1996	93.19560195	0.512033996
6	China	1997	93.28624764	0.522326866
6	China	1998	94.93241203	0.531379484
6	China	1999	97.30290535	0.540333688
6	China	2000	102.5464155	0.547989193
6	China	2001	107.1187576	0.553680374
6	China	2002	111.7654169	0.560542936
6	China	2003	121.0866178	0.569018827
6	China	2004	126.7709604	0.578178091
6	China	2005	128.0425588	0.588052194
6	China	2006	120.8328099	0.59875817
6	China	2007	119.8361686	0.609520246
6	China	2008	122.4341143	0.619454833
6	China	2009	125.5544743	0.629307727
6	China	2010	128.606537	0.641521443
6	China	2011	129.7381556	0.651447978
6	China	2012	125.8285931	0.657144292
6	China	2013	126.4887515	0.663103007
6	China	2014	123.9296328	0.668640847
6	China	2015	119.6582745	0.671940111
6	China	2016	117.5629759	0.676218437
6	China	2017	116.1167557	0.684619661
6	China	2018	114.2835438	0.693717996
6	China	2019	112.8374611	0.703686824
6	China	2020	111.9994833	0.713364585
6	China	2021	110.9111825	0.72162976
7	Democratic People's Republic of Korea	1990	112.6479473	0.497780128
7	Democratic People's Republic of Korea	1991	114.0711063	0.499120512
7	Democratic People's Republic of Korea	1992	115.5779997	0.499629841
7	Democratic People's Republic of Korea	1993	117.1852657	0.499700646
7	Democratic People's Republic of Korea	1994	118.7319078	0.49953134
7	Democratic People's Republic of Korea	1995	120.2730315	0.499080529
7	Democratic People's Republic of Korea	1996	121.8661779	0.498429014
7	Democratic People's Republic of Korea	1997	123.562689	0.497136869
7	Democratic People's Republic of Korea	1998	125.3703273	0.49615921
7	Democratic People's Republic of Korea	1999	127.0836131	0.496484754
7	Democratic People's Republic of Korea	2000	128.8249605	0.497461995
7	Democratic People's Republic of Korea	2001	130.3520688	0.499674043
7	Democratic People's Republic of Korea	2002	132.6990374	0.502633569
7	Democratic People's Republic of Korea	2003	134.7068449	0.506165243

7	Democratic People's Republic of Korea	2004	136.0055132	0.510229134
7	Democratic People's Republic of Korea	2005	136.4985183	0.515097763
7	Democratic People's Republic of Korea	2006	136.1267049	0.51974144
7	Democratic People's Republic of Korea	2007	135.5545393	0.524066965
7	Democratic People's Republic of Korea	2008	135.3458704	0.528813498
7	Democratic People's Republic of Korea	2009	135.3597659	0.533114411
7	Democratic People's Republic of Korea	2010	135.1321164	0.537192918
7	Democratic People's Republic of Korea	2011	137.4234581	0.54121227
7	Democratic People's Republic of Korea	2012	137.122438	0.545331713
7	Democratic People's Republic of Korea	2013	137.8035166	0.549483297
7	Democratic People's Republic of Korea	2014	135.5571973	0.55360356
7	Democratic People's Republic of Korea	2015	132.6467705	0.557321271
7	Democratic People's Republic of Korea	2016	128.6697291	0.560743563
7	Democratic People's Republic of Korea	2017	127.2480211	0.563388289
7	Democratic People's Republic of Korea	2018	127.3677096	0.565161484
7	Democratic People's Republic of Korea	2019	126.8148207	0.566765706
7	Democratic People's Republic of Korea	2020	126.0873144	0.568188551
7	Democratic People's Republic of Korea	2021	125.3462237	0.569854634
8	Taiwan (Province of China)	1990	73.72110447	0.667633854
8	Taiwan (Province of China)	1991	73.62686191	0.676537237
8	Taiwan (Province of China)	1992	74.26331993	0.684803636
8	Taiwan (Province of China)	1993	70.42693609	0.69292413
8	Taiwan (Province of China)	1994	66.3089609	0.70107875
8	Taiwan (Province of China)	1995	63.25174819	0.709626821
8	Taiwan (Province of China)	1996	62.16320925	0.719128415
8	Taiwan (Province of China)	1997	57.28336713	0.729145497
8	Taiwan (Province of China)	1998	54.88623694	0.738125787
8	Taiwan (Province of China)	1999	51.39683261	0.745338399
8	Taiwan (Province of China)	2000	48.88060504	0.752926841
8	Taiwan (Province of China)	2001	48.22664414	0.760829809
8	Taiwan (Province of China)	2002	46.79071335	0.769008984
8	Taiwan (Province of China)	2003	47.41824169	0.776955803
8	Taiwan (Province of China)	2004	47.93476343	0.784677978
8	Taiwan (Province of China)	2005	46.92697195	0.792445129
8	Taiwan (Province of China)	2006	43.69030018	0.800174675
8	Taiwan (Province of China)	2007	42.33330351	0.808137694
8	Taiwan (Province of China)	2008	41.80212931	0.815400691
8	Taiwan (Province of China)	2009	39.19978074	0.821432279
8	Taiwan (Province of China)	2010	38.38972207	0.827226752
8	Taiwan (Province of China)	2011	39.73214875	0.832064074
8	Taiwan (Province of China)	2012	39.21054022	0.836677665
8	Taiwan (Province of China)	2013	38.82229461	0.841280463
8	Taiwan (Province of China)	2014	40.02577784	0.84569556
8	Taiwan (Province of China)	2015	39.00791405	0.850055269
8	Taiwan (Province of China)	2016	40.26689822	0.854466038
8	Taiwan (Province of China)	2017	38.35536129	0.858888019
8	Taiwan (Province of China)	2018	36.87724567	0.863158361
8	Taiwan (Province of China)	2019	35.4836299	0.86741185
8	Taiwan (Province of China)	2020	33.25619977	0.871247947
8	Taiwan (Province of China)	2021	33.60599652	0.874747053

10	Cambodia	1990	115.7344967	0.289075059
10	Cambodia	1991	114.4044793	0.2924792
10	Cambodia	1992	113.6203242	0.296900955
10	Cambodia	1993	113.491286	0.301627202
10	Cambodia	1994	113.6936381	0.305722592
10	Cambodia	1995	113.7219632	0.310678881
10	Cambodia	1996	113.0703393	0.31563097
10	Cambodia	1997	112.4237691	0.32050375
10	Cambodia	1998	111.9874197	0.324974299
10	Cambodia	1999	111.5501956	0.330224246
10	Cambodia	2000	110.8187424	0.33607503
10	Cambodia	2001	109.83351	0.342507911
10	Cambodia	2002	108.9939109	0.349465712
10	Cambodia	2003	107.9294055	0.35692966
10	Cambodia	2004	107.1492693	0.364854645
10	Cambodia	2005	106.303317	0.373331815
10	Cambodia	2006	105.4577671	0.381698166
10	Cambodia	2007	105.8470323	0.38987623
10	Cambodia	2008	105.7764606	0.39767952
10	Cambodia	2009	105.9292167	0.404220933
10	Cambodia	2010	105.6598132	0.410211121
10	Cambodia	2011	104.6952074	0.415964409
10	Cambodia	2012	105.5412736	0.421769754
10	Cambodia	2013	108.2644427	0.42771506
10	Cambodia	2014	107.6317539	0.433597749
10	Cambodia	2015	108.3758729	0.439487358
10	Cambodia	2016	109.530644	0.445358172
10	Cambodia	2017	109.110969	0.451221744
10	Cambodia	2018	109.8716263	0.45734149
10	Cambodia	2019	110.1763718	0.46364343
10	Cambodia	2020	110.8267664	0.468876054
10	Cambodia	2021	111.546706	0.473621491
11	Indonesia	1990	102.9582132	0.457134954
11	Indonesia	1991	104.9690216	0.466505583
11	Indonesia	1992	106.845871	0.475753157
11	Indonesia	1993	108.7309334	0.484775241
11	Indonesia	1994	110.0460132	0.4936071
11	Indonesia	1995	110.9377033	0.502008381
11	Indonesia	1996	112.0200862	0.510108146
11	Indonesia	1997	113.4973322	0.518102189
11	Indonesia	1998	114.7807329	0.524045604
11	Indonesia	1999	116.3470085	0.52917633
11	Indonesia	2000	118.1291558	0.533789587
11	Indonesia	2001	119.6167165	0.537977981
11	Indonesia	2002	122.0698331	0.542245676
11	Indonesia	2003	124.4922637	0.546550264
11	Indonesia	2004	127.0600277	0.550902314
11	Indonesia	2005	129.3667718	0.555424913
11	Indonesia	2006	131.5262777	0.560265924
11	Indonesia	2007	132.7104504	0.565811896

11	Indonesia	2008	135.2297192	0.572225267
11	Indonesia	2009	137.3977949	0.578866902
11	Indonesia	2010	139.061343	0.585982419
11	Indonesia	2011	138.2503562	0.593652985
11	Indonesia	2012	140.7792871	0.601185701
11	Indonesia	2013	141.8764543	0.608509904
11	Indonesia	2014	142.3720204	0.615499887
11	Indonesia	2015	144.3177205	0.622222469
11	Indonesia	2016	144.7197844	0.628666625
11	Indonesia	2017	143.0073628	0.634895745
11	Indonesia	2018	143.2201971	0.64094875
11	Indonesia	2019	143.3699733	0.646865457
11	Indonesia	2020	143.5332374	0.651926648
11	Indonesia	2021	143.2548234	0.656868336
12	Lao People's Democratic Republic	1990	235.3437968	0.264283164
12	Lao People's Democratic Republic	1991	232.7952769	0.268787618
12	Lao People's Democratic Republic	1992	230.4209767	0.273435312
12	Lao People's Democratic Republic	1993	227.8491901	0.278140418
12	Lao People's Democratic Republic	1994	224.7710024	0.28327023
12	Lao People's Democratic Republic	1995	221.6719506	0.288795341
12	Lao People's Democratic Republic	1996	219.056424	0.294838372
12	Lao People's Democratic Republic	1997	216.7554312	0.301477967
12	Lao People's Democratic Republic	1998	214.1630072	0.308402545
12	Lao People's Democratic Republic	1999	211.0413641	0.315972615
12	Lao People's Democratic Republic	2000	207.1281337	0.323947359
12	Lao People's Democratic Republic	2001	205.4297202	0.332580409
12	Lao People's Democratic Republic	2002	200.5442956	0.341667271
12	Lao People's Democratic Republic	2003	197.2974839	0.350658178
12	Lao People's Democratic Republic	2004	194.2580592	0.359681759
12	Lao People's Democratic Republic	2005	191.7629987	0.368728678
12	Lao People's Democratic Republic	2006	189.5047026	0.378122281
12	Lao People's Democratic Republic	2007	186.9201412	0.387723649
12	Lao People's Democratic Republic	2008	185.0098562	0.397011299
12	Lao People's Democratic Republic	2009	182.8699017	0.406139022
12	Lao People's Democratic Republic	2010	179.8153096	0.414865566
12	Lao People's Democratic Republic	2011	174.5508339	0.423354523
12	Lao People's Democratic Republic	2012	173.9887789	0.431149361
12	Lao People's Democratic Republic	2013	175.8094644	0.438691039
12	Lao People's Democratic Republic	2014	175.0513859	0.445869424
12	Lao People's Democratic Republic	2015	175.1920186	0.452749603
12	Lao People's Democratic Republic	2016	177.0812149	0.459583996
12	Lao People's Democratic Republic	2017	175.2404256	0.466168892
12	Lao People's Democratic Republic	2018	176.2240642	0.472618103
12	Lao People's Democratic Republic	2019	176.699509	0.478811373
12	Lao People's Democratic Republic	2020	176.671837	0.484192959
12	Lao People's Democratic Republic	2021	176.4620672	0.489136091
13	Malaysia	1990	177.4537397	0.545799405
13	Malaysia	1991	172.6215303	0.551683548
13	Malaysia	1992	171.7188657	0.558186752
13	Malaysia	1993	172.3943938	0.565845656

13	Malaysia	1994	171.5311374	0.574380446
13	Malaysia	1995	177.7827323	0.583441103
13	Malaysia	1996	173.4155613	0.593152135
13	Malaysia	1997	171.9169405	0.603418105
13	Malaysia	1998	173.9891784	0.612645412
13	Malaysia	1999	192.1481675	0.621724901
13	Malaysia	2000	177.6365385	0.630523544
13	Malaysia	2001	173.0201322	0.638257477
13	Malaysia	2002	171.2216584	0.645044248
13	Malaysia	2003	175.0016718	0.650885678
13	Malaysia	2004	172.506145	0.656243326
13	Malaysia	2005	169.83369	0.661242241
13	Malaysia	2006	165.9281554	0.665956287
13	Malaysia	2007	163.8727409	0.670843726
13	Malaysia	2008	168.2942131	0.67639991
13	Malaysia	2009	170.9848018	0.682070654
13	Malaysia	2010	169.7658643	0.688258622
13	Malaysia	2011	162.4788763	0.694083403
13	Malaysia	2012	154.9696518	0.699664734
13	Malaysia	2013	153.0307538	0.704930717
13	Malaysia	2014	151.9550758	0.709953756
13	Malaysia	2015	157.6462479	0.714659871
13	Malaysia	2016	154.2490087	0.719223425
13	Malaysia	2017	153.7161772	0.724235412
13	Malaysia	2018	151.8207976	0.729388078
13	Malaysia	2019	150.0032508	0.734587751
13	Malaysia	2020	133.4496973	0.738743993
13	Malaysia	2021	149.6591175	0.742523828
14	Maldives	1990	167.9912258	0.331601544
14	Maldives	1991	170.6015544	0.344398607
14	Maldives	1992	171.0805991	0.35825234
14	Maldives	1993	169.1455263	0.372806999
14	Maldives	1994	162.8536211	0.388081604
14	Maldives	1995	159.9915007	0.403945583
14	Maldives	1996	161.0656489	0.420519128
14	Maldives	1997	158.7873289	0.437436026
14	Maldives	1998	149.3045113	0.453973361
14	Maldives	1999	141.4120523	0.469338066
14	Maldives	2000	139.8550625	0.483495671
14	Maldives	2001	136.9575913	0.495923695
14	Maldives	2002	129.8769929	0.50740486
14	Maldives	2003	125.1170422	0.518926864
14	Maldives	2004	120.575109	0.529910191
14	Maldives	2005	116.7036364	0.538253332
14	Maldives	2006	113.0284128	0.547950412
14	Maldives	2007	109.264351	0.557529247
14	Maldives	2008	106.3799902	0.566839634
14	Maldives	2009	103.7769869	0.57444206
14	Maldives	2010	100.8152349	0.581980986
14	Maldives	2011	97.31584219	0.58968446

14	Maldives	2012	94.68822371	0.596948519
14	Maldives	2013	92.49373835	0.604238519
14	Maldives	2014	89.89458251	0.611578071
14	Maldives	2015	87.21696328	0.61845439
14	Maldives	2016	86.41826583	0.62487595
14	Maldives	2017	85.8978394	0.631083288
14	Maldives	2018	83.66604495	0.637205659
14	Maldives	2019	81.49860693	0.643082794
14	Maldives	2020	76.61578583	0.647608589
14	Maldives	2021	79.42926642	0.650886627
15	Myanmar	1990	193.3281004	0.319219724
15	Myanmar	1991	192.4856742	0.322034719
15	Myanmar	1992	191.2685195	0.325628884
15	Myanmar	1993	190.1182822	0.329707434
15	Myanmar	1994	188.4279818	0.33429012
15	Myanmar	1995	186.7469715	0.339590976
15	Myanmar	1996	184.902763	0.345191641
15	Myanmar	1997	182.9600484	0.350892883
15	Myanmar	1998	181.1851477	0.356696902
15	Myanmar	1999	179.3376346	0.363037732
15	Myanmar	2000	177.8437799	0.370360017
15	Myanmar	2001	176.4020769	0.37817198
15	Myanmar	2002	174.6582797	0.386343628
15	Myanmar	2003	173.7994191	0.395218766
15	Myanmar	2004	173.4272935	0.404692358
15	Myanmar	2005	171.6293286	0.414662082
15	Myanmar	2006	170.608495	0.424705071
15	Myanmar	2007	168.0783078	0.434536927
15	Myanmar	2008	165.7839007	0.443595531
15	Myanmar	2009	163.3470812	0.451954884
15	Myanmar	2010	159.4305731	0.460180429
15	Myanmar	2011	152.5341596	0.467850872
15	Myanmar	2012	149.8428109	0.475336423
15	Myanmar	2013	148.0491949	0.482821855
15	Myanmar	2014	143.6593211	0.490255145
15	Myanmar	2015	141.4885408	0.497374156
15	Myanmar	2016	140.5671404	0.504115978
15	Myanmar	2017	137.7852188	0.510823431
15	Myanmar	2018	137.215309	0.517557469
15	Myanmar	2019	136.9154736	0.52410897
15	Myanmar	2020	137.4229699	0.529434703
15	Myanmar	2021	138.2095124	0.53390084
16	Philippines	1990	174.1435094	0.510011796
16	Philippines	1991	160.8930226	0.513707183
16	Philippines	1992	158.3916426	0.517109502
16	Philippines	1993	157.385417	0.520600378
16	Philippines	1994	158.8659083	0.524613607
16	Philippines	1995	160.2872833	0.528827907
16	Philippines	1996	162.9761677	0.532938849
16	Philippines	1997	160.5402344	0.536589868

16	Philippines	1998	160.8212036	0.53949647
16	Philippines	1999	158.9061124	0.542257968
16	Philippines	2000	159.5864287	0.544997723
16	Philippines	2001	161.0060134	0.547409432
16	Philippines	2002	161.3589304	0.549521153
16	Philippines	2003	155.5107418	0.551567968
16	Philippines	2004	152.0740797	0.553704287
16	Philippines	2005	154.3730529	0.555664103
16	Philippines	2006	153.4151899	0.557976265
16	Philippines	2007	150.0271678	0.560822024
16	Philippines	2008	150.395798	0.563974721
16	Philippines	2009	151.2953877	0.56735582
16	Philippines	2010	150.1325153	0.571765888
16	Philippines	2011	148.3607818	0.577240283
16	Philippines	2012	149.1277443	0.583767625
16	Philippines	2013	148.855873	0.590837549
16	Philippines	2014	147.2542138	0.59838452
16	Philippines	2015	149.1084852	0.606215624
16	Philippines	2016	151.3417676	0.614224961
16	Philippines	2017	148.8694421	0.622315029
16	Philippines	2018	149.276148	0.630464276
16	Philippines	2019	151.7382335	0.638547384
16	Philippines	2020	148.3497897	0.644867233
16	Philippines	2021	150.4195702	0.651219329
17	Sri Lanka	1990	138.4419478	0.522622553
17	Sri Lanka	1991	127.9507002	0.528239203
17	Sri Lanka	1992	127.0156766	0.533345535
17	Sri Lanka	1993	120.0459126	0.538891102
17	Sri Lanka	1994	122.4460817	0.545124551
17	Sri Lanka	1995	124.5512073	0.551744058
17	Sri Lanka	1996	125.4809654	0.558938888
17	Sri Lanka	1997	132.5586641	0.566128422
17	Sri Lanka	1998	128.1553673	0.572326187
17	Sri Lanka	1999	128.3644332	0.577630048
17	Sri Lanka	2000	126.2871135	0.582942638
17	Sri Lanka	2001	119.0134753	0.587556599
17	Sri Lanka	2002	116.6107457	0.592359127
17	Sri Lanka	2003	119.4367473	0.597410284
17	Sri Lanka	2004	119.6796522	0.602250062
17	Sri Lanka	2005	125.7072247	0.606906656
17	Sri Lanka	2006	127.1388364	0.611807083
17	Sri Lanka	2007	125.6165595	0.616979265
17	Sri Lanka	2008	127.1293809	0.622414317
17	Sri Lanka	2009	123.2855077	0.62814963
17	Sri Lanka	2010	119.3408142	0.634263123
17	Sri Lanka	2011	119.3245855	0.640799571
17	Sri Lanka	2012	116.0594696	0.648049816
17	Sri Lanka	2013	117.3705959	0.655435838
17	Sri Lanka	2014	115.0723959	0.662782434
17	Sri Lanka	2015	113.0251615	0.669760292

17	Sri Lanka	2016	109.0375485	0.676277441
17	Sri Lanka	2017	107.471535	0.682405274
17	Sri Lanka	2018	105.0420287	0.688125858
17	Sri Lanka	2019	100.6398426	0.693516063
17	Sri Lanka	2020	89.58391778	0.697648535
17	Sri Lanka	2021	94.58632316	0.701534935
18	Thailand	1990	84.41757402	0.506644861
18	Thailand	1991	86.32113503	0.515911466
18	Thailand	1992	87.27099444	0.525161869
18	Thailand	1993	87.83883196	0.534303709
18	Thailand	1994	87.83445769	0.543325547
18	Thailand	1995	87.14874501	0.552452209
18	Thailand	1996	86.42088176	0.561302487
18	Thailand	1997	80.26029667	0.568449492
18	Thailand	1998	81.61209797	0.573817902
18	Thailand	1999	82.23600308	0.578854168
18	Thailand	2000	80.77141963	0.583500917
18	Thailand	2001	77.54343679	0.587661561
18	Thailand	2002	78.59521737	0.591759098
18	Thailand	2003	77.31953765	0.59604892
18	Thailand	2004	76.78677045	0.600520665
18	Thailand	2005	74.93977597	0.605040775
18	Thailand	2006	71.62583848	0.610356678
18	Thailand	2007	68.89072359	0.616368591
18	Thailand	2008	66.00614155	0.621067543
18	Thailand	2009	62.40463427	0.62432907
18	Thailand	2010	60.57228275	0.62858944
18	Thailand	2011	56.1427502	0.633226728
18	Thailand	2012	53.13900723	0.638410972
18	Thailand	2013	51.20688567	0.643672916
18	Thailand	2014	48.68842466	0.648772057
18	Thailand	2015	46.8935552	0.654015623
18	Thailand	2016	46.90236116	0.659369303
18	Thailand	2017	44.79611372	0.664804134
18	Thailand	2018	45.14925535	0.670225587
18	Thailand	2019	46.16614294	0.675273901
18	Thailand	2020	46.50252212	0.679119989
18	Thailand	2021	47.05461676	0.682547933
19	Timor-Leste	1990	122.7987264	0.262468083
19	Timor-Leste	1991	122.5516737	0.270943349
19	Timor-Leste	1992	122.2338524	0.27991676
19	Timor-Leste	1993	121.857931	0.289266629
19	Timor-Leste	1994	121.2247469	0.298763189
19	Timor-Leste	1995	120.2405426	0.308018684
19	Timor-Leste	1996	118.8990024	0.31758875
19	Timor-Leste	1997	117.7853455	0.326705374
19	Timor-Leste	1998	118.0559926	0.334894497
19	Timor-Leste	1999	118.911905	0.33925214
19	Timor-Leste	2000	119.3415441	0.343793649
19	Timor-Leste	2001	119.2597114	0.34949032

19	Timor-Leste	2002	118.9135486	0.35416202
19	Timor-Leste	2003	120.2466609	0.358440025
19	Timor-Leste	2004	120.9560832	0.362687998
19	Timor-Leste	2005	122.5594179	0.366913584
19	Timor-Leste	2006	125.2233959	0.370654092
19	Timor-Leste	2007	128.0164414	0.375390768
19	Timor-Leste	2008	131.0639639	0.381227528
19	Timor-Leste	2009	136.2938232	0.387944823
19	Timor-Leste	2010	139.0883116	0.394887019
19	Timor-Leste	2011	144.5811019	0.401872356
19	Timor-Leste	2012	149.1317563	0.4089103
19	Timor-Leste	2013	152.8784881	0.415217878
19	Timor-Leste	2014	155.804706	0.421155538
19	Timor-Leste	2015	154.620031	0.42638362
19	Timor-Leste	2016	155.3078286	0.431207272
19	Timor-Leste	2017	155.7185929	0.434519734
19	Timor-Leste	2018	156.7759152	0.437018663
19	Timor-Leste	2019	158.2397188	0.440195845
19	Timor-Leste	2020	157.1405437	0.442349336
19	Timor-Leste	2021	156.9483945	0.444667619
20	Viet Nam	1990	66.3986341	0.407630048
20	Viet Nam	1991	65.52822124	0.41321725
20	Viet Nam	1992	64.84462375	0.419781657
20	Viet Nam	1993	64.267835	0.427072185
20	Viet Nam	1994	63.839668	0.435106272
20	Viet Nam	1995	63.493645	0.443858785
20	Viet Nam	1996	63.12647233	0.45310241
20	Viet Nam	1997	62.89874769	0.462853518
20	Viet Nam	1998	62.63021836	0.472478892
20	Viet Nam	1999	62.53232247	0.481445774
20	Viet Nam	2000	62.57456628	0.489802045
20	Viet Nam	2001	63.48723751	0.497630349
20	Viet Nam	2002	64.85218433	0.505453447
20	Viet Nam	2003	66.44284964	0.513367511
20	Viet Nam	2004	68.50213067	0.52122939
20	Viet Nam	2005	70.77534366	0.528956332
20	Viet Nam	2006	73.2043807	0.536300653
20	Viet Nam	2007	75.63630038	0.543342652
20	Viet Nam	2008	78.00472141	0.550127119
20	Viet Nam	2009	80.21755033	0.556455944
20	Viet Nam	2010	81.01984558	0.562768571
20	Viet Nam	2011	80.33264726	0.568945458
20	Viet Nam	2012	80.35197199	0.575078672
20	Viet Nam	2013	80.97831017	0.581029215
20	Viet Nam	2014	79.93819362	0.586913815
20	Viet Nam	2015	79.82472989	0.592849573
20	Viet Nam	2016	79.71220345	0.598849485
20	Viet Nam	2017	78.63605712	0.605019421
20	Viet Nam	2018	78.38060569	0.611351406
20	Viet Nam	2019	78.14163451	0.617733815

20	Viet Nam	2020	77.84202251	0.62309032
20	Viet Nam	2021	76.99095272	0.627933721
22	Fiji	1990	291.0858157	0.534648908
22	Fiji	1991	295.600545	0.53877826
22	Fiji	1992	299.8793675	0.543188552
22	Fiji	1993	304.8686548	0.547776474
22	Fiji	1994	308.6018125	0.55284902
22	Fiji	1995	314.8859705	0.558530972
22	Fiji	1996	320.8440136	0.565041879
22	Fiji	1997	319.4675763	0.571187753
22	Fiji	1998	310.9592013	0.577172354
22	Fiji	1999	325.3374327	0.583573942
22	Fiji	2000	330.818348	0.589196631
22	Fiji	2001	318.9598621	0.594497419
22	Fiji	2002	307.4615605	0.599322031
22	Fiji	2003	307.1265526	0.603489622
22	Fiji	2004	287.3458418	0.60754489
22	Fiji	2005	282.1208601	0.610812944
22	Fiji	2006	281.7254946	0.613932767
22	Fiji	2007	279.8468357	0.616582915
22	Fiji	2008	280.7600451	0.618958809
22	Fiji	2009	279.8765863	0.620883465
22	Fiji	2010	282.2281221	0.623308786
22	Fiji	2011	282.8144608	0.626438251
22	Fiji	2012	281.3753447	0.629881335
22	Fiji	2013	281.2302441	0.634278114
22	Fiji	2014	281.4781563	0.639390066
22	Fiji	2015	280.8503282	0.645026353
22	Fiji	2016	279.7848103	0.65074268
22	Fiji	2017	278.8207913	0.65646401
22	Fiji	2018	276.0982718	0.661991454
22	Fiji	2019	272.9263987	0.667060979
22	Fiji	2020	270.734529	0.671431491
22	Fiji	2021	266.7765143	0.675051631
23	Kiribati	1990	189.6442847	0.410389821
23	Kiribati	1991	189.9896572	0.412884199
23	Kiribati	1992	191.4368698	0.415577609
23	Kiribati	1993	194.4001854	0.418481661
23	Kiribati	1994	194.036253	0.421716217
23	Kiribati	1995	194.7709959	0.425024354
23	Kiribati	1996	195.2734975	0.428300682
23	Kiribati	1997	196.1567568	0.431641941
23	Kiribati	1998	196.9806866	0.435741904
23	Kiribati	1999	197.9468791	0.439692923
23	Kiribati	2000	198.757451	0.444436339
23	Kiribati	2001	197.1609782	0.448782301
23	Kiribati	2002	196.7861503	0.453462298
23	Kiribati	2003	196.0231907	0.458208374
23	Kiribati	2004	195.734046	0.462336213
23	Kiribati	2005	195.4106816	0.466722276

23	Kiribati	2006	196.6207902	0.470557197
23	Kiribati	2007	198.0667225	0.47422745
23	Kiribati	2008	200.1267146	0.477201933
23	Kiribati	2009	201.9196142	0.480035601
23	Kiribati	2010	203.4402917	0.482447689
23	Kiribati	2011	205.0402887	0.485028211
23	Kiribati	2012	205.3152723	0.488175864
23	Kiribati	2013	205.5451754	0.491880767
23	Kiribati	2014	205.5090725	0.495317241
23	Kiribati	2015	205.5453855	0.500059699
23	Kiribati	2016	205.6699758	0.505207837
23	Kiribati	2017	205.4153288	0.510019241
23	Kiribati	2018	204.4646876	0.514810384
23	Kiribati	2019	203.9139835	0.519441321
23	Kiribati	2020	203.5038985	0.523454113
23	Kiribati	2021	202.9437363	0.527186583
24	Marshall Islands	1990	263.6776059	0.430839288
24	Marshall Islands	1991	268.2780298	0.435571412
24	Marshall Islands	1992	273.0413023	0.440491018
24	Marshall Islands	1993	274.1332661	0.445479084
24	Marshall Islands	1994	262.7939783	0.450848221
24	Marshall Islands	1995	266.341985	0.456859674
24	Marshall Islands	1996	267.6566102	0.46120943
24	Marshall Islands	1997	270.6846775	0.464752728
24	Marshall Islands	1998	272.6136456	0.468133032
24	Marshall Islands	1999	275.2959156	0.471292727
24	Marshall Islands	2000	277.6542366	0.474562536
24	Marshall Islands	2001	279.5619438	0.478588316
24	Marshall Islands	2002	282.0476514	0.482776259
24	Marshall Islands	2003	284.9468797	0.48624862
24	Marshall Islands	2004	288.1707989	0.489573077
24	Marshall Islands	2005	290.7478941	0.493271391
24	Marshall Islands	2006	294.3585294	0.497456175
24	Marshall Islands	2007	294.7965811	0.502250042
24	Marshall Islands	2008	295.3032029	0.506359588
24	Marshall Islands	2009	295.7749243	0.510794536
24	Marshall Islands	2010	294.8818566	0.516085138
24	Marshall Islands	2011	294.1061855	0.521177087
24	Marshall Islands	2012	292.5948912	0.526042019
24	Marshall Islands	2013	290.7773969	0.5314463
24	Marshall Islands	2014	289.3749797	0.536640131
24	Marshall Islands	2015	287.3498507	0.541793167
24	Marshall Islands	2016	285.0313897	0.546782936
24	Marshall Islands	2017	283.0895075	0.552065152
24	Marshall Islands	2018	281.0435461	0.557599278
24	Marshall Islands	2019	279.6299879	0.56342875
24	Marshall Islands	2020	277.8807665	0.568837509
24	Marshall Islands	2021	275.8971777	0.574091128
25	Micronesia (Federated States of)	1990	268.0171124	0.462511617
25	Micronesia (Federated States of)	1991	267.8235502	0.468144831

25	Micronesia (Federated States of)	1992	267.3114897	0.473792936
25	Micronesia (Federated States of)	1993	266.6853857	0.479939595
25	Micronesia (Federated States of)	1994	266.4352891	0.485300006
25	Micronesia (Federated States of)	1995	265.9960626	0.491015185
25	Micronesia (Federated States of)	1996	265.6255642	0.495570308
25	Micronesia (Federated States of)	1997	265.3379174	0.499110106
25	Micronesia (Federated States of)	1998	264.9856271	0.502861564
25	Micronesia (Federated States of)	1999	264.8213986	0.506663431
25	Micronesia (Federated States of)	2000	264.8727475	0.511004228
25	Micronesia (Federated States of)	2001	264.4377334	0.515316814
25	Micronesia (Federated States of)	2002	265.5278509	0.519545672
25	Micronesia (Federated States of)	2003	264.7975759	0.523829599
25	Micronesia (Federated States of)	2004	264.8272738	0.527610965
25	Micronesia (Federated States of)	2005	264.9764356	0.531703321
25	Micronesia (Federated States of)	2006	265.1112509	0.535718682
25	Micronesia (Federated States of)	2007	266.2097533	0.539431464
25	Micronesia (Federated States of)	2008	267.6274587	0.542766354
25	Micronesia (Federated States of)	2009	268.4481777	0.546275496
25	Micronesia (Federated States of)	2010	269.6977101	0.550122546
25	Micronesia (Federated States of)	2011	269.6144155	0.554240411
25	Micronesia (Federated States of)	2012	269.4413061	0.557965853
25	Micronesia (Federated States of)	2013	269.720529	0.561082821
25	Micronesia (Federated States of)	2014	269.27099	0.56384003
25	Micronesia (Federated States of)	2015	270.5942488	0.567151342
25	Micronesia (Federated States of)	2016	268.5600614	0.570458101
25	Micronesia (Federated States of)	2017	267.6665642	0.574039833
25	Micronesia (Federated States of)	2018	267.2831754	0.577555081
25	Micronesia (Federated States of)	2019	266.9705933	0.581195255
25	Micronesia (Federated States of)	2020	267.2776515	0.584527229
25	Micronesia (Federated States of)	2021	266.4343558	0.587534967
26	Papua New Guinea	1990	136.5213452	0.310668629
26	Papua New Guinea	1991	138.188415	0.314967305
26	Papua New Guinea	1992	138.3369425	0.319530259
26	Papua New Guinea	1993	138.4830553	0.325005984
26	Papua New Guinea	1994	138.2456944	0.330664468
26	Papua New Guinea	1995	138.5729374	0.335421285
26	Papua New Guinea	1996	140.0406533	0.340348698
26	Papua New Guinea	1997	140.2382059	0.344258325
26	Papua New Guinea	1998	141.2199076	0.347801408
26	Papua New Guinea	1999	142.3988506	0.351250621
26	Papua New Guinea	2000	144.1211807	0.354135791
26	Papua New Guinea	2001	145.9319294	0.356588986
26	Papua New Guinea	2002	147.8679929	0.358572086
26	Papua New Guinea	2003	148.672321	0.360736816
26	Papua New Guinea	2004	149.5395306	0.362827542
26	Papua New Guinea	2005	151.7026241	0.365294965
26	Papua New Guinea	2006	154.5057732	0.367826759
26	Papua New Guinea	2007	155.3046846	0.370794147
26	Papua New Guinea	2008	154.5870427	0.373426209
26	Papua New Guinea	2009	152.3211444	0.376528285

26	Papua New Guinea	2010	153.2314894	0.380286778
26	Papua New Guinea	2011	153.7641486	0.383587539
26	Papua New Guinea	2012	152.2205963	0.386781028
26	Papua New Guinea	2013	149.7459972	0.389926437
26	Papua New Guinea	2014	148.2530994	0.394004571
26	Papua New Guinea	2015	148.3351361	0.398285864
26	Papua New Guinea	2016	146.8791099	0.402407267
26	Papua New Guinea	2017	145.9330882	0.406153435
26	Papua New Guinea	2018	145.3295834	0.409165083
26	Papua New Guinea	2019	146.1556251	0.412311147
26	Papua New Guinea	2020	145.785398	0.415186879
26	Papua New Guinea	2021	144.4477023	0.417797443
27	Samoa	1990	205.7266389	0.487491428
27	Samoa	1991	205.670576	0.490987146
27	Samoa	1992	206.8826636	0.494027448
27	Samoa	1993	208.3870213	0.497079264
27	Samoa	1994	209.9150635	0.499484836
27	Samoa	1995	211.3559261	0.502369735
27	Samoa	1996	213.1329394	0.505778026
27	Samoa	1997	215.0147837	0.509096382
27	Samoa	1998	216.2263644	0.512367566
27	Samoa	1999	217.7254904	0.51540905
27	Samoa	2000	218.5756246	0.518902327
27	Samoa	2001	218.9408735	0.522971626
27	Samoa	2002	219.4553734	0.52724267
27	Samoa	2003	220.2903169	0.531603736
27	Samoa	2004	220.4710226	0.536003276
27	Samoa	2005	220.4039415	0.540557908
27	Samoa	2006	220.0243099	0.544812068
27	Samoa	2007	220.5111593	0.549110752
27	Samoa	2008	221.0648668	0.553164596
27	Samoa	2009	221.752678	0.556474015
27	Samoa	2010	222.6476273	0.559622905
27	Samoa	2011	222.6760495	0.562891574
27	Samoa	2012	225.6751251	0.565320388
27	Samoa	2013	228.2757495	0.567448536
27	Samoa	2014	230.239847	0.569493661
27	Samoa	2015	231.7165699	0.571998983
27	Samoa	2016	233.4111155	0.575402051
27	Samoa	2017	232.2489818	0.578925435
27	Samoa	2018	230.7777134	0.582320661
27	Samoa	2019	229.1962271	0.586256695
27	Samoa	2020	227.7980081	0.590075296
27	Samoa	2021	226.7242162	0.593392769
28	Solomon Islands	1990	292.8172221	0.301217167
28	Solomon Islands	1991	295.679809	0.305830668
28	Solomon Islands	1992	294.7761327	0.311431697
28	Solomon Islands	1993	294.3298797	0.317151092
28	Solomon Islands	1994	292.8098292	0.323300247
28	Solomon Islands	1995	288.0100018	0.329968564

28	Solomon Islands	1996	285.8868726	0.336208053
28	Solomon Islands	1997	285.5651929	0.341835627
28	Solomon Islands	1998	282.2847317	0.34713506
28	Solomon Islands	1999	277.3388949	0.351867191
28	Solomon Islands	2000	276.6313418	0.354799143
28	Solomon Islands	2001	272.828207	0.356626745
28	Solomon Islands	2002	270.9131238	0.357910116
28	Solomon Islands	2003	267.865183	0.359424359
28	Solomon Islands	2004	264.9006538	0.361290558
28	Solomon Islands	2005	264.4169265	0.363580019
28	Solomon Islands	2006	266.314902	0.366443698
28	Solomon Islands	2007	263.0011857	0.369827951
28	Solomon Islands	2008	260.1068722	0.373764884
28	Solomon Islands	2009	265.3566971	0.377821028
28	Solomon Islands	2010	263.6627769	0.38271759
28	Solomon Islands	2011	272.0047834	0.388055337
28	Solomon Islands	2012	274.4416304	0.39300464
28	Solomon Islands	2013	276.2005279	0.39790625
28	Solomon Islands	2014	278.8708546	0.402244745
28	Solomon Islands	2015	278.8156898	0.406233897
28	Solomon Islands	2016	275.9908099	0.410406962
28	Solomon Islands	2017	276.6092408	0.414676394
28	Solomon Islands	2018	276.6674775	0.418889221
28	Solomon Islands	2019	278.764359	0.422736386
28	Solomon Islands	2020	278.9448961	0.426112429
28	Solomon Islands	2021	275.1674793	0.429360316
29	Tonga	1990	138.6901401	0.49180684
29	Tonga	1991	137.2111812	0.498166205
29	Tonga	1992	134.1090359	0.503659848
29	Tonga	1993	134.878919	0.50890445
29	Tonga	1994	135.1326381	0.514207835
29	Tonga	1995	131.3768988	0.52021502
29	Tonga	1996	131.7774872	0.525808135
29	Tonga	1997	135.3935337	0.531009092
29	Tonga	1998	143.295074	0.536172314
29	Tonga	1999	150.238678	0.54131702
29	Tonga	2000	148.6574561	0.546303195
29	Tonga	2001	145.4428027	0.551054251
29	Tonga	2002	145.4147297	0.556008345
29	Tonga	2003	145.4336569	0.560736066
29	Tonga	2004	142.1188705	0.564806379
29	Tonga	2005	134.5676443	0.568449169
29	Tonga	2006	136.44548	0.571619868
29	Tonga	2007	138.9451232	0.57435151
29	Tonga	2008	141.370545	0.577305415
29	Tonga	2009	143.3724501	0.57917075
29	Tonga	2010	145.0469852	0.580657299
29	Tonga	2011	145.4671312	0.582656723
29	Tonga	2012	145.8084724	0.58477088
29	Tonga	2013	145.7425046	0.587351141

29	Tonga	2014	145.2499518	0.590826853
29	Tonga	2015	144.6490885	0.594965748
29	Tonga	2016	143.6230814	0.600343669
29	Tonga	2017	142.3071863	0.606243592
29	Tonga	2018	141.1811337	0.611953942
29	Tonga	2019	140.4094175	0.617324063
29	Tonga	2020	139.5147947	0.622104456
29	Tonga	2021	139.1274907	0.626349936
30	Vanuatu	1990	312.3512339	0.353100252
30	Vanuatu	1991	315.7687794	0.357005379
30	Vanuatu	1992	318.1850738	0.360893296
30	Vanuatu	1993	319.4945251	0.364444155
30	Vanuatu	1994	323.3049099	0.368501002
30	Vanuatu	1995	320.7929015	0.372479126
30	Vanuatu	1996	322.5237985	0.376460885
30	Vanuatu	1997	322.8507593	0.380667171
30	Vanuatu	1998	323.0714248	0.384690196
30	Vanuatu	1999	324.0401338	0.388300526
30	Vanuatu	2000	319.3788225	0.392158349
30	Vanuatu	2001	320.8976842	0.395325055
30	Vanuatu	2002	314.8124377	0.397723782
30	Vanuatu	2003	308.8199381	0.400396456
30	Vanuatu	2004	309.4182067	0.403226097
30	Vanuatu	2005	315.7687086	0.406488572
30	Vanuatu	2006	317.3690138	0.410501729
30	Vanuatu	2007	320.2596106	0.414915578
30	Vanuatu	2008	321.4617444	0.419853237
30	Vanuatu	2009	319.120083	0.42485465
30	Vanuatu	2010	317.1761388	0.429658049
30	Vanuatu	2011	318.1518463	0.434283044
30	Vanuatu	2012	316.1192607	0.438661998
30	Vanuatu	2013	314.6792652	0.442720176
30	Vanuatu	2014	313.0804671	0.446687298
30	Vanuatu	2015	312.3931543	0.450329861
30	Vanuatu	2016	311.8991049	0.454072763
30	Vanuatu	2017	311.2136491	0.458016834
30	Vanuatu	2018	310.792226	0.462080221
30	Vanuatu	2019	310.3106518	0.466276162
30	Vanuatu	2020	309.719277	0.470038391
30	Vanuatu	2021	308.3905609	0.473100706
33	Armenia	1990	307.4353058	0.544414535
33	Armenia	1991	332.6922677	0.547561863
33	Armenia	1992	377.0820434	0.548974724
33	Armenia	1993	399.0738491	0.551214674
33	Armenia	1994	381.0590832	0.553783844
33	Armenia	1995	376.5211308	0.556815513
33	Armenia	1996	372.3906118	0.560537778
33	Armenia	1997	354.8277123	0.564627462
33	Armenia	1998	348.7586319	0.569556663
33	Armenia	1999	349.047123	0.574323064

33	Armenia	2000	335.1635054	0.579437569
33	Armenia	2001	323.9799862	0.585419295
33	Armenia	2002	313.5459713	0.592710614
33	Armenia	2003	312.302982	0.60080917
33	Armenia	2004	307.4279554	0.609031766
33	Armenia	2005	299.7298851	0.617759634
33	Armenia	2006	290.7821134	0.626688335
33	Armenia	2007	277.1668924	0.635870982
33	Armenia	2008	274.9552081	0.644431591
33	Armenia	2009	269.9139682	0.649714845
33	Armenia	2010	265.8926282	0.654811534
33	Armenia	2011	258.361906	0.659818092
33	Armenia	2012	250.1219965	0.664833143
33	Armenia	2013	242.1567648	0.669534496
33	Armenia	2014	239.5889215	0.67393219
33	Armenia	2015	235.3841276	0.678054635
33	Armenia	2016	234.1356021	0.681746395
33	Armenia	2017	224.8087692	0.685980265
33	Armenia	2018	219.4528742	0.690276872
33	Armenia	2019	214.4598868	0.694765962
33	Armenia	2020	212.9641745	0.698268899
33	Armenia	2021	209.7752951	0.701833194
34	Azerbaijan	1990	362.3062123	0.595986033
34	Azerbaijan	1991	370.4465061	0.595807712
34	Azerbaijan	1992	392.1372109	0.594651335
34	Azerbaijan	1993	405.8984314	0.593542676
34	Azerbaijan	1994	412.7712244	0.592088785
34	Azerbaijan	1995	411.3791371	0.589441457
34	Azerbaijan	1996	397.98125	0.586315946
34	Azerbaijan	1997	381.7715712	0.583221813
34	Azerbaijan	1998	373.3298259	0.580392893
34	Azerbaijan	1999	369.373174	0.579192506
34	Azerbaijan	2000	367.9430783	0.580020573
34	Azerbaijan	2001	358.5359318	0.582193708
34	Azerbaijan	2002	365.3129492	0.585716143
34	Azerbaijan	2003	374.4718176	0.590038705
34	Azerbaijan	2004	376.1717641	0.594646085
34	Azerbaijan	2005	387.1583088	0.602485193
34	Azerbaijan	2006	385.0027661	0.613771097
34	Azerbaijan	2007	379.2868877	0.626110926
34	Azerbaijan	2008	379.7652866	0.636901109
34	Azerbaijan	2009	377.3604116	0.645163341
34	Azerbaijan	2010	377.6378506	0.652056507
34	Azerbaijan	2011	375.9938722	0.657971629
34	Azerbaijan	2012	381.8674731	0.662897002
34	Azerbaijan	2013	382.1339637	0.667658779
34	Azerbaijan	2014	384.1987838	0.672162741
34	Azerbaijan	2015	378.188746	0.676244911
34	Azerbaijan	2016	377.8582431	0.679807643
34	Azerbaijan	2017	376.4442117	0.683263204

34	Azerbaijan	2018	347.5733681	0.686561093
34	Azerbaijan	2019	334.2928763	0.689834715
34	Azerbaijan	2020	322.6759944	0.692605192
34	Azerbaijan	2021	306.1346909	0.694851274
35	Georgia	1990	358.6450299	0.656136044
35	Georgia	1991	371.8605605	0.662113186
35	Georgia	1992	386.2663681	0.665021428
35	Georgia	1993	362.2098683	0.663466862
35	Georgia	1994	350.7534025	0.658134139
35	Georgia	1995	348.714348	0.651363604
35	Georgia	1996	372.7106084	0.64455809
35	Georgia	1997	370.0150174	0.638525631
35	Georgia	1998	373.9704264	0.633347931
35	Georgia	1999	375.7942075	0.630277946
35	Georgia	2000	366.6086525	0.630265991
35	Georgia	2001	352.5400431	0.6333883
35	Georgia	2002	336.75982	0.638370108
35	Georgia	2003	304.1054017	0.644065579
35	Georgia	2004	284.4699366	0.648920978
35	Georgia	2005	243.1236564	0.653616005
35	Georgia	2006	217.4904681	0.658403372
35	Georgia	2007	208.8069976	0.663697966
35	Georgia	2008	238.3478881	0.668442496
35	Georgia	2009	229.3785224	0.672449381
35	Georgia	2010	219.4532058	0.677034892
35	Georgia	2011	192.431517	0.681567044
35	Georgia	2012	183.9668554	0.685519223
35	Georgia	2013	175.3186028	0.688603268
35	Georgia	2014	171.0433868	0.692155207
35	Georgia	2015	158.318236	0.697316133
35	Georgia	2016	146.8546738	0.70373149
35	Georgia	2017	140.9867149	0.710874718
35	Georgia	2018	132.7604643	0.717993654
35	Georgia	2019	127.8037396	0.724536462
35	Georgia	2020	132.579402	0.729125889
35	Georgia	2021	124.1554838	0.732473604
36	Kazakhstan	1990	298.6516728	0.589435804
36	Kazakhstan	1991	322.0352171	0.590967832
36	Kazakhstan	1992	353.9425962	0.596127664
36	Kazakhstan	1993	400.476085	0.603138328
36	Kazakhstan	1994	432.7947449	0.6105911
36	Kazakhstan	1995	464.2701123	0.618573582
36	Kazakhstan	1996	462.6495798	0.626013138
36	Kazakhstan	1997	448.1649531	0.631166626
36	Kazakhstan	1998	436.8754031	0.635388624
36	Kazakhstan	1999	429.4060698	0.640180485
36	Kazakhstan	2000	432.1550376	0.644689623
36	Kazakhstan	2001	430.7284727	0.648397258
36	Kazakhstan	2002	434.4539157	0.651529335
36	Kazakhstan	2003	436.5355995	0.654835926

36	Kazakhstan	2004	399.8897221	0.658733071
36	Kazakhstan	2005	397.0252852	0.662943942
36	Kazakhstan	2006	394.975127	0.667048858
36	Kazakhstan	2007	396.0859793	0.671367208
36	Kazakhstan	2008	369.8331347	0.676352869
36	Kazakhstan	2009	316.1281206	0.681606839
36	Kazakhstan	2010	289.2324642	0.686996643
36	Kazakhstan	2011	282.4551723	0.691758952
36	Kazakhstan	2012	280.3325421	0.695312767
36	Kazakhstan	2013	277.3121912	0.699053593
36	Kazakhstan	2014	271.6067279	0.703511921
36	Kazakhstan	2015	267.6648247	0.708229736
36	Kazakhstan	2016	263.2794553	0.71256904
36	Kazakhstan	2017	251.7617262	0.716026688
36	Kazakhstan	2018	249.8509466	0.718531999
36	Kazakhstan	2019	247.1205812	0.720797602
36	Kazakhstan	2020	244.8315607	0.722788768
36	Kazakhstan	2021	235.9767142	0.725144495
37	Kyrgyzstan	1990	264.381502	0.519407652
37	Kyrgyzstan	1991	263.3469676	0.523746714
37	Kyrgyzstan	1992	275.0940356	0.527901552
37	Kyrgyzstan	1993	299.7932591	0.532431008
37	Kyrgyzstan	1994	321.6070304	0.53623298
37	Kyrgyzstan	1995	320.8927198	0.53905282
37	Kyrgyzstan	1996	318.643112	0.541628927
37	Kyrgyzstan	1997	317.0226915	0.54334115
37	Kyrgyzstan	1998	315.2452523	0.543258546
37	Kyrgyzstan	1999	306.1202363	0.542867282
37	Kyrgyzstan	2000	315.87607	0.543036487
37	Kyrgyzstan	2001	313.4335428	0.543805551
37	Kyrgyzstan	2002	326.3505501	0.54437467
37	Kyrgyzstan	2003	334.4629363	0.545649262
37	Kyrgyzstan	2004	328.3269424	0.547262343
37	Kyrgyzstan	2005	343.6949578	0.548260449
37	Kyrgyzstan	2006	357.373192	0.549383594
37	Kyrgyzstan	2007	361.3534526	0.551146192
37	Kyrgyzstan	2008	362.766037	0.553206382
37	Kyrgyzstan	2009	359.3857294	0.555123866
37	Kyrgyzstan	2010	366.1945971	0.556416545
37	Kyrgyzstan	2011	373.0132847	0.558923335
37	Kyrgyzstan	2012	374.4957981	0.561541574
37	Kyrgyzstan	2013	356.9950764	0.565776769
37	Kyrgyzstan	2014	349.2327102	0.570569357
37	Kyrgyzstan	2015	352.2706641	0.575463231
37	Kyrgyzstan	2016	341.2320469	0.580388551
37	Kyrgyzstan	2017	324.5659866	0.585766096
37	Kyrgyzstan	2018	301.7170417	0.591237134
37	Kyrgyzstan	2019	291.1997349	0.596501638
37	Kyrgyzstan	2020	285.4482971	0.600499554
37	Kyrgyzstan	2021	274.4089663	0.603979328

38	Mongolia	1990	324.7935127	0.466550118
38	Mongolia	1991	357.4114081	0.472199014
38	Mongolia	1992	374.3051235	0.477586554
38	Mongolia	1993	366.4318643	0.482726727
38	Mongolia	1994	334.9262911	0.488276683
38	Mongolia	1995	360.0809876	0.495006781
38	Mongolia	1996	377.3402239	0.501703718
38	Mongolia	1997	377.5426395	0.508558268
38	Mongolia	1998	370.0836706	0.514793383
38	Mongolia	1999	366.5949848	0.520781189
38	Mongolia	2000	365.3258102	0.526650345
38	Mongolia	2001	369.198763	0.532620201
38	Mongolia	2002	355.6102029	0.538424835
38	Mongolia	2003	341.9672214	0.543971651
38	Mongolia	2004	328.5747761	0.5495515
38	Mongolia	2005	322.7648138	0.554680284
38	Mongolia	2006	313.6814544	0.559561138
38	Mongolia	2007	297.5084586	0.564236795
38	Mongolia	2008	290.4529182	0.568500599
38	Mongolia	2009	296.3489052	0.571341686
38	Mongolia	2010	287.4395209	0.574406669
38	Mongolia	2011	286.3037092	0.578600663
38	Mongolia	2012	277.5549509	0.583255886
38	Mongolia	2013	268.9220105	0.588080811
38	Mongolia	2014	254.1245126	0.592867353
38	Mongolia	2015	255.7260113	0.596859648
38	Mongolia	2016	250.1166259	0.600260767
38	Mongolia	2017	241.942089	0.603721965
38	Mongolia	2018	241.3633523	0.607501833
38	Mongolia	2019	228.6963104	0.611250681
38	Mongolia	2020	216.8345267	0.614629637
38	Mongolia	2021	219.5871972	0.617621565
39	Tajikistan	1990	304.8271152	0.466155413
39	Tajikistan	1991	314.6347973	0.472342173
39	Tajikistan	1992	341.9803186	0.475745195
39	Tajikistan	1993	350.1479336	0.478238813
39	Tajikistan	1994	378.7883734	0.478726011
39	Tajikistan	1995	373.2878641	0.477586868
39	Tajikistan	1996	346.248173	0.474346027
39	Tajikistan	1997	327.3287211	0.470274796
39	Tajikistan	1998	329.5171963	0.465341742
39	Tajikistan	1999	324.991905	0.460505616
39	Tajikistan	2000	323.1994988	0.457061333
39	Tajikistan	2001	324.5765397	0.456256513
39	Tajikistan	2002	327.6116588	0.45880778
39	Tajikistan	2003	328.0293003	0.463329706
39	Tajikistan	2004	332.562953	0.469154214
39	Tajikistan	2005	343.7536101	0.474637786
39	Tajikistan	2006	350.8485073	0.479651831
39	Tajikistan	2007	363.1409334	0.484211156

39	Tajikistan	2008	362.9558775	0.488441136
39	Tajikistan	2009	358.6837054	0.492064041
39	Tajikistan	2010	337.5816826	0.495893964
39	Tajikistan	2011	330.182564	0.499869804
39	Tajikistan	2012	312.7567344	0.503953885
39	Tajikistan	2013	286.9250483	0.507970676
39	Tajikistan	2014	281.5284779	0.511989518
39	Tajikistan	2015	274.5200091	0.515783245
39	Tajikistan	2016	267.4482384	0.519897002
39	Tajikistan	2017	259.7759918	0.524492607
39	Tajikistan	2018	254.7634813	0.529153916
39	Tajikistan	2019	250.0683927	0.533769951
39	Tajikistan	2020	248.5224338	0.537690531
39	Tajikistan	2021	244.9206236	0.541511187
40	Turkmenistan	1990	407.00521	0.563126887
40	Turkmenistan	1991	432.0349096	0.564939386
40	Turkmenistan	1992	451.8378872	0.565587505
40	Turkmenistan	1993	509.3731504	0.567174449
40	Turkmenistan	1994	508.6504733	0.567288976
40	Turkmenistan	1995	508.5610263	0.567198635
40	Turkmenistan	1996	531.9478843	0.567061261
40	Turkmenistan	1997	519.3719177	0.565623084
40	Turkmenistan	1998	499.8357735	0.564405264
40	Turkmenistan	1999	472.6573084	0.564716006
40	Turkmenistan	2000	462.9653381	0.567421033
40	Turkmenistan	2001	453.2175207	0.571043819
40	Turkmenistan	2002	469.0106727	0.575596166
40	Turkmenistan	2003	477.1789468	0.580820126
40	Turkmenistan	2004	481.8748674	0.586712349
40	Turkmenistan	2005	485.0357498	0.593388895
40	Turkmenistan	2006	469.0001655	0.599896069
40	Turkmenistan	2007	453.7726949	0.606042198
40	Turkmenistan	2008	399.4028781	0.611923309
40	Turkmenistan	2009	346.4611784	0.617652905
40	Turkmenistan	2010	327.1054877	0.623234589
40	Turkmenistan	2011	325.3892389	0.629233722
40	Turkmenistan	2012	323.2207983	0.635263183
40	Turkmenistan	2013	328.4357948	0.641361893
40	Turkmenistan	2014	336.6325149	0.647542417
40	Turkmenistan	2015	341.9377081	0.65335279
40	Turkmenistan	2016	338.7953412	0.658945613
40	Turkmenistan	2017	345.9518386	0.664284692
40	Turkmenistan	2018	346.4999582	0.669433599
40	Turkmenistan	2019	348.6818277	0.674303536
40	Turkmenistan	2020	346.7060115	0.67829818
40	Turkmenistan	2021	343.6764756	0.682160776
41	Uzbekistan	1990	318.6204598	0.500241735
41	Uzbekistan	1991	331.5894638	0.501544609
41	Uzbekistan	1992	344.21492	0.502905194
41	Uzbekistan	1993	372.0620544	0.506556615

41	Uzbekistan	1994	382.8127266	0.511018525
41	Uzbekistan	1995	388.404301	0.516986264
41	Uzbekistan	1996	389.5999789	0.52439291
41	Uzbekistan	1997	387.1060611	0.532686328
41	Uzbekistan	1998	392.5201142	0.541279069
41	Uzbekistan	1999	382.5379488	0.549739391
41	Uzbekistan	2000	388.3928348	0.557522311
41	Uzbekistan	2001	390.8406064	0.564803383
41	Uzbekistan	2002	399.2445528	0.571690143
41	Uzbekistan	2003	407.140033	0.578006538
41	Uzbekistan	2004	409.9499207	0.583942986
41	Uzbekistan	2005	433.4105174	0.589530777
41	Uzbekistan	2006	443.3507594	0.594498057
41	Uzbekistan	2007	446.4248585	0.599628055
41	Uzbekistan	2008	445.3145995	0.604889158
41	Uzbekistan	2009	433.0689831	0.610351745
41	Uzbekistan	2010	431.891547	0.616097907
41	Uzbekistan	2011	436.1269732	0.621344619
41	Uzbekistan	2012	443.3383527	0.626202125
41	Uzbekistan	2013	438.539245	0.630796403
41	Uzbekistan	2014	432.1995185	0.63525705
41	Uzbekistan	2015	428.0036947	0.639805639
41	Uzbekistan	2016	411.9169174	0.644230597
41	Uzbekistan	2017	395.9741236	0.648119347
41	Uzbekistan	2018	375.5048414	0.651918144
41	Uzbekistan	2019	349.34772	0.655836959
41	Uzbekistan	2020	344.6775136	0.659124204
41	Uzbekistan	2021	339.4785785	0.662621694
43	Albania	1990	174.2346015	0.5577733
43	Albania	1991	171.9373495	0.555629412
43	Albania	1992	160.6873209	0.553107897
43	Albania	1993	150.5263258	0.552453328
43	Albania	1994	139.0140773	0.553763669
43	Albania	1995	144.3923873	0.557040889
43	Albania	1996	154.1843381	0.562248886
43	Albania	1997	154.8552233	0.566802804
43	Albania	1998	151.5786204	0.572534102
43	Albania	1999	150.0943941	0.578295462
43	Albania	2000	152.4596747	0.584664188
43	Albania	2001	148.867513	0.592412947
43	Albania	2002	154.7896401	0.599865978
43	Albania	2003	163.0520695	0.606971166
43	Albania	2004	163.3438655	0.613864247
43	Albania	2005	160.8226417	0.620679615
43	Albania	2006	153.3871107	0.627485617
43	Albania	2007	143.6808687	0.634167969
43	Albania	2008	146.0312081	0.640766134
43	Albania	2009	144.7701905	0.64681613
43	Albania	2010	146.9506618	0.652654203
43	Albania	2011	148.9592395	0.658196773

43	Albania	2012	152.2024734	0.66355678
43	Albania	2013	156.4593976	0.668739559
43	Albania	2014	161.0033882	0.674112985
43	Albania	2015	163.7665168	0.679668601
43	Albania	2016	166.1356808	0.685036648
43	Albania	2017	167.8947312	0.690379199
43	Albania	2018	164.3800602	0.695566878
43	Albania	2019	161.3096848	0.700287863
43	Albania	2020	158.1512689	0.703790245
43	Albania	2021	158.2861727	0.706849791
44	Bosnia and Herzegovina	1990	200.0943779	0.54113254
44	Bosnia and Herzegovina	1991	209.7560411	0.541346251
44	Bosnia and Herzegovina	1992	210.919832	0.539303227
44	Bosnia and Herzegovina	1993	216.501257	0.536031882
44	Bosnia and Herzegovina	1994	216.161994	0.53385339
44	Bosnia and Herzegovina	1995	213.7714796	0.534025536
44	Bosnia and Herzegovina	1996	210.8693396	0.545549215
44	Bosnia and Herzegovina	1997	205.8997039	0.563565415
44	Bosnia and Herzegovina	1998	194.1220712	0.581829515
44	Bosnia and Herzegovina	1999	191.027834	0.597997469
44	Bosnia and Herzegovina	2000	184.9830562	0.610950685
44	Bosnia and Herzegovina	2001	174.0048197	0.621989464
44	Bosnia and Herzegovina	2002	165.3907184	0.632075518
44	Bosnia and Herzegovina	2003	156.0562342	0.640635102
44	Bosnia and Herzegovina	2004	148.4458895	0.647793732
44	Bosnia and Herzegovina	2005	146.5197208	0.653859137
44	Bosnia and Herzegovina	2006	141.1488883	0.659813662
44	Bosnia and Herzegovina	2007	148.0610578	0.665578735
44	Bosnia and Herzegovina	2008	141.7557613	0.671100814
44	Bosnia and Herzegovina	2009	143.1160738	0.6760329
44	Bosnia and Herzegovina	2010	140.4130234	0.680824196
44	Bosnia and Herzegovina	2011	141.2434315	0.685399264
44	Bosnia and Herzegovina	2012	139.5343609	0.689506566
44	Bosnia and Herzegovina	2013	141.1879884	0.693508711
44	Bosnia and Herzegovina	2014	141.893072	0.697031893
44	Bosnia and Herzegovina	2015	146.3337782	0.700446047
44	Bosnia and Herzegovina	2016	143.0279935	0.704079344
44	Bosnia and Herzegovina	2017	143.9225331	0.708053431
44	Bosnia and Herzegovina	2018	143.5804307	0.712347302
44	Bosnia and Herzegovina	2019	147.0436931	0.716857125
44	Bosnia and Herzegovina	2020	147.3880999	0.720202056
44	Bosnia and Herzegovina	2021	138.2950349	0.723077893
45	Bulgaria	1990	403.9782016	0.633446498
45	Bulgaria	1991	375.8541404	0.643069488
45	Bulgaria	1992	365.3003475	0.651186876
45	Bulgaria	1993	410.2521858	0.659161569
45	Bulgaria	1994	428.6388126	0.66743046
45	Bulgaria	1995	431.3942856	0.673999977
45	Bulgaria	1996	431.5645784	0.678862584
45	Bulgaria	1997	440.8601863	0.680221466

45	Bulgaria	1998	415.7229524	0.678496877
45	Bulgaria	1999	387.8137454	0.677446543
45	Bulgaria	2000	373.1160866	0.681004524
45	Bulgaria	2001	367.3311457	0.686487366
45	Bulgaria	2002	360.8109304	0.691511674
45	Bulgaria	2003	340.1941582	0.695565965
45	Bulgaria	2004	315.9265612	0.699881951
45	Bulgaria	2005	293.6212016	0.704471207
45	Bulgaria	2006	278.1598507	0.708868294
45	Bulgaria	2007	259.5609091	0.713192402
45	Bulgaria	2008	245.4144619	0.71767245
45	Bulgaria	2009	242.6102718	0.723676627
45	Bulgaria	2010	240.9575935	0.730701737
45	Bulgaria	2011	235.1772801	0.735749281
45	Bulgaria	2012	222.7215065	0.739177517
45	Bulgaria	2013	215.0942841	0.742135948
45	Bulgaria	2014	218.1336456	0.745372643
45	Bulgaria	2015	217.3298318	0.748979058
45	Bulgaria	2016	215.4564342	0.752259199
45	Bulgaria	2017	215.930585	0.755665865
45	Bulgaria	2018	211.7167454	0.75914846
45	Bulgaria	2019	207.0011262	0.762359791
45	Bulgaria	2020	200.4335104	0.765089711
45	Bulgaria	2021	204.4041154	0.768150939
46	Croatia	1990	287.3852516	0.668906358
46	Croatia	1991	288.1732689	0.6747246
46	Croatia	1992	274.6868458	0.678093133
46	Croatia	1993	268.62353	0.678221047
46	Croatia	1994	264.4970423	0.677548133
46	Croatia	1995	255.66203	0.677029465
46	Croatia	1996	242.3676864	0.679507318
46	Croatia	1997	237.313095	0.68536501
46	Croatia	1998	226.6150933	0.691365519
46	Croatia	1999	215.7321002	0.696636411
46	Croatia	2000	203.3180286	0.703039899
46	Croatia	2001	195.6638637	0.709536841
46	Croatia	2002	196.9860761	0.715383422
46	Croatia	2003	204.0088062	0.721130377
46	Croatia	2004	186.7947783	0.726327652
46	Croatia	2005	183.2896816	0.73215414
46	Croatia	2006	177.4792598	0.738066879
46	Croatia	2007	176.0131376	0.743148777
46	Croatia	2008	178.2448774	0.748469602
46	Croatia	2009	171.7713929	0.754065605
46	Croatia	2010	168.7673912	0.759472252
46	Croatia	2011	162.1006242	0.763686381
46	Croatia	2012	157.6703414	0.767405336
46	Croatia	2013	147.7835935	0.77115188
46	Croatia	2014	146.4815064	0.774479241
46	Croatia	2015	151.1066756	0.777865888

46	Croatia	2016	138.9293183	0.781443605
46	Croatia	2017	142.0095202	0.785074537
46	Croatia	2018	139.2368652	0.78867141
46	Croatia	2019	133.6083028	0.792475415
46	Croatia	2020	131.3749506	0.795462272
46	Croatia	2021	130.5223715	0.798341027
47	Czechia	1990	296.0319999	0.681848021
47	Czechia	1991	284.6336848	0.688002859
47	Czechia	1992	277.247652	0.697577809
47	Czechia	1993	268.4968125	0.710853416
47	Czechia	1994	264.1191377	0.725969686
47	Czechia	1995	261.5835233	0.737981722
47	Czechia	1996	245.1869463	0.74604138
47	Czechia	1997	242.1366447	0.751680344
47	Czechia	1998	224.6213271	0.757033755
47	Czechia	1999	216.9952612	0.76242888
47	Czechia	2000	210.1608217	0.767766159
47	Czechia	2001	206.4071363	0.77216724
47	Czechia	2002	203.8785162	0.776927934
47	Czechia	2003	206.5956073	0.782448081
47	Czechia	2004	196.6343275	0.787126405
47	Czechia	2005	194.5320744	0.791333948
47	Czechia	2006	187.9078313	0.794922784
47	Czechia	2007	190.1856028	0.798254118
47	Czechia	2008	187.7308977	0.801764846
47	Czechia	2009	181.0789656	0.804962225
47	Czechia	2010	175.8870717	0.808747328
47	Czechia	2011	175.1823647	0.812054784
47	Czechia	2012	170.8032446	0.814230401
47	Czechia	2013	164.2922984	0.815615361
47	Czechia	2014	154.2455149	0.816227257
47	Czechia	2015	151.4827593	0.816564684
47	Czechia	2016	144.2911541	0.817153026
47	Czechia	2017	141.3830094	0.81906768
47	Czechia	2018	137.4433753	0.822034486
47	Czechia	2019	132.4537625	0.824784473
47	Czechia	2020	133.6354857	0.826626631
47	Czechia	2021	126.6839516	0.828450433
48	Hungary	1990	262.8009852	0.649419991
48	Hungary	1991	261.6499405	0.653707155
48	Hungary	1992	270.740824	0.660382905
48	Hungary	1993	274.4744565	0.667543556
48	Hungary	1994	267.6669127	0.675051406
48	Hungary	1995	265.480885	0.683186412
48	Hungary	1996	255.0171475	0.690883334
48	Hungary	1997	252.4684084	0.697935514
48	Hungary	1998	254.3856609	0.70420272
48	Hungary	1999	252.6262565	0.709980627
48	Hungary	2000	235.0488099	0.715805281
48	Hungary	2001	225.8824116	0.722099055

48	Hungary	2002	224.9760306	0.728384596
48	Hungary	2003	227.7848409	0.734450658
48	Hungary	2004	230.1025666	0.740099975
48	Hungary	2005	239.8583496	0.745356806
48	Hungary	2006	227.9108278	0.750515381
48	Hungary	2007	223.2308286	0.754942942
48	Hungary	2008	212.8041164	0.759317468
48	Hungary	2009	212.0390702	0.763563687
48	Hungary	2010	207.0015654	0.767328649
48	Hungary	2011	199.7579441	0.769701942
48	Hungary	2012	194.7903124	0.770784245
48	Hungary	2013	186.3228964	0.771561698
48	Hungary	2014	184.3382467	0.772575046
48	Hungary	2015	185.8589516	0.774047383
48	Hungary	2016	177.8345804	0.776150305
48	Hungary	2017	179.1597131	0.779116417
48	Hungary	2018	175.7895237	0.782083661
48	Hungary	2019	168.5960599	0.784910309
48	Hungary	2020	164.6158524	0.78762039
48	Hungary	2021	155.3585723	0.790754768
49	North Macedonia	1990	208.5703275	0.609026094
49	North Macedonia	1991	216.5784845	0.611337453
49	North Macedonia	1992	234.0016546	0.612708856
49	North Macedonia	1993	241.9950681	0.613195099
49	North Macedonia	1994	251.0325255	0.614362905
49	North Macedonia	1995	268.7087277	0.616994878
49	North Macedonia	1996	280.9666495	0.620944017
49	North Macedonia	1997	291.7560363	0.626223811
49	North Macedonia	1998	304.7887161	0.63205941
49	North Macedonia	1999	308.9612829	0.636687259
49	North Macedonia	2000	307.5370934	0.641651243
49	North Macedonia	2001	305.5406072	0.648049676
49	North Macedonia	2002	298.7871283	0.65539879
49	North Macedonia	2003	280.4036837	0.663408891
49	North Macedonia	2004	263.456432	0.67123752
49	North Macedonia	2005	265.5492288	0.678047273
49	North Macedonia	2006	276.4754254	0.684044336
49	North Macedonia	2007	281.6650328	0.689729481
49	North Macedonia	2008	273.7621077	0.695376955
49	North Macedonia	2009	269.5483495	0.700453052
49	North Macedonia	2010	262.7639562	0.705589324
49	North Macedonia	2011	257.8650342	0.710592521
49	North Macedonia	2012	252.8554661	0.715173148
49	North Macedonia	2013	240.4182795	0.719671149
49	North Macedonia	2014	237.4780496	0.724089589
49	North Macedonia	2015	232.9592028	0.728603773
49	North Macedonia	2016	227.3221931	0.733173624
49	North Macedonia	2017	221.1913737	0.737339955
49	North Macedonia	2018	219.9650807	0.74133437
49	North Macedonia	2019	221.7233379	0.745193569

49	North Macedonia	2020	201.137746	0.7478499
49	North Macedonia	2021	195.8631642	0.750629703
50	Montenegro	1990	164.639364	0.67422572
50	Montenegro	1991	161.6621215	0.674305723
50	Montenegro	1992	160.2480268	0.672664772
50	Montenegro	1993	162.1524917	0.668665719
50	Montenegro	1994	166.0266332	0.665011931
50	Montenegro	1995	164.2888593	0.662765437
50	Montenegro	1996	162.477725	0.663414769
50	Montenegro	1997	166.0901266	0.665542032
50	Montenegro	1998	175.8050862	0.669024771
50	Montenegro	1999	180.459626	0.672096103
50	Montenegro	2000	179.1863546	0.67730685
50	Montenegro	2001	177.8273053	0.683433454
50	Montenegro	2002	178.5087706	0.689939483
50	Montenegro	2003	180.6922476	0.696517506
50	Montenegro	2004	176.0734111	0.702998276
50	Montenegro	2005	168.1925199	0.709065465
50	Montenegro	2006	166.0848041	0.715459074
50	Montenegro	2007	158.1940892	0.72250394
50	Montenegro	2008	157.830023	0.729981756
50	Montenegro	2009	169.9881653	0.736538926
50	Montenegro	2010	178.2894704	0.743347873
50	Montenegro	2011	184.3217942	0.750080328
50	Montenegro	2012	185.5735325	0.75557838
50	Montenegro	2013	189.4983391	0.760886907
50	Montenegro	2014	195.406196	0.765776527
50	Montenegro	2015	203.2448627	0.770561278
50	Montenegro	2016	210.8962273	0.77520077
50	Montenegro	2017	216.5639564	0.779764632
50	Montenegro	2018	218.2085435	0.784408971
50	Montenegro	2019	217.9234856	0.789026903
50	Montenegro	2020	228.672255	0.792554393
50	Montenegro	2021	212.8253993	0.795800584
51	Poland	1990	269.7513401	0.627227888
51	Poland	1991	278.2148588	0.632671471
51	Poland	1992	267.9526772	0.640661793
51	Poland	1993	255.648382	0.6489505
51	Poland	1994	246.7897227	0.658010514
51	Poland	1995	241.729346	0.666678867
51	Poland	1996	233.1112261	0.674378829
51	Poland	1997	228.4054847	0.682587612
51	Poland	1998	215.7972234	0.690805671
51	Poland	1999	211.5788143	0.698536056
51	Poland	2000	194.2773145	0.706662052
51	Poland	2001	185.1088509	0.714831304
51	Poland	2002	177.3477623	0.722009458
51	Poland	2003	173.8504436	0.728155254
51	Poland	2004	167.8506796	0.733673192
51	Poland	2005	162.579205	0.738428767

51	Poland	2006	160.4590704	0.742363709
51	Poland	2007	159.3072039	0.746008817
51	Poland	2008	157.4031713	0.750521426
51	Poland	2009	153.5226193	0.756427378
51	Poland	2010	143.0976854	0.763184974
51	Poland	2011	136.8059405	0.769407164
51	Poland	2012	135.1055941	0.774680767
51	Poland	2013	129.5594985	0.779434257
51	Poland	2014	122.8414309	0.783682369
51	Poland	2015	123.0449914	0.787445343
51	Poland	2016	118.4121104	0.790651015
51	Poland	2017	118.8292256	0.794530345
51	Poland	2018	118.8965478	0.799478982
51	Poland	2019	115.1949698	0.804791332
51	Poland	2020	113.7906339	0.808795318
51	Poland	2021	113.2647118	0.812042809
52	Romania	1990	278.0332886	0.619298862
52	Romania	1991	283.1852408	0.626795627
52	Romania	1992	297.389593	0.630475082
52	Romania	1993	295.4306304	0.633801422
52	Romania	1994	294.0610165	0.638371932
52	Romania	1995	300.6980667	0.643718879
52	Romania	1996	307.661918	0.648104195
52	Romania	1997	298.7721602	0.651225576
52	Romania	1998	285.7758431	0.654927769
52	Romania	1999	269.0090053	0.659544846
52	Romania	2000	249.6391327	0.665294346
52	Romania	2001	246.4877508	0.672020167
52	Romania	2002	245.792332	0.678297417
52	Romania	2003	239.0805875	0.684074929
52	Romania	2004	229.3399056	0.690417055
52	Romania	2005	224.4074714	0.69499895
52	Romania	2006	215.9832418	0.699490766
52	Romania	2007	205.8475118	0.704523727
52	Romania	2008	203.1597202	0.710757128
52	Romania	2009	202.1247073	0.717445122
52	Romania	2010	198.2525712	0.723891349
52	Romania	2011	184.6530555	0.729018272
52	Romania	2012	182.495133	0.733892482
52	Romania	2013	172.330864	0.737880473
52	Romania	2014	175.2217038	0.740395467
52	Romania	2015	172.557832	0.74347353
52	Romania	2016	167.5565697	0.74705871
52	Romania	2017	163.6607772	0.751117367
52	Romania	2018	160.9161414	0.755533333
52	Romania	2019	156.1018886	0.760283971
52	Romania	2020	151.6905125	0.764276444
52	Romania	2021	154.192475	0.768453864
53	Serbia	1990	309.1175515	0.63051102
53	Serbia	1991	312.5320835	0.638016934

53	Serbia	1992	311.3667787	0.641971994
53	Serbia	1993	321.7182046	0.642096034
53	Serbia	1994	337.3439221	0.642223221
53	Serbia	1995	348.4841603	0.642739671
53	Serbia	1996	358.9513551	0.644846417
53	Serbia	1997	363.2100169	0.647162094
53	Serbia	1998	329.4244427	0.650245989
53	Serbia	1999	317.7637954	0.655534174
53	Serbia	2000	308.1088253	0.6607528
53	Serbia	2001	284.8293521	0.665407335
53	Serbia	2002	262.9230841	0.671511072
53	Serbia	2003	256.2697932	0.678334929
53	Serbia	2004	247.9021222	0.686819842
53	Serbia	2005	243.3918074	0.696097568
53	Serbia	2006	234.5015105	0.704945018
53	Serbia	2007	226.4043284	0.713242305
53	Serbia	2008	221.3410933	0.721505306
53	Serbia	2009	214.2184866	0.729965596
53	Serbia	2010	205.6523228	0.740091311
53	Serbia	2011	201.0304399	0.750072762
53	Serbia	2012	194.9727619	0.757105082
53	Serbia	2013	186.3271788	0.762170908
53	Serbia	2014	185.7776543	0.766043091
53	Serbia	2015	187.9636217	0.769842223
53	Serbia	2016	180.6780203	0.773654077
53	Serbia	2017	181.072616	0.777460384
53	Serbia	2018	175.2283429	0.781494974
53	Serbia	2019	175.3933122	0.7857683
53	Serbia	2020	179.7758392	0.789183333
53	Serbia	2021	174.28362	0.792416294
54	Slovakia	1990	327.254579	0.653853505
54	Slovakia	1991	319.0973266	0.658731402
54	Slovakia	1992	310.4512123	0.665830111
54	Slovakia	1993	303.5893193	0.675967937
54	Slovakia	1994	293.2183073	0.688136477
54	Slovakia	1995	292.9439786	0.697476536
54	Slovakia	1996	280.6551379	0.704405003
54	Slovakia	1997	289.3286105	0.71151321
54	Slovakia	1998	288.2209281	0.719058766
54	Slovakia	1999	281.7026346	0.726144173
54	Slovakia	2000	269.6906873	0.73404624
54	Slovakia	2001	266.9805037	0.741392554
54	Slovakia	2002	260.5878475	0.746791142
54	Slovakia	2003	258.0364362	0.75116727
54	Slovakia	2004	251.626611	0.755836694
54	Slovakia	2005	254.6300335	0.761724185
54	Slovakia	2006	249.7539581	0.767673036
54	Slovakia	2007	250.6187255	0.772914153
54	Slovakia	2008	241.5690921	0.7771974
54	Slovakia	2009	238.9168699	0.78105226

54	Slovakia	2010	229.7762333	0.786254422
54	Slovakia	2011	226.7181504	0.791465382
54	Slovakia	2012	217.8173395	0.795456216
54	Slovakia	2013	210.8348815	0.797903723
54	Slovakia	2014	204.6708318	0.799618796
54	Slovakia	2015	208.6039436	0.80039418
54	Slovakia	2016	200.260867	0.80087821
54	Slovakia	2017	201.4760764	0.802277317
54	Slovakia	2018	197.9515161	0.804357302
54	Slovakia	2019	188.1984234	0.806448457
54	Slovakia	2020	189.39275	0.808305966
54	Slovakia	2021	184.7659386	0.81061053
55	Slovenia	1990	128.3015372	0.727463928
55	Slovenia	1991	136.4242242	0.732666986
55	Slovenia	1992	142.2822244	0.737391552
55	Slovenia	1993	148.1517917	0.741774172
55	Slovenia	1994	141.3472288	0.746234861
55	Slovenia	1995	125.3931544	0.750994975
55	Slovenia	1996	120.6349219	0.756348987
55	Slovenia	1997	120.4243599	0.762275292
55	Slovenia	1998	120.2090828	0.767728526
55	Slovenia	1999	114.0161265	0.773236192
55	Slovenia	2000	104.2065556	0.77957801
55	Slovenia	2001	100.6777212	0.786240776
55	Slovenia	2002	91.55905275	0.791805569
55	Slovenia	2003	90.97922758	0.796371531
55	Slovenia	2004	86.16401223	0.80096224
55	Slovenia	2005	80.36913338	0.805601248
55	Slovenia	2006	75.76836288	0.809708595
55	Slovenia	2007	73.71380837	0.813006319
55	Slovenia	2008	70.668693	0.81634725
55	Slovenia	2009	68.17453856	0.81887856
55	Slovenia	2010	66.56769939	0.821178486
55	Slovenia	2011	64.10588285	0.823023561
55	Slovenia	2012	63.1318181	0.824336563
55	Slovenia	2013	60.40285213	0.825826275
55	Slovenia	2014	55.73943703	0.827799246
55	Slovenia	2015	57.47790055	0.829837071
55	Slovenia	2016	54.49060937	0.831367915
55	Slovenia	2017	53.73045248	0.833038139
55	Slovenia	2018	51.53150189	0.835472481
55	Slovenia	2019	49.72866113	0.838266782
55	Slovenia	2020	49.31931354	0.840373846
55	Slovenia	2021	45.94794722	0.842430731
57	Belarus	1990	331.1290855	0.622446576
57	Belarus	1991	335.1908638	0.627038267
57	Belarus	1992	344.468164	0.632891737
57	Belarus	1993	383.5396701	0.639394169
57	Belarus	1994	394.8387938	0.644652781
57	Belarus	1995	410.300556	0.648959504

57	Belarus	1996	401.6559378	0.652855221
57	Belarus	1997	405.2495718	0.655627296
57	Belarus	1998	409.9888219	0.657246467
57	Belarus	1999	424.1031581	0.66017101
57	Belarus	2000	402.5755948	0.664705822
57	Belarus	2001	421.0017654	0.671333269
57	Belarus	2002	448.8507609	0.678121782
57	Belarus	2003	437.0389813	0.684868556
57	Belarus	2004	423.909628	0.692502135
57	Belarus	2005	428.1192205	0.698985483
57	Belarus	2006	410.9293104	0.705119103
57	Belarus	2007	391.0364573	0.711496562
57	Belarus	2008	394.0608122	0.719120871
57	Belarus	2009	398.9801311	0.727292784
57	Belarus	2010	401.1515146	0.734954626
57	Belarus	2011	405.7166576	0.739504326
57	Belarus	2012	372.1049031	0.743468837
57	Belarus	2013	376.6913604	0.748529323
57	Belarus	2014	370.3112694	0.75351148
57	Belarus	2015	356.6628011	0.758205743
57	Belarus	2016	352.8592999	0.764328222
57	Belarus	2017	340.5414449	0.771131466
57	Belarus	2018	342.3101901	0.776568957
57	Belarus	2019	346.0917265	0.780508019
57	Belarus	2020	345.0792489	0.782381565
57	Belarus	2021	341.5058801	0.784484711
58	Estonia	1990	368.9511867	0.674967632
58	Estonia	1991	373.1482605	0.685223089
58	Estonia	1992	365.3635468	0.695586674
58	Estonia	1993	386.596841	0.70369315
58	Estonia	1994	408.1831923	0.708456181
58	Estonia	1995	380.981877	0.713172725
58	Estonia	1996	338.7062624	0.718813024
58	Estonia	1997	328.8178564	0.72465244
58	Estonia	1998	340.9523945	0.729027035
58	Estonia	1999	319.6172983	0.733250616
58	Estonia	2000	309.5582245	0.739713154
58	Estonia	2001	311.8748379	0.746602207
58	Estonia	2002	297.8534642	0.752629898
58	Estonia	2003	286.7838559	0.757815678
58	Estonia	2004	271.7115656	0.763223727
58	Estonia	2005	259.3922416	0.769112182
58	Estonia	2006	251.277915	0.774451842
58	Estonia	2007	243.0826373	0.781074443
58	Estonia	2008	221.2427647	0.788598874
58	Estonia	2009	203.966791	0.794728213
58	Estonia	2010	186.9553916	0.800829718
58	Estonia	2011	177.8373	0.806633078
58	Estonia	2012	168.5560375	0.811109358
58	Estonia	2013	151.097374	0.814887177

58	Estonia	2014	142.8052991	0.818770732
58	Estonia	2015	131.8007962	0.823221278
58	Estonia	2016	130.1312152	0.82736436
58	Estonia	2017	115.5695215	0.830975311
58	Estonia	2018	108.7239352	0.834566296
58	Estonia	2019	95.96307539	0.838573509
58	Estonia	2020	92.55608647	0.841817139
58	Estonia	2021	92.05654067	0.844917787
59	Latvia	1990	343.0614092	0.680193638
59	Latvia	1991	340.423108	0.688117297
59	Latvia	1992	349.7281865	0.696157036
59	Latvia	1993	393.4935544	0.705249855
59	Latvia	1994	422.4626341	0.712531353
59	Latvia	1995	388.9263892	0.717639459
59	Latvia	1996	329.8919721	0.721139538
59	Latvia	1997	314.0641178	0.72374658
59	Latvia	1998	324.3411689	0.725427336
59	Latvia	1999	301.1700709	0.727219685
59	Latvia	2000	290.1619808	0.730951449
59	Latvia	2001	295.8267544	0.736294196
59	Latvia	2002	286.6369872	0.74187044
59	Latvia	2003	282.0998436	0.748168162
59	Latvia	2004	280.0046851	0.755263211
59	Latvia	2005	284.4979199	0.761929433
59	Latvia	2006	294.8310005	0.768459929
59	Latvia	2007	290.8388237	0.776481959
59	Latvia	2008	265.3410989	0.786324573
59	Latvia	2009	252.2084622	0.794504427
59	Latvia	2010	247.3023141	0.798171594
59	Latvia	2011	239.0317916	0.799080776
59	Latvia	2012	228.6922394	0.801026474
59	Latvia	2013	222.7298156	0.80266049
59	Latvia	2014	209.4173819	0.803721486
59	Latvia	2015	197.8329151	0.806985995
59	Latvia	2016	186.5720522	0.811463591
59	Latvia	2017	177.1102618	0.816923118
59	Latvia	2018	172.4724035	0.821419809
59	Latvia	2019	163.3160757	0.824781777
59	Latvia	2020	163.3692685	0.827900448
59	Latvia	2021	160.3001591	0.830663516
60	Lithuania	1990	365.286145	0.668503938
60	Lithuania	1991	374.8380595	0.673234121
60	Lithuania	1992	365.2006554	0.68099288
60	Lithuania	1993	395.0687276	0.687480621
60	Lithuania	1994	397.0179887	0.691151653
60	Lithuania	1995	381.702046	0.69473438
60	Lithuania	1996	356.9354064	0.698591301
60	Lithuania	1997	336.0117774	0.70284095
60	Lithuania	1998	323.4867356	0.707468182
60	Lithuania	1999	306.3223359	0.712834171

60	Lithuania	2000	295.3212892	0.71989696
60	Lithuania	2001	308.5335757	0.727133585
60	Lithuania	2002	302.4434956	0.733793398
60	Lithuania	2003	302.5000495	0.741229601
60	Lithuania	2004	298.4969735	0.748949762
60	Lithuania	2005	313.1194364	0.75635236
60	Lithuania	2006	312.4052373	0.763059124
60	Lithuania	2007	313.246388	0.770229639
60	Lithuania	2008	300.8697366	0.778273712
60	Lithuania	2009	277.1314476	0.785689739
60	Lithuania	2010	280.9298782	0.793364682
60	Lithuania	2011	273.8844063	0.800924994
60	Lithuania	2012	266.4794545	0.807827026
60	Lithuania	2013	262.736976	0.813219722
60	Lithuania	2014	250.4599558	0.817886376
60	Lithuania	2015	253.4166893	0.823112629
60	Lithuania	2016	246.9821097	0.829303695
60	Lithuania	2017	228.0842352	0.83587174
60	Lithuania	2018	217.0383498	0.84191891
60	Lithuania	2019	205.9224012	0.847906339
60	Lithuania	2020	199.6553121	0.852752388
60	Lithuania	2021	197.077632	0.856484049
61	Republic of Moldova	1990	404.1664362	0.604251762
61	Republic of Moldova	1991	439.9455253	0.607270774
61	Republic of Moldova	1992	433.4513956	0.609664558
61	Republic of Moldova	1993	460.5124855	0.61415292
61	Republic of Moldova	1994	511.9883138	0.617603767
61	Republic of Moldova	1995	537.3815918	0.620951321
61	Republic of Moldova	1996	519.2707018	0.623113984
61	Republic of Moldova	1997	472.9814843	0.624370105
61	Republic of Moldova	1998	439.6563632	0.624116761
61	Republic of Moldova	1999	434.5670098	0.622834529
61	Republic of Moldova	2000	420.2536553	0.622004934
61	Republic of Moldova	2001	398.0744006	0.62293641
61	Republic of Moldova	2002	397.8223148	0.625874845
61	Republic of Moldova	2003	392.2685815	0.629985045
61	Republic of Moldova	2004	375.4603664	0.635394822
61	Republic of Moldova	2005	384.8880341	0.641530965
61	Republic of Moldova	2006	365.396426	0.647996264
61	Republic of Moldova	2007	350.8308582	0.654215851
61	Republic of Moldova	2008	336.3314567	0.661046597
61	Republic of Moldova	2009	334.3105486	0.666284394
61	Republic of Moldova	2010	346.6234401	0.671989616
61	Republic of Moldova	2011	312.7088526	0.678004137
61	Republic of Moldova	2012	306.6293961	0.682979796
61	Republic of Moldova	2013	288.3753893	0.688519175
61	Republic of Moldova	2014	290.024948	0.694104557
61	Republic of Moldova	2015	282.2396967	0.699355166
61	Republic of Moldova	2016	260.9954902	0.705144542
61	Republic of Moldova	2017	240.3425359	0.711206637

61	Republic of Moldova	2018	238.6613125	0.717244427
61	Republic of Moldova	2019	234.7264841	0.723011648
61	Republic of Moldova	2020	229.1002015	0.72771054
61	Republic of Moldova	2021	230.5175115	0.732214875
62	Russian Federation	1990	311.2670485	0.671600578
62	Russian Federation	1991	307.3891932	0.680099211
62	Russian Federation	1992	324.7105564	0.688068022
62	Russian Federation	1993	382.3826311	0.692036194
62	Russian Federation	1994	409.8338136	0.694144966
62	Russian Federation	1995	385.570568	0.697854214
62	Russian Federation	1996	360.9513937	0.701468231
62	Russian Federation	1997	340.9662332	0.703752867
62	Russian Federation	1998	337.0929419	0.705198518
62	Russian Federation	1999	363.9052852	0.706863538
62	Russian Federation	2000	377.9718355	0.708643648
62	Russian Federation	2001	382.9248445	0.710570359
62	Russian Federation	2002	395.4782424	0.713460957
62	Russian Federation	2003	400.7981373	0.718875116
62	Russian Federation	2004	381.6181877	0.726305319
62	Russian Federation	2005	388.5162311	0.733592777
62	Russian Federation	2006	359.3085836	0.740064664
62	Russian Federation	2007	343.7740553	0.746191512
62	Russian Federation	2008	345.5682865	0.752623243
62	Russian Federation	2009	329.5587093	0.757960405
62	Russian Federation	2010	331.5145005	0.763320116
62	Russian Federation	2011	309.8501949	0.767633588
62	Russian Federation	2012	298.0522982	0.771920384
62	Russian Federation	2013	284.1276635	0.776526979
62	Russian Federation	2014	274.5303543	0.780617524
62	Russian Federation	2015	261.4070677	0.784913774
62	Russian Federation	2016	250.8768716	0.790330149
62	Russian Federation	2017	231.6308752	0.795669484
62	Russian Federation	2018	227.1720158	0.800108144
62	Russian Federation	2019	219.2146124	0.803658201
62	Russian Federation	2020	214.866085	0.806011474
62	Russian Federation	2021	212.9139642	0.808536005
63	Ukraine	1990	338.0567746	0.647461466
63	Ukraine	1991	355.5992533	0.651950021
63	Ukraine	1992	379.83694	0.657600333
63	Ukraine	1993	414.9095283	0.662867405
63	Ukraine	1994	440.5504855	0.665506375
63	Ukraine	1995	472.0187344	0.666950167
63	Ukraine	1996	476.4846967	0.668104744
63	Ukraine	1997	477.3560465	0.669207846
63	Ukraine	1998	465.7072203	0.670322301
63	Ukraine	1999	496.8176988	0.670642905
63	Ukraine	2000	528.4114799	0.671095001
63	Ukraine	2001	532.9050513	0.67246782
63	Ukraine	2002	551.7929719	0.674543221
63	Ukraine	2003	556.0240764	0.678614636

63	Ukraine	2004	545.7239542	0.685380936
63	Ukraine	2005	535.236113	0.692023301
63	Ukraine	2006	505.7104401	0.698093383
63	Ukraine	2007	508.95884	0.704202308
63	Ukraine	2008	507.4111116	0.710980184
63	Ukraine	2009	468.581228	0.716185465
63	Ukraine	2010	456.8838137	0.72101987
63	Ukraine	2011	433.7312853	0.724891957
63	Ukraine	2012	427.8636261	0.7288172
63	Ukraine	2013	425.1732564	0.734033573
63	Ukraine	2014	415.8919579	0.739883557
63	Ukraine	2015	406.1876598	0.743790863
63	Ukraine	2016	397.3310534	0.746857989
63	Ukraine	2017	392.0316542	0.750593324
63	Ukraine	2018	395.1366671	0.754451727
63	Ukraine	2019	395.632426	0.757980081
63	Ukraine	2020	369.8817324	0.759929872
63	Ukraine	2021	373.4659501	0.760773913
66	Brunei Darussalam	1990	149.6243756	0.666081917
66	Brunei Darussalam	1991	147.4367959	0.671233831
66	Brunei Darussalam	1992	144.6335597	0.676702526
66	Brunei Darussalam	1993	141.1772263	0.68217544
66	Brunei Darussalam	1994	135.2046338	0.687394093
66	Brunei Darussalam	1995	129.6710938	0.692471156
66	Brunei Darussalam	1996	123.5250103	0.697602233
66	Brunei Darussalam	1997	120.8351501	0.702944386
66	Brunei Darussalam	1998	121.8100533	0.708630119
66	Brunei Darussalam	1999	120.9505004	0.714851237
66	Brunei Darussalam	2000	116.7385865	0.721552493
66	Brunei Darussalam	2001	113.5259138	0.728535069
66	Brunei Darussalam	2002	112.8674006	0.735343916
66	Brunei Darussalam	2003	113.3028916	0.741578091
66	Brunei Darussalam	2004	111.8266535	0.747311715
66	Brunei Darussalam	2005	104.9925459	0.752481793
66	Brunei Darussalam	2006	100.2438163	0.757318655
66	Brunei Darussalam	2007	99.05251414	0.761643129
66	Brunei Darussalam	2008	97.73232971	0.76571426
66	Brunei Darussalam	2009	98.60808689	0.769601682
66	Brunei Darussalam	2010	99.25029258	0.773219221
66	Brunei Darussalam	2011	99.39040907	0.776631893
66	Brunei Darussalam	2012	99.37579492	0.780216552
66	Brunei Darussalam	2013	104.9508702	0.784017041
66	Brunei Darussalam	2014	106.7603425	0.787733289
66	Brunei Darussalam	2015	107.0049986	0.791367567
66	Brunei Darussalam	2016	110.8602191	0.794980772
66	Brunei Darussalam	2017	106.7627836	0.798309936
66	Brunei Darussalam	2018	97.5402193	0.801436553
66	Brunei Darussalam	2019	94.50658696	0.804499388
66	Brunei Darussalam	2020	85.04563133	0.807424091
66	Brunei Darussalam	2021	84.50476718	0.810234367

67	Japan	1990	66.68591454	0.790253516
67	Japan	1991	64.3205201	0.794616189
67	Japan	1992	62.27402381	0.799156225
67	Japan	1993	59.81920705	0.802972994
67	Japan	1994	55.15307744	0.806689483
67	Japan	1995	51.19529125	0.810510771
67	Japan	1996	47.67860252	0.813870746
67	Japan	1997	45.18744209	0.816675782
67	Japan	1998	44.16539996	0.819016202
67	Japan	1999	43.29173648	0.821066637
67	Japan	2000	40.47671243	0.822938999
67	Japan	2001	38.84743023	0.824723241
67	Japan	2002	37.77521969	0.826936774
67	Japan	2003	37.33089994	0.829392223
67	Japan	2004	36.42661618	0.832037381
67	Japan	2005	36.63175727	0.834288685
67	Japan	2006	35.67109408	0.836222264
67	Japan	2007	34.64156194	0.83829416
67	Japan	2008	33.97686317	0.840301217
67	Japan	2009	33.21747556	0.842133664
67	Japan	2010	32.63430043	0.844361811
67	Japan	2011	32.36480268	0.846750267
67	Japan	2012	31.42398435	0.849061489
67	Japan	2013	30.26475057	0.851356994
67	Japan	2014	29.21630563	0.853692749
67	Japan	2015	28.0328706	0.856097499
67	Japan	2016	27.45727843	0.858698173
67	Japan	2017	26.84334827	0.861393309
67	Japan	2018	26.49432998	0.864237137
67	Japan	2019	26.04394569	0.867148183
67	Japan	2020	25.17262962	0.869106879
67	Japan	2021	25.35552097	0.871241813
68	Republic of Korea	1990	60.14919172	0.692329307
68	Republic of Korea	1991	60.76839017	0.702631128
68	Republic of Korea	1992	61.40794464	0.712440842
68	Republic of Korea	1993	61.96805691	0.72228674
68	Republic of Korea	1994	61.99376103	0.732197906
68	Republic of Korea	1995	60.80788305	0.74194003
68	Republic of Korea	1996	59.90938268	0.75155324
68	Republic of Korea	1997	60.38549131	0.760591683
68	Republic of Korea	1998	60.9064598	0.768281885
68	Republic of Korea	1999	61.1952504	0.776773436
68	Republic of Korea	2000	59.16951195	0.785428821
68	Republic of Korea	2001	56.09378765	0.793496281
68	Republic of Korea	2002	54.97653346	0.801024295
68	Republic of Korea	2003	53.41582941	0.807687786
68	Republic of Korea	2004	51.98678302	0.813720927
68	Republic of Korea	2005	50.08636852	0.8190934
68	Republic of Korea	2006	47.65263948	0.82417982
68	Republic of Korea	2007	44.72181192	0.829127953

68	Republic of Korea	2008	41.71896039	0.833894627
68	Republic of Korea	2009	39.39097182	0.838108331
68	Republic of Korea	2010	38.01056561	0.842511429
68	Republic of Korea	2011	36.98577264	0.846913229
68	Republic of Korea	2012	35.78226526	0.851140263
68	Republic of Korea	2013	34.21989612	0.855441
68	Republic of Korea	2014	32.73483883	0.859665568
68	Republic of Korea	2015	31.78821277	0.863907408
68	Republic of Korea	2016	31.05670472	0.868317824
68	Republic of Korea	2017	29.99618069	0.872617764
68	Republic of Korea	2018	29.52172897	0.876823317
68	Republic of Korea	2019	28.2497558	0.88073151
68	Republic of Korea	2020	27.41949695	0.883717569
68	Republic of Korea	2021	28.2727991	0.886675267
69	Singapore	1990	148.219918	0.686404444
69	Singapore	1991	138.894718	0.695055558
69	Singapore	1992	137.9068193	0.703427834
69	Singapore	1993	134.5698937	0.712315204
69	Singapore	1994	131.8114533	0.721256797
69	Singapore	1995	132.0187521	0.729982231
69	Singapore	1996	132.7612188	0.739028235
69	Singapore	1997	130.4470039	0.747571898
69	Singapore	1998	126.3773822	0.754873204
69	Singapore	1999	123.0646342	0.760831136
69	Singapore	2000	116.7513602	0.76771737
69	Singapore	2001	112.5794938	0.774294657
69	Singapore	2002	105.8272289	0.77973577
69	Singapore	2003	101.9263925	0.784165555
69	Singapore	2004	97.54806376	0.789852774
69	Singapore	2005	89.19286575	0.796455751
69	Singapore	2006	88.30458069	0.803342694
69	Singapore	2007	88.93760193	0.810625304
69	Singapore	2008	86.6651217	0.818124162
69	Singapore	2009	79.48628884	0.824251516
69	Singapore	2010	73.87104022	0.83022216
69	Singapore	2011	66.23040913	0.833829608
69	Singapore	2012	61.56418419	0.837391807
69	Singapore	2013	58.04110107	0.840420178
69	Singapore	2014	56.76920756	0.842894538
69	Singapore	2015	55.6841362	0.845401857
69	Singapore	2016	53.19846714	0.847596999
69	Singapore	2017	53.51815335	0.849051518
69	Singapore	2018	52.84743798	0.850762729
69	Singapore	2019	50.6592933	0.852497338
69	Singapore	2020	49.16984005	0.85429601
69	Singapore	2021	46.73770508	0.856097766
71	Australia	1990	175.0667216	0.725982519
71	Australia	1991	165.0809485	0.729759859
71	Australia	1992	161.4307861	0.733901404
71	Australia	1993	154.1146956	0.738374743

71	Australia	1994	150.6450291	0.742846572
71	Australia	1995	144.0917418	0.747426824
71	Australia	1996	138.6678703	0.751991736
71	Australia	1997	131.5517267	0.756633901
71	Australia	1998	125.0035457	0.761133265
71	Australia	1999	117.6399684	0.765697506
71	Australia	2000	109.7021364	0.770223843
71	Australia	2001	103.4874044	0.774662787
71	Australia	2002	98.62651096	0.77936094
71	Australia	2003	93.28784074	0.783600181
71	Australia	2004	87.66369308	0.787016543
71	Australia	2005	82.39440709	0.789615521
71	Australia	2006	78.22601985	0.791155688
71	Australia	2007	76.55989408	0.792532683
71	Australia	2008	75.08869693	0.7952929
71	Australia	2009	71.29537423	0.799174373
71	Australia	2010	67.56788057	0.803561544
71	Australia	2011	63.80748635	0.80752022
71	Australia	2012	59.34007066	0.811644461
71	Australia	2013	56.86538165	0.816453661
71	Australia	2014	55.70944686	0.82084085
71	Australia	2015	53.89194473	0.824860715
71	Australia	2016	50.74960569	0.828559943
71	Australia	2017	48.11849201	0.831798266
71	Australia	2018	45.35205886	0.835354624
71	Australia	2019	45.92401425	0.839317426
71	Australia	2020	42.79761361	0.842051314
71	Australia	2021	44.0818399	0.844252814
72	New Zealand	1990	185.3683613	0.752321655
72	New Zealand	1991	178.1863279	0.755616091
72	New Zealand	1992	177.0454004	0.759461372
72	New Zealand	1993	171.8487761	0.763634604
72	New Zealand	1994	163.5853881	0.767118658
72	New Zealand	1995	160.9669856	0.769998036
72	New Zealand	1996	153.1487577	0.773575283
72	New Zealand	1997	145.6325653	0.778205776
72	New Zealand	1998	139.4227099	0.781730757
72	New Zealand	1999	136.6191034	0.784428638
72	New Zealand	2000	129.5326122	0.787659557
72	New Zealand	2001	127.2771087	0.791426079
72	New Zealand	2002	123.4270427	0.794519591
72	New Zealand	2003	118.9989294	0.796286883
72	New Zealand	2004	115.3325441	0.798177477
72	New Zealand	2005	108.8730196	0.799107294
72	New Zealand	2006	103.1859188	0.798456866
72	New Zealand	2007	97.8458242	0.798447616
72	New Zealand	2008	93.45112497	0.799669514
72	New Zealand	2009	90.06420314	0.801727535
72	New Zealand	2010	86.6076968	0.805133185
72	New Zealand	2011	84.35795492	0.809760352

72	New Zealand	2012	80.28514818	0.814957564
72	New Zealand	2013	75.97539028	0.82014599
72	New Zealand	2014	73.66414758	0.823908944
72	New Zealand	2015	70.68199699	0.827462493
72	New Zealand	2016	66.86144654	0.831843967
72	New Zealand	2017	66.01778538	0.836301795
72	New Zealand	2018	64.77496637	0.840597863
72	New Zealand	2019	64.02125459	0.844533209
72	New Zealand	2020	59.69649823	0.847398737
72	New Zealand	2021	60.98497599	0.849442499
74	Andorra	1990	83.56688724	0.76146388
74	Andorra	1991	82.62286149	0.764709071
74	Andorra	1992	82.44197598	0.767188282
74	Andorra	1993	81.37120125	0.768953828
74	Andorra	1994	80.15692257	0.770023765
74	Andorra	1995	78.39123158	0.770988683
74	Andorra	1996	77.15942235	0.772403471
74	Andorra	1997	75.59762564	0.774580971
74	Andorra	1998	74.19829477	0.776972578
74	Andorra	1999	72.78205543	0.779629634
74	Andorra	2000	71.49024346	0.781976506
74	Andorra	2001	65.78171247	0.785417035
74	Andorra	2002	61.72236164	0.790158759
74	Andorra	2003	59.49492485	0.797278901
74	Andorra	2004	57.8050079	0.80473198
74	Andorra	2005	55.33308241	0.81221398
74	Andorra	2006	54.13693401	0.81978726
74	Andorra	2007	52.30804839	0.826617254
74	Andorra	2008	53.41761216	0.831821715
74	Andorra	2009	54.34925116	0.835929887
74	Andorra	2010	55.80882933	0.839489966
74	Andorra	2011	55.53422509	0.842794802
74	Andorra	2012	54.81064162	0.845810831
74	Andorra	2013	54.1481896	0.84832991
74	Andorra	2014	53.73650168	0.851010492
74	Andorra	2015	53.20989863	0.853591218
74	Andorra	2016	52.51454202	0.856257053
74	Andorra	2017	51.74542508	0.85947703
74	Andorra	2018	50.96599125	0.862588951
74	Andorra	2019	49.43158823	0.865621024
74	Andorra	2020	40.99648011	0.867449169
74	Andorra	2021	41.0241081	0.869444113
75	Austria	1990	163.4956742	0.749853693
75	Austria	1991	168.3313584	0.751652734
75	Austria	1992	168.7080982	0.754375286
75	Austria	1993	167.881399	0.758804554
75	Austria	1994	166.0563363	0.764704364
75	Austria	1995	164.1460832	0.769827471
75	Austria	1996	164.2266692	0.774446565
75	Austria	1997	162.7124302	0.779232856

75	Austria	1998	162.4990601	0.783521016
75	Austria	1999	157.3712226	0.787451584
75	Austria	2000	145.7983621	0.79184768
75	Austria	2001	138.0849327	0.795770118
75	Austria	2002	132.3862956	0.79915412
75	Austria	2003	125.2998805	0.802043253
75	Austria	2004	117.4880666	0.804889144
75	Austria	2005	111.8690487	0.808419194
75	Austria	2006	108.5621172	0.812064609
75	Austria	2007	106.6937154	0.815480522
75	Austria	2008	103.2513038	0.818793658
75	Austria	2009	102.4792344	0.821541167
75	Austria	2010	98.11525703	0.824500109
75	Austria	2011	94.65032835	0.828001999
75	Austria	2012	92.07826513	0.831113309
75	Austria	2013	89.53251785	0.83367978
75	Austria	2014	85.64129396	0.835838577
75	Austria	2015	84.18730492	0.837579895
75	Austria	2016	80.97389474	0.83988379
75	Austria	2017	78.25783732	0.843173331
75	Austria	2018	77.46346145	0.84661447
75	Austria	2019	73.91978924	0.849862515
75	Austria	2020	73.48964041	0.852020385
75	Austria	2021	68.10999798	0.853837004
76	Belgium	1990	134.4863743	0.737390656
76	Belgium	1991	128.4944951	0.742173074
76	Belgium	1992	123.2693884	0.748012299
76	Belgium	1993	120.1549897	0.753685087
76	Belgium	1994	115.1008243	0.758903638
76	Belgium	1995	112.9115135	0.763170401
76	Belgium	1996	112.2116994	0.766826075
76	Belgium	1997	107.1876419	0.770813953
76	Belgium	1998	107.2634753	0.774228268
76	Belgium	1999	103.4881976	0.77704104
76	Belgium	2000	97.63745283	0.780396331
76	Belgium	2001	93.04242417	0.784713043
76	Belgium	2002	92.60659451	0.788836054
76	Belgium	2003	91.58891952	0.792014384
76	Belgium	2004	85.34387874	0.795224936
76	Belgium	2005	79.74651957	0.798497236
76	Belgium	2006	74.24830769	0.801572763
76	Belgium	2007	71.42558673	0.804612664
76	Belgium	2008	68.83181008	0.807757945
76	Belgium	2009	65.50461893	0.811055825
76	Belgium	2010	61.62711586	0.815030207
76	Belgium	2011	57.81543243	0.819470362
76	Belgium	2012	55.87318285	0.824226016
76	Belgium	2013	53.72917819	0.828619832
76	Belgium	2014	50.64274368	0.832425052
76	Belgium	2015	49.88137003	0.8358784

76	Belgium	2016	47.34576566	0.83946294
76	Belgium	2017	45.42949455	0.843212044
76	Belgium	2018	43.46787002	0.846617625
76	Belgium	2019	41.72472593	0.849498395
76	Belgium	2020	39.57686178	0.851346649
76	Belgium	2021	38.0384461	0.853654016
77	Cyprus	1990	271.5059623	0.648230872
77	Cyprus	1991	275.3835349	0.655726169
77	Cyprus	1992	277.7784459	0.666011849
77	Cyprus	1993	268.8555606	0.677547477
77	Cyprus	1994	248.3511447	0.688829361
77	Cyprus	1995	240.4879433	0.699923898
77	Cyprus	1996	230.5975072	0.709839946
77	Cyprus	1997	232.3340057	0.718864244
77	Cyprus	1998	227.4837667	0.727964382
77	Cyprus	1999	210.8865532	0.736846135
77	Cyprus	2000	198.1609394	0.745199301
77	Cyprus	2001	187.1256422	0.753594717
77	Cyprus	2002	177.9234424	0.761502465
77	Cyprus	2003	168.0948384	0.767896216
77	Cyprus	2004	151.923308	0.773764775
77	Cyprus	2005	142.3232279	0.779031361
77	Cyprus	2006	137.5409913	0.784560965
77	Cyprus	2007	131.9257242	0.792068383
77	Cyprus	2008	126.5051968	0.799454089
77	Cyprus	2009	123.9888269	0.805112085
77	Cyprus	2010	124.0542671	0.809840188
77	Cyprus	2011	119.8845322	0.813726132
77	Cyprus	2012	113.5709805	0.816978525
77	Cyprus	2013	108.1243966	0.819380602
77	Cyprus	2014	104.649495	0.821184302
77	Cyprus	2015	102.3035509	0.822732851
77	Cyprus	2016	97.55261817	0.824283624
77	Cyprus	2017	98.01266334	0.826139878
77	Cyprus	2018	96.23783157	0.828343243
77	Cyprus	2019	95.35444605	0.830980728
77	Cyprus	2020	89.74372667	0.833059523
77	Cyprus	2021	86.95099785	0.835630545
78	Denmark	1990	212.5279999	0.801154655
78	Denmark	1991	204.6056562	0.80454035
78	Denmark	1992	200.7820766	0.808164434
78	Denmark	1993	196.2232033	0.811585713
78	Denmark	1994	185.8122265	0.815138614
78	Denmark	1995	178.4672853	0.819104612
78	Denmark	1996	165.8583394	0.823375243
78	Denmark	1997	153.810838	0.827679979
78	Denmark	1998	142.1657597	0.831683237
78	Denmark	1999	136.315341	0.835381543
78	Denmark	2000	124.1826014	0.839184298
78	Denmark	2001	120.687627	0.84317775

78	Denmark	2002	112.5652647	0.847086357
78	Denmark	2003	105.0205454	0.850441326
78	Denmark	2004	96.79750177	0.853304103
78	Denmark	2005	89.76468781	0.855690732
78	Denmark	2006	84.52157322	0.85803321
78	Denmark	2007	79.85528076	0.859781734
78	Denmark	2008	74.97421549	0.861782061
78	Denmark	2009	71.20441821	0.86394805
78	Denmark	2010	66.32250465	0.866767079
78	Denmark	2011	60.34883824	0.869778682
78	Denmark	2012	56.70447322	0.87251751
78	Denmark	2013	53.36367593	0.874932974
78	Denmark	2014	50.04252194	0.876814932
78	Denmark	2015	48.05141005	0.878543945
78	Denmark	2016	46.54571395	0.880589783
78	Denmark	2017	44.61961797	0.883688487
78	Denmark	2018	43.3985158	0.887529846
78	Denmark	2019	41.85552727	0.891624571
78	Denmark	2020	40.32035454	0.894368297
78	Denmark	2021	39.94567537	0.896424204
79	Finland	1990	215.868055	0.756221509
79	Finland	1991	208.3972475	0.758410687
79	Finland	1992	205.5670554	0.7608041
79	Finland	1993	199.0078821	0.763526604
79	Finland	1994	187.7526525	0.767101901
79	Finland	1995	183.0712975	0.771163742
79	Finland	1996	173.7782344	0.774809059
79	Finland	1997	168.8150267	0.778814215
79	Finland	1998	164.6676738	0.782305059
79	Finland	1999	162.2268276	0.785773988
79	Finland	2000	157.9728152	0.78998523
79	Finland	2001	152.1822711	0.794453835
79	Finland	2002	150.1438423	0.798770274
79	Finland	2003	144.2153434	0.802325431
79	Finland	2004	137.7289857	0.805694227
79	Finland	2005	132.2302863	0.808936787
79	Finland	2006	128.949403	0.812081482
79	Finland	2007	127.0615813	0.815597613
79	Finland	2008	122.8666375	0.818875544
79	Finland	2009	118.920109	0.821457588
79	Finland	2010	115.8409426	0.824844982
79	Finland	2011	110.1489754	0.828270577
79	Finland	2012	106.5737554	0.831434484
79	Finland	2013	101.8233555	0.834425401
79	Finland	2014	98.08458627	0.837431872
79	Finland	2015	94.24185091	0.840878477
79	Finland	2016	90.73120629	0.844622338
79	Finland	2017	87.07522038	0.848586128
79	Finland	2018	83.33757685	0.852202943
79	Finland	2019	80.7908745	0.855365179

79	Finland	2020	78.50125327	0.857655553
79	Finland	2021	77.38263528	0.859831368
80	France	1990	84.2611052	0.730747466
80	France	1991	82.03946856	0.736452382
80	France	1992	79.3624925	0.742871973
80	France	1993	77.79893269	0.748582327
80	France	1994	75.29993581	0.753176132
80	France	1995	74.66932964	0.757125789
80	France	1996	73.67171689	0.760899579
80	France	1997	70.72961707	0.764650672
80	France	1998	68.97338984	0.767852561
80	France	1999	67.1076413	0.770304363
80	France	2000	65.37085626	0.772792033
80	France	2001	63.80562577	0.776225157
80	France	2002	62.38435069	0.780088872
80	France	2003	60.35542545	0.783178757
80	France	2004	55.82511883	0.786084655
80	France	2005	53.82162666	0.789042086
80	France	2006	51.30868245	0.79232192
80	France	2007	49.35488472	0.795557651
80	France	2008	47.73732065	0.79817743
80	France	2009	45.74430054	0.80050867
80	France	2010	43.49113515	0.803240247
80	France	2011	41.5127029	0.806378738
80	France	2012	40.24316389	0.809615389
80	France	2013	38.23778374	0.812984015
80	France	2014	36.28201494	0.816551448
80	France	2015	36.087736	0.820238385
80	France	2016	34.80636855	0.823730929
80	France	2017	33.99216535	0.827098441
80	France	2018	32.92034605	0.83044859
80	France	2019	31.95159869	0.833772805
80	France	2020	31.11130085	0.836049443
80	France	2021	29.87362192	0.838364875
81	Germany	1990	192.3990325	0.817077666
81	Germany	1991	188.5433444	0.823339898
81	Germany	1992	182.3419276	0.828323503
81	Germany	1993	179.9982042	0.832286809
81	Germany	1994	172.724942	0.835561963
81	Germany	1995	164.9190195	0.837643152
81	Germany	1996	160.7278278	0.839188129
81	Germany	1997	157.1551663	0.841385903
81	Germany	1998	153.917688	0.843854978
81	Germany	1999	146.9604	0.846232398
81	Germany	2000	136.2797115	0.848925756
81	Germany	2001	131.701316	0.852070413
81	Germany	2002	130.1294643	0.855066793
81	Germany	2003	126.6900113	0.85767579
81	Germany	2004	115.7362074	0.860240333
81	Germany	2005	108.374558	0.862894178

81	Germany	2006	104.2821717	0.865873707
81	Germany	2007	101.5633029	0.86914044
81	Germany	2008	97.68876428	0.872552849
81	Germany	2009	93.07448023	0.875087911
81	Germany	2010	87.68120356	0.87794671
81	Germany	2011	84.4435176	0.881027508
81	Germany	2012	83.25393811	0.883656408
81	Germany	2013	82.23940578	0.885926953
81	Germany	2014	78.00603931	0.887651614
81	Germany	2015	77.38627122	0.888867854
81	Germany	2016	75.79879394	0.890896369
81	Germany	2017	72.7529172	0.894050472
81	Germany	2018	70.76755962	0.896975545
81	Germany	2019	66.9950587	0.899703158
81	Germany	2020	65.61230691	0.901438614
81	Germany	2021	63.67090624	0.902957091
82	Greece	1990	142.0443403	0.674186465
82	Greece	1991	139.0833569	0.68031648
82	Greece	1992	136.9910723	0.687055389
82	Greece	1993	133.7328712	0.693328926
82	Greece	1994	133.2648742	0.699153022
82	Greece	1995	134.2536131	0.704862873
82	Greece	1996	133.8212365	0.710139336
82	Greece	1997	130.8182675	0.715687268
82	Greece	1998	128.7809869	0.721593982
82	Greece	1999	127.3529743	0.727529455
82	Greece	2000	124.0292535	0.73271054
82	Greece	2001	121.3765635	0.737154008
82	Greece	2002	120.3597531	0.741945221
82	Greece	2003	121.4022942	0.747185956
82	Greece	2004	117.7964451	0.752418607
82	Greece	2005	111.6825	0.756384396
82	Greece	2006	109.9690012	0.760209696
82	Greece	2007	120.6143366	0.763927173
82	Greece	2008	104.980256	0.767527658
82	Greece	2009	112.4429812	0.771247356
82	Greece	2010	111.3588003	0.775155815
82	Greece	2011	108.7305161	0.778525867
82	Greece	2012	104.1726274	0.780789483
82	Greece	2013	92.65764855	0.781949052
82	Greece	2014	79.60173001	0.782442013
82	Greece	2015	75.1386948	0.782489523
82	Greece	2016	72.54529052	0.782798443
82	Greece	2017	73.24238619	0.784108594
82	Greece	2018	72.38944933	0.785999753
82	Greece	2019	72.6928206	0.788138138
82	Greece	2020	72.92777679	0.78963222
82	Greece	2021	72.90311487	0.791854408
83	Iceland	1990	166.2021545	0.764212517
83	Iceland	1991	166.1820633	0.769027393

83	Iceland	1992	162.736167	0.772706415
83	Iceland	1993	156.5596487	0.776389423
83	Iceland	1994	155.4283619	0.779967961
83	Iceland	1995	150.01343	0.782740057
83	Iceland	1996	145.9452626	0.78535642
83	Iceland	1997	141.9044779	0.788528335
83	Iceland	1998	137.2082343	0.792626368
83	Iceland	1999	129.8625147	0.797368808
83	Iceland	2000	121.0166444	0.80303033
83	Iceland	2001	118.6378989	0.808965786
83	Iceland	2002	117.4138879	0.813400392
83	Iceland	2003	114.0581582	0.816597067
83	Iceland	2004	110.3832436	0.819528087
83	Iceland	2005	103.1966444	0.822766536
83	Iceland	2006	101.7017289	0.826244743
83	Iceland	2007	97.37727263	0.83028989
83	Iceland	2008	96.28899128	0.834622678
83	Iceland	2009	93.34111131	0.838121013
83	Iceland	2010	91.61681682	0.841384642
83	Iceland	2011	89.13262093	0.84461993
83	Iceland	2012	85.28849495	0.847299444
83	Iceland	2013	82.4769469	0.850173948
83	Iceland	2014	78.43153856	0.853558697
83	Iceland	2015	74.97729369	0.857724976
83	Iceland	2016	70.94432379	0.861844467
83	Iceland	2017	67.80753344	0.865702891
83	Iceland	2018	64.3438655	0.869219822
83	Iceland	2019	62.60818138	0.87242558
83	Iceland	2020	58.87277459	0.874323153
83	Iceland	2021	57.95472307	0.87636168
84	Ireland	1990	227.0070586	0.719891819
84	Ireland	1991	218.2342061	0.725195019
84	Ireland	1992	212.5671454	0.731918691
84	Ireland	1993	212.4893278	0.738384074
84	Ireland	1994	209.0238148	0.744011728
84	Ireland	1995	206.2133299	0.748921575
84	Ireland	1996	197.468227	0.754010124
84	Ireland	1997	188.9476615	0.759970384
84	Ireland	1998	183.7347898	0.766488721
84	Ireland	1999	176.2248144	0.773538834
84	Ireland	2000	163.8026632	0.780190655
84	Ireland	2001	150.0973922	0.786353741
84	Ireland	2002	142.4279197	0.793010606
84	Ireland	2003	132.7816487	0.800005865
84	Ireland	2004	126.396236	0.80685314
84	Ireland	2005	117.8008852	0.811713818
84	Ireland	2006	114.0307344	0.814505479
84	Ireland	2007	107.5409473	0.816931026
84	Ireland	2008	100.954275	0.819932128
84	Ireland	2009	95.50981496	0.823929666

84	Ireland	2010	87.70173595	0.828103848
84	Ireland	2011	86.22210919	0.831804792
84	Ireland	2012	83.47463683	0.835325359
84	Ireland	2013	80.03429341	0.838644433
84	Ireland	2014	75.34672133	0.842082115
84	Ireland	2015	74.24362887	0.847437887
84	Ireland	2016	72.98187172	0.852642502
84	Ireland	2017	68.6313211	0.857816164
84	Ireland	2018	66.14849594	0.863925284
84	Ireland	2019	62.7963725	0.869412503
84	Ireland	2020	57.45510555	0.871958822
84	Ireland	2021	52.76537497	0.87375385
85	Israel	1990	177.2991947	0.709178347
85	Israel	1991	181.7396536	0.713371633
85	Israel	1992	182.6222556	0.717897923
85	Israel	1993	176.4355285	0.722250176
85	Israel	1994	172.5435396	0.726654736
85	Israel	1995	154.1494239	0.731127859
85	Israel	1996	129.6227449	0.734714026
85	Israel	1997	124.7991808	0.738015277
85	Israel	1998	126.1063959	0.74192719
85	Israel	1999	113.6720852	0.745443338
85	Israel	2000	103.3055359	0.749184084
85	Israel	2001	96.01603189	0.752834297
85	Israel	2002	91.4143641	0.755988076
85	Israel	2003	87.07061885	0.758875409
85	Israel	2004	80.09536778	0.762213291
85	Israel	2005	76.03166899	0.765543461
85	Israel	2006	73.05222013	0.768733898
85	Israel	2007	71.5032563	0.770756143
85	Israel	2008	65.45155642	0.772011615
85	Israel	2009	59.67657363	0.773303913
85	Israel	2010	55.32184821	0.77525953
85	Israel	2011	53.45821131	0.777649669
85	Israel	2012	49.62872912	0.780403415
85	Israel	2013	47.41541317	0.783267167
85	Israel	2014	45.05277652	0.786151084
85	Israel	2015	44.02523912	0.788874457
85	Israel	2016	42.30626544	0.791772445
85	Israel	2017	40.41470504	0.795173437
85	Israel	2018	38.17919971	0.799124994
85	Israel	2019	37.44904556	0.803232991
85	Israel	2020	35.77789759	0.806351589
85	Israel	2021	34.77493235	0.809011652
86	Italy	1990	108.5007796	0.706255224
86	Italy	1991	106.9582909	0.711457349
86	Italy	1992	103.9291844	0.716649746
86	Italy	1993	102.9285599	0.721720992
86	Italy	1994	101.487352	0.726870913
86	Italy	1995	99.10177908	0.731644082

86	Italy	1996	95.78416297	0.73605087
86	Italy	1997	94.07659309	0.739972336
86	Italy	1998	94.22305482	0.743101596
86	Italy	1999	90.62861851	0.746074084
86	Italy	2000	85.25204475	0.749675986
86	Italy	2001	80.74164296	0.753554839
86	Italy	2002	80.25118139	0.757191479
86	Italy	2003	81.10880334	0.760093744
86	Italy	2004	76.44287494	0.762787749
86	Italy	2005	74.22712456	0.765689914
86	Italy	2006	69.80803125	0.768402408
86	Italy	2007	67.55661551	0.77069838
86	Italy	2008	66.01858121	0.77306847
86	Italy	2009	64.08350818	0.775499262
86	Italy	2010	61.5619064	0.778109206
86	Italy	2011	60.01803835	0.780703468
86	Italy	2012	58.32142854	0.783090981
86	Italy	2013	55.84544305	0.785356877
86	Italy	2014	54.00157849	0.787533956
86	Italy	2015	54.50791381	0.789902119
86	Italy	2016	50.78835132	0.792477244
86	Italy	2017	49.20521535	0.795311186
86	Italy	2018	46.68030046	0.79836269
86	Italy	2019	45.26163555	0.801530021
86	Italy	2020	45.73055241	0.80363568
86	Italy	2021	44.27217652	0.805773534
87	Luxembourg	1990	153.8273444	0.781051609
87	Luxembourg	1991	150.0317696	0.785961442
87	Luxembourg	1992	143.8514732	0.789053364
87	Luxembourg	1993	141.27623	0.792843836
87	Luxembourg	1994	137.3401369	0.797824008
87	Luxembourg	1995	131.7680896	0.80268247
87	Luxembourg	1996	132.2461999	0.806516138
87	Luxembourg	1997	126.4907239	0.810235288
87	Luxembourg	1998	118.3270157	0.813889026
87	Luxembourg	1999	108.0653393	0.817570995
87	Luxembourg	2000	103.6986054	0.822006568
87	Luxembourg	2001	99.91872097	0.825312816
87	Luxembourg	2002	99.06741324	0.828223491
87	Luxembourg	2003	100.8470688	0.830983946
87	Luxembourg	2004	95.5774497	0.833672567
87	Luxembourg	2005	90.96096369	0.836675264
87	Luxembourg	2006	87.74747905	0.840574453
87	Luxembourg	2007	80.80316768	0.844707006
87	Luxembourg	2008	75.65514859	0.847978883
87	Luxembourg	2009	68.69269522	0.850688725
87	Luxembourg	2010	64.34831616	0.853258277
87	Luxembourg	2011	63.52271179	0.856216834
87	Luxembourg	2012	61.40216047	0.859789031
87	Luxembourg	2013	60.3145053	0.863368792

87	Luxembourg	2014	58.5094281	0.866693556
87	Luxembourg	2015	57.25844718	0.870112937
87	Luxembourg	2016	57.30702509	0.87342088
87	Luxembourg	2017	53.89533477	0.876347684
87	Luxembourg	2018	52.95508725	0.878637895
87	Luxembourg	2019	50.55591154	0.880593895
87	Luxembourg	2020	47.61669616	0.882495316
87	Luxembourg	2021	45.69920375	0.884428955
88	Malta	1990	207.7052486	0.656504584
88	Malta	1991	197.5553282	0.661112691
88	Malta	1992	190.2383653	0.666324648
88	Malta	1993	179.3951762	0.672120257
88	Malta	1994	170.3734583	0.677847314
88	Malta	1995	166.132587	0.682617704
88	Malta	1996	165.1559781	0.686623261
88	Malta	1997	165.3654112	0.691756406
88	Malta	1998	159.6726589	0.698095287
88	Malta	1999	164.3102753	0.704734545
88	Malta	2000	157.0010486	0.711805254
88	Malta	2001	149.00671	0.717803038
88	Malta	2002	144.1734849	0.722790275
88	Malta	2003	139.4077247	0.727511133
88	Malta	2004	131.9541137	0.731933853
88	Malta	2005	131.5683925	0.736188747
88	Malta	2006	124.4721467	0.740140181
88	Malta	2007	114.9728698	0.744237812
88	Malta	2008	109.4724624	0.748427095
88	Malta	2009	105.781248	0.752171059
88	Malta	2010	105.4268882	0.7562505
88	Malta	2011	110.2444691	0.760310547
88	Malta	2012	112.1126052	0.76453788
88	Malta	2013	100.8724152	0.76861614
88	Malta	2014	92.78152017	0.772728741
88	Malta	2015	86.62715122	0.777276905
88	Malta	2016	79.61779783	0.78167341
88	Malta	2017	76.98696991	0.786410184
88	Malta	2018	75.34395892	0.790965618
88	Malta	2019	73.39746779	0.795197846
88	Malta	2020	67.32810908	0.798375166
88	Malta	2021	71.18744991	0.801585034
89	Netherlands	1990	141.6293733	0.794612123
89	Netherlands	1991	136.8024373	0.799285437
89	Netherlands	1992	133.089065	0.803965978
89	Netherlands	1993	131.845601	0.808278967
89	Netherlands	1994	125.9663896	0.812530216
89	Netherlands	1995	122.9112951	0.816662769
89	Netherlands	1996	119.3788759	0.81998422
89	Netherlands	1997	113.7723479	0.82288177
89	Netherlands	1998	109.7968025	0.825882234
89	Netherlands	1999	105.2272063	0.828693827

89	Netherlands	2000	98.32244752	0.831913984
89	Netherlands	2001	92.73899805	0.835518148
89	Netherlands	2002	88.34439364	0.838676077
89	Netherlands	2003	83.83897986	0.841639477
89	Netherlands	2004	77.05767261	0.844807348
89	Netherlands	2005	71.82609332	0.84793419
89	Netherlands	2006	66.87870801	0.850798808
89	Netherlands	2007	62.23396813	0.853595391
89	Netherlands	2008	58.37910224	0.856368467
89	Netherlands	2009	54.51720668	0.858600459
89	Netherlands	2010	51.78547509	0.861255298
89	Netherlands	2011	48.82644123	0.864246678
89	Netherlands	2012	47.18125494	0.866950626
89	Netherlands	2013	45.62625106	0.869402979
89	Netherlands	2014	43.85946033	0.871848914
89	Netherlands	2015	43.15416678	0.874402841
89	Netherlands	2016	41.9883789	0.876784052
89	Netherlands	2017	40.32925332	0.879300152
89	Netherlands	2018	39.35304361	0.881900218
89	Netherlands	2019	38.03504407	0.88464384
89	Netherlands	2020	37.60496702	0.886558566
89	Netherlands	2021	37.41670017	0.888464256
90	Norway	1990	175.8160791	0.795887277
90	Norway	1991	170.9268896	0.800468886
90	Norway	1992	165.7541172	0.805437473
90	Norway	1993	159.5600343	0.810565689
90	Norway	1994	154.1811051	0.815414668
90	Norway	1995	150.6670013	0.820223522
90	Norway	1996	144.1447068	0.825701668
90	Norway	1997	140.2188378	0.83146877
90	Norway	1998	137.8197889	0.836562809
90	Norway	1999	132.9521137	0.841130237
90	Norway	2000	125.1400545	0.846475451
90	Norway	2001	118.9055655	0.852111936
90	Norway	2002	114.444138	0.856757888
90	Norway	2003	106.5396266	0.860302576
90	Norway	2004	97.966795	0.863468908
90	Norway	2005	90.02177025	0.866217488
90	Norway	2006	85.37277692	0.868588435
90	Norway	2007	83.12999186	0.87092967
90	Norway	2008	79.65372952	0.873745121
90	Norway	2009	75.62481006	0.876582817
90	Norway	2010	71.75065533	0.880378381
90	Norway	2011	69.53213565	0.884351667
90	Norway	2012	65.89762693	0.888160475
90	Norway	2013	61.87703713	0.891989618
90	Norway	2014	57.40122375	0.895499546
90	Norway	2015	54.73697147	0.898912644
90	Norway	2016	51.08361427	0.902584187
90	Norway	2017	46.78299412	0.906044049

90	Norway	2018	44.62433791	0.909261782
90	Norway	2019	41.98641438	0.912278761
90	Norway	2020	40.49308229	0.91452992
90	Norway	2021	40.87629853	0.91613281
91	Portugal	1990	116.8723957	0.599777757
91	Portugal	1991	115.0911118	0.607269449
91	Portugal	1992	111.9933054	0.61480808
91	Portugal	1993	112.4472921	0.622101177
91	Portugal	1994	105.6282361	0.629306387
91	Portugal	1995	102.7168776	0.635498761
91	Portugal	1996	101.3938461	0.640864526
91	Portugal	1997	97.96600978	0.646192904
91	Portugal	1998	96.15533223	0.651335994
91	Portugal	1999	93.9883499	0.656120667
91	Portugal	2000	88.19209936	0.661610852
91	Portugal	2001	84.892321	0.667247151
91	Portugal	2002	82.08334689	0.67258394
91	Portugal	2003	78.63408643	0.677663412
91	Portugal	2004	72.60547892	0.682352926
91	Portugal	2005	67.75318206	0.686887105
91	Portugal	2006	62.38431963	0.691158795
91	Portugal	2007	58.25779193	0.694974853
91	Portugal	2008	54.54792549	0.698604599
91	Portugal	2009	51.32019547	0.702223399
91	Portugal	2010	48.3768077	0.706563555
91	Portugal	2011	45.26616406	0.711262893
91	Portugal	2012	43.40306875	0.715804084
91	Portugal	2013	42.69784148	0.719836991
91	Portugal	2014	43.20424262	0.723021282
91	Portugal	2015	42.27383044	0.72575622
91	Portugal	2016	41.56090109	0.728300849
91	Portugal	2017	40.61032898	0.731225535
91	Portugal	2018	39.46008931	0.734402947
91	Portugal	2019	38.59245946	0.738090481
91	Portugal	2020	37.30870469	0.741037384
91	Portugal	2021	36.80043515	0.744151851
92	Spain	1990	98.16921193	0.636673166
92	Spain	1991	96.18823983	0.644130745
92	Spain	1992	92.97795206	0.651597597
92	Spain	1993	91.03501062	0.658637569
92	Spain	1994	88.22999241	0.665321549
92	Spain	1995	87.2065665	0.671576034
92	Spain	1996	86.01167873	0.677088943
92	Spain	1997	82.67450974	0.682429497
92	Spain	1998	81.48207594	0.687490717
92	Spain	1999	80.38790256	0.692245197
92	Spain	2000	75.05423278	0.697056031
92	Spain	2001	71.36632271	0.70172607
92	Spain	2002	69.90509019	0.706309655
92	Spain	2003	69.03981623	0.710678584

92	Spain	2004	66.00065484	0.71488623
92	Spain	2005	64.22520447	0.718717289
92	Spain	2006	60.64071579	0.721970128
92	Spain	2007	58.49048452	0.725211443
92	Spain	2008	55.80694679	0.729411041
92	Spain	2009	52.44142061	0.733956055
92	Spain	2010	49.62296299	0.738352055
92	Spain	2011	47.66035581	0.74209645
92	Spain	2012	46.0355971	0.745181867
92	Spain	2013	43.54180514	0.747651982
92	Spain	2014	41.96521171	0.749800929
92	Spain	2015	42.19318915	0.752269823
92	Spain	2016	40.49256587	0.754757939
92	Spain	2017	39.76057255	0.757663276
92	Spain	2018	39.17396001	0.760659575
92	Spain	2019	37.67271762	0.763942918
92	Spain	2020	36.57110109	0.766506025
92	Spain	2021	35.36782589	0.769283698
93	Sweden	1990	181.5805175	0.785535792
93	Sweden	1991	177.0624387	0.790096441
93	Sweden	1992	171.2130425	0.795431458
93	Sweden	1993	167.6316268	0.801009522
93	Sweden	1994	158.2854402	0.806700264
93	Sweden	1995	154.6129008	0.812244654
93	Sweden	1996	149.2298866	0.817227025
93	Sweden	1997	143.9771233	0.821694059
93	Sweden	1998	140.4457036	0.825731995
93	Sweden	1999	136.1150226	0.829487099
93	Sweden	2000	128.6248329	0.833028112
93	Sweden	2001	124.3015644	0.835956398
93	Sweden	2002	120.9787937	0.838741527
93	Sweden	2003	115.5992355	0.841663138
93	Sweden	2004	109.9663836	0.844506882
93	Sweden	2005	104.8856091	0.846876344
93	Sweden	2006	102.4541994	0.849115871
93	Sweden	2007	98.87939412	0.851420847
93	Sweden	2008	94.71643895	0.853705851
93	Sweden	2009	90.63964907	0.855702748
93	Sweden	2010	86.89798025	0.858727941
93	Sweden	2011	83.73836698	0.861892379
93	Sweden	2012	80.23958491	0.864706672
93	Sweden	2013	75.61722188	0.867383768
93	Sweden	2014	71.74710938	0.869923325
93	Sweden	2015	68.59801485	0.872388515
93	Sweden	2016	65.76190249	0.874661655
93	Sweden	2017	62.57346452	0.877120611
93	Sweden	2018	59.61906331	0.879888912
93	Sweden	2019	55.45691325	0.882984116
93	Sweden	2020	54.11389324	0.88506201
93	Sweden	2021	51.79852619	0.886880299

94	Switzerland	1990	141.4600181	0.862766844
94	Switzerland	1991	139.1632764	0.864796867
94	Switzerland	1992	134.0188458	0.867736079
94	Switzerland	1993	129.2117515	0.870863558
94	Switzerland	1994	125.1969549	0.873140871
94	Switzerland	1995	122.1153356	0.874835331
94	Switzerland	1996	117.0271865	0.876640557
94	Switzerland	1997	114.3028626	0.878485545
94	Switzerland	1998	110.9491524	0.880035152
94	Switzerland	1999	106.1700567	0.882045586
94	Switzerland	2000	99.91660534	0.885243531
94	Switzerland	2001	94.42859703	0.888445951
94	Switzerland	2002	91.17467789	0.890902811
94	Switzerland	2003	87.70976464	0.892780807
94	Switzerland	2004	82.51184642	0.894909812
94	Switzerland	2005	79.40506653	0.89727263
94	Switzerland	2006	75.71604686	0.899962305
94	Switzerland	2007	72.21155083	0.902913413
94	Switzerland	2008	69.10030426	0.905778454
94	Switzerland	2009	66.24040172	0.908049998
94	Switzerland	2010	62.17829906	0.910530522
94	Switzerland	2011	58.85608984	0.913276573
94	Switzerland	2012	57.61795945	0.915714893
94	Switzerland	2013	54.97457533	0.917955006
94	Switzerland	2014	51.81580861	0.919994182
94	Switzerland	2015	49.74330039	0.921832991
94	Switzerland	2016	47.15867388	0.923930598
94	Switzerland	2017	45.65013501	0.926202291
94	Switzerland	2018	44.67958683	0.92855059
94	Switzerland	2019	42.24176956	0.930682102
94	Switzerland	2020	42.43184574	0.932027645
94	Switzerland	2021	39.95723901	0.933059111
95	United Kingdom	1990	196.4944724	0.744334126
95	United Kingdom	1991	191.9265225	0.749148231
95	United Kingdom	1992	187.0808588	0.754556689
95	United Kingdom	1993	182.1465249	0.76015435
95	United Kingdom	1994	171.6504895	0.7651646
95	United Kingdom	1995	166.2185912	0.768656864
95	United Kingdom	1996	159.66923	0.771576484
95	United Kingdom	1997	152.4473563	0.775050122
95	United Kingdom	1998	147.3524142	0.779057165
95	United Kingdom	1999	140.933079	0.784036475
95	United Kingdom	2000	131.6631949	0.78921896
95	United Kingdom	2001	125.5431958	0.793513165
95	United Kingdom	2002	120.9349222	0.79665556
95	United Kingdom	2003	115.0607934	0.799186111
95	United Kingdom	2004	106.413087	0.80195087
95	United Kingdom	2005	99.32948693	0.804489194
95	United Kingdom	2006	92.75952533	0.806817046
95	United Kingdom	2007	87.71741071	0.809352772

95	United Kingdom	2008	82.94655428	0.812039287
95	United Kingdom	2009	77.20820796	0.814793642
95	United Kingdom	2010	72.75178438	0.818033994
95	United Kingdom	2011	68.41660125	0.821718995
95	United Kingdom	2012	66.46641189	0.826453754
95	United Kingdom	2013	65.02135254	0.831508668
95	United Kingdom	2014	62.0804317	0.835516642
95	United Kingdom	2015	61.11015383	0.838951919
95	United Kingdom	2016	59.46630569	0.842460233
95	United Kingdom	2017	58.06466099	0.846263618
95	United Kingdom	2018	56.73801681	0.849969788
95	United Kingdom	2019	54.88927665	0.854130573
95	United Kingdom	2020	52.09205792	0.85692029
95	United Kingdom	2021	52.10890635	0.859000182
97	Argentina	1990	158.8035129	0.587397284
97	Argentina	1991	148.2647237	0.59170754
97	Argentina	1992	144.953669	0.597996324
97	Argentina	1993	138.4812256	0.603739312
97	Argentina	1994	128.1939957	0.60978733
97	Argentina	1995	126.0733697	0.614473706
97	Argentina	1996	121.4158896	0.618942624
97	Argentina	1997	115.5104128	0.623934409
97	Argentina	1998	115.269376	0.628723436
97	Argentina	1999	114.2223738	0.633175129
97	Argentina	2000	104.3148949	0.638194472
97	Argentina	2001	100.492516	0.642175705
97	Argentina	2002	99.08811701	0.644472507
97	Argentina	2003	96.05222886	0.645906577
97	Argentina	2004	90.56157262	0.648296828
97	Argentina	2005	87.69542789	0.65336883
97	Argentina	2006	86.30551393	0.6574459
97	Argentina	2007	87.10119484	0.660187576
97	Argentina	2008	82.88383673	0.663079235
97	Argentina	2009	81.83289358	0.665911944
97	Argentina	2010	82.04130998	0.66992588
97	Argentina	2011	81.25607315	0.675242697
97	Argentina	2012	79.5164611	0.67921824
97	Argentina	2013	77.06135576	0.681633215
97	Argentina	2014	73.45476649	0.684974509
97	Argentina	2015	73.50034586	0.691710126
97	Argentina	2016	75.49421039	0.698618031
97	Argentina	2017	72.35911586	0.705135529
97	Argentina	2018	71.03315976	0.712888693
97	Argentina	2019	69.23351355	0.719002843
97	Argentina	2020	66.28042764	0.721294303
97	Argentina	2021	60.79185483	0.723122973
98	Chile	1990	118.4788401	0.5864951
98	Chile	1991	109.6707404	0.59300658
98	Chile	1992	103.9539144	0.599106083
98	Chile	1993	101.028257	0.604828509

98	Chile	1994	97.02078084	0.610673933
98	Chile	1995	93.53432684	0.617234677
98	Chile	1996	88.73328249	0.62369446
98	Chile	1997	83.90775637	0.630540085
98	Chile	1998	81.74052711	0.638082758
98	Chile	1999	79.03682898	0.645928084
98	Chile	2000	74.02206124	0.652989028
98	Chile	2001	72.07719114	0.66026551
98	Chile	2002	68.98880458	0.667888408
98	Chile	2003	68.27803165	0.673445927
98	Chile	2004	66.23329182	0.678258212
98	Chile	2005	63.60717428	0.682589191
98	Chile	2006	61.4337264	0.685892748
98	Chile	2007	59.80638882	0.688930323
98	Chile	2008	55.29851221	0.692359787
98	Chile	2009	53.7207019	0.696602141
98	Chile	2010	53.3147278	0.702304704
98	Chile	2011	50.586674	0.708597936
98	Chile	2012	50.06032392	0.714743477
98	Chile	2013	49.03030437	0.720448781
98	Chile	2014	47.90536869	0.727275927
98	Chile	2015	47.41828041	0.736098082
98	Chile	2016	45.52666616	0.744972224
98	Chile	2017	44.17793985	0.752058077
98	Chile	2018	42.58205499	0.758625714
98	Chile	2019	41.91628855	0.765120323
98	Chile	2020	38.72839699	0.769213676
98	Chile	2021	39.05029328	0.771514716
99	Uruguay	1990	152.2769373	0.581921855
99	Uruguay	1991	147.3179397	0.586156118
99	Uruguay	1992	141.1802983	0.589822355
99	Uruguay	1993	138.9094235	0.592295025
99	Uruguay	1994	131.1724814	0.595188424
99	Uruguay	1995	126.5423802	0.596825409
99	Uruguay	1996	119.1019836	0.599775104
99	Uruguay	1997	108.7424794	0.605665051
99	Uruguay	1998	108.1833917	0.612700815
99	Uruguay	1999	105.571685	0.619009271
99	Uruguay	2000	97.27429239	0.623208291
99	Uruguay	2001	93.55238905	0.625796068
99	Uruguay	2002	92.16595881	0.6282858
99	Uruguay	2003	90.78402043	0.631437774
99	Uruguay	2004	87.86676727	0.634216906
99	Uruguay	2005	84.24221312	0.63697577
99	Uruguay	2006	81.11021976	0.640590696
99	Uruguay	2007	83.08036949	0.646331714
99	Uruguay	2008	77.62673875	0.653525522
99	Uruguay	2009	73.87868671	0.660321135
99	Uruguay	2010	72.85510764	0.666099135
99	Uruguay	2011	73.84914415	0.667079733

99	Uruguay	2012	71.93925816	0.665802192
99	Uruguay	2013	67.97290823	0.668228361
99	Uruguay	2014	65.62305915	0.67333426
99	Uruguay	2015	65.72786906	0.680595506
99	Uruguay	2016	64.98142169	0.689816733
99	Uruguay	2017	62.85092211	0.699189456
99	Uruguay	2018	62.46724913	0.70690493
99	Uruguay	2019	62.18273881	0.712536588
99	Uruguay	2020	57.9168549	0.716169766
99	Uruguay	2021	60.54835406	0.719283445
101	Canada	1990	156.6586063	0.781977864
101	Canada	1991	150.6095604	0.78415936
101	Canada	1992	146.0432204	0.787030537
101	Canada	1993	144.0258715	0.789716593
101	Canada	1994	141.1632178	0.792598518
101	Canada	1995	138.3594137	0.796521166
101	Canada	1996	134.517247	0.80106497
101	Canada	1997	130.5661183	0.804833356
101	Canada	1998	125.9953901	0.808135441
101	Canada	1999	121.2101999	0.81226594
101	Canada	2000	115.1072855	0.816685059
101	Canada	2001	109.2159964	0.82069625
101	Canada	2002	104.4318047	0.824402272
101	Canada	2003	99.52374528	0.827801096
101	Canada	2004	94.34808737	0.83092811
101	Canada	2005	90.20351619	0.833288294
101	Canada	2006	84.46630463	0.83491566
101	Canada	2007	80.79523593	0.836625096
101	Canada	2008	77.79021914	0.838913685
101	Canada	2009	73.70343237	0.841583671
101	Canada	2010	70.11316574	0.844853489
101	Canada	2011	66.14968723	0.848017833
101	Canada	2012	64.23311857	0.850978048
101	Canada	2013	62.6613285	0.853844945
101	Canada	2014	61.87038204	0.856759934
101	Canada	2015	58.51930249	0.859532904
101	Canada	2016	57.96055576	0.862174226
101	Canada	2017	57.49667714	0.86505383
101	Canada	2018	56.16762987	0.867905541
101	Canada	2019	54.47112992	0.870554479
101	Canada	2020	52.50760433	0.871992631
101	Canada	2021	51.11770607	0.87317068
102	United States of America	1990	179.8318273	0.76364769
102	United States of America	1991	174.523265	0.766044295
102	United States of America	1992	169.8506389	0.769595492
102	United States of America	1993	169.6933921	0.773103517
102	United States of America	1994	166.5191171	0.776368588
102	United States of America	1995	163.3396138	0.779410254
102	United States of America	1996	157.9608863	0.78193477
102	United States of America	1997	152.6364902	0.783979278

102	United States of America	1998	149.4235199	0.786076332
102	United States of America	1999	150.1968032	0.788544535
102	United States of America	2000	144.7195535	0.79231665
102	United States of America	2001	140.1998048	0.796676793
102	United States of America	2002	135.9941709	0.800403318
102	United States of America	2003	130.1497962	0.803228025
102	United States of America	2004	122.7210957	0.805304692
102	United States of America	2005	118.3486099	0.80603308
102	United States of America	2006	112.7337914	0.806783583
102	United States of America	2007	106.7276187	0.80968805
102	United States of America	2008	103.168257	0.814577102
102	United States of America	2009	98.70632885	0.820294574
102	United States of America	2010	94.48585918	0.825884658
102	United States of America	2011	92.48277862	0.830266388
102	United States of America	2012	90.21469084	0.833958192
102	United States of America	2013	88.33243774	0.837412988
102	United States of America	2014	86.58905694	0.840746528
102	United States of America	2015	85.26851183	0.844370336
102	United States of America	2016	84.33991701	0.848015521
102	United States of America	2017	82.87635557	0.851630299
102	United States of America	2018	80.68599872	0.855049193
102	United States of America	2019	78.78627922	0.858578065
102	United States of America	2020	77.62632799	0.860792773
102	United States of America	2021	78.92105918	0.862448354
105	Antigua and Barbuda	1990	127.1593069	0.612104591
105	Antigua and Barbuda	1991	129.6000931	0.618817129
105	Antigua and Barbuda	1992	131.2457927	0.624695818
105	Antigua and Barbuda	1993	141.5954178	0.630075854
105	Antigua and Barbuda	1994	139.1382154	0.634768955
105	Antigua and Barbuda	1995	141.9282901	0.638008629
105	Antigua and Barbuda	1996	132.3462086	0.641647192
105	Antigua and Barbuda	1997	124.7599427	0.645895591
105	Antigua and Barbuda	1998	118.7445353	0.650614287
105	Antigua and Barbuda	1999	113.7107922	0.655534994
105	Antigua and Barbuda	2000	104.4809359	0.660819064
105	Antigua and Barbuda	2001	100.1725976	0.665620632
105	Antigua and Barbuda	2002	96.89775489	0.670360801
105	Antigua and Barbuda	2003	97.26872397	0.67531149
105	Antigua and Barbuda	2004	94.98514407	0.680412429
105	Antigua and Barbuda	2005	96.57462395	0.685486437
105	Antigua and Barbuda	2006	93.60522282	0.690826021
105	Antigua and Barbuda	2007	102.3030079	0.696360039
105	Antigua and Barbuda	2008	99.09282438	0.702175362
105	Antigua and Barbuda	2009	96.17108341	0.707128564
105	Antigua and Barbuda	2010	93.65689187	0.711432246
105	Antigua and Barbuda	2011	88.89186676	0.715489989
105	Antigua and Barbuda	2012	86.59232662	0.719509323
105	Antigua and Barbuda	2013	84.01423294	0.722864978
105	Antigua and Barbuda	2014	81.2211416	0.725585978
105	Antigua and Barbuda	2015	76.98931486	0.728372762

105	Antigua and Barbuda	2016	74.44649452	0.731598944
105	Antigua and Barbuda	2017	76.86099415	0.734963713
105	Antigua and Barbuda	2018	76.32871892	0.738879106
105	Antigua and Barbuda	2019	76.48581733	0.74305296
105	Antigua and Barbuda	2020	73.74529886	0.74634533
105	Antigua and Barbuda	2021	71.43079285	0.749886887
106	Bahamas	1990	130.3526782	0.693509268
106	Bahamas	1991	127.5498969	0.689126784
106	Bahamas	1992	125.0247563	0.687343838
106	Bahamas	1993	124.5850426	0.693816501
106	Bahamas	1994	122.8202765	0.706819892
106	Bahamas	1995	121.570981	0.719381607
106	Bahamas	1996	120.2122874	0.728478844
106	Bahamas	1997	113.7437341	0.734781215
106	Bahamas	1998	110.5247296	0.739281293
106	Bahamas	1999	116.3765041	0.741622848
106	Bahamas	2000	115.8180397	0.742517946
106	Bahamas	2001	111.4516527	0.743592882
106	Bahamas	2002	104.0075988	0.746250322
106	Bahamas	2003	96.38060795	0.749916714
106	Bahamas	2004	93.6296039	0.753759643
106	Bahamas	2005	89.05345543	0.756782661
106	Bahamas	2006	89.13000932	0.759185939
106	Bahamas	2007	82.39518026	0.761990997
106	Bahamas	2008	84.91446464	0.765590503
106	Bahamas	2009	85.52976012	0.769561674
106	Bahamas	2010	86.36233771	0.77411981
106	Bahamas	2011	85.14297212	0.778281891
106	Bahamas	2012	82.30411237	0.781838639
106	Bahamas	2013	79.66524085	0.784623911
106	Bahamas	2014	81.03767416	0.787253492
106	Bahamas	2015	81.10353662	0.789956145
106	Bahamas	2016	82.36644279	0.792738696
106	Bahamas	2017	79.90657629	0.795347181
106	Bahamas	2018	78.9988487	0.798179034
106	Bahamas	2019	78.55991193	0.80103307
106	Bahamas	2020	77.04656019	0.802948018
106	Bahamas	2021	76.30775247	0.805020668
107	Barbados	1990	120.8039102	0.653582517
107	Barbados	1991	113.3414245	0.656654073
107	Barbados	1992	113.2902973	0.662446635
107	Barbados	1993	110.4895128	0.668815012
107	Barbados	1994	109.7759207	0.673409365
107	Barbados	1995	106.3575421	0.676012247
107	Barbados	1996	104.884625	0.677193054
107	Barbados	1997	102.3239562	0.678644826
107	Barbados	1998	91.67763515	0.679827167
107	Barbados	1999	84.90729867	0.680398459
107	Barbados	2000	85.17746117	0.681304199
107	Barbados	2001	80.36890452	0.683329389

107	Barbados	2002	77.80405388	0.687807576
107	Barbados	2003	80.55564184	0.693235962
107	Barbados	2004	76.11793134	0.698029703
107	Barbados	2005	69.72353102	0.701510049
107	Barbados	2006	71.99210311	0.703856186
107	Barbados	2007	68.89037744	0.705871649
107	Barbados	2008	68.90611397	0.708514495
107	Barbados	2009	66.04350377	0.712143362
107	Barbados	2010	63.11972139	0.716208546
107	Barbados	2011	61.53467798	0.720060687
107	Barbados	2012	60.53024282	0.724162458
107	Barbados	2013	62.11302805	0.72782617
107	Barbados	2014	62.48380383	0.730383287
107	Barbados	2015	62.76569627	0.732602993
107	Barbados	2016	63.03513123	0.734912535
107	Barbados	2017	63.46239759	0.737157255
107	Barbados	2018	64.40146913	0.739531437
107	Barbados	2019	67.07923997	0.742239309
107	Barbados	2020	63.51162881	0.744366646
107	Barbados	2021	62.54727572	0.746748764
108	Belize	1990	119.1871883	0.423726992
108	Belize	1991	120.1820447	0.433834392
108	Belize	1992	118.6960997	0.444198507
108	Belize	1993	119.7631069	0.455258577
108	Belize	1994	121.2005429	0.465729308
108	Belize	1995	123.2968616	0.475278511
108	Belize	1996	130.459418	0.482702165
108	Belize	1997	134.0709029	0.488020129
108	Belize	1998	133.9266702	0.491819335
108	Belize	1999	137.1177871	0.495070196
108	Belize	2000	145.0629238	0.49864373
108	Belize	2001	124.817291	0.502399434
108	Belize	2002	116.4016585	0.506871655
108	Belize	2003	113.2681854	0.512679459
108	Belize	2004	108.2822677	0.519697333
108	Belize	2005	104.4972957	0.527231062
108	Belize	2006	97.91830015	0.534889621
108	Belize	2007	93.38983774	0.541900694
108	Belize	2008	88.05935383	0.548093223
108	Belize	2009	88.56941597	0.553465869
108	Belize	2010	86.67244032	0.558546893
108	Belize	2011	84.23706362	0.563440363
108	Belize	2012	78.90401638	0.568456236
108	Belize	2013	76.10577663	0.573236457
108	Belize	2014	75.47117696	0.578091326
108	Belize	2015	75.48284518	0.583279094
108	Belize	2016	71.59108326	0.588275912
108	Belize	2017	71.8384358	0.59333729
108	Belize	2018	72.85500944	0.598512015
108	Belize	2019	71.80060806	0.603351601

108	Belize	2020	65.95989018	0.607060046
108	Belize	2021	66.97374485	0.610229002
109	Cuba	1990	214.2372239	0.558019071
109	Cuba	1991	204.3135404	0.563413638
109	Cuba	1992	204.326651	0.566169006
109	Cuba	1993	205.4654414	0.565441047
109	Cuba	1994	201.183609	0.563186313
109	Cuba	1995	195.6618782	0.560765902
109	Cuba	1996	189.4995049	0.559346329
109	Cuba	1997	177.2123008	0.558924568
109	Cuba	1998	169.2555274	0.55936322
109	Cuba	1999	160.2111189	0.561820953
109	Cuba	2000	146.4354217	0.566676902
109	Cuba	2001	140.7810017	0.572753861
109	Cuba	2002	129.2562232	0.57996829
109	Cuba	2003	129.5384723	0.587647762
109	Cuba	2004	130.2614423	0.594348715
109	Cuba	2005	132.9492057	0.599863799
109	Cuba	2006	122.8178056	0.604970567
109	Cuba	2007	120.3357287	0.607618099
109	Cuba	2008	120.5349467	0.609400923
109	Cuba	2009	117.842206	0.611775641
109	Cuba	2010	117.1667522	0.615261262
109	Cuba	2011	107.1234196	0.62038669
109	Cuba	2012	106.5028547	0.626156025
109	Cuba	2013	105.1853849	0.631839801
109	Cuba	2014	104.9798759	0.637304129
109	Cuba	2015	107.0013848	0.642818963
109	Cuba	2016	104.1177232	0.647907866
109	Cuba	2017	106.9057241	0.652309286
109	Cuba	2018	105.8538467	0.656858654
109	Cuba	2019	106.8817307	0.661674717
109	Cuba	2020	105.7451223	0.665210747
109	Cuba	2021	104.7572906	0.668729864
110	Dominica	1990	151.5034683	0.56360259
110	Dominica	1991	150.5948834	0.565237843
110	Dominica	1992	147.3537438	0.569570684
110	Dominica	1993	144.6441501	0.578811811
110	Dominica	1994	141.5522699	0.590119147
110	Dominica	1995	137.7975949	0.601986932
110	Dominica	1996	135.2574093	0.61438205
110	Dominica	1997	129.4540053	0.625057353
110	Dominica	1998	124.967554	0.633017994
110	Dominica	1999	120.4501746	0.639453915
110	Dominica	2000	115.6004033	0.645830937
110	Dominica	2001	110.317122	0.652368567
110	Dominica	2002	107.3395312	0.658423554
110	Dominica	2003	105.7374039	0.663678868
110	Dominica	2004	102.4837632	0.668952132
110	Dominica	2005	98.88884303	0.674549077

110	Dominica	2006	97.48756644	0.680342482
110	Dominica	2007	94.93731714	0.685415917
110	Dominica	2008	93.58222067	0.690197519
110	Dominica	2009	91.47122381	0.694461869
110	Dominica	2010	92.11794087	0.698489193
110	Dominica	2011	91.43172332	0.70246326
110	Dominica	2012	91.83093175	0.706870608
110	Dominica	2013	91.48292744	0.712210475
110	Dominica	2014	91.04465961	0.718455556
110	Dominica	2015	91.551507	0.724785536
110	Dominica	2016	91.65724532	0.731760801
110	Dominica	2017	91.87659567	0.736062473
110	Dominica	2018	92.26059673	0.738935954
110	Dominica	2019	92.24698329	0.742132066
110	Dominica	2020	92.30418388	0.744419944
110	Dominica	2021	92.21187243	0.746967185
111	Dominican Republic	1990	150.3572996	0.442654076
111	Dominican Republic	1991	141.0047866	0.446144578
111	Dominican Republic	1992	133.0678044	0.449953081
111	Dominican Republic	1993	128.591672	0.454229297
111	Dominican Republic	1994	122.5544871	0.458620614
111	Dominican Republic	1995	121.0596233	0.463313869
111	Dominican Republic	1996	124.4371462	0.468516938
111	Dominican Republic	1997	127.0714177	0.474504389
111	Dominican Republic	1998	127.9369276	0.481170939
111	Dominican Republic	1999	129.9981472	0.488178541
111	Dominican Republic	2000	128.8403665	0.495468586
111	Dominican Republic	2001	127.9886826	0.502775415
111	Dominican Republic	2002	129.9521638	0.510298019
111	Dominican Republic	2003	139.0255839	0.517395574
111	Dominican Republic	2004	149.1869377	0.524543275
111	Dominican Republic	2005	152.2289871	0.532228649
111	Dominican Republic	2006	154.8743381	0.54022269
111	Dominican Republic	2007	154.1684454	0.548187021
111	Dominican Republic	2008	152.3722878	0.555371313
111	Dominican Republic	2009	149.8753189	0.561783754
111	Dominican Republic	2010	146.7761796	0.568130582
111	Dominican Republic	2011	143.2065419	0.573834813
111	Dominican Republic	2012	147.0159961	0.578966381
111	Dominican Republic	2013	152.3191614	0.583625825
111	Dominican Republic	2014	148.1690875	0.588236743
111	Dominican Republic	2015	148.8821236	0.592924164
111	Dominican Republic	2016	148.9638912	0.597619609
111	Dominican Republic	2017	147.7454432	0.602140291
111	Dominican Republic	2018	147.4306408	0.606960839
111	Dominican Republic	2019	144.0027859	0.611913021
111	Dominican Republic	2020	151.6589098	0.615635625
111	Dominican Republic	2021	144.6552252	0.619388201
112	Grenada	1990	143.9665308	0.436734419
112	Grenada	1991	149.6984868	0.445887953

112	Grenada	1992	166.9387224	0.455910058
112	Grenada	1993	174.0480276	0.46599489
112	Grenada	1994	162.8735368	0.476410341
112	Grenada	1995	150.7933408	0.486558999
112	Grenada	1996	139.321479	0.496589808
112	Grenada	1997	139.3379916	0.506217602
112	Grenada	1998	135.326378	0.516590019
112	Grenada	1999	120.0891673	0.527333411
112	Grenada	2000	115.0476914	0.538388116
112	Grenada	2001	113.7762836	0.54905897
112	Grenada	2002	118.1526516	0.559473128
112	Grenada	2003	125.6083325	0.569718037
112	Grenada	2004	132.5129952	0.578487456
112	Grenada	2005	131.9215956	0.587158877
112	Grenada	2006	124.9605702	0.59405452
112	Grenada	2007	116.1571226	0.600766336
112	Grenada	2008	122.293679	0.606879282
112	Grenada	2009	119.2908646	0.611805257
112	Grenada	2010	114.5319752	0.616486997
112	Grenada	2011	101.5387035	0.620978521
112	Grenada	2012	94.52550526	0.624977512
112	Grenada	2013	95.75732864	0.629048336
112	Grenada	2014	94.8174249	0.633734696
112	Grenada	2015	89.41462261	0.638936084
112	Grenada	2016	96.3065219	0.644335234
112	Grenada	2017	93.4190384	0.649831522
112	Grenada	2018	96.32404018	0.655372772
112	Grenada	2019	98.21713556	0.660808512
112	Grenada	2020	97.71437667	0.665086347
112	Grenada	2021	95.35999173	0.668993028
113	Guyana	1990	230.5925731	0.460430129
113	Guyana	1991	210.4802056	0.462677069
113	Guyana	1992	207.189942	0.466753989
113	Guyana	1993	222.3350971	0.472174229
113	Guyana	1994	223.9195359	0.478992924
113	Guyana	1995	226.3581152	0.486573457
113	Guyana	1996	227.6964184	0.495216036
113	Guyana	1997	218.3391002	0.504362779
113	Guyana	1998	199.521925	0.513068898
113	Guyana	1999	176.0586544	0.521595978
113	Guyana	2000	164.3729892	0.528693992
113	Guyana	2001	184.9046587	0.534869806
113	Guyana	2002	190.900914	0.540514273
113	Guyana	2003	203.8447064	0.545516473
113	Guyana	2004	211.6682793	0.55032637
113	Guyana	2005	210.7977299	0.554844797
113	Guyana	2006	206.6629413	0.559465611
113	Guyana	2007	203.4182606	0.564541135
113	Guyana	2008	194.4349671	0.569595206
113	Guyana	2009	184.4781186	0.574769931

113	Guyana	2010	179.9502229	0.580083911
113	Guyana	2011	175.7840221	0.585688269
113	Guyana	2012	175.7743387	0.591336582
113	Guyana	2013	168.1406108	0.596826295
113	Guyana	2014	162.9405393	0.602136511
113	Guyana	2015	161.1664984	0.607605487
113	Guyana	2016	159.0341373	0.61391436
113	Guyana	2017	157.7991019	0.620602607
113	Guyana	2018	157.0004153	0.627167041
113	Guyana	2019	153.605502	0.633640347
113	Guyana	2020	149.069314	0.642284645
113	Guyana	2021	143.2302519	0.650812335
114	Haiti	1990	264.0044757	0.31033463
114	Haiti	1991	256.7891145	0.315455441
114	Haiti	1992	252.3285678	0.320389979
114	Haiti	1993	249.3249076	0.324895851
114	Haiti	1994	244.7656782	0.328399456
114	Haiti	1995	241.2010405	0.332999152
114	Haiti	1996	235.7546802	0.338229396
114	Haiti	1997	233.3257305	0.343880049
114	Haiti	1998	228.8128872	0.349698073
114	Haiti	1999	229.9798253	0.355980743
114	Haiti	2000	228.6022941	0.362572209
114	Haiti	2001	227.4745006	0.368643508
114	Haiti	2002	225.3370754	0.374473325
114	Haiti	2003	225.3826654	0.380067255
114	Haiti	2004	225.7210144	0.385061512
114	Haiti	2005	226.0842008	0.38984542
114	Haiti	2006	224.2527766	0.394322001
114	Haiti	2007	222.0364046	0.398973441
114	Haiti	2008	220.7540803	0.403285871
114	Haiti	2009	219.4183722	0.407944424
114	Haiti	2010	218.6021378	0.411840105
114	Haiti	2011	219.5530281	0.41617719
114	Haiti	2012	218.705768	0.420021528
114	Haiti	2013	217.8020356	0.423985549
114	Haiti	2014	217.9174834	0.427940902
114	Haiti	2015	218.8402937	0.43175676
114	Haiti	2016	217.5298982	0.435288722
114	Haiti	2017	215.5491927	0.438541003
114	Haiti	2018	214.7933236	0.441678925
114	Haiti	2019	214.1182781	0.444328854
114	Haiti	2020	212.545006	0.446390614
114	Haiti	2021	210.1112777	0.448278285
115	Jamaica	1990	71.38080383	0.534781234
115	Jamaica	1991	60.68871478	0.53959448
115	Jamaica	1992	63.85295834	0.545088753
115	Jamaica	1993	67.38205186	0.551218492
115	Jamaica	1994	67.40335363	0.557643773
115	Jamaica	1995	62.62787826	0.564315038

115	Jamaica	1996	51.95289067	0.570940131
115	Jamaica	1997	49.91501946	0.577212091
115	Jamaica	1998	49.55327888	0.583048035
115	Jamaica	1999	52.40868304	0.588846467
115	Jamaica	2000	51.41218455	0.594592917
115	Jamaica	2001	51.10806573	0.600192022
115	Jamaica	2002	48.80425382	0.605591748
115	Jamaica	2003	47.3797061	0.610877424
115	Jamaica	2004	45.166939	0.615836278
115	Jamaica	2005	42.70755693	0.620400057
115	Jamaica	2006	41.87463651	0.62501886
115	Jamaica	2007	42.9660473	0.62954947
115	Jamaica	2008	38.37194337	0.633967851
115	Jamaica	2009	56.26842579	0.638110835
115	Jamaica	2010	45.23393226	0.642129388
115	Jamaica	2011	52.56313519	0.64639583
115	Jamaica	2012	45.40041305	0.65058376
115	Jamaica	2013	47.65128511	0.654573624
115	Jamaica	2014	54.1916901	0.658505341
115	Jamaica	2015	54.41250278	0.662465809
115	Jamaica	2016	53.62597942	0.666469279
115	Jamaica	2017	53.13882888	0.670291045
115	Jamaica	2018	52.98601981	0.674075313
115	Jamaica	2019	53.52245188	0.677778539
115	Jamaica	2020	51.49781426	0.680567177
115	Jamaica	2021	52.44656625	0.683263064
116	Saint Lucia	1990	150.2257245	0.49629657
116	Saint Lucia	1991	140.4101703	0.505975555
116	Saint Lucia	1992	136.3262998	0.515368261
116	Saint Lucia	1993	137.650128	0.524234949
116	Saint Lucia	1994	130.6369345	0.532731083
116	Saint Lucia	1995	127.5926691	0.541479794
116	Saint Lucia	1996	114.4227736	0.550242412
116	Saint Lucia	1997	104.8802632	0.558293461
116	Saint Lucia	1998	97.76948555	0.566138703
116	Saint Lucia	1999	92.1609315	0.573428555
116	Saint Lucia	2000	80.7216463	0.579788896
116	Saint Lucia	2001	77.41029785	0.585087558
116	Saint Lucia	2002	76.2923139	0.590167578
116	Saint Lucia	2003	73.53931581	0.595675146
116	Saint Lucia	2004	71.52574756	0.601625482
116	Saint Lucia	2005	74.09775987	0.607239866
116	Saint Lucia	2006	65.24456472	0.6127553
116	Saint Lucia	2007	59.24097461	0.617994714
116	Saint Lucia	2008	56.3205265	0.623273001
116	Saint Lucia	2009	52.17440149	0.628169849
116	Saint Lucia	2010	50.74540564	0.632782905
116	Saint Lucia	2011	47.19480265	0.637439442
116	Saint Lucia	2012	47.03411578	0.641567127
116	Saint Lucia	2013	49.21795371	0.645153079

116	Saint Lucia	2014	45.63827081	0.648743507
116	Saint Lucia	2015	42.54959985	0.65215748
116	Saint Lucia	2016	44.3115469	0.65565334
116	Saint Lucia	2017	45.04342255	0.659341346
116	Saint Lucia	2018	45.46665808	0.663086682
116	Saint Lucia	2019	45.6262082	0.666840359
116	Saint Lucia	2020	47.43874937	0.66972049
116	Saint Lucia	2021	48.50328781	0.672509735
117	Saint Vincent and the Grenadines	1990	176.8552347	0.475930186
117	Saint Vincent and the Grenadines	1991	177.1441033	0.481075658
117	Saint Vincent and the Grenadines	1992	181.7578681	0.485977871
117	Saint Vincent and the Grenadines	1993	187.5899971	0.490998791
117	Saint Vincent and the Grenadines	1994	185.4442615	0.496379132
117	Saint Vincent and the Grenadines	1995	176.3602441	0.50298609
117	Saint Vincent and the Grenadines	1996	167.8952606	0.509527158
117	Saint Vincent and the Grenadines	1997	169.6909044	0.515482619
117	Saint Vincent and the Grenadines	1998	159.0431984	0.521209679
117	Saint Vincent and the Grenadines	1999	152.9093497	0.527099552
117	Saint Vincent and the Grenadines	2000	146.7720471	0.53317329
117	Saint Vincent and the Grenadines	2001	135.4472804	0.539455712
117	Saint Vincent and the Grenadines	2002	130.6029939	0.546063616
117	Saint Vincent and the Grenadines	2003	125.6870779	0.552693724
117	Saint Vincent and the Grenadines	2004	121.8953277	0.558663543
117	Saint Vincent and the Grenadines	2005	123.5899403	0.563588116
117	Saint Vincent and the Grenadines	2006	126.9580352	0.568221748
117	Saint Vincent and the Grenadines	2007	128.1901903	0.572819052
117	Saint Vincent and the Grenadines	2008	136.5384788	0.577611145
117	Saint Vincent and the Grenadines	2009	130.3584925	0.582549107
117	Saint Vincent and the Grenadines	2010	129.2528433	0.587190384
117	Saint Vincent and the Grenadines	2011	127.5587583	0.590259096
117	Saint Vincent and the Grenadines	2012	128.9914188	0.592153908
117	Saint Vincent and the Grenadines	2013	124.1198891	0.594614075
117	Saint Vincent and the Grenadines	2014	117.8453644	0.598378672
117	Saint Vincent and the Grenadines	2015	108.1491266	0.603754135
117	Saint Vincent and the Grenadines	2016	101.9537093	0.61005921
117	Saint Vincent and the Grenadines	2017	95.55433555	0.616438473
117	Saint Vincent and the Grenadines	2018	99.81258787	0.622634944
117	Saint Vincent and the Grenadines	2019	105.1495433	0.628325986
117	Saint Vincent and the Grenadines	2020	103.5899346	0.632860904
117	Saint Vincent and the Grenadines	2021	100.9712536	0.637195963
118	Suriname	1990	172.9647317	0.502054305
118	Suriname	1991	172.187071	0.506655795
118	Suriname	1992	173.6227353	0.510959772
118	Suriname	1993	183.4181454	0.514419437
118	Suriname	1994	166.9373006	0.517650245
118	Suriname	1995	138.9049103	0.521002622
118	Suriname	1996	128.9604556	0.525059318
118	Suriname	1997	127.8084992	0.529750812
118	Suriname	1998	129.1323415	0.534599696
118	Suriname	1999	130.3854024	0.538932791

118	Suriname	2000	128.8129275	0.543660023
118	Suriname	2001	127.4452386	0.548683524
118	Suriname	2002	128.7340439	0.553879432
118	Suriname	2003	127.0747475	0.559418373
118	Suriname	2004	123.3714445	0.565109708
118	Suriname	2005	119.4532489	0.570462186
118	Suriname	2006	113.7913805	0.575647048
118	Suriname	2007	109.440909	0.580654574
118	Suriname	2008	107.0033828	0.585644825
118	Suriname	2009	103.9960949	0.590512805
118	Suriname	2010	102.2054292	0.595469745
118	Suriname	2011	98.85768486	0.600356336
118	Suriname	2012	98.36657416	0.60474187
118	Suriname	2013	101.8729629	0.608910612
118	Suriname	2014	102.5835652	0.612704423
118	Suriname	2015	104.2103598	0.616097315
118	Suriname	2016	101.0024626	0.618868088
118	Suriname	2017	100.2230835	0.62168418
118	Suriname	2018	102.2833665	0.624708011
118	Suriname	2019	103.2319083	0.627896781
118	Suriname	2020	100.6010448	0.630683705
118	Suriname	2021	90.94083242	0.633665739
119	Trinidad and Tobago	1990	234.7150033	0.62397015
119	Trinidad and Tobago	1991	228.8709268	0.628758918
119	Trinidad and Tobago	1992	227.1390721	0.634039552
119	Trinidad and Tobago	1993	230.3975054	0.639191914
119	Trinidad and Tobago	1994	231.7108073	0.64424291
119	Trinidad and Tobago	1995	212.3605395	0.649220055
119	Trinidad and Tobago	1996	218.5671939	0.654169455
119	Trinidad and Tobago	1997	204.9281625	0.659293633
119	Trinidad and Tobago	1998	211.6512159	0.664507714
119	Trinidad and Tobago	1999	207.3282066	0.670244574
119	Trinidad and Tobago	2000	182.3634696	0.676683835
119	Trinidad and Tobago	2001	176.0290654	0.682763494
119	Trinidad and Tobago	2002	168.1710934	0.688872271
119	Trinidad and Tobago	2003	170.3549272	0.695828341
119	Trinidad and Tobago	2004	159.2958267	0.702610809
119	Trinidad and Tobago	2005	152.4245235	0.708520985
119	Trinidad and Tobago	2006	144.1505465	0.71460021
119	Trinidad and Tobago	2007	136.0191651	0.720196417
119	Trinidad and Tobago	2008	139.835408	0.725720702
119	Trinidad and Tobago	2009	128.0035169	0.729538892
119	Trinidad and Tobago	2010	122.3058886	0.733237077
119	Trinidad and Tobago	2011	113.2503317	0.736802405
119	Trinidad and Tobago	2012	104.4792382	0.739999073
119	Trinidad and Tobago	2013	107.9107825	0.743593955
119	Trinidad and Tobago	2014	107.4706828	0.747338963
119	Trinidad and Tobago	2015	107.4705047	0.751510682
119	Trinidad and Tobago	2016	112.4268047	0.754953348
119	Trinidad and Tobago	2017	109.4570307	0.758226988

119	Trinidad and Tobago	2018	106.5262679	0.761241991
119	Trinidad and Tobago	2019	105.4550244	0.764169599
119	Trinidad and Tobago	2020	107.1506669	0.766422081
119	Trinidad and Tobago	2021	105.9553405	0.768763254
121	Bolivia (Plurinational State of)	1990	142.7445527	0.423917961
121	Bolivia (Plurinational State of)	1991	140.1941243	0.429397795
121	Bolivia (Plurinational State of)	1992	137.1077781	0.434835542
121	Bolivia (Plurinational State of)	1993	134.2090506	0.440601646
121	Bolivia (Plurinational State of)	1994	129.9673905	0.4468225
121	Bolivia (Plurinational State of)	1995	126.8993806	0.453479243
121	Bolivia (Plurinational State of)	1996	122.1368746	0.460220669
121	Bolivia (Plurinational State of)	1997	119.3335209	0.466982897
121	Bolivia (Plurinational State of)	1998	114.8619104	0.473796201
121	Bolivia (Plurinational State of)	1999	110.9542405	0.48019431
121	Bolivia (Plurinational State of)	2000	106.7696382	0.486327903
121	Bolivia (Plurinational State of)	2001	102.496918	0.492030764
121	Bolivia (Plurinational State of)	2002	99.74628899	0.497348837
121	Bolivia (Plurinational State of)	2003	97.02991466	0.502365803
121	Bolivia (Plurinational State of)	2004	94.62683885	0.507273036
121	Bolivia (Plurinational State of)	2005	93.57575584	0.512166491
121	Bolivia (Plurinational State of)	2006	92.69415528	0.517146194
121	Bolivia (Plurinational State of)	2007	92.70709836	0.52194613
121	Bolivia (Plurinational State of)	2008	92.63626852	0.52694375
121	Bolivia (Plurinational State of)	2009	92.49871892	0.53179244
121	Bolivia (Plurinational State of)	2010	92.32275491	0.537009068
121	Bolivia (Plurinational State of)	2011	91.39448381	0.542680745
121	Bolivia (Plurinational State of)	2012	91.23097744	0.548615482
121	Bolivia (Plurinational State of)	2013	90.63043659	0.554788216
121	Bolivia (Plurinational State of)	2014	91.14319672	0.560934581
121	Bolivia (Plurinational State of)	2015	90.94137082	0.567000898
121	Bolivia (Plurinational State of)	2016	89.83560706	0.573080316
121	Bolivia (Plurinational State of)	2017	89.65260312	0.579241285
121	Bolivia (Plurinational State of)	2018	88.84968373	0.585184106
121	Bolivia (Plurinational State of)	2019	87.97872385	0.590692043
121	Bolivia (Plurinational State of)	2020	86.52181129	0.594854443
121	Bolivia (Plurinational State of)	2021	84.62657478	0.599010799
122	Ecuador	1990	99.85633061	0.518430614
122	Ecuador	1991	105.4759585	0.517679421
122	Ecuador	1992	101.9420953	0.51837907
122	Ecuador	1993	97.59784363	0.520707291
122	Ecuador	1994	93.34453062	0.524908291
122	Ecuador	1995	77.83081089	0.529380752
122	Ecuador	1996	71.39095209	0.532691399
122	Ecuador	1997	72.56559045	0.535349652
122	Ecuador	1998	76.55265671	0.53719633
122	Ecuador	1999	77.75701901	0.539032308
122	Ecuador	2000	75.09016302	0.543044779
122	Ecuador	2001	76.08530529	0.54855215
122	Ecuador	2002	78.89603986	0.554897325
122	Ecuador	2003	75.16084284	0.560955485

122	Ecuador	2004	73.66222395	0.566290293
122	Ecuador	2005	77.46094785	0.569927345
122	Ecuador	2006	76.39477103	0.572516273
122	Ecuador	2007	73.95220694	0.574704702
122	Ecuador	2008	70.44875333	0.57812504
122	Ecuador	2009	61.57767551	0.582484258
122	Ecuador	2010	57.18441437	0.588763056
122	Ecuador	2011	54.5285894	0.596729076
122	Ecuador	2012	55.68980388	0.605454319
122	Ecuador	2013	57.93086759	0.614080275
122	Ecuador	2014	63.4120616	0.622027433
122	Ecuador	2015	68.05331177	0.62854671
122	Ecuador	2016	76.50435721	0.634281143
122	Ecuador	2017	81.60983991	0.640011219
122	Ecuador	2018	84.92399849	0.64588852
122	Ecuador	2019	84.20220353	0.651787987
122	Ecuador	2020	77.38768222	0.656714457
122	Ecuador	2021	77.47383905	0.661017053
123	Peru	1990	79.04668668	0.510419852
123	Peru	1991	70.82188682	0.512161525
123	Peru	1992	75.23261035	0.513821552
123	Peru	1993	75.25066748	0.515981506
123	Peru	1994	74.85947729	0.51930608
123	Peru	1995	75.28881556	0.523640527
123	Peru	1996	70.15993116	0.528312042
123	Peru	1997	69.18312856	0.533576491
123	Peru	1998	67.72287848	0.538721717
123	Peru	1999	58.45303535	0.543853377
123	Peru	2000	56.13015671	0.548952201
123	Peru	2001	52.01872411	0.553775453
123	Peru	2002	51.48038157	0.558853263
123	Peru	2003	51.05093825	0.563871072
123	Peru	2004	48.39589675	0.569063024
123	Peru	2005	45.68804699	0.574517001
123	Peru	2006	42.99885891	0.580503365
123	Peru	2007	40.44740924	0.586958489
123	Peru	2008	39.38905786	0.593750811
123	Peru	2009	42.2560586	0.599954993
123	Peru	2010	42.21796016	0.606520932
123	Peru	2011	41.08259955	0.613051221
123	Peru	2012	40.53821152	0.619355264
123	Peru	2013	39.45602638	0.625519024
123	Peru	2014	38.0333052	0.631114354
123	Peru	2015	37.39108002	0.636278271
123	Peru	2016	38.76817791	0.641285279
123	Peru	2017	42.67514205	0.646180855
123	Peru	2018	45.15478101	0.650977317
123	Peru	2019	49.41104611	0.655433735
123	Peru	2020	46.43990579	0.658672244
123	Peru	2021	44.80022266	0.662054037

125	Colombia	1990	143.5227285	0.480720054
125	Colombia	1991	146.5596181	0.482339894
125	Colombia	1992	143.3994575	0.484572359
125	Colombia	1993	139.3430457	0.488229373
125	Colombia	1994	134.9331131	0.493278182
125	Colombia	1995	129.6425442	0.499102757
125	Colombia	1996	128.0857777	0.504986969
125	Colombia	1997	116.0314813	0.511079502
125	Colombia	1998	108.1253752	0.517022505
125	Colombia	1999	107.0489057	0.52243594
125	Colombia	2000	105.5956843	0.527839094
125	Colombia	2001	103.5572106	0.533076501
125	Colombia	2002	99.97166373	0.538189342
125	Colombia	2003	99.11054157	0.543265131
125	Colombia	2004	99.27113748	0.548601832
125	Colombia	2005	96.26061406	0.554089191
125	Colombia	2006	95.952907	0.560182735
125	Colombia	2007	91.35762281	0.566822644
125	Colombia	2008	89.99085101	0.573494652
125	Colombia	2009	90.29056198	0.579876469
125	Colombia	2010	88.89262343	0.586408108
125	Colombia	2011	84.65736329	0.593340477
125	Colombia	2012	83.64032584	0.600254238
125	Colombia	2013	82.07340946	0.607250637
125	Colombia	2014	80.13996006	0.614151923
125	Colombia	2015	80.51478357	0.620889228
125	Colombia	2016	80.76262878	0.627554497
125	Colombia	2017	78.62802005	0.634130359
125	Colombia	2018	80.08757757	0.640255072
125	Colombia	2019	76.87565343	0.646038852
125	Colombia	2020	78.87293645	0.650855354
125	Colombia	2021	79.05254853	0.655442913
126	Costa Rica	1990	124.7538392	0.534125181
126	Costa Rica	1991	123.3034634	0.539342164
126	Costa Rica	1992	121.4821356	0.544624238
126	Costa Rica	1993	119.3584558	0.549946626
126	Costa Rica	1994	122.1523092	0.55517125
126	Costa Rica	1995	121.922752	0.560547086
126	Costa Rica	1996	114.0394322	0.565180862
126	Costa Rica	1997	105.1971344	0.570128417
126	Costa Rica	1998	100.3626257	0.576067051
126	Costa Rica	1999	103.6235979	0.582229492
126	Costa Rica	2000	100.1305719	0.588243191
126	Costa Rica	2001	100.0747873	0.594889115
126	Costa Rica	2002	94.40720516	0.600894433
126	Costa Rica	2003	90.15890867	0.605765687
126	Costa Rica	2004	89.10288562	0.610315699
126	Costa Rica	2005	81.76614354	0.614412619
126	Costa Rica	2006	85.05970212	0.617850162
126	Costa Rica	2007	73.44597426	0.621200571

126	Costa Rica	2008	73.36554382	0.62570391
126	Costa Rica	2009	70.59234096	0.631291379
126	Costa Rica	2010	77.80855556	0.636595426
126	Costa Rica	2011	73.09877695	0.641201295
126	Costa Rica	2012	72.70190842	0.646574093
126	Costa Rica	2013	71.3178481	0.651945813
126	Costa Rica	2014	70.60117887	0.656890456
126	Costa Rica	2015	68.32095987	0.662030136
126	Costa Rica	2016	68.4307821	0.667430092
126	Costa Rica	2017	65.86998039	0.673621392
126	Costa Rica	2018	65.13564677	0.681296147
126	Costa Rica	2019	57.53040171	0.690049088
126	Costa Rica	2020	54.09858218	0.696194605
126	Costa Rica	2021	55.09711988	0.700340477
127	El Salvador	1990	118.019363	0.373057975
127	El Salvador	1991	114.4297361	0.374855325
127	El Salvador	1992	117.1099664	0.377076643
127	El Salvador	1993	120.9107597	0.380307326
127	El Salvador	1994	121.8839258	0.385149094
127	El Salvador	1995	114.7766406	0.391780266
127	El Salvador	1996	111.478548	0.400178468
127	El Salvador	1997	110.3478123	0.410455826
127	El Salvador	1998	113.3322793	0.42142119
127	El Salvador	1999	105.0719993	0.432733148
127	El Salvador	2000	103.0125422	0.443962666
127	El Salvador	2001	100.4402573	0.454177659
127	El Salvador	2002	94.61436568	0.46296886
127	El Salvador	2003	96.37962417	0.470397413
127	El Salvador	2004	98.90673461	0.476536664
127	El Salvador	2005	98.88533399	0.482041719
127	El Salvador	2006	99.07642433	0.486826
127	El Salvador	2007	96.59337976	0.490809752
127	El Salvador	2008	93.05557763	0.495077739
127	El Salvador	2009	97.58756	0.499489714
127	El Salvador	2010	91.14860118	0.504171335
127	El Salvador	2011	92.76491611	0.508969239
127	El Salvador	2012	87.33584415	0.513396874
127	El Salvador	2013	91.51731254	0.518121908
127	El Salvador	2014	96.30960491	0.523468901
127	El Salvador	2015	104.4100709	0.52938695
127	El Salvador	2016	97.88335311	0.535650269
127	El Salvador	2017	97.3245364	0.541998957
127	El Salvador	2018	93.70947207	0.548143174
127	El Salvador	2019	95.78536476	0.554031648
127	El Salvador	2020	96.90682432	0.558919894
127	El Salvador	2021	94.73525542	0.563775188
128	Guatemala	1990	149.1527444	0.311792455
128	Guatemala	1991	159.3673631	0.314329484
128	Guatemala	1992	165.7235368	0.319165481
128	Guatemala	1993	167.1802453	0.326840819

128	Guatemala	1994	166.8974738	0.3334691
128	Guatemala	1995	147.9387282	0.339642101
128	Guatemala	1996	134.9264274	0.346961212
128	Guatemala	1997	139.9579194	0.355995963
128	Guatemala	1998	145.9290688	0.366461193
128	Guatemala	1999	131.0585688	0.374455316
128	Guatemala	2000	122.2014905	0.379877297
128	Guatemala	2001	111.4812803	0.386927686
128	Guatemala	2002	101.8398075	0.396899639
128	Guatemala	2003	101.9199649	0.408234164
128	Guatemala	2004	99.83674601	0.419777065
128	Guatemala	2005	104.72712	0.430327529
128	Guatemala	2006	106.9348837	0.438856376
128	Guatemala	2007	105.3360835	0.447245965
128	Guatemala	2008	102.6232186	0.456541865
128	Guatemala	2009	104.6624073	0.464003077
128	Guatemala	2010	109.5188438	0.470477364
128	Guatemala	2011	115.3522762	0.476909684
128	Guatemala	2012	113.1015528	0.481862853
128	Guatemala	2013	113.5422096	0.487286692
128	Guatemala	2014	113.4256069	0.494027188
128	Guatemala	2015	114.300775	0.500655607
128	Guatemala	2016	114.1276371	0.507149792
128	Guatemala	2017	110.9689053	0.513735682
128	Guatemala	2018	112.4013073	0.520995469
128	Guatemala	2019	115.4241931	0.52863047
128	Guatemala	2020	98.14181468	0.534571414
128	Guatemala	2021	97.25891722	0.539972424
129	Honduras	1990	112.0467208	0.332042889
129	Honduras	1991	109.4731241	0.337034649
129	Honduras	1992	115.5446556	0.342305657
129	Honduras	1993	114.1269841	0.347838446
129	Honduras	1994	113.8151101	0.353164852
129	Honduras	1995	127.5071714	0.358787268
129	Honduras	1996	142.1087861	0.364328424
129	Honduras	1997	142.4976674	0.369983894
129	Honduras	1998	143.316441	0.375950111
129	Honduras	1999	144.8326626	0.381752316
129	Honduras	2000	145.9647525	0.387988053
129	Honduras	2001	147.0494829	0.394174012
129	Honduras	2002	147.436807	0.400371798
129	Honduras	2003	147.8688856	0.406668381
129	Honduras	2004	147.9151064	0.413061816
129	Honduras	2005	147.8368702	0.419612332
129	Honduras	2006	147.2430953	0.426352997
129	Honduras	2007	146.4323484	0.433240315
129	Honduras	2008	148.2570879	0.439998164
129	Honduras	2009	152.6749228	0.446151054
129	Honduras	2010	159.6285702	0.452382226
129	Honduras	2011	173.7209006	0.458700329

129	Honduras	2012	180.6367255	0.464925155
129	Honduras	2013	180.9841633	0.470766833
129	Honduras	2014	178.9778674	0.476587799
129	Honduras	2015	178.9647419	0.482294176
129	Honduras	2016	177.081981	0.487705932
129	Honduras	2017	175.426125	0.493130694
129	Honduras	2018	173.7059298	0.498657227
129	Honduras	2019	171.8767776	0.504156505
129	Honduras	2020	170.2906408	0.508669246
129	Honduras	2021	167.9342562	0.513037248
130	Mexico	1990	113.3946232	0.504996083
130	Mexico	1991	110.6678219	0.50951067
130	Mexico	1992	109.3071866	0.514638057
130	Mexico	1993	108.7770109	0.520493167
130	Mexico	1994	108.9909446	0.526749241
130	Mexico	1995	112.35678	0.531554141
130	Mexico	1996	114.2014316	0.536530827
130	Mexico	1997	113.4841542	0.541988171
130	Mexico	1998	109.2889787	0.547842121
130	Mexico	1999	104.7474804	0.55381337
130	Mexico	2000	100.1087515	0.559532547
130	Mexico	2001	98.30272762	0.564774176
130	Mexico	2002	99.43351795	0.570113531
130	Mexico	2003	99.4855506	0.575698215
130	Mexico	2004	95.75629827	0.580984982
130	Mexico	2005	95.80039316	0.58522408
130	Mexico	2006	92.66499551	0.588543226
130	Mexico	2007	90.70881363	0.591447408
130	Mexico	2008	94.28806411	0.594454558
130	Mexico	2009	97.43459574	0.596684358
130	Mexico	2010	99.27731948	0.599527028
130	Mexico	2011	97.37069111	0.603646095
130	Mexico	2012	97.12642824	0.608914289
130	Mexico	2013	101.7747859	0.615089993
130	Mexico	2014	102.9486396	0.621857405
130	Mexico	2015	104.3740999	0.628896724
130	Mexico	2016	108.9851786	0.635925349
130	Mexico	2017	109.589154	0.642667198
130	Mexico	2018	111.7780007	0.649053317
130	Mexico	2019	113.1059013	0.655095338
130	Mexico	2020	110.4322529	0.660119062
130	Mexico	2021	113.2126981	0.664575304
131	Nicaragua	1990	89.15531943	0.346035235
131	Nicaragua	1991	95.5031079	0.351740622
131	Nicaragua	1992	90.45846383	0.357712704
131	Nicaragua	1993	91.51130942	0.363562069
131	Nicaragua	1994	88.24877951	0.369727968
131	Nicaragua	1995	90.38442246	0.376625345
131	Nicaragua	1996	92.7805878	0.384087266
131	Nicaragua	1997	88.01700017	0.391775893

131	Nicaragua	1998	86.1905625	0.399807535
131	Nicaragua	1999	87.94156562	0.408085965
131	Nicaragua	2000	86.64519255	0.416108363
131	Nicaragua	2001	90.19407099	0.423790295
131	Nicaragua	2002	94.29707177	0.430813755
131	Nicaragua	2003	98.61332972	0.437248152
131	Nicaragua	2004	100.0020957	0.443274511
131	Nicaragua	2005	103.4137976	0.448844739
131	Nicaragua	2006	95.09490599	0.453950499
131	Nicaragua	2007	98.89462894	0.459042798
131	Nicaragua	2008	100.8608866	0.463837699
131	Nicaragua	2009	99.89590254	0.467961839
131	Nicaragua	2010	96.08425038	0.472172
131	Nicaragua	2011	99.25364116	0.476593466
131	Nicaragua	2012	99.12684035	0.4812969
131	Nicaragua	2013	98.99708142	0.48610547
131	Nicaragua	2014	96.73629312	0.491207346
131	Nicaragua	2015	92.39304074	0.496391663
131	Nicaragua	2016	96.181366	0.501853345
131	Nicaragua	2017	90.8353782	0.507482022
131	Nicaragua	2018	91.44135024	0.512338228
131	Nicaragua	2019	90.71332048	0.516597046
131	Nicaragua	2020	83.80579677	0.52029267
131	Nicaragua	2021	81.9272041	0.523958472
132	Panama	1990	105.3325891	0.546048123
132	Panama	1991	101.4989172	0.550484218
132	Panama	1992	102.4761386	0.554972755
132	Panama	1993	100.9021736	0.559168666
132	Panama	1994	96.15280506	0.561714534
132	Panama	1995	95.90650567	0.563353023
132	Panama	1996	91.94875862	0.56531007
132	Panama	1997	91.47151082	0.569395939
132	Panama	1998	80.64865168	0.574697318
132	Panama	1999	74.12311002	0.580432842
132	Panama	2000	70.50200998	0.587378393
132	Panama	2001	68.91729821	0.594535701
132	Panama	2002	68.72823942	0.600138677
132	Panama	2003	70.22636794	0.604043365
132	Panama	2004	69.96537598	0.60687703
132	Panama	2005	71.42986425	0.609494125
132	Panama	2006	69.9986365	0.612249713
132	Panama	2007	68.50858198	0.615285417
132	Panama	2008	67.84575085	0.618958907
132	Panama	2009	70.54081254	0.622402806
132	Panama	2010	71.01242434	0.625116323
132	Panama	2011	68.67222067	0.628633942
132	Panama	2012	65.8104226	0.634263435
132	Panama	2013	65.32737429	0.641324353
132	Panama	2014	62.51331099	0.649108174
132	Panama	2015	58.88236898	0.657962047

132	Panama	2016	58.45609167	0.667276758
132	Panama	2017	59.13450732	0.676783503
132	Panama	2018	58.69307475	0.686719925
132	Panama	2019	56.93677226	0.69667068
132	Panama	2020	56.18044592	0.70378849
132	Panama	2021	54.47595445	0.708864828
133	Venezuela (Bolivarian Republic of)	1990	169.5558935	0.516891218
133	Venezuela (Bolivarian Republic of)	1991	169.0318972	0.522272296
133	Venezuela (Bolivarian Republic of)	1992	167.1651921	0.529517991
133	Venezuela (Bolivarian Republic of)	1993	164.5869719	0.536279978
133	Venezuela (Bolivarian Republic of)	1994	167.1181735	0.541866058
133	Venezuela (Bolivarian Republic of)	1995	162.350371	0.546348234
133	Venezuela (Bolivarian Republic of)	1996	153.5131445	0.548649226
133	Venezuela (Bolivarian Republic of)	1997	145.1258778	0.552364437
133	Venezuela (Bolivarian Republic of)	1998	146.6148046	0.557243117
133	Venezuela (Bolivarian Republic of)	1999	145.1601697	0.560277999
133	Venezuela (Bolivarian Republic of)	2000	144.4283431	0.56350626
133	Venezuela (Bolivarian Republic of)	2001	143.445001	0.565458381
133	Venezuela (Bolivarian Republic of)	2002	140.9552839	0.562604461
133	Venezuela (Bolivarian Republic of)	2003	141.3371738	0.555212224
133	Venezuela (Bolivarian Republic of)	2004	131.0103952	0.550972256
133	Venezuela (Bolivarian Republic of)	2005	125.8198573	0.553941679
133	Venezuela (Bolivarian Republic of)	2006	126.8099575	0.56333419
133	Venezuela (Bolivarian Republic of)	2007	125.6181156	0.575367174
133	Venezuela (Bolivarian Republic of)	2008	127.4544217	0.586184887
133	Venezuela (Bolivarian Republic of)	2009	124.5764895	0.593288422
133	Venezuela (Bolivarian Republic of)	2010	118.561648	0.599127923
133	Venezuela (Bolivarian Republic of)	2011	118.4115675	0.604823484
133	Venezuela (Bolivarian Republic of)	2012	117.5673365	0.610187465
133	Venezuela (Bolivarian Republic of)	2013	116.8954723	0.614441024
133	Venezuela (Bolivarian Republic of)	2014	120.7901162	0.614686806
133	Venezuela (Bolivarian Republic of)	2015	121.0006535	0.610675357
133	Venezuela (Bolivarian Republic of)	2016	132.2617038	0.606993213
133	Venezuela (Bolivarian Republic of)	2017	128.0111691	0.605160131
133	Venezuela (Bolivarian Republic of)	2018	128.8426292	0.604068247
133	Venezuela (Bolivarian Republic of)	2019	132.6312047	0.602313887
133	Venezuela (Bolivarian Republic of)	2020	136.6697217	0.600034781
133	Venezuela (Bolivarian Republic of)	2021	141.3953853	0.596513059
135	Brazil	1990	136.6145185	0.500070509
135	Brazil	1991	128.4879568	0.504784798
135	Brazil	1992	124.6883893	0.508539943
135	Brazil	1993	124.4658676	0.512081793
135	Brazil	1994	120.1648864	0.515760842
135	Brazil	1995	116.8020507	0.519685963
135	Brazil	1996	114.3302873	0.523863329
135	Brazil	1997	109.3863015	0.528177889
135	Brazil	1998	106.9235807	0.532481169
135	Brazil	1999	103.443855	0.536972035
135	Brazil	2000	99.48219218	0.542051497
135	Brazil	2001	96.78976852	0.54730443

135	Brazil	2002	94.86578	0.552776035
135	Brazil	2003	93.28836191	0.55814571
135	Brazil	2004	91.82993164	0.563772907
135	Brazil	2005	86.84637036	0.569458118
135	Brazil	2006	85.64955251	0.575338775
135	Brazil	2007	83.87034077	0.581588755
135	Brazil	2008	82.30049231	0.588077818
135	Brazil	2009	80.85375769	0.594073297
135	Brazil	2010	79.88847037	0.600445846
135	Brazil	2011	79.18435075	0.606777291
135	Brazil	2012	76.83294994	0.612619337
135	Brazil	2013	75.29084299	0.618218237
135	Brazil	2014	73.65312489	0.623433814
135	Brazil	2015	72.84036175	0.628149803
135	Brazil	2016	73.14771186	0.632235114
135	Brazil	2017	69.88706023	0.636422188
135	Brazil	2018	67.73962271	0.640802974
135	Brazil	2019	66.7776786	0.645298005
135	Brazil	2020	65.8971536	0.649201568
135	Brazil	2021	64.01345529	0.653043887
136	Paraguay	1990	109.3095727	0.469527785
136	Paraguay	1991	100.5713191	0.474352286
136	Paraguay	1992	103.3864549	0.479314169
136	Paraguay	1993	103.1519031	0.4846142
136	Paraguay	1994	102.184079	0.490226935
136	Paraguay	1995	101.7393315	0.496336876
136	Paraguay	1996	101.2298163	0.502322804
136	Paraguay	1997	94.38930523	0.508286948
136	Paraguay	1998	93.40412381	0.513819896
136	Paraguay	1999	91.41650312	0.518720583
136	Paraguay	2000	88.35556321	0.523007793
136	Paraguay	2001	84.2356424	0.526916763
136	Paraguay	2002	82.32326192	0.531177358
136	Paraguay	2003	82.46657957	0.535360126
136	Paraguay	2004	84.73765154	0.539556655
136	Paraguay	2005	84.39708689	0.543622697
136	Paraguay	2006	85.32468192	0.547943305
136	Paraguay	2007	85.30491538	0.552758676
136	Paraguay	2008	85.23179119	0.558016903
136	Paraguay	2009	88.23973647	0.562900059
136	Paraguay	2010	90.68344945	0.568846562
136	Paraguay	2011	89.80683138	0.574954463
136	Paraguay	2012	88.23684306	0.580650186
136	Paraguay	2013	89.6758247	0.587247042
136	Paraguay	2014	87.3305814	0.593953153
136	Paraguay	2015	89.14420715	0.600484125
136	Paraguay	2016	93.92682022	0.607018145
136	Paraguay	2017	90.67558038	0.613581591
136	Paraguay	2018	93.16581818	0.620032362
136	Paraguay	2019	94.89669873	0.626070139

136	Paraguay	2020	87.91737942	0.631057689
136	Paraguay	2021	86.53048465	0.635718099
139	Algeria	1990	310.6341238	0.460486906
139	Algeria	1991	297.4552311	0.468319197
139	Algeria	1992	293.792127	0.475950937
139	Algeria	1993	290.6346909	0.483255591
139	Algeria	1994	286.7644725	0.49045066
139	Algeria	1995	284.4496875	0.497957543
139	Algeria	1996	281.5402891	0.505990967
139	Algeria	1997	278.59995	0.514056566
139	Algeria	1998	273.0721067	0.522382419
139	Algeria	1999	273.5303766	0.530785583
139	Algeria	2000	271.670332	0.539481508
139	Algeria	2001	268.6630981	0.547529277
139	Algeria	2002	262.12485	0.555172222
139	Algeria	2003	260.3902956	0.562536956
139	Algeria	2004	254.3230502	0.569352852
139	Algeria	2005	248.3956887	0.576001965
139	Algeria	2006	244.3504216	0.582126362
139	Algeria	2007	240.3660264	0.587790538
139	Algeria	2008	237.5300174	0.593190973
139	Algeria	2009	234.049006	0.597870384
139	Algeria	2010	234.598894	0.602823535
139	Algeria	2011	232.6041888	0.607825529
139	Algeria	2012	229.0052271	0.612703533
139	Algeria	2013	225.9257488	0.617422204
139	Algeria	2014	224.6900017	0.622087292
139	Algeria	2015	222.2557645	0.626745568
139	Algeria	2016	218.6199179	0.631710736
139	Algeria	2017	216.9932881	0.636973176
139	Algeria	2018	217.4242355	0.642500039
139	Algeria	2019	216.6609004	0.648210785
139	Algeria	2020	214.3116743	0.653651466
139	Algeria	2021	212.0152452	0.659500924
140	Bahrain	1990	408.0706529	0.584578852
140	Bahrain	1991	404.9236604	0.590596897
140	Bahrain	1992	394.6993264	0.596040229
140	Bahrain	1993	376.9796996	0.602165622
140	Bahrain	1994	377.5150645	0.608133725
140	Bahrain	1995	386.3023124	0.613850722
140	Bahrain	1996	385.8521718	0.619485376
140	Bahrain	1997	390.2330044	0.624596062
140	Bahrain	1998	378.7960932	0.630738301
140	Bahrain	1999	361.8868137	0.638083187
140	Bahrain	2000	350.5279312	0.646750697
140	Bahrain	2001	344.0453862	0.657196064
140	Bahrain	2002	333.4852535	0.664901201
140	Bahrain	2003	340.0602729	0.669564111
140	Bahrain	2004	334.0982076	0.674147775
140	Bahrain	2005	324.1234358	0.679790598

140	Bahrain	2006	299.7813158	0.686144289
140	Bahrain	2007	279.2598588	0.693045147
140	Bahrain	2008	261.7994471	0.699806387
140	Bahrain	2009	249.5599115	0.70488584
140	Bahrain	2010	250.9585854	0.70790262
140	Bahrain	2011	241.1854027	0.708958739
140	Bahrain	2012	227.1042764	0.710362418
140	Bahrain	2013	201.6030525	0.713343156
140	Bahrain	2014	162.1291952	0.71676806
140	Bahrain	2015	161.519305	0.719926605
140	Bahrain	2016	163.3529741	0.723611297
140	Bahrain	2017	161.9289603	0.729323103
140	Bahrain	2018	157.8786143	0.736192583
140	Bahrain	2019	156.7840941	0.742847804
140	Bahrain	2020	159.3684267	0.748103083
140	Bahrain	2021	161.6237068	0.753043204
141	Egypt	1990	379.2026717	0.417182742
141	Egypt	1991	371.1875976	0.426909452
141	Egypt	1992	356.1133364	0.437744555
141	Egypt	1993	360.3084027	0.448490053
141	Egypt	1994	366.0870848	0.458727004
141	Egypt	1995	358.7349338	0.468035368
141	Egypt	1996	353.4916189	0.476116627
141	Egypt	1997	355.581224	0.482970886
141	Egypt	1998	358.0665853	0.489184071
141	Egypt	1999	353.2729968	0.495502619
141	Egypt	2000	336.474244	0.502169886
141	Egypt	2001	356.6838151	0.508716824
141	Egypt	2002	373.6198565	0.515038129
141	Egypt	2003	388.894696	0.520623395
141	Egypt	2004	386.694076	0.525181678
141	Egypt	2005	381.7171949	0.528665483
141	Egypt	2006	387.9729942	0.530395807
141	Egypt	2007	388.9503961	0.529457594
141	Egypt	2008	398.2725791	0.524743833
141	Egypt	2009	406.563274	0.516255831
141	Egypt	2010	409.1811571	0.507715477
141	Egypt	2011	400.7914579	0.504594044
141	Egypt	2012	406.6289991	0.509157068
141	Egypt	2013	404.3065033	0.519334464
141	Egypt	2014	410.9351337	0.531749809
141	Egypt	2015	413.5358884	0.544370149
141	Egypt	2016	387.2232678	0.556012469
141	Egypt	2017	368.2564455	0.566914632
141	Egypt	2018	365.3879447	0.577442786
141	Egypt	2019	357.4037918	0.587736877
141	Egypt	2020	351.4680042	0.597363341
141	Egypt	2021	347.7279154	0.606787094
142	Iran (Islamic Republic of)	1990	259.8760734	0.453799944
142	Iran (Islamic Republic of)	1991	257.7677116	0.468982858

142	Iran (Islamic Republic of)	1992	255.8435866	0.480626697
142	Iran (Islamic Republic of)	1993	253.2270192	0.492277025
142	Iran (Islamic Republic of)	1994	250.3670789	0.505072857
142	Iran (Islamic Republic of)	1995	247.1296447	0.517531494
142	Iran (Islamic Republic of)	1996	243.0986687	0.52860072
142	Iran (Islamic Republic of)	1997	239.543159	0.537727779
142	Iran (Islamic Republic of)	1998	236.1877119	0.546366017
142	Iran (Islamic Republic of)	1999	233.5451346	0.555624332
142	Iran (Islamic Republic of)	2000	230.803644	0.565474561
142	Iran (Islamic Republic of)	2001	228.883597	0.574618208
142	Iran (Islamic Republic of)	2002	224.5985731	0.584097268
142	Iran (Islamic Republic of)	2003	219.3240796	0.593963012
142	Iran (Islamic Republic of)	2004	211.8388889	0.60371745
142	Iran (Islamic Republic of)	2005	204.2266758	0.613291056
142	Iran (Islamic Republic of)	2006	197.2503619	0.621268095
142	Iran (Islamic Republic of)	2007	189.5735904	0.627749442
142	Iran (Islamic Republic of)	2008	182.0482676	0.63286223
142	Iran (Islamic Republic of)	2009	176.2938633	0.63739838
142	Iran (Islamic Republic of)	2010	169.7012556	0.64255917
142	Iran (Islamic Republic of)	2011	163.5503452	0.647899089
142	Iran (Islamic Republic of)	2012	159.3291704	0.651500587
142	Iran (Islamic Republic of)	2013	158.9352224	0.654475289
142	Iran (Islamic Republic of)	2014	157.3371648	0.657961511
142	Iran (Islamic Republic of)	2015	159.4786269	0.662057987
142	Iran (Islamic Republic of)	2016	159.9939451	0.667652445
142	Iran (Islamic Republic of)	2017	159.3883462	0.674343462
142	Iran (Islamic Republic of)	2018	156.3498925	0.680909621
142	Iran (Islamic Republic of)	2019	155.8622299	0.686741662
142	Iran (Islamic Republic of)	2020	147.2621087	0.691918763
142	Iran (Islamic Republic of)	2021	146.110536	0.697207398
143	Iraq	1990	249.6744896	0.412044173
143	Iraq	1991	248.4943992	0.415814116
143	Iraq	1992	251.3041811	0.420353594
143	Iraq	1993	253.2245827	0.424834933
143	Iraq	1994	250.8434659	0.428751731
143	Iraq	1995	251.9722121	0.432075545
143	Iraq	1996	251.9177565	0.436818479
143	Iraq	1997	251.1388237	0.443104027
143	Iraq	1998	248.2651819	0.452384092
143	Iraq	1999	244.6692856	0.462736441
143	Iraq	2000	241.6073346	0.472376399
143	Iraq	2001	238.8011845	0.48145832
143	Iraq	2002	236.4487697	0.488664737
143	Iraq	2003	236.7931653	0.492174445
143	Iraq	2004	234.3673746	0.498933881
143	Iraq	2005	231.8430672	0.504719431
143	Iraq	2006	229.5414002	0.510918049
143	Iraq	2007	231.9349224	0.517698972
143	Iraq	2008	233.8584669	0.525242908
143	Iraq	2009	232.2065162	0.533200914

143	Iraq	2010	229.0277689	0.542343278
143	Iraq	2011	224.350058	0.553048555
143	Iraq	2012	220.6281776	0.564745467
143	Iraq	2013	216.2000238	0.576968998
143	Iraq	2014	211.9665168	0.588317652
143	Iraq	2015	212.9168149	0.599494007
143	Iraq	2016	209.9839058	0.611508646
143	Iraq	2017	208.8739086	0.622674532
143	Iraq	2018	216.903018	0.633232108
143	Iraq	2019	229.6132163	0.643635318
143	Iraq	2020	256.1586248	0.65330038
143	Iraq	2021	254.8309618	0.662626231
144	Jordan	1990	199.1572568	0.539147468
144	Jordan	1991	195.7977111	0.542819411
144	Jordan	1992	191.7304854	0.547458846
144	Jordan	1993	196.5110604	0.55218235
144	Jordan	1994	195.7620985	0.557254335
144	Jordan	1995	197.1718912	0.562571536
144	Jordan	1996	197.6000961	0.567529655
144	Jordan	1997	193.6436608	0.572581508
144	Jordan	1998	189.8815348	0.57764357
144	Jordan	1999	186.0188598	0.582759199
144	Jordan	2000	186.9096401	0.587909394
144	Jordan	2001	190.4483788	0.593182803
144	Jordan	2002	186.0656028	0.599000385
144	Jordan	2003	171.9210172	0.60494595
144	Jordan	2004	172.6906893	0.611934404
144	Jordan	2005	177.8022253	0.619909228
144	Jordan	2006	172.3344291	0.627803813
144	Jordan	2007	147.7955346	0.635670276
144	Jordan	2008	133.8131656	0.643892693
144	Jordan	2009	128.0165135	0.652196319
144	Jordan	2010	123.4571528	0.659949495
144	Jordan	2011	120.5587142	0.66739668
144	Jordan	2012	118.0191709	0.674568925
144	Jordan	2013	114.813636	0.681119962
144	Jordan	2014	110.9765321	0.68713404
144	Jordan	2015	105.4837373	0.692952032
144	Jordan	2016	102.3456971	0.698473302
144	Jordan	2017	99.32156866	0.703923826
144	Jordan	2018	95.18780806	0.709412879
144	Jordan	2019	94.63390223	0.714998946
144	Jordan	2020	97.09193877	0.72006824
144	Jordan	2021	98.28284047	0.725307227
145	Kuwait	1990	198.3181109	0.664517904
145	Kuwait	1991	161.5365541	0.668809621
145	Kuwait	1992	139.8275282	0.67015911
145	Kuwait	1993	149.3608221	0.670036252
145	Kuwait	1994	151.6727435	0.671393213
145	Kuwait	1995	174.4120279	0.677215523

145	Kuwait	1996	164.3082701	0.686093304
145	Kuwait	1997	190.6723426	0.695789389
145	Kuwait	1998	173.8635106	0.704383736
145	Kuwait	1999	181.6695463	0.711841328
145	Kuwait	2000	167.8242926	0.718357562
145	Kuwait	2001	158.3544992	0.722840631
145	Kuwait	2002	169.2666732	0.726816976
145	Kuwait	2003	159.2563673	0.731778685
145	Kuwait	2004	155.675794	0.738153707
145	Kuwait	2005	158.3340718	0.746384913
145	Kuwait	2006	157.671016	0.754928493
145	Kuwait	2007	144.1349493	0.762411484
145	Kuwait	2008	160.7295778	0.770002615
145	Kuwait	2009	144.8706465	0.777546551
145	Kuwait	2010	127.7318921	0.784873929
145	Kuwait	2011	115.7549266	0.791812065
145	Kuwait	2012	115.1363925	0.798356988
145	Kuwait	2013	108.3283577	0.804583834
145	Kuwait	2014	102.9266769	0.810743233
145	Kuwait	2015	112.845801	0.816998413
145	Kuwait	2016	102.2990895	0.823026527
145	Kuwait	2017	104.0814422	0.828886987
145	Kuwait	2018	99.84060626	0.834371461
145	Kuwait	2019	104.8210422	0.839688307
145	Kuwait	2020	114.7234525	0.843459812
145	Kuwait	2021	109.0962361	0.846651055
146	Lebanon	1990	235.272028	0.536718969
146	Lebanon	1991	222.8205734	0.539197492
146	Lebanon	1992	218.4209484	0.541793516
146	Lebanon	1993	215.1524092	0.544581306
146	Lebanon	1994	204.7723646	0.547684033
146	Lebanon	1995	196.0872014	0.55147172
146	Lebanon	1996	189.9402283	0.556436159
146	Lebanon	1997	185.6242729	0.562120818
146	Lebanon	1998	178.6948066	0.56905907
146	Lebanon	1999	169.2447658	0.576078528
146	Lebanon	2000	160.9644443	0.582698277
146	Lebanon	2001	151.3133742	0.589185502
146	Lebanon	2002	136.0097782	0.595978788
146	Lebanon	2003	125.7130078	0.60296724
146	Lebanon	2004	119.5343791	0.61084576
146	Lebanon	2005	117.268091	0.619391355
146	Lebanon	2006	115.4942986	0.627791217
146	Lebanon	2007	114.3198137	0.636938234
146	Lebanon	2008	113.4265569	0.646667663
146	Lebanon	2009	112.5046123	0.657433186
146	Lebanon	2010	110.8867926	0.668804525
146	Lebanon	2011	111.9088004	0.680920617
146	Lebanon	2012	111.5634984	0.693910983
146	Lebanon	2013	111.2753681	0.704811001

146	Lebanon	2014	109.2709489	0.712423873
146	Lebanon	2015	106.2457944	0.718697292
146	Lebanon	2016	101.0900752	0.724583347
146	Lebanon	2017	96.6706249	0.729963294
146	Lebanon	2018	93.73579356	0.734746243
146	Lebanon	2019	92.69228773	0.738644009
146	Lebanon	2020	92.32600358	0.74200972
146	Lebanon	2021	92.09427475	0.744746351
147	Libya	1990	162.0727056	0.527998168
147	Libya	1991	159.5228385	0.541432399
147	Libya	1992	151.7401415	0.554468227
147	Libya	1993	149.1949537	0.566798415
147	Libya	1994	146.28241	0.578651416
147	Libya	1995	142.8039488	0.589578634
147	Libya	1996	140.1047372	0.600350044
147	Libya	1997	146.5924225	0.610620315
147	Libya	1998	156.7822372	0.619973984
147	Libya	1999	161.7803501	0.628600604
147	Libya	2000	166.1876516	0.637047935
147	Libya	2001	175.4919338	0.644685131
147	Libya	2002	183.6765892	0.651669147
147	Libya	2003	185.5913808	0.659345125
147	Libya	2004	183.7195185	0.666913926
147	Libya	2005	185.467405	0.675322268
147	Libya	2006	178.0233892	0.683702
147	Libya	2007	180.9209137	0.691855134
147	Libya	2008	187.4032352	0.698873253
147	Libya	2009	192.3607163	0.704998471
147	Libya	2010	192.7467361	0.711362496
147	Libya	2011	192.0267294	0.711466657
147	Libya	2012	184.3224619	0.716700693
147	Libya	2013	185.3293473	0.71766525
147	Libya	2014	185.173222	0.716042207
147	Libya	2015	183.9124734	0.713651367
147	Libya	2016	187.4418177	0.71076014
147	Libya	2017	187.1398015	0.710666264
147	Libya	2018	186.1219781	0.712720438
147	Libya	2019	183.799641	0.716157239
147	Libya	2020	181.5300343	0.720270393
147	Libya	2021	178.473559	0.725771399
148	Morocco	1990	296.2049437	0.35807287
148	Morocco	1991	296.3172132	0.364984629
148	Morocco	1992	295.7283313	0.371187194
148	Morocco	1993	297.158248	0.376778977
148	Morocco	1994	293.1283563	0.382849451
148	Morocco	1995	290.405232	0.38816411
148	Morocco	1996	287.6501375	0.394150096
148	Morocco	1997	287.3466683	0.399504009
148	Morocco	1998	285.1309163	0.405038792
148	Morocco	1999	285.0177213	0.410303191

148	Morocco	2000	281.9778472	0.415344026
148	Morocco	2001	280.5565833	0.420595133
148	Morocco	2002	275.7599451	0.42577575
148	Morocco	2003	276.3190137	0.43121781
148	Morocco	2004	276.1277301	0.436822352
148	Morocco	2005	276.0337461	0.442531339
148	Morocco	2006	275.4274518	0.44867616
148	Morocco	2007	275.6680858	0.455036325
148	Morocco	2008	272.8024354	0.461803552
148	Morocco	2009	270.469145	0.468929893
148	Morocco	2010	268.9986685	0.476269799
148	Morocco	2011	267.6522274	0.483922152
148	Morocco	2012	266.0768915	0.491603392
148	Morocco	2013	266.7496025	0.499528607
148	Morocco	2014	267.2754744	0.507443265
148	Morocco	2015	269.4134349	0.515582893
148	Morocco	2016	269.5835324	0.523518592
148	Morocco	2017	271.5609792	0.531610862
148	Morocco	2018	272.1819473	0.539729671
148	Morocco	2019	271.6141946	0.547837444
148	Morocco	2020	270.0869553	0.555246069
148	Morocco	2021	267.1520948	0.562698301
149	Palestine	1990	288.9930961	0.40179221
149	Palestine	1991	288.9349779	0.40572715
149	Palestine	1992	287.64296	0.411146869
149	Palestine	1993	286.6135467	0.416779649
149	Palestine	1994	284.8738678	0.423136673
149	Palestine	1995	283.0081212	0.429307027
149	Palestine	1996	281.6380415	0.434731371
149	Palestine	1997	279.7156731	0.440901776
149	Palestine	1998	276.5179786	0.447782415
149	Palestine	1999	273.9324912	0.454909692
149	Palestine	2000	273.0077087	0.460681496
149	Palestine	2001	273.2201168	0.465299102
149	Palestine	2002	275.0127682	0.468600257
149	Palestine	2003	273.2733122	0.472881732
149	Palestine	2004	276.7019602	0.478527322
149	Palestine	2005	275.1434508	0.485320586
149	Palestine	2006	273.8569527	0.491890407
149	Palestine	2007	272.333959	0.498978426
149	Palestine	2008	266.902168	0.506849479
149	Palestine	2009	261.8952684	0.515815836
149	Palestine	2010	257.3017837	0.525711905
149	Palestine	2011	250.6375472	0.53677763
149	Palestine	2012	233.3909466	0.548295425
149	Palestine	2013	218.7877409	0.559163481
149	Palestine	2014	208.9867663	0.569239882
149	Palestine	2015	206.7276002	0.579204261
149	Palestine	2016	197.7573463	0.58949277
149	Palestine	2017	194.0959921	0.599157187

149	Palestine	2018	189.2697949	0.608124861
149	Palestine	2019	187.0871999	0.616466839
149	Palestine	2020	183.4361285	0.623810015
149	Palestine	2021	188.6824894	0.631011665
150	Oman	1990	301.1631701	0.429270949
150	Oman	1991	298.1469383	0.442268122
150	Oman	1992	296.3608978	0.457737972
150	Oman	1993	294.4212424	0.474999033
150	Oman	1994	293.1694343	0.493513107
150	Oman	1995	290.6176261	0.513194359
150	Oman	1996	287.8853861	0.533246394
150	Oman	1997	285.3749083	0.554315537
150	Oman	1998	282.8202668	0.574474902
150	Oman	1999	280.0110331	0.59239847
150	Oman	2000	276.100901	0.608421541
150	Oman	2001	262.4520978	0.622160627
150	Oman	2002	256.6742503	0.635162126
150	Oman	2003	252.6492094	0.647249093
150	Oman	2004	244.0883669	0.657626118
150	Oman	2005	243.6534562	0.666401231
150	Oman	2006	243.4063385	0.675353885
150	Oman	2007	240.3908523	0.685055583
150	Oman	2008	239.4955878	0.694952263
150	Oman	2009	240.9498146	0.703098571
150	Oman	2010	242.7952924	0.710688695
150	Oman	2011	239.9985722	0.718264528
150	Oman	2012	231.9274661	0.726450074
150	Oman	2013	225.5484038	0.73343249
150	Oman	2014	222.3635435	0.738743772
150	Oman	2015	228.8318977	0.743597182
150	Oman	2016	229.2689292	0.748797193
150	Oman	2017	222.9637429	0.753786058
150	Oman	2018	201.4058061	0.759179514
150	Oman	2019	194.4900996	0.764527863
150	Oman	2020	202.1995677	0.768854216
150	Oman	2021	179.2517015	0.773391602
151	Qatar	1990	384.9484155	0.651208376
151	Qatar	1991	387.4143488	0.655338767
151	Qatar	1992	385.8010178	0.660673763
151	Qatar	1993	388.8197449	0.666194399
151	Qatar	1994	330.1856328	0.672025384
151	Qatar	1995	277.4741092	0.678320339
151	Qatar	1996	319.7068993	0.684835919
151	Qatar	1997	378.3179152	0.693109654
151	Qatar	1998	375.4581877	0.701409064
151	Qatar	1999	373.3129794	0.708880981
151	Qatar	2000	364.5381923	0.714831609
151	Qatar	2001	370.1885646	0.720831064
151	Qatar	2002	369.3628494	0.727112174
151	Qatar	2003	356.5292962	0.733536599

151	Qatar	2004	341.2254727	0.740223208
151	Qatar	2005	330.2263689	0.747189359
151	Qatar	2006	313.9364356	0.754123028
151	Qatar	2007	297.8279386	0.760960134
151	Qatar	2008	275.3342584	0.767659233
151	Qatar	2009	254.8633148	0.774276849
151	Qatar	2010	230.0861344	0.780807074
151	Qatar	2011	198.7078473	0.78721245
151	Qatar	2012	174.6162814	0.793494901
151	Qatar	2013	150.5503665	0.799611555
151	Qatar	2014	133.6371179	0.80572571
151	Qatar	2015	121.297803	0.811872852
151	Qatar	2016	114.7444044	0.817979841
151	Qatar	2017	112.9984473	0.824016868
151	Qatar	2018	111.8897303	0.829962127
151	Qatar	2019	109.188988	0.835805284
151	Qatar	2020	123.0483116	0.841467395
151	Qatar	2021	123.1649871	0.846860584
152	Saudi Arabia	1990	225.5208609	0.538954515
152	Saudi Arabia	1991	228.5552964	0.549907717
152	Saudi Arabia	1992	231.0970017	0.561144266
152	Saudi Arabia	1993	233.2310601	0.572186913
152	Saudi Arabia	1994	234.5481149	0.582878871
152	Saudi Arabia	1995	235.8497403	0.593251423
152	Saudi Arabia	1996	236.720077	0.603648418
152	Saudi Arabia	1997	237.6003856	0.613749116
152	Saudi Arabia	1998	237.9720073	0.623518214
152	Saudi Arabia	1999	238.112877	0.632796447
152	Saudi Arabia	2000	236.3814362	0.642311774
152	Saudi Arabia	2001	235.5307177	0.651502092
152	Saudi Arabia	2002	233.804103	0.660356856
152	Saudi Arabia	2003	234.0250985	0.669839577
152	Saudi Arabia	2004	235.1203921	0.679917856
152	Saudi Arabia	2005	239.7058066	0.690520852
152	Saudi Arabia	2006	241.6988736	0.701152613
152	Saudi Arabia	2007	240.0420363	0.711442141
152	Saudi Arabia	2008	237.1434347	0.721960205
152	Saudi Arabia	2009	232.8313544	0.731158488
152	Saudi Arabia	2010	227.5559706	0.740300669
152	Saudi Arabia	2011	220.5762445	0.749959451
152	Saudi Arabia	2012	215.3648007	0.759054935
152	Saudi Arabia	2013	209.9247985	0.767387864
152	Saudi Arabia	2014	201.9699244	0.775178133
152	Saudi Arabia	2015	203.3598539	0.782300802
152	Saudi Arabia	2016	198.6163113	0.788867882
152	Saudi Arabia	2017	197.6778899	0.794886288
152	Saudi Arabia	2018	193.2725749	0.800656985
152	Saudi Arabia	2019	189.5418475	0.806087841
152	Saudi Arabia	2020	187.3090883	0.810541861
152	Saudi Arabia	2021	185.9013151	0.815143493

153	Syrian Arab Republic	1990	370.8017484	0.430492643
153	Syrian Arab Republic	1991	385.2798778	0.437907493
153	Syrian Arab Republic	1992	410.2724086	0.445904236
153	Syrian Arab Republic	1993	399.6527835	0.45414786
153	Syrian Arab Republic	1994	399.8197651	0.462437298
153	Syrian Arab Republic	1995	401.320617	0.470692043
153	Syrian Arab Republic	1996	405.3462199	0.478743085
153	Syrian Arab Republic	1997	405.8223801	0.48623235
153	Syrian Arab Republic	1998	405.0011	0.493707992
153	Syrian Arab Republic	1999	402.221804	0.500474869
153	Syrian Arab Republic	2000	407.0019988	0.507143686
153	Syrian Arab Republic	2001	398.5169489	0.513850111
153	Syrian Arab Republic	2002	403.5864313	0.521309744
153	Syrian Arab Republic	2003	379.4615615	0.528935756
153	Syrian Arab Republic	2004	372.7404389	0.538084469
153	Syrian Arab Republic	2005	364.5856156	0.549750449
153	Syrian Arab Republic	2006	361.792586	0.560484761
153	Syrian Arab Republic	2007	365.0606	0.57002812
153	Syrian Arab Republic	2008	378.5127631	0.578990493
153	Syrian Arab Republic	2009	369.5034733	0.587789118
153	Syrian Arab Republic	2010	366.691361	0.595967764
153	Syrian Arab Republic	2011	365.481764	0.601524456
153	Syrian Arab Republic	2012	359.0819195	0.602872601
153	Syrian Arab Republic	2013	358.710997	0.602410134
153	Syrian Arab Republic	2014	360.2202354	0.60223576
153	Syrian Arab Republic	2015	357.03853	0.602282914
153	Syrian Arab Republic	2016	357.1413276	0.602911181
153	Syrian Arab Republic	2017	358.3338043	0.604896366
153	Syrian Arab Republic	2018	358.5878545	0.608507164
153	Syrian Arab Republic	2019	357.8744138	0.613336836
153	Syrian Arab Republic	2020	355.8860685	0.617727593
153	Syrian Arab Republic	2021	353.0071557	0.623004075
154	Tunisia	1990	218.2600952	0.471138521
154	Tunisia	1991	229.0469336	0.479543057
154	Tunisia	1992	229.4642958	0.488285281
154	Tunisia	1993	230.4890322	0.496852039
154	Tunisia	1994	227.1640753	0.505564882
154	Tunisia	1995	236.606187	0.51419694
154	Tunisia	1996	235.4664006	0.523294274
154	Tunisia	1997	227.9936914	0.532064044
154	Tunisia	1998	227.8698705	0.540498161
154	Tunisia	1999	225.7001336	0.548724563
154	Tunisia	2000	223.9607564	0.55667265
154	Tunisia	2001	222.3354903	0.56442101
154	Tunisia	2002	218.8850187	0.571719534
154	Tunisia	2003	217.2930497	0.578882669
154	Tunisia	2004	212.7432302	0.586032594
154	Tunisia	2005	208.4835929	0.592937759
154	Tunisia	2006	204.3335664	0.599758513
154	Tunisia	2007	200.5602821	0.606696784

154	Tunisia	2008	197.260438	0.613529322
154	Tunisia	2009	194.334215	0.620106833
154	Tunisia	2010	191.4189348	0.626432774
154	Tunisia	2011	185.3404311	0.63201984
154	Tunisia	2012	182.9011953	0.637578345
154	Tunisia	2013	180.3637104	0.643008512
154	Tunisia	2014	179.5141466	0.648297395
154	Tunisia	2015	176.9526425	0.653413538
154	Tunisia	2016	175.6889684	0.658353517
154	Tunisia	2017	172.6116835	0.663222845
154	Tunisia	2018	169.6077258	0.668218997
154	Tunisia	2019	166.2088518	0.673170552
154	Tunisia	2020	163.9402168	0.677615148
154	Tunisia	2021	163.5886587	0.682432216
155	Turkey	1990	204.646014	0.461606984
155	Turkey	1991	194.8700352	0.469244066
155	Turkey	1992	190.6648584	0.476814569
155	Turkey	1993	189.2340501	0.48465697
155	Turkey	1994	188.3200732	0.49161995
155	Turkey	1995	185.9853633	0.49882484
155	Turkey	1996	182.2578754	0.506238546
155	Turkey	1997	177.762687	0.513933356
155	Turkey	1998	171.0266998	0.521509898
155	Turkey	1999	163.8832019	0.528512416
155	Turkey	2000	155.0873038	0.535866331
155	Turkey	2001	149.159379	0.54235913
155	Turkey	2002	143.8947072	0.549023644
155	Turkey	2003	139.9536199	0.556102527
155	Turkey	2004	136.3343362	0.564024
155	Turkey	2005	132.9857845	0.572495886
155	Turkey	2006	130.4596576	0.581383179
155	Turkey	2007	130.2020456	0.590442387
155	Turkey	2008	132.5406161	0.599212061
155	Turkey	2009	138.6795197	0.607104618
155	Turkey	2010	135.4945027	0.615597228
155	Turkey	2011	133.532599	0.624787211
155	Turkey	2012	133.6096497	0.634097092
155	Turkey	2013	136.1367318	0.643723823
155	Turkey	2014	140.3363896	0.653388835
155	Turkey	2015	144.5343514	0.662971739
155	Turkey	2016	145.0634213	0.671948148
155	Turkey	2017	140.0492112	0.681096272
155	Turkey	2018	134.4612283	0.689895867
155	Turkey	2019	136.5276231	0.698263774
155	Turkey	2020	137.4807635	0.705799744
155	Turkey	2021	133.3767882	0.712692673
156	United Arab Emirates	1990	266.9189648	0.644412271
156	United Arab Emirates	1991	264.8578643	0.660739899
156	United Arab Emirates	1992	236.3855846	0.675926211
156	United Arab Emirates	1993	227.0948501	0.689463801

156	United Arab Emirates	1994	222.5203052	0.701656792
156	United Arab Emirates	1995	208.8116086	0.713138288
156	United Arab Emirates	1996	202.6365504	0.724181976
156	United Arab Emirates	1997	205.9802248	0.734723175
156	United Arab Emirates	1998	225.9591383	0.744476623
156	United Arab Emirates	1999	239.8720894	0.753253916
156	United Arab Emirates	2000	258.7424833	0.760969094
156	United Arab Emirates	2001	267.3903442	0.767858793
156	United Arab Emirates	2002	237.6427831	0.774346424
156	United Arab Emirates	2003	222.1763609	0.781143992
156	United Arab Emirates	2004	231.5827965	0.788525951
156	United Arab Emirates	2005	240.9104982	0.796428086
156	United Arab Emirates	2006	253.8986491	0.805537023
156	United Arab Emirates	2007	293.8834098	0.814870878
156	United Arab Emirates	2008	313.7053855	0.822793435
156	United Arab Emirates	2009	304.1392605	0.828304165
156	United Arab Emirates	2010	260.7126842	0.831284254
156	United Arab Emirates	2011	254.9459503	0.832912804
156	United Arab Emirates	2012	242.3090543	0.83358611
156	United Arab Emirates	2013	254.1498797	0.833803818
156	United Arab Emirates	2014	270.6562992	0.833858727
156	United Arab Emirates	2015	280.1226158	0.834175498
156	United Arab Emirates	2016	290.8548521	0.83522071
156	United Arab Emirates	2017	275.6561513	0.837213553
156	United Arab Emirates	2018	261.2874038	0.83990014
156	United Arab Emirates	2019	245.2433893	0.842985326
156	United Arab Emirates	2020	177.7958013	0.846151976
156	United Arab Emirates	2021	167.7082797	0.849317734
157	Yemen	1990	320.3518477	0.215664586
157	Yemen	1991	317.041344	0.222885409
157	Yemen	1992	315.0030193	0.230415046
157	Yemen	1993	312.9306382	0.238025246
157	Yemen	1994	311.1188261	0.245879426
157	Yemen	1995	308.4993114	0.253962572
157	Yemen	1996	305.5997235	0.262278613
157	Yemen	1997	302.938754	0.270782536
157	Yemen	1998	299.4063837	0.279500507
157	Yemen	1999	298.3143278	0.288261301
157	Yemen	2000	295.9770993	0.297145157
157	Yemen	2001	291.9355189	0.306112424
157	Yemen	2002	287.1773133	0.315065023
157	Yemen	2003	282.4315916	0.324081689
157	Yemen	2004	279.7494907	0.333371285
157	Yemen	2005	275.2105045	0.343462562
157	Yemen	2006	270.9933188	0.353472947
157	Yemen	2007	266.5130716	0.363301218
157	Yemen	2008	264.8289744	0.373140288
157	Yemen	2009	261.7412095	0.382762252
157	Yemen	2010	256.6604453	0.392936641
157	Yemen	2011	257.8628172	0.401471801

157	Yemen	2012	258.5535578	0.409561349
157	Yemen	2013	256.2105351	0.417642405
157	Yemen	2014	250.5353137	0.425189667
157	Yemen	2015	254.6766086	0.430197789
157	Yemen	2016	251.6458527	0.434232983
157	Yemen	2017	251.0491123	0.437549155
157	Yemen	2018	251.8548888	0.440727991
157	Yemen	2019	258.0282935	0.4440014
157	Yemen	2020	261.569536	0.446893069
157	Yemen	2021	263.5396622	0.450376375
160	Afghanistan	1990	361.3923176	0.173832165
160	Afghanistan	1991	359.4366357	0.17647255
160	Afghanistan	1992	358.2275394	0.179633724
160	Afghanistan	1993	359.3420466	0.180183713
160	Afghanistan	1994	361.5302473	0.17851866
160	Afghanistan	1995	362.2591697	0.178279128
160	Afghanistan	1996	362.8213426	0.178085404
160	Afghanistan	1997	362.902982	0.177884124
160	Afghanistan	1998	362.35145	0.177513403
160	Afghanistan	1999	361.5898407	0.177075239
160	Afghanistan	2000	361.6770701	0.177025772
160	Afghanistan	2001	359.8651824	0.177773144
160	Afghanistan	2002	348.3131768	0.18380843
160	Afghanistan	2003	341.2658918	0.190518991
160	Afghanistan	2004	336.005547	0.196903541
160	Afghanistan	2005	330.5599736	0.203928133
160	Afghanistan	2006	325.0209157	0.211074194
160	Afghanistan	2007	319.2211766	0.219627611
160	Afghanistan	2008	314.4892898	0.228089171
160	Afghanistan	2009	309.2608237	0.237900824
160	Afghanistan	2010	305.0593926	0.247759949
160	Afghanistan	2011	303.3812279	0.257041676
160	Afghanistan	2012	298.7325149	0.266484407
160	Afghanistan	2013	298.1344752	0.275636558
160	Afghanistan	2014	297.889709	0.284030351
160	Afghanistan	2015	295.6030137	0.291849505
160	Afghanistan	2016	291.6502287	0.299630696
160	Afghanistan	2017	287.9076228	0.307424618
160	Afghanistan	2018	286.934537	0.314866093
160	Afghanistan	2019	286.2076673	0.322454047
160	Afghanistan	2020	282.1804184	0.329830068
160	Afghanistan	2021	280.0347651	0.337199998
161	Bangladesh	1990	119.1241349	0.228548934
161	Bangladesh	1991	111.6178553	0.237207413
161	Bangladesh	1992	111.4314835	0.245247956
161	Bangladesh	1993	110.4808919	0.25209072
161	Bangladesh	1994	109.3950121	0.258997294
161	Bangladesh	1995	111.7054737	0.265293918
161	Bangladesh	1996	110.5022116	0.271177781
161	Bangladesh	1997	109.4527864	0.27777328

161	Bangladesh	1998	108.2678539	0.28451594
161	Bangladesh	1999	111.9048864	0.290951431
161	Bangladesh	2000	113.8278741	0.297251976
161	Bangladesh	2001	113.142037	0.303188546
161	Bangladesh	2002	115.0388637	0.308640093
161	Bangladesh	2003	115.2209956	0.31436004
161	Bangladesh	2004	113.2859827	0.320922851
161	Bangladesh	2005	113.8169218	0.32809051
161	Bangladesh	2006	116.990621	0.335690598
161	Bangladesh	2007	117.5557157	0.343932546
161	Bangladesh	2008	114.8004593	0.352520033
161	Bangladesh	2009	113.6999636	0.361481513
161	Bangladesh	2010	114.7712261	0.370598956
161	Bangladesh	2011	106.0731274	0.380124886
161	Bangladesh	2012	101.4301383	0.390623552
161	Bangladesh	2013	101.1835491	0.401706478
161	Bangladesh	2014	106.690389	0.413263169
161	Bangladesh	2015	105.0624016	0.425482121
161	Bangladesh	2016	105.4010981	0.437709906
161	Bangladesh	2017	109.3846268	0.449752413
161	Bangladesh	2018	109.3504838	0.461717749
161	Bangladesh	2019	108.7546891	0.473264998
161	Bangladesh	2020	108.0717103	0.483079169
161	Bangladesh	2021	107.5026744	0.492420885
162	Bhutan	1990	106.7290845	0.21503985
162	Bhutan	1991	105.9740572	0.22131244
162	Bhutan	1992	105.7093308	0.228148272
162	Bhutan	1993	106.1985881	0.236004687
162	Bhutan	1994	106.235426	0.244331246
162	Bhutan	1995	106.6981307	0.253291957
162	Bhutan	1996	104.8245735	0.262362746
162	Bhutan	1997	105.9537434	0.271487422
162	Bhutan	1998	105.4436688	0.280440384
162	Bhutan	1999	104.2124919	0.289510488
162	Bhutan	2000	106.2480986	0.298804875
162	Bhutan	2001	105.9657909	0.308200652
162	Bhutan	2002	105.9825739	0.317943536
162	Bhutan	2003	107.2699873	0.327670422
162	Bhutan	2004	107.7221865	0.337286495
162	Bhutan	2005	108.3806164	0.346998578
162	Bhutan	2006	108.2764915	0.356824209
162	Bhutan	2007	108.5031856	0.367560498
162	Bhutan	2008	108.4264842	0.378233543
162	Bhutan	2009	108.9229548	0.388977162
162	Bhutan	2010	109.2020576	0.399751936
162	Bhutan	2011	108.5043459	0.410149249
162	Bhutan	2012	110.8033186	0.419443216
162	Bhutan	2013	110.1761826	0.427790675
162	Bhutan	2014	112.3946029	0.43496927
162	Bhutan	2015	111.1716803	0.441810748

162	Bhutan	2016	111.2189596	0.448401855
162	Bhutan	2017	110.3437383	0.454280497
162	Bhutan	2018	110.8015418	0.459624711
162	Bhutan	2019	110.9701964	0.464633353
162	Bhutan	2020	111.2098546	0.468714479
162	Bhutan	2021	110.9750646	0.473062378
163	India	1990	137.8713072	0.332593603
163	India	1991	137.7803572	0.338591779
163	India	1992	139.7276085	0.344591727
163	India	1993	138.3213518	0.350685397
163	India	1994	139.2376268	0.35699603
163	India	1995	143.5339377	0.363418501
163	India	1996	143.2815009	0.370018644
163	India	1997	142.4819229	0.376358763
163	India	1998	142.2662541	0.382817848
163	India	1999	134.2409632	0.389453889
163	India	2000	132.2624528	0.395752331
163	India	2001	133.4226254	0.401837472
163	India	2002	133.3181995	0.407411414
163	India	2003	134.8427125	0.413115783
163	India	2004	133.3408819	0.419184961
163	India	2005	132.6418883	0.425913062
163	India	2006	136.5944144	0.433321558
163	India	2007	141.9929386	0.44135146
163	India	2008	143.8099219	0.449235851
163	India	2009	141.5155837	0.457672332
163	India	2010	143.2124732	0.467018996
163	India	2011	146.9847726	0.476832909
163	India	2012	151.3365206	0.487210876
163	India	2013	159.0630591	0.498317981
163	India	2014	167.2872548	0.5097221
163	India	2015	159.6007452	0.521242719
163	India	2016	157.6637498	0.532220023
163	India	2017	152.9323849	0.542388507
163	India	2018	153.7893489	0.551930326
163	India	2019	153.7803471	0.560809927
163	India	2020	152.3826177	0.568138149
163	India	2021	151.1666504	0.575401649
164	Nepal	1990	130.1110119	0.199560654
164	Nepal	1991	128.6437134	0.205687443
164	Nepal	1992	126.4867061	0.21211309
164	Nepal	1993	126.9130988	0.218843431
164	Nepal	1994	125.871661	0.22607306
164	Nepal	1995	124.7014793	0.233253154
164	Nepal	1996	122.678082	0.240700616
164	Nepal	1997	121.8735422	0.248382797
164	Nepal	1998	121.2830781	0.256227179
164	Nepal	1999	120.5586113	0.26435918
164	Nepal	2000	120.151645	0.272719115
164	Nepal	2001	118.7271234	0.281287467

164	Nepal	2002	118.2665834	0.289513608
164	Nepal	2003	118.4312129	0.297772595
164	Nepal	2004	119.3738651	0.30619653
164	Nepal	2005	120.6829435	0.314661796
164	Nepal	2006	122.5312426	0.323056346
164	Nepal	2007	125.2560505	0.33144561
164	Nepal	2008	127.5060066	0.340150706
164	Nepal	2009	129.9414186	0.348701893
164	Nepal	2010	132.2025953	0.3568681
164	Nepal	2011	135.3018122	0.364642605
164	Nepal	2012	140.4099706	0.372437588
164	Nepal	2013	140.5174115	0.380022326
164	Nepal	2014	142.9150002	0.387689088
164	Nepal	2015	140.7822991	0.395021154
164	Nepal	2016	140.4923558	0.401628776
164	Nepal	2017	135.9305324	0.408463874
164	Nepal	2018	136.3895925	0.415236684
164	Nepal	2019	136.235433	0.422007252
164	Nepal	2020	136.6038272	0.427417089
164	Nepal	2021	136.5280952	0.433174635
165	Pakistan	1990	140.7060389	0.310467621
165	Pakistan	1991	142.4537422	0.316265667
165	Pakistan	1992	145.5833536	0.322248051
165	Pakistan	1993	150.1633646	0.328219252
165	Pakistan	1994	155.1908196	0.334433128
165	Pakistan	1995	160.5309917	0.340926296
165	Pakistan	1996	163.187541	0.347656203
165	Pakistan	1997	166.0198314	0.354218627
165	Pakistan	1998	167.1482733	0.360875336
165	Pakistan	1999	168.4558556	0.367641397
165	Pakistan	2000	169.6080946	0.374319132
165	Pakistan	2001	170.3640729	0.380593927
165	Pakistan	2002	171.1490824	0.386629992
165	Pakistan	2003	172.2360487	0.392719263
165	Pakistan	2004	172.0551541	0.399084167
165	Pakistan	2005	171.7767903	0.405667437
165	Pakistan	2006	171.4510734	0.41223483
165	Pakistan	2007	171.3420562	0.418519756
165	Pakistan	2008	172.6133593	0.4243482
165	Pakistan	2009	174.1901336	0.430019833
165	Pakistan	2010	175.7724106	0.435640657
165	Pakistan	2011	178.2797516	0.441455585
165	Pakistan	2012	183.39311	0.447096599
165	Pakistan	2013	187.2892925	0.452823095
165	Pakistan	2014	190.4397519	0.458781424
165	Pakistan	2015	188.9728734	0.464792668
165	Pakistan	2016	190.4368055	0.471022168
165	Pakistan	2017	186.4557631	0.477544137
165	Pakistan	2018	185.318847	0.484326128
165	Pakistan	2019	184.8225613	0.491022981

165	Pakistan	2020	184.4147418	0.497364406
165	Pakistan	2021	183.447358	0.504028689
168	Angola	1990	126.9152359	0.270736935
168	Angola	1991	126.5969591	0.275160625
168	Angola	1992	126.5281105	0.2792315
168	Angola	1993	127.8107812	0.281867703
168	Angola	1994	128.9081701	0.284487594
168	Angola	1995	128.6809922	0.287447813
168	Angola	1996	126.7596051	0.291421279
168	Angola	1997	126.0515303	0.295433993
168	Angola	1998	127.4142806	0.299553353
168	Angola	1999	128.0437244	0.303720042
168	Angola	2000	127.8310665	0.307603312
168	Angola	2001	125.4462343	0.311697534
168	Angola	2002	123.6125916	0.316706571
168	Angola	2003	123.2136854	0.321777362
168	Angola	2004	124.4270716	0.327395266
168	Angola	2005	121.7905858	0.333785188
168	Angola	2006	121.8789219	0.341012622
168	Angola	2007	120.5734173	0.348846841
168	Angola	2008	121.7944407	0.357082005
168	Angola	2009	121.0999951	0.364407744
168	Angola	2010	120.2655053	0.372033537
168	Angola	2011	119.8410705	0.380006613
168	Angola	2012	119.4063559	0.387891857
168	Angola	2013	118.4091675	0.395796329
168	Angola	2014	115.5522423	0.403720086
168	Angola	2015	116.406179	0.411372026
168	Angola	2016	117.3973174	0.418822062
168	Angola	2017	118.8241024	0.426177082
168	Angola	2018	121.0150058	0.433355005
168	Angola	2019	123.417627	0.440454598
168	Angola	2020	125.3913614	0.447283992
168	Angola	2021	125.4817408	0.453721949
169	Central African Republic	1990	165.9631235	0.216825191
169	Central African Republic	1991	166.7352982	0.220191656
169	Central African Republic	1992	165.5956032	0.223007541
169	Central African Republic	1993	167.9550301	0.225451125
169	Central African Republic	1994	169.769495	0.228487724
169	Central African Republic	1995	170.1121519	0.231696792
169	Central African Republic	1996	171.9164838	0.233989591
169	Central African Republic	1997	171.5023811	0.236671999
169	Central African Republic	1998	172.3342017	0.239743529
169	Central African Republic	1999	172.8205633	0.24298853
169	Central African Republic	2000	171.4815465	0.246021132
169	Central African Republic	2001	169.8827647	0.24924674
169	Central African Republic	2002	169.2081623	0.252524369
169	Central African Republic	2003	167.4413846	0.255101519
169	Central African Republic	2004	165.8276834	0.257778778
169	Central African Republic	2005	164.161986	0.260451918

169	Central African Republic	2006	163.3593011	0.263712446
169	Central African Republic	2007	161.6188559	0.267332195
169	Central African Republic	2008	160.3327295	0.271158118
169	Central African Republic	2009	158.6577436	0.275338515
169	Central African Republic	2010	159.749602	0.279981382
169	Central African Republic	2011	161.0652236	0.285097245
169	Central African Republic	2012	159.3574239	0.290460023
169	Central African Republic	2013	156.7472987	0.29010493
169	Central African Republic	2014	153.6020753	0.289999498
169	Central African Republic	2015	155.9329819	0.290827871
169	Central African Republic	2016	153.609184	0.2924304
169	Central African Republic	2017	152.6004328	0.294752243
169	Central African Republic	2018	153.4176999	0.298011223
169	Central African Republic	2019	153.2017334	0.301953064
169	Central African Republic	2020	151.1470426	0.305426298
169	Central African Republic	2021	150.2649594	0.30916769
170	Congo	1990	186.7270462	0.420654655
170	Congo	1991	186.5504613	0.426734374
170	Congo	1992	187.0577365	0.431987662
170	Congo	1993	189.4164008	0.436606594
170	Congo	1994	194.6062641	0.440168347
170	Congo	1995	197.3959713	0.443744125
170	Congo	1996	200.2369743	0.447566642
170	Congo	1997	201.1245708	0.451026961
170	Congo	1998	196.9650941	0.454445045
170	Congo	1999	195.1074414	0.45717654
170	Congo	2000	191.2590191	0.460764814
170	Congo	2001	183.8671417	0.463751682
170	Congo	2002	178.5653644	0.467037268
170	Congo	2003	177.1127426	0.470134374
170	Congo	2004	174.7947046	0.473612436
170	Congo	2005	171.7221411	0.478137627
170	Congo	2006	169.6152824	0.483355822
170	Congo	2007	169.0572977	0.48764074
170	Congo	2008	168.1158978	0.492822855
170	Congo	2009	164.3639315	0.498299969
170	Congo	2010	162.5843384	0.505542849
170	Congo	2011	160.8276048	0.513815777
170	Congo	2012	160.538364	0.522928283
170	Congo	2013	161.984154	0.531551679
170	Congo	2014	161.1763358	0.540594486
170	Congo	2015	162.8687759	0.54841403
170	Congo	2016	160.6580928	0.555108608
170	Congo	2017	159.2063187	0.5618341
170	Congo	2018	159.8542387	0.56779397
170	Congo	2019	159.9105239	0.573373665
170	Congo	2020	159.8800099	0.578451546
170	Congo	2021	158.9452255	0.583075236
171	Democratic Republic of the Congo	1990	130.6261392	0.28984321
171	Democratic Republic of the Congo	1991	129.7005126	0.290431567

171	Democratic Republic of the Congo	1992	128.3315113	0.290959242
171	Democratic Republic of the Congo	1993	126.3490298	0.28962879
171	Democratic Republic of the Congo	1994	126.0316927	0.286859146
171	Democratic Republic of the Congo	1995	126.5057381	0.283967532
171	Democratic Republic of the Congo	1996	128.6483179	0.28041316
171	Democratic Republic of the Congo	1997	124.1807217	0.276336503
171	Democratic Republic of the Congo	1998	123.0561072	0.271813823
171	Democratic Republic of the Congo	1999	121.1569068	0.267367662
171	Democratic Republic of the Congo	2000	120.3609454	0.262456747
171	Democratic Republic of the Congo	2001	118.8369569	0.257857505
171	Democratic Republic of the Congo	2002	115.9749456	0.25413636
171	Democratic Republic of the Congo	2003	116.0130918	0.252333885
171	Democratic Republic of the Congo	2004	113.9041098	0.252470919
171	Democratic Republic of the Congo	2005	112.0595922	0.254161023
171	Democratic Republic of the Congo	2006	111.5875827	0.257355123
171	Democratic Republic of the Congo	2007	111.4479454	0.262272267
171	Democratic Republic of the Congo	2008	113.0151732	0.268103872
171	Democratic Republic of the Congo	2009	111.9150213	0.274361144
171	Democratic Republic of the Congo	2010	110.9284988	0.282223754
171	Democratic Republic of the Congo	2011	110.4467633	0.290872953
171	Democratic Republic of the Congo	2012	109.8616436	0.300454783
171	Democratic Republic of the Congo	2013	107.9420931	0.310831256
171	Democratic Republic of the Congo	2014	107.129186	0.321622927
171	Democratic Republic of the Congo	2015	108.9148191	0.332421307
171	Democratic Republic of the Congo	2016	107.9242815	0.341988787
171	Democratic Republic of the Congo	2017	107.1452025	0.351102035
171	Democratic Republic of the Congo	2018	108.3481247	0.360287677
171	Democratic Republic of the Congo	2019	109.2124802	0.369340684
171	Democratic Republic of the Congo	2020	111.0056074	0.376712271
171	Democratic Republic of the Congo	2021	112.3156526	0.383179849
172	Equatorial Guinea	1990	159.2898615	0.268783629
172	Equatorial Guinea	1991	157.0596596	0.275296992
172	Equatorial Guinea	1992	154.0801014	0.283487764
172	Equatorial Guinea	1993	153.585939	0.292236032
172	Equatorial Guinea	1994	153.9721505	0.30068634
172	Equatorial Guinea	1995	155.4918722	0.310443551
172	Equatorial Guinea	1996	157.4582736	0.324601221
172	Equatorial Guinea	1997	151.1453872	0.350550963
172	Equatorial Guinea	1998	149.0584799	0.371237163
172	Equatorial Guinea	1999	147.3844252	0.391128023
172	Equatorial Guinea	2000	145.2109591	0.41138345
172	Equatorial Guinea	2001	140.2804783	0.433590057
172	Equatorial Guinea	2002	135.9264645	0.451939
172	Equatorial Guinea	2003	135.5779574	0.467846833
172	Equatorial Guinea	2004	134.7762871	0.483879612
172	Equatorial Guinea	2005	131.8866237	0.498683715
172	Equatorial Guinea	2006	131.0519574	0.511579828
172	Equatorial Guinea	2007	129.1692973	0.524076461
172	Equatorial Guinea	2008	129.5715106	0.53701899
172	Equatorial Guinea	2009	128.9458487	0.548514683

172	Equatorial Guinea	2010	129.3504682	0.559109377
172	Equatorial Guinea	2011	130.9249989	0.570277424
172	Equatorial Guinea	2012	128.3223022	0.581833899
172	Equatorial Guinea	2013	125.0499154	0.592800613
172	Equatorial Guinea	2014	123.3265336	0.603473167
172	Equatorial Guinea	2015	132.1151136	0.613134624
172	Equatorial Guinea	2016	136.1995153	0.621858157
172	Equatorial Guinea	2017	135.2265956	0.630234538
172	Equatorial Guinea	2018	137.9018825	0.638022823
172	Equatorial Guinea	2019	141.2239799	0.645303093
172	Equatorial Guinea	2020	141.9992628	0.652124903
172	Equatorial Guinea	2021	141.8046428	0.657857456
173	Gabon	1990	135.2663287	0.455421187
173	Gabon	1991	133.8974157	0.461563098
173	Gabon	1992	133.433471	0.467024048
173	Gabon	1993	133.6271406	0.472394463
173	Gabon	1994	136.3423917	0.478061947
173	Gabon	1995	139.0900402	0.483826218
173	Gabon	1996	141.1936915	0.48946146
173	Gabon	1997	140.2318951	0.495074162
173	Gabon	1998	140.8588804	0.50037255
173	Gabon	1999	141.8858658	0.50480635
173	Gabon	2000	144.0109783	0.509133087
173	Gabon	2001	144.1583687	0.513045709
173	Gabon	2002	141.5227112	0.516917621
173	Gabon	2003	141.9928435	0.521051412
173	Gabon	2004	141.0652491	0.525479071
173	Gabon	2005	137.2792522	0.530488575
173	Gabon	2006	136.1371357	0.534797716
173	Gabon	2007	134.4598842	0.539441424
173	Gabon	2008	135.1967176	0.544110892
173	Gabon	2009	132.9277767	0.548591319
173	Gabon	2010	129.9061052	0.554022706
173	Gabon	2011	129.4217322	0.560232082
173	Gabon	2012	127.4240267	0.566842964
173	Gabon	2013	127.2098494	0.57380813
173	Gabon	2014	125.2382963	0.580915656
173	Gabon	2015	128.9933068	0.588365
173	Gabon	2016	128.396578	0.596062305
173	Gabon	2017	126.9828105	0.603816252
173	Gabon	2018	128.2192569	0.61172438
173	Gabon	2019	128.5549995	0.619904727
173	Gabon	2020	128.3304372	0.627609647
173	Gabon	2021	126.7711485	0.634691393
175	Burundi	1990	104.9402734	0.20586736
175	Burundi	1991	106.9705541	0.20849572
175	Burundi	1992	108.9325648	0.210864619
175	Burundi	1993	111.1101725	0.213249386
175	Burundi	1994	112.8484573	0.215222973
175	Burundi	1995	114.413514	0.215631676

175	Burundi	1996	115.5457397	0.215526325
175	Burundi	1997	114.9565095	0.21576182
175	Burundi	1998	113.8967256	0.216402603
175	Burundi	1999	112.0221213	0.216855767
175	Burundi	2000	109.9431976	0.216869858
175	Burundi	2001	105.6109054	0.216988726
175	Burundi	2002	102.4291424	0.217782477
175	Burundi	2003	99.17938654	0.218961537
175	Burundi	2004	97.82331644	0.220918709
175	Burundi	2005	95.48779529	0.223084372
175	Burundi	2006	92.97817029	0.22582189
175	Burundi	2007	89.11728598	0.228996819
175	Burundi	2008	86.51534279	0.232853551
175	Burundi	2009	86.15731059	0.23694304
175	Burundi	2010	85.67079563	0.241447786
175	Burundi	2011	86.14390301	0.246277277
175	Burundi	2012	86.30614392	0.251572303
175	Burundi	2013	85.17392108	0.257191355
175	Burundi	2014	85.03006015	0.262794722
175	Burundi	2015	85.38522329	0.267327938
175	Burundi	2016	85.36260415	0.271587852
175	Burundi	2017	84.5256472	0.275840505
175	Burundi	2018	84.944225	0.27973329
175	Burundi	2019	85.27472876	0.283442515
175	Burundi	2020	85.19327199	0.286496
175	Burundi	2021	85.7968566	0.289374365
176	Comoros	1990	77.2555976	0.270048116
176	Comoros	1991	77.75885377	0.279145649
176	Comoros	1992	78.08176774	0.288506271
176	Comoros	1993	78.42730135	0.297607417
176	Comoros	1994	79.10598186	0.305706587
176	Comoros	1995	79.2111306	0.314220341
176	Comoros	1996	79.57145605	0.322249069
176	Comoros	1997	79.92182182	0.330148125
176	Comoros	1998	79.35188504	0.337517914
176	Comoros	1999	77.85747655	0.344774694
176	Comoros	2000	77.65589538	0.351875506
176	Comoros	2001	76.30140216	0.359211999
176	Comoros	2002	75.52104122	0.366486127
176	Comoros	2003	74.07035905	0.373410566
176	Comoros	2004	71.59098583	0.380035976
176	Comoros	2005	71.87755104	0.386891845
176	Comoros	2006	70.19647771	0.393521434
176	Comoros	2007	71.1966348	0.399051094
176	Comoros	2008	71.45369992	0.404649755
176	Comoros	2009	69.69029566	0.410253575
176	Comoros	2010	68.94503912	0.415543376
176	Comoros	2011	69.36564938	0.421092972
176	Comoros	2012	68.98537866	0.426729957
176	Comoros	2013	68.97606461	0.432789932

176	Comoros	2014	70.57036987	0.438844505
176	Comoros	2015	71.08790152	0.444712394
176	Comoros	2016	71.14761166	0.450506979
176	Comoros	2017	70.97837768	0.456280841
176	Comoros	2018	70.76194501	0.461951344
176	Comoros	2019	71.53072113	0.467388014
176	Comoros	2020	72.58712247	0.472003132
176	Comoros	2021	73.16341394	0.475978688
177	Djibouti	1990	69.58520292	0.337781789
177	Djibouti	1991	70.33430049	0.338770011
177	Djibouti	1992	71.68137575	0.340986061
177	Djibouti	1993	73.95306748	0.344904394
177	Djibouti	1994	74.14863853	0.347981404
177	Djibouti	1995	76.42342581	0.351245633
177	Djibouti	1996	77.651778	0.354385634
177	Djibouti	1997	78.54113743	0.358096401
177	Djibouti	1998	81.04488452	0.361596764
177	Djibouti	1999	82.59677671	0.365305211
177	Djibouti	2000	84.11990673	0.369015095
177	Djibouti	2001	84.92298036	0.372853155
177	Djibouti	2002	85.87040352	0.376650004
177	Djibouti	2003	85.81300597	0.380686615
177	Djibouti	2004	87.47001172	0.384987706
177	Djibouti	2005	88.06021599	0.389568706
177	Djibouti	2006	88.01512781	0.395037471
177	Djibouti	2007	88.29470992	0.400618546
177	Djibouti	2008	88.1805593	0.406658303
177	Djibouti	2009	87.99322946	0.411969335
177	Djibouti	2010	88.2719675	0.41699935
177	Djibouti	2011	88.96778417	0.42231614
177	Djibouti	2012	89.03029838	0.42771144
177	Djibouti	2013	88.834082	0.433360437
177	Djibouti	2014	87.5181343	0.439455722
177	Djibouti	2015	89.36539963	0.445855285
177	Djibouti	2016	89.762425	0.453442854
177	Djibouti	2017	90.64709653	0.460440152
177	Djibouti	2018	90.43951599	0.467705672
177	Djibouti	2019	90.952127	0.475263243
177	Djibouti	2020	91.03784261	0.481879945
177	Djibouti	2021	91.49478676	0.487958371
178	Eritrea	1990	83.06502818	0.216028239
178	Eritrea	1991	83.0895445	0.222574737
178	Eritrea	1992	81.92614879	0.230296066
178	Eritrea	1993	81.6022898	0.239385041
178	Eritrea	1994	83.152314	0.250752541
178	Eritrea	1995	86.12336395	0.261498485
178	Eritrea	1996	86.83187595	0.272527055
178	Eritrea	1997	87.50172655	0.283551721
178	Eritrea	1998	88.16670394	0.293764828
178	Eritrea	1999	88.88173656	0.30314234

178	Eritrea	2000	89.45838091	0.310248162
178	Eritrea	2001	89.03263825	0.317247988
178	Eritrea	2002	89.31285994	0.323566585
178	Eritrea	2003	89.3990663	0.328593581
178	Eritrea	2004	89.4563966	0.33287938
178	Eritrea	2005	89.55290184	0.336682511
178	Eritrea	2006	89.51745134	0.339835824
178	Eritrea	2007	89.617323	0.342843174
178	Eritrea	2008	90.15672308	0.343427801
178	Eritrea	2009	90.68674406	0.345147743
178	Eritrea	2010	90.94144081	0.348146177
178	Eritrea	2011	90.49507401	0.354163147
178	Eritrea	2012	90.58833803	0.360112751
178	Eritrea	2013	90.59491977	0.364343653
178	Eritrea	2014	87.96608552	0.372527588
178	Eritrea	2015	89.6809442	0.376935162
178	Eritrea	2016	88.99181695	0.382171666
178	Eritrea	2017	90.18760703	0.385936029
178	Eritrea	2018	90.25221705	0.391154884
178	Eritrea	2019	90.60973317	0.396330558
178	Eritrea	2020	90.76100003	0.400459411
178	Eritrea	2021	90.94820209	0.403863943
179	Ethiopia	1990	81.26878091	0.148033885
179	Ethiopia	1991	80.40076615	0.14959971
179	Ethiopia	1992	79.50532194	0.150174386
179	Ethiopia	1993	78.64234354	0.151582364
179	Ethiopia	1994	78.04468271	0.152982637
179	Ethiopia	1995	78.00443917	0.155085605
179	Ethiopia	1996	77.06871016	0.158127229
179	Ethiopia	1997	76.36142985	0.161874743
179	Ethiopia	1998	75.19680947	0.16507771
179	Ethiopia	1999	74.01156476	0.168903545
179	Ethiopia	2000	72.53903844	0.17341853
179	Ethiopia	2001	70.58419533	0.178972969
179	Ethiopia	2002	67.93529998	0.184138013
179	Ethiopia	2003	66.20538607	0.188694814
179	Ethiopia	2004	65.43271189	0.195030701
179	Ethiopia	2005	64.7121233	0.202868243
179	Ethiopia	2006	64.03291189	0.211524229
179	Ethiopia	2007	62.80237981	0.221819931
179	Ethiopia	2008	61.79270041	0.232872186
179	Ethiopia	2009	60.7885957	0.244265818
179	Ethiopia	2010	60.0763963	0.25608146
179	Ethiopia	2011	59.15508705	0.267880862
179	Ethiopia	2012	57.60346884	0.279006958
179	Ethiopia	2013	56.24231044	0.289885267
179	Ethiopia	2014	54.58292844	0.300484548
179	Ethiopia	2015	53.96823998	0.310736419
179	Ethiopia	2016	53.61556106	0.320431867
179	Ethiopia	2017	54.23666009	0.329469244

179	Ethiopia	2018	54.51307062	0.338050075
179	Ethiopia	2019	54.86027301	0.346422664
179	Ethiopia	2020	55.41753733	0.35321635
179	Ethiopia	2021	55.81201586	0.358823295
180	Kenya	1990	39.81407327	0.333850293
180	Kenya	1991	40.41030554	0.34047622
180	Kenya	1992	41.12604809	0.346298636
180	Kenya	1993	41.63913692	0.351611769
180	Kenya	1994	42.11850685	0.356779194
180	Kenya	1995	42.68887062	0.361535807
180	Kenya	1996	43.5062268	0.366203076
180	Kenya	1997	44.85237692	0.370436465
180	Kenya	1998	46.40657415	0.374581401
180	Kenya	1999	47.74704815	0.378630009
180	Kenya	2000	49.8409799	0.382395616
180	Kenya	2001	51.35884818	0.386188415
180	Kenya	2002	53.65503141	0.389886256
180	Kenya	2003	55.52722717	0.39368048
180	Kenya	2004	57.25053034	0.397743651
180	Kenya	2005	58.08273543	0.402444011
180	Kenya	2006	58.87937532	0.407745764
180	Kenya	2007	59.93547219	0.413736227
180	Kenya	2008	61.33173738	0.419738147
180	Kenya	2009	62.62732083	0.426123888
180	Kenya	2010	63.54955574	0.433305839
180	Kenya	2011	64.57887081	0.44089383
180	Kenya	2012	64.59917843	0.448666396
180	Kenya	2013	63.96560843	0.456666942
180	Kenya	2014	63.52698722	0.464724302
180	Kenya	2015	63.78354815	0.473084051
180	Kenya	2016	63.22845046	0.481709072
180	Kenya	2017	62.6294303	0.490296619
180	Kenya	2018	62.24028969	0.499101274
180	Kenya	2019	61.66571695	0.508004211
180	Kenya	2020	61.78158309	0.516136572
180	Kenya	2021	61.49918928	0.523768077
181	Madagascar	1990	82.43103816	0.279889465
181	Madagascar	1991	85.51669097	0.280429589
181	Madagascar	1992	89.95707944	0.280501283
181	Madagascar	1993	95.93152507	0.280204299
181	Madagascar	1994	93.84516355	0.279615149
181	Madagascar	1995	94.61884717	0.279164003
181	Madagascar	1996	95.89335868	0.279066493
181	Madagascar	1997	97.14473653	0.27942619
181	Madagascar	1998	99.21247084	0.280317671
181	Madagascar	1999	98.36633482	0.281571072
181	Madagascar	2000	99.39612894	0.283190721
181	Madagascar	2001	99.67130864	0.28566818
181	Madagascar	2002	101.0969965	0.287153149
181	Madagascar	2003	102.1465698	0.289675457

181	Madagascar	2004	101.6992665	0.292629885
181	Madagascar	2005	100.1886622	0.295919327
181	Madagascar	2006	98.71439561	0.299723392
181	Madagascar	2007	97.27698252	0.304026245
181	Madagascar	2008	97.70397462	0.308990265
181	Madagascar	2009	98.02541948	0.313450891
181	Madagascar	2010	97.82445487	0.318353184
181	Madagascar	2011	97.61230866	0.323801043
181	Madagascar	2012	96.56141137	0.329947541
181	Madagascar	2013	96.84146265	0.336519232
181	Madagascar	2014	97.16810529	0.343352611
181	Madagascar	2015	98.22713892	0.350443595
181	Madagascar	2016	98.50972018	0.358026341
181	Madagascar	2017	98.22565768	0.366235587
181	Madagascar	2018	98.81872732	0.374969644
181	Madagascar	2019	99.03004465	0.384209196
181	Madagascar	2020	99.53678541	0.392778709
181	Madagascar	2021	100.0249516	0.400246943
182	Malawi	1990	69.58104087	0.204010245
182	Malawi	1991	70.52735189	0.206043786
182	Malawi	1992	72.29946748	0.206732359
182	Malawi	1993	73.95959261	0.208091205
182	Malawi	1994	76.82090626	0.209144566
182	Malawi	1995	79.49123323	0.211756232
182	Malawi	1996	82.28297535	0.215264475
182	Malawi	1997	85.00511223	0.219416252
182	Malawi	1998	88.26364849	0.22312233
182	Malawi	1999	89.61610715	0.227068368
182	Malawi	2000	90.72582271	0.23090081
182	Malawi	2001	90.01272705	0.234020507
182	Malawi	2002	90.98471534	0.237602155
182	Malawi	2003	91.86300166	0.241559228
182	Malawi	2004	93.5410685	0.24643454
182	Malawi	2005	91.5010586	0.251672318
182	Malawi	2006	90.46022807	0.257595649
182	Malawi	2007	89.46361622	0.265226928
182	Malawi	2008	89.88124132	0.273689856
182	Malawi	2009	89.80283363	0.282296509
182	Malawi	2010	89.39379898	0.29117962
182	Malawi	2011	88.36431574	0.300448509
182	Malawi	2012	86.41836094	0.309197624
182	Malawi	2013	84.8975161	0.317936213
182	Malawi	2014	84.3219423	0.327042426
182	Malawi	2015	84.86125536	0.336140138
182	Malawi	2016	84.76307742	0.344731542
182	Malawi	2017	84.14641876	0.353121067
182	Malawi	2018	85.08923362	0.36147041
182	Malawi	2019	85.0990671	0.370114605
182	Malawi	2020	85.23077053	0.37787902
182	Malawi	2021	84.26766357	0.384553634

183	Mauritius	1990	228.1764381	0.544586533
183	Mauritius	1991	208.9602336	0.548209121
183	Mauritius	1992	208.2338684	0.55345463
183	Mauritius	1993	214.0981288	0.56068864
183	Mauritius	1994	210.8202412	0.569092171
183	Mauritius	1995	206.0733513	0.57731939
183	Mauritius	1996	205.0425574	0.584797061
183	Mauritius	1997	209.0155727	0.591497735
183	Mauritius	1998	196.3380642	0.597363039
183	Mauritius	1999	191.6165586	0.602034234
183	Mauritius	2000	182.6097877	0.607087901
183	Mauritius	2001	178.7730271	0.612240817
183	Mauritius	2002	177.2134297	0.617190007
183	Mauritius	2003	176.9509927	0.622764533
183	Mauritius	2004	174.4283386	0.629030193
183	Mauritius	2005	161.9552012	0.63476658
183	Mauritius	2006	146.7943364	0.639545183
183	Mauritius	2007	132.7752557	0.644358656
183	Mauritius	2008	125.8421132	0.649630852
183	Mauritius	2009	120.9676732	0.655000295
183	Mauritius	2010	113.9789429	0.6604698
183	Mauritius	2011	107.4466296	0.666497627
183	Mauritius	2012	106.6185349	0.67312765
183	Mauritius	2013	104.1650172	0.680114023
183	Mauritius	2014	106.0386744	0.686616503
183	Mauritius	2015	102.3693097	0.69223973
183	Mauritius	2016	98.80454132	0.697020562
183	Mauritius	2017	98.17557558	0.701618136
183	Mauritius	2018	104.0186377	0.706266651
183	Mauritius	2019	98.52843982	0.710935045
183	Mauritius	2020	93.27730173	0.714629804
183	Mauritius	2021	97.48678037	0.718260446
184	Mozambique	1990	34.95614477	0.173064715
184	Mozambique	1991	34.98611962	0.175550435
184	Mozambique	1992	35.23402858	0.177285188
184	Mozambique	1993	35.43956589	0.179851807
184	Mozambique	1994	35.49847089	0.182236861
184	Mozambique	1995	35.74205811	0.183427982
184	Mozambique	1996	36.3440801	0.185705869
184	Mozambique	1997	37.16222395	0.188650436
184	Mozambique	1998	37.76171	0.191998629
184	Mozambique	1999	38.50618606	0.195435288
184	Mozambique	2000	39.852064	0.198534397
184	Mozambique	2001	39.8402637	0.202241924
184	Mozambique	2002	40.96635417	0.20639006
184	Mozambique	2003	41.47724467	0.21053449
184	Mozambique	2004	42.62018885	0.214972457
184	Mozambique	2005	44.06084452	0.219692372
184	Mozambique	2006	45.08295349	0.224758087
184	Mozambique	2007	45.86300364	0.230158227

184	Mozambique	2008	47.22630305	0.23579017
184	Mozambique	2009	48.6693561	0.241360656
184	Mozambique	2010	50.4053966	0.2467515
184	Mozambique	2011	51.40976767	0.252232471
184	Mozambique	2012	52.0510996	0.258315562
184	Mozambique	2013	52.50015183	0.265280462
184	Mozambique	2014	52.25367829	0.272892814
184	Mozambique	2015	52.368968	0.281032685
184	Mozambique	2016	51.80135034	0.289152425
184	Mozambique	2017	51.36783354	0.297164772
184	Mozambique	2018	50.8634371	0.305226772
184	Mozambique	2019	50.49293241	0.313174508
184	Mozambique	2020	50.08791746	0.320291182
184	Mozambique	2021	49.06221455	0.326462614
185	Rwanda	1990	97.17063103	0.27509719
185	Rwanda	1991	99.69697635	0.276194698
185	Rwanda	1992	102.5412355	0.277178171
185	Rwanda	1993	105.2169715	0.276980133
185	Rwanda	1994	106.5901704	0.272331258
185	Rwanda	1995	107.9017629	0.271102227
185	Rwanda	1996	107.4151315	0.271097922
185	Rwanda	1997	107.4143727	0.272455861
185	Rwanda	1998	106.1096083	0.274582551
185	Rwanda	1999	102.0660982	0.277014387
185	Rwanda	2000	97.04375942	0.280983454
185	Rwanda	2001	90.44113996	0.286082055
185	Rwanda	2002	85.05176724	0.29295543
185	Rwanda	2003	79.78945125	0.299676918
185	Rwanda	2004	75.60118301	0.306943916
185	Rwanda	2005	71.87008733	0.314219578
185	Rwanda	2006	69.04059154	0.321998519
185	Rwanda	2007	66.44286746	0.329616591
185	Rwanda	2008	64.44739019	0.337039226
185	Rwanda	2009	62.57503854	0.344494633
185	Rwanda	2010	61.74803653	0.351659416
185	Rwanda	2011	61.49049625	0.35894034
185	Rwanda	2012	61.71559727	0.366411821
185	Rwanda	2013	61.20566579	0.373769682
185	Rwanda	2014	61.26605817	0.381533179
185	Rwanda	2015	61.68618078	0.389895415
185	Rwanda	2016	61.61502215	0.398287959
185	Rwanda	2017	61.46493868	0.406411475
185	Rwanda	2018	62.47321509	0.414657912
185	Rwanda	2019	63.01728942	0.423086727
185	Rwanda	2020	64.25554727	0.429896596
185	Rwanda	2021	65.09033858	0.435588706
186	Seychelles	1990	134.1322639	0.575526496
186	Seychelles	1991	132.7734589	0.582698331
186	Seychelles	1992	133.1132327	0.590052919
186	Seychelles	1993	132.525446	0.598044194

186	Seychelles	1994	132.5862149	0.606041268
186	Seychelles	1995	130.0106198	0.613853281
186	Seychelles	1996	128.1066014	0.62213517
186	Seychelles	1997	124.8392368	0.630697732
186	Seychelles	1998	121.815727	0.638438882
186	Seychelles	1999	118.0254852	0.645339666
186	Seychelles	2000	112.8249016	0.651551988
186	Seychelles	2001	110.5522387	0.656490668
186	Seychelles	2002	111.4947559	0.66090779
186	Seychelles	2003	113.1171923	0.664162027
186	Seychelles	2004	111.3492636	0.666461353
186	Seychelles	2005	112.420166	0.669264742
186	Seychelles	2006	110.6609752	0.672149914
186	Seychelles	2007	108.6161155	0.675077438
186	Seychelles	2008	108.7839592	0.676590946
186	Seychelles	2009	108.1352765	0.677847028
186	Seychelles	2010	102.9678287	0.68016364
186	Seychelles	2011	100.2978512	0.683681491
186	Seychelles	2012	101.5548906	0.687629394
186	Seychelles	2013	99.63544589	0.692525734
186	Seychelles	2014	97.64707397	0.697114589
186	Seychelles	2015	97.37035741	0.70172282
186	Seychelles	2016	99.35870503	0.706183623
186	Seychelles	2017	100.4758673	0.711027281
186	Seychelles	2018	100.663386	0.716407819
186	Seychelles	2019	99.7381002	0.722073685
186	Seychelles	2020	84.31004297	0.726663769
186	Seychelles	2021	87.60242655	0.730150775
187	Somalia	1990	66.16633009	0.048848564
187	Somalia	1991	65.58456989	0.049422382
187	Somalia	1992	65.5588878	0.050115531
187	Somalia	1993	65.7155074	0.050797999
187	Somalia	1994	66.47147142	0.051471696
187	Somalia	1995	67.47362215	0.052148606
187	Somalia	1996	68.51103363	0.052840548
187	Somalia	1997	69.93401575	0.053570967
187	Somalia	1998	71.51128979	0.054321527
187	Somalia	1999	72.66264012	0.055071838
187	Somalia	2000	73.66123739	0.055842063
187	Somalia	2001	73.50517904	0.056643165
187	Somalia	2002	73.67512782	0.057457612
187	Somalia	2003	73.45580023	0.05827215
187	Somalia	2004	73.52505272	0.059076945
187	Somalia	2005	73.53531038	0.059898539
187	Somalia	2006	73.58360104	0.060741484
187	Somalia	2007	73.42403476	0.061598151
187	Somalia	2008	73.58140988	0.062475387
187	Somalia	2009	73.75599613	0.063382225
187	Somalia	2010	73.35484197	0.064322128
187	Somalia	2011	73.58839448	0.065306811

187	Somalia	2012	72.73190643	0.066354879
187	Somalia	2013	71.73356571	0.067459486
187	Somalia	2014	71.0070349	0.068609299
187	Somalia	2015	70.9246355	0.069811521
187	Somalia	2016	70.89282767	0.071064047
187	Somalia	2017	71.43827579	0.072334776
187	Somalia	2018	71.21603324	0.073642248
187	Somalia	2019	71.16446506	0.074979811
187	Somalia	2020	70.23315373	0.07636337
187	Somalia	2021	68.48456724	0.077688109
189	United Republic of Tanzania	1990	66.88089072	0.259306074
189	United Republic of Tanzania	1991	68.80555769	0.262416238
189	United Republic of Tanzania	1992	71.30246238	0.264989448
189	United Republic of Tanzania	1993	73.86992443	0.267254915
189	United Republic of Tanzania	1994	76.77840006	0.269417204
189	United Republic of Tanzania	1995	79.22685483	0.272078202
189	United Republic of Tanzania	1996	80.23923309	0.275507909
189	United Republic of Tanzania	1997	81.39569007	0.279259184
189	United Republic of Tanzania	1998	82.75681546	0.28315261
189	United Republic of Tanzania	1999	82.79805766	0.287381679
189	United Republic of Tanzania	2000	82.41780183	0.291827265
189	United Republic of Tanzania	2001	82.78172583	0.296715645
189	United Republic of Tanzania	2002	83.62290721	0.301983995
189	United Republic of Tanzania	2003	84.89395139	0.307615488
189	United Republic of Tanzania	2004	86.36840786	0.313772983
189	United Republic of Tanzania	2005	87.83281933	0.320437339
189	United Republic of Tanzania	2006	89.03752717	0.327234002
189	United Republic of Tanzania	2007	89.66394688	0.334313237
189	United Republic of Tanzania	2008	89.90745008	0.34135263
189	United Republic of Tanzania	2009	90.2542993	0.348378035
189	United Republic of Tanzania	2010	90.88521065	0.355567563
189	United Republic of Tanzania	2011	91.31926102	0.36303632
189	United Republic of Tanzania	2012	91.67622213	0.370412488
189	United Republic of Tanzania	2013	92.25669212	0.377963171
189	United Republic of Tanzania	2014	93.28003208	0.385974579
189	United Republic of Tanzania	2015	95.58031584	0.394277757
189	United Republic of Tanzania	2016	96.99363523	0.402879308
189	United Republic of Tanzania	2017	96.09001539	0.411824974
189	United Republic of Tanzania	2018	96.19414428	0.420985364
189	United Republic of Tanzania	2019	96.2003588	0.430225379
189	United Republic of Tanzania	2020	96.51199336	0.438747238
189	United Republic of Tanzania	2021	96.27637308	0.446568273
190	Uganda	1990	70.72191434	0.187001096
190	Uganda	1991	73.5970458	0.188924156
190	Uganda	1992	75.8375855	0.191003433
190	Uganda	1993	78.82632556	0.193557456
190	Uganda	1994	82.01632997	0.197293193
190	Uganda	1995	84.73741713	0.201835423
190	Uganda	1996	87.1876726	0.207080731
190	Uganda	1997	89.16929523	0.213091696

190	Uganda	1998	92.05366467	0.219712442
190	Uganda	1999	94.42015068	0.226916945
190	Uganda	2000	97.119919	0.234323225
190	Uganda	2001	97.43655524	0.242186388
190	Uganda	2002	97.39075106	0.250552388
190	Uganda	2003	95.59275331	0.259164883
190	Uganda	2004	93.71648563	0.268004611
190	Uganda	2005	91.15196051	0.277369193
190	Uganda	2006	88.49376003	0.287030583
190	Uganda	2007	85.39343779	0.29674042
190	Uganda	2008	82.58884551	0.306748843
190	Uganda	2009	79.92870958	0.316731153
190	Uganda	2010	77.77020478	0.326543661
190	Uganda	2011	76.05675739	0.336217716
190	Uganda	2012	74.70758377	0.345364515
190	Uganda	2013	73.58324556	0.354381615
190	Uganda	2014	72.28181118	0.363430903
190	Uganda	2015	72.80180481	0.372537986
190	Uganda	2016	72.32032133	0.381312226
190	Uganda	2017	70.28734604	0.390082259
190	Uganda	2018	70.09376952	0.399117777
190	Uganda	2019	69.98055209	0.408431557
190	Uganda	2020	70.79974289	0.416846079
190	Uganda	2021	71.02995884	0.423261181
191	Zambia	1990	62.20214533	0.304008549
191	Zambia	1991	64.4371671	0.30584236
191	Zambia	1992	66.60693686	0.307316277
191	Zambia	1993	68.60938619	0.308780208
191	Zambia	1994	70.83458575	0.309223449
191	Zambia	1995	72.96448588	0.309995813
191	Zambia	1996	74.45885857	0.311326691
191	Zambia	1997	75.43570754	0.31308733
191	Zambia	1998	76.32456683	0.314926435
191	Zambia	1999	76.84792424	0.317396437
191	Zambia	2000	77.87913886	0.320625322
191	Zambia	2001	77.94376628	0.324401368
191	Zambia	2002	78.9134588	0.328680122
191	Zambia	2003	79.11579914	0.333721398
191	Zambia	2004	79.27360323	0.339488917
191	Zambia	2005	79.50951753	0.346048864
191	Zambia	2006	80.52583041	0.353540709
191	Zambia	2007	80.70626208	0.362450967
191	Zambia	2008	81.52093825	0.371688496
191	Zambia	2009	82.60847567	0.381667118
191	Zambia	2010	84.83196358	0.392470989
191	Zambia	2011	87.97231105	0.403475629
191	Zambia	2012	89.51663093	0.414574501
191	Zambia	2013	90.52323891	0.425492302
191	Zambia	2014	92.04903516	0.436244006
191	Zambia	2015	94.90280158	0.446744719

191	Zambia	2016	96.55125794	0.457122596
191	Zambia	2017	95.78540183	0.467532631
191	Zambia	2018	95.82882559	0.477887712
191	Zambia	2019	95.60642589	0.487911523
191	Zambia	2020	95.60745123	0.497485561
191	Zambia	2021	92.69806923	0.505948954
193	Botswana	1990	101.4221445	0.418077748
193	Botswana	1991	102.9374712	0.429752286
193	Botswana	1992	105.3248429	0.440490843
193	Botswana	1993	107.6630288	0.450523614
193	Botswana	1994	109.4425477	0.459954967
193	Botswana	1995	110.5458555	0.469266513
193	Botswana	1996	110.1634473	0.478588352
193	Botswana	1997	108.2368081	0.487767857
193	Botswana	1998	105.1555322	0.496193622
193	Botswana	1999	106.777683	0.504978826
193	Botswana	2000	110.7112235	0.513498223
193	Botswana	2001	112.7698599	0.521370182
193	Botswana	2002	113.9898659	0.529252964
193	Botswana	2003	110.8099437	0.537000179
193	Botswana	2004	104.7183436	0.544349223
193	Botswana	2005	98.50100735	0.55170697
193	Botswana	2006	93.49158687	0.55922162
193	Botswana	2007	95.92994212	0.566702496
193	Botswana	2008	99.83191342	0.573883805
193	Botswana	2009	102.7869163	0.579696412
193	Botswana	2010	105.498523	0.58582415
193	Botswana	2011	106.3878771	0.591985457
193	Botswana	2012	102.8625253	0.597739553
193	Botswana	2013	101.439884	0.604064731
193	Botswana	2014	99.14599889	0.610086049
193	Botswana	2015	96.62444892	0.615411124
193	Botswana	2016	89.64665895	0.620797575
193	Botswana	2017	84.433693	0.625704953
193	Botswana	2018	81.70951159	0.630606841
193	Botswana	2019	80.45356188	0.635324093
193	Botswana	2020	80.65250659	0.639275298
193	Botswana	2021	78.60319871	0.642721629
194	Lesotho	1990	50.87064218	0.339155125
194	Lesotho	1991	50.51485206	0.345496571
194	Lesotho	1992	50.5412136	0.35216963
194	Lesotho	1993	50.50632571	0.358780457
194	Lesotho	1994	51.08639428	0.365310801
194	Lesotho	1995	51.06941975	0.371265315
194	Lesotho	1996	51.27861061	0.377221202
194	Lesotho	1997	52.6219036	0.383096648
194	Lesotho	1998	58.05218259	0.388406478
194	Lesotho	1999	64.92053124	0.393495001
194	Lesotho	2000	73.82462569	0.398635802
194	Lesotho	2001	76.30282019	0.403990126

194	Lesotho	2002	80.22904187	0.409021657
194	Lesotho	2003	87.40312755	0.413982571
194	Lesotho	2004	92.80370875	0.418670919
194	Lesotho	2005	96.76770279	0.423211228
194	Lesotho	2006	98.94889685	0.427806732
194	Lesotho	2007	99.3109708	0.432745038
194	Lesotho	2008	99.69798748	0.438340567
194	Lesotho	2009	99.72379376	0.443580127
194	Lesotho	2010	100.9672318	0.448966476
194	Lesotho	2011	101.1588517	0.454530098
194	Lesotho	2012	103.1402053	0.460419689
194	Lesotho	2013	104.249401	0.466692256
194	Lesotho	2014	106.1304234	0.473188157
194	Lesotho	2015	106.3714595	0.479754456
194	Lesotho	2016	104.4085083	0.486144827
194	Lesotho	2017	101.6235399	0.491978155
194	Lesotho	2018	100.3112001	0.497381791
194	Lesotho	2019	99.92178902	0.502457317
194	Lesotho	2020	98.87328326	0.506746655
194	Lesotho	2021	90.89511989	0.510393066
195	Namibia	1990	104.5340711	0.450040233
195	Namibia	1991	104.7772721	0.45453444
195	Namibia	1992	106.1327551	0.460021314
195	Namibia	1993	107.4036808	0.465734135
195	Namibia	1994	109.2022104	0.471873217
195	Namibia	1995	111.4560965	0.477794329
195	Namibia	1996	113.7484335	0.483116378
195	Namibia	1997	116.2995462	0.488012938
195	Namibia	1998	119.9313555	0.492812548
195	Namibia	1999	124.2761802	0.497613862
195	Namibia	2000	129.0087348	0.502406958
195	Namibia	2001	129.4228212	0.506719033
195	Namibia	2002	132.5162335	0.510818275
195	Namibia	2003	132.4189886	0.514574645
195	Namibia	2004	130.5570477	0.519103605
195	Namibia	2005	127.4577547	0.523881736
195	Namibia	2006	122.7868806	0.529350118
195	Namibia	2007	118.4119997	0.535062126
195	Namibia	2008	115.2332288	0.540896995
195	Namibia	2009	113.4901166	0.546516477
195	Namibia	2010	112.9384379	0.552741565
195	Namibia	2011	110.777723	0.559618899
195	Namibia	2012	108.8349126	0.566978697
195	Namibia	2013	107.970847	0.574590344
195	Namibia	2014	108.2104952	0.582321425
195	Namibia	2015	109.2959005	0.58982104
195	Namibia	2016	108.9091929	0.596283059
195	Namibia	2017	108.8818931	0.601754645
195	Namibia	2018	108.6956887	0.606618403
195	Namibia	2019	107.8680618	0.610921091

195	Namibia	2020	107.2194526	0.614435889
195	Namibia	2021	104.1710291	0.617564872
196	South Africa	1990	74.42743468	0.541571435
196	South Africa	1991	74.65493396	0.546921136
196	South Africa	1992	78.26351963	0.552066899
196	South Africa	1993	75.60736365	0.557198439
196	South Africa	1994	79.87992331	0.562448636
196	South Africa	1995	80.80191475	0.567905304
196	South Africa	1996	87.16999093	0.573453988
196	South Africa	1997	98.52998687	0.579099278
196	South Africa	1998	100.0487457	0.584437313
196	South Africa	1999	96.1273807	0.589407349
196	South Africa	2000	97.79846342	0.594093683
196	South Africa	2001	96.37983604	0.597884168
196	South Africa	2002	97.59499695	0.600921676
196	South Africa	2003	99.25954357	0.603515602
196	South Africa	2004	97.40092848	0.606262514
196	South Africa	2005	97.15266099	0.609989979
196	South Africa	2006	98.494779	0.61501469
196	South Africa	2007	97.89830638	0.62060815
196	South Africa	2008	97.27165944	0.625948031
196	South Africa	2009	96.77985293	0.630645626
196	South Africa	2010	95.14106601	0.635268538
196	South Africa	2011	92.54679061	0.639991722
196	South Africa	2012	89.70654604	0.644715661
196	South Africa	2013	86.9728886	0.649430554
196	South Africa	2014	86.42398761	0.653964495
196	South Africa	2015	87.00332356	0.658417761
196	South Africa	2016	85.77033776	0.662642487
196	South Africa	2017	82.34627222	0.666622608
196	South Africa	2018	80.4744771	0.670335365
196	South Africa	2019	77.47048118	0.674041037
196	South Africa	2020	78.54455716	0.677166096
196	South Africa	2021	78.03897285	0.679626598
197	Eswatini	1990	90.34710246	0.399420955
197	Eswatini	1991	90.875047	0.408269969
197	Eswatini	1992	90.45129737	0.416683379
197	Eswatini	1993	90.24182167	0.424802995
197	Eswatini	1994	92.34753922	0.432503472
197	Eswatini	1995	96.04913808	0.440127047
197	Eswatini	1996	100.3085864	0.447399238
197	Eswatini	1997	103.1743378	0.454089854
197	Eswatini	1998	108.0607618	0.460136984
197	Eswatini	1999	113.7614205	0.465990845
197	Eswatini	2000	122.3967381	0.471852843
197	Eswatini	2001	129.1434559	0.477233374
197	Eswatini	2002	135.4087278	0.482474423
197	Eswatini	2003	139.5062965	0.487608829
197	Eswatini	2004	143.5148188	0.492669159
197	Eswatini	2005	145.580186	0.498043849

197	Eswatini	2006	144.7262475	0.503658294
197	Eswatini	2007	144.8537623	0.509260219
197	Eswatini	2008	144.0317173	0.514730896
197	Eswatini	2009	143.443297	0.520427398
197	Eswatini	2010	141.0990592	0.526245802
197	Eswatini	2011	137.4437849	0.53191391
197	Eswatini	2012	136.2878636	0.537798023
197	Eswatini	2013	134.7685663	0.543836416
197	Eswatini	2014	132.145671	0.549613848
197	Eswatini	2015	129.296124	0.555373739
197	Eswatini	2016	126.3066523	0.561061186
197	Eswatini	2017	125.0724296	0.566698493
197	Eswatini	2018	122.8670802	0.572154807
197	Eswatini	2019	120.473442	0.577206305
197	Eswatini	2020	116.2066752	0.581653071
197	Eswatini	2021	106.4373339	0.585459713
198	Zimbabwe	1990	83.54363529	0.398559341
198	Zimbabwe	1991	81.29160231	0.406052877
198	Zimbabwe	1992	81.04422274	0.412083826
198	Zimbabwe	1993	80.20495489	0.4179742
198	Zimbabwe	1994	80.46378464	0.424051564
198	Zimbabwe	1995	82.47524885	0.42892904
198	Zimbabwe	1996	83.8564977	0.434187192
198	Zimbabwe	1997	87.39477313	0.438141988
198	Zimbabwe	1998	96.196116	0.441093135
198	Zimbabwe	1999	106.5783647	0.443246604
198	Zimbabwe	2000	117.4793711	0.444841402
198	Zimbabwe	2001	123.1397	0.446354681
198	Zimbabwe	2002	130.6284903	0.446970895
198	Zimbabwe	2003	135.8412195	0.446002182
198	Zimbabwe	2004	140.1877728	0.443696404
198	Zimbabwe	2005	140.9701446	0.439651657
198	Zimbabwe	2006	140.6381298	0.434405758
198	Zimbabwe	2007	139.8573407	0.42916514
198	Zimbabwe	2008	141.2304147	0.422448974
198	Zimbabwe	2009	140.7918808	0.418330613
198	Zimbabwe	2010	138.7818008	0.417305357
198	Zimbabwe	2011	135.8979089	0.419729695
198	Zimbabwe	2012	133.3447895	0.42574852
198	Zimbabwe	2013	131.240285	0.432881888
198	Zimbabwe	2014	130.8673417	0.439997226
198	Zimbabwe	2015	130.6857896	0.446552872
198	Zimbabwe	2016	130.3613538	0.452473037
198	Zimbabwe	2017	128.7073402	0.458424984
198	Zimbabwe	2018	128.7021848	0.464379262
198	Zimbabwe	2019	127.8830894	0.468384438
198	Zimbabwe	2020	126.9404942	0.471573171
198	Zimbabwe	2021	118.5399648	0.473819486
200	Benin	1990	73.3385432	0.218907154
200	Benin	1991	72.82283833	0.222539019

200	Benin	1992	72.30722135	0.226323465
200	Benin	1993	71.879958	0.230312594
200	Benin	1994	72.11170815	0.234032036
200	Benin	1995	72.3933627	0.238009696
200	Benin	1996	73.11458531	0.241895323
200	Benin	1997	73.35904755	0.245731534
200	Benin	1998	73.85551136	0.249376451
200	Benin	1999	74.16274172	0.253215341
200	Benin	2000	74.53251227	0.257380681
200	Benin	2001	72.75093856	0.261388011
200	Benin	2002	71.70728829	0.265417458
200	Benin	2003	69.70789031	0.269544281
200	Benin	2004	68.34209826	0.273941145
200	Benin	2005	67.49077923	0.27840675
200	Benin	2006	67.56803889	0.282933936
200	Benin	2007	67.25368754	0.287673055
200	Benin	2008	68.30118467	0.292627446
200	Benin	2009	69.78198648	0.297702227
200	Benin	2010	71.28478094	0.302809008
200	Benin	2011	72.90610751	0.307925538
200	Benin	2012	75.06224436	0.313373588
200	Benin	2013	74.65301547	0.319318688
200	Benin	2014	75.39036116	0.325727272
200	Benin	2015	80.20705716	0.33221635
200	Benin	2016	79.10439521	0.338763921
200	Benin	2017	78.35715701	0.345606759
200	Benin	2018	77.67704908	0.352652696
200	Benin	2019	77.90456205	0.360031163
200	Benin	2020	78.18773936	0.366964487
200	Benin	2021	77.64556118	0.373486574
201	Burkina Faso	1990	84.03122307	0.129695615
201	Burkina Faso	1991	82.78114483	0.133299128
201	Burkina Faso	1992	82.57959321	0.136594798
201	Burkina Faso	1993	82.76111406	0.139825465
201	Burkina Faso	1994	82.98980023	0.142848973
201	Burkina Faso	1995	83.32417317	0.146115673
201	Burkina Faso	1996	83.64824891	0.150041686
201	Burkina Faso	1997	84.60328068	0.154228401
201	Burkina Faso	1998	84.97319457	0.158707048
201	Burkina Faso	1999	87.77301735	0.163468067
201	Burkina Faso	2000	90.63422062	0.16808434
201	Burkina Faso	2001	92.74680518	0.172996395
201	Burkina Faso	2002	97.11813663	0.17798199
201	Burkina Faso	2003	95.54071613	0.183253539
201	Burkina Faso	2004	95.81062193	0.188564995
201	Burkina Faso	2005	96.29131363	0.194086128
201	Burkina Faso	2006	98.20632294	0.199571952
201	Burkina Faso	2007	97.96714974	0.204891092
201	Burkina Faso	2008	99.1437529	0.210223295
201	Burkina Faso	2009	100.1466553	0.215423374

201	Burkina Faso	2010	99.92332796	0.22100103
201	Burkina Faso	2011	99.81399888	0.226696632
201	Burkina Faso	2012	101.0533797	0.232589135
201	Burkina Faso	2013	98.41882573	0.238497491
201	Burkina Faso	2014	97.76410167	0.244378901
201	Burkina Faso	2015	103.637198	0.250208261
201	Burkina Faso	2016	102.6200353	0.256081774
201	Burkina Faso	2017	101.1494568	0.262033009
201	Burkina Faso	2018	99.78864807	0.268102073
201	Burkina Faso	2019	98.76546108	0.274287092
201	Burkina Faso	2020	97.55738846	0.279871317
201	Burkina Faso	2021	97.57319675	0.285118402
202	Cameroon	1990	79.8769201	0.303055334
202	Cameroon	1991	79.81792311	0.309623458
202	Cameroon	1992	81.01907392	0.315601269
202	Cameroon	1993	82.85681655	0.320831654
202	Cameroon	1994	84.91877953	0.325410123
202	Cameroon	1995	88.14594978	0.329539318
202	Cameroon	1996	92.90221702	0.333425982
202	Cameroon	1997	100.0906496	0.337346001
202	Cameroon	1998	107.4309449	0.34119709
202	Cameroon	1999	114.3176759	0.344955364
202	Cameroon	2000	122.2625002	0.348814789
202	Cameroon	2001	125.6739886	0.352631873
202	Cameroon	2002	130.1200348	0.356511817
202	Cameroon	2003	131.6824721	0.360668581
202	Cameroon	2004	130.8435771	0.36510003
202	Cameroon	2005	130.4625802	0.369545756
202	Cameroon	2006	130.7151834	0.374355878
202	Cameroon	2007	129.4149872	0.379490349
202	Cameroon	2008	128.4189794	0.384838304
202	Cameroon	2009	127.1332966	0.390288643
202	Cameroon	2010	125.1549281	0.396067145
202	Cameroon	2011	123.548702	0.402092799
202	Cameroon	2012	123.2116216	0.408580123
202	Cameroon	2013	118.9791037	0.415607965
202	Cameroon	2014	116.2755737	0.42303356
202	Cameroon	2015	119.9645874	0.430830276
202	Cameroon	2016	114.5758705	0.438878945
202	Cameroon	2017	110.8804628	0.4471851
202	Cameroon	2018	109.0029995	0.455687847
202	Cameroon	2019	107.1629832	0.464246473
202	Cameroon	2020	105.0925921	0.472285846
202	Cameroon	2021	103.2924139	0.479691223
203	Cabo Verde	1990	71.83135576	0.276723367
203	Cabo Verde	1991	73.11731563	0.282931722
203	Cabo Verde	1992	74.54004084	0.288460371
203	Cabo Verde	1993	77.45904104	0.294517286
203	Cabo Verde	1994	80.22259585	0.302216794
203	Cabo Verde	1995	83.13631959	0.310675634

203	Cabo Verde	1996	88.43042671	0.319736229
203	Cabo Verde	1997	94.78087938	0.329049585
203	Cabo Verde	1998	96.87079251	0.338786069
203	Cabo Verde	1999	98.625591	0.349296918
203	Cabo Verde	2000	99.04900639	0.359899017
203	Cabo Verde	2001	98.90473165	0.370070276
203	Cabo Verde	2002	98.14165673	0.379846674
203	Cabo Verde	2003	97.24516459	0.389419786
203	Cabo Verde	2004	97.0146512	0.398838732
203	Cabo Verde	2005	96.95512755	0.408009221
203	Cabo Verde	2006	90.20323589	0.417433786
203	Cabo Verde	2007	87.41260529	0.427379787
203	Cabo Verde	2008	85.85512408	0.43737213
203	Cabo Verde	2009	84.72311974	0.446691506
203	Cabo Verde	2010	83.72577956	0.455647568
203	Cabo Verde	2011	90.15533664	0.464473969
203	Cabo Verde	2012	92.71600617	0.472857771
203	Cabo Verde	2013	89.53882854	0.480796481
203	Cabo Verde	2014	89.77228112	0.488259896
203	Cabo Verde	2015	97.31877435	0.495229489
203	Cabo Verde	2016	100.1487045	0.501855424
203	Cabo Verde	2017	103.4608594	0.508435792
203	Cabo Verde	2018	126.0787638	0.515285886
203	Cabo Verde	2019	124.7730526	0.522488008
203	Cabo Verde	2020	123.5850801	0.528461595
203	Cabo Verde	2021	121.5054155	0.533534539
204	Chad	1990	87.87417398	0.114638829
204	Chad	1991	87.42576821	0.117954634
204	Chad	1992	88.54248941	0.121132832
204	Chad	1993	90.31563335	0.123600072
204	Chad	1994	91.75258699	0.126080572
204	Chad	1995	93.343967	0.128289492
204	Chad	1996	96.87428759	0.130169484
204	Chad	1997	100.2497511	0.132136021
204	Chad	1998	103.3662588	0.134090304
204	Chad	1999	106.1625255	0.135825261
204	Chad	2000	108.28324	0.137412374
204	Chad	2001	108.8985041	0.139663692
204	Chad	2002	109.8676539	0.1420417
204	Chad	2003	110.248628	0.145348052
204	Chad	2004	109.3465028	0.150910868
204	Chad	2005	107.9041361	0.157033036
204	Chad	2006	107.1513095	0.162576402
204	Chad	2007	106.7975379	0.168161062
204	Chad	2008	107.3621798	0.173396784
204	Chad	2009	106.6493514	0.178335361
204	Chad	2010	106.6596899	0.184110621
204	Chad	2011	107.1351473	0.189591609
204	Chad	2012	107.2269643	0.195262749
204	Chad	2013	104.1800981	0.200952884

204	Chad	2014	102.156552	0.206658836
204	Chad	2015	106.7804402	0.212428697
204	Chad	2016	104.7206751	0.21753218
204	Chad	2017	104.621431	0.22220695
204	Chad	2018	104.5547636	0.226913881
204	Chad	2019	104.2632732	0.231704755
204	Chad	2020	104.460449	0.236103083
204	Chad	2021	104.2866521	0.240436019
205	Côte d'Ivoire	1990	118.0864443	0.279320353
205	Côte d'Ivoire	1991	117.5489762	0.286492681
205	Côte d'Ivoire	1992	119.172035	0.293451293
205	Côte d'Ivoire	1993	121.9163284	0.299743215
205	Côte d'Ivoire	1994	124.6552357	0.305210653
205	Côte d'Ivoire	1995	127.5225582	0.310105508
205	Côte d'Ivoire	1996	129.5874392	0.314965385
205	Côte d'Ivoire	1997	131.6742522	0.319843979
205	Côte d'Ivoire	1998	134.9068651	0.324712873
205	Côte d'Ivoire	1999	138.3879633	0.329201009
205	Côte d'Ivoire	2000	142.2817396	0.333015393
205	Côte d'Ivoire	2001	142.2747369	0.336545511
205	Côte d'Ivoire	2002	142.5641005	0.339977638
205	Côte d'Ivoire	2003	140.9348839	0.343348932
205	Côte d'Ivoire	2004	140.8313364	0.346375411
205	Côte d'Ivoire	2005	139.66734	0.348727913
205	Côte d'Ivoire	2006	138.6345924	0.350715944
205	Côte d'Ivoire	2007	134.9095825	0.352727478
205	Côte d'Ivoire	2008	132.2503717	0.355087643
205	Côte d'Ivoire	2009	129.6928799	0.357938755
205	Côte d'Ivoire	2010	127.9844609	0.361205187
205	Côte d'Ivoire	2011	129.361055	0.36408365
205	Côte d'Ivoire	2012	129.2137055	0.367831685
205	Côte d'Ivoire	2013	126.2005881	0.37235379
205	Côte d'Ivoire	2014	124.6087801	0.377575444
205	Côte d'Ivoire	2015	131.2429776	0.38349138
205	Côte d'Ivoire	2016	128.6942047	0.389825192
205	Côte d'Ivoire	2017	124.925923	0.396693974
205	Côte d'Ivoire	2018	123.9081395	0.404091933
205	Côte d'Ivoire	2019	121.5645985	0.41172558
205	Côte d'Ivoire	2020	120.6340261	0.419040103
205	Côte d'Ivoire	2021	119.9011055	0.425941883
206	Gambia	1990	112.5749671	0.238714846
206	Gambia	1991	113.5814296	0.245010164
206	Gambia	1992	115.6042281	0.251087863
206	Gambia	1993	115.5654437	0.256996141
206	Gambia	1994	116.1130328	0.262424792
206	Gambia	1995	121.5113753	0.267696507
206	Gambia	1996	120.9504672	0.27295019
206	Gambia	1997	122.8023021	0.278274122
206	Gambia	1998	127.6335428	0.283946056
206	Gambia	1999	132.6452551	0.290070621

206	Gambia	2000	131.9765129	0.296354282
206	Gambia	2001	130.3988325	0.302543257
206	Gambia	2002	131.1871714	0.307712927
206	Gambia	2003	132.4070014	0.312930916
206	Gambia	2004	136.5540844	0.318615029
206	Gambia	2005	137.8010394	0.32356835
206	Gambia	2006	137.102965	0.328151431
206	Gambia	2007	137.033311	0.332640147
206	Gambia	2008	134.8177443	0.337315245
206	Gambia	2009	135.0329877	0.342389614
206	Gambia	2010	132.9260891	0.34799783
206	Gambia	2011	127.996611	0.352869236
206	Gambia	2012	130.2533255	0.35837365
206	Gambia	2013	127.5588116	0.364255878
206	Gambia	2014	132.9457288	0.3699013
206	Gambia	2015	135.5484347	0.375840485
206	Gambia	2016	136.2935197	0.381569217
206	Gambia	2017	142.081893	0.387273644
206	Gambia	2018	139.4998384	0.393155679
206	Gambia	2019	141.1979505	0.39918978
206	Gambia	2020	139.7514869	0.404524194
206	Gambia	2021	140.0220444	0.40971416
207	Ghana	1990	142.8002998	0.373112005
207	Ghana	1991	141.9122997	0.379239256
207	Ghana	1992	141.773891	0.384856767
207	Ghana	1993	140.2035258	0.389968494
207	Ghana	1994	140.5128343	0.394953663
207	Ghana	1995	141.4881808	0.399930204
207	Ghana	1996	143.329337	0.405003047
207	Ghana	1997	147.5432632	0.410038073
207	Ghana	1998	149.1139279	0.414888532
207	Ghana	1999	152.4486314	0.419755496
207	Ghana	2000	156.5973436	0.424725212
207	Ghana	2001	150.5290089	0.429411846
207	Ghana	2002	142.3651193	0.434104128
207	Ghana	2003	131.6755521	0.439118213
207	Ghana	2004	121.6531265	0.444236977
207	Ghana	2005	113.0192256	0.44936002
207	Ghana	2006	105.9270138	0.454692737
207	Ghana	2007	98.0328429	0.460606245
207	Ghana	2008	99.67529262	0.467259067
207	Ghana	2009	99.8182701	0.473848047
207	Ghana	2010	99.83962072	0.480777892
207	Ghana	2011	100.8688515	0.489020974
207	Ghana	2012	100.1770753	0.497513932
207	Ghana	2013	96.62761742	0.506004647
207	Ghana	2014	95.19701246	0.514119061
207	Ghana	2015	100.6694444	0.521539202
207	Ghana	2016	99.6445641	0.528629586
207	Ghana	2017	97.71635101	0.53616994

207	Ghana	2018	97.42777372	0.543840539
207	Ghana	2019	97.17250731	0.551632027
207	Ghana	2020	97.26773543	0.558462173
207	Ghana	2021	97.55908071	0.56493039
208	Guinea	1990	92.09659401	0.178295421
208	Guinea	1991	91.68869035	0.181459391
208	Guinea	1992	91.78852604	0.184485016
208	Guinea	1993	91.94283227	0.187686242
208	Guinea	1994	92.96469137	0.191128837
208	Guinea	1995	93.56194117	0.194573245
208	Guinea	1996	94.6219774	0.198633755
208	Guinea	1997	96.77752579	0.203280449
208	Guinea	1998	99.55844102	0.208003383
208	Guinea	1999	102.0008153	0.212412705
208	Guinea	2000	103.3244277	0.216649792
208	Guinea	2001	103.9410577	0.221090941
208	Guinea	2002	105.0651567	0.225898779
208	Guinea	2003	104.7949048	0.230522594
208	Guinea	2004	105.6407539	0.23513497
208	Guinea	2005	106.7273538	0.239691845
208	Guinea	2006	109.1693279	0.244010127
208	Guinea	2007	110.9354155	0.24847169
208	Guinea	2008	112.8687109	0.253167689
208	Guinea	2009	114.4911416	0.257552785
208	Guinea	2010	115.4143215	0.262405949
208	Guinea	2011	116.1041975	0.2676246
208	Guinea	2012	116.5652033	0.273428319
208	Guinea	2013	113.2599599	0.279600444
208	Guinea	2014	114.4504248	0.285961264
208	Guinea	2015	118.3511274	0.292363614
208	Guinea	2016	116.3746552	0.299112412
208	Guinea	2017	115.1907646	0.306881462
208	Guinea	2018	113.6667511	0.314839588
208	Guinea	2019	112.5411771	0.322839848
208	Guinea	2020	111.509484	0.329877997
208	Guinea	2021	110.4052036	0.336401293
209	Guinea-Bissau	1990	150.5850073	0.207614839
209	Guinea-Bissau	1991	150.5304096	0.212505984
209	Guinea-Bissau	1992	150.807928	0.217026088
209	Guinea-Bissau	1993	151.3937474	0.221678978
209	Guinea-Bissau	1994	152.2146319	0.226434933
209	Guinea-Bissau	1995	153.6052606	0.231223526
209	Guinea-Bissau	1996	155.7356688	0.236106374
209	Guinea-Bissau	1997	157.8066194	0.241360584
209	Guinea-Bissau	1998	160.5184945	0.244371797
209	Guinea-Bissau	1999	162.8909516	0.248217317
209	Guinea-Bissau	2000	164.0715609	0.252026519
209	Guinea-Bissau	2001	163.143973	0.255786768
209	Guinea-Bissau	2002	162.638444	0.259321816
209	Guinea-Bissau	2003	161.737993	0.262747724

209	Guinea-Bissau	2004	163.062188	0.266271736
209	Guinea-Bissau	2005	162.1430496	0.270056132
209	Guinea-Bissau	2006	162.396775	0.273803987
209	Guinea-Bissau	2007	162.3230139	0.277805017
209	Guinea-Bissau	2008	162.551999	0.282151502
209	Guinea-Bissau	2009	163.5068739	0.286423345
209	Guinea-Bissau	2010	163.5346602	0.291141639
209	Guinea-Bissau	2011	164.1646874	0.296682967
209	Guinea-Bissau	2012	165.6150235	0.301562342
209	Guinea-Bissau	2013	159.3017202	0.306602009
209	Guinea-Bissau	2014	160.7217966	0.311602905
209	Guinea-Bissau	2015	165.8201865	0.317090015
209	Guinea-Bissau	2016	162.7283373	0.32297967
209	Guinea-Bissau	2017	164.388223	0.329326492
209	Guinea-Bissau	2018	161.9418458	0.335667833
209	Guinea-Bissau	2019	161.349917	0.342156115
209	Guinea-Bissau	2020	158.7395826	0.347798998
209	Guinea-Bissau	2021	156.9172876	0.353109621
210	Liberia	1990	107.5459322	0.235296847
210	Liberia	1991	108.2669881	0.238212218
210	Liberia	1992	107.6043152	0.238283313
210	Liberia	1993	107.1289457	0.235984406
210	Liberia	1994	108.5255985	0.232656774
210	Liberia	1995	109.6404232	0.228424478
210	Liberia	1996	113.7508601	0.222682591
210	Liberia	1997	115.1679066	0.219672765
210	Liberia	1998	117.8593745	0.219053855
210	Liberia	1999	119.5490603	0.222212867
210	Liberia	2000	121.8195607	0.231173362
210	Liberia	2001	119.9019976	0.239594386
210	Liberia	2002	119.8760429	0.248311971
210	Liberia	2003	117.8677572	0.251595458
210	Liberia	2004	117.343907	0.25517961
210	Liberia	2005	115.8586181	0.25896799
210	Liberia	2006	116.139881	0.262897258
210	Liberia	2007	114.596475	0.267239568
210	Liberia	2008	115.263424	0.271853596
210	Liberia	2009	117.3311193	0.276804111
210	Liberia	2010	118.7873441	0.282235752
210	Liberia	2011	122.0916694	0.288015145
210	Liberia	2012	120.9931913	0.294854315
210	Liberia	2013	118.0438272	0.302985656
210	Liberia	2014	116.322744	0.31095544
210	Liberia	2015	120.8627726	0.318485166
210	Liberia	2016	118.176923	0.325085174
210	Liberia	2017	113.3518723	0.331527786
210	Liberia	2018	113.2740953	0.337784578
210	Liberia	2019	113.6574031	0.343403262
210	Liberia	2020	113.9221163	0.34813389
210	Liberia	2021	114.2465693	0.352442452

211	Mali	1990	75.18716185	0.126526428
211	Mali	1991	73.70758906	0.12912815
211	Mali	1992	73.16374175	0.131537449
211	Mali	1993	72.56091207	0.134177054
211	Mali	1994	72.7806388	0.136937829
211	Mali	1995	73.39812752	0.139866225
211	Mali	1996	73.35220205	0.143078916
211	Mali	1997	72.9713852	0.146414326
211	Mali	1998	72.31516857	0.14998673
211	Mali	1999	71.91230442	0.153737336
211	Mali	2000	72.28353753	0.157190028
211	Mali	2001	71.10796706	0.161344448
211	Mali	2002	70.86770401	0.165420696
211	Mali	2003	69.61492837	0.169733502
211	Mali	2004	68.74295477	0.173779552
211	Mali	2005	68.07528911	0.178029844
211	Mali	2006	68.30798752	0.182452468
211	Mali	2007	68.53628393	0.186912995
211	Mali	2008	70.083309	0.191536746
211	Mali	2009	70.69581496	0.196537651
211	Mali	2010	71.62042917	0.201803536
211	Mali	2011	70.90652353	0.207097221
211	Mali	2012	72.95766966	0.21246506
211	Mali	2013	70.30999713	0.217993867
211	Mali	2014	71.46305753	0.22372998
211	Mali	2015	75.45688121	0.229826263
211	Mali	2016	73.10614427	0.236186231
211	Mali	2017	73.41740951	0.242726135
211	Mali	2018	72.96328307	0.249419731
211	Mali	2019	72.87867429	0.256250746
211	Mali	2020	72.40012051	0.262629219
211	Mali	2021	71.65063772	0.268579941
212	Mauritania	1990	136.7169149	0.335780942
212	Mauritania	1991	135.1846324	0.341135175
212	Mauritania	1992	134.7804228	0.346531288
212	Mauritania	1993	133.1366777	0.352176572
212	Mauritania	1994	131.9192771	0.357585912
212	Mauritania	1995	130.693527	0.363037695
212	Mauritania	1996	130.3466696	0.368508328
212	Mauritania	1997	128.6447035	0.373021339
212	Mauritania	1998	126.0856758	0.37708025
212	Mauritania	1999	123.3184196	0.381112297
212	Mauritania	2000	122.4481818	0.38420538
212	Mauritania	2001	120.414814	0.386487416
212	Mauritania	2002	117.0474408	0.388564478
212	Mauritania	2003	111.9032906	0.391104879
212	Mauritania	2004	110.9524442	0.394012934
212	Mauritania	2005	109.0566831	0.397499105
212	Mauritania	2006	107.7111479	0.403159467
212	Mauritania	2007	105.1968351	0.408377122

212	Mauritania	2008	102.9533654	0.413233621
212	Mauritania	2009	103.9892366	0.417910609
212	Mauritania	2010	103.446336	0.423202436
212	Mauritania	2011	103.1405975	0.42886729
212	Mauritania	2012	105.6649014	0.434724376
212	Mauritania	2013	101.413089	0.44106891
212	Mauritania	2014	100.5608202	0.447653881
212	Mauritania	2015	105.484079	0.454523488
212	Mauritania	2016	102.6352201	0.461269545
212	Mauritania	2017	105.8919705	0.468452365
212	Mauritania	2018	105.4737773	0.475839507
212	Mauritania	2019	108.5714551	0.483767738
212	Mauritania	2020	109.9497323	0.491365603
212	Mauritania	2021	112.2262123	0.4989451
213	Niger	1990	68.9134412	0.08086848
213	Niger	1991	67.50203579	0.082848208
213	Niger	1992	67.16004499	0.084771578
213	Niger	1993	66.81907357	0.08669649
213	Niger	1994	66.30611398	0.088608733
213	Niger	1995	66.79038484	0.090473708
213	Niger	1996	66.29969684	0.092346359
213	Niger	1997	67.17290904	0.094219115
213	Niger	1998	67.66274638	0.096545153
213	Niger	1999	68.73180597	0.098813686
213	Niger	2000	70.00755949	0.100886948
213	Niger	2001	70.54126312	0.103271777
213	Niger	2002	70.26180666	0.105741109
213	Niger	2003	70.04365009	0.108322891
213	Niger	2004	68.77636386	0.110764495
213	Niger	2005	68.90777661	0.113452023
213	Niger	2006	68.03011616	0.116247074
213	Niger	2007	67.68928307	0.118971626
213	Niger	2008	66.38807335	0.121957786
213	Niger	2009	67.15393531	0.124829381
213	Niger	2010	67.24747622	0.127952996
213	Niger	2011	69.06338155	0.130958067
213	Niger	2012	69.29281413	0.134469718
213	Niger	2013	67.52046459	0.13797817
213	Niger	2014	67.58201554	0.141731738
213	Niger	2015	74.56241671	0.145440503
213	Niger	2016	73.87339171	0.149200109
213	Niger	2017	71.82047992	0.15295917
213	Niger	2018	72.24339699	0.156875196
213	Niger	2019	72.71974068	0.160889484
213	Niger	2020	72.52647495	0.164539265
213	Niger	2021	72.55734814	0.168072774
214	Nigeria	1990	107.4501106	0.305868047
214	Nigeria	1991	105.4536892	0.308489225
214	Nigeria	1992	104.1362732	0.311234052
214	Nigeria	1993	102.97675	0.31398306

214	Nigeria	1994	102.0052888	0.316747447
214	Nigeria	1995	101.8915096	0.319637439
214	Nigeria	1996	104.5595665	0.323137685
214	Nigeria	1997	106.9210224	0.326690553
214	Nigeria	1998	109.4301956	0.330263851
214	Nigeria	1999	111.9168739	0.3339365
214	Nigeria	2000	114.3496157	0.338201743
214	Nigeria	2001	114.3412099	0.343029751
214	Nigeria	2002	114.0619114	0.349092528
214	Nigeria	2003	111.3793441	0.355911392
214	Nigeria	2004	108.9416823	0.363780814
214	Nigeria	2005	106.2091842	0.372583553
214	Nigeria	2006	105.1196061	0.38102604
214	Nigeria	2007	103.4801468	0.389779649
214	Nigeria	2008	104.6688858	0.398037706
214	Nigeria	2009	106.1091126	0.406353307
214	Nigeria	2010	107.2179208	0.414997748
214	Nigeria	2011	108.7654812	0.423351915
214	Nigeria	2012	109.9434321	0.431780376
214	Nigeria	2013	108.2416281	0.440124664
214	Nigeria	2014	108.0319326	0.448766823
214	Nigeria	2015	114.0961189	0.457240991
214	Nigeria	2016	111.1937238	0.465183781
214	Nigeria	2017	110.3268067	0.472972869
214	Nigeria	2018	110.2851249	0.480844413
214	Nigeria	2019	109.9465711	0.488787361
214	Nigeria	2020	109.3067835	0.496204741
214	Nigeria	2021	109.4781024	0.503390833
215	Sao Tome and Principe	1990	80.50550439	0.309542852
215	Sao Tome and Principe	1991	81.33019491	0.310601028
215	Sao Tome and Principe	1992	82.46266407	0.311515447
215	Sao Tome and Principe	1993	80.9083974	0.312743558
215	Sao Tome and Principe	1994	81.94425825	0.314261087
215	Sao Tome and Principe	1995	82.66652999	0.31584392
215	Sao Tome and Principe	1996	85.69156168	0.317729288
215	Sao Tome and Principe	1997	88.40094825	0.31979567
215	Sao Tome and Principe	1998	91.21256825	0.322519219
215	Sao Tome and Principe	1999	92.83903269	0.325651916
215	Sao Tome and Principe	2000	94.28135218	0.329460109
215	Sao Tome and Principe	2001	94.97920017	0.334484786
215	Sao Tome and Principe	2002	96.29510352	0.340302837
215	Sao Tome and Principe	2003	96.89039568	0.346851981
215	Sao Tome and Principe	2004	99.01572477	0.353722384
215	Sao Tome and Principe	2005	99.0621627	0.361105881
215	Sao Tome and Principe	2006	100.8043802	0.369413007
215	Sao Tome and Principe	2007	100.5949394	0.377813517
215	Sao Tome and Principe	2008	100.3057006	0.386688944
215	Sao Tome and Principe	2009	101.2781861	0.395821238
215	Sao Tome and Principe	2010	102.1848839	0.405530666
215	Sao Tome and Principe	2011	103.1640562	0.415783728

215	Sao Tome and Principe	2012	103.3355931	0.425721315
215	Sao Tome and Principe	2013	104.4766487	0.43559246
215	Sao Tome and Principe	2014	105.1996507	0.445146237
215	Sao Tome and Principe	2015	109.392464	0.454051473
215	Sao Tome and Principe	2016	108.955955	0.463230082
215	Sao Tome and Principe	2017	105.6883527	0.47217093
215	Sao Tome and Principe	2018	106.5985335	0.481098204
215	Sao Tome and Principe	2019	106.0773875	0.48983823
215	Sao Tome and Principe	2020	105.1755078	0.497861409
215	Sao Tome and Principe	2021	103.540362	0.505413747
216	Senegal	1990	128.2218261	0.238047613
216	Senegal	1991	129.1552037	0.244482178
216	Senegal	1992	130.6911969	0.250416532
216	Senegal	1993	132.0852028	0.255767964
216	Senegal	1994	133.7518243	0.260379997
216	Senegal	1995	133.0135663	0.264438773
216	Senegal	1996	133.5770028	0.268318965
216	Senegal	1997	132.9509303	0.272072875
216	Senegal	1998	132.9568236	0.275995656
216	Senegal	1999	133.1537652	0.280111918
216	Senegal	2000	130.600039	0.283784903
216	Senegal	2001	138.3241059	0.287451899
216	Senegal	2002	136.340882	0.291046288
216	Senegal	2003	131.3059003	0.294887172
216	Senegal	2004	130.8204581	0.298984521
216	Senegal	2005	128.677108	0.303257209
216	Senegal	2006	127.0174094	0.30763391
216	Senegal	2007	126.6820345	0.312570298
216	Senegal	2008	127.5065784	0.317894089
216	Senegal	2009	127.9863632	0.323657726
216	Senegal	2010	125.5075865	0.329804072
216	Senegal	2011	124.7119189	0.336059897
216	Senegal	2012	125.0193162	0.342977114
216	Senegal	2013	119.36414	0.350134004
216	Senegal	2014	119.7370609	0.357181573
216	Senegal	2015	121.0322035	0.364144496
216	Senegal	2016	118.3192493	0.371114525
216	Senegal	2017	119.8771467	0.378351477
216	Senegal	2018	118.6870514	0.386079666
216	Senegal	2019	121.052617	0.394012316
216	Senegal	2020	121.2317903	0.401197516
216	Senegal	2021	121.617104	0.408054193
217	Sierra Leone	1990	128.7941666	0.211569335
217	Sierra Leone	1991	125.9299196	0.213013567
217	Sierra Leone	1992	124.8578099	0.21422304
217	Sierra Leone	1993	124.0444027	0.216089201
217	Sierra Leone	1994	123.2384767	0.218277411
217	Sierra Leone	1995	124.5473676	0.219822604
217	Sierra Leone	1996	126.522103	0.21996927
217	Sierra Leone	1997	130.0329962	0.219089791

217	Sierra Leone	1998	132.4253182	0.218584313
217	Sierra Leone	1999	134.3400902	0.217943042
217	Sierra Leone	2000	137.1820921	0.218492837
217	Sierra Leone	2001	139.9022352	0.220391233
217	Sierra Leone	2002	143.7526179	0.224585825
217	Sierra Leone	2003	144.9908038	0.2292359
217	Sierra Leone	2004	145.3091995	0.234131674
217	Sierra Leone	2005	145.7643508	0.23954994
217	Sierra Leone	2006	145.6873609	0.245647886
217	Sierra Leone	2007	144.3379838	0.252523077
217	Sierra Leone	2008	142.5343703	0.259781562
217	Sierra Leone	2009	141.1115225	0.26705838
217	Sierra Leone	2010	140.8101388	0.274270275
217	Sierra Leone	2011	142.3350522	0.281673575
217	Sierra Leone	2012	140.3357494	0.290338512
217	Sierra Leone	2013	135.4376443	0.300736693
217	Sierra Leone	2014	134.4168039	0.310348867
217	Sierra Leone	2015	138.5288118	0.31699865
217	Sierra Leone	2016	135.7660798	0.324091565
217	Sierra Leone	2017	132.8797461	0.331213431
217	Sierra Leone	2018	131.9014564	0.338341564
217	Sierra Leone	2019	131.3139845	0.345770019
217	Sierra Leone	2020	129.9673568	0.352412022
217	Sierra Leone	2021	129.5334094	0.358665881
218	Togo	1990	114.2375093	0.269692273
218	Togo	1991	114.8543557	0.274530568
218	Togo	1992	116.2240145	0.27878931
218	Togo	1993	118.4181633	0.281224631
218	Togo	1994	120.5572726	0.284653387
218	Togo	1995	122.2468178	0.288536692
218	Togo	1996	125.2428473	0.292606677
218	Togo	1997	129.1724377	0.296947835
218	Togo	1998	132.9905608	0.300022333
218	Togo	1999	133.8898006	0.303020232
218	Togo	2000	135.6634078	0.305472963
218	Togo	2001	135.1642552	0.307828258
218	Togo	2002	134.4138564	0.31046991
218	Togo	2003	133.0710907	0.313792064
218	Togo	2004	129.6114035	0.316687944
218	Togo	2005	128.3586043	0.31902558
218	Togo	2006	128.7016118	0.32165939
218	Togo	2007	127.9916068	0.324382497
218	Togo	2008	129.2297438	0.327743136
218	Togo	2009	127.8870591	0.331558542
218	Togo	2010	124.9722543	0.335899583
218	Togo	2011	122.9287554	0.34077726
218	Togo	2012	121.0076184	0.346320495
218	Togo	2013	116.5269665	0.352278605
218	Togo	2014	114.9578911	0.358661442
218	Togo	2015	120.0699975	0.365600169

218	Togo	2016	117.9331314	0.372910028
218	Togo	2017	117.002777	0.380336231
218	Togo	2018	116.3462031	0.387921229
218	Togo	2019	115.9001212	0.395667269
218	Togo	2020	115.993665	0.402356922
218	Togo	2021	117.0039835	0.408533695
298	American Samoa	1990	166.5022298	0.613633924
298	American Samoa	1991	166.315097	0.615847612
298	American Samoa	1992	166.4970122	0.618583654
298	American Samoa	1993	166.4122971	0.622078598
298	American Samoa	1994	165.8334479	0.626053241
298	American Samoa	1995	165.0708062	0.629814054
298	American Samoa	1996	164.3760202	0.632706983
298	American Samoa	1997	163.2700442	0.634926685
298	American Samoa	1998	164.2496956	0.636597976
298	American Samoa	1999	167.2762287	0.638322193
298	American Samoa	2000	169.7657923	0.64060955
298	American Samoa	2001	172.7571859	0.643214421
298	American Samoa	2002	172.8454994	0.646002062
298	American Samoa	2003	171.4259525	0.648951964
298	American Samoa	2004	168.9334274	0.651921141
298	American Samoa	2005	168.8764188	0.655061005
298	American Samoa	2006	170.0278489	0.657725723
298	American Samoa	2007	171.2621209	0.660781176
298	American Samoa	2008	173.2414343	0.664025762
298	American Samoa	2009	176.3125434	0.667300532
298	American Samoa	2010	176.5105328	0.671248916
298	American Samoa	2011	175.7424541	0.675979288
298	American Samoa	2012	175.9145687	0.680861539
298	American Samoa	2013	177.2922935	0.685833061
298	American Samoa	2014	178.6531872	0.691343137
298	American Samoa	2015	180.3109661	0.697162912
298	American Samoa	2016	181.8573797	0.702726398
298	American Samoa	2017	182.0880351	0.707647909
298	American Samoa	2018	181.0484765	0.712800591
298	American Samoa	2019	180.3654006	0.717308133
298	American Samoa	2020	189.2606789	0.7208385
298	American Samoa	2021	178.991894	0.723727533
305	Bermuda	1990	245.2010502	0.696451196
305	Bermuda	1991	233.0524363	0.700276851
305	Bermuda	1992	216.871527	0.703984095
305	Bermuda	1993	194.9709143	0.707678222
305	Bermuda	1994	189.5179267	0.711068998
305	Bermuda	1995	181.259579	0.714468828
305	Bermuda	1996	171.0058066	0.717831671
305	Bermuda	1997	153.9236937	0.721642813
305	Bermuda	1998	145.6586851	0.725853528
305	Bermuda	1999	141.3734553	0.730539823
305	Bermuda	2000	132.4721473	0.735996502
305	Bermuda	2001	123.8581266	0.741918908

305	Bermuda	2002	110.7560788	0.747544503
305	Bermuda	2003	105.4261168	0.753305163
305	Bermuda	2004	106.3516393	0.759008885
305	Bermuda	2005	103.0106318	0.764690903
305	Bermuda	2006	103.1253624	0.77059677
305	Bermuda	2007	98.11160286	0.776114264
305	Bermuda	2008	88.99472649	0.780358325
305	Bermuda	2009	82.27055156	0.784759107
305	Bermuda	2010	79.88184818	0.789181467
305	Bermuda	2011	82.3651807	0.793380524
305	Bermuda	2012	82.34846945	0.79748487
305	Bermuda	2013	82.36471252	0.801388201
305	Bermuda	2014	79.09413581	0.804923407
305	Bermuda	2015	76.92798758	0.807998795
305	Bermuda	2016	74.55558195	0.810550842
305	Bermuda	2017	70.47172222	0.812807229
305	Bermuda	2018	69.23564485	0.814892802
305	Bermuda	2019	68.80955618	0.817000338
305	Bermuda	2020	65.38347503	0.81920342
305	Bermuda	2021	68.34745779	0.821365422
320	Cook Islands	1990	160.2665577	0.564514854
320	Cook Islands	1991	157.4630117	0.570622776
320	Cook Islands	1992	154.3391287	0.577123468
320	Cook Islands	1993	150.8229655	0.584059175
320	Cook Islands	1994	148.4172833	0.591540868
320	Cook Islands	1995	146.3286003	0.598789968
320	Cook Islands	1996	144.525727	0.606414151
320	Cook Islands	1997	142.8709055	0.613991564
320	Cook Islands	1998	141.6188146	0.621202228
320	Cook Islands	1999	139.9722973	0.62802903
320	Cook Islands	2000	137.7302387	0.635365607
320	Cook Islands	2001	135.3992332	0.644421442
320	Cook Islands	2002	133.02862	0.652790488
320	Cook Islands	2003	128.4022032	0.661465038
320	Cook Islands	2004	124.8671945	0.668769675
320	Cook Islands	2005	121.7162134	0.674004103
320	Cook Islands	2006	119.1372369	0.680644001
320	Cook Islands	2007	117.8389712	0.687505191
320	Cook Islands	2008	116.0269522	0.695592069
320	Cook Islands	2009	115.1575476	0.70377698
320	Cook Islands	2010	115.5240773	0.711861612
320	Cook Islands	2011	115.8447332	0.719444666
320	Cook Islands	2012	116.7092759	0.726963385
320	Cook Islands	2013	117.6352843	0.733317015
320	Cook Islands	2014	117.1265739	0.739595704
320	Cook Islands	2015	116.5879147	0.746550297
320	Cook Islands	2016	115.6346077	0.752353087
320	Cook Islands	2017	113.7889828	0.758233572
320	Cook Islands	2018	111.9977917	0.764041182
320	Cook Islands	2019	110.3008611	0.769795913

320	Cook Islands	2020	108.5356734	0.774700578
320	Cook Islands	2021	107.1540132	0.779109955
349	Greenland	1990	210.1866416	0.732258252
349	Greenland	1991	207.0359059	0.730626532
349	Greenland	1992	201.7827409	0.729781103
349	Greenland	1993	198.1332112	0.729894842
349	Greenland	1994	194.8135454	0.730875896
349	Greenland	1995	190.6230719	0.730831638
349	Greenland	1996	181.4671541	0.730216225
349	Greenland	1997	172.7522789	0.730551087
349	Greenland	1998	163.8347733	0.733242588
349	Greenland	1999	158.013923	0.73655713
349	Greenland	2000	148.963077	0.74085701
349	Greenland	2001	141.7248379	0.746289605
349	Greenland	2002	137.7702344	0.752878371
349	Greenland	2003	131.8765774	0.759444152
349	Greenland	2004	127.4142871	0.765401567
349	Greenland	2005	123.6448841	0.77209958
349	Greenland	2006	118.9605106	0.779141406
349	Greenland	2007	114.5035344	0.785321922
349	Greenland	2008	111.6309275	0.791441257
349	Greenland	2009	107.2373467	0.79702575
349	Greenland	2010	103.849447	0.803003131
349	Greenland	2011	100.1536922	0.808105185
349	Greenland	2012	99.35321852	0.811684562
349	Greenland	2013	97.59265774	0.814368308
349	Greenland	2014	95.16429279	0.81652706
349	Greenland	2015	93.46046349	0.817719189
349	Greenland	2016	91.61102737	0.818961902
349	Greenland	2017	90.78880862	0.819781101
349	Greenland	2018	89.4439401	0.820815514
349	Greenland	2019	88.28355564	0.822339996
349	Greenland	2020	89.81040914	0.824359028
349	Greenland	2021	81.19910153	0.826210336
351	Guam	1990	203.5458882	0.676220305
351	Guam	1991	199.5341673	0.669974292
351	Guam	1992	183.9395498	0.66635634
351	Guam	1993	165.0475245	0.667318181
351	Guam	1994	151.8023498	0.672111712
351	Guam	1995	147.779506	0.678120215
351	Guam	1996	147.6025102	0.684173735
351	Guam	1997	147.7503418	0.691684871
351	Guam	1998	148.5991976	0.701819289
351	Guam	1999	149.3418634	0.713641593
351	Guam	2000	151.8122952	0.724802357
351	Guam	2001	150.0846291	0.734267376
351	Guam	2002	150.5055543	0.741284245
351	Guam	2003	144.7265926	0.74591153
351	Guam	2004	139.8723241	0.74901652
351	Guam	2005	137.2658951	0.750865875

351	Guam	2006	133.2310444	0.751176245
351	Guam	2007	131.2354714	0.752625471
351	Guam	2008	133.7998728	0.755152408
351	Guam	2009	137.0671443	0.758252296
351	Guam	2010	135.7734465	0.761484317
351	Guam	2011	130.9275555	0.763987394
351	Guam	2012	132.7892354	0.766845984
351	Guam	2013	142.3265074	0.770224957
351	Guam	2014	152.6921934	0.773441684
351	Guam	2015	149.2940146	0.776723614
351	Guam	2016	145.9622649	0.780511644
351	Guam	2017	150.513836	0.78555492
351	Guam	2018	154.9543763	0.791294773
351	Guam	2019	153.0421286	0.796775452
351	Guam	2020	125.2738776	0.800969472
351	Guam	2021	126.6007495	0.803982203
367	Monaco	1990	103.5151965	0.845495153
367	Monaco	1991	101.1524175	0.84830369
367	Monaco	1992	98.97955006	0.85086394
367	Monaco	1993	97.25801491	0.853264992
367	Monaco	1994	95.88801777	0.855626927
367	Monaco	1995	94.20482267	0.857986147
367	Monaco	1996	92.48846172	0.860245432
367	Monaco	1997	90.79421468	0.86244672
367	Monaco	1998	88.20524659	0.864716513
367	Monaco	1999	84.92820965	0.866842394
367	Monaco	2000	81.10928294	0.868973583
367	Monaco	2001	77.17110753	0.871094755
367	Monaco	2002	74.43117956	0.873182769
367	Monaco	2003	72.47195744	0.875197652
367	Monaco	2004	70.86969049	0.877231549
367	Monaco	2005	69.69919263	0.879288089
367	Monaco	2006	68.23413571	0.881274148
367	Monaco	2007	66.29640273	0.883262291
367	Monaco	2008	64.43649716	0.885331394
367	Monaco	2009	63.48382644	0.887328279
367	Monaco	2010	62.84810982	0.889262841
367	Monaco	2011	62.10083615	0.891237592
367	Monaco	2012	60.08630954	0.893073822
367	Monaco	2013	58.63537693	0.894864472
367	Monaco	2014	57.06482565	0.896547874
367	Monaco	2015	55.99367323	0.898356337
367	Monaco	2016	54.21882237	0.900051303
367	Monaco	2017	53.14359082	0.901752712
367	Monaco	2018	52.07613941	0.903388687
367	Monaco	2019	50.85540077	0.905030991
367	Monaco	2020	49.90394643	0.906685437
367	Monaco	2021	49.33314312	0.908262831
369	Nauru	1990	412.2541505	0.539145981
369	Nauru	1991	417.9395984	0.538433557

369	Nauru	1992	424.4758304	0.537038893
369	Nauru	1993	428.7708025	0.535059742
369	Nauru	1994	436.3900394	0.533001097
369	Nauru	1995	448.0073942	0.530890925
369	Nauru	1996	459.6833941	0.528544474
369	Nauru	1997	471.5603953	0.52624159
369	Nauru	1998	480.7234665	0.523885302
369	Nauru	1999	488.4867992	0.521894324
369	Nauru	2000	493.5556469	0.520254888
369	Nauru	2001	501.6418647	0.519235425
369	Nauru	2002	506.690433	0.518438705
369	Nauru	2003	510.1770782	0.518459986
369	Nauru	2004	512.4916542	0.51880954
369	Nauru	2005	513.4035563	0.519892863
369	Nauru	2006	512.782712	0.522673179
369	Nauru	2007	513.1205559	0.52320135
369	Nauru	2008	512.213553	0.526084096
369	Nauru	2009	509.6175345	0.530221092
369	Nauru	2010	505.2247904	0.535689995
369	Nauru	2011	499.7577339	0.542342743
369	Nauru	2012	494.021282	0.549890621
369	Nauru	2013	485.5829105	0.560112906
369	Nauru	2014	475.5974275	0.571424364
369	Nauru	2015	467.2219749	0.581283234
369	Nauru	2016	459.2116392	0.590102988
369	Nauru	2017	451.9449763	0.597619076
369	Nauru	2018	445.3954305	0.605311879
369	Nauru	2019	439.7739079	0.612697253
369	Nauru	2020	436.3453422	0.619330076
369	Nauru	2021	432.6438475	0.625177834
374	Niue	1990	241.2750519	0.587532984
374	Niue	1991	244.2994971	0.593882057
374	Niue	1992	249.1803836	0.599881243
374	Niue	1993	250.4539325	0.605514779
374	Niue	1994	251.5057865	0.610650175
374	Niue	1995	253.6815653	0.615374609
374	Niue	1996	255.6590949	0.619734555
374	Niue	1997	257.7156129	0.623913848
374	Niue	1998	256.2002368	0.627578934
374	Niue	1999	255.5182183	0.630857172
374	Niue	2000	254.5145731	0.63406895
374	Niue	2001	253.3983918	0.637366627
374	Niue	2002	254.7702309	0.641122021
374	Niue	2003	255.4950651	0.64577954
374	Niue	2004	256.1192714	0.650614832
374	Niue	2005	254.5017947	0.657281265
374	Niue	2006	255.2172174	0.664162983
374	Niue	2007	254.8751262	0.670199299
374	Niue	2008	255.3039155	0.676396859
374	Niue	2009	252.3288852	0.682579173

374	Niue	2010	249.8901457	0.68628063
374	Niue	2011	249.3265633	0.690315578
374	Niue	2012	248.6539749	0.694150949
374	Niue	2013	247.4118137	0.697221078
374	Niue	2014	246.435387	0.700545924
374	Niue	2015	246.0388119	0.704232979
374	Niue	2016	245.7910267	0.707476099
374	Niue	2017	244.4849562	0.711220996
374	Niue	2018	243.2044941	0.71510823
374	Niue	2019	242.3595641	0.719078885
374	Niue	2020	244.018154	0.72279025
374	Niue	2021	243.5136394	0.72622205
376	Northern Mariana Islands	1990	134.0776884	0.708593838
376	Northern Mariana Islands	1991	132.3578782	0.712223642
376	Northern Mariana Islands	1992	130.4311442	0.715787607
376	Northern Mariana Islands	1993	128.8258837	0.719179396
376	Northern Mariana Islands	1994	127.8031786	0.722510494
376	Northern Mariana Islands	1995	127.1436924	0.726219053
376	Northern Mariana Islands	1996	126.565468	0.730074086
376	Northern Mariana Islands	1997	126.0816289	0.733795366
376	Northern Mariana Islands	1998	125.5280892	0.737423127
376	Northern Mariana Islands	1999	126.7183359	0.740934815
376	Northern Mariana Islands	2000	127.142445	0.744492865
376	Northern Mariana Islands	2001	127.2262803	0.747170606
376	Northern Mariana Islands	2002	128.0506063	0.748755189
376	Northern Mariana Islands	2003	128.2443264	0.750171533
376	Northern Mariana Islands	2004	128.2312125	0.751528841
376	Northern Mariana Islands	2005	128.9936706	0.751826692
376	Northern Mariana Islands	2006	129.5086336	0.751797809
376	Northern Mariana Islands	2007	132.084451	0.751608416
376	Northern Mariana Islands	2008	136.3061404	0.750807192
376	Northern Mariana Islands	2009	141.8267992	0.748597173
376	Northern Mariana Islands	2010	143.3798322	0.746617262
376	Northern Mariana Islands	2011	145.4826948	0.744411908
376	Northern Mariana Islands	2012	147.6932412	0.742937351
376	Northern Mariana Islands	2013	150.7248734	0.74228436
376	Northern Mariana Islands	2014	153.6249789	0.742605449
376	Northern Mariana Islands	2015	156.0634994	0.743797398
376	Northern Mariana Islands	2016	155.2492998	0.748455466
376	Northern Mariana Islands	2017	153.4436609	0.756495479
376	Northern Mariana Islands	2018	152.6924409	0.7612554
376	Northern Mariana Islands	2019	151.7034122	0.765770647
376	Northern Mariana Islands	2020	132.8681982	0.76942766
376	Northern Mariana Islands	2021	152.0909593	0.771535213
380	Palau	1990	249.8738893	0.66290951
380	Palau	1991	249.1803208	0.668230731
380	Palau	1992	248.4994635	0.673302462
380	Palau	1993	248.7132981	0.676648715
380	Palau	1994	241.5242545	0.680195775
380	Palau	1995	240.1628516	0.68483214

380	Palau	1996	240.937778	0.690095673
380	Palau	1997	242.0133597	0.695122123
380	Palau	1998	243.1899121	0.69965291
380	Palau	1999	243.9512432	0.703115322
380	Palau	2000	242.2885589	0.705196945
380	Palau	2001	241.5338705	0.707402386
380	Palau	2002	240.3541802	0.709587644
380	Palau	2003	238.808536	0.71100022
380	Palau	2004	240.0853588	0.712543811
380	Palau	2005	241.7300375	0.714339831
380	Palau	2006	242.9872144	0.716353431
380	Palau	2007	244.6403089	0.718809542
380	Palau	2008	246.3844068	0.720806623
380	Palau	2009	248.0452461	0.722288267
380	Palau	2010	249.707589	0.724065956
380	Palau	2011	252.9303586	0.726733446
380	Palau	2012	254.4684043	0.729612899
380	Palau	2013	257.4913552	0.731953475
380	Palau	2014	253.8401219	0.734768297
380	Palau	2015	251.2113008	0.738166425
380	Palau	2016	248.2494457	0.741361911
380	Palau	2017	246.0257556	0.744224767
380	Palau	2018	239.5293504	0.74776125
380	Palau	2019	235.9205637	0.750715307
380	Palau	2020	233.4698184	0.752780929
380	Palau	2021	233.6220501	0.754046931
385	Puerto Rico	1990	161.5588103	0.658758146
385	Puerto Rico	1991	150.9091882	0.66326172
385	Puerto Rico	1992	144.287472	0.667272192
385	Puerto Rico	1993	139.1431262	0.671024105
385	Puerto Rico	1994	132.1120314	0.67545081
385	Puerto Rico	1995	129.7469275	0.68067395
385	Puerto Rico	1996	126.9482211	0.685865893
385	Puerto Rico	1997	118.1457411	0.691274382
385	Puerto Rico	1998	119.8172599	0.697040445
385	Puerto Rico	1999	109.0783507	0.702850671
385	Puerto Rico	2000	102.3437786	0.710035652
385	Puerto Rico	2001	102.2391698	0.718955861
385	Puerto Rico	2002	92.98236771	0.725956856
385	Puerto Rico	2003	91.08552547	0.730475138
385	Puerto Rico	2004	89.20654277	0.734219546
385	Puerto Rico	2005	87.19966103	0.737742266
385	Puerto Rico	2006	80.98310024	0.741629431
385	Puerto Rico	2007	75.51131662	0.745883807
385	Puerto Rico	2008	71.07033111	0.750922167
385	Puerto Rico	2009	69.66211689	0.756361695
385	Puerto Rico	2010	62.68811182	0.761763253
385	Puerto Rico	2011	63.84499461	0.766784882
385	Puerto Rico	2012	57.29110743	0.772311804
385	Puerto Rico	2013	57.76841946	0.778461162

385	Puerto Rico	2014	55.83115228	0.785120345
385	Puerto Rico	2015	53.90744249	0.791800167
385	Puerto Rico	2016	55.28908524	0.799084083
385	Puerto Rico	2017	57.81409597	0.806648961
385	Puerto Rico	2018	53.23319494	0.812906066
385	Puerto Rico	2019	53.91827593	0.818843191
385	Puerto Rico	2020	53.01921122	0.822918436
385	Puerto Rico	2021	53.61732105	0.825525847
393	Saint Kitts and Nevis	1990	220.6161303	0.580685877
393	Saint Kitts and Nevis	1991	180.4058905	0.59025495
393	Saint Kitts and Nevis	1992	172.0969077	0.59918719
393	Saint Kitts and Nevis	1993	164.9626905	0.607235892
393	Saint Kitts and Nevis	1994	169.8355662	0.614514658
393	Saint Kitts and Nevis	1995	186.6190075	0.620716498
393	Saint Kitts and Nevis	1996	181.1203423	0.626207411
393	Saint Kitts and Nevis	1997	173.2370698	0.63127474
393	Saint Kitts and Nevis	1998	165.6202983	0.635539988
393	Saint Kitts and Nevis	1999	160.1896104	0.639713739
393	Saint Kitts and Nevis	2000	155.372693	0.644353377
393	Saint Kitts and Nevis	2001	148.4848726	0.649226653
393	Saint Kitts and Nevis	2002	144.4875108	0.654593409
393	Saint Kitts and Nevis	2003	136.1289899	0.659925312
393	Saint Kitts and Nevis	2004	131.104957	0.66756813
393	Saint Kitts and Nevis	2005	129.5212023	0.679145457
393	Saint Kitts and Nevis	2006	119.9155147	0.690078667
393	Saint Kitts and Nevis	2007	111.45411	0.697727628
393	Saint Kitts and Nevis	2008	108.5750445	0.704141494
393	Saint Kitts and Nevis	2009	105.863511	0.709499498
393	Saint Kitts and Nevis	2010	106.0116717	0.714460415
393	Saint Kitts and Nevis	2011	107.4340274	0.719324381
393	Saint Kitts and Nevis	2012	104.5159836	0.723436152
393	Saint Kitts and Nevis	2013	100.6545049	0.726706593
393	Saint Kitts and Nevis	2014	98.94153476	0.729530219
393	Saint Kitts and Nevis	2015	96.16987666	0.732220672
393	Saint Kitts and Nevis	2016	92.97211243	0.735566138
393	Saint Kitts and Nevis	2017	91.72624785	0.739470681
393	Saint Kitts and Nevis	2018	90.96377134	0.743736461
393	Saint Kitts and Nevis	2019	92.68088463	0.748152066
393	Saint Kitts and Nevis	2020	91.66860068	0.751568409
393	Saint Kitts and Nevis	2021	91.15046291	0.754987055
396	San Marino	1990	76.91902357	0.813244888
396	San Marino	1991	75.60848242	0.818282513
396	San Marino	1992	74.44835802	0.822782758
396	San Marino	1993	72.9604435	0.827382324
396	San Marino	1994	71.95519363	0.831970983
396	San Marino	1995	70.04244123	0.836762726
396	San Marino	1996	67.79557517	0.841389495
396	San Marino	1997	65.15365474	0.845864865
396	San Marino	1998	62.84623658	0.850474771
396	San Marino	1999	60.01366363	0.855338613

396	San Marino	2000	57.27193557	0.859695363
396	San Marino	2001	54.56611855	0.863747757
396	San Marino	2002	51.86295707	0.865680592
396	San Marino	2003	49.60863742	0.867522009
396	San Marino	2004	44.27705649	0.869287134
396	San Marino	2005	42.69178864	0.870979596
396	San Marino	2006	41.31328449	0.87265215
396	San Marino	2007	39.90775697	0.874307253
396	San Marino	2008	38.55501798	0.875991881
396	San Marino	2009	37.58730365	0.877705228
396	San Marino	2010	36.93403073	0.879440814
396	San Marino	2011	36.50294918	0.881197518
396	San Marino	2012	36.17032241	0.882924531
396	San Marino	2013	35.79887027	0.884592627
396	San Marino	2014	36.39820365	0.885353694
396	San Marino	2015	36.84649285	0.884693365
396	San Marino	2016	37.23807704	0.884477665
396	San Marino	2017	37.71111631	0.884557607
396	San Marino	2018	37.56401422	0.885133516
396	San Marino	2019	37.37988269	0.88620066
396	San Marino	2020	24.42120866	0.886867937
396	San Marino	2021	23.18765002	0.888005474
413	Tokelau	1990	225.7612599	0.521942386
413	Tokelau	1991	226.3955789	0.526628473
413	Tokelau	1992	226.8861199	0.53130785
413	Tokelau	1993	227.3770126	0.535935005
413	Tokelau	1994	227.8505757	0.540523665
413	Tokelau	1995	228.7354214	0.545295634
413	Tokelau	1996	229.3197113	0.550282652
413	Tokelau	1997	229.5184255	0.555631326
413	Tokelau	1998	229.5160554	0.561047611
413	Tokelau	1999	229.199669	0.566415406
413	Tokelau	2000	228.8286375	0.57181437
413	Tokelau	2001	228.6118566	0.576915597
413	Tokelau	2002	229.1195944	0.581858317
413	Tokelau	2003	229.498475	0.586854346
413	Tokelau	2004	229.2947883	0.592342442
413	Tokelau	2005	228.0526467	0.598176117
413	Tokelau	2006	227.0707125	0.60442939
413	Tokelau	2007	226.2188428	0.611163237
413	Tokelau	2008	225.1175648	0.617940247
413	Tokelau	2009	224.1500672	0.624070078
413	Tokelau	2010	223.2271406	0.63017554
413	Tokelau	2011	222.0531677	0.636016518
413	Tokelau	2012	221.0776786	0.641611991
413	Tokelau	2013	219.221493	0.647966148
413	Tokelau	2014	217.6074088	0.653231999
413	Tokelau	2015	215.6753724	0.658798995
413	Tokelau	2016	213.5218607	0.663974319
413	Tokelau	2017	211.5131481	0.669054134

413	Tokelau	2018	209.3704614	0.674179514
413	Tokelau	2019	207.4276278	0.679247839
413	Tokelau	2020	207.3342831	0.682937378
413	Tokelau	2021	205.4059403	0.686425621
416	Tuvalu	1990	274.8897438	0.406247566
416	Tuvalu	1991	274.459003	0.414819937
416	Tuvalu	1992	274.1900661	0.42290198
416	Tuvalu	1993	274.3493064	0.43051826
416	Tuvalu	1994	273.1403811	0.438563051
416	Tuvalu	1995	275.4805981	0.444962242
416	Tuvalu	1996	277.3545835	0.44980498
416	Tuvalu	1997	281.1429971	0.455368428
416	Tuvalu	1998	281.2915691	0.462454678
416	Tuvalu	1999	281.9579681	0.468845802
416	Tuvalu	2000	281.880556	0.474956823
416	Tuvalu	2001	280.4180928	0.480850886
416	Tuvalu	2002	280.0472006	0.487252252
416	Tuvalu	2003	279.672385	0.492754711
416	Tuvalu	2004	279.2788953	0.497692152
416	Tuvalu	2005	277.5326432	0.50185131
416	Tuvalu	2006	278.2700107	0.505975411
416	Tuvalu	2007	279.2675712	0.510504301
416	Tuvalu	2008	280.2013516	0.515611601
416	Tuvalu	2009	281.2369063	0.519870566
416	Tuvalu	2010	282.2954502	0.52345551
416	Tuvalu	2011	282.8654626	0.527744245
416	Tuvalu	2012	282.8497461	0.531203235
416	Tuvalu	2013	282.3488877	0.535166235
416	Tuvalu	2014	282.5353134	0.539122244
416	Tuvalu	2015	281.7720371	0.54404788
416	Tuvalu	2016	280.9281465	0.549173928
416	Tuvalu	2017	278.4379162	0.55446648
416	Tuvalu	2018	276.1323929	0.559859395
416	Tuvalu	2019	273.830594	0.566045489
416	Tuvalu	2020	271.721	0.571666102
416	Tuvalu	2021	269.53603	0.576620529
422	United States Virgin Islands	1990	213.3528443	0.655160856
422	United States Virgin Islands	1991	207.1217504	0.664018659
422	United States Virgin Islands	1992	204.2856128	0.671081871
422	United States Virgin Islands	1993	202.4557487	0.676872745
422	United States Virgin Islands	1994	202.2044613	0.681982223
422	United States Virgin Islands	1995	192.5141683	0.686730211
422	United States Virgin Islands	1996	190.9991025	0.691247699
422	United States Virgin Islands	1997	186.0175683	0.695598204
422	United States Virgin Islands	1998	181.9905471	0.699768686
422	United States Virgin Islands	1999	181.456853	0.703949342
422	United States Virgin Islands	2000	182.5252788	0.708436565
422	United States Virgin Islands	2001	180.1688956	0.713751115
422	United States Virgin Islands	2002	174.4561639	0.724398272
422	United States Virgin Islands	2003	171.5156394	0.73460985

422	United States Virgin Islands	2004	168.6297375	0.744613598
422	United States Virgin Islands	2005	163.0479862	0.754267558
422	United States Virgin Islands	2006	152.4000699	0.763447496
422	United States Virgin Islands	2007	146.7492777	0.772180593
422	United States Virgin Islands	2008	138.3810754	0.780342623
422	United States Virgin Islands	2009	134.3051757	0.787055146
422	United States Virgin Islands	2010	133.2385582	0.793219696
422	United States Virgin Islands	2011	131.4674858	0.798071698
422	United States Virgin Islands	2012	130.9331273	0.801092851
422	United States Virgin Islands	2013	128.8827211	0.803405789
422	United States Virgin Islands	2014	126.8924711	0.805641378
422	United States Virgin Islands	2015	124.3919028	0.807889245
422	United States Virgin Islands	2016	122.5576136	0.810199044
422	United States Virgin Islands	2017	121.786278	0.812405172
422	United States Virgin Islands	2018	121.6777525	0.81482544
422	United States Virgin Islands	2019	122.8008114	0.817429008
422	United States Virgin Islands	2020	104.1128486	0.819601712
422	United States Virgin Islands	2021	102.6217483	0.821830853
435	South Sudan	1990	72.36636531	0.2066565
435	South Sudan	1991	72.44371221	0.208932716
435	South Sudan	1992	72.81147937	0.211199401
435	South Sudan	1993	73.13746446	0.213416471
435	South Sudan	1994	73.37616453	0.215612089
435	South Sudan	1995	74.25725423	0.217920463
435	South Sudan	1996	74.52540438	0.220399437
435	South Sudan	1997	74.15049387	0.223037171
435	South Sudan	1998	74.91945041	0.225788869
435	South Sudan	1999	75.09924697	0.228648585
435	South Sudan	2000	75.29143223	0.231730344
435	South Sudan	2001	73.90422385	0.235014128
435	South Sudan	2002	73.65848414	0.238341186
435	South Sudan	2003	73.98653534	0.241797191
435	South Sudan	2004	74.45831027	0.245462499
435	South Sudan	2005	74.36624295	0.249280904
435	South Sudan	2006	74.58180376	0.253300508
435	South Sudan	2007	74.36461535	0.257491764
435	South Sudan	2008	74.32869727	0.261740458
435	South Sudan	2009	74.38508433	0.266179235
435	South Sudan	2010	73.93793943	0.270572622
435	South Sudan	2011	73.88811126	0.274719143
435	South Sudan	2012	74.66546203	0.275066143
435	South Sudan	2013	75.16391027	0.275813942
435	South Sudan	2014	74.74922951	0.276311461
435	South Sudan	2015	77.11256898	0.275833634
435	South Sudan	2016	77.60923056	0.274455491
435	South Sudan	2017	78.63862537	0.274387859
435	South Sudan	2018	79.68439108	0.274987848
435	South Sudan	2019	80.36888039	0.275706201
435	South Sudan	2020	80.81164124	0.276787924
435	South Sudan	2021	81.21632213	0.278371125

522	Sudan	1990	339.5686036	0.292178643
522	Sudan	1991	336.776559	0.296473446
522	Sudan	1992	334.3675646	0.300863677
522	Sudan	1993	331.5404948	0.305409326
522	Sudan	1994	329.4302007	0.31009643
522	Sudan	1995	326.7587511	0.315208259
522	Sudan	1996	324.3082925	0.320680229
522	Sudan	1997	321.9565013	0.326761462
522	Sudan	1998	319.480958	0.333087761
522	Sudan	1999	316.7966471	0.339754884
522	Sudan	2000	314.5632872	0.346875809
522	Sudan	2001	311.2445896	0.354215515
522	Sudan	2002	306.5025758	0.361728237
522	Sudan	2003	301.7059735	0.369693653
522	Sudan	2004	297.6494324	0.377920656
522	Sudan	2005	292.6278487	0.386685712
522	Sudan	2006	287.6089845	0.396151374
522	Sudan	2007	282.8758616	0.406079992
522	Sudan	2008	279.4088368	0.4160876
522	Sudan	2009	276.2776202	0.425975917
522	Sudan	2010	272.7870274	0.436171097
522	Sudan	2011	269.8523546	0.447239442
522	Sudan	2012	267.3101904	0.456790265
522	Sudan	2013	263.0832786	0.466435995
522	Sudan	2014	257.4261575	0.476362135
522	Sudan	2015	257.192679	0.486549437
522	Sudan	2016	254.9382807	0.496848219
522	Sudan	2017	254.6891646	0.506883534
522	Sudan	2018	254.656046	0.516214086
522	Sudan	2019	255.0997099	0.525009122
522	Sudan	2020	255.2673071	0.533455422
522	Sudan	2021	255.881304	0.541949735

hic Index (SDI): Effective	
**frontier**	**eff_diff**
34.99628081	59.14456588
35.00418672	58.67801883
35.01083508	58.22417737
34.99165399	58.34130301
35.00088401	58.03847798
35.01684287	58.19599073
35.00592129	58.18968067
35.01304897	58.27319867
35.01192923	59.9204828
34.97797212	62.32493322
35.02681422	67.51960132
35.005908	72.11284964
35.03195729	76.73345958
35.06130063	86.02531714
35.04406474	91.72689562
34.98992812	93.05263071
34.96932606	85.86348389
34.99904684	84.8371218
34.98455786	87.44955644
34.96306204	90.59141222
35.01531746	93.59121955
35.02445352	94.71370211
34.97525629	90.85333682
35.00800924	91.48074222
34.99834458	88.9312882
35.00949354	84.64878095
35.00363899	82.55933688
35.01669458	81.10006109
35.00457994	79.27896388
34.99787858	77.83958249
34.96159474	77.0378886
35.03629078	75.87489177
34.98851573	77.65943152
35.02531507	79.0457912
35.04150938	80.53649032
34.98883785	82.19642783
34.98656161	83.74534615
34.9958103	85.27722124
35.00940019	86.85677769
34.99364283	88.56904614
34.98367935	90.38664795
34.98855094	92.09506216
34.97642838	93.84853216
34.98562839	95.36644042
35.02875597	97.67028141
35.00573275	99.70111212

34.99119012	101.0143231
35.03840549	101.4601128
34.97284421	101.1538606
35.0362238	100.5183155
35.01127736	100.334593
35.00845996	100.3513059
34.99545693	100.1366595
35.00429052	102.4191676
34.96023225	102.1622058
35.00281682	102.8006998
35.0201942	100.5370031
34.99249945	97.65427109
35.01226231	93.65746675
35.03539822	92.21262284
35.04850546	92.31920418
35.01226231	91.80255838
34.992343	91.09497143
34.99553744	90.35068625
34.99420951	38.72689497
35.02608965	38.60077226
34.98166449	39.28165544
35.01092901	35.41600709
34.98806241	31.32089848
34.99211251	28.25963569
34.9931895	27.17001975
34.99637789	22.28698924
35.0389093	19.84732764
34.98594858	16.41088404
35.02019185	13.86041319
35.0482443	13.17839984
35.00251505	11.7881983
35.00794878	12.41029291
34.99451446	12.94024897
35.0359796	11.89099235
34.97654236	8.713757819
34.9993912	7.333912315
34.81821441	6.983914898
34.84725238	4.352528364
34.21459865	4.175123425
33.34410257	6.388046178
31.63247487	7.578065349
30.46028414	8.362010468
30.23138697	9.794390867
30.57079994	8.437114102
29.53005764	10.73684058
27.64071269	10.7146486
27.30278796	9.57445771
26.31593858	9.167691317
25.29306317	7.963136597
25.53447691	8.071519604

34.97378582	80.76071092
34.99403953	79.41043978
34.99097442	78.62934974
34.98938405	78.50190199
34.9973658	78.69627233
35.02727044	78.69469273
35.00177003	78.06856924
35.00022946	77.42353962
34.97722789	77.01019178
35.01746316	76.53273241
35.00564288	75.81309948
34.97768506	74.85582492
34.98327842	74.01063252
35.03692808	72.89247739
34.99950832	72.14976094
34.99625538	71.30706162
35.03743452	70.42033258
35.02122858	70.82580373
35.00595192	70.77050872
34.9981568	70.93105986
35.0328904	70.62692279
35.02724399	69.66796344
34.99463992	70.54663371
34.99938342	73.26505928
35.03494982	72.59680411
35.02949211	73.3463808
35.00173513	74.52890885
34.98644215	74.12452684
34.99056522	74.88106106
35.0116559	75.16471594
35.01136494	75.8154015
35.00392357	76.54278245
35.03633493	67.92187831
35.01047613	69.95854542
34.99308781	71.85278317
35.03718858	73.69374482
35.03631429	75.00969891
34.98836597	75.94933734
34.99972678	77.02035941
35.01418495	78.48314721
35.0193182	79.76141472
35.01331816	81.33369038
35.02156362	83.10759215
35.00287191	84.61384458
35.0137182	87.05611488
35.03271625	89.45954744
35.04718337	92.01284433
34.98831939	94.37845244
34.99161266	96.53466502
35.10442556	97.60602481

34.99796378	100.2317554
35.02114202	102.3766529
34.97683643	104.0845066
34.98751206	103.2628441
34.97819608	105.801091
34.99573684	106.8807175
34.98556142	107.386459
34.97435802	109.3433625
35.0459613	109.6738231
34.98987098	108.0174918
35.00294703	108.2172501
35.02277684	108.3471965
35.00819128	108.5250461
35.00890681	108.2459166
35.02842974	200.3153671
35.017126	197.7781509
35.02049868	195.400478
34.99023692	192.8589532
34.9711322	189.7998702
34.9916722	186.6802784
35.02366755	184.0327564
35.00747585	181.7479554
35.0371268	179.1258804
34.99953425	176.0418298
34.98166449	172.1464692
34.98443772	170.4452824
34.98170311	165.5625925
35.00527591	162.292208
34.98649255	159.2715666
34.972597	156.7904017
35.05415336	154.4505492
34.99770334	151.9224379
35.01285035	149.9970058
34.99046045	147.8794412
34.9718257	144.8434839
34.99782811	139.5530058
35.00263226	138.9861466
34.99284478	140.8166196
34.97990911	140.0714768
34.99536698	140.1966517
35.03413816	142.0470767
35.01299153	140.227434
35.00735301	141.2167112
35.01951651	141.6799925
35.01034905	141.6614879
35.01045203	141.4516152
35.00978324	142.4439565
34.98956682	137.6319635
35.0082671	136.7105986
34.99984733	137.3945465

34.97817488	136.5529625
35.00371431	142.779018
35.0106558	138.4049055
34.99900516	136.9179353
35.04106345	138.9481149
34.99528428	157.1528833
34.99892565	142.6376129
34.97001546	138.0501167
35.01615516	136.2055032
35.01691851	139.9847533
35.00596653	137.5001785
34.99308531	134.8406047
35.01821408	130.9099413
35.00627756	128.8664633
35.00779663	133.2864165
35.00430679	135.980495
34.97624387	134.7896205
34.96213974	127.5167365
35.04583613	119.9238157
35.0269252	118.0038286
35.0061542	116.9489216
35.01651798	122.6297299
34.99921417	119.2497946
34.97533554	118.7408417
35.00736718	116.8134304
34.9928198	115.010431
34.97335555	98.47634173
35.03633493	114.6227826
35.02382631	132.9673995
35.00242619	135.5991282
34.98598195	136.0946171
35.00174659	134.1437797
35.01362864	127.8399925
35.03653129	124.9549694
35.02477877	126.0408701
35.00012458	123.7872043
35.01630411	114.2882072
34.98007696	106.4319753
34.99865372	104.8564088
35.00300782	101.9545835
35.03065983	94.84633306
35.02820038	90.0888418
35.03335065	85.54175835
35.02138994	81.68224649
35.09649796	77.93191487
35.04182009	74.22253094
35.00865639	71.37133382
35.01057596	68.76641095
34.9892657	65.82596924
35.03519227	62.28064992

34.99046643	59.69775729
35.03117434	57.46256401
34.96748681	54.9270957
34.97661953	52.24034375
34.99012857	51.42813727
35.01078508	50.88705432
34.9770904	48.68895455
35.00770992	46.49089701
35.03534592	41.58043991
35.01483294	44.41443348
35.017653	158.3104474
35.02642855	157.4592457
34.97839667	156.2901229
34.97420802	155.1440742
34.99119286	153.4367889
34.97973166	151.7672398
34.9980691	149.9046939
34.9823915	147.9776569
34.98805909	146.1970886
35.02721201	144.3104226
34.9641868	142.8795931
34.9964355	141.4056414
35.0034353	139.6548445
35.00392838	138.7954907
35.0049996	138.4222939
35.00252394	136.6268046
34.99498992	135.613505
35.01615705	133.0621508
34.96421492	130.8196858
35.04622255	128.3008586
34.99192396	124.4386491
35.0044096	117.52975
35.03754297	114.8052679
35.0194044	113.0297905
35.01060205	108.648719
35.00735877	106.4811821
34.9896182	105.5775222
35.05832954	102.7268893
34.98283532	102.2324737
34.96213974	101.9533339
35.0196269	102.403343
34.97595177	103.2335607
34.98475308	139.1587563
35.03444715	125.8585754
34.99260335	123.3990392
35.00351468	122.3819024
34.98313048	123.8827779
35.03708913	125.2501942
35.04370829	127.9324594
34.98649255	125.5537418

35.01580784	125.8053958
34.96008883	123.9460236
34.96819881	124.6182299
35.02077767	125.9852357
35.02944054	126.3294899
35.05196278	120.458779
34.98906868	117.0850111
35.0035958	119.3694571
35.02975512	118.3854348
35.01588387	115.0112839
35.00066274	115.3951353
34.98071033	116.3146773
35.00209115	115.1304241
34.97821327	113.3825685
35.00588828	114.121856
34.97455689	113.8813162
35.00160185	112.2526119
34.97175645	114.1367288
35.01487117	116.3268964
34.98595835	113.8834837
35.01857979	114.2575682
34.9820695	116.756164
35.00457994	113.3452098
34.97420802	115.4453622
34.99597917	103.4459687
34.99097442	92.95972579
35.01242465	92.00325198
35.04714367	84.99876897
34.97947444	87.46660728
34.99720111	89.55400621
34.98230336	90.49866205
35.04731867	97.51134545
35.02721373	93.12815358
35.01478451	93.34964873
34.98416985	91.30294369
35.01576033	83.99771493
34.98116117	81.62958453
35.01638481	84.42036245
34.99951027	84.68014194
35.00226564	90.70495904
35.00498761	92.13384879
34.99521121	90.6213483
34.98439346	92.14498742
34.99968183	88.28582591
34.97533554	84.3654787
35.00595192	84.31863357
34.99164431	81.06782526
34.98241679	82.38817913
35.00253617	80.06985975
35.03696768	77.98819382

35.00148323	74.03606523
34.9802521	72.49128292
35.03820836	70.00382033
34.98027573	65.65956684
35.02240408	54.56151371
35.03611249	59.55021067
35.02288061	49.39469341
34.99933229	51.32180275
34.98069426	52.29030018
35.01338934	52.82544262
35.04405517	52.79040251
34.98466197	52.16408304
35.00773443	51.41314732
35.01669725	45.24359942
35.02122802	46.59086995
35.04052605	47.19547702
34.98867356	45.78274607
34.96403289	42.5794039
34.99920624	43.59601112
35.02796716	42.29157049
35.02570112	41.76106933
35.04088455	39.89889142
35.00852237	36.61731611
35.041015	33.84970859
34.98343028	31.02271127
34.96928078	27.43535349
34.99506655	25.5772162
35.03042862	21.11232158
34.99049521	18.14851202
35.01885859	16.18802708
34.99634296	13.69208169
34.96438785	11.92916735
34.99211229	11.91024887
34.99403953	9.802074183
35.00911982	10.14013553
35.03942631	11.12671664
34.97903141	11.52349071
35.00811009	12.04650667
35.01603274	87.78269362
35.00271176	87.54896193
35.02088291	87.21296944
35.00600542	86.8519256
35.02237376	86.20237318
35.06817117	85.17237147
35.00455879	83.89444362
35.0462851	82.73906042
34.99113516	83.0648574
35.0279806	83.88392437
34.992343	84.34920114
35.0311435	84.22856788

35.01879818	83.89475044
34.99151322	85.25514772
35.01480581	85.94127736
34.98450001	87.57491787
35.0532801	90.17011577
34.98116603	93.03527537
35.05755265	96.00641121
34.98504025	101.3087829
35.01373015	104.0745815
35.00225831	109.5788436
35.00115058	114.1306057
35.02301422	117.8554739
35.01524683	120.7894592
34.98447977	119.6355513
35.01623319	120.2915954
34.98140831	120.7371846
34.98975741	121.7861577
35.04690854	123.1928103
34.99968937	122.1408543
35.02649968	121.9218949
35.01686366	31.38177044
35.02987102	30.49835023
34.99084696	29.85377679
35.00990257	29.25793243
35.00499537	28.83467263
35.01104123	28.48260377
34.99288044	28.13359189
34.97623421	27.92251348
35.03282301	27.59739535
35.00934661	27.52297586
35.00922654	27.56533974
35.02237838	28.46485914
35.04455063	29.8076337
35.00917543	31.43367421
35.02273298	33.47939768
35.05055442	35.72478924
34.97828204	38.22609866
35.03723944	40.59906094
35.02607813	42.97864327
35.02484343	45.1927069
34.97540158	46.044444
35.01480498	45.31784228
35.01483294	45.33713905
34.97409132	46.00421885
35.03704683	44.90114678
34.99483702	44.82989287
35.00089489	44.71130855
35.01120753	43.62484959
35.03313739	43.3474683
35.02733588	43.11429863

35.00134704	42.84067547
35.05042812	41.9405246
34.98682485	256.0989909
35.03348388	260.5670611
35.00640271	264.8729648
35.01776786	269.8508869
34.99576125	273.6060513
35.00758305	279.8783874
34.96573672	285.8782768
34.99197887	284.4755974
35.04155253	275.9176488
35.02768477	290.3097479
35.01174341	295.8066046
35.04948949	283.9103726
35.00177003	272.4597904
34.98591272	272.1406399
35.06157493	252.2842669
35.01803048	247.1028296
35.00309094	246.7224036
35.07330837	244.7735273
34.99380637	245.7662388
35.02187615	244.8547102
35.00487454	247.2232476
35.00443256	247.8100282
34.98381636	246.3915283
34.97232235	246.2579217
35.01452098	246.4636353
35.01712236	245.8332059
35.02578984	244.7590204
35.03421346	243.7865778
35.01044073	241.0878311
35.01603591	237.9103628
35.0074794	235.7270496
35.02139362	231.7551207
35.0146045	154.6296802
35.00318977	154.9864675
35.03241052	156.4044593
34.9731517	159.4270337
35.01149865	159.0247544
34.98687995	159.7841159
34.98687995	160.2866176
35.03462342	161.1221333
34.99596582	161.9847208
35.01185882	162.9350203
35.00759153	163.7498595
35.05218218	162.108796
35.05483558	161.7313148
35.01631134	161.0068794
34.97907647	160.7549695
34.99640689	160.4142748

35.04879182	161.5719984
34.97459312	163.0921294
34.99564638	165.1310682
35.07013382	166.8494803
35.01366793	168.4266238
35.01688564	170.023403
34.99475431	170.320518
34.97445409	170.5707214
35.07666081	170.4324117
34.9807701	170.5646154
34.9648217	170.7051541
35.0256901	170.3896388
34.96190916	169.5027784
35.01994336	168.8940401
35.01331816	168.4905803
35.01315331	167.930583
34.9634114	228.7141945
35.01767694	233.2603528
35.00558382	238.0357185
35.01016834	239.1230978
35.0019351	227.7920432
34.99381488	231.3481702
34.98281584	232.6737944
35.03488293	235.6497945
34.98906868	237.6245769
35.02326799	240.2726477
34.98587134	242.6683653
35.05564046	244.5063033
35.02153094	247.0261204
35.02801573	249.918864
35.00472694	253.166072
34.9875216	255.7603725
34.97774211	259.3807872
34.99693706	259.7996441
35.03791132	260.2652915
34.99977685	260.7751474
34.97029263	259.911564
35.00251505	259.1036705
34.9986113	257.5962799
35.01293272	255.7644642
35.00798032	254.3669994
34.97865835	252.3711923
34.96005453	250.0713352
34.99259194	248.0969156
35.00260266	246.0409435
34.97808261	244.6519053
35.00444011	242.8763264
35.00197803	240.8951996
35.00457168	233.0125407
34.99689329	232.8266569

35.0031963	232.3082934
34.97601733	231.7093684
35.01198692	231.4233022
34.97423815	231.0218244
34.97055681	230.6550074
34.98474323	230.3531742
34.98481317	230.000814
34.98728375	229.8341149
34.99255264	229.8801949
35.03889007	229.3988433
35.04439727	230.4834537
34.98648125	229.8110947
34.99973555	229.8275382
34.99839925	229.9780363
35.01879821	230.0924527
35.01694585	231.1928075
35.01603591	232.6114228
34.9858774	233.4623003
34.9946468	234.7030633
34.99475145	234.619664
35.00320764	234.4380984
35.0159302	234.7045988
34.97055681	234.3004332
34.99236627	235.6018826
34.99356781	233.5664936
35.0202969	232.6462673
35.01331816	232.2698573
34.98693063	231.9836627
34.96159474	232.3160568
35.02860181	231.405754
34.99869746	101.5226477
34.99295092	103.1954641
34.98012317	103.3568193
35.0056847	103.4773706
35.07258674	103.1731077
34.97094632	103.601991
35.0191988	105.0214545
34.99521121	105.2429947
35.04498491	106.1749227
35.02031281	107.3785378
34.98202702	109.1391537
35.01303988	110.9188895
35.02401163	112.8439813
35.02098066	113.6513404
35.03393473	114.5055959
35.0128442	116.6897799
34.98742926	119.518344
35.02557718	120.2791074
34.95947531	119.6275674
34.97654964	117.3445947

34.97326161	118.2582278
34.98521267	118.7789359
35.0267953	117.193801
34.99840204	114.7475951
34.99987947	113.2532199
35.03607219	113.2990639
34.96526755	111.9138423
34.99939173	110.9336964
35.01001413	110.3195693
35.00105349	111.1545716
35.02178296	110.763615
34.98970791	109.4579944
35.04002064	170.6866183
35.03998299	170.630593
35.0197347	171.8629289
34.9891743	173.397847
35.04598347	174.86908
35.01488756	176.3410386
34.99689329	178.1360462
34.98930269	180.0254811
35.02715488	181.1992095
35.01150677	182.7139836
35.02085537	183.5547692
35.03749933	183.9033742
35.02492472	184.4304487
35.03003791	185.260279
35.01960831	185.4514143
35.0007098	185.4032317
35.01803048	185.0062794
35.04907596	185.4620833
34.99599049	186.0688763
35.02314463	186.7295333
34.98522914	187.6623981
35.00105014	187.6749994
35.02634815	190.648777
34.99788732	193.2778621
35.0173654	195.2224816
34.96958177	196.7469881
34.97135748	198.439758
35.00404265	197.2449392
35.00909027	195.7686232
34.97453519	194.2216919
34.9648217	192.8331864
35.02178883	191.7024274
34.98320163	257.8340204
35.01347934	260.6663297
35.02238941	259.7537433
35.05048273	259.2793969
35.01769304	257.7921361
34.98728284	253.0227189

35.00500945	250.8818632
34.98077123	250.5844216
35.0499289	247.2348028
34.97140253	242.3674923
34.97217283	241.6591689
35.01984984	237.8083572
34.9842455	235.9288782
34.99452631	232.8706567
34.98258268	229.9180712
35.03599311	229.3809334
35.01861422	231.2962878
35.02951447	227.9716712
34.98650458	225.1203676
35.04651888	230.3101782
35.02189867	228.6408782
34.99837031	237.0064131
35.01524683	239.4263835
34.97865835	241.2218696
35.05419945	243.8166551
35.00302219	243.8126676
35.00452941	240.9862805
35.01245035	241.5967904
35.00030322	241.6671742
35.00794282	243.7564161
34.98077123	243.9641249
35.00951044	240.1579688
35.01162286	103.6785172
35.02648057	102.1847007
34.98389131	99.12514457
35.04420151	99.83471748
34.98817074	100.1444674
35.06330297	96.31359579
34.98735222	96.79013498
35.03634115	100.3571925
34.97169555	108.3233785
35.00101199	115.237666
34.99012857	113.6673275
35.00610528	110.4366974
35.01198692	110.4027428
35.00953079	110.4241261
34.99919091	107.1196796
35.02323462	99.54440971
34.99431718	101.4511628
35.00794282	103.9371804
34.97121909	106.3993259
35.00976755	108.3626826
34.96176505	110.0852201
35.00115058	110.4659806
35.03790326	110.7705691
35.03727865	110.705226

35.04532833	110.2046235
35.01155558	109.6375329
34.98561019	108.6374712
34.99967102	107.3075153
34.992969	106.1881647
35.01976546	105.389652
34.97509535	104.5396993
34.99760172	104.129889
34.98892896	277.3623049
35.03568597	280.7330935
34.99891543	283.1861584
35.04306581	284.4514593
35.0093325	288.2955774
34.99190375	285.8009977
35.0165193	287.5072792
34.9993687	287.8513906
35.01747564	288.0539492
34.96665358	289.0734802
35.02755282	284.3512696
34.99049521	285.907189
35.02216474	279.7902729
34.99590219	273.824036
35.02708056	274.3911262
35.00735573	280.7613529
35.02019185	282.3488219
35.04656265	285.2130479
35.03876137	286.422983
34.99795687	284.1221262
34.97593525	282.2002036
35.04330879	283.1085375
35.01164257	281.1076181
35.03794101	279.6413242
35.00462486	278.0758422
35.01009587	277.3830584
35.00865639	276.8904485
35.01002003	276.203629
35.0110628	275.7811632
35.01480581	275.295846
35.00362829	274.7156487
35.01373528	273.3768256
35.0121394	272.4231664
35.02258009	297.6696876
35.05209504	342.0299483
35.02624792	364.0476012
34.98656504	346.0725181
35.00821137	341.5129195
35.0117453	337.3788665
35.01905986	319.8086525
34.97577532	313.7828565
34.97884967	314.0682733

35.03895057	300.1245548
35.03425079	288.9457354
35.01098977	278.5349815
34.98507199	277.31791
34.9957163	272.4322391
35.00676632	264.7231188
35.0166653	255.7654481
34.9964355	242.1704569
35.03777791	239.9174302
35.06227329	234.8516949
34.99308531	230.8995429
35.01696766	223.3449383
35.01623319	215.1057633
34.99865086	207.1581139
34.99284919	204.5960723
35.00632368	200.3778039
35.05085887	199.0847432
34.99626706	189.8125021
34.98912276	184.4637514
34.97805107	179.4818357
34.98084127	177.9833332
34.98859845	174.7866967
34.96128331	327.344929
35.01260043	335.4339056
34.9984819	357.138729
34.96613639	370.932295
34.99328381	377.7779406
34.97722275	376.4019143
35.02710582	362.9541442
34.96385259	346.8077186
35.00048668	338.3293393
35.03315416	334.3400198
35.01996754	332.9231108
35.04785442	323.4880774
34.98267558	330.3302737
34.97798254	339.4938351
34.97839152	341.1933726
35.03080428	352.1275045
34.99000693	350.0127591
34.98006078	344.3068269
34.97967362	344.785613
34.99318834	342.3672233
35.00170373	342.6361469
35.03834094	340.9555313
35.00175346	346.8657196
34.99976553	347.1341982
35.02243232	349.1763515
34.97240169	343.2163443
35.04097834	342.8172648
35.02326799	341.4209437

34.98771949	312.5856486
35.01513447	299.2777418
34.97092379	287.7050706
34.97343969	271.1612512
35.01358242	323.6314475
35.039541	336.8210195
34.98831939	351.2780487
35.0210099	327.1888584
35.01322822	315.7401743
34.99082794	313.72352
34.96851593	337.7420925
35.04952012	334.9654973
35.00472694	338.9656995
34.96840505	340.8258025
35.04408209	331.5645704
34.97433243	317.5657106
34.99124508	301.7685749
35.00850327	269.0968984
35.02090413	249.4490325
34.99410329	208.1295531
35.01824816	182.47222
35.03564461	173.771353
34.97646593	203.3714222
35.01859849	194.3599239
34.99082794	184.4623778
35.00818953	157.4233275
35.01493052	148.9519249
35.01318534	140.3054175
35.00872904	136.0346577
34.995506	123.32273
35.00560564	111.8490681
35.00187522	105.9848397
35.03082533	97.72963895
34.96784427	92.83589533
35.03108622	97.5483158
34.97901616	89.17646768
34.99743132	263.6542414
34.99915527	287.0360618
34.96958177	318.9730144
35.02897518	365.4471098
34.98747151	397.8072734
35.02162396	429.2484883
35.05917279	427.5904071
35.00521661	413.1597365
35.02380749	401.8515956
34.97753463	394.4285352
35.05779697	397.0972407
35.00096163	395.7275111
35.01859849	399.4353172
34.99651527	401.5390842

35.03361861	364.8561035
35.00364559	362.0216397
35.02899079	359.9461362
35.03585868	361.0501207
35.01278302	334.8203516
35.005043	281.1230776
34.99700475	254.2354595
34.98110873	247.4740636
34.97340086	245.3591412
35.00538562	242.3068056
34.99067749	236.6160504
35.01287984	232.6519449
34.99368851	228.2857668
35.0424171	216.7193091
34.97825378	214.8726929
35.0124102	212.108171
35.02557718	209.8059836
34.97325906	201.0034552
35.0148166	229.3666854
34.99392682	228.3530407
35.01547631	240.0785593
34.98892896	264.8043302
34.97743467	286.6295957
34.99361798	285.8991019
34.99782811	283.6452839
35.01059692	282.0120946
35.00386028	280.241392
35.01766056	271.1025757
35.04685474	280.8292152
34.96484715	278.4686957
34.96324408	291.387306
34.98713643	299.4757999
34.99433286	293.3326095
35.02147523	308.6734825
35.0360687	322.3371233
34.98817074	326.3652818
34.99490197	327.771135
34.9820695	324.4036599
35.01615907	331.1784381
34.98555821	338.0277265
35.01592599	339.4798721
35.04042863	321.9546478
35.04730502	314.1854052
34.97841002	317.2922541
35.03000604	306.2020408
35.00306574	289.5629209
34.99250484	266.7245368
35.03269235	256.1670425
35.02795766	250.4203394
34.99941619	239.4095501

35.02509919	289.7684136
35.0117494	322.3996587
34.97947444	339.325649
35.01698949	331.4148748
34.98125126	299.9450399
34.99750464	325.083483
35.01536556	342.3248584
34.99868817	342.5439513
35.04282668	335.0408439
35.00642566	331.5885591
35.00105014	330.3247601
35.00977696	334.1889861
34.98654582	320.6236571
35.03903894	306.9281825
35.00247786	293.5722983
34.98805095	287.7767629
35.00223346	278.6792209
34.98903308	262.5194255
35.03352927	255.4193889
35.03028636	261.3186189
35.01797181	252.4215491
35.00355027	251.3001589
34.98834166	242.5666093
35.06845014	233.8535604
35.00601423	219.1184984
34.9824896	220.7435217
34.97264855	215.1439773
35.04653386	206.8955551
35.00746332	206.355889
34.99266731	193.7036431
35.03903491	181.7954918
34.99555281	184.5916444
34.96836643	269.8587488
34.99510866	279.6396886
34.96932606	307.0109926
35.01454748	315.1333861
34.96042689	343.8279465
34.96114058	338.3267235
35.03181328	311.2163598
35.03589985	292.2928212
34.99987158	294.5173248
34.99506655	289.9968385
34.99780233	288.2016965
35.02111272	289.5554269
34.96961711	292.6420417
35.00845996	293.0208404
35.05416158	297.5087915
35.03203076	308.7215794
34.97453519	315.8739721
35.00805042	328.132883

35.02309201	327.9327855
35.01215675	323.6715486
34.98987098	302.5918116
35.02160513	295.1609588
35.01098977	277.7457446
35.00756249	251.9174858
35.01686366	246.5116142
35.01544269	239.5045664
35.01621189	232.4320265
35.01243299	224.7635588
35.02191325	219.741568
35.02309001	215.0453027
35.01028333	213.5121505
35.03022061	209.8904029
35.01299192	371.9922181
34.98366913	397.0512405
34.98212163	416.8557656
34.99301572	474.3801346
35.02667654	473.6237967
35.00554212	473.5554841
35.01063405	496.9372502
35.00364049	484.3682772
35.00460668	464.8311668
35.00187796	437.6554304
34.96447112	428.000867
35.02511822	418.1924025
35.04279751	433.9678752
34.96613639	442.2128105
35.01405325	446.8608142
35.02199529	450.0137545
34.969768	434.0303975
34.99951724	418.7731776
34.98650458	364.4163735
35.06661546	311.3945629
34.97604049	292.1294472
35.00852237	290.3807165
35.01653082	288.2042675
35.00289343	293.4329014
35.02306524	301.6094497
35.00157049	306.9361376
35.03910113	303.7562401
35.1206223	310.8312163
34.97565308	311.5243052
35.00439021	313.6774375
34.98140795	311.7246035
34.98592498	308.6905506
34.99405103	283.6264088
35.02207705	296.5673867
35.01173832	309.2031817
35.00233966	337.0597147

34.97794157	347.834785
35.00737019	353.3969308
35.00472316	354.5952557
34.99865372	352.1074074
34.99968183	357.5204323
35.0037735	347.5341753
34.98737009	353.4054647
34.98041164	355.8601947
34.97040337	364.2741495
35.08585064	372.0541823
34.9972885	374.9526322
35.01486439	398.395653
35.0356172	408.3151422
34.9842455	411.440613
35.04449648	410.270103
35.04675011	398.022233
35.00846387	396.8830831
35.00973401	401.1172392
34.99618892	408.3421638
35.02639253	403.5128525
34.98493092	397.2145876
34.98258268	393.021112
34.99175915	376.9251583
34.98066533	360.9934583
34.96804008	340.5368013
34.98127143	314.3664485
35.05734436	309.6201693
35.03259212	304.4459863
35.01281785	139.2217836
34.99640535	136.9409441
34.96311566	125.7242052
34.99878931	115.5275365
34.99891396	104.0151633
35.004797	109.3875903
35.03970904	119.1446291
35.00945845	119.8457649
34.99513407	116.5834863
34.97786954	115.1165245
35.00790039	117.4517743
35.00064444	113.8668685
35.03024148	119.7593986
35.05406284	127.9980066
35.07591839	128.2679471
35.00347288	125.8191688
35.01276682	118.3743439
34.98735222	108.6935164
35.01263633	111.0185717
35.02171726	109.7484733
34.99474378	111.955918
34.98845937	113.9707801

35.02367776	117.1787957
35.02639253	121.433005
34.98100622	126.022382
34.98025284	128.7862639
35.04150302	131.0941778
34.97309784	132.9216334
34.99988749	129.3801727
35.01314392	126.2965408
35.00455474	123.1467141
34.98890738	123.2972653
35.00125679	165.0931211
35.0480015	174.7080396
35.01093612	175.9088959
34.99317101	181.508086
35.00573504	181.156259
35.01322822	178.7582514
35.00564842	175.8636911
35.00737351	170.8923304
34.97687196	159.1451993
35.01338916	156.0144448
34.96100556	150.0220506
34.99475431	139.0100653
34.97921115	130.4115073
34.99137996	121.0648543
35.0089461	113.4369434
34.98595361	111.5337672
35.00503669	106.1438516
35.01322015	113.0478376
35.041885	106.7138763
35.02443245	108.0916413
35.00250829	105.4105152
35.01198692	106.2314446
35.01896457	104.5153963
34.96844214	106.2195463
34.96441369	106.9286583
34.99929026	111.3344879
35.03163572	107.9963578
35.04012567	108.8824074
34.98290055	108.5975302
35.02902524	112.0146678
35.00180902	112.3862909
35.0161501	103.2788848
35.01309347	368.9651081
35.0030953	340.8510451
35.00150603	330.2988415
35.00748867	375.2446972
35.00676583	393.6320468
35.02702953	396.367256
34.98943136	396.5751471
35.00426793	405.8559184

35.00964705	380.7133053
34.97701102	352.8367344
34.98254448	338.1335421
35.00948432	332.3216613
35.01580574	325.7951247
34.98855891	305.2055993
35.0113083	280.9152529
35.0208894	258.6003122
34.96488743	243.1949633
35.01874548	224.5421636
34.99279461	210.4216673
35.01187511	207.5983967
34.9957163	205.9618772
35.00209881	200.1751813
35.01175409	187.7097524
35.04239947	180.0518847
35.00121907	183.1324265
34.99693706	182.3328948
35.0115343	180.4448999
34.99780233	180.9327827
35.03862372	176.6781216
34.99107207	172.0100541
35.0291999	165.4043105
35.02987102	169.3742444
34.99777853	252.387473
35.02137119	253.1518977
35.02724212	239.6596037
34.98733959	233.6361905
34.99677557	229.5002667
34.97018727	220.6918427
35.00853573	207.3591507
34.96421492	202.3488801
34.98797993	191.6271134
35.01963607	180.7124642
35.00471608	168.3133126
35.03487686	160.6289868
35.02351681	161.9625593
35.01077691	168.9980293
35.03126396	151.7635143
35.00650471	148.2831769
34.98429756	142.4949622
34.99192771	141.0212099
35.00969418	143.2351832
35.01961572	136.7517771
35.0384495	133.7289417
35.01866941	127.0819548
35.00971011	122.6606312
34.98918768	112.7944058
35.07168559	111.4098208
34.9753472	116.1313284

35.01960447	103.9097138
35.01725648	106.9922637
35.00872782	104.2281374
35.01688051	98.59142232
35.00472694	96.37022367
35.00318977	95.51918174
34.99529596	261.0367039
35.02239255	249.6112922
34.99245851	242.2551934
35.02984129	233.4669712
35.04309874	229.0760389
34.9993687	226.5841546
34.98066533	210.2062809
35.00381086	207.1328339
34.98669892	189.6346282
35.00885741	181.9864038
35.02040465	175.140417
34.99468834	171.412448
34.98417507	168.8943411
34.99857988	171.5970274
35.06579826	161.5685293
34.99420306	159.5378713
34.99704984	152.9107814
35.01289508	155.1727078
34.97084594	152.7600518
34.98323965	146.095726
34.97335555	140.9137162
34.81007714	140.3722876
34.84413596	135.9591086
34.82701468	129.4652837
34.79699436	119.4485205
34.84283458	116.6399248
34.80779263	109.4833615
34.84183115	106.5411782
34.85517638	102.5881989
34.80356225	97.65020026
34.82194132	98.81354435
34.31661742	92.36733422
34.97365945	227.8273258
35.01067679	226.6392637
35.0371155	235.7037085
34.99291559	239.4815409
35.04085557	232.6260571
34.99000693	230.4908781
35.02625496	219.9908926
34.99448228	217.4739261
35.01522034	219.3704406
34.99986144	217.626395
35.02858983	200.02022
34.99091738	190.8914942

35.0329966	189.943034
35.03006663	192.7547743
35.02624792	195.0763187
35.0202969	204.8380527
34.99436781	192.91646
34.98042539	188.2504032
35.04454508	177.7595713
35.00193167	177.0371385
34.99611313	172.0054523
35.02840655	164.7295375
35.00289343	159.7874189
35.0325133	151.2903831
35.02638046	149.3118662
34.99147868	150.867473
34.99696168	142.8376187
35.02314836	144.1365647
35.01951584	140.7700078
34.98355444	133.6125055
34.99574668	129.6201057
35.04584865	120.3127237
35.01680449	173.553523
35.00335591	181.5751286
34.97422527	199.0274293
34.99383667	207.0012314
34.96055283	216.0719727
34.9993687	233.709359
35.02140079	245.9452487
35.00747748	256.7485588
35.0284039	269.7603122
35.04735827	273.9139246
35.0191988	272.5178946
35.00853001	270.5320772
34.99782811	263.7893001
35.01339656	245.3902871
34.98447977	228.4719522
35.04022619	230.5090027
35.02160513	241.4538203
35.01568667	246.6493461
35.04521733	238.7168903
35.00469766	234.5436519
35.02940844	227.7345477
34.96176505	222.9032692
35.04342465	217.8120415
35.03274999	205.3855296
34.99977038	202.4782792
35.05734353	197.9018592
35.03092465	192.2912684
35.01322015	186.1781535
35.07162783	184.8934529
34.99059115	186.7327467

34.97778055	166.1599654
34.9792052	160.883959
34.97399589	129.6653681
34.9639643	126.6981572
34.98122261	125.2668042
35.01553287	127.1369589
34.98258268	131.0440506
35.00453966	129.2843196
34.96363848	127.5140866
34.99780233	131.0923242
35.02695801	140.7781282
35.01350198	145.446124
34.97942625	144.2069283
34.99284919	142.8344562
35.0267682	143.4820024
34.9953471	145.6969005
35.01019577	141.0632153
35.00339785	133.189122
34.9677058	131.1170983
35.01262692	123.1814623
34.98594858	122.8440745
34.99111694	134.9970483
35.01716553	143.2723049
35.01448483	149.3073093
35.00494455	150.568588
34.99395252	154.5043866
35.02066553	160.3855304
35.00691839	168.2379443
35.00701987	175.8892074
35.01707913	181.5468772
35.00000223	183.2085412
34.99546857	182.928017
35.01036967	193.6618853
35.00738096	177.8180183
35.03618806	234.715152
35.01615705	243.1987017
35.03312807	232.9195491
34.98130701	220.667075
35.00982999	211.7798927
35.00146854	206.7278775
35.00821215	198.1030139
35.06832909	193.3371556
34.99855856	180.7986649
34.96190916	176.6169052
34.98197246	159.2953421
35.0436056	150.0652453
34.98401176	142.3637506
34.96573672	138.8847069
34.99295758	132.857722
34.99079436	127.5884107

34.98718385	125.4718865
34.99259194	124.314612
34.96994342	122.4332279
35.01873221	118.5038871
34.98617208	108.1115133
34.9671943	101.8387462
34.98030752	100.1252866
34.98047725	94.57902128
35.02290762	87.81852332
34.98603675	88.05895463
35.00022606	83.41188435
35.02792148	83.80130412
34.98485604	83.91169171
35.01603102	80.17893873
34.99395252	78.79668139
34.81982118	78.44489062
34.9818141	243.0514745
34.97879893	248.2064419
34.97007928	262.4195137
35.0039334	260.426697
34.99790388	259.0631126
35.00561689	265.6924498
35.03060098	272.631317
34.97092379	263.8012364
35.01176289	250.7640802
35.01223735	233.996768
35.0044278	214.6347049
34.99854348	211.4892073
35.02351681	210.7688152
34.99328381	204.0873036
34.99543464	194.344471
34.99136569	189.4161057
34.98434681	180.998895
35.0330088	170.814503
34.9958103	168.1639099
35.01281956	167.1118878
34.9876057	163.2649655
34.99855856	149.654497
34.97960015	147.5155329
34.98904924	137.3418147
35.02837636	140.1933275
35.00263382	137.5551982
35.00501576	132.551554
34.98565446	128.6751228
34.99286162	125.9232798
35.04513441	121.0567541
34.99059115	116.6999214
35.01631134	119.1761636
35.03291346	274.084638
35.04523811	277.4868454

35.0121394	276.3546393
34.9878714	286.7303332
35.02512541	302.3187967
35.03065983	313.4535005
35.0168107	323.9345444
34.96176505	328.2482518
34.99982031	294.4246224
35.00386028	282.7599351
34.9760462	273.1327791
35.00030962	249.8290424
34.98047725	227.9426069
34.98521773	221.2845755
35.02421345	212.8779088
34.99941619	208.3923912
34.97954378	199.5219667
35.03284802	191.3714804
34.99757765	186.3435157
34.99506655	179.22342
35.01157722	170.6407456
34.98213196	166.048308
34.98525086	159.9875111
35.040482	151.2866968
35.0018891	150.7757652
35.04340973	152.920212
35.01389256	145.6641278
34.97606312	146.0965529
35.00635321	140.2219897
35.04118309	140.3521291
35.03708972	144.7387495
34.99376205	139.2898579
35.0233337	292.2312453
35.0430841	284.0542425
35.01714941	275.4340628
35.03106012	268.5582592
34.99634469	258.2219627
34.99800261	257.9459759
35.01638481	245.6387531
35.0171145	254.311496
34.97769193	253.2432361
34.99711048	246.7055241
35.04505157	234.6456357
35.01558519	231.9649185
35.02456774	225.5632797
34.99999268	223.0364436
35.01151537	216.6150956
35.02665703	219.6033765
35.00517253	214.7487856
34.97780868	215.6409168
35.00258817	206.566504
35.0152327	203.9016372

34.97128821	194.804945
34.98863701	191.7295133
35.000874	182.8164655
34.98327842	175.8516031
35.04281162	169.6280202
34.98332976	173.6206139
35.04393113	165.2169359
35.01414647	166.4619299
35.06358234	162.8879338
35.02147523	153.1769482
34.97824384	154.4145062
34.83806148	149.9278771
34.9823915	93.31914574
34.98042539	101.4437988
34.97135748	107.310867
34.98097908	113.1708126
34.99464366	106.3525851
35.01970592	90.37344853
35.02254242	85.61237947
35.00051491	85.42384502
35.01316889	85.19591388
34.98932862	79.0267979
35.00888192	69.19767368
34.98227965	65.6954415
35.02951777	56.52953497
34.98385098	55.9953766
35.00803159	51.15598064
35.03762289	45.33151049
34.83885444	40.92950844
34.82751712	38.88629125
34.85240232	35.81629068
34.81788709	33.35665147
34.81440989	31.7532895
34.84918697	29.25669588
34.80394061	28.32787748
34.81355145	25.58930068
34.34106102	21.398376
34.21463431	23.26326625
33.31659531	21.17401406
33.3676924	20.36276008
32.48414811	19.04735378
31.31748841	18.41117272
30.45633583	18.86297771
30.38033126	15.56761595
35.03639494	296.0926905
35.02214292	300.1687208
35.02114716	309.4470168
34.99795687	348.5417132
34.97907647	359.8597174
35.01895268	375.2816033

35.0256816	366.6302562
34.99383667	370.2557352
35.04583465	374.9429872
35.01837857	389.0847796
35.0304111	367.5451837
35.02400497	385.9777604
34.99199882	413.8587621
35.05804011	401.9809412
34.99281844	388.9168095
35.00377744	393.1154431
34.99500827	375.9343021
34.98549441	356.0509629
35.02413591	359.0366763
34.98029615	363.999835
35.02537989	366.1261347
34.97917887	370.7374787
35.02860407	337.076299
35.01366793	341.6776924
35.03480498	335.2764644
34.96872589	321.6940752
34.99064143	317.8686584
34.99427764	305.5471673
35.00781371	307.3023763
35.0137457	311.0779808
35.0166801	310.0625688
34.98127953	306.5246005
34.99501404	333.9561727
35.01383994	338.1344206
35.01120753	330.3523393
34.9820695	351.6147715
35.02090776	373.1622845
35.01690891	345.9649681
35.02047108	303.6857913
35.00533548	293.8125209
34.99067749	305.961717
34.998525	284.6187733
35.01744102	274.5407835
34.98053016	276.8943078
35.03002585	262.8234384
35.01885083	251.7650051
34.97236644	236.7391992
35.01175521	224.3804864
34.98819179	216.2897232
34.96986143	208.1127758
34.98797993	186.2547848
35.01569205	168.951099
34.96800955	151.987382
35.03415446	142.8031455
34.82100256	133.735035
34.91238681	116.1849872

34.80660215	107.998697
34.82633046	96.97446574
34.40370614	95.72750907
33.38222391	82.18729755
32.44742481	76.27651043
30.32802013	65.63505526
30.1365782	62.41950827
30.29606827	61.7604724
35.00480973	308.0565995
35.03806737	305.3850407
35.00682611	314.7213604
35.0144091	358.4791453
34.99475431	387.4678798
35.02458839	353.9018008
34.98697332	294.9049988
34.98104551	279.0830723
35.00770992	289.333459
35.01439014	266.1556807
35.02770315	255.1342776
34.96319767	260.8635567
34.98451908	251.6524681
34.98271951	247.1171241
35.03220068	244.9724844
35.0518572	249.4460627
34.99310012	259.8379004
34.96014142	255.8786823
35.0259453	230.3151536
35.00803438	217.2004279
35.01582117	212.2864929
35.00022383	204.0315678
35.00444753	193.6877919
34.9939748	187.7358408
35.01225981	174.405122
35.0401287	162.7927864
34.85721175	151.7148405
34.84003426	142.2702275
34.8951187	137.5772848
34.79769596	128.5183798
34.36658576	129.0026828
33.51582635	126.7843328
34.99580001	330.2903449
34.98892896	339.8491306
34.99757765	330.2030778
34.98837304	360.0803545
34.98940537	362.0285834
34.99124508	346.7108009
34.99614563	321.9392608
35.05299023	300.9587871
35.00095436	288.4857812
34.98930828	271.3330276

35.02665703	260.2946322
35.00219925	273.5313764
34.99000693	267.4534887
35.00115058	267.4988989
35.01615516	263.4808184
35.00907046	278.110366
34.9784732	277.4267641
35.0230802	278.2233078
35.01794318	265.8517935
35.030559	242.1008886
34.97288243	245.9569958
35.04569493	238.8387113
34.98237724	231.4970772
34.85702293	227.8799531
34.8453786	215.6145772
34.8216466	218.5950427
34.34055868	212.641551
32.40917919	195.675056
30.30785959	186.7304902
30.4583291	175.4640721
30.02405601	169.6312561
28.66737632	168.4102557
34.97937747	369.1870588
35.01445674	404.9310686
34.969768	398.4816276
34.99445348	425.518032
35.00175133	476.9865625
34.98990187	502.39169
35.00077601	484.2699257
34.99498185	437.9865025
35.00906858	404.6472946
34.98653112	399.5804787
34.98683522	385.2668201
35.03138044	363.0430201
34.97892425	362.8433905
34.98980234	357.2787792
35.0016526	340.4587138
35.00914641	349.8788877
34.98117236	330.4152536
35.00674086	315.8241173
35.00284189	301.3286148
35.0261847	299.2843639
34.9767073	311.6467328
34.9794177	277.7294349
34.9900758	271.6393203
35.01762948	253.3577598
35.02216474	255.0027832
35.01388411	247.2258126
35.02344481	225.9720454
34.96584251	205.3766934

34.98132203	203.6799905
35.04471578	199.6817683
35.01475476	194.0854468
35.02803838	195.4894731
34.97423892	276.2928095
34.97140253	272.4177907
34.97187728	289.7386791
35.04168853	347.3409426
34.96238953	374.8714241
34.99650053	350.5740675
35.01443884	325.9369548
34.98822471	305.9780085
35.03328727	302.0596546
34.98515694	328.9201282
35.00676583	342.9650696
34.99556382	347.9292806
35.00294702	360.4752953
34.98382577	365.8143115
34.99316029	346.6250274
34.9770011	353.53923
34.9909569	324.3176267
35.00159946	308.7724558
34.99450164	310.5737849
34.98789848	294.5708108
34.99561074	296.5188898
35.02490626	274.8252886
34.98933633	263.0629619
35.00300249	249.124661
34.99216877	239.5381855
34.98687995	226.4201878
34.98685691	215.8900147
35.01195456	196.6189206
34.9814553	192.1905605
34.98896532	184.2256471
35.02037155	179.8457134
34.9960275	177.9179367
34.97087748	303.0858971
35.05820821	320.5410451
35.04021708	344.7967229
35.00594625	379.9035821
35.06085905	405.4896264
35.04337876	436.9753557
34.99740243	441.4872943
35.02678364	442.3292628
34.99083717	430.7163832
35.0143385	461.8033603
34.99973619	493.4117437
34.96363848	497.9414128
34.99552528	516.7974466
34.97203898	521.0520374

35.02665703	510.6972972
35.00160428	500.2345087
34.97440002	470.73604
34.98366585	473.9751742
35.00281652	472.4082951
35.03479955	433.5464284
34.97449013	421.9093236
34.99583537	398.73545
35.0384993	392.8251268
34.97274045	390.2005159
35.0180174	380.8739405
35.03068212	371.1569777
34.98767179	362.3433816
34.98747151	357.0441827
34.98170311	360.154964
34.98649255	360.6459335
35.08302892	334.7987035
35.01691748	338.4490326
35.04306581	114.5813098
34.96760905	112.4691868
34.97993539	109.6536243
34.99580001	106.1814263
34.97521697	100.2294168
34.9874359	94.68365793
35.00142567	88.52358465
35.02656514	85.80858497
35.02484343	86.78520985
35.0539922	85.89650817
35.01366793	81.72491858
35.01492651	78.51098728
34.98642561	77.88097497
35.00051677	78.30237486
34.9665708	76.86008266
35.01679479	69.97575114
34.95839288	65.28542341
35.01254415	64.03996998
35.02705693	62.70527279
35.00415305	63.60393384
35.00539949	64.24489309
34.98317145	64.40723762
35.03567724	64.34011768
34.97309784	69.97777233
35.00020041	71.7601421
34.97913279	72.02586577
34.99199783	75.86822129
35.02702953	71.73575404
34.98312572	62.55709359
34.96951221	59.53707475
34.97240169	50.07322963
34.87218424	49.63258294

34.97961272	31.70630182
34.99363953	29.32688057
35.0358789	27.23814491
34.99960516	24.81960189
34.9985252	20.15455225
34.80613358	16.38915767
34.80969672	12.86890579
34.83463459	10.3528075
34.84623489	9.319165069
34.87046419	8.421272291
34.79776252	5.678949911
34.78547311	4.061957121
34.81150854	2.963711149
34.30730776	3.02359218
33.64939959	2.777216598
32.47162304	4.160134228
31.63688781	4.034206269
31.56564253	3.075919416
30.5520963	3.424766874
30.42948849	2.787987074
30.39462275	2.23967768
30.41442118	1.9503815
30.3072758	1.116708551
30.11903597	0.145714594
29.21630563	0
28.0328706	0
27.45727843	0
26.84334827	0
26.49432998	0
26.04394569	0
25.17262962	0
25.25632566	0.099195306
35.03501082	25.1141809
35.0365881	25.73180207
35.00922855	26.3987161
35.00252394	26.96553297
34.99314239	27.00061863
34.98617833	25.82170472
34.99133923	24.91804345
35.04147758	25.34401373
34.97966616	25.92679364
35.01892799	26.1763224
34.97558832	24.19392363
35.00376032	21.09002733
35.02649999	19.95003347
34.96897918	18.44685023
34.84983579	17.13694724
34.86630934	15.22005918
34.78929028	12.8633492
34.37356549	10.34824644

32.35758976	9.361370632
31.51903711	7.871934703
30.41940764	7.591157966
30.48729687	6.498475771
30.41187733	5.370387929
29.55924647	4.660649645
27.86738896	4.867449863
27.17039766	4.617815109
26.35216882	4.704535892
25.52884939	4.467331298
25.53579589	3.985933085
25.36714558	2.882610224
25.43621782	1.983279136
25.27653987	2.996259227
34.98693063	113.2329874
35.02457588	103.8701421
35.01151537	102.8953039
35.04171076	99.52818298
34.99781172	96.81364161
34.98747151	97.03128056
35.00276302	97.75845579
35.02428749	95.42271645
34.99839512	91.37898708
34.98658575	88.07804848
35.00945287	81.74190735
35.00642334	77.57307041
35.00570009	70.82152878
34.99251582	66.93387667
35.01482519	62.53323856
35.02275549	54.17011026
34.98988397	53.31469672
34.84790175	54.08970018
34.84417612	51.82094559
34.80016762	44.68612122
34.42062498	39.45041524
32.36762957	33.86277955
31.38593607	30.17824812
30.47989548	27.56120559
30.26085552	26.50835204
30.59376116	25.09037504
30.50348993	22.69497721
30.43770909	23.08044426
30.47158539	22.37585259
30.20724629	20.45204701
29.46705859	19.70278146
28.46654394	18.27116114
35.00105014	140.0656714
34.98416985	130.0967786
34.99083957	126.4399465
34.99217698	119.1225186

35.05645282	115.5885763
34.98685691	109.1048849
35.01653082	103.6513394
34.98074068	96.57098604
34.96306204	90.04048366
35.01454369	82.62542472
34.96262582	74.73951056
35.04057104	68.44683335
34.98457071	63.64194025
35.00362834	58.2842124
34.99689329	52.66679978
34.97753856	47.41686853
34.96014142	43.26587843
35.0029485	41.55694558
35.0221611	40.06653583
34.99212668	36.30324755
34.99638635	32.57149421
34.98871201	28.81877433
34.82023546	24.51983521
34.81336102	22.05202063
34.86143204	20.84801481
34.78770201	19.10424272
34.42727023	16.32233546
33.42589944	14.69259257
32.5229528	12.82910606
30.32727993	15.59673432
30.45428923	12.34332437
30.27079893	13.81104097
34.98598836	150.382373
34.99101739	143.1953105
34.98966258	142.0557378
35.02018886	136.8285872
35.01300416	128.5723839
34.99301305	125.9739725
35.01469419	118.1340635
34.99561074	110.6369546
35.00401184	104.418698
35.00867268	101.6104307
35.00573201	94.52688017
35.05091551	92.22619323
35.00635153	88.42069112
35.00535858	83.99357079
34.9923504	80.34019373
35.06189362	73.81112599
35.01378926	68.1721295
34.99387125	62.85195294
35.00444011	58.44668486
34.9938687	55.07033444
35.00919223	51.59850457
34.85361666	49.50433826

34.84355276	45.44159542
34.8298452	41.14554507
34.79753939	38.86660819
34.37694567	36.30505133
33.31438749	33.54705905
31.44706185	34.57072353
30.52261726	34.25234912
30.3142672	33.70698739
30.71126677	28.98523146
30.35211276	30.63286324
35.06023635	48.50665089
35.04120648	47.58165502
34.98681478	47.4551612
34.99989981	46.37130144
34.99281844	45.16410413
34.985589	43.40564259
35.03897252	42.12044983
34.98903308	40.60859257
34.97722789	39.22106689
35.04377028	37.73828515
35.01688051	36.47336295
34.98029932	30.80141315
34.99588989	26.72647175
35.05859386	24.43633099
35.00072508	22.80428281
34.84920698	20.48387544
34.82197269	19.31496132
34.79956753	17.50848085
33.566858	19.85075417
32.56649526	21.7827559
30.61909702	25.18973232
30.56520422	24.96902087
30.2751432	24.53549842
30.19414284	23.95404676
30.38548792	23.35101376
30.03735019	23.17254844
28.7178111	23.79673092
27.82077429	23.9246508
27.23495925	23.73103199
26.86692142	22.56466681
26.24879409	14.74768602
25.39748538	15.62662272
34.97987718	128.515797
35.0220079	133.3093505
34.98510193	133.7229963
34.97898414	132.9024148
35.02601975	131.0303166
34.97284421	129.173239
35.01604913	129.21062
35.04165269	127.6707775

35.00377744	127.4952827
34.99249945	122.3787232
34.97343969	110.8249224
34.99749705	103.0874356
34.9928198	97.39347583
35.00309094	90.29678957
34.96921221	82.51885438
34.98560037	76.88344829
34.91348105	73.64863617
34.86333974	71.83037569
34.87131731	68.37998651
34.84679904	67.63243532
34.8089685	63.30628854
34.35227882	60.29804953
33.25676994	58.82149519
33.29952194	56.2329959
32.51972393	53.12157004
31.57883197	52.60847294
30.60000386	50.37389088
30.77150611	47.48633122
30.48373462	46.97972683
30.47506257	43.44472666
30.03637938	43.45326103
29.64480984	38.46518814
35.01484406	99.47153021
34.99529596	93.49919912
34.99454435	88.27484401
34.99777853	85.15721114
34.98460047	80.11622384
35.00672914	77.90478436
34.98630169	77.22539771
35.07671875	72.11092317
35.03387589	72.22959944
34.99163639	68.49656117
34.99706451	62.64038832
35.03614273	58.00628144
35.02062825	57.58596625
34.9951785	56.59374102
35.010015	50.33386375
34.96413806	44.78238151
34.98659685	39.26171084
34.99907396	36.42651277
34.98303488	33.8487752
34.83800092	30.66661801
34.8569634	26.77015246
34.83924066	22.97619178
34.77787949	21.09530335
34.40740701	19.32177118
33.43217724	17.21056644
32.48232757	17.39904247

30.70764488	16.63812078
30.36878811	15.06070643
30.30589637	13.16197365
30.11753565	11.60719028
30.30302397	9.273837813
30.03244647	8.005999633
34.99397268	236.5119897
35.00308316	240.3804517
35.01849775	242.7599482
35.00206551	233.8534951
34.98852148	213.3626232
34.97852104	205.5094223
35.01589089	195.5816164
35.00714401	197.3268617
35.00215717	192.4816095
34.98693063	175.8996226
35.02995772	163.1309817
34.99233308	152.1333091
34.98504025	142.9384021
35.00441041	133.090428
35.00620783	116.9171001
35.05055747	107.2726704
35.01112191	102.5298694
34.99880827	96.92691597
34.99124198	91.51395478
35.04110553	88.94772139
34.85657198	89.19769517
34.83323792	85.05129432
34.83725174	78.7337288
34.80584811	73.31854849
34.86230904	69.78718594
34.82698921	67.47656166
34.80325303	62.74936514
34.81663359	63.19602974
34.33414174	61.90368982
33.42806026	61.92638579
33.26305104	56.48067563
32.34711983	54.60387802
35.01471422	177.5132857
35.02898762	169.5766686
35.00273674	165.7793398
34.84476044	161.3784428
34.80512409	151.0071024
34.86988796	143.5973973
34.80436233	131.053977
34.30236446	119.5084735
33.41244642	108.7533133
32.55182289	103.7635181
30.57130592	93.61129549
30.19351951	90.49410748

30.4760475	82.08921718
30.47887421	74.54167121
30.1008268	66.69667498
29.50582589	60.25886193
28.55847723	55.96309599
27.91698721	51.93829354
27.25353183	47.72068366
27.07704884	44.12736937
26.92469932	39.39780533
25.63314912	34.71568912
25.31797485	31.38649837
25.23215538	28.13152055
25.23077977	24.81174217
25.3407657	22.71064434
25.3605615	21.18515245
25.34326393	19.27635404
24.94222215	18.45629366
23.96377412	17.89175315
23.70712265	16.61323189
23.84534264	16.10033272
34.99891665	180.8691384
34.99864548	173.398602
34.96197321	170.6050822
35.05765541	163.9502267
34.99122779	152.7614247
35.015898	148.0553995
34.96213974	138.8160947
35.02585296	133.7891737
34.9976035	129.6700703
34.97720852	127.249619
35.0013093	122.9715059
34.98158136	117.2006897
34.99868817	115.1451541
34.9972885	109.2180549
35.02796077	102.7010249
34.99397145	97.23631484
34.81425471	94.13514831
34.83307024	92.22851104
34.86250699	88.00413051
34.81182075	84.10828823
34.81293176	81.0280108
34.38357491	75.76540045
33.54031344	73.03344196
32.45557266	69.36778287
31.59714623	66.48744003
30.41650546	63.82534545
30.14563218	60.58557411
30.57128917	56.50393121
29.98362685	53.35395
29.55260993	51.23826457

28.38350571	50.11774756
27.84479557	49.5378397
34.98597851	49.27512669
35.04651888	46.99294968
35.00916351	44.35332898
34.97796533	42.82096735
35.00020499	40.29973082
35.0113083	39.65802134
35.00782793	38.66388896
35.03439483	35.69522224
35.00730436	33.96608548
35.03229235	32.07534895
35.0255594	30.34529687
34.97987718	28.82574859
35.04092983	27.34342086
34.99184237	25.36358309
35.01016291	20.81495592
35.03188001	18.78974665
35.03177487	16.27690758
35.024414	14.33047072
34.96229346	12.7750272
35.01709697	10.72720356
35.04329784	8.447837303
35.01063307	6.502069832
34.85590919	5.387254708
34.80512709	3.432656649
34.826551	1.455463947
34.85228289	1.235453113
34.79065045	0.0157181
33.99216535	0
32.92034605	0
31.95159869	0
31.11130085	0
29.87362192	0
34.85162866	157.5474038
34.81561269	153.7277317
34.37813264	147.963795
33.29367445	146.7045298
32.41398798	140.310954
31.59689199	133.3221275
30.5016185	130.2262093
30.49319547	126.6619708
30.31021928	123.6074688
30.42554127	116.5348588
30.49734014	105.7823714
30.0403478	101.6609682
29.7260365	100.4034278
28.61140193	98.07860939
27.79921393	87.93699352
27.23695882	81.13759919

26.92297453	77.35919719
25.3052953	76.25800756
25.49755916	72.19120512
25.55678786	67.51769237
25.337124	62.34407956
25.36600729	59.07751031
25.30853225	57.94540585
25.25386368	56.9855421
24.84215896	53.16388034
23.97361355	53.41265767
23.86853238	51.93026156
23.81021936	48.94269784
23.94983627	46.81772335
23.9294431	43.0656156
23.79796118	41.81434573
23.71978819	39.95111805
35.04817839	106.996162
34.98970791	104.093649
35.01120886	101.9798635
35.01133944	98.72153172
35.01987051	98.24500365
35.03718375	99.21642935
35.01781275	98.80342371
35.01419548	95.80407204
35.00363899	93.77734796
34.99521121	92.35776311
35.06424995	88.96500353
34.97565272	86.40091082
34.99763585	85.36211727
35.05243313	86.34986103
34.9957163	82.80072881
35.00993361	76.67256636
34.98281071	74.98619052
35.0283214	85.58601523
34.98781666	69.99243937
35.01173657	77.43124459
35.01063947	76.34816079
34.96008883	73.77042731
34.98980234	69.18282505
34.99913954	57.65850901
35.03301862	44.5687114
35.01720204	40.12149276
35.02607086	37.51921966
34.99238297	38.25000323
35.03288991	37.35655941
35.02752988	37.66529072
35.00286364	37.92491315
34.97475848	37.92835639
35.00320149	131.198953
34.96027923	131.221784

35.02630052	127.7098665
34.99946932	121.5601794
35.05358071	120.3747812
35.01889013	114.9945399
34.98122869	110.9640339
35.03762287	106.8668551
35.00263145	102.2056028
35.05766546	94.80484924
34.9882979	86.0283465
35.01960132	83.61829754
34.80392634	82.60996155
34.8323567	79.22580146
34.86135216	75.52189139
34.82600472	68.37063968
34.81399743	66.88773143
34.30521693	63.07205569
32.43534965	63.85364163
31.65532233	61.68578898
30.29774205	61.31907477
30.44652281	58.68609812
30.67184612	54.61664882
30.38549289	52.091454
30.16402118	48.26751739
28.5764907	46.40080299
27.27386391	43.67045987
26.90688628	40.90064717
25.60886815	38.73499735
25.34833086	37.25985052
25.3753163	33.49745829
25.59145745	32.36326562
35.03304657	191.9740121
35.02659589	183.2076102
35.00883059	177.5583148
34.97595301	177.5133748
35.00866167	174.0151531
35.04523811	171.1680918
35.02309001	162.445137
34.96897648	153.978685
35.00113037	148.7336595
35.0157417	141.2090727
35.00383252	128.7988307
34.97913279	115.1182595
35.00108746	107.4268323
34.98781666	97.793832
34.99812254	91.3981135
34.84275631	82.95812886
34.82138833	79.20934607
34.81821441	72.72273291
34.83805528	66.11621976
34.8090902	60.70072476

34.31633036	53.38540559
33.49915671	52.72295248
32.55143134	50.92320549
30.74659913	49.28769428
30.39612083	44.9506005
30.51706787	43.726561
30.1198031	42.86206862
28.71339399	39.91792711
27.1476882	39.00080773
25.94421905	36.85215345
25.25864673	32.19645882
25.20995439	27.55542059
35.00583634	142.2933583
34.98277513	146.7568785
34.98902583	147.6332298
35.02344296	141.4120856
35.01296156	137.5305781
35.01186957	119.1375544
35.00446609	94.61827879
35.01033059	89.78885025
34.99400726	91.11238865
35.03415949	78.6379257
34.99738157	68.30815428
34.98283532	61.03319657
35.06923619	56.34512791
35.00907046	52.06154839
34.9876712	45.10769658
35.02242674	41.00924225
35.00914641	38.04307371
35.00584781	36.49740848
34.97378582	30.4777706
34.98436061	24.69221302
35.03226055	20.28958766
34.98764144	18.47056987
35.02504608	14.60368304
35.03261123	12.38280194
34.9894761	10.06330042
34.96613639	9.059102733
35.04139162	7.264873813
34.99431718	5.420387863
35.0018229	3.177376815
35.02803846	2.421007095
35.03307336	0.744824225
34.77493235	0
34.99036167	73.51041792
35.03854469	71.91974624
34.99376205	68.93542234
34.9879298	67.94063008
35.03153413	66.4558179
35.00429052	64.09748856

35.01743119	60.76673178
34.96882782	59.10776528
35.0069021	59.21615271
34.99955525	55.62906327
35.02754663	50.22449813
35.01403018	45.72761278
35.05616334	45.19501805
35.00704791	46.10175544
35.01314392	41.42973102
35.01747564	39.20964892
35.00357225	34.804459
34.98054136	32.57607415
34.99752704	31.02105417
34.98146816	29.10204003
34.98130701	26.58059939
34.99346536	25.02457299
34.96928078	23.35214776
34.97213443	20.87330862
34.99754077	19.00403772
35.00167739	19.50623642
34.97921115	15.80914017
35.03967629	14.16553906
34.97855952	11.70174094
35.01067628	10.25095927
35.0180059	10.7125465
34.9968048	9.275371722
35.00790145	118.8194429
34.98045258	115.051317
34.99266018	108.858813
35.01762712	106.2586029
35.00062009	102.3395169
34.97183212	96.79625748
34.97255504	97.27364484
34.84022184	91.65050202
34.86095035	83.46606537
34.85376806	73.21157123
34.81267358	68.88593187
34.7901568	65.12856417
34.42312366	64.64428958
33.31876204	67.52830675
33.56787867	62.00957103
31.49233524	59.46862845
30.50045444	57.24702461
30.313025	50.49014268
30.39799464	45.25715396
30.33322742	38.3594678
30.07347244	34.27484372
28.53125369	34.9914581
28.07133933	33.33082113
27.16519509	33.14931021

26.87739671	31.6320314
25.44126876	31.81717842
25.33533302	31.97169208
25.3503257	28.54500907
25.38991838	27.56516886
25.59920532	24.95670622
25.24264326	22.3740529
25.2402482	20.45895555
35.04168591	172.6635627
34.98867356	162.5666546
34.990712	155.2476533
35.03280007	144.3623762
35.00053691	135.3729214
34.99941619	131.1331708
35.04268177	130.1132963
35.02526134	130.3401498
34.99671947	124.6759394
35.03859576	129.2716796
35.00201509	121.9990335
34.99478104	114.0119289
35.02633273	109.1471522
35.02029131	104.3874334
35.04359685	96.9105169
35.01061959	96.55777289
34.98631079	89.48583592
35.00022204	79.97264776
35.0359559	74.43650652
34.99689329	70.78435466
34.98587134	70.44101689
34.97770252	75.26676657
35.02952924	77.08307592
34.99419391	65.87822125
35.02952449	57.75199568
35.00096952	51.6261817
35.00666638	44.61113145
35.00476543	41.98220448
34.96642186	40.37753706
35.00540215	38.39206563
34.988168	32.33994108
34.99576223	36.19168768
34.99823011	106.6311432
35.02481882	101.7776185
35.00030962	98.08875542
34.98784023	96.8577608
34.849056	91.11733363
34.80822624	88.10306887
34.87421825	84.50465765
34.86413751	78.90821038
34.80387318	74.99292932
34.3417368	70.88546949

33.30375568	65.01869184
32.46491506	60.27408299
30.36188234	57.9825113
30.70222655	53.13675331
29.95613385	47.10153877
30.46520723	41.36088609
30.15954442	36.71916359
30.04576243	32.1882057
28.23323352	30.14586872
28.79839959	25.71880709
28.12042541	23.66504968
26.65915055	22.16729068
26.85259014	20.3286648
25.78476081	19.84149025
25.34888343	18.5105769
25.25277369	17.90139309
25.42386324	16.56451567
25.22334355	15.10590977
25.32049501	14.0325486
25.28427368	12.75077039
25.23688874	12.36807828
23.95471353	13.46198664
34.99776356	140.8183155
34.99919091	135.9276987
35.00189817	130.7522191
34.83244928	124.7275851
34.84298323	119.3381218
34.83932607	115.8276753
34.80523663	109.3394701
33.42589944	106.7929383
31.29595216	106.5238367
30.55804945	102.3940643
30.47412232	94.66593213
29.99934184	88.90622364
28.68379512	85.76034284
27.83073827	78.70888832
27.28847854	70.67831646
26.83152358	63.19024667
26.54434538	58.82843153
25.45137953	57.67861233
25.32516145	54.32856807
25.32174134	50.30306872
25.32591198	46.42474335
25.37035358	44.16178207
23.78636055	42.11126638
23.80747921	38.06955792
23.71659297	33.68463078
23.77769888	30.9592726
24.03640674	27.04720754
23.85576002	22.9272341

23.63741665	20.98692126
23.91978888	18.0666255
23.86538314	16.62769915
24.0128058	16.86349273
34.99491835	81.87747737
35.00331097	80.08780082
35.00490628	76.98839909
34.98868729	77.4586048
35.01874548	70.60949065
35.01961572	67.69726186
34.9892224	66.40462373
35.02379686	62.94221292
34.9944224	61.16090983
35.04657674	58.94177316
35.01743914	53.17466021
34.97878522	49.91353578
35.0060647	47.07728218
34.97944716	43.65463927
35.01835231	37.58712661
35.0087973	32.74438476
35.02170366	27.36261597
34.99119012	23.2666018
35.0021672	19.54575829
35.00153177	16.3186637
34.9876281	13.3891796
35.02318722	10.24297684
34.96613639	8.436932366
34.99840538	7.699436101
34.99979756	8.204445061
34.99933229	7.274498153
34.99118708	6.569714003
34.97023336	5.640095617
34.99062023	4.469469082
35.02644343	3.566016037
35.00790145	2.300803236
34.9907722	1.809662952
34.9938523	63.17535963
34.9903723	61.19786753
35.00953079	57.96842127
35.03646157	55.99854905
35.01918913	53.21080327
34.98066533	52.22590117
35.03510583	50.9765729
35.03394097	47.64056877
34.99452738	46.48754857
34.99667951	45.39122305
35.00426793	40.04996485
34.99771614	36.36860657
34.99480472	34.91028546
34.9639643	34.07585194

34.97309784	31.027557
35.02393659	29.20126788
35.03099638	25.60971941
34.99880617	23.49167836
34.98805095	20.81889583
34.97175645	17.46966416
35.02021694	14.60274606
35.00177003	12.65858578
34.99451446	11.04108263
34.99000693	8.551798211
35.03163177	6.933579937
35.05000244	7.143186708
34.98828161	5.504284266
35.0064457	4.754126854
34.98249225	4.191467757
35.00544492	2.667272696
34.98165516	1.589445923
34.98909007	0.378735815
35.01416263	146.5663549
35.02950431	142.0329344
35.02366755	136.1893749
34.98279306	132.6488337
35.02199286	123.2634473
34.82405418	119.7888466
34.82210888	114.4077777
34.86807241	109.1090509
34.77926838	105.6664352
34.42272107	101.6923016
33.31034945	95.31448342
32.37533773	91.9262267
31.00600254	89.97279114
30.33216662	85.26706884
30.46472753	79.50165607
30.56078295	74.32482618
30.06750372	72.38669566
30.06043482	68.8189593
29.67288525	65.0435537
29.40768002	61.23196904
27.99050845	58.9074718
27.33549548	56.4028715
26.69739197	53.54219294
26.44741431	49.16980757
25.36425782	46.38285157
25.37304096	43.22497389
25.38672655	40.37517594
25.35054261	37.22292191
25.4246283	34.19443501
25.25342914	30.20348411
25.29322468	28.82066856
24.80094155	26.99758464

27.42121039	114.0388077
26.71914861	112.4441278
26.36757468	107.6512711
25.43448223	103.7772693
25.2356956	99.96125933
25.44926635	96.66606928
25.57477809	91.4524084
25.47814321	88.82471937
25.51128404	85.43786837
25.35164225	80.8184145
25.31910605	74.5974993
23.62753063	70.8010664
23.84240081	67.33227708
23.74208436	63.96768028
23.9906232	58.52122322
23.72847484	55.67659169
23.87023376	51.8458131
23.78630546	48.42524537
23.89861918	45.20168508
23.71171056	42.52869116
23.8031266	38.37517246
23.76300274	35.0930871
23.69905717	33.91890227
24.23566924	30.73890608
23.87779736	27.93801125
23.67499192	26.06830847
24.01400308	23.1446708
23.75829259	21.89184242
23.93652396	20.74306287
23.90712875	18.33464081
23.75724893	18.67459681
23.84611019	16.11112883
34.97097226	161.5235001
34.97381114	156.9527114
35.01665171	152.0642071
35.0175372	147.1289878
34.97232235	136.6781671
34.98241679	131.2361744
34.98493092	124.684299
34.98524222	117.4621141
35.01323393	112.3391802
35.05364457	105.8794344
34.98384113	96.67935375
35.00776276	90.53543302
35.01742598	85.9174962
34.97689894	80.08389444
35.04479786	71.36828919
34.98450001	64.34498692
35.03517542	57.72434991
34.82988648	52.88752423

34.80135916	48.14519512
34.8356799	42.37252806
34.81492355	37.93686083
34.84621642	33.57038483
34.80394343	31.66246846
33.41286803	31.6084845
32.33954029	29.74089141
30.30017513	30.8099787
30.32533336	29.14097233
30.18996014	27.87470085
30.25929134	26.47872547
29.6913678	25.19790885
28.75067061	23.34138731
27.77377295	24.3351334
35.04069884	123.7628141
34.98909174	113.275632
34.97089505	109.9827739
34.97434724	103.5068783
34.96608105	93.2279146
34.98162211	91.09174763
35.00024794	86.4156417
34.99956278	80.51084998
35.01089508	80.25848088
34.99716242	79.2252114
35.02612556	69.28876932
35.01978749	65.47272848
34.97574593	64.11237108
35.01624012	61.03598874
34.96231606	55.59925656
34.99545693	52.69997096
35.00072508	51.30478884
35.00932797	52.09186686
34.97890741	47.90492932
35.03366324	46.79923034
34.96739874	47.07391125
35.00888192	46.24719123
35.00626412	44.51019699
35.01555766	42.0457981
34.96014142	38.49462507
34.97346956	38.5268763
35.01703498	40.4771754
34.97841002	37.38070585
35.00332269	36.02983707
35.0356172	34.19789635
35.01141292	31.26901472
34.98058809	25.81126674
34.98140831	83.49743175
35.02275922	74.64798118
35.00747024	68.94644415
35.03998299	65.98827399

35.02700911	61.99377172
34.98130588	58.55302096
35.0199207	53.71336179
35.01941219	48.88834418
34.98901352	46.75151358
35.07756394	43.95926505
35.10357301	38.91848822
34.99450164	37.0826895
35.02110563	33.96769895
35.01035232	33.26767933
35.04853219	31.18475963
34.99822237	28.60895191
34.98849956	26.44522684
35.11925364	24.68713517
34.99556382	20.3029484
35.02557265	18.69512925
34.99525654	18.31947126
35.00896074	15.57771325
34.97866217	15.08166175
35.03536834	13.99493602
34.96413806	12.94123063
35.0402661	12.37801432
35.02989523	10.49677093
35.01339656	9.164543296
35.00027261	7.581782374
34.98025962	6.936028926
35.00120912	3.72718787
34.97364878	4.076644498
34.9944224	117.2825149
35.01846313	112.2994765
34.96819037	106.212108
34.99316029	103.9162632
34.9809424	96.19153904
35.00397382	91.53840635
35.02215619	84.07982741
35.05023159	73.6922478
35.00306609	73.18032561
35.00720177	70.56448327
34.99297844	62.28131395
35.01007533	58.54231372
35.00767255	57.15828626
34.99410174	55.78991868
35.03541564	52.83135164
35.06632042	49.1758927
35.00640271	46.10381705
34.96950227	48.11086722
35.00790928	42.61882948
35.02448874	38.85419797
35.0450188	37.81008884
35.02512541	38.82401874

34.97208432	36.96717384
35.00469931	32.96820892
35.01880605	30.6042531
35.01176289	30.71610617
35.01008095	29.97134074
34.96319767	27.88772444
35.00191095	27.46533818
35.01001699	27.17272182
35.00157049	22.91528441
35.03653129	25.51182278
35.01629101	121.6423153
34.99068724	115.6188732
35.03946239	111.003758
34.96114058	109.0647309
35.00579803	106.1574197
35.02544212	103.3339716
35.01233128	99.50491575
34.97459312	95.59152517
35.00488934	90.99050075
34.81977096	86.39042891
34.83757427	80.26971125
34.83396098	74.38203538
34.79468352	69.63712116
34.42483905	65.09890623
33.24068627	61.10740111
33.51129413	56.69222206
32.45981454	52.00649009
31.39703043	49.3982055
30.63701696	47.15320218
30.31062374	43.39280862
30.53675306	39.57641268
30.44271286	35.70697437
30.45226824	33.78085033
29.55340573	33.10792276
28.37656679	33.49381525
28.06448758	30.45481491
27.1540319	30.80652386
27.08017064	30.41650649
26.39280132	29.77482855
25.72827783	28.74285208
25.33665498	27.17094935
25.35104018	25.76666589
34.99606066	144.8357666
35.05814511	139.4651199
35.00410112	134.8465378
35.02135252	134.6720395
34.96424608	131.554871
34.97509535	128.3645184
35.00335931	122.957527
35.03704322	117.599447

34.97478327	114.4487366
34.97654236	115.2202609
34.97545746	109.7440961
35.01365972	105.1861451
34.98303488	101.011136
34.97896383	95.17083234
35.00794282	87.71315284
34.99333747	83.35527244
35.015949	77.71784238
34.82776704	71.8998517
34.84743726	68.32081977
34.8560157	63.85031315
34.82216181	59.66369737
34.4313994	58.05137922
32.36717785	57.84751299
31.80883188	56.52360586
30.55703225	56.03202469
30.43365642	54.83485541
30.64005077	53.69986624
30.00552892	52.87082665
29.57117104	51.11482768
28.61598163	50.1702976
27.98287867	49.64344932
27.26663658	51.6544226
34.99451758	92.16478927
35.01671037	94.58338272
34.99487475	96.2509179
35.01887328	106.5765445
34.99744425	104.1407712
35.02081047	106.9074797
35.00067526	97.34553333
34.99661288	89.76332984
35.01032166	83.73421366
34.99236627	78.71842593
34.97626053	69.5046754
34.9645752	65.20802235
34.99810688	61.89964801
35.01047768	62.25824629
35.0157036	59.96944047
34.98676843	61.58785552
35.01322822	58.5919946
35.01316392	67.28984399
35.04065033	64.05217406
35.015288	61.15579541
35.01603102	58.64086085
35.02762396	53.8642428
34.99288044	51.59944618
34.98951865	49.02471428
35.03678147	46.18436014
34.98560037	42.0037145

35.0128934	39.43360112
34.98163482	41.87935933
34.9990676	41.32965132
34.98116117	41.50465616
35.03117994	38.71411892
35.02129131	36.40950154
34.98220594	95.37047222
35.0165193	92.53337758
34.99627844	90.02847784
35.0294551	89.5555875
34.98847237	87.83180417
34.99220429	86.5787767
34.96114058	85.25114683
34.98776542	78.75596868
35.04987978	75.47484982
35.05391318	81.32259092
34.99772593	80.82031375
35.00351468	76.44813801
35.02570112	68.9818977
34.99183085	61.3887771
35.01873445	58.61086945
34.99611333	54.0573421
34.96431973	54.16568959
35.01045203	47.38472823
34.99142956	49.92303508
35.01700091	50.51275921
35.01164257	51.35069514
35.02124968	50.12172244
35.02236055	47.28175182
34.96238953	44.70285132
35.00313958	46.03453458
35.02326799	46.08026863
34.99988321	47.36655959
34.9858774	44.92069889
35.03609597	43.96275273
34.98151086	43.57840107
35.02341975	42.02314044
34.98978113	41.31797134
35.0030736	85.80083659
34.99650053	78.34492399
34.99192396	78.29837331
34.99299311	75.49651969
34.99929026	74.77663045
34.96311566	71.39442643
35.01872703	69.86589797
35.039225	67.28473124
34.96955457	56.70808058
34.99363359	49.91366508
35.02856961	50.14889156
35.00726563	45.36163889

35.00885741	42.79519647
35.00726917	45.54837266
35.01672476	41.10120658
35.00235708	34.72117394
34.99035129	37.00175182
35.03082742	33.85955002
35.04502944	33.86108453
35.00711019	31.03639358
35.00150603	28.11821536
35.01121074	26.52346723
35.0089804	25.52126242
34.99561569	27.11741236
34.97558832	27.50821551
34.99450164	27.77119463
35.00371431	28.03141692
34.95947531	28.50292229
34.99972603	29.40174311
35.0284577	32.05078227
35.00973732	28.50189149
35.02607813	27.52119759
34.99961027	84.18757804
34.98312057	85.19892408
35.00495829	83.69114137
35.03271625	84.73039061
34.99344092	86.20710198
35.0018891	88.29497253
35.01479224	95.44462579
34.98887254	99.08203033
35.04478781	98.8818824
34.98042299	102.1373641
35.0202969	110.0426269
34.98258268	89.83470833
35.00271941	81.39893914
35.02296749	78.24521793
35.03752086	73.24474688
35.01262692	69.48466881
34.97270098	62.94559918
34.98627893	58.40355881
34.99091092	53.06844291
34.9909569	53.57845907
35.00872163	51.66371869
35.01133944	49.22572418
35.00357583	43.90044054
35.05692817	41.04884846
35.00679424	40.46438273
34.9696075	40.51323767
35.00730432	36.58377894
34.97706685	36.86136896
34.98966258	37.86534687
35.02619782	36.77441025

34.997151	30.96273918
35.00773443	31.96601042
35.03941175	179.1978121
35.01573032	169.29781
35.03729847	169.2893525
35.00173341	170.463708
35.00497087	166.1786381
35.01251857	160.6493597
34.99503975	154.5044651
34.97778055	142.2345203
34.9973658	134.2581616
35.09010411	125.1210148
35.00462486	111.4307968
34.98594858	105.7950532
35.03044928	94.22577391
35.00467795	94.5337943
34.98423555	95.27720674
34.96437316	97.98483254
34.97463343	87.84317221
35.0034353	85.33229338
35.00346563	85.53148107
34.98728375	82.85492226
35.0546494	82.11210282
34.99233226	72.13108737
34.99155139	71.51130333
34.99777271	70.18761215
34.98951489	69.99036103
34.99632626	72.00505858
35.02945271	69.08827052
35.03539822	71.87032587
35.01381663	70.84003008
35.02008679	71.86164393
34.9641868	70.78093548
34.97130116	69.78598944
34.97812165	116.5253466
35.03485731	115.5600261
35.04115135	112.3125924
34.96094074	109.6832094
35.01520681	106.5370631
34.9988398	102.7987551
35.02117324	100.2362361
34.98006951	94.4739358
34.97701102	89.99054303
35.00281672	85.44735787
34.98339122	80.61701204
35.0053366	75.31178536
35.01175675	72.3277744
35.01065895	70.72674493
34.97213644	67.51162672
35.00094632	63.88789671

35.01992506	62.46764137
35.00821137	59.92910577
34.99823011	58.58399056
35.04637087	56.42485294
35.00490397	57.1130369
35.0308555	56.40086782
34.99325789	56.83767386
35.00269385	56.48023359
34.98521267	56.05944694
34.96644862	56.58505837
34.96729048	56.68995484
35.02152195	56.85507372
35.05928405	57.20131268
34.99768835	57.24929493
35.05130788	57.252876
35.01828799	57.19358444
34.99284919	115.3644504
35.01684287	105.9879438
35.0007276	98.06707683
34.98344694	93.60822505
34.98992812	87.56455903
35.02671577	86.03290752
34.9968536	89.44029256
35.04801058	92.02340712
34.99945561	92.93747196
35.02428749	94.97385968
35.05276492	93.78760156
35.01522034	92.97346228
35.00423102	94.94793283
34.99419391	104.03139
35.06109038	114.1258473
34.99218504	117.2368021
35.05151674	119.8228213
35.00877783	119.1596675
35.05591163	117.3163761
34.98751657	114.8878023
35.0124555	111.7637241
35.01436349	108.1921784
34.99881349	112.0171826
34.98649255	117.3326688
35.0125658	113.1565217
34.98338983	113.8987338
35.05072582	113.9131654
34.97683643	112.7686068
35.00281672	112.4278241
34.97595177	109.0268342
34.9768082	116.6821016
34.9874359	109.6677893
35.00150603	108.9650247
34.99161266	114.7068742

35.04327352	131.8954489
35.02458265	139.0234449
34.96692982	127.906607
34.98647061	115.8068702
34.96256795	104.358911
35.02874919	104.3092424
35.04029175	100.2860862
34.97013619	85.11903109
34.9944196	80.05327181
35.04412231	78.73216127
35.01436576	83.13828585
35.01867213	90.58966032
35.03024148	97.48275372
35.02301422	96.89858139
34.98880177	89.97176839
35.00095436	81.15616822
35.00443256	87.28924644
34.97087748	84.3199871
35.00890681	79.52306836
34.98658575	66.55211779
35.00348292	59.52202233
35.00369833	60.75363031
34.98518227	59.83224263
35.01123122	54.40339139
35.0191988	61.2873231
34.99420951	58.42482889
35.01487156	61.30916862
35.01045942	63.20667614
34.9851302	62.72924648
35.01262692	60.34736482
34.97087748	195.6216956
34.97120572	175.5089999
35.00669089	172.1832511
34.98069426	187.3544028
35.02081761	188.8987183
35.02444542	191.3336698
35.00242619	192.6939922
34.96613639	183.3729638
34.97325906	164.5486659
35.01047857	141.0481759
35.06169155	129.3112976
35.03767292	149.8669858
35.01589089	155.8850231
35.06306113	168.7816453
34.99097442	176.6773048
35.00027261	175.7974572
35.00991207	171.6530292
34.96114058	168.4571201
35.01887328	159.4160938
34.99869746	149.4794212

34.99000693	144.960216
35.00920304	140.774819
34.98843844	140.7859002
34.98411985	133.1564909
34.99338615	127.9471531
35.03141899	126.1350794
35.01512408	124.0190132
35.01452668	122.7845752
34.97089505	122.0295202
34.9865372	118.6189648
35.02543533	114.0438786
35.03928956	108.1909623
35.02255828	228.9819174
34.99716242	221.791952
35.00998208	217.3185857
35.02271405	214.3021936
34.97824384	209.7874344
34.98118093	206.2198595
35.01689005	200.7377902
35.02356316	198.3021673
35.01334753	193.7995396
35.01405325	194.9657721
35.01282065	193.5894735
35.02874274	192.4457578
35.03578687	190.3012885
34.97808108	190.4045843
35.0044278	190.7165866
35.0296016	191.0545992
35.00210332	189.2506733
34.99853473	187.0378699
34.99314239	185.7609379
34.98355922	184.434813
35.01976546	183.5823724
34.98312057	184.5699075
35.04722311	183.6585449
35.01314392	182.7888917
34.97752786	182.9399555
35.04512148	183.7951723
34.9990325	182.5308657
35.00120912	180.5479836
35.00298362	179.79034
35.05589086	179.0623872
35.0524645	177.4925415
35.04622255	175.0650552
35.05305971	36.32774411
35.00784316	25.68087162
35.01443884	28.8385195
34.97890741	32.40314445
35.01393573	32.3894179
35.05278742	27.57509084

34.97401557	16.9788751
35.03756243	14.87745703
34.97903141	14.57424746
34.98012334	17.4285597
35.04446949	16.36771507
35.00521235	16.10285337
35.01001699	13.79423683
34.96447112	12.41523499
35.02255102	10.14438797
35.03698785	7.670569084
35.00922654	6.865409972
35.02995772	7.93608958
34.99600002	3.375943349
35.00274249	21.2656833
34.99465223	10.23928003
34.99475663	17.56837856
35.00109398	10.39931908
34.97632265	12.67496246
34.9997604	19.1919297
34.97313395	19.43936882
35.04057855	18.58540087
34.97966616	18.15916271
35.05317905	17.93284076
35.02107246	18.50137942
35.06210162	16.43571263
34.99933629	17.44722996
35.0059332	115.2197913
35.00876428	105.4014061
34.98909174	101.337208
34.98466197	102.665466
34.99202851	95.64490596
35.01559792	92.57707116
35.06023635	79.36253726
34.99440012	69.88586307
35.0194044	62.75008115
34.99911573	57.16181577
34.99871111	45.72293519
34.96840505	42.44189281
35.02825087	41.26406303
35.02993156	38.50938425
34.99902898	36.52671858
35.00330235	39.09445752
34.99619951	30.24836521
35.01312777	24.22784683
35.06041901	21.26010749
34.99626114	17.17814035
34.98943225	15.7559734
34.99464366	12.20015899
35.0583865	11.97572928
34.97673758	14.24121613

34.97718592	10.66108489
34.9819396	7.567660247
34.99643906	9.315107838
35.01330784	10.03011471
35.02326799	10.44339009
34.99333747	10.63287073
35.05496727	12.3837821
35.0519429	13.45134491
35.04215344	141.8130813
35.00229971	142.1418036
35.02477048	146.7330976
35.01856514	152.5714319
35.00771224	150.4365493
35.00384468	141.3563994
34.99104138	132.9042192
34.98006951	134.7108349
35.00209881	124.0410996
34.9907722	117.9185775
34.98716467	111.7848824
35.04944278	100.3978376
34.98298102	95.62001284
35.0312585	90.6558194
35.00890322	86.88642444
34.99678157	88.59315869
34.99507971	91.96295553
34.98127143	93.20891884
34.98823384	101.550245
35.05197272	95.30651978
34.972597	94.28024633
35.02418666	92.53457166
35.01850336	93.97291545
35.01155558	89.10833348
34.99353471	82.85182972
35.03843005	73.11069659
35.00941591	66.9442934
34.97262445	60.5817111
35.00862642	64.80396145
35.03801102	70.11153225
35.01526732	68.57466732
35.03579842	65.93545517
34.97673758	137.9879941
35.04321819	137.1438528
34.99389789	138.6288374
34.99250484	148.4256405
35.04918101	131.8881196
35.00395522	103.9009551
35.00359343	93.9568622
35.01486338	92.79363582
35.00735573	94.12498575
35.04121473	95.34418767

35.04533437	93.76759316
34.99476266	92.45047599
34.992495	93.74154887
34.9981074	92.07664008
34.98258268	88.38886184
34.98630613	84.46694278
34.99529596	78.79608455
34.98525086	74.45565816
35.0019351	72.00144773
34.99754077	68.99855412
34.9785532	67.22687598
35.03615683	63.82152803
34.99104705	63.37552712
34.99026314	66.88269979
35.0133498	67.57021539
34.99677557	69.21358422
34.99015941	66.01230316
35.02620382	65.19687972
35.03274999	67.25061649
35.01907516	68.21283318
34.98027573	65.62076904
34.98068664	55.96014578
34.98588317	199.7291201
34.98645111	193.8844756
35.01062474	192.1284473
34.99532359	195.4021818
35.0138504	196.6969569
35.00443256	177.356107
35.00415929	183.5630346
35.04237057	169.885792
35.0243022	176.6269137
35.01945491	172.3087517
34.99107749	147.3723922
35.01990391	141.0091615
34.97233185	133.1987616
35.04382047	135.3111067
35.02327345	124.2725533
34.99368183	117.4308417
34.98679547	109.1637511
34.9784567	101.0407084
34.98127609	104.8541319
35.0110628	92.99245414
35.04251855	87.2633701
35.04778591	78.20254576
35.000981	69.47825716
35.01365219	72.89713031
34.98990187	72.4807809
34.99612482	72.47437985
35.00094632	77.42585843
35.04169397	74.41533676

35.00854885	71.51771907
35.02826387	70.42676049
35.03683403	72.11383291
35.01154114	70.94379933
35.01535836	107.7291943
34.9916722	105.2024521
34.98846074	102.1193174
35.01227608	99.1967745
35.0021672	94.96522328
35.02363609	91.8757445
34.98590373	87.15097091
35.02800828	84.30551264
35.03772936	79.82418104
35.01714941	75.93709112
35.01808862	71.75154961
34.9874359	67.50948208
35.00377965	64.74250934
35.03545525	61.99445941
34.97907647	59.64776238
34.99079436	58.58496148
35.00789437	57.68626091
35.01089508	57.69620328
35.00458757	57.63168095
35.00776276	57.49095616
35.00418184	57.31857307
35.02131697	56.37316684
35.01015672	56.22082072
35.00591778	55.62451882
35.0256816	56.11751513
34.98366281	55.957708
34.97280795	54.86279911
34.99344092	54.65916219
35.03650936	53.81317438
35.00619996	52.97252389
34.9876712	51.53414009
35.05867207	49.56790272
34.98868729	64.86764332
35.02019185	70.45576662
34.98924111	66.95285417
34.99915527	62.59868836
34.98646058	58.35807004
35.07249663	42.75831426
34.97035131	36.42060078
34.99011456	37.57547588
35.0065718	41.5460849
34.97198766	42.78503135
34.9942397	40.09592332
34.97096446	41.11434084
34.98208715	43.91395271
34.99488367	40.16595918

35.00628303	38.65594092
34.9900758	42.47087205
34.99097442	41.40379661
35.017653	38.93455394
34.98751657	35.46123676
34.98863585	26.58903966
35.03718375	22.14723062
35.02704124	19.50154815
35.02899079	20.66081309
35.03766308	22.89320451
34.98738253	28.42467907
35.00719508	33.04611669
35.00272156	41.50163565
35.04408609	46.56575382
34.9867772	49.9372213
35.02861196	49.17359157
35.03759418	42.35008804
34.98170079	42.49213826
34.99824325	44.04844343
34.98413192	35.8377549
34.99706451	40.23554584
35.00865082	40.24201665
35.02986884	39.82960845
34.998525	40.29029056
34.98660849	35.17332266
34.99513407	34.1879945
35.00539949	32.71747899
35.005043	23.44799235
35.01013622	21.12002049
34.98703723	17.03168688
34.99590784	16.48447374
34.9804851	16.07045315
35.00939764	13.38649911
34.9913544	10.6966926
35.03802769	7.960831215
35.01139713	5.436012115
34.97716239	4.411895473
34.97138564	7.284672966
34.98197246	7.235987705
34.97547241	6.107127141
35.02230341	5.515908109
35.02570422	4.430322162
34.98660849	3.046696708
34.99636614	2.394713887
34.98859836	3.779579551
35.03797386	7.637168185
34.98674348	10.16803753
34.98863585	14.42241026
34.99351154	11.44639425
34.98834166	9.811880999

35.05468855	108.4680399
34.992343	111.5672751
35.01189424	108.3875633
34.99521121	104.3478345
35.03707928	99.89603379
34.98348733	94.65905687
34.98549441	93.10028331
35.01480581	81.01667546
35.01123122	73.114144
34.97264855	72.07625715
34.99250852	70.60317577
35.01690891	68.54030171
35.03481326	64.93685048
34.99552528	64.11501629
35.04939361	64.22174387
35.04246207	61.21815199
35.02417311	60.92873389
34.98561019	56.37201262
35.00024828	54.99060273
35.04946916	55.24109282
35.00024828	53.89237515
34.98555583	49.67180746
34.99890292	48.64142293
34.99111694	47.08229252
34.96100556	45.1789545
35.05921232	45.45557126
34.99206907	45.77055971
35.01413869	43.61388135
35.03291346	45.05466411
35.04826536	41.82738807
35.00332269	43.86961376
35.01766651	44.03488202
34.99117414	89.76266508
34.99368183	88.30978154
35.02859089	86.45354468
34.99185149	84.36660435
35.02761573	87.12469346
34.99739319	86.9253588
34.98155553	79.05787665
34.98123002	70.21590435
35.01362365	65.34900207
35.10406114	68.51953678
35.00080165	65.12977026
35.01640417	65.05838313
35.01696766	59.3902375
34.97373631	55.18517236
34.98975741	54.11312821
34.97693067	46.78921287
34.990712	50.06899012
35.03099085	38.41498341

34.97048911	38.3950547
35.02497865	35.56736231
35.00389086	42.8046647
34.98084127	38.11793568
35.00187796	37.70003046
35.05560256	36.26224554
35.0284191	35.57275977
34.99083957	33.3301203
35.01174732	33.41903478
35.00309094	30.86688945
34.99672322	30.13892355
35.08143678	22.44896493
35.00654288	19.0920393
34.99855856	20.09856132
34.97477807	83.04458495
35.03073758	79.39899855
35.01767144	82.09229498
35.01223683	85.89852289
34.99986144	86.88406436
34.98756474	79.78907583
34.99484833	76.48369968
34.97018727	75.37762498
34.97148481	78.36079452
35.0180174	70.05398185
35.02078469	67.99175749
35.02041325	65.41984408
34.98502835	59.62933734
35.01950719	61.36011698
34.96213974	63.94459487
35.0084099	63.87692409
34.98574788	64.09067645
34.9942397	61.59914005
34.98694554	58.06863209
34.97630596	62.61125404
34.99012601	56.15847518
34.9697673	57.79514881
34.97843618	52.35740797
34.96166645	56.55564609
35.03151994	61.27808496
35.00533548	69.40473547
35.00357547	62.87977764
34.99506867	62.32946773
34.98317145	58.72630062
35.04296921	60.74239555
35.03032725	61.87649707
35.01010816	59.72514726
35.00856634	114.144178
35.00056615	124.3667969
34.97335555	130.7501812
34.98878197	132.1914633

34.99368183	131.903792
34.99931371	112.9394145
34.99109306	99.93533433
35.00024779	104.9576717
34.99743132	110.9316374
35.00609279	96.05247604
35.01373528	87.18775523
34.99653362	76.48474667
35.01887328	66.82093417
34.97221632	66.94774854
35.01195456	64.82479145
35.01020723	69.71691275
34.98328484	71.95159885
35.00281713	70.33326633
35.0059332	67.61728539
35.00444581	69.65796153
34.98082043	74.53802338
35.00148323	80.35079298
34.96365352	78.13789925
35.00162599	78.54058366
35.02683642	78.39877053
35.02447098	79.27630398
34.99063583	79.13700126
34.96941868	75.99948659
35.06546652	77.33584075
34.99782582	80.42636732
34.96413806	63.17767661
35.01247165	62.24644557
34.99720111	77.04951965
35.04975351	74.42337056
34.99661288	80.54804268
34.98025284	79.14673123
35.00633673	78.80877339
35.05275578	92.4544156
35.00846387	107.1003222
34.99403887	107.5036285
34.99709429	108.3193467
34.98065865	109.852004
35.015748	110.9490045
35.00510056	112.0443824
34.99505903	112.4417479
35.01537922	112.8535064
34.990118	112.9249885
34.97864874	112.8582215
35.02624462	112.2168507
34.97763206	111.4547163
35.005043	113.2520449
35.01846015	117.6564627
34.95790799	124.6706622
35.0300066	138.690894

35.0284039	145.6083216
35.00341821	145.9807451
34.98241679	143.9954506
35.0053964	143.9593455
35.02271186	142.0592692
35.00539949	140.4207255
35.0064457	138.6994841
34.97313395	136.9036437
35.01684102	135.2737998
35.02533404	132.9089221
35.00105913	78.39356411
34.97824384	75.68957809
35.02607813	74.28110849
34.97623421	73.80077671
35.02070841	73.97023624
34.96683872	77.38994123
34.9953992	79.2060324
35.05923293	78.42492123
35.01122347	74.27775525
35.01946331	69.72801714
35.06435462	65.04439687
35.01761199	63.28511563
35.0233471	64.41017085
35.01258086	64.47296974
35.00475621	60.75154206
34.98227965	60.81811351
35.00899201	57.6560035
34.97225234	55.73656129
34.98082043	59.30724368
35.02512306	62.40947267
35.02482517	64.2524943
34.98737009	62.38332103
35.02213578	62.10429246
34.99891543	66.77587052
35.00105646	67.94758315
34.98701538	69.38708457
35.03507318	73.95010539
34.97987718	74.60927684
35.06653302	76.71146772
35.05290433	78.05299696
35.00900528	75.42324765
35.01175797	78.20094009
34.98951103	54.16580839
34.97882885	60.52427905
35.00825905	55.45020478
35.01122004	56.50008938
34.97121531	53.27756419
35.00148323	55.38293923
34.96784427	57.81274353
35.04054335	52.97645682

34.99536698	51.19519552
35.04653386	52.89503175
34.97843618	51.66675637
35.03105349	55.1630175
34.98828161	59.30879016
34.96306204	63.65026768
35.0180174	64.98407827
35.03551402	68.37828355
35.02292759	60.0719784
35.00459405	63.89003489
35.06683035	65.7940563
35.01028333	64.88561921
34.98617208	61.0980783
35.01278302	64.24085815
34.96897918	64.15786116
34.99984264	63.99723879
34.9807839	61.75550923
35.00983668	57.38320406
34.98933817	61.19202782
34.99650053	55.83887768
35.05192779	56.38942245
35.00283806	55.71048242
34.99097442	48.81482235
35.00538511	46.92181899
34.98940537	70.34318372
35.02383573	66.47508144
34.98327842	67.49286016
34.99588179	65.90629183
35.01389622	61.13890884
35.0188369	60.88766878
35.03865905	56.91009957
35.01454369	56.45696713
35.06464417	45.58400751
34.98640375	39.13670627
35.02908999	35.47291999
35.0226921	33.89460611
35.01141292	33.7168265
34.97754331	35.24882463
35.02959337	34.9357826
34.96128331	36.46858094
34.99840538	35.00023111
35.01299192	33.49559006
35.02897111	32.81677974
35.0065808	35.53423173
35.02792882	35.98449552
34.99420951	33.67801116
34.97753267	30.83288993
35.01047613	30.31689816
34.99120095	27.52211004
34.96758809	23.91478089

35.02802386	23.42806781
35.00193804	24.13256927
35.01075293	23.68232182
34.99270965	21.94406261
34.9804494	21.19999653
35.00604216	19.4699123
35.0083259	134.5475676
35.0060521	134.0258451
34.9879298	132.1772623
34.98447977	129.6024921
34.97418685	132.1439866
35.05400919	127.2963618
35.00828651	118.504858
34.99119286	110.1346849
34.99333747	111.6214672
35.00645044	110.1537193
35.05160885	109.3767343
35.039541	108.40546
35.00648948	105.9487945
35.05434798	106.2828259
35.04068061	95.96971463
35.00804728	90.81180999
35.00042372	91.80953375
34.99758321	90.62053242
34.996251	92.45817071
35.00331276	89.57317676
34.98117236	83.58047564
35.02774864	83.3838189
35.02191579	82.54542066
35.0092675	81.88620479
35.00172503	85.78839118
35.01887328	85.98178024
34.99667951	97.2650243
35.00546767	93.00570143
34.98675571	93.8558735
35.01667957	97.61452516
35.00955109	101.6601706
35.01991836	106.3754669
35.01905326	101.5954653
35.00489038	93.48306642
35.01303988	89.67534937
34.97979003	89.48607759
35.00094632	85.16394011
35.00318133	81.79886942
34.98789848	79.34238878
34.99812254	74.38817895
34.98987098	71.93370975
35.0284191	68.41543586
35.01590784	64.46628434
35.02060111	61.76916741

35.02896051	59.83681948
35.00825905	58.28010286
35.03885617	56.79107547
34.98334217	51.86302819
35.02648057	50.62307193
34.99810652	48.87223425
35.01281956	47.28767275
34.99146147	45.86229623
35.02742491	44.86104545
35.03395896	44.15039179
34.99354369	41.83940624
35.00747024	40.28337275
34.98227965	38.67084524
35.07973516	37.76062659
35.0106558	38.13705605
34.98901352	34.89804671
34.99368183	32.74594088
35.00940019	31.76827841
34.99795687	30.89919673
34.98629746	29.02715783
34.97993539	74.32963731
34.98474773	65.58657132
35.03067211	68.35578281
35.01123122	68.14067191
35.00177517	67.18230386
35.0403174	66.69901407
35.03019725	66.19961902
35.01357817	59.37572706
34.98485604	58.41926777
35.04041467	56.37608845
35.01229051	53.34327269
35.01684287	49.21879953
35.01615705	47.30710487
35.02065443	47.44592514
34.99921417	49.73843737
35.01408465	49.38300224
34.97892425	50.34575767
35.01918961	50.28572577
35.00458757	50.22720362
34.97255504	53.26718143
34.99821421	55.68523525
35.00424768	54.80258369
35.00976755	53.22707551
35.01602444	54.65980027
35.00668143	52.32389998
35.01457657	54.12963058
34.99576125	58.93105897
35.0074652	55.66811518
34.97627992	58.18953826
34.98929337	59.90740536

35.00387755	52.91350188
34.96724657	51.56323808
34.98653112	275.6475927
35.02429441	262.4309367
34.98047725	258.8116497
34.99431942	255.6403715
34.99122931	251.7732432
34.99111721	249.4585703
35.04570052	246.4945886
34.96886137	243.6310886
35.09487598	237.9772307
35.00066274	238.5297139
35.01433112	236.6560009
35.03163572	233.6314624
34.99740191	227.127448
34.98909174	225.4012038
34.99284478	219.3302054
34.99855976	213.397129
34.99126865	209.359153
35.03240855	205.3336179
35.03360073	202.4964167
34.98681478	199.0621912
34.99451446	199.6043796
35.04168591	197.5625029
35.0291073	193.9761198
35.02654954	190.8991992
35.02208519	189.6679165
34.99611297	187.2596515
35.01252828	183.6073896
35.03505399	181.9582341
35.00767628	182.4165592
34.97219011	181.6887103
35.02548421	179.2861901
35.0206968	176.9945484
34.99877769	373.0718752
35.03010665	369.8935538
35.03717838	359.6621481
34.98940537	341.9902943
34.98056193	342.5345026
35.01665392	351.2856585
35.00780129	350.8443705
35.01174053	355.2212638
35.01762948	343.7784637
35.01106204	326.8757516
35.00619996	315.5217312
34.9854994	309.0598868
35.04223105	298.4430224
35.02828804	305.0319849
35.04855405	299.0496536
34.98927442	289.1341613

35.07044767	264.7108682
34.99861482	244.261244
34.98785861	226.8115885
35.02131697	214.5385946
35.0259386	215.9326468
35.00278582	206.1826169
35.03162811	192.0726483
35.049812	166.5532405
34.9957163	127.1334789
34.9783683	126.5409367
35.118525	128.2344491
34.98272654	126.9462338
35.03729847	122.8413159
35.03867769	121.7454164
34.98966241	124.3787643
35.02759377	126.596113
35.02400684	344.1786649
35.00640271	336.1811949
34.9631389	321.1501975
35.0290822	325.2793205
35.03181328	331.0552716
35.00773443	323.7271993
34.99660847	318.4950104
35.03481731	320.5464067
35.00144441	323.0651409
35.01405325	318.2589436
34.99508801	301.479156
35.02762396	321.6561911
35.02432642	338.5955301
34.99250484	353.9021912
34.9765142	351.7175618
35.027042	346.6901529
34.98515105	352.9878431
35.00621709	353.944179
34.98401378	363.2885653
34.99817012	371.5651039
35.02142209	374.1597351
35.01797181	365.7734861
35.01318876	371.6158104
35.03034347	369.2761598
34.9677942	375.9673395
35.02021756	378.5156708
35.03386449	352.1894033
35.00475403	333.2516915
34.98084127	330.4071034
35.06437837	322.3394134
35.06958172	316.3984225
35.02544212	312.7024733
35.04755665	224.8285168
35.02089408	222.7468175

34.98980234	220.8537842
34.99977038	218.2272488
35.03397588	215.333103
34.99336865	212.136276
34.98228794	208.1163808
35.01226815	204.5308909
34.96291328	201.2247986
35.04788571	198.4972488
34.99625538	195.8073886
35.02236055	193.8612364
35.03682802	189.5617451
34.98053016	184.3435495
34.97440002	176.8644888
35.02636643	169.2003093
35.01766651	162.2326954
34.97866217	154.5949283
35.00329062	147.044977
34.98084127	141.3130221
35.00583634	134.6954193
34.98975741	128.5605878
34.97500674	124.3541637
35.03597654	123.8992458
35.02447098	122.3126939
34.97626956	124.5023573
34.97670866	125.0172365
34.98280804	124.4055382
35.01023404	121.3396585
34.97175645	120.8904734
35.01154114	112.2505676
34.98740215	111.1231338
34.9896182	214.6848714
35.00289343	213.4915057
35.02712698	216.2770541
35.00936595	218.2152168
35.00178545	215.8416804
35.01664348	216.9555686
35.00414345	216.913613
34.97272652	216.1660972
34.98317145	213.2820104
35.04353638	209.6257492
35.03179841	206.5755362
34.96159474	203.8395898
34.99664162	201.4521281
34.99336865	201.7997966
34.98246928	199.3849053
35.00628303	196.8367842
34.990118	194.5512822
34.98697332	196.9479491
34.9837304	198.8747365
35.01414784	197.1923684

35.04414699	193.9836219
34.99172661	189.3583314
35.00234593	185.6258317
34.99295758	181.2070663
35.01151537	176.9550014
35.01631909	177.9004958
35.03046121	174.9534446
34.96082834	173.9130803
35.012089	181.890929
35.05786488	194.5553514
35.01265174	221.145973
35.03168744	219.7992744
35.02015787	164.1370989
35.00294702	160.7947641
34.97092379	156.7595616
34.98274569	161.5283147
35.05545863	160.7066399
34.99711507	162.1747761
35.00489038	162.5952058
34.99991938	158.6437414
35.01115429	154.8703805
34.98689201	151.0319678
35.02070666	151.8889335
35.01023404	155.4381448
34.99581592	151.0697869
34.99606788	136.9249493
35.03326694	137.6574223
35.03941147	142.7628138
35.00960809	137.324821
34.97990974	112.8156249
35.00397382	98.8091918
35.01093612	93.00557738
35.00215717	88.45499563
34.96190916	85.59680501
35.01160744	83.00756345
35.02223205	79.79140396
34.9799959	75.99653624
35.06720938	70.41652797
34.98596707	67.35972998
34.97240169	64.34916697
34.97422527	60.21358279
34.9941885	59.63971372
34.99475663	62.09718214
34.99513407	63.2877064
35.00891068	163.3092002
34.99066559	126.5458885
34.99941619	104.828112
35.02065443	114.3401677
35.02271041	116.650033
35.02041325	139.3916146

34.96986143	129.3384087
35.00233966	155.6700029
35.0083689	138.8551417
34.98482189	146.6847244
35.03988787	132.7844047
35.00133473	123.3531644
34.98826823	134.2784049
34.99777271	124.2585945
35.00444753	120.6713465
35.0157795	123.3182923
34.97786954	122.6931465
35.06786284	109.0670865
35.03616681	125.693411
34.98355444	109.887092
34.95935636	92.77253576
34.97948064	80.77544598
35.00675193	80.12964056
34.97969985	73.34865783
34.81250705	68.11416981
34.81196754	78.03383348
34.821307	67.47778252
34.36784886	69.71359336
32.46742118	67.37318508
30.6601783	74.16086395
30.41728325	84.30616923
30.4881017	78.60813441
34.99132862	200.2806993
35.02309001	187.7974834
35.00170373	183.4192447
35.0074681	180.1449411
35.01115608	169.7612085
34.99788732	161.0893141
35.00532105	154.9349073
35.02738548	150.5968874
34.98883885	143.7059678
35.00036278	134.244403
34.98882994	125.9756144
35.00977696	116.3035973
35.01684287	100.9929353
34.99302654	90.71998124
34.99258471	84.54179442
35.01783802	82.25025297
35.00620559	80.48809299
34.98554711	79.33426659
35.02825755	78.39829935
34.98564485	77.51896747
35.03293212	75.85386043
34.99561779	76.91318266
34.98653112	76.57696724
35.02744976	76.2479183

34.99189847	74.27905043
35.01557178	71.23022264
34.98672788	66.10334732
35.00027261	61.67035228
35.02005684	58.71573672
35.0302815	57.66200623
34.98461171	57.34139187
35.0107897	57.08348505
34.97854554	127.09416
35.07115447	124.451684
35.00143754	116.738704
35.00929303	114.1856607
34.99720111	111.2852089
34.99361862	107.8103302
34.97689829	105.1278389
34.99175356	111.600669
35.01737511	121.764862
35.01442699	126.7659231
34.98461912	131.2030324
34.99678157	140.4951523
35.01830348	148.6582857
35.00226564	150.5891152
35.05631647	148.6632021
34.98281584	150.4845892
35.03512876	142.9882604
34.9802521	145.9406616
34.97524647	152.4279887
34.96908683	157.3916294
34.99920624	157.7475299
35.00173513	157.0249942
35.03170887	149.2907531
34.96613639	150.3632109
34.97629192	150.1969301
34.98588867	148.9265848
35.02257423	152.4192435
34.98090352	152.158898
34.96880325	151.1531748
34.98129303	148.818348
34.97294577	146.5570885
35.02520804	143.448351
34.97274045	261.2322033
35.00348292	261.3137303
35.06849998	260.6598313
34.98941644	262.1688316
34.97896885	258.1493874
34.98399587	255.4212362
35.01388947	252.636248
34.98360378	252.3630645
35.01774632	250.1131699
35.01041728	250.0073041

35.01278302	246.9650642
34.96657789	245.5900054
35.00570009	240.754245
35.03223145	241.2867822
35.0154652	241.1122649
35.02212793	241.0116181
35.03115892	240.3962929
34.98592489	240.6821609
35.01318876	237.7892466
35.02475595	235.4443891
34.9735629	234.0251056
35.02485993	232.6273675
35.03715353	231.0397379
35.03521603	231.7143865
34.9982369	232.2772375
35.00155206	234.4118829
35.012106	234.5714264
34.98279034	236.5781889
35.00245383	237.1794935
34.98596283	236.6282318
35.03555405	235.0514013
34.96800955	232.1840852
34.99552528	253.9975709
34.96898104	253.9659969
35.01531746	252.6276425
34.99452631	251.6190204
35.01402553	249.8598422
34.99840538	248.0097158
35.0193182	246.6187233
35.05372188	244.6619512
35.00865082	241.5093278
35.07226599	238.8602252
34.997562	238.0101467
34.98401176	238.236105
35.02774864	239.9850196
35.13495446	238.1383577
34.97488075	241.7270795
35.01174985	240.131701
35.02711869	238.829834
34.96872589	237.3652331
34.97786954	231.9242984
34.997334	226.8979344
35.01278302	222.2890007
35.00294703	215.6346002
35.02437604	198.3665705
34.9851138	183.8026271
34.95967122	174.0270951
35.04515583	171.6824444
35.00653991	162.7508064
35.02755821	159.0684339

35.04297374	154.2268212
35.0213424	152.0658575
35.08240371	148.3537248
35.00429052	153.6781989
34.97187728	266.1912928
35.04476829	263.10217
34.98217846	261.3787193
34.99931371	259.4219287
35.01489172	258.1545426
34.9770593	255.6405668
34.9975553	252.8878308
35.0475812	250.3273271
35.00858528	247.8116815
34.99950832	245.0115248
34.99506655	241.1058344
35.02539024	227.4267076
34.98970791	221.6845424
34.9697673	217.6794421
34.97137073	209.1169961
34.99079344	208.6626627
35.00268416	208.4036543
34.99379093	205.3970614
35.04262306	204.4529647
35.02706352	205.9227511
35.02131697	207.7739754
35.00281682	204.9957553
34.99026314	196.937203
34.99412666	190.5542771
34.98208715	187.3814564
35.00236076	193.829537
35.0340741	194.2348551
34.96082834	188.0029145
35.03919422	166.3666118
34.9757767	159.5143229
35.00304467	167.196523
34.99201367	144.2596879
34.98042299	349.9679925
35.03863081	352.375718
35.0277268	350.773291
34.99555611	353.8241887
35.02659228	295.1590406
35.00614907	242.4679601
35.00053649	284.7063629
35.0005977	343.3173175
35.02255431	340.4356334
34.97560672	338.3373727
34.99222162	329.5459707
35.02946149	335.1591032
35.01233128	334.3505181
35.01520312	321.5140931

35.00444011	306.2210326
35.01227874	295.2140902
35.02730086	278.9091347
34.99051406	262.8374245
34.99436781	240.3398905
35.01428795	219.8490269
34.99565232	195.0904821
35.04814569	163.6597017
35.01327943	139.603002
34.97605564	115.5743108
34.99395987	98.64315802
34.81786621	86.47993675
34.81985629	79.9245481
34.79763792	78.20080941
34.28296334	77.60676701
32.7533051	76.4356829
30.39998841	92.64832321
30.20484024	92.96014684
34.99375879	190.5271021
35.00527591	193.5500204
35.00768806	196.0893137
35.00160185	198.2294582
34.97406182	199.5740531
34.99288044	200.8568599
35.02292759	201.6971494
35.05417184	202.5462138
35.02742491	202.9445824
35.01204707	203.1008299
35.00427623	201.37716
34.9993687	200.531349
35.04790852	198.7561945
35.00170373	199.0233948
35.01709666	200.1032955
34.9971625	204.7086441
35.017126	206.6817476
35.00762725	205.0344091
34.99936755	202.1440671
35.02886764	197.8024867
35.02757628	192.5283943
35.01278302	185.5634615
35.00197803	180.3628226
34.99614339	174.9286551
35.02197786	166.9479465
35.00889024	168.3509637
35.05148875	163.5648225
35.02153841	162.6563514
34.99142686	158.281148
35.01175292	154.5300946
34.86884855	152.4402398
34.87533799	151.0259771

34.98443772	335.8173107
35.04702454	350.2328532
35.0081729	375.2642357
34.98930269	364.6634809
35.00066274	364.8191023
34.97689894	366.3437181
34.99611297	370.3501069
35.00894329	370.8134368
34.99871111	370.0023889
35.00393003	367.217874
34.99892777	372.003071
35.00018414	363.5167647
35.01629735	368.5701339
34.97784503	344.4837164
34.99714694	337.7432919
35.03240855	329.5532071
34.99083957	326.8017464
35.03336802	330.027232
34.98951489	343.5232482
35.01113562	334.4923377
35.03298104	331.65838
35.00329579	330.4784683
35.00132363	324.0805959
35.07938043	323.6316166
34.99596582	325.2242696
34.98275521	322.0557747
34.992969	322.1483586
35.03059796	323.3032063
35.01233759	323.5755169
35.02029131	322.8541225
34.96319767	320.9228708
34.98140831	318.0257474
34.98594323	183.2741519
35.00166086	194.0452727
34.99251238	194.4717835
35.0279806	195.4610516
34.96526755	192.1988078
35.04628753	201.5598994
34.99611313	200.4702875
35.00320247	192.9904889
35.00583634	192.8640342
35.00702245	190.6931111
35.00131355	188.9594428
35.02219859	187.3132917
35.00747748	183.8775412
34.97168728	182.3213624
35.02281642	177.7204137
35.02350542	173.4600875
34.99354369	169.3400227
34.96097942	165.5993027

34.98837304	162.272065
34.99704984	159.3371652
34.974985	156.4439498
34.99574342	150.3446877
34.99821421	147.9029811
35.02858581	145.3351246
35.02232124	144.4918253
35.01373528	141.9389072
34.96100556	140.7279629
34.99677936	137.6149041
35.02065443	134.5870714
35.05828587	131.150566
35.00696327	128.9332535
34.99882057	128.5898382
34.98846074	169.6575532
35.09290167	159.7771335
35.04262306	155.6222353
34.98695566	154.2470944
35.02374129	153.2963319
35.017126	150.9682373
35.01049908	147.2473763
35.04182126	142.7208658
35.00853573	136.0181641
35.00852041	128.8746815
35.01242963	120.0748742
35.02362667	114.1357523
35.00569553	108.8890117
34.98133143	104.9722884
35.05178374	101.2825524
35.04282668	97.94295785
35.01062516	95.44903242
35.00591304	95.19613254
35.02765075	97.51296531
35.04315362	103.6363661
34.99183085	100.5026718
34.97983084	98.55276813
35.0357122	98.57393752
35.01195791	101.1247739
34.99600816	105.3403815
35.01192878	109.5224227
34.96441369	110.0990076
34.99874017	105.050471
34.99826679	99.46296151
34.9957802	101.5318429
35.00492261	102.4758409
34.99660211	98.38018611
35.00020767	231.9187571
35.03542369	229.8224406
35.00177003	201.3838146
34.99891396	192.0959362

35.03627191	187.4840333
35.03646482	173.7751438
34.97555087	167.6609995
35.03298788	170.9472369
34.98679547	190.9723429
34.99361862	204.8784708
34.99855976	223.7439236
34.98956682	232.4007773
34.99857421	202.6442089
35.00730417	187.1690567
34.99873294	196.5840635
34.96299616	205.9475021
35.01972224	218.8789269
34.82350841	259.0599014
34.85780652	278.847579
34.28246741	269.8567931
33.45731944	227.2553647
33.34053975	221.6054106
33.43148635	208.8775679
32.4629185	221.6869612
32.55716779	238.0991315
32.47801714	247.6445987
32.50105382	258.3537983
31.57116956	244.0849817
30.11731552	231.1700883
30.32373005	214.9196593
30.20638326	147.589418
30.36030082	137.3479789
34.99361862	285.3582291
34.98811946	282.0532245
35.03080428	279.972215
35.04627022	277.884368
35.0110628	276.1077633
35.01449873	273.4848127
35.01072979	270.5889937
35.02225969	267.9164943
34.97315791	264.4332258
34.98594323	263.3283846
34.99410174	260.9829976
35.01037013	256.9251488
34.99376205	252.1835513
35.00081343	247.4307782
34.97944363	244.770047
35.02131697	240.1891876
35.01000259	235.9833162
34.98679547	231.5262761
35.02828804	229.8006864
34.98740215	226.7538074
34.97423892	221.6862063
35.05094374	222.8118735

35.01383499	223.5397228
34.99987947	221.2106556
35.05648628	215.4788274
35.02146168	219.6551469
34.99483702	216.6510157
34.9836824	216.0654299
35.02039012	216.8344986
34.99689329	223.0314002
35.00821137	226.5613246
34.98590373	228.5537584
41.96914673	319.4231709
39.13128983	320.3053459
35.78729659	322.4402428
34.99667224	324.3453743
37.76141206	323.7688353
34.99005945	327.2691102
34.97500674	327.8463358
37.02911183	325.8738702
36.25941185	326.0920382
38.18537426	323.4044664
38.47204155	323.2050285
35.5618797	324.3033027
35.00247786	313.310699
35.04677738	306.2191144
34.98167623	301.0238708
34.9976035	295.5623701
34.99498992	290.0259258
34.99795687	284.2232197
35.00085956	279.4884302
34.98389131	274.2769324
34.9946271	270.0647655
34.99376205	268.3874658
34.97843618	263.7540787
34.98747151	263.1470037
35.01550548	262.8742035
35.01905986	260.5839538
34.98455786	256.6656709
35.01700929	252.8906135
34.99955838	251.9349786
34.98990745	251.2177598
35.03576988	247.1446486
34.97366093	245.0611041
34.99288044	84.13125445
35.03759986	76.58025548
34.96431973	76.46716376
34.98630169	75.4945902
35.03665259	74.35835949
35.01558519	76.6898885
34.99099149	75.5112201
34.99464876	74.45813759

34.98293069	73.28492325
35.01466044	76.89022599
35.01256695	78.8153072
35.02130689	78.12073012
35.02860841	80.01025525
35.00976755	80.21122805
35.0143385	78.27164424
34.99104705	78.82587477
35.01174053	81.97888044
34.99364283	82.56207287
34.9818136	79.81864574
35.04454508	78.65541853
34.99376643	79.77745964
34.99743724	71.07569015
34.9709357	66.45920259
34.992969	66.19058009
34.99354369	71.69684529
34.98687405	70.0755275
35.01156118	70.38953695
35.02676129	74.35786554
35.03682802	74.31365575
35.0024987	73.7521904
35.0458428	73.0258675
34.99199882	72.51067555
34.97650138	71.75258307
34.97029124	71.00376593
35.02152195	70.68780883
34.97990911	71.21867903
35.02380942	71.21161655
35.00056615	71.69756453
35.02122832	69.8033452
35.03027161	70.92347176
34.96082834	70.48284041
34.99256778	69.2199241
34.98907633	71.25902228
34.99216877	70.9736221
35.0340285	70.94854539
34.98331748	72.28666982
34.97876246	72.74342405
35.04310327	73.33751316
34.96197321	73.31451832
35.03679572	73.46638991
35.03285851	73.39362565
35.03055758	73.89239724
35.00621036	74.19584719
34.97500674	73.52933912
34.99732359	75.80599501
35.02162396	75.15455866
35.02233441	77.37226848
35.04092474	76.1307556

34.99682191	76.22213772
35.03908153	75.30465681
34.99529596	75.80624585
35.01487117	75.95532523
35.0284577	76.18139686
34.99525213	75.9798125
35.00489038	102.8664168
34.9849826	102.7953746
34.99527332	104.7323352
35.04925993	103.2720919
35.0196269	104.2179999
35.00163228	108.5323054
35.02271405	108.2587868
35.05442221	107.4275007
35.00114877	107.2651053
34.99579198	99.24517124
34.98750663	97.27494613
35.00919246	98.41343299
34.98867356	98.32952595
35.00712488	99.83558764
35.01877147	98.32211041
35.0206968	97.62119146
34.99483702	101.5995774
34.96941868	107.0235199
35.01120886	108.7987131
35.01626614	106.4993176
35.04037601	108.1720972
35.04975204	111.9350206
34.98102479	116.3554958
34.99943271	124.0636264
35.02440652	132.2628482
34.96713555	124.6336096
34.98890065	122.6748492
35.01397521	117.9184097
35.00573275	118.7836162
34.97894911	118.801398
35.03017644	117.3524413
35.03362114	116.1330292
35.02636643	95.08464549
35.07626164	93.56745173
35.03368716	91.45301898
35.00015239	91.91294638
35.01615169	90.8555093
34.97094632	89.73053299
34.978667	87.69941501
34.96256795	86.91097424
35.02137382	86.26170425
34.98520631	85.57340501
34.97481773	85.17682724
35.04892971	83.67819373

34.98169384	83.28488959
35.01799997	83.41321293
34.99581988	84.37804526
34.99137996	85.69156358
34.99250852	87.53873404
35.01130149	90.24474896
35.00075237	92.50525424
34.97934451	94.9620741
34.99376205	97.20883321
35.0126437	100.2891685
34.99822237	105.4117482
35.03826029	105.4791512
35.00904786	107.9059523
34.98348733	105.7988118
35.01016291	105.4821929
35.03171261	100.8988198
34.9826711	101.4069214
35.00408925	101.2313438
35.03633493	101.5674923
34.99568585	101.5324094
35.00150603	105.7045329
35.03031791	107.4234243
35.05200284	110.5313508
35.02386909	115.1394955
35.05806918	120.1327505
34.98974676	125.541245
34.98893932	128.1986017
34.99513407	131.0246973
34.98169722	132.1665761
35.03179841	133.4240572
34.98553765	134.622557
34.98228794	135.381785
35.00395184	136.1451306
35.01077691	137.2252718
35.0163606	137.0387935
35.01439014	136.7624002
35.0114627	136.4396107
34.99185718	136.350199
34.99199882	137.6213605
35.03541114	139.1547225
35.02107246	140.7513382
34.99750464	143.282247
35.03000737	148.3631027
35.01062576	152.2786668
34.98416985	155.4555821
35.06508927	153.9077842
34.96042689	155.4763786
35.00956347	151.4461996
34.98794656	150.3309005
35.02015847	149.8024028

35.01451065	149.4002312
35.01716553	148.4301924
35.01378926	91.90144664
35.01209794	91.58486118
34.99490197	91.53320852
34.98759076	92.82319049
35.00664676	93.90152338
34.98507357	93.69591866
34.96609799	91.7935071
35.03364643	91.01788382
35.00849732	92.4057833
34.9735629	93.07016145
34.99927811	92.83178837
34.97234786	90.47388644
35.01028635	88.60230522
35.10269432	88.11099108
35.01083353	89.41623803
34.98595835	86.80462744
35.02349462	86.85542731
35.04476829	85.52864906
35.022284	86.77215665
35.01857979	86.08141528
34.99104705	85.27445821
35.0019351	84.83913544
35.01953569	84.3868202
34.97987718	83.42929027
34.99597551	80.55626677
35.0018891	81.40428993
34.96636167	82.43095575
35.02000537	83.80409698
35.00066274	86.01434301
34.96630535	88.45132168
34.99677795	90.39458349
35.01639099	90.46534984
35.03785701	130.9252665
35.02332748	131.7119707
34.97808108	130.6175221
35.0450796	132.9099505
34.99892777	134.7705672
35.0482856	135.0638663
35.0024531	136.9140307
35.00386028	136.4985209
34.97913279	137.3550689
34.985588	137.8349753
34.97274045	136.5088061
34.99522753	134.8875372
34.98836597	134.2197963
35.03447297	132.4069116
34.99192644	130.8357569
35.07580486	129.0861811

34.98679547	128.3725057
34.99146147	126.6273945
35.01234624	125.3203833
34.98469366	123.6730499
35.0364392	124.7131628
35.02448874	126.0407348
35.06390856	124.2935153
34.97657857	121.7707202
35.05205747	118.5500178
34.98157438	120.9514076
34.98567593	118.6235081
35.00332269	117.5971102
35.04829276	118.3694071
35.02215619	118.1795772
35.01265174	116.1343908
34.98303488	115.2819245
34.99647024	151.730576
34.97934451	151.5711168
34.98703723	152.0706993
34.98050228	154.4358985
34.98644215	159.619822
35.00226564	162.3937057
35.04232565	165.1946486
35.03956043	166.0850104
35.01764308	161.947451
34.99517642	160.112265
35.00024828	156.2587708
35.01828998	148.8488517
35.00991134	143.5554531
34.98951489	142.1232277
34.96788151	139.8268231
34.99588989	136.7262512
34.9818082	134.6334742
34.98868688	134.0686108
34.97647338	133.1394244
35.00287191	129.3610596
35.01408465	127.5702537
35.04636173	125.7812431
34.98650458	125.5518594
34.9767833	127.0073707
34.9965653	126.1797705
35.04834191	127.820434
34.97517687	125.682916
35.00016924	124.2061495
35.01306198	124.8411768
35.00309094	124.907433
34.96863429	124.9113756
35.00799454	123.9372309
35.01411048	95.61202876
34.99907396	94.70143867

34.99600002	93.3355113
34.99452738	91.35450245
35.00668143	91.02501127
34.97359925	91.53213889
34.96660111	93.6817168
35.05210438	89.1286173
34.98859836	88.06750886
34.99345226	86.16345455
35.01635377	85.34459163
35.01487117	83.82208572
35.00761571	80.96732985
34.97683643	81.03625537
34.99308379	78.91102604
34.96522806	77.0943641
35.00018957	76.58739318
35.04786515	76.4000802
34.9755031	78.03967014
35.00197048	76.91305083
34.97972373	75.94877506
34.96213974	75.48462352
35.0205669	74.84107671
34.99798656	72.9441065
35.01397521	72.11521084
34.97644605	73.93837304
35.04046181	72.88381967
35.0078676	72.13733489
34.9927413	73.3553834
35.00072508	74.21175515
35.08581788	75.91978957
34.98025962	77.33539301
35.02374164	124.2661198
35.01740658	122.042253
34.99217698	119.0879245
35.01684287	118.5690961
35.04975204	118.9223984
35.03013431	120.4617379
35.02213578	122.4361379
34.97089505	116.1744922
35.00966755	114.0488124
35.02238356	112.3620417
35.08752031	110.1234388
35.00064517	105.2798332
34.99823011	100.9282344
35.0282438	100.5497136
34.99687522	99.77941189
35.00016494	96.8864588
35.0229501	96.02900727
35.02603579	94.14326146
34.98855891	94.58295164
35.03870968	93.90713898

35.05652795	94.29394029
35.02258009	95.90241885
34.96114058	93.36116167
35.04795196	90.00196348
35.00961691	88.31691666
35.07001699	97.04509659
34.98461912	101.2148962
35.02329189	100.2033037
35.01820074	102.8836818
34.99968183	106.2242981
34.98303488	107.0162279
35.07853599	106.7261068
35.00983668	100.256492
35.0384993	98.85891636
35.03078637	98.40268463
35.01142439	98.6157162
34.98478584	101.3576058
35.0277268	104.0623134
34.99124508	106.2024464
34.99363166	105.2382634
34.98596707	105.8729134
35.00024779	106.885618
34.99259194	109.0183864
34.99059614	109.1677726
35.03108603	106.4916252
35.01035648	106.982487
34.99787858	106.0673705
34.99304817	102.286204
35.0499744	101.0871613
35.01491685	99.44496731
35.02085537	100.1758622
35.01358242	97.91419429
34.9757522	94.93035302
35.0260534	94.39567883
34.98683522	92.4371915
34.98470899	92.22514041
35.03157825	90.20671805
34.9661002	94.02720658
35.0228773	93.37370074
35.01769629	91.96511419
35.02102683	93.19823012
35.01987051	93.535129
34.98229291	93.3481443
35.01252934	91.75861916
35.02763979	69.9126336
35.00070312	71.96985102
34.9921249	73.94043989
34.99555281	76.1146197
35.02214292	77.82631435
34.97396802	79.43954597

35.03288991	80.51284983
35.0166653	79.93984417
34.96319767	78.93352794
34.97296228	77.04915906
35.01251084	74.93068681
35.00824093	70.60266451
34.98461171	67.44453073
35.0125658	64.16682074
34.9958103	62.82750615
34.99573684	60.49205846
35.03417978	57.94399052
35.00541587	54.11187012
35.0001638	51.51517899
34.99444235	51.16286824
34.98263229	50.68816334
35.0148166	51.12908641
35.01337571	51.29276821
35.0064457	50.16747538
35.04974926	49.98031089
35.03598648	50.3492368
35.01404801	50.34855613
34.98956272	49.53608448
34.97001546	49.97420954
35.01348087	50.26124789
34.97582235	50.21744965
34.99630025	50.80055636
35.00319608	42.25240152
35.00501113	42.75384264
34.98937411	43.09239363
34.97240169	43.45489965
35.01651751	44.08946435
34.98561848	44.22551212
34.9824896	44.58896644
34.99314239	44.92867943
34.98939004	44.362495
35.0424171	42.81505946
35.05216442	42.60373096
35.05097356	41.2504286
35.00113037	40.51991085
34.99691711	39.07344194
34.9653678	36.62561803
34.98474773	36.8928033
35.05548287	35.14099484
35.02084062	36.17579418
35.05505474	36.39864518
35.01900394	34.67129172
35.02993871	33.91510041
34.97673758	34.38891179
34.99517251	33.99020614
35.03517351	33.9408911

35.00131355	35.56905632
34.99063392	36.09726761
34.99175915	36.15585252
35.00310546	35.97527222
34.98561019	35.77633482
34.98827639	36.54244474
34.99346097	37.5936615
35.04897971	38.11443423
34.99517095	34.59003197
35.04727963	35.28702086
34.9824896	36.69888615
34.9907526	38.96231488
35.01300416	39.13563437
34.99977038	41.42365543
34.97973166	42.67204633
34.97040337	43.57073406
35.01435943	46.03052509
35.0222004	47.57457631
35.01341228	49.10649445
34.9709357	49.95204465
34.98687995	50.88352357
34.99977038	50.81323558
35.01852893	52.45148279
35.00049895	53.05971704
35.01014263	53.00498518
35.02807929	53.26663062
35.05045343	53.13010587
34.96638215	53.02684732
35.00487528	53.26709222
35.02039195	53.94739222
35.02949211	54.00080627
35.01517354	53.81890846
34.96250307	52.55563123
34.99179864	54.37360099
34.99950759	54.76291742
34.98754872	55.6595478
34.97533554	55.46418045
34.98627256	55.96585444
35.00487454	56.03296806
35.00922855	56.48555821
35.04953395	48.01549423
34.97978079	48.10976372
35.01544821	46.91070058
34.99878931	46.60350049
34.9696075	48.1827065
35.05064822	51.07271573
35.00494455	51.82693139
35.02784716	52.47387939
34.97438806	53.19231588
34.96513722	53.91659934

35.00710883	54.45127208
34.99355084	54.03908741
35.0350417	54.27781824
35.04566408	54.35340221
35.01390356	54.44249304
35.00047131	54.55243052
35.00845371	54.50899764
35.02566124	54.59166176
35.0210759	55.13564718
34.99448228	55.69226178
35.00747748	55.93396333
35.04521733	55.44985668
34.99249945	55.59583858
34.99839925	55.59652052
35.01337844	52.95270708
34.96696266	54.71398154
35.03488293	53.95693402
35.00132363	55.1862834
35.00439021	55.24782684
35.06468482	55.54504836
34.98683522	55.77416481
35.07265111	55.87555098
65.61071184	15.65806907
65.61772378	14.78304237
65.60223524	13.90308671
65.6009488	13.04139474
65.62642503	12.41825768
65.60721171	12.39722745
65.57253769	11.49617247
65.61694483	10.74448501
65.56894547	9.627863995
65.57762763	8.433937129
41.22349951	31.31553893
40.08558737	30.49860796
35.07224239	32.8630576
35.01927326	31.18611281
34.99525213	30.43745976
35.00934661	29.70277669
35.06079058	28.97212131
34.99448228	27.80789753
34.99220429	26.80049613
34.9768082	25.81178751
34.98298102	25.09341527
35.00377965	24.1513074
34.99855856	22.60491028
35.00955109	21.23275935
35.01815595	19.56477249
34.98191641	18.98632357
34.99731467	18.6182464
34.97001546	19.26664463

34.99973619	19.51333443
35.00406294	19.85621007
34.99580001	20.42173732
35.01809625	20.79391961
34.99117414	4.822899133
35.00056819	5.409737356
35.02957846	6.096469624
34.98554651	6.653590405
35.0133417	7.105165156
35.01487117	7.673999455
34.97627992	8.529946877
34.98648125	9.865895678
35.00894996	11.39762419
35.02948705	12.7175611
34.99368183	14.84729807
34.98651741	16.37233077
34.99556382	18.65946759
34.98281584	20.54441134
34.9671943	22.28333604
35.00082472	23.08191071
35.04013333	23.83924199
34.97819652	24.95727567
35.01138069	26.32035669
35.00195017	27.62537066
35.04865396	28.50090177
35.03188302	29.54698779
34.99397719	29.60520124
35.00735301	28.95825542
34.99706451	28.52992271
34.96847055	28.81507761
34.98290055	28.24554991
35.01651798	27.61291232
35.02216474	27.21812495
35.04050892	26.62520802
35.00215717	26.77942592
35.01309347	26.48609581
35.01445674	47.41658142
34.99798563	50.51870534
34.99891396	54.95816548
35.05000903	60.88151604
35.06211311	58.78305045
35.05262157	59.5662256
34.97419578	60.9191629
34.9851302	62.15960633
34.99513407	64.21733677
34.99561074	63.37072409
35.02354	64.37258894
35.01384548	64.65746316
35.05413653	66.04285998
34.9867772	67.15979257

35.0059332	66.69333332
35.01559792	65.17306429
34.98116117	63.73323444
35.03080428	62.24617824
34.98218862	62.721786
35.00350764	63.02191184
34.99250484	62.83195003
35.00627756	62.60603111
34.99805244	61.56335893
34.9987867	61.84267595
34.97593525	62.19217004
34.97378582	63.2533531
35.03383054	63.47588964
34.99434535	63.23131233
35.01217672	63.80655061
35.04306581	63.98697885
35.04128433	64.49550108
35.02173303	65.00321861
35.01945922	34.56158165
35.02435324	35.50299864
34.96526755	37.33419993
35.04249866	38.91709395
35.0201942	41.80071206
34.99272227	44.49851096
34.97947444	47.30350091
35.01448483	49.9906274
34.98342048	53.28022802
35.00062681	54.61548034
34.99837396	55.72744876
34.9836824	55.02904464
35.02373384	55.9609815
35.05275158	56.81025008
35.03864609	58.50242241
35.02178676	56.47927184
35.06268406	55.39754401
34.99464366	54.46897256
34.98859845	54.89264287
35.0025821	54.80025153
35.00211555	54.39168343
34.97422582	53.39008992
35.00232357	51.41603737
34.99955525	49.89796086
34.97887524	49.34306707
34.99426036	49.866995
35.02945939	49.73361803
34.99259194	49.15382683
35.04070425	50.04852937
34.97378582	50.12528129
35.00429916	50.22647137
34.99308531	49.27457826

35.04256324	193.1338749
35.00708578	173.9531478
35.02836104	173.2055074
35.03478796	179.0633408
34.96683872	175.8534025
35.00426793	171.0690834
35.01074233	170.0318151
35.04594793	173.9696248
35.05380789	161.2842563
34.99250852	156.6240501
35.02957846	147.5802092
34.98731701	143.7857101
35.00676583	142.2066639
34.98566114	141.9653315
35.00904786	139.4192907
34.98789725	126.9673039
35.03465363	111.7596828
35.0109211	97.76433458
35.02477048	90.81734269
35.04808528	85.91958788
34.98742926	78.9915136
35.00133473	72.4452949
35.01186957	71.60666531
34.97671193	69.1883053
35.00517766	71.03349673
35.03278045	67.33652926
35.06347994	63.74106139
34.99037695	63.18519863
35.00934661	69.00929106
34.98841705	63.54002277
34.99660847	58.28069326
35.0331318	62.45364857
34.95614477	0
34.9693337	0.016785918
35.01620655	0.217822027
35.05215744	0.387408449
34.97480287	0.523668021
34.99192644	0.750131671
35.01164257	1.332437531
35.00700153	2.155222413
34.9778796	2.783830399
35.02740079	3.47878527
34.96957808	4.882485922
35.00344239	4.83682131
35.01308018	5.953273989
34.98667894	6.490565731
35.03553524	7.584653605
35.01147831	9.049366216
35.00245383	10.08049965
35.05761277	10.80539086

34.97052484	12.25577821
35.00093739	13.6684187
35.00456614	15.40083045
35.03263696	16.37713071
35.02530699	17.02579261
35.00377611	17.49637572
35.0669208	17.18675749
35.0291999	17.33976809
34.9986113	16.80273903
35.02168158	16.34615195
35.0193182	15.8441189
34.99840538	15.49452703
35.00188169	15.08603577
34.9985254	14.06368915
35.05812084	62.11251019
35.00209881	64.69487755
34.99092728	67.55030823
34.99465838	70.22231309
35.04453554	71.54563488
34.99316029	72.90860266
34.97244104	72.44269045
34.98182401	72.43254867
35.02556949	71.08403884
34.99291559	67.07318263
35.01268565	62.03107377
34.99985999	55.44127997
35.01677956	50.03498767
34.97290367	44.81654758
35.02533404	40.57584897
34.98651741	36.88356993
35.00232603	34.03826552
34.98588317	31.45698429
35.00569553	29.44169467
35.02271405	27.55232449
34.98100622	26.76703031
35.00693346	26.48356279
35.00252394	26.71307333
34.99545693	26.21020886
35.01951651	26.24654166
35.08046456	26.60571622
35.11337895	26.50164319
35.00292357	26.46201511
34.985589	27.48762609
35.02542703	27.9918624
34.96769742	29.28784985
35.00821215	30.08212643
35.01406536	99.11819858
35.02653802	97.7469209
35.00901842	98.10421426
34.98390061	97.54154539

34.98987098	97.59634392
35.0188369	94.99178293
35.03956043	93.06704096
34.99036757	89.84886924
35.01827218	86.79745485
35.03428772	82.99119752
34.99092728	77.83397435
34.99292561	75.55931313
35.08439412	76.41036183
35.02448874	78.09270358
34.99618596	76.35307762
34.99387403	77.42629196
34.98560416	75.67537104
34.97905763	73.63705792
34.98653112	73.79742806
34.98320163	73.15207484
35.04633855	67.9214901
34.96819037	65.32966081
34.9809424	66.57394823
35.02459756	64.61084833
35.01907681	62.62799716
35.02306803	62.34728938
35.00012458	64.35858045
35.03463054	65.44123672
35.00094632	65.66243966
35.01623319	64.72186702
35.01015672	49.29988625
35.03213487	52.57029168
66.16633009	0
65.58456989	0
65.5588878	0
65.59375667	0.121750734
65.60556395	0.865907472
65.62686705	1.846755101
65.58489596	2.926137668
65.58434097	4.349674779
65.56964131	5.941648471
65.60523781	7.05740231
65.6150204	8.046216991
65.57657061	7.92860843
65.59847312	8.076654705
65.59268014	7.863120089
65.61365224	7.911400478
65.57402996	7.961280427
65.61410316	7.969497875
65.58703832	7.836996436
65.62509045	7.956319424
65.6135811	8.142415023
65.59754082	7.757301153
65.58192691	8.006467568

65.57519007	7.156716363
65.5796039	6.153961815
65.56667025	5.44036465
65.57139613	5.353239367
65.60516412	5.287663551
65.60250529	5.835770499
65.6385498	5.577483444
65.64443078	5.520034283
65.57951777	4.65363596
65.58103346	2.903533782
35.06754129	31.81334943
35.02435368	33.78120401
34.9734759	36.32898648
34.99939173	38.8705327
35.00289634	41.77550372
35.01686817	44.20998666
34.99970302	45.23953007
35.02269238	46.37299769
35.06320248	47.69361298
35.00023108	47.79782658
35.02913709	47.38866474
34.99407263	47.7876532
35.01773887	48.60516835
35.03447756	49.85947383
35.02185089	51.34655697
34.9639643	52.86885504
35.01774632	54.01978084
35.00318977	54.66075711
34.98589893	54.92155115
34.9929902	55.2613091
35.07405718	55.81115347
34.99137996	56.32788106
35.02387962	56.65234252
35.00702245	57.24966966
35.00377105	58.27626103
34.99378188	60.58653396
35.07696709	61.91666814
34.96114058	61.12887481
34.99666244	61.19748184
35.00893928	61.19141953
34.97754058	61.53445278
34.99614563	61.28022745
35.02838062	35.69353373
34.98554651	38.61149929
35.02305141	40.81453409
34.99599049	43.83033507
34.98742926	47.02890071
34.99561074	49.74180639
34.99118708	52.19648552
34.99152821	54.17776702

34.98799222	57.06567245
34.97422582	59.44592486
34.98680985	62.13310915
35.03756243	62.39899281
35.00153177	62.38921929
34.99172954	60.60102377
34.99139294	58.72509269
35.01808862	56.13387189
35.01316392	53.48059611
35.05277787	50.34065991
35.00539949	47.58344602
35.07239633	44.85631325
34.99444235	42.77576243
34.99561074	41.06114666
35.01709377	39.69049
34.99818056	38.58506501
34.99882057	37.28299061
35.00187796	37.79992685
34.98304458	37.33727674
34.99483702	35.29250902
34.99922229	35.09454724
34.98474773	34.99580435
34.98281201	35.81693088
35.03148553	35.99847331
34.9987867	27.20335863
34.98136338	29.45580372
34.96581408	31.64112278
34.96403289	33.6453533
35.03100576	35.80357999
35.01045942	37.95402646
35.06206956	39.39678902
35.02255214	40.41315541
35.00724065	41.31732617
35.01295951	41.83496473
35.00226564	42.87687323
35.00077601	42.94299027
34.98880177	43.92465703
34.98589893	44.12990021
35.01290807	44.26069516
35.01639743	44.4931201
35.01915962	45.50667079
34.98802423	45.71823785
34.96722904	46.55370922
34.99939173	47.60908393
34.99113253	49.84083104
35.00930044	52.9630106
34.97453519	54.54209574
35.01049908	55.51273983
34.98893932	57.06009584
35.0068535	59.89594809

35.03646482	61.51479312
34.99338615	60.79201568
34.98939004	60.83943555
34.98169722	60.62472867
34.99786628	60.60958495
35.01566182	57.68240741
34.969768	66.45237646
35.00961691	67.9278543
35.00427705	70.32056585
35.02441271	72.6386161
35.00991134	74.43263634
35.0205711	75.52528443
35.03615683	75.12729048
35.00163228	73.23517578
35.01101314	70.14451902
35.00825905	71.76942392
35.0325136	75.67870995
35.01490715	77.75495272
35.00790928	78.9819566
34.98722614	75.82271754
35.02320806	69.69513558
35.02676129	63.47424606
34.99036757	58.5012193
35.08088484	60.84905728
34.98901352	64.8428999
34.99531037	67.79160598
34.9653678	70.53315516
35.03303298	71.35484417
35.00177324	67.86075203
35.02164338	66.41824066
34.98761635	64.15838254
34.96829674	61.65615218
35.01140145	54.6352575
35.00510056	49.42859243
35.02254818	46.68696341
34.96696266	45.48659922
34.98321702	45.66928957
35.00909027	43.59410844
35.00490397	15.86573821
34.9842455	15.53060656
35.02541529	15.51579831
35.01615337	15.49017234
35.00411154	16.08228275
35.01217649	16.05724325
34.98153519	16.29707542
35.00935876	17.61254485
35.0065817	23.04560089
35.00860806	29.91192318
35.0024987	38.82212699
35.01032166	41.29249854

35.00377965	45.22526222
35.02828804	52.37483951
34.99777853	57.80593022
35.02714286	61.74055993
34.98597489	63.96292196
34.97852104	64.33244975
34.98042539	64.71756208
34.99810688	64.72568688
34.98090352	65.98632825
34.96591237	66.19293936
35.02273298	68.11747229
34.99411057	69.25529044
35.00210332	71.12832011
34.9823761	71.38908341
34.9938523	69.41465599
35.00580353	66.61773638
34.9852521	65.32594802
34.98389131	64.93789771
35.01422314	63.85906012
35.02435324	55.87076665
34.96613639	69.56793472
34.97440002	69.80287206
35.0191988	71.1135563
35.01162286	72.39205791
34.97882885	74.22338158
35.04895866	76.40713781
34.99147665	78.75695688
35.00701796	81.29252826
35.00067526	84.93068021
35.03970904	89.23647121
34.98926969	94.01946507
35.00738096	94.4154402
35.02202418	97.49420929
34.98123821	97.43775041
35.01637888	95.54066884
34.97824384	92.47951082
34.99600816	87.79087248
34.9846328	83.42736688
35.02440652	80.20882226
35.02179699	78.46831965
34.99915527	77.9392826
35.00166086	75.77606217
34.9891743	73.84573832
34.98429756	72.9865494
35.00219925	73.20829594
35.0219069	74.27399361
34.98805095	73.92114192
35.02907502	73.85281812
35.00100656	73.69468219
35.00157986	72.86648197

35.01603436	72.20341822
34.9774112	69.19361791
34.98089742	39.44653725
34.99704984	39.65788412
35.01322822	43.25029141
35.02040407	40.58695959
34.9819076	44.89801571
34.97719281	45.82472194
35.0013663	52.16862463
34.97288243	63.55710444
34.99302654	65.05571916
34.98253208	61.14484863
35.01669725	62.78176617
35.01217004	61.367666
35.01278302	62.58221393
35.00441041	64.25513316
35.02906078	62.3718677
35.00942277	62.14323822
35.00445706	63.49032194
35.0797611	62.81854528
35.0109211	62.26073834
34.99324505	61.78660788
35.01697844	60.12408757
34.97817488	57.56861573
35.02603878	54.68050726
34.9907526	51.98213601
35.01531746	51.40867014
35.05292591	51.95039765
34.98158136	50.7887564
35.0185165	47.32775572
35.01067679	45.46380031
34.9851302	42.48535098
35.0116522	43.53290496
34.98378103	43.05519182
35.01174053	55.33536193
34.98920272	55.88584427
35.00890322	55.44239416
35.00583634	55.23598534
35.01557178	57.33196744
35.03992529	61.00921279
34.98927442	65.31931199
35.03765905	68.13667877
35.00545058	73.05531124
35.00554212	78.75587838
34.98901352	87.40772458
34.98992812	94.15352777
35.00951044	100.3992174
35.00443256	104.501864
35.02238356	108.4924352
35.01899132	110.5611947

34.9881281	109.7381194
34.99117612	109.8625862
35.03146798	109.0002493
35.00995245	108.4333446
35.02989595	106.0691632
35.00734779	102.4364372
35.01073861	101.2771249
34.99102356	99.77754276
35.03720729	97.10846374
34.9634114	94.33271263
35.03924196	91.26741032
35.00599707	90.06643251
34.97521617	87.89186407
35.01160744	85.46183453
34.99976553	81.20690964
35.02053855	71.41679532
35.00306574	48.54056956
35.03816661	46.25343571
35.00856634	46.03565639
35.02147523	45.18347966
35.03195729	45.43182735
35.01537931	47.45986953
34.98466459	48.87183312
34.98802423	52.4067489
35.01769629	61.17841971
35.02073635	71.55762832
35.04205512	82.43731596
34.98549751	88.15420251
35.01164257	95.61684777
35.02351681	100.8177027
35.04152962	105.1462432
35.00346563	105.966679
34.95828583	105.6798439
34.97419578	104.8831449
35.01923761	106.2111771
35.02273298	105.7691479
35.01615516	103.7656456
34.98980032	100.9081086
35.0005977	98.34419176
35.04495452	96.19533046
35.0350974	95.83224425
35.04831446	95.63747515
35.00457168	95.35678214
35.0058134	93.70152676
34.99754077	93.70464404
34.99869746	92.88439191
35.03212464	91.90836955
34.97843618	83.56152863
34.98817343	38.35036976
35.00089489	37.82194343

35.00872214	37.29849922
35.03203076	36.84792724
35.03944531	37.07226284
34.98374211	37.4096206
35.01759635	38.09698896
34.98353667	38.37551087
34.98281584	38.87269553
35.00991207	39.15282966
34.99257165	39.53994062
35.06528681	37.68565176
35.00901842	36.69826987
35.03818008	34.66971023
35.01743759	33.32466067
34.9784732	32.51230602
34.99599049	32.5720484
34.9958103	32.25787725
34.99837396	33.30281071
35.00461703	34.77736945
35.02567484	36.2591061
35.00545058	37.90065692
34.98768001	40.07456436
35.02559673	39.62741874
35.02030259	40.37005857
34.97975892	45.22729824
35.01175521	44.09264
35.00187796	43.35527905
35.03112717	42.6459219
34.97774211	42.92681994
35.02570112	43.16203824
35.01524683	42.63031436
65.61549185	18.41573122
65.59055055	17.19059428
65.56678998	17.01280323
65.59081261	17.17030145
65.58360167	17.40619857
65.58815921	17.73601397
65.67293975	17.97530916
65.57492064	19.02836004
65.59176443	19.38143014
65.57400941	22.19900794
65.58647922	25.0477414
65.5791789	27.16762629
36.51311818	60.60501845
35.02215619	60.51855993
35.00923009	60.80139183
35.03126563	61.260048
34.98066649	63.22565645
35.00178545	62.96536428
34.97399589	64.169757
35.05280226	65.09385308

35.06143382	64.86189414
35.04143386	64.77256502
35.00544492	66.04793478
34.97708073	63.441745
35.01112717	62.7529745
34.99250484	68.64469318
34.98180582	67.63822953
34.99192644	66.15753033
35.01257302	64.77607504
35.01202973	63.75343135
35.01308018	62.54430827
34.98297783	62.59021892
34.99552528	44.88139482
34.98940537	44.82851774
35.00319116	46.01588277
35.00862127	47.84819528
34.98761635	49.93116318
35.00031772	53.14563206
34.99411724	57.90809978
34.9875216	65.10312798
34.99525213	72.43569278
35.04659574	79.27108018
35.0044096	87.25809062
34.98084127	90.69314729
35.00790145	95.1121333
34.99399364	96.68847842
34.99630025	95.84727683
35.00422834	95.45835189
35.00148323	95.71370012
34.98366913	94.43131806
34.97284421	93.44613519
35.00922654	92.12407001
35.02618383	90.12874426
35.03326694	88.51543504
34.97788384	88.23373775
35.01576965	83.96333407
35.02484092	81.2507328
35.03646909	84.92811829
34.99077691	79.58509356
34.96821025	75.91225256
34.98561019	74.01738933
34.97764656	72.18533659
35.01859849	70.0739936
34.99189847	68.30051545
35.08918522	36.74217053
34.97440002	38.14291561
35.00654288	39.53349796
35.00653454	42.45250649
34.98756474	45.23503111
34.98353667	48.15278291

35.03741868	53.39300803
35.0001638	59.78071558
34.99831339	61.87247912
34.98594858	63.63964242
34.98855094	64.06045544
34.9968354	63.90789626
35.00395836	63.13769837
35.00332269	62.2418419
34.99518211	62.01946909
35.01846495	61.9366626
35.05275158	55.15048431
34.98134626	52.43125903
35.02085537	50.83426871
34.9711322	49.75198754
34.99597551	48.72980405
34.98298102	55.17235561
35.05243943	57.66356674
34.99601519	54.54281335
35.03130399	54.74097714
35.00997109	62.30880326
35.08088484	65.06781969
35.05836215	68.40249724
35.00684522	91.07191856
35.04753303	89.72551961
35.04118198	88.54389813
34.98042299	86.52499252
65.59472127	22.27945271
65.64258594	21.78318227
65.56452533	22.97796408
65.57329602	24.74233733
65.57598341	26.17660358
65.58503389	27.7589331
65.66637084	31.20791675
65.57396301	34.67578807
65.60286614	37.76339266
65.63293723	40.52958831
65.60529369	42.67794631
65.60764404	43.29086011
65.61384391	44.25380995
65.64453012	44.6040979
65.63043963	43.71606314
65.61141441	42.29272173
65.61445963	41.53684987
65.5792902	41.21824772
45.44998135	61.91219849
36.02926289	70.62008851
34.99861482	71.66107507
34.9903723	72.14477501
34.98817074	72.23879356
34.99328381	69.18681429

35.04914602	67.10740595
35.01047613	71.76996406
34.99764695	69.72302817
35.00934661	69.61208435
34.99044746	69.56431618
34.99968183	69.26359135
35.00510056	69.45534848
35.00684216	69.2798099
35.01615516	83.07028915
34.96194635	82.58702986
35.00370729	84.16832775
34.97735031	86.93897805
34.99099506	89.66424062
35.03269864	92.48985958
35.04944278	94.53799646
34.96631661	96.70793556
34.97023336	99.93663174
34.97463343	103.4133298
35.00281682	107.2789228
34.98434681	107.2903901
35.04135348	107.522747
35.00895921	105.9259247
35.02988413	105.8014523
35.00914466	104.6581953
34.98228794	103.6523044
34.99250484	99.91707764
34.97325213	97.27711953
34.98828161	94.70459825
34.98951489	92.99494605
34.99233226	94.36872276
34.98597108	94.22773442
35.02981944	91.17076864
35.06564539	89.54313473
34.98281071	96.26016684
34.99513407	93.69907065
35.00686422	89.91905882
35.00728378	88.90085571
35.00292357	86.56167492
34.97456894	85.65945718
34.97302275	84.9280828
35.02051808	77.55444903
35.01032166	78.57110795
34.98240701	80.62182108
35.00027032	80.56517342
34.98123821	81.1317946
34.98232648	86.52904885
34.99953425	85.95093299
35.00626412	87.79603795
35.00692279	92.62661997
35.00423102	97.64102404

35.04634467	96.93016819
35.02987102	95.36896149
34.98058809	96.20658332
35.03149168	97.37550969
34.98983523	101.5642491
35.04730759	102.7537318
34.96573672	102.1372283
34.99026314	102.0430479
34.97766784	99.84007647
35.00400947	100.0289782
35.00105913	97.92502997
35.02494765	92.97166336
34.97137073	95.28195477
35.01352143	92.54529022
35.03024336	97.91548547
35.0212982	100.5271366
34.98518227	101.3083374
35.00966711	107.0722259
34.98852762	104.5113108
35.01226231	106.1856882
34.98645111	104.7650358
35.03241389	104.9896305
35.02653802	107.7737618
35.0070987	106.905201
34.97474295	106.799148
35.00105349	105.2024723
35.01174226	105.5010921
35.01576715	106.4724136
35.01871566	108.3106214
35.04370909	112.4995541
35.01484855	114.0990793
34.98181766	117.4668137
35.04006853	121.5572751
34.99691711	115.5320918
35.00948902	107.3556302
35.05227202	96.62328007
34.98598195	86.66714456
35.00843109	78.0107945
34.99066559	70.93634821
34.99363359	63.03920931
34.96840721	64.70688541
35.07747664	64.74079346
35.0019351	64.83768562
34.99853473	65.87031676
34.99760172	65.17947357
35.07378423	61.55383319
35.0196269	60.17738556
35.01378074	65.65566363
35.03566754	64.60889656
35.01603436	62.70031664

35.02952449	62.39824923
34.96502621	62.20748111
35.01339656	62.25433887
34.9639643	62.59511641
37.66608455	54.43050946
34.97545746	56.71323289
35.0277268	56.76079925
34.98655535	56.95627691
35.00798032	57.95671106
34.9755031	58.58643807
35.02001019	59.60196721
34.99891396	61.77861183
35.00912894	64.54931209
34.99235269	67.00846257
34.98367935	68.34074838
34.9995722	68.94148551
34.99035839	70.07479831
35.0123274	69.7825774
35.01423838	70.62651553
35.01202973	71.71532411
34.98762177	74.18170615
35.01458264	75.9208329
34.99005945	77.87865142
35.04291933	79.44822223
34.98439346	80.42992804
35.00133473	81.10286275
34.99556382	81.56963951
34.97565308	78.28430684
34.99044724	79.45997757
35.04909139	83.30203606
35.02284947	81.35180572
34.98116947	80.20959514
34.98313048	78.68362061
35.02612556	77.51505152
35.00184794	76.50763604
35.01140145	75.39380217
34.9960275	115.5889798
34.98493092	115.5454786
35.017126	115.790802
35.0024531	116.3912943
34.9908905	117.2237414
35.012753	118.5925076
35.01046464	120.7252041
34.97142184	122.8351976
35.01110169	125.5073928
35.01327943	127.8776722
34.985589	129.0859719
35.06799058	128.0759824
34.98901352	127.6494305
35.00771224	126.7302807

34.99465223	128.0675358
35.00862642	127.1344232
35.0478569	127.3489181
35.03780107	127.2852128
34.9804494	127.5715496
35.0277268	128.4791471
35.0168421	128.5178181
35.02001196	129.1446754
35.0583865	130.556637
34.98042121	124.321299
35.00478645	125.7170101
35.01482918	130.8053573
35.02117324	127.707164
35.01047768	129.3777453
35.00307294	126.9387729
35.01327943	126.3366375
35.02392972	123.7156528
35.00228694	121.9150007
35.0140377	72.53189447
35.05829117	73.20869694
34.98629746	72.61801772
34.9932184	72.13572729
34.97774211	73.54785636
35.03979627	74.60062691
34.98737009	78.76349004
35.01108608	80.15682054
35.02499053	82.834384
35.00564288	84.54341742
35.03521603	86.78434464
34.98992812	84.91206952
35.01339656	84.86264639
35.01736231	82.85039488
34.99453494	82.34937206
35.000981	80.8576371
34.97925294	81.16062809
35.06714027	79.52933475
35.00269064	80.26073332
35.03238243	82.29873687
35.01769304	83.76965108
34.99026396	87.10140541
35.03370227	85.95948908
35.02770315	83.01612405
34.99328381	81.32946019
35.06297674	85.79979583
34.98998139	83.18694159
35.01905986	78.33281247
34.9712944	78.30280088
34.97744485	78.67995822
34.99740243	78.92471392
34.98890738	79.25766193

65.5695371	9.617624754
65.58137971	8.126209354
65.6120877	7.551654052
65.59320506	6.967707008
65.59831182	7.182326978
65.61326023	7.784867289
65.58565444	7.766547609
65.60179426	7.369590931
65.60511552	6.710053048
65.59195698	6.320347436
65.57337085	6.710166679
65.58053052	5.527436533
65.60003698	5.267667034
65.58421727	4.030711102
49.44528262	19.29767216
37.10116773	30.97412139
34.97365945	33.33432806
34.97769193	33.558592
35.00911982	35.07418919
35.0215878	35.67422716
35.02689415	36.59353502
34.98556142	35.92096211
35.04659863	37.91107103
35.01128781	35.29870932
35.03583621	36.42722131
34.98551825	40.47136296
35.05823803	38.04790624
35.03388117	38.38352834
34.9876281	37.97565497
34.98297783	37.89569646
34.98482189	37.41529862
35.05218679	36.59845093
34.97013619	101.7467788
35.00845996	100.1761724
35.02019185	99.76023092
35.0369621	98.09971557
34.97419578	96.9450813
35.00562893	95.68789811
35.01219298	95.33447665
35.01717792	93.62752555
35.03032725	91.05534851
34.97499853	88.34342104
34.99636614	87.45181571
34.99682191	85.4179921
35.01062091	82.03681994
34.9852521	76.91803853
34.99220429	75.9602399
34.98250735	74.07417574
34.99805753	72.71309039
34.99212668	70.20470839

35.00400222	67.94936319
35.01233128	68.97690527
35.03637719	68.40995881
35.04517492	68.09542256
34.99977038	70.66513102
34.9999481	66.4131409
34.9851302	65.57569001
34.99250484	70.49157419
34.98521267	67.65000745
34.98989215	70.90207838
35.01600856	70.45776873
35.00059655	73.57085854
35.01927326	74.93045907
34.9993912	77.22682115
65.60858606	3.304855134
65.59991929	1.902116505
65.58139831	1.578646679
65.59778989	1.221283684
65.57583386	0.730280122
65.57584609	1.214538752
65.5668581	0.732838741
65.58409795	1.588811084
65.59511054	2.067635841
65.60289658	3.128909391
65.59596532	4.411594168
65.57661169	4.964651433
65.62189447	4.639912196
65.60409355	4.439556538
65.63293723	3.143426631
65.62993999	3.277836616
65.61788407	2.412232085
65.58633034	2.102952725
65.61588636	0.772186993
65.63646501	1.517470298
65.61438086	1.633095358
65.5869188	3.476462755
65.57104781	3.721766318
65.59982419	1.920640401
65.58489596	1.997119582
65.59650834	8.965908368
65.59145671	8.281935005
65.58565444	6.234825481
65.61445963	6.62893736
65.63300203	7.086738656
65.58356365	6.942911309
65.61336082	6.94398732
35.00109453	72.44901602
35.00620559	70.44748364
34.99206278	69.14421047
35.01701244	67.95973757

35.05254748	66.95274136
35.02747231	66.86403725
35.01485025	69.54471626
35.00872904	71.91229336
35.01903674	74.41115887
35.00486712	76.91200677
34.9793494	79.37026633
35.03578687	79.30542303
35.0384495	79.02346187
34.99711507	76.38222906
35.04454508	73.8971372
34.9823915	71.2267927
34.99961443	70.11999169
34.97979003	68.50035682
35.00845996	69.66042587
34.99328381	71.11582876
34.98140831	72.23651253
35.02985858	73.7356226
35.01048185	74.9329503
35.0014089	73.24021921
35.03080428	73.00112828
34.99333576	79.10278311
35.00989593	76.18382784
34.98338983	75.34341684
34.96143445	75.32369041
35.03616115	74.9104099
35.00056615	74.30621733
34.98738957	74.49071282
34.98789848	45.51760591
34.98568979	46.34450512
34.9823915	47.48027257
35.0294908	45.87890661
35.01567502	46.92858323
34.99322269	47.6733073
35.01173832	50.67982335
34.98250735	53.4184409
35.03255356	56.18001469
34.97817488	57.86085781
34.97709674	59.30425544
35.02900313	59.95019704
35.00682611	61.28827741
35.00935876	61.88103692
34.98836597	64.02735879
35.03591673	64.02624597
34.97175645	65.83262371
34.99490197	65.60003747
35.02631584	65.27938474
34.98098942	66.29719666
35.03092052	67.15396338
35.03714608	68.12691013

34.99948799	68.33610509
34.98199356	69.4946551
34.97278806	70.22686266
34.98655535	74.40590869
35.00218695	73.95376805
34.97639834	70.71195432
34.999725	71.59880854
34.9823915	71.09499603
35.01045942	70.16504835
35.04288775	68.49747428
34.98228794	93.23953815
34.98415467	94.17104904
34.98663356	95.70456331
35.04071038	97.04449245
35.01008698	98.7417373
35.03357125	97.97999507
34.98353667	98.59346612
34.99275987	97.95817044
34.98041164	97.97641192
35.03577424	98.11799098
35.06019317	95.53984586
35.02645608	103.2976498
34.9953542	101.3455278
34.98868654	96.31721376
35.0133498	95.80710831
35.03433724	93.6427708
34.98144451	92.03596492
34.98519158	91.69684297
35.01254415	92.49403425
34.97934451	93.0070187
35.00930029	90.49828622
34.99473856	89.71718038
35.02471735	89.99459887
34.98056193	84.38357811
35.03883884	84.69822207
35.03314138	85.9990621
35.00019301	83.31905628
34.98650458	84.89064209
34.98109032	83.70596107
35.04607423	86.00654279
35.00406294	86.22772736
35.03277108	86.58433293
35.00871438	93.78545219
34.98761635	90.94230326
35.01414047	89.84366939
34.97500674	89.06939601
35.07839101	88.16008573
35.0146079	89.53275971
35.02401928	91.49808372
35.00735573	95.02564051

34.99139855	97.4339197
34.9793494	99.36074082
35.01602444	102.1660677
35.00015239	104.9020828
35.00920976	108.7434082
35.00860139	109.9822024
35.01621776	110.2929817
35.01254415	110.7518066
34.99397719	110.6933837
35.02400497	109.3139788
35.00568906	107.5286813
35.04319129	106.0683312
35.00304467	105.8070941
35.0754329	107.2596193
34.99825365	105.3374957
34.97882885	100.4588155
35.04027506	99.37652886
34.99211251	103.5366993
35.04301089	100.7230689
34.98920759	97.89053852
34.99493567	96.90652072
35.01797181	96.2960127
35.01371868	94.9536381
35.05478246	94.47862698
34.96328164	79.2742277
35.01833389	79.83602179
34.97249237	81.25152213
35.05864528	83.35951798
34.98475308	85.57251955
34.97138564	87.27543221
35.02981129	90.21303599
34.96213974	94.21029801
34.98868688	98.0018739
34.99192396	98.89787665
35.01865997	100.6447478
34.98116117	100.183094
34.98348733	99.43036906
34.99059115	98.08049957
35.02290787	94.58849561
35.00704132	93.35156294
35.02843424	93.67317755
35.04616247	92.94544433
35.00472694	94.22501687
34.9876712	92.89938794
34.97334831	89.99890596
34.99527707	87.93347831
35.00779663	85.99982178
35.0048598	81.52210665
35.06394003	79.8939511
35.05074637	85.01925116

34.99374526	82.93938618
34.96729048	82.03548651
35.0058134	81.34038966
34.98217846	80.91794272
34.99818092	80.99548411
34.9851302	82.01885326
35.00528868	131.4969411
34.99536698	131.31973
34.99557943	131.5014327
35.04570741	131.3665897
34.9896182	130.8438297
35.05616334	130.0146428
34.98687995	129.3891402
35.02043536	128.2496089
35.00960809	129.2400875
34.99354369	132.282685
35.03453512	134.7312572
35.00225831	137.7549276
35.02632695	137.8191724
35.02531507	136.4006374
34.98228794	133.9511395
35.01755199	133.8588668
34.99212668	135.0357222
35.0205669	136.241554
34.98421834	138.257216
35.00668645	141.305857
34.99529596	141.5152368
34.98564533	140.7568088
35.01403018	140.9005385
35.00384468	142.2884488
34.98151086	143.6716764
34.98660849	145.3243576
34.99361798	146.8637618
35.00894835	147.0790867
34.99234122	146.0561353
34.98982854	145.375572
34.97175645	154.2889225
35.02924044	143.9626536
34.99922229	210.2018279
34.96804008	198.0843962
34.96023225	181.9112947
34.99411724	159.9767971
35.01974464	154.498182
35.01524856	146.2443304
34.98042299	136.0253836
34.97621904	118.9474746
35.00770992	110.6509752
35.00133473	106.3721206
35.05085593	97.42129136
35.05151539	88.8066112

34.98429756	75.77178124
35.01049031	70.41562649
35.01128781	71.34035152
34.97841065	68.03222119
34.98117236	68.14419
35.02291738	63.08868547
35.05727845	53.93744804
34.97966616	47.29088539
35.0128934	44.86895478
35.00934661	47.35583408
34.98585273	47.36261671
35.00501113	47.35970138
35.00838233	44.08575348
34.99374526	41.93424232
34.82525399	39.73032796
34.84706912	35.6246531
34.82988648	34.40575838
34.82273336	33.98682282
34.82846648	30.55500855
34.8073939	33.54006389
35.00852237	125.2580353
35.03337226	122.4296394
35.01518083	119.3239478
35.02699387	115.7959717
35.0126437	113.4046396
34.9874359	111.3411644
35.03112466	109.4946024
35.06456819	107.8063373
34.98728375	106.6315309
35.03352927	104.938768
34.98457071	102.7456679
35.00525416	100.3939791
34.97521782	98.05340213
35.01518083	93.38702234
35.01513586	89.85205864
35.01936156	86.69685179
35.07390851	84.06332837
34.98843844	82.85053279
35.00411248	81.02283974
34.96131285	80.1962348
35.00018414	80.52389319
35.00745455	80.83727862
34.97715456	81.73212134
35.00930029	82.62598402
34.969768	82.15680592
34.99574342	81.5921713
35.03397588	80.6006318
34.99773316	78.79124965
35.02257303	76.97521867
35.03565211	75.26520895

34.99626706	73.53940634
35.00650511	72.1475081
35.00134704	175.1852946
34.99982942	172.0360765
35.02196066	166.7607803
35.00856634	163.1246448
34.99201656	159.8215288
35.02556949	155.5975024
35.02980374	146.4373503
34.99626706	137.7560118
35.03181328	128.80296
35.01365219	123.0002708
35.00679424	113.9562827
35.01738962	106.7074483
34.99892565	102.7713087
35.01830752	96.8582699
34.99325789	92.42102926
35.00564288	88.63924123
34.98780954	83.97270109
35.0019088	79.50162563
35.00410157	76.62682598
34.98040403	72.25694268
35.02572358	68.82372341
34.99992191	65.15377031
34.83547453	64.51774399
34.8178253	62.77483243
34.84190769	60.3223851
34.95775462	58.50270887
34.8271492	56.78387817
34.83767901	55.95112961
34.84104768	54.60289242
34.84264443	53.44091121
34.81639141	54.99401773
34.81611397	46.38298756
35.03232033	168.5135678
35.02566478	164.5085025
35.01474663	148.9248032
35.07255934	129.9749652
35.01603436	116.7863155
34.99956278	112.7799432
35.01981744	112.5826927
35.05753564	112.6928062
34.9784732	113.6207244
34.99389789	114.3479656
34.98563202	116.8266632
35.03229591	115.0523332
35.03171261	115.4738417
34.99268727	109.7339054
35.01563256	104.8566915
34.97693273	102.2889624

35.00170888	98.22933554
34.97249181	96.26297958
35.00386028	98.79601252
35.01697844	102.0501658
35.03491219	100.7385343
35.02791319	95.89964234
35.02086564	97.76836976
34.96097942	107.365528
34.98327842	117.708915
35.02242194	114.2715927
35.02809237	110.9341725
35.03622723	115.4776088
35.02065443	119.9337218
34.99092728	118.0512013
35.00568906	90.2681885
34.97142184	91.62932768
30.51673596	72.99846051
30.63129729	70.52112016
30.7592436	68.22030646
30.06679416	67.19122075
29.48885565	66.39916212
28.95811926	65.24670341
27.95314366	64.53531806
27.28775196	63.50646272
26.69252676	61.51271983
26.73125002	58.19695963
26.43558093	54.67370201
25.41455061	51.75655692
25.24964449	49.18153507
25.26167859	47.21027885
25.49463141	45.37505909
25.34546232	44.35373031
25.24593412	42.98820159
25.23913556	41.05726717
25.32086834	39.11562882
24.70057953	38.78324691
24.10637629	38.74173354
23.78059709	38.32023906
23.90347448	36.18283506
23.99990087	34.63547606
24.16828398	32.89654167
23.7051657	32.28850753
23.92265786	30.29616451
24.02945852	29.1141323
24.01587794	28.06026147
23.78535158	27.07004919
23.70753476	26.19641167
23.7382764	25.59486672
35.02964015	377.2245104
34.98909174	382.9505067

34.98382577	389.4920046
35.00063312	393.7701694
34.99800261	401.3920368
35.00242119	413.004973
35.00610528	424.6772889
35.01174341	436.5486519
35.00654288	445.7169236
35.01209794	453.4747012
34.97456894	458.581078
34.99378188	466.6480828
35.00320082	471.6872322
34.99579198	475.1812863
35.02001019	477.471644
35.02238356	478.3811728
35.01587634	477.7668357
34.99100233	478.1295536
34.99550719	477.2180458
35.01044785	474.6070867
35.02948952	470.1953009
34.98525086	464.7724831
35.05468855	458.9665934
35.01238329	450.5705272
34.97912093	440.6183066
35.02861196	432.1933629
34.98706017	424.224579
34.97654391	416.9684324
34.99864275	410.3967878
34.98047725	404.7934307
35.0137299	401.3316123
34.982117	397.6617305
34.98305758	206.2919943
35.00684216	209.2926549
34.97350497	214.2068786
35.01941281	215.4345196
35.07280988	216.4329766
35.01694687	218.6646184
35.03384311	220.6252518
34.98486997	222.7307429
34.97325509	221.2269817
35.03563219	220.4825861
34.98651741	219.5280557
34.99036032	218.4080315
35.05081916	219.7194118
34.9946271	220.500438
34.99816856	221.1211028
34.96995402	219.5318407
35.01686907	220.2003483
34.98151752	219.8936087
35.02009918	220.2838163
35.0191988	217.3096864

35.0393369	214.8508088
35.00705732	214.319506
35.02392972	213.6300452
34.98294416	212.4288695
35.0290822	211.4063048
34.98082043	211.0579915
34.98890738	210.8021193
35.03794101	209.4470152
34.99948799	208.2050061
34.98811946	207.3714447
34.97471429	209.0434397
35.01360387	208.5000356
35.00733528	99.07035314
35.02539024	97.33248793
35.04944278	95.38170144
34.99405103	93.83183265
35.06147778	92.74170085
34.99488367	92.14880873
34.98495414	91.58051389
34.98356843	91.09806043
35.04223105	90.48585818
34.96613639	91.75219954
35.04716043	92.09528455
35.02242467	92.20385568
34.97407099	93.07653532
34.99816856	93.24615779
35.04444215	93.18677037
34.98663356	94.00703709
35.06425319	94.44438043
34.9957163	97.08873472
35.00332269	101.3028177
35.01075293	106.8160462
34.96143445	108.4183978
34.99574668	110.4869481
34.99911573	112.6941255
34.98761635	115.7372571
34.9855906	118.6393883
35.01637195	121.0471275
35.07390851	120.1753913
34.98322359	118.4604374
34.9851302	117.7073107
35.01490788	116.6885044
35.01079177	97.85740643
34.99133403	117.0996253
34.9900758	214.8838135
35.0369838	214.143337
35.023479	213.4759845
35.04105844	213.6722397
35.00692279	206.5173317
35.01243299	205.1504186

35.02238327	205.9153947
35.00363061	207.0097291
35.01582738	208.1740847
35.03462079	208.9166224
34.9708571	207.3177018
35.0236597	206.5102108
35.07199956	205.2821807
34.99230616	203.8162298
34.98799222	205.0973666
34.97780868	206.7522288
35.00487454	207.9823399
35.08210345	209.5582055
34.97999922	211.4044076
35.00050777	213.0447384
35.0021672	214.7054218
35.03198248	217.8983762
35.00306609	219.4653382
35.03528244	222.4560727
35.01257302	218.8275489
35.00459405	216.2067067
35.01047857	213.2389671
35.01164257	211.0141131
35.01024596	204.5191044
34.995506	200.9250577
35.03401465	198.4358037
35.01774206	198.6043081
35.00864856	126.5501618
34.9818082	115.92738
35.01736231	109.2701097
34.9823915	104.1607347
35.05777417	97.05425719
34.99483702	94.75209048
35.03401937	91.91420168
35.02596357	83.11977748
35.00895921	84.80830068
34.99948628	74.07886439
35.01740275	67.32637581
35.00799673	67.23117307
35.00617329	57.97619442
34.98134626	56.1041792
34.98811638	54.21842639
35.01791067	52.18175036
34.9849826	45.99811763
35.03937399	40.47194263
35.00951184	36.06081927
34.99921417	34.66290272
34.97175645	27.71635537
35.00735877	28.83763583
35.00572033	22.28538709
35.05055357	22.71786588

35.02711214	20.80404013
35.04728709	18.8601554
34.97296384	20.3161214
35.00417962	22.80991635
34.86507484	18.3681201
34.82021541	19.09806052
34.84018249	18.17902873
34.79806677	18.81925428
34.99420951	185.6219208
35.02184062	145.3840499
35.05698124	137.0399265
35.02876054	129.9339299
34.98594858	134.8496177
34.985589	151.6334185
34.98563202	146.1347103
34.9818082	138.2552616
34.99431718	130.6259811
35.00154474	125.1880657
35.00459405	120.3680989
35.0061542	113.4787184
35.00191095	109.4855999
34.9938523	101.1351376
35.00289634	96.10206067
34.98817274	94.53302959
34.99217698	84.92333768
34.99260151	76.46150847
34.99464876	73.58039572
35.02043536	70.84307563
35.00125679	71.01041492
35.05950679	72.37452064
35.01089508	69.50508853
35.00138536	65.65311953
35.04693614	63.89459862
35.00142008	61.16845658
34.98647061	57.98564183
35.02229953	56.70394832
35.05641943	55.90735191
35.0108636	57.67002103
35.02978221	56.63881847
35.0070374	56.14342551
34.81686705	42.10215652
34.82323294	40.78524948
34.81751919	39.63083883
34.30751532	38.65292818
33.42869958	38.52649405
31.5002739	38.54216733
30.28618156	37.50939361
30.41598999	34.73766475
30.50548923	32.34074734
29.43818752	30.57547611

27.65660197	29.6153336
27.03040919	27.53570936
26.99112171	24.87183536
26.64118732	22.9674501
25.56587002	18.71118646
25.53300133	17.15878731
25.22433547	16.08894901
25.247636	14.66012097
25.34728969	13.20772829
25.61736813	11.96993552
25.21443336	11.71959738
25.34917674	11.15377244
25.41028187	10.76004054
25.44573037	10.3531399
25.41506085	10.9831428
25.25951819	11.58697466
25.2698583	11.96821873
25.25600272	12.45511359
25.37733145	12.18668276
25.27752959	12.10235309
24.42120866	0
23.18765002	0
35.01563072	190.7456292
35.03494046	191.3606384
35.00407679	191.8820431
35.05759089	192.3194217
34.98324959	192.8673261
35.00486132	193.7305601
34.99218504	194.3275263
35.02900744	194.4894181
35.03990961	194.4761458
34.98466197	194.215007
34.9903595	193.838278
35.0019351	193.6099215
35.05144184	194.0681525
34.99267234	194.5058027
35.00157049	194.2932178
35.02516338	193.0274833
35.01138069	192.0593318
35.00430679	191.214536
34.99713102	190.1204338
34.97778055	189.1722866
35.01615516	188.2109855
35.01219298	187.0409747
34.99325789	186.0844207
34.98395321	184.2375397
34.97603393	182.6313749
35.01561003	180.6597624
35.00759847	178.5142622
35.02077767	176.4923705

35.00318977	174.3672716
34.99962291	172.4280049
34.99111721	172.3431659
35.02202061	170.3839197
35.00544492	239.8842989
34.99799693	239.4610061
35.03081635	239.1592497
34.96363848	239.3856679
34.9709357	238.1694454
35.01224496	240.4683532
35.0122025	242.342381
35.01576033	246.1272367
35.05299023	246.2385789
35.01032166	246.9476465
34.99660211	246.8839538
34.98645111	245.4316417
35.05109834	244.9961023
34.96994342	244.7024416
35.01032166	244.2685737
35.01272996	242.5199133
35.04998377	243.220027
34.98565446	244.2819167
34.99395982	245.2073918
34.98738957	246.2495168
34.97183212	247.3236181
34.99688076	247.8685819
35.0124752	247.8372709
34.98366913	247.3652186
35.01019001	247.5251234
34.96775686	246.8042802
35.05258208	245.8755645
35.01544784	243.4224684
34.99347744	241.1389155
34.99495697	238.835637
35.00747318	236.7135268
35.03573036	234.5002996
35.02487305	178.3279713
34.99821421	172.1235362
35.02948952	169.2561233
35.00961747	167.4461312
35.02615284	167.1783084
34.9968354	157.5173329
35.0018229	155.9972796
35.04013333	150.9774349
34.99364283	146.9969043
35.01579749	146.4410555
34.99741931	147.5278595
35.0106558	145.1582398
35.01130457	139.4448593
35.04244266	136.4731967

35.04021708	133.5895204
34.98701129	128.0609749
35.02833598	117.3717339
34.9954858	111.7537919
34.98554651	103.3955289
34.98456671	99.32060901
35.00181539	98.23674283
35.01088057	96.45660525
34.99399364	95.93913362
35.00233677	93.88038437
34.97142184	91.9210493
34.98374206	89.40816077
34.83197566	87.72563799
34.83479685	86.95148112
34.84850729	86.82924525
34.85502418	87.94578722
34.80238878	69.31045982
34.83530515	67.78644312
35.02147523	37.34489008
34.97697355	37.46673866
34.9852521	37.82622727
34.99000693	38.14745753
35.00573504	38.37042949
35.01774206	39.23951217
34.97596568	39.5494387
34.99442435	39.15606952
35.00319116	39.91625925
35.04180381	40.05744315
34.99315475	40.29827749
34.98522914	38.91899472
34.98144451	38.67703963
35.02649968	38.96003566
35.01650588	39.44180438
35.02288452	39.34335844
35.00650511	39.57529865
35.01747564	39.34713971
34.99049284	39.33820442
34.99798656	39.38709777
35.02900313	38.90893629
35.01229191	38.87581935
35.0093325	39.65612953
34.9749133	40.18899697
35.00049597	39.74873354
35.00119726	42.11137173
34.98466459	42.62456598
35.02084062	43.61778475
35.07150586	44.61288522
35.03883884	45.33004155
34.98948006	45.82216118
35.0084016	46.20792053

35.02081047	304.5477932
35.01047483	301.7660842
35.00533548	299.3622291
34.9740302	296.5664646
35.02762396	294.4025768
34.99185718	291.7668939
35.02237376	289.2859187
35.01576245	286.9407389
34.97806052	284.5028975
34.96299616	281.8336509
35.01638341	279.5469038
34.96291328	276.2816763
35.02015442	271.4824214
35.00569553	266.700278
35.02173387	262.6276985
34.976362	257.6514867
34.99379093	252.6151936
35.04525993	247.8306016
34.99804404	244.4107927
35.00682611	241.2707941
35.00773443	237.779293
35.02608965	234.8262649
34.97722789	232.3329625
35.01019309	228.0730855
35.02236522	222.4037923
35.04507774	222.1476013
34.99836936	219.9399114
34.9884886	219.700676
35.02108251	219.6349635
34.97998354	220.1197264
34.98466377	220.2826434
35.00040475	220.8808993

Table S13. Decomposition Analysis of Ischemic Heart Disease Mortality: Contributions [illegible] Population Growth, and Epidemiological Changes Across SDI and GBD Regions, 199[illegible]

location_id	location_name	overll_difference	varname
1	Global	3624.5	Aging
1	Global	3624.5	Population
1	Global	3624.5	Epidemiological change
5	East Asia	1480.59	Aging
5	East Asia	1480.59	Population
5	East Asia	1480.59	Epidemiological change
9	Southeast Asia	386.06	Aging
9	Southeast Asia	386.06	Population
9	Southeast Asia	386.06	Epidemiological change
21	Oceania	6.61	Aging
21	Oceania	6.61	Population
21	Oceania	6.61	Epidemiological change
32	Central Asia	567.53	Aging
32	Central Asia	567.53	Population
32	Central Asia	567.53	Epidemiological change
42	Central Europe	-32.81	Aging
42	Central Europe	-32.81	Population
42	Central Europe	-32.81	Epidemiological change
56	Eastern Europe	117.35	Aging
56	Eastern Europe	117.35	Population
56	Eastern Europe	117.35	Epidemiological change
65	High-income Asia Pacific	30.12	Aging
65	High-income Asia Pacific	30.12	Population
65	High-income Asia Pacific	30.12	Epidemiological change
70	Australasia	-15.52	Aging
70	Australasia	-15.52	Population
70	Australasia	-15.52	Epidemiological change
73	Western Europe	-336.25	Aging
73	Western Europe	-336.25	Population
73	Western Europe	-336.25	Epidemiological change
96	Southern Latin America	-13.69	Aging
96	Southern Latin America	-13.69	Population
96	Southern Latin America	-13.69	Epidemiological change
100	High-income North America	-175.28	Aging
100	High-income North America	-175.28	Population
100	High-income North America	-175.28	Epidemiological change
104	Caribbean	16.85	Aging
104	Caribbean	16.85	Population
104	Caribbean	16.85	Epidemiological change
120	Andean Latin America	18.23	Aging
120	Andean Latin America	18.23	Population
120	Andean Latin America	18.23	Epidemiological change
124	Central Latin America	158.19	Aging
124	Central Latin America	158.19	Population
124	Central Latin America	158.19	Epidemiological change
134	Tropical Latin America	54.75	Aging

134	Tropical Latin America	54.75	Population
134	Tropical Latin America	54.75	Epidemiological change
138	North Africa and Middle East	383.1	Aging
138	North Africa and Middle East	383.1	Population
138	North Africa and Middle East	383.1	Epidemiological change
159	South Asia	1947.33	Aging
159	South Asia	1947.33	Population
159	South Asia	1947.33	Epidemiological change
167	Central Sub-Saharan Africa	26.58	Aging
167	Central Sub-Saharan Africa	26.58	Population
167	Central Sub-Saharan Africa	26.58	Epidemiological change
174	Eastern Sub-Saharan Africa	91.24	Aging
174	Eastern Sub-Saharan Africa	91.24	Population
174	Eastern Sub-Saharan Africa	91.24	Epidemiological change
192	Southern Sub-Saharan Africa	21.92	Aging
192	Southern Sub-Saharan Africa	21.92	Population
192	Southern Sub-Saharan Africa	21.92	Epidemiological change
199	Western Sub-Saharan Africa	144	Aging
199	Western Sub-Saharan Africa	144	Population
199	Western Sub-Saharan Africa	144	Epidemiological change
44634	High-middle SDI	1740.3	Aging
44634	High-middle SDI	1740.3	Population
44634	High-middle SDI	1740.3	Epidemiological change
44635	High SDI	-1765.87	Aging
44635	High SDI	-1765.87	Population
44635	High SDI	-1765.87	Epidemiological change
44636	Low-middle SDI	1633.24	Aging
44636	Low-middle SDI	1633.24	Population
44636	Low-middle SDI	1633.24	Epidemiological change
44637	Low SDI	264.66	Aging
44637	Low SDI	264.66	Population
44637	Low SDI	264.66	Epidemiological change
44639	Middle SDI	2847.25	Aging
44639	Middle SDI	2847.25	Population
44639	Middle SDI	2847.25	Epidemiological change

of Aging, 0-2021.
**value**
2866.338
3545.049
-2786.886
1005.711
366.641
108.236
167.255
239.847
-21.046
1.112
6.057
-0.561
166.18
987.207
-585.858
204.439
7.428
-244.675
337.012
-18.461
-201.197
245.607
42.649
-258.139
30.375
31.433
-77.328
372.015
128.859
-837.125
21.022
26.883
-61.594
315.082
328.649
-819.011
23.192
22.478
-28.824
11.394
20.038
-13.198
90.888
99.881
-32.58
80.623

84.324
-110.194
142.673
450.575
-210.149
513.045
1282.492
151.795
-1.959
34.447
-5.91
-6.853
96.157
1.931
4.475
15.565
1.876
-33.316
177.873
-0.556
2300.432
1942.364
-2502.495
1731.8
1593.032
-5090.699
423.425
1203.594
6.224
-12.611
305.525
-28.25
1621.361
1428.869
-202.977

**Table S14. Risk Factor Analysis of Ischemic Heart Disease Mortality: Contributions of Top 8
1990 and 2021.**

location_id	location_name	year	rei_name	val
1	Global	1990	Air pollution	45.07209786
1	Global	1990	Dietary risks	75.05012648
1	Global	1990	High LDL cholesterol	50.47907356
1	Global	1990	High body-mass index	13.77581371
1	Global	1990	High fasting plasma glucose	17.08233428
1	Global	1990	High systolic blood pressure	85.74426636
1	Global	1990	Impaired kidney function	26.52519471
1	Global	1990	Tobacco	33.46980676
1	Global	1990	other risk	20.14506979
1	Global	2021	Air pollution	29.88041659
1	Global	2021	Dietary risks	46.7582474
1	Global	2021	High LDL cholesterol	32.28635416
1	Global	2021	High body-mass index	11.71022721
1	Global	2021	High fasting plasma glucose	16.26780206
1	Global	2021	High systolic blood pressure	56.72633868
1	Global	2021	Impaired kidney function	17.18265441
1	Global	2021	Tobacco	19.93729551
1	Global	2021	other risk	15.85455001
5	East Asia	1990	Air pollution	41.33363052
5	East Asia	1990	Dietary risks	46.23940923
5	East Asia	1990	High LDL cholesterol	25.05662
5	East Asia	1990	High body-mass index	3.34206363
5	East Asia	1990	High fasting plasma glucose	10.72913162
5	East Asia	1990	High systolic blood pressure	40.92961616
5	East Asia	1990	Impaired kidney function	14.63695693
5	East Asia	1990	Tobacco	25.07966786
5	East Asia	1990	other risk	14.79011231
5	East Asia	2021	Air pollution	37.88099571
5	East Asia	2021	Dietary risks	43.57290969
5	East Asia	2021	High LDL cholesterol	28.7438245
5	East Asia	2021	High body-mass index	8.105100143
5	East Asia	2021	High fasting plasma glucose	14.20648713
5	East Asia	2021	High systolic blood pressure	56.10386019
5	East Asia	2021	Impaired kidney function	14.65262684
5	East Asia	2021	Tobacco	25.82855236
5	East Asia	2021	other risk	18.55370187
9	Southeast Asia	1990	Air pollution	47.664622
9	Southeast Asia	1990	Dietary risks	51.3731452
9	Southeast Asia	1990	High LDL cholesterol	34.25375133
9	Southeast Asia	1990	High body-mass index	3.694247408
9	Southeast Asia	1990	High fasting plasma glucose	12.1352367
9	Southeast Asia	1990	High systolic blood pressure	59.27861496
9	Southeast Asia	1990	Impaired kidney function	20.82090405
9	Southeast Asia	1990	Tobacco	26.79141071
9	Southeast Asia	1990	other risk	9.608538151
9	Southeast Asia	2021	Air pollution	32.16343198

9	Southeast Asia	2021	Dietary risks	40.34491988
9	Southeast Asia	2021	High LDL cholesterol	32.56604133
9	Southeast Asia	2021	High body-mass index	6.502904443
9	Southeast Asia	2021	High fasting plasma glucose	15.01743721
9	Southeast Asia	2021	High systolic blood pressure	62.18473807
9	Southeast Asia	2021	Impaired kidney function	20.51363867
9	Southeast Asia	2021	Tobacco	22.4570202
9	Southeast Asia	2021	other risk	10.53113633
21	Oceania	1990	Air pollution	71.4723123
21	Oceania	1990	Dietary risks	85.28280459
21	Oceania	1990	High LDL cholesterol	57.91521335
21	Oceania	1990	High body-mass index	17.77100992
21	Oceania	1990	High fasting plasma glucose	28.74853832
21	Oceania	1990	High systolic blood pressure	64.26472246
21	Oceania	1990	Impaired kidney function	26.88231025
21	Oceania	1990	Tobacco	36.42996752
21	Oceania	1990	other risk	11.77292497
21	Oceania	2021	Air pollution	61.99731633
21	Oceania	2021	Dietary risks	77.32599437
21	Oceania	2021	High LDL cholesterol	54.08384017
21	Oceania	2021	High body-mass index	21.22868965
21	Oceania	2021	High fasting plasma glucose	32.62961479
21	Oceania	2021	High systolic blood pressure	70.96561281
21	Oceania	2021	Impaired kidney function	25.91555738
21	Oceania	2021	Tobacco	31.73125324
21	Oceania	2021	other risk	10.71085846
32	Central Asia	1990	Air pollution	90.95351532
32	Central Asia	1990	Dietary risks	182.2237294
32	Central Asia	1990	High LDL cholesterol	90.7854059
32	Central Asia	1990	High body-mass index	36.46805684
32	Central Asia	1990	High fasting plasma glucose	23.34676325
32	Central Asia	1990	High systolic blood pressure	172.0845885
32	Central Asia	1990	Impaired kidney function	65.64567628
32	Central Asia	1990	Tobacco	56.71716188
32	Central Asia	1990	other risk	39.95702968
32	Central Asia	2021	Air pollution	72.01945676
32	Central Asia	2021	Dietary risks	139.2067003
32	Central Asia	2021	High LDL cholesterol	74.61006874
32	Central Asia	2021	High body-mass index	36.47063689
32	Central Asia	2021	High fasting plasma glucose	35.22233676
32	Central Asia	2021	High systolic blood pressure	150.5367297
32	Central Asia	2021	Impaired kidney function	55.40894397
32	Central Asia	2021	Tobacco	43.21864739
32	Central Asia	2021	other risk	36.65364615
42	Central Europe	1990	Air pollution	81.16023286
42	Central Europe	1990	Dietary risks	148.6348837
42	Central Europe	1990	High LDL cholesterol	93.93231504
42	Central Europe	1990	High body-mass index	34.28291954
42	Central Europe	1990	High fasting plasma glucose	33.0494699
42	Central Europe	1990	High systolic blood pressure	166.216893

42	Central Europe	1990	Impaired kidney function	38.28041177
42	Central Europe	1990	Tobacco	64.49281166
42	Central Europe	1990	other risk	26.95570484
42	Central Europe	2021	Air pollution	25.1761889
42	Central Europe	2021	Dietary risks	68.09449119
42	Central Europe	2021	High LDL cholesterol	45.65940285
42	Central Europe	2021	High body-mass index	20.88058779
42	Central Europe	2021	High fasting plasma glucose	24.71816856
42	Central Europe	2021	High systolic blood pressure	80.37307571
42	Central Europe	2021	Impaired kidney function	20.20661484
42	Central Europe	2021	Tobacco	23.19563609
42	Central Europe	2021	other risk	15.66121889
56	Eastern Europe	1990	Air pollution	83.49833928
56	Eastern Europe	1990	Dietary risks	167.9267777
56	Eastern Europe	1990	High LDL cholesterol	111.6320493
56	Eastern Europe	1990	High body-mass index	37.30402813
56	Eastern Europe	1990	High fasting plasma glucose	23.84559866
56	Eastern Europe	1990	High systolic blood pressure	187.2076278
56	Eastern Europe	1990	Impaired kidney function	56.26857419
56	Eastern Europe	1990	Tobacco	56.78327348
56	Eastern Europe	1990	other risk	29.54960881
56	Eastern Europe	2021	Air pollution	32.44285816
56	Eastern Europe	2021	Dietary risks	115.5284036
56	Eastern Europe	2021	High LDL cholesterol	86.29239645
56	Eastern Europe	2021	High body-mass index	40.00150434
56	Eastern Europe	2021	High fasting plasma glucose	28.336619
56	Eastern Europe	2021	High systolic blood pressure	146.8864854
56	Eastern Europe	2021	Impaired kidney function	43.42868132
56	Eastern Europe	2021	Tobacco	42.90255086
56	Eastern Europe	2021	other risk	26.24976253
65	High-income Asia Pacific	1990	Air pollution	9.306810189
65	High-income Asia Pacific	1990	Dietary risks	25.98251684
65	High-income Asia Pacific	1990	High LDL cholesterol	19.72904995
65	High-income Asia Pacific	1990	High body-mass index	2.767935635
65	High-income Asia Pacific	1990	High fasting plasma glucose	9.774080968
65	High-income Asia Pacific	1990	High systolic blood pressure	39.20579657
65	High-income Asia Pacific	1990	Impaired kidney function	11.57348495
65	High-income Asia Pacific	1990	Tobacco	15.2787962
65	High-income Asia Pacific	1990	other risk	6.696920668
65	High-income Asia Pacific	2021	Air pollution	3.725534171
65	High-income Asia Pacific	2021	Dietary risks	9.64889916
65	High-income Asia Pacific	2021	High LDL cholesterol	7.941310781
65	High-income Asia Pacific	2021	High body-mass index	1.655601129
65	High-income Asia Pacific	2021	High fasting plasma glucose	4.052197153
65	High-income Asia Pacific	2021	High systolic blood pressure	12.4419897
65	High-income Asia Pacific	2021	Impaired kidney function	4.177458254
65	High-income Asia Pacific	2021	Tobacco	4.519997837
65	High-income Asia Pacific	2021	other risk	2.373689
70	Australasia	1990	Air pollution	9.638905358
70	Australasia	1990	Dietary risks	71.90474842

70	Australasia	1990	High LDL cholesterol	61.20676375
70	Australasia	1990	High body-mass index	19.18333636
70	Australasia	1990	High fasting plasma glucose	18.14658415
70	Australasia	1990	High systolic blood pressure	102.6998545
70	Australasia	1990	Impaired kidney function	26.73752664
70	Australasia	1990	Tobacco	30.78615027
70	Australasia	1990	other risk	26.08880838
70	Australasia	2021	Air pollution	3.348351099
70	Australasia	2021	Dietary risks	17.4180332
70	Australasia	2021	High LDL cholesterol	15.37632417
70	Australasia	2021	High body-mass index	7.117001782
70	Australasia	2021	High fasting plasma glucose	6.786094135
70	Australasia	2021	High systolic blood pressure	22.40704539
70	Australasia	2021	Impaired kidney function	7.323114251
70	Australasia	2021	Tobacco	5.201611034
70	Australasia	2021	other risk	5.501869692
73	Western Europe	1990	Air pollution	28.87585699
73	Western Europe	1990	Dietary risks	64.33657985
73	Western Europe	1990	High LDL cholesterol	53.24825925
73	Western Europe	1990	High body-mass index	15.78606507
73	Western Europe	1990	High fasting plasma glucose	15.67651583
73	Western Europe	1990	High systolic blood pressure	90.93476755
73	Western Europe	1990	Impaired kidney function	23.55065458
73	Western Europe	1990	Tobacco	34.67515263
73	Western Europe	1990	other risk	15.23059267
73	Western Europe	2021	Air pollution	4.504800817
73	Western Europe	2021	Dietary risks	19.18943727
73	Western Europe	2021	High LDL cholesterol	15.66961316
73	Western Europe	2021	High body-mass index	6.162729668
73	Western Europe	2021	High fasting plasma glucose	6.860181
73	Western Europe	2021	High systolic blood pressure	24.75569814
73	Western Europe	2021	Impaired kidney function	7.695633675
73	Western Europe	2021	Tobacco	7.56451181
73	Western Europe	2021	other risk	4.617580109
96	Southern Latin America	1990	Air pollution	31.91405392
96	Southern Latin America	1990	Dietary risks	79.69505149
96	Southern Latin America	1990	High LDL cholesterol	46.77568918
96	Southern Latin America	1990	High body-mass index	17.3686703
96	Southern Latin America	1990	High fasting plasma glucose	16.03894888
96	Southern Latin America	1990	High systolic blood pressure	70.68821515
96	Southern Latin America	1990	Impaired kidney function	17.4139437
96	Southern Latin America	1990	Tobacco	27.06296313
96	Southern Latin America	1990	other risk	13.32239485
96	Southern Latin America	2021	Air pollution	8.364878635
96	Southern Latin America	2021	Dietary risks	26.86319529
96	Southern Latin America	2021	High LDL cholesterol	17.77727611
96	Southern Latin America	2021	High body-mass index	8.945947389
96	Southern Latin America	2021	High fasting plasma glucose	8.804079196
96	Southern Latin America	2021	High systolic blood pressure	29.46481812
96	Southern Latin America	2021	Impaired kidney function	6.339171645

96	Southern Latin America	2021	Tobacco	8.530943887
96	Southern Latin America	2021	other risk	4.527344116
100	High-income North America	1990	Air pollution	23.23933829
100	High-income North America	1990	Dietary risks	73.60135018
100	High-income North America	1990	High LDL cholesterol	64.00431146
100	High-income North America	1990	High body-mass index	22.79906892
100	High-income North America	1990	High fasting plasma glucose	22.34543019
100	High-income North America	1990	High systolic blood pressure	98.82189727
100	High-income North America	1990	Impaired kidney function	31.23889067
100	High-income North America	1990	Tobacco	42.69103884
100	High-income North America	1990	other risk	21.93779024
100	High-income North America	2021	Air pollution	3.530221409
100	High-income North America	2021	Dietary risks	28.78311097
100	High-income North America	2021	High LDL cholesterol	22.04140762
100	High-income North America	2021	High body-mass index	13.60990171
100	High-income North America	2021	High fasting plasma glucose	16.20253781
100	High-income North America	2021	High systolic blood pressure	35.27941793
100	High-income North America	2021	Impaired kidney function	14.14164595
100	High-income North America	2021	Tobacco	13.30935252
100	High-income North America	2021	other risk	7.795056441
104	Caribbean	1990	Air pollution	45.8915141
104	Caribbean	1990	Dietary risks	74.10022515
104	Caribbean	1990	High LDL cholesterol	54.37556959
104	Caribbean	1990	High body-mass index	14.12846224
104	Caribbean	1990	High fasting plasma glucose	26.99734578
104	Caribbean	1990	High systolic blood pressure	88.69757842
104	Caribbean	1990	Impaired kidney function	25.37662797
104	Caribbean	1990	Tobacco	34.46003143
104	Caribbean	1990	other risk	18.05674776
104	Caribbean	2021	Air pollution	27.08984088
104	Caribbean	2021	Dietary risks	39.33428607
104	Caribbean	2021	High LDL cholesterol	34.05583265
104	Caribbean	2021	High body-mass index	13.18780804
104	Caribbean	2021	High fasting plasma glucose	18.49307997
104	Caribbean	2021	High systolic blood pressure	55.3892732
104	Caribbean	2021	Impaired kidney function	14.89544544
104	Caribbean	2021	Tobacco	16.41196131
104	Caribbean	2021	other risk	11.35894839
120	Andean Latin America	1990	Air pollution	39.4351305
120	Andean Latin America	1990	Dietary risks	42.17164046
120	Andean Latin America	1990	High LDL cholesterol	26.2727635
120	Andean Latin America	1990	High body-mass index	7.907379609
120	Andean Latin America	1990	High fasting plasma glucose	8.378534734
120	Andean Latin America	1990	High systolic blood pressure	29.29443425
120	Andean Latin America	1990	Impaired kidney function	11.20197381
120	Andean Latin America	1990	Tobacco	10.05902177
120	Andean Latin America	1990	other risk	8.375583528
120	Andean Latin America	2021	Air pollution	14.45912237
120	Andean Latin America	2021	Dietary risks	23.5727632
120	Andean Latin America	2021	High LDL cholesterol	17.02355745

120	Andean Latin America	2021	High body-mass index	7.724369047
120	Andean Latin America	2021	High fasting plasma glucose	8.592200541
120	Andean Latin America	2021	High systolic blood pressure	26.67033795
120	Andean Latin America	2021	Impaired kidney function	7.221406954
120	Andean Latin America	2021	Tobacco	5.134813433
120	Andean Latin America	2021	other risk	5.574022844
124	Central Latin America	1990	Air pollution	38.22720434
124	Central Latin America	1990	Dietary risks	53.27870411
124	Central Latin America	1990	High LDL cholesterol	37.12991155
124	Central Latin America	1990	High body-mass index	13.15890621
124	Central Latin America	1990	High fasting plasma glucose	18.45063807
124	Central Latin America	1990	High systolic blood pressure	61.90973492
124	Central Latin America	1990	Impaired kidney function	23.18287741
124	Central Latin America	1990	Tobacco	19.55362602
124	Central Latin America	1990	other risk	15.87257758
124	Central Latin America	2021	Air pollution	17.7480937
124	Central Latin America	2021	Dietary risks	40.67581712
124	Central Latin America	2021	High LDL cholesterol	31.69328428
124	Central Latin America	2021	High body-mass index	16.10488616
124	Central Latin America	2021	High fasting plasma glucose	18.00353567
124	Central Latin America	2021	High systolic blood pressure	54.62651209
124	Central Latin America	2021	Impaired kidney function	19.31220595
124	Central Latin America	2021	Tobacco	10.08978563
124	Central Latin America	2021	other risk	13.59148239
134	Tropical Latin America	1990	Air pollution	31.96507456
134	Tropical Latin America	1990	Dietary risks	53.58350614
134	Tropical Latin America	1990	High LDL cholesterol	47.02403861
134	Tropical Latin America	1990	High body-mass index	13.62242258
134	Tropical Latin America	1990	High fasting plasma glucose	16.66322494
134	Tropical Latin America	1990	High systolic blood pressure	69.28576669
134	Tropical Latin America	1990	Impaired kidney function	22.30665751
134	Tropical Latin America	1990	Tobacco	37.7349246
134	Tropical Latin America	1990	other risk	15.776205
134	Tropical Latin America	2021	Air pollution	8.569601509
134	Tropical Latin America	2021	Dietary risks	22.34679505
134	Tropical Latin America	2021	High LDL cholesterol	23.60889214
134	Tropical Latin America	2021	High body-mass index	10.06938078
134	Tropical Latin America	2021	High fasting plasma glucose	9.963322816
134	Tropical Latin America	2021	High systolic blood pressure	33.18813067
134	Tropical Latin America	2021	Impaired kidney function	10.05094754
134	Tropical Latin America	2021	Tobacco	11.83694138
134	Tropical Latin America	2021	other risk	6.99477273
138	North Africa and Middle East	1990	Air pollution	88.57717195
138	North Africa and Middle East	1990	Dietary risks	124.1883291
138	North Africa and Middle East	1990	High LDL cholesterol	82.73110474
138	North Africa and Middle East	1990	High body-mass index	29.305266
138	North Africa and Middle East	1990	High fasting plasma glucose	31.68311283
138	North Africa and Middle East	1990	High systolic blood pressure	137.3649307
138	North Africa and Middle East	1990	Impaired kidney function	41.36659462
138	North Africa and Middle East	1990	Tobacco	61.78230399

138	North Africa and Middle East	1990	other risk	59.01581534
138	North Africa and Middle East	2021	Air pollution	62.17563899
138	North Africa and Middle East	2021	Dietary risks	80.20983551
138	North Africa and Middle East	2021	High LDL cholesterol	61.25036955
138	North Africa and Middle East	2021	High body-mass index	35.10208668
138	North Africa and Middle East	2021	High fasting plasma glucose	38.41500868
138	North Africa and Middle East	2021	High systolic blood pressure	106.4003618
138	North Africa and Middle East	2021	Impaired kidney function	30.9248557
138	North Africa and Middle East	2021	Tobacco	38.26131889
138	North Africa and Middle East	2021	other risk	44.23712459
159	South Asia	1990	Air pollution	60.65165541
159	South Asia	1990	Dietary risks	73.14624926
159	South Asia	1990	High LDL cholesterol	37.20688105
159	South Asia	1990	High body-mass index	3.67880532
159	South Asia	1990	High fasting plasma glucose	16.17760042
159	South Asia	1990	High systolic blood pressure	63.41880633
159	South Asia	1990	Impaired kidney function	22.18103585
159	South Asia	1990	Tobacco	29.45702865
159	South Asia	1990	other risk	24.87351267
159	South Asia	2021	Air pollution	59.96643207
159	South Asia	2021	Dietary risks	72.41909191
159	South Asia	2021	High LDL cholesterol	40.43121276
159	South Asia	2021	High body-mass index	8.602709251
159	South Asia	2021	High fasting plasma glucose	24.41610077
159	South Asia	2021	High systolic blood pressure	74.12802515
159	South Asia	2021	Impaired kidney function	24.54497412
159	South Asia	2021	Tobacco	24.43192127
159	South Asia	2021	other risk	28.15795891
167	Central Sub-Saharan Africa	1990	Air pollution	60.78389723
167	Central Sub-Saharan Africa	1990	Dietary risks	53.56140897
167	Central Sub-Saharan Africa	1990	High LDL cholesterol	31.89340349
167	Central Sub-Saharan Africa	1990	High body-mass index	5.073169572
167	Central Sub-Saharan Africa	1990	High fasting plasma glucose	16.77918444
167	Central Sub-Saharan Africa	1990	High systolic blood pressure	74.94821159
167	Central Sub-Saharan Africa	1990	Impaired kidney function	25.44009941
167	Central Sub-Saharan Africa	1990	Tobacco	11.41917129
167	Central Sub-Saharan Africa	1990	other risk	8.743043107
167	Central Sub-Saharan Africa	2021	Air pollution	49.26063068
167	Central Sub-Saharan Africa	2021	Dietary risks	49.18947356
167	Central Sub-Saharan Africa	2021	High LDL cholesterol	28.07086805
167	Central Sub-Saharan Africa	2021	High body-mass index	9.481970225
167	Central Sub-Saharan Africa	2021	High fasting plasma glucose	18.04350677
167	Central Sub-Saharan Africa	2021	High systolic blood pressure	62.80218948
167	Central Sub-Saharan Africa	2021	Impaired kidney function	22.7814658
167	Central Sub-Saharan Africa	2021	Tobacco	8.829153042
167	Central Sub-Saharan Africa	2021	other risk	9.288138849
174	Eastern Sub-Saharan Africa	1990	Air pollution	31.72675891
174	Eastern Sub-Saharan Africa	1990	Dietary risks	34.18393435
174	Eastern Sub-Saharan Africa	1990	High LDL cholesterol	14.74834964
174	Eastern Sub-Saharan Africa	1990	High body-mass index	2.305349426

174	Eastern Sub-Saharan Africa	1990	High fasting plasma glucose	5.106631482
174	Eastern Sub-Saharan Africa	1990	High systolic blood pressure	27.70824031
174	Eastern Sub-Saharan Africa	1990	Impaired kidney function	7.573656986
174	Eastern Sub-Saharan Africa	1990	Tobacco	7.65387931
174	Eastern Sub-Saharan Africa	1990	other risk	8.131921865
174	Eastern Sub-Saharan Africa	2021	Air pollution	32.40139932
174	Eastern Sub-Saharan Africa	2021	Dietary risks	31.42324634
174	Eastern Sub-Saharan Africa	2021	High LDL cholesterol	16.31627237
174	Eastern Sub-Saharan Africa	2021	High body-mass index	4.362720779
174	Eastern Sub-Saharan Africa	2021	High fasting plasma glucose	6.959316596
174	Eastern Sub-Saharan Africa	2021	High systolic blood pressure	35.37222119
174	Eastern Sub-Saharan Africa	2021	Impaired kidney function	8.078821244
174	Eastern Sub-Saharan Africa	2021	Tobacco	6.512133018
174	Eastern Sub-Saharan Africa	2021	other risk	7.558165958
192	Southern Sub-Saharan Africa	1990	Air pollution	24.29046441
192	Southern Sub-Saharan Africa	1990	Dietary risks	35.17772653
192	Southern Sub-Saharan Africa	1990	High LDL cholesterol	20.12039761
192	Southern Sub-Saharan Africa	1990	High body-mass index	8.035165509
192	Southern Sub-Saharan Africa	1990	High fasting plasma glucose	7.780750064
192	Southern Sub-Saharan Africa	1990	High systolic blood pressure	43.32190462
192	Southern Sub-Saharan Africa	1990	Impaired kidney function	14.75092981
192	Southern Sub-Saharan Africa	1990	Tobacco	16.60772148
192	Southern Sub-Saharan Africa	1990	other risk	10.55680908
192	Southern Sub-Saharan Africa	2021	Air pollution	21.33534568
192	Southern Sub-Saharan Africa	2021	Dietary risks	36.64317961
192	Southern Sub-Saharan Africa	2021	High LDL cholesterol	21.79344332
192	Southern Sub-Saharan Africa	2021	High body-mass index	12.98705433
192	Southern Sub-Saharan Africa	2021	High fasting plasma glucose	11.54974272
192	Southern Sub-Saharan Africa	2021	High systolic blood pressure	48.63898059
192	Southern Sub-Saharan Africa	2021	Impaired kidney function	16.83850864
192	Southern Sub-Saharan Africa	2021	Tobacco	10.34333828
192	Southern Sub-Saharan Africa	2021	other risk	11.96749973
199	Western Sub-Saharan Africa	1990	Air pollution	48.17836502
199	Western Sub-Saharan Africa	1990	Dietary risks	42.75234966
199	Western Sub-Saharan Africa	1990	High LDL cholesterol	24.69976839
199	Western Sub-Saharan Africa	1990	High body-mass index	5.68904393
199	Western Sub-Saharan Africa	1990	High fasting plasma glucose	9.288384843
199	Western Sub-Saharan Africa	1990	High systolic blood pressure	51.20319667
199	Western Sub-Saharan Africa	1990	Impaired kidney function	19.07148752
199	Western Sub-Saharan Africa	1990	Tobacco	6.756515581
199	Western Sub-Saharan Africa	1990	other risk	8.911043734
199	Western Sub-Saharan Africa	2021	Air pollution	45.99429531
199	Western Sub-Saharan Africa	2021	Dietary risks	38.53253363
199	Western Sub-Saharan Africa	2021	High LDL cholesterol	24.99367679
199	Western Sub-Saharan Africa	2021	High body-mass index	9.839207703
199	Western Sub-Saharan Africa	2021	High fasting plasma glucose	13.48551006
199	Western Sub-Saharan Africa	2021	High systolic blood pressure	61.42341112
199	Western Sub-Saharan Africa	2021	Impaired kidney function	19.51863235
199	Western Sub-Saharan Africa	2021	Tobacco	5.735818508
199	Western Sub-Saharan Africa	2021	other risk	10.11507616

44634	High-middle SDI	1990	Air pollution	54.36788519
44634	High-middle SDI	1990	Dietary risks	95.88676613
44634	High-middle SDI	1990	High LDL cholesterol	63.11024145
44634	High-middle SDI	1990	High body-mass index	19.48423639
44634	High-middle SDI	1990	High fasting plasma glucose	18.54588426
44634	High-middle SDI	1990	High systolic blood pressure	108.7783557
44634	High-middle SDI	1990	Impaired kidney function	31.87428368
44634	High-middle SDI	1990	Tobacco	39.65098015
44634	High-middle SDI	1990	other risk	21.7229738
44634	High-middle SDI	2021	Air pollution	27.74606117
44634	High-middle SDI	2021	Dietary risks	53.79053605
44634	High-middle SDI	2021	High LDL cholesterol	39.27100326
44634	High-middle SDI	2021	High body-mass index	15.79828
44634	High-middle SDI	2021	High fasting plasma glucose	17.22862076
44634	High-middle SDI	2021	High systolic blood pressure	70.22029201
44634	High-middle SDI	2021	Impaired kidney function	19.5022668
44634	High-middle SDI	2021	Tobacco	25.05417468
44634	High-middle SDI	2021	other risk	16.77919862
44635	High SDI	1990	Air pollution	27.68463058
44635	High SDI	1990	Dietary risks	68.84410661
44635	High SDI	1990	High LDL cholesterol	55.39417263
44635	High SDI	1990	High body-mass index	17.60038419
44635	High SDI	1990	High fasting plasma glucose	18.31172965
44635	High SDI	1990	High systolic blood pressure	92.01148128
44635	High SDI	1990	Impaired kidney function	26.23143805
44635	High SDI	1990	Tobacco	36.54269366
44635	High SDI	1990	other risk	17.08965037
44635	High SDI	2021	Air pollution	6.739916589
44635	High SDI	2021	Dietary risks	23.542476
44635	High SDI	2021	High LDL cholesterol	18.46279625
44635	High SDI	2021	High body-mass index	8.849647626
44635	High SDI	2021	High fasting plasma glucose	10.39533426
44635	High SDI	2021	High systolic blood pressure	28.79770243
44635	High SDI	2021	Impaired kidney function	9.677534329
44635	High SDI	2021	Tobacco	10.39989499
44635	High SDI	2021	other risk	6.133867176
44636	Low-middle SDI	1990	Air pollution	59.50004769
44636	Low-middle SDI	1990	Dietary risks	71.27341161
44636	Low-middle SDI	1990	High LDL cholesterol	39.07496032
44636	Low-middle SDI	1990	High body-mass index	7.405427485
44636	Low-middle SDI	1990	High fasting plasma glucose	14.98110856
44636	Low-middle SDI	1990	High systolic blood pressure	66.87949711
44636	Low-middle SDI	1990	Impaired kidney function	22.52346506
44636	Low-middle SDI	1990	Tobacco	29.72384281
44636	Low-middle SDI	1990	other risk	24.23937929
44636	Low-middle SDI	2021	Air pollution	54.20870988
44636	Low-middle SDI	2021	Dietary risks	64.72472825
44636	Low-middle SDI	2021	High LDL cholesterol	39.17914123
44636	Low-middle SDI	2021	High body-mass index	12.30047113
44636	Low-middle SDI	2021	High fasting plasma glucose	22.51301871

44636	Low-middle SDI	2021	High systolic blood pressure	73.98031731
44636	Low-middle SDI	2021	Impaired kidney function	23.1847704
44636	Low-middle SDI	2021	Tobacco	25.47629387
44636	Low-middle SDI	2021	other risk	25.70519145
44637	Low SDI	1990	Air pollution	54.5612741
44637	Low SDI	1990	Dietary risks	59.36447653
44637	Low SDI	1990	High LDL cholesterol	29.57069103
44637	Low SDI	1990	High body-mass index	4.406242676
44637	Low SDI	1990	High fasting plasma glucose	12.23687435
44637	Low SDI	1990	High systolic blood pressure	55.87344293
44637	Low SDI	1990	Impaired kidney function	18.45857
44637	Low SDI	1990	Tobacco	16.99544027
44637	Low SDI	1990	other risk	18.71599092
44637	Low SDI	2021	Air pollution	52.02130915
44637	Low SDI	2021	Dietary risks	54.74700813
44637	Low SDI	2021	High LDL cholesterol	28.63958619
44637	Low SDI	2021	High body-mass index	6.866912738
44637	Low SDI	2021	High fasting plasma glucose	16.33645676
44637	Low SDI	2021	High systolic blood pressure	56.96978647
44637	Low SDI	2021	Impaired kidney function	18.32199679
44637	Low SDI	2021	Tobacco	14.23434011
44637	Low SDI	2021	other risk	19.37207066
44639	Middle SDI	1990	Air pollution	47.16397664
44639	Middle SDI	1990	Dietary risks	60.33449947
44639	Middle SDI	1990	High LDL cholesterol	36.61239277
44639	Middle SDI	1990	High body-mass index	7.512277491
44639	Middle SDI	1990	High fasting plasma glucose	14.82800206
44639	Middle SDI	1990	High systolic blood pressure	60.36144491
44639	Middle SDI	1990	Impaired kidney function	21.61949211
44639	Middle SDI	1990	Tobacco	28.39483258
44639	Middle SDI	1990	other risk	18.59545511
44639	Middle SDI	2021	Air pollution	37.1399855
44639	Middle SDI	2021	Dietary risks	49.48597324
44639	Middle SDI	2021	High LDL cholesterol	33.99495861
44639	Middle SDI	2021	High body-mass index	10.93556849
44639	Middle SDI	2021	High fasting plasma glucose	17.56996745
44639	Middle SDI	2021	High systolic blood pressure	61.54585409
44639	Middle SDI	2021	Impaired kidney function	18.81007041
44639	Middle SDI	2021	Tobacco	22.20047076
44639	Middle SDI	2021	other risk	18.80945411

Risk Factors by Region in

total_Number	proportion
367.3437835	12.26973203
367.3437835	20.43048769
367.3437835	13.74164361
367.3437835	3.750114832
367.3437835	4.650230941
367.3437835	23.34169522
367.3437835	7.220809471
367.3437835	9.111303434
367.3437835	5.483982769
246.603886	12.11676631
246.603886	18.96087209
246.603886	13.09239472
246.603886	4.748598003
246.603886	6.596733864
246.603886	23.00301897
246.603886	6.967714375
246.603886	8.084745065
246.603886	6.429156599
222.1372083	18.60725218
222.1372083	20.81569747
222.1372083	11.27979423
222.1372083	1.504504201
222.1372083	4.829956989
222.1372083	18.42537614
222.1372083	6.589151385
222.1372083	11.29016974
222.1372083	6.65809768
247.6480584	15.29630232
247.6480584	17.59469061
247.6480584	11.60672314
247.6480584	3.272830077
247.6480584	5.736563097
247.6480584	22.65467395
247.6480584	5.916713798
247.6480584	10.42953961
247.6480584	7.491963388
265.6204705	17.94463428
265.6204705	19.340808
265.6204705	12.89574981
265.6204705	1.390799211
265.6204705	4.568637605
265.6204705	22.3170356
265.6204705	7.83859166
265.6204705	10.08635014
265.6204705	3.617393694
242.2812681	13.27524502

242.2812681	16.65210034
242.2812681	13.44141938
242.2812681	2.684031041
242.2812681	6.198348443
242.2812681	25.66634167
242.2812681	8.466869448
242.2812681	9.268987393
242.2812681	4.346657259
400.5398037	17.84399744
400.5398037	21.29196744
400.5398037	14.45929039
400.5398037	4.436765026
400.5398037	7.177448547
400.5398037	16.04452838
400.5398037	6.711520305
400.5398037	9.0952178
400.5398037	2.939264678
386.5887372	16.03702083
386.5887372	20.00213326
386.5887372	13.99001962
386.5887372	5.491285082
386.5887372	8.440394571
386.5887372	18.3568754
386.5887372	6.703650389
386.5887372	8.208012854
386.5887372	2.770607994
758.181927	11.99626529
758.181927	24.03430139
758.181927	11.97409258
758.181927	4.809934864
758.181927	3.07930886
758.181927	22.69700481
758.181927	8.658301384
758.181927	7.48067975
758.181927	5.270111071
643.3471667	11.1944935
643.3471667	21.63788193
643.3471667	11.59717064
643.3471667	5.668889019
643.3471667	5.474856902
643.3471667	23.39898853
643.3471667	8.612604024
643.3471667	6.717780015
643.3471667	5.697335443
687.0056423	11.81361955
687.0056423	21.63517656
687.0056423	13.67271377
687.0056423	4.990194757
687.0056423	4.810654798
687.0056423	24.19439999

687.0056423	5.572066576
687.0056423	9.387522852
687.0056423	3.923651158
323.9653848	7.771258932
323.9653848	21.01906388
323.9653848	14.09391404
323.9653848	6.445314458
323.9653848	7.629879525
323.9653848	24.80915538
323.9653848	6.237275891
323.9653848	7.159911886
323.9653848	4.834226007
754.0158773	11.07381712
754.0158773	22.27098695
754.0158773	14.80499983
754.0158773	4.947379657
754.0158773	3.162479647
754.0158773	24.82807504
754.0158773	7.46251848
754.0158773	7.530779548
754.0158773	3.918963738
562.0692617	5.772039208
562.0692617	20.55412232
562.0692617	15.35262686
562.0692617	7.116828311
562.0692617	5.041481706
562.0692617	26.13316462
562.0692617	7.726571134
562.0692617	7.63296515
562.0692617	4.670200689
140.315392	6.63277924
140.315392	18.517225
140.315392	14.06050304
140.315392	1.97265289
140.315392	6.965793867
140.315392	27.94119449
140.315392	8.248193433
140.315392	10.88889536
140.315392	4.772762684
50.53667719	7.371941287
50.53667719	19.09286423
50.53667719	15.71395514
50.53667719	3.276038752
50.53667719	8.018329219
50.53667719	24.61972254
50.53667719	8.266190984
50.53667719	8.943994914
50.53667719	4.696962943
366.3926778	2.630758184
366.3926778	19.6250506

366.3926778	16.70523661
366.3926778	5.235731368
366.3926778	4.952769324
366.3926778	28.02999643
366.3926778	7.297505724
366.3926778	8.402501505
366.3926778	7.120450258
90.47944475	3.700676003
90.47944475	19.25081796
90.47944475	16.99427336
90.47944475	7.865876942
90.47944475	7.500150066
90.47944475	24.764791
90.47944475	8.093677267
90.47944475	5.748942258
90.47944475	6.080795154
342.3144444	8.435477223
342.3144444	18.79458518
342.3144444	15.55536441
342.3144444	4.611568495
342.3144444	4.579565976
342.3144444	26.564689
342.3144444	6.8798308
342.3144444	10.12962006
342.3144444	4.449298859
97.02018565	4.643158315
97.02018565	19.77880906
97.02018565	16.15087938
97.02018565	6.352007705
97.02018565	7.0708801
97.02018565	25.51602842
97.02018565	7.93199232
97.02018565	7.796843265
97.02018565	4.759401436
320.2799306	9.964425138
320.2799306	24.88293642
320.2799306	14.60462699
320.2799306	5.42296555
320.2799306	5.007790795
320.2799306	22.07076011
320.2799306	5.437101121
320.2799306	8.449784249
320.2799306	4.159609634
119.6176544	6.993013429
119.6176544	22.45755062
119.6176544	14.86174946
119.6176544	7.478785163
119.6176544	7.36018378
119.6176544	24.6324995
119.6176544	5.299528466

119.6176544	7.131843482
119.6176544	3.784846091
400.6791161	5.799987411
400.6791161	18.36915058
400.6791161	15.97395744
400.6791161	5.690106624
400.6791161	5.576889161
400.6791161	24.6636007
400.6791161	7.796485871
400.6791161	10.65467032
400.6791161	5.475151901
154.6926524	2.282087323
154.6926524	18.60664391
154.6926524	14.24851619
154.6926524	8.798027252
154.6926524	10.47401901
154.6926524	22.80613681
154.6926524	9.141769653
154.6926524	8.603739296
154.6926524	5.039060565
382.0841024	12.01084102
382.0841024	19.39369492
382.0841024	14.23130909
382.0841024	3.697736217
382.0841024	7.065812372
382.0841024	23.21415046
382.0841024	6.641634081
382.0841024	9.01896499
382.0841024	4.725856859
230.2164759	11.76711648
230.2164759	17.08578238
230.2164759	14.79296063
230.2164759	5.728437977
230.2164759	8.03290898
230.2164759	24.05964776
230.2164759	6.470190882
230.2164759	7.128925609
230.2164759	4.934029305
183.0964622	21.53789868
183.0964622	23.0324715
183.0964622	14.34913771
183.0964622	4.318696012
183.0964622	4.576022188
183.0964622	15.99945401
183.0964622	6.118072231
183.0964622	5.493837318
183.0964622	4.574410357
115.9725938	12.46770629
115.9725938	20.32614985
115.9725938	14.67894861

115.9725938	6.66051245
115.9725938	7.408819844
115.9725938	22.99710395
115.9725938	6.226821975
115.9725938	4.427609374
115.9725938	4.806327653
280.7641802	13.61541359
280.7641802	18.97631816
280.7641802	13.22458995
280.7641802	4.686818027
280.7641802	6.571578347
280.7641802	22.05043922
280.7641802	8.257063774
280.7641802	6.964430435
280.7641802	5.653348503
221.845603	8.000200799
221.845603	18.33519194
221.845603	14.28618997
221.845603	7.259502078
221.845603	8.11534483
221.845603	24.62366229
221.845603	8.705246212
221.845603	4.548111613
221.845603	6.12655027
307.9618206	10.3795576
307.9618206	17.39939907
307.9618206	15.26943779
307.9618206	4.423412796
307.9618206	5.410808687
307.9618206	22.49816764
307.9618206	7.243319144
307.9618206	12.25311778
307.9618206	5.122779494
136.6287846	6.272178687
136.6287846	16.35584706
136.6287846	17.27958878
136.6287846	7.369882419
136.6287846	7.292257516
136.6287846	24.29073109
136.6287846	7.356390948
136.6287846	8.663578042
136.6287846	5.119545453
656.0146292	13.50231656
656.0146292	18.93072556
656.0146292	12.61116766
656.0146292	4.467166538
656.0146292	4.829635106
656.0146292	20.93930905
656.0146292	6.305742703
656.0146292	9.417824121

656.0146292	8.996112695
496.9766004	12.51077796
496.9766004	16.13955978
496.9766004	12.32459828
496.9766004	7.063126645
496.9766004	7.729741934
496.9766004	21.4095315
496.9766004	6.222597941
496.9766004	7.698816979
496.9766004	8.901248983
330.791575	18.33530839
330.791575	22.11248859
330.791575	11.24783213
330.791575	1.112121831
330.791575	4.890572083
330.791575	19.1718324
330.791575	6.705441593
330.791575	8.905011761
330.791575	7.519391229
357.0984262	16.7926901
357.0984262	20.27986869
357.0984262	11.32214812
357.0984262	2.409058293
357.0984262	6.837358827
357.0984262	20.75842953
357.0984262	6.873447856
357.0984262	6.841789121
357.0984262	7.885209466
288.6415891	21.05860677
288.6415891	18.55637268
288.6415891	11.04948306
288.6415891	1.757601733
288.6415891	5.81315551
288.6415891	25.96583944
288.6415891	8.813733146
288.6415891	3.956176699
288.6415891	3.029030963
257.7473964	19.11197993
257.7473964	19.08437262
257.7473964	10.89084446
257.7473964	3.678784095
257.7473964	7.00046131
257.7473964	24.36579005
257.7473964	8.838679311
257.7473964	3.425506199
257.7473964	3.603582025
139.1387223	22.80224972
139.1387223	24.56823937
139.1387223	10.59974492
139.1387223	1.656871206

139.1387223	3.670172759
139.1387223	19.91411151
139.1387223	5.443241724
139.1387223	5.500898085
139.1387223	5.844470706
148.9842968	21.74819764
148.9842968	21.09164994
148.9842968	10.95167257
148.9842968	2.928309139
148.9842968	4.671174577
148.9842968	23.74224797
148.9842968	5.422599171
148.9842968	4.371019736
148.9842968	5.073129263
180.6418691	13.44675215
180.6418691	19.47373923
180.6418691	11.13828024
180.6418691	4.448119114
180.6418691	4.307279426
180.6418691	23.98220569
180.6418691	8.165842106
180.6418691	9.193727656
180.6418691	5.844054391
192.0970929	11.10654271
192.0970929	19.07534313
192.0970929	11.34501465
192.0970929	6.760671978
192.0970929	6.012450552
192.0970929	25.31999826
192.0970929	8.765623878
192.0970929	5.38443249
192.0970929	6.22992235
216.5501553	22.24813228
216.5501553	19.74247009
216.5501553	11.40602663
216.5501553	2.627125305
216.5501553	4.289253373
216.5501553	23.64495957
216.5501553	8.806960903
216.5501553	3.120069607
216.5501553	4.115002236
229.6381616	20.02902957
229.6381616	16.77967345
229.6381616	10.88393872
229.6381616	4.284657059
229.6381616	5.872503928
229.6381616	26.74791101
229.6381616	8.499733762
229.6381616	2.497763641
229.6381616	4.404788857

453.4216067	11.99058104
453.4216067	21.1473747
453.4216067	13.91866654
453.4216067	4.297156577
453.4216067	4.090207432
453.4216067	23.99055405
453.4216067	7.029723155
453.4216067	8.74483694
453.4216067	4.790899568
285.3904333	9.722141293
285.3904333	18.84805157
285.3904333	13.76044838
285.3904333	5.535672591
285.3904333	6.036859946
285.3904333	24.60499155
285.3904333	6.833539084
285.3904333	8.778911889
285.3904333	5.879383702
359.710287	7.696368878
359.710287	19.13876503
359.710287	15.39966318
359.710287	4.892933236
359.710287	5.090688344
359.710287	25.57932998
359.710287	7.292379171
359.710287	10.15892372
359.710287	4.750948469
122.9991697	5.479643975
122.9991697	19.1403536
122.9991697	15.01050479
122.9991697	7.19488404
122.9991697	8.451548324
122.9991697	23.41292426
122.9991697	7.867967204
122.9991697	8.455256256
122.9991697	4.986917548
335.6011399	17.7293938
335.6011399	21.23753561
335.6011399	11.64327401
335.6011399	2.206615712
335.6011399	4.463962359
335.6011399	19.92826876
335.6011399	6.711379187
335.6011399	8.856895662
335.6011399	7.222674898
341.2726422	15.88428229
341.2726422	18.96569494
341.2726422	11.4803053
341.2726422	3.604294516
341.2726422	6.596783899

341.2726422	21.67777552
341.2726422	6.793621149
341.2726422	7.465085306
341.2726422	7.532157071
270.1830028	20.19419191
270.1830028	21.97195083
270.1830028	10.94468961
270.1830028	1.63083637
270.1830028	4.529105912
270.1830028	20.67985119
270.1830028	6.831876841
270.1830028	6.290343986
270.1830028	6.927153347
267.509467	19.44653015
267.509467	20.46544698
267.509467	10.70600847
267.509467	2.566979335
267.509467	6.106870513
267.509467	21.29636274
267.509467	6.8491022
267.509467	5.321060323
267.509467	7.241639286
295.4223731	15.96493053
295.4223731	20.42313141
295.4223731	12.39323629
295.4223731	2.542893895
295.4223731	5.01925494
295.4223731	20.43225239
295.4223731	7.318163441
295.4223731	9.611605336
295.4223731	6.294531762
270.4923026	13.73051475
270.4923026	18.29478058
270.4923026	12.56780998
270.4923026	4.042839069
270.4923026	6.49555173
270.4923026	22.75327375
270.4923026	6.954013192
270.4923026	8.207431613
270.4923026	6.953785347

Table S15. Bayesian APC Projections of Ischemic Heart Disease Mortality: Projected Number of Deaths Across Regions, 2022-2036.

location_id	location_name	age_strat	year	Number
120	Andean Latin America	15-29 years	1990	437.5723852
120	Andean Latin America	15-29 years	1991	432.8524597
120	Andean Latin America	15-29 years	1992	435.1123115
120	Andean Latin America	15-29 years	1993	434.0437291
120	Andean Latin America	15-29 years	1994	428.3355356
120	Andean Latin America	15-29 years	1995	413.4776015
120	Andean Latin America	15-29 years	1996	398.0976282
120	Andean Latin America	15-29 years	1997	392.557617
120	Andean Latin America	15-29 years	1998	387.3624495
120	Andean Latin America	15-29 years	1999	373.6172975
120	Andean Latin America	15-29 years	2000	363.3316749
120	Andean Latin America	15-29 years	2001	357.6244551
120	Andean Latin America	15-29 years	2002	357.4634749
120	Andean Latin America	15-29 years	2003	355.8579882
120	Andean Latin America	15-29 years	2004	352.5760058
120	Andean Latin America	15-29 years	2005	351.4687075
120	Andean Latin America	15-29 years	2006	348.0258354
120	Andean Latin America	15-29 years	2007	343.8027416
120	Andean Latin America	15-29 years	2008	341.0861218
120	Andean Latin America	15-29 years	2009	340.4087225
120	Andean Latin America	15-29 years	2010	338.7477324
120	Andean Latin America	15-29 years	2011	337.9112873
120	Andean Latin America	15-29 years	2012	342.1351323
120	Andean Latin America	15-29 years	2013	349.9183353
120	Andean Latin America	15-29 years	2014	360.4573582
120	Andean Latin America	15-29 years	2015	373.2395996
120	Andean Latin America	15-29 years	2016	393.4945098
120	Andean Latin America	15-29 years	2017	420.3741169
120	Andean Latin America	15-29 years	2018	446.1842317
120	Andean Latin America	15-29 years	2019	466.4753024
120	Andean Latin America	15-29 years	2020	462.1284313
120	Andean Latin America	15-29 years	2021	456.3440877
120	Andean Latin America	15-29 years	2022	416.2594668
120	Andean Latin America	15-29 years	2023	407.6051883
120	Andean Latin America	15-29 years	2024	398.3501073
120	Andean Latin America	15-29 years	2025	389.6660584
120	Andean Latin America	15-29 years	2026	382.3203273
120	Andean Latin America	15-29 years	2027	376.7552376
120	Andean Latin America	15-29 years	2028	373.0729321
120	Andean Latin America	15-29 years	2029	371.2408093
120	Andean Latin America	15-29 years	2030	371.3687045
120	Andean Latin America	15-29 years	2031	373.6406279
120	Andean Latin America	15-29 years	2032	378.5545729
120	Andean Latin America	15-29 years	2033	387.0207085
120	Andean Latin America	15-29 years	2034	399.1978482
120	Andean Latin America	15-29 years	2035	414.880937

120	Andean Latin America	15-29 years	2036	434.0195488
120	Andean Latin America	30-44 years	1990	907.5070186
120	Andean Latin America	30-44 years	1991	910.7767634
120	Andean Latin America	30-44 years	1992	928.1225805
120	Andean Latin America	30-44 years	1993	936.3967855
120	Andean Latin America	30-44 years	1994	934.5919111
120	Andean Latin America	30-44 years	1995	913.3681996
120	Andean Latin America	30-44 years	1996	889.1232027
120	Andean Latin America	30-44 years	1997	885.0797904
120	Andean Latin America	30-44 years	1998	880.8164918
120	Andean Latin America	30-44 years	1999	854.1715016
120	Andean Latin America	30-44 years	2000	835.8454099
120	Andean Latin America	30-44 years	2001	825.118189
120	Andean Latin America	30-44 years	2002	827.7492233
120	Andean Latin America	30-44 years	2003	826.1811983
120	Andean Latin America	30-44 years	2004	820.1357265
120	Andean Latin America	30-44 years	2005	819.7648159
120	Andean Latin America	30-44 years	2006	814.0760081
120	Andean Latin America	30-44 years	2007	805.7350881
120	Andean Latin America	30-44 years	2008	802.2506501
120	Andean Latin America	30-44 years	2009	804.7409997
120	Andean Latin America	30-44 years	2010	804.6459531
120	Andean Latin America	30-44 years	2011	808.069023
120	Andean Latin America	30-44 years	2012	824.7469611
120	Andean Latin America	30-44 years	2013	850.727216
120	Andean Latin America	30-44 years	2014	885.2635246
120	Andean Latin America	30-44 years	2015	928.919509
120	Andean Latin America	30-44 years	2016	994.103035
120	Andean Latin America	30-44 years	2017	1076.534861
120	Andean Latin America	30-44 years	2018	1154.764961
120	Andean Latin America	30-44 years	2019	1215.825428
120	Andean Latin America	30-44 years	2020	1216.434468
120	Andean Latin America	30-44 years	2021	1223.896964
120	Andean Latin America	30-44 years	2022	1167.145477
120	Andean Latin America	30-44 years	2023	1164.167101
120	Andean Latin America	30-44 years	2024	1160.456895
120	Andean Latin America	30-44 years	2025	1158.08296
120	Andean Latin America	30-44 years	2026	1157.98474
120	Andean Latin America	30-44 years	2027	1160.607838
120	Andean Latin America	30-44 years	2028	1166.606667
120	Andean Latin America	30-44 years	2029	1176.091885
120	Andean Latin America	30-44 years	2030	1188.663225
120	Andean Latin America	30-44 years	2031	1204.415003
120	Andean Latin America	30-44 years	2032	1223.332483
120	Andean Latin America	30-44 years	2033	1245.354736
120	Andean Latin America	30-44 years	2034	1271.770146
120	Andean Latin America	30-44 years	2035	1304.524483
120	Andean Latin America	30-44 years	2036	1346.136604
120	Andean Latin America	45-59 years	1990	2274.710076
120	Andean Latin America	45-59 years	1991	2257.920137

120	Andean Latin America	45-59 years	1992	2289.529269
120	Andean Latin America	45-59 years	1993	2305.504796
120	Andean Latin America	45-59 years	1994	2294.21359
120	Andean Latin America	45-59 years	1995	2234.005184
120	Andean Latin America	45-59 years	1996	2184.179946
120	Andean Latin America	45-59 years	1997	2184.742915
120	Andean Latin America	45-59 years	1998	2195.740447
120	Andean Latin America	45-59 years	1999	2146.898112
120	Andean Latin America	45-59 years	2000	2124.08233
120	Andean Latin America	45-59 years	2001	2121.861679
120	Andean Latin America	45-59 years	2002	2164.444032
120	Andean Latin America	45-59 years	2003	2190.533822
120	Andean Latin America	45-59 years	2004	2204.682665
120	Andean Latin America	45-59 years	2005	2240.13301
120	Andean Latin America	45-59 years	2006	2258.485594
120	Andean Latin America	45-59 years	2007	2264.667098
120	Andean Latin America	45-59 years	2008	2284.175033
120	Andean Latin America	45-59 years	2009	2318.81965
120	Andean Latin America	45-59 years	2010	2342.333632
120	Andean Latin America	45-59 years	2011	2366.471927
120	Andean Latin America	45-59 years	2012	2424.801241
120	Andean Latin America	45-59 years	2013	2502.788023
120	Andean Latin America	45-59 years	2014	2608.233489
120	Andean Latin America	45-59 years	2015	2737.471648
120	Andean Latin America	45-59 years	2016	2937.23919
120	Andean Latin America	45-59 years	2017	3190.497421
120	Andean Latin America	45-59 years	2018	3407.860033
120	Andean Latin America	45-59 years	2019	3563.649904
120	Andean Latin America	45-59 years	2020	3544.229024
120	Andean Latin America	45-59 years	2021	3550.418694
120	Andean Latin America	45-59 years	2022	3495.138657
120	Andean Latin America	45-59 years	2023	3499.69217
120	Andean Latin America	45-59 years	2024	3507.950486
120	Andean Latin America	45-59 years	2025	3522.578652
120	Andean Latin America	45-59 years	2026	3546.278746
120	Andean Latin America	45-59 years	2027	3580.940428
120	Andean Latin America	45-59 years	2028	3627.886917
120	Andean Latin America	45-59 years	2029	3688.062831
120	Andean Latin America	45-59 years	2030	3762.945179
120	Andean Latin America	45-59 years	2031	3855.630486
120	Andean Latin America	45-59 years	2032	3968.870751
120	Andean Latin America	45-59 years	2033	4105.791678
120	Andean Latin America	45-59 years	2034	4270.32651
120	Andean Latin America	45-59 years	2035	4467.352288
120	Andean Latin America	45-59 years	2036	4704.219848
120	Andean Latin America	60-75 years	1990	5088.310975
120	Andean Latin America	60-75 years	1991	5137.44848
120	Andean Latin America	60-75 years	1992	5296.711317
120	Andean Latin America	60-75 years	1993	5401.489135
120	Andean Latin America	60-75 years	1994	5462.880193

120	Andean Latin America	60-75 years	1995	5397.705572
120	Andean Latin America	60-75 years	1996	5281.321228
120	Andean Latin America	60-75 years	1997	5334.390927
120	Andean Latin America	60-75 years	1998	5386.672114
120	Andean Latin America	60-75 years	1999	5250.997779
120	Andean Latin America	60-75 years	2000	5192.274573
120	Andean Latin America	60-75 years	2001	5171.969989
120	Andean Latin America	60-75 years	2002	5250.625704
120	Andean Latin America	60-75 years	2003	5278.91296
120	Andean Latin America	60-75 years	2004	5269.45086
120	Andean Latin America	60-75 years	2005	5322.031244
120	Andean Latin America	60-75 years	2006	5313.762635
120	Andean Latin America	60-75 years	2007	5293.010679
120	Andean Latin America	60-75 years	2008	5320.179801
120	Andean Latin America	60-75 years	2009	5387.795418
120	Andean Latin America	60-75 years	2010	5421.578685
120	Andean Latin America	60-75 years	2011	5493.169133
120	Andean Latin America	60-75 years	2012	5646.574224
120	Andean Latin America	60-75 years	2013	5855.752579
120	Andean Latin America	60-75 years	2014	6149.209403
120	Andean Latin America	60-75 years	2015	6508.224091
120	Andean Latin America	60-75 years	2016	7049.091561
120	Andean Latin America	60-75 years	2017	7740.344598
120	Andean Latin America	60-75 years	2018	8345.146954
120	Andean Latin America	60-75 years	2019	8823.348163
120	Andean Latin America	60-75 years	2020	8850.043252
120	Andean Latin America	60-75 years	2021	8860.363574
120	Andean Latin America	60-75 years	2022	9091.976807
120	Andean Latin America	60-75 years	2023	9204.895703
120	Andean Latin America	60-75 years	2024	9326.361106
120	Andean Latin America	60-75 years	2025	9459.740062
120	Andean Latin America	60-75 years	2026	9608.546137
120	Andean Latin America	60-75 years	2027	9775.122723
120	Andean Latin America	60-75 years	2028	9963.077707
120	Andean Latin America	60-75 years	2029	10179.61062
120	Andean Latin America	60-75 years	2030	10431.92106
120	Andean Latin America	60-75 years	2031	10729.76791
120	Andean Latin America	60-75 years	2032	11083.27298
120	Andean Latin America	60-75 years	2033	11504.43179
120	Andean Latin America	60-75 years	2034	12009.42627
120	Andean Latin America	60-75 years	2035	12618.64216
120	Andean Latin America	60-75 years	2036	13360.32337
120	Andean Latin America	75+ years	1990	7865.758507
120	Andean Latin America	75+ years	1991	7962.901377
120	Andean Latin America	75+ years	1992	8282.239732
120	Andean Latin America	75+ years	1993	8502.067457
120	Andean Latin America	75+ years	1994	8631.542858
120	Andean Latin America	75+ years	1995	8537.629237
120	Andean Latin America	75+ years	1996	8374.414575
120	Andean Latin America	75+ years	1997	8502.089111

120	Andean Latin America	75+ years	1998	8646.363884
120	Andean Latin America	75+ years	1999	8515.063356
120	Andean Latin America	75+ years	2000	8505.701935
120	Andean Latin America	75+ years	2001	8605.1776
120	Andean Latin America	75+ years	2002	8938.598086
120	Andean Latin America	75+ years	2003	9224.500946
120	Andean Latin America	75+ years	2004	9438.270761
120	Andean Latin America	75+ years	2005	9714.478689
120	Andean Latin America	75+ years	2006	9910.698562
120	Andean Latin America	75+ years	2007	10100.22241
120	Andean Latin America	75+ years	2008	10380.19662
120	Andean Latin America	75+ years	2009	10751.06111
120	Andean Latin America	75+ years	2010	11027.019
120	Andean Latin America	75+ years	2011	11207.3285
120	Andean Latin America	75+ years	2012	11639.8378
120	Andean Latin America	75+ years	2013	12146.56227
120	Andean Latin America	75+ years	2014	12764.30722
120	Andean Latin America	75+ years	2015	13468.5503
120	Andean Latin America	75+ years	2016	14678.78443
120	Andean Latin America	75+ years	2017	16147.99036
120	Andean Latin America	75+ years	2018	17403.32945
120	Andean Latin America	75+ years	2019	18505.57757
120	Andean Latin America	75+ years	2020	17893.64617
120	Andean Latin America	75+ years	2021	17277.10977
120	Andean Latin America	75+ years	2022	19303.02359
120	Andean Latin America	75+ years	2023	19536.82471
120	Andean Latin America	75+ years	2024	19824.36042
120	Andean Latin America	75+ years	2025	20170.94585
120	Andean Latin America	75+ years	2026	20585.82658
120	Andean Latin America	75+ years	2027	21071.27669
120	Andean Latin America	75+ years	2028	21655.80258
120	Andean Latin America	75+ years	2029	22371.50006
120	Andean Latin America	75+ years	2030	23224.52673
120	Andean Latin America	75+ years	2031	24232.36726
120	Andean Latin America	75+ years	2032	25418.45599
120	Andean Latin America	75+ years	2033	26844.3707
120	Andean Latin America	75+ years	2034	28581.40763
120	Andean Latin America	75+ years	2035	30677.4704
120	Andean Latin America	75+ years	2036	33199.46222
70	Australasia	15-29 years	1990	50.78806517
70	Australasia	15-29 years	1991	48.6036181
70	Australasia	15-29 years	1992	46.70919773
70	Australasia	15-29 years	1993	44.9370439
70	Australasia	15-29 years	1994	43.40642508
70	Australasia	15-29 years	1995	42.09875856
70	Australasia	15-29 years	1996	40.84759088
70	Australasia	15-29 years	1997	39.38789177
70	Australasia	15-29 years	1998	37.55777212
70	Australasia	15-29 years	1999	35.43639619
70	Australasia	15-29 years	2000	33.20225268

70	Australasia	15-29 years	2001	30.87454343
70	Australasia	15-29 years	2002	28.96996316
70	Australasia	15-29 years	2003	27.46223351
70	Australasia	15-29 years	2004	26.12786786
70	Australasia	15-29 years	2005	24.97133271
70	Australasia	15-29 years	2006	24.17811138
70	Australasia	15-29 years	2007	23.64520724
70	Australasia	15-29 years	2008	23.26218224
70	Australasia	15-29 years	2009	22.81607096
70	Australasia	15-29 years	2010	22.23126419
70	Australasia	15-29 years	2011	21.49986709
70	Australasia	15-29 years	2012	20.68848269
70	Australasia	15-29 years	2013	19.96036919
70	Australasia	15-29 years	2014	19.40600132
70	Australasia	15-29 years	2015	18.95577545
70	Australasia	15-29 years	2016	18.48677764
70	Australasia	15-29 years	2017	18.06278953
70	Australasia	15-29 years	2018	17.69135159
70	Australasia	15-29 years	2019	17.410539
70	Australasia	15-29 years	2020	17.15155409
70	Australasia	15-29 years	2021	16.91595123
70	Australasia	15-29 years	2022	15.8140427
70	Australasia	15-29 years	2023	15.57709236
70	Australasia	15-29 years	2024	15.31056465
70	Australasia	15-29 years	2025	15.04821176
70	Australasia	15-29 years	2026	14.81208461
70	Australasia	15-29 years	2027	14.61125107
70	Australasia	15-29 years	2028	14.45496683
70	Australasia	15-29 years	2029	14.32710556
70	Australasia	15-29 years	2030	14.22294603
70	Australasia	15-29 years	2031	14.13454185
70	Australasia	15-29 years	2032	14.04169335
70	Australasia	15-29 years	2033	13.9649193
70	Australasia	15-29 years	2034	13.91518088
70	Australasia	15-29 years	2035	13.88395484
70	Australasia	15-29 years	2036	13.8601384
70	Australasia	30-44 years	1990	625.2149417
70	Australasia	30-44 years	1991	611.7081365
70	Australasia	30-44 years	1992	590.610784
70	Australasia	30-44 years	1993	574.3668811
70	Australasia	30-44 years	1994	561.8821108
70	Australasia	30-44 years	1995	551.7691625
70	Australasia	30-44 years	1996	539.6999024
70	Australasia	30-44 years	1997	527.7750477
70	Australasia	30-44 years	1998	513.5079769
70	Australasia	30-44 years	1999	498.2081283
70	Australasia	30-44 years	2000	481.9059516
70	Australasia	30-44 years	2001	466.0407084
70	Australasia	30-44 years	2002	451.8618285
70	Australasia	30-44 years	2003	439.6091766

70	Australasia	30-44 years	2004	426.5918586
70	Australasia	30-44 years	2005	413.235091
70	Australasia	30-44 years	2006	398.6007516
70	Australasia	30-44 years	2007	387.9052314
70	Australasia	30-44 years	2008	379.6105788
70	Australasia	30-44 years	2009	371.2354232
70	Australasia	30-44 years	2010	362.0684847
70	Australasia	30-44 years	2011	354.7578202
70	Australasia	30-44 years	2012	347.1315354
70	Australasia	30-44 years	2013	340.1585465
70	Australasia	30-44 years	2014	335.233359
70	Australasia	30-44 years	2015	328.6563617
70	Australasia	30-44 years	2016	318.9589221
70	Australasia	30-44 years	2017	311.2186626
70	Australasia	30-44 years	2018	307.0040814
70	Australasia	30-44 years	2019	307.0466359
70	Australasia	30-44 years	2020	307.6328517
70	Australasia	30-44 years	2021	312.7369215
70	Australasia	30-44 years	2022	294.1329125
70	Australasia	30-44 years	2023	293.7065503
70	Australasia	30-44 years	2024	293.3200299
70	Australasia	30-44 years	2025	292.3734384
70	Australasia	30-44 years	2026	290.4985389
70	Australasia	30-44 years	2027	287.5630966
70	Australasia	30-44 years	2028	283.7877835
70	Australasia	30-44 years	2029	279.6887923
70	Australasia	30-44 years	2030	275.6378062
70	Australasia	30-44 years	2031	271.9082596
70	Australasia	30-44 years	2032	268.6745614
70	Australasia	30-44 years	2033	265.8410349
70	Australasia	30-44 years	2034	263.2414553
70	Australasia	30-44 years	2035	260.9123744
70	Australasia	30-44 years	2036	258.8688125
70	Australasia	45-59 years	1990	3246.815586
70	Australasia	45-59 years	1991	3061.90468
70	Australasia	45-59 years	1992	2967.853347
70	Australasia	45-59 years	1993	2852.254586
70	Australasia	45-59 years	1994	2764.95155
70	Australasia	45-59 years	1995	2702.893453
70	Australasia	45-59 years	1996	2630.108262
70	Australasia	45-59 years	1997	2553.573893
70	Australasia	45-59 years	1998	2475.90235
70	Australasia	45-59 years	1999	2404.091713
70	Australasia	45-59 years	2000	2318.775982
70	Australasia	45-59 years	2001	2262.951711
70	Australasia	45-59 years	2002	2214.069734
70	Australasia	45-59 years	2003	2171.623849
70	Australasia	45-59 years	2004	2122.850407
70	Australasia	45-59 years	2005	2081.145403
70	Australasia	45-59 years	2006	2044.014385

70	Australasia	45-59 years	2007	2004.474917
70	Australasia	45-59 years	2008	1967.934733
70	Australasia	45-59 years	2009	1939.246322
70	Australasia	45-59 years	2010	1916.416976
70	Australasia	45-59 years	2011	1884.674872
70	Australasia	45-59 years	2012	1845.890759
70	Australasia	45-59 years	2013	1832.672074
70	Australasia	45-59 years	2014	1830.881058
70	Australasia	45-59 years	2015	1826.789775
70	Australasia	45-59 years	2016	1811.746175
70	Australasia	45-59 years	2017	1807.079948
70	Australasia	45-59 years	2018	1806.093565
70	Australasia	45-59 years	2019	1837.05041
70	Australasia	45-59 years	2020	1830.185869
70	Australasia	45-59 years	2021	1844.27111
70	Australasia	45-59 years	2022	1813.915981
70	Australasia	45-59 years	2023	1830.308131
70	Australasia	45-59 years	2024	1844.874948
70	Australasia	45-59 years	2025	1855.733841
70	Australasia	45-59 years	2026	1863.932588
70	Australasia	45-59 years	2027	1870.156513
70	Australasia	45-59 years	2028	1872.567249
70	Australasia	45-59 years	2029	1871.114639
70	Australasia	45-59 years	2030	1867.539029
70	Australasia	45-59 years	2031	1864.660319
70	Australasia	45-59 years	2032	1865.404503
70	Australasia	45-59 years	2033	1871.008619
70	Australasia	45-59 years	2034	1879.841667
70	Australasia	45-59 years	2035	1890.381797
70	Australasia	45-59 years	2036	1901.647883
70	Australasia	60-75 years	1990	13082.18263
70	Australasia	60-75 years	1991	12509.41115
70	Australasia	60-75 years	1992	12126.35122
70	Australasia	60-75 years	1993	11674.78133
70	Australasia	60-75 years	1994	11289.53074
70	Australasia	60-75 years	1995	10774.84966
70	Australasia	60-75 years	1996	10229.77182
70	Australasia	60-75 years	1997	9624.410611
70	Australasia	60-75 years	1998	9038.395818
70	Australasia	60-75 years	1999	8457.595861
70	Australasia	60-75 years	2000	7842.671344
70	Australasia	60-75 years	2001	7360.904323
70	Australasia	60-75 years	2002	6911.00185
70	Australasia	60-75 years	2003	6457.363804
70	Australasia	60-75 years	2004	6076.968057
70	Australasia	60-75 years	2005	5693.792578
70	Australasia	60-75 years	2006	5423.5063
70	Australasia	60-75 years	2007	5333.627615
70	Australasia	60-75 years	2008	5234.168667
70	Australasia	60-75 years	2009	5125.858221

70	Australasia	60-75 years	2010	5012.155002
70	Australasia	60-75 years	2011	4896.000948
70	Australasia	60-75 years	2012	4787.04797
70	Australasia	60-75 years	2013	4747.778431
70	Australasia	60-75 years	2014	4740.305597
70	Australasia	60-75 years	2015	4736.639005
70	Australasia	60-75 years	2016	4706.535855
70	Australasia	60-75 years	2017	4722.937196
70	Australasia	60-75 years	2018	4747.091114
70	Australasia	60-75 years	2019	4873.013731
70	Australasia	60-75 years	2020	4915.919574
70	Australasia	60-75 years	2021	5096.040128
70	Australasia	60-75 years	2022	4985.437041
70	Australasia	60-75 years	2023	5084.177236
70	Australasia	60-75 years	2024	5177.262913
70	Australasia	60-75 years	2025	5269.419594
70	Australasia	60-75 years	2026	5369.464818
70	Australasia	60-75 years	2027	5484.537244
70	Australasia	60-75 years	2028	5615.387119
70	Australasia	60-75 years	2029	5755.190891
70	Australasia	60-75 years	2030	5895.464046
70	Australasia	60-75 years	2031	6033.425685
70	Australasia	60-75 years	2032	6166.996422
70	Australasia	60-75 years	2033	6289.336109
70	Australasia	60-75 years	2034	6401.608933
70	Australasia	60-75 years	2035	6507.679641
70	Australasia	60-75 years	2036	6616.508853
70	Australasia	75+ years	1990	21925.85643
70	Australasia	75+ years	1991	22122.0823
70	Australasia	75+ years	1992	22650.92604
70	Australasia	75+ years	1993	22828.23337
70	Australasia	75+ years	1994	23198.76915
70	Australasia	75+ years	1995	23355.0857
70	Australasia	75+ years	1996	23606.33435
70	Australasia	75+ years	1997	23574.65377
70	Australasia	75+ years	1998	23534.66698
70	Australasia	75+ years	1999	23394.5446
70	Australasia	75+ years	2000	23044.20191
70	Australasia	75+ years	2001	22948.83473
70	Australasia	75+ years	2002	22849.66296
70	Australasia	75+ years	2003	22580.09977
70	Australasia	75+ years	2004	22156.28291
70	Australasia	75+ years	2005	21752.35862
70	Australasia	75+ years	2006	21520.40785
70	Australasia	75+ years	2007	21468.28431
70	Australasia	75+ years	2008	21378.17183
70	Australasia	75+ years	2009	20886.51585
70	Australasia	75+ years	2010	20402.73092
70	Australasia	75+ years	2011	20002.81203
70	Australasia	75+ years	2012	19392.83591

70	Australasia	75+ years	2013	19024.29071
70	Australasia	75+ years	2014	18915.54733
70	Australasia	75+ years	2015	18582.13742
70	Australasia	75+ years	2016	18047.04468
70	Australasia	75+ years	2017	17654.06456
70	Australasia	75+ years	2018	17250.14125
70	Australasia	75+ years	2019	17411.77651
70	Australasia	75+ years	2020	16978.40592
70	Australasia	75+ years	2021	17699.92907
70	Australasia	75+ years	2022	17020.56652
70	Australasia	75+ years	2023	17060.66341
70	Australasia	75+ years	2024	17158.36999
70	Australasia	75+ years	2025	17311.35722
70	Australasia	75+ years	2026	17520.45568
70	Australasia	75+ years	2027	17759.50628
70	Australasia	75+ years	2028	18061.10356
70	Australasia	75+ years	2029	18446.99266
70	Australasia	75+ years	2030	18892.08542
70	Australasia	75+ years	2031	19383.69271
70	Australasia	75+ years	2032	19897.09223
70	Australasia	75+ years	2033	20472.70361
70	Australasia	75+ years	2034	21134.08329
70	Australasia	75+ years	2035	21846.4636
70	Australasia	75+ years	2036	22592.05669
104	Caribbean	15-29 years	1990	282.9875607
104	Caribbean	15-29 years	1991	282.5771504
104	Caribbean	15-29 years	1992	283.5088352
104	Caribbean	15-29 years	1993	284.7782061
104	Caribbean	15-29 years	1994	283.9716951
104	Caribbean	15-29 years	1995	281.950142
104	Caribbean	15-29 years	1996	279.2639625
104	Caribbean	15-29 years	1997	276.3612733
104	Caribbean	15-29 years	1998	273.4645908
104	Caribbean	15-29 years	1999	269.7003088
104	Caribbean	15-29 years	2000	264.4802033
104	Caribbean	15-29 years	2001	261.0552806
104	Caribbean	15-29 years	2002	259.6302275
104	Caribbean	15-29 years	2003	263.1155916
104	Caribbean	15-29 years	2004	268.9778206
104	Caribbean	15-29 years	2005	274.0039751
104	Caribbean	15-29 years	2006	275.9994581
104	Caribbean	15-29 years	2007	277.9629982
104	Caribbean	15-29 years	2008	280.8393459
104	Caribbean	15-29 years	2009	284.0982493
104	Caribbean	15-29 years	2010	285.5059649
104	Caribbean	15-29 years	2011	287.312114
104	Caribbean	15-29 years	2012	292.2632696
104	Caribbean	15-29 years	2013	300.0419217
104	Caribbean	15-29 years	2014	308.6650862
104	Caribbean	15-29 years	2015	317.0823324

104	Caribbean	15-29 years	2016	324.4304423
104	Caribbean	15-29 years	2017	330.4382348
104	Caribbean	15-29 years	2018	334.6172309
104	Caribbean	15-29 years	2019	338.6558566
104	Caribbean	15-29 years	2020	339.6572075
104	Caribbean	15-29 years	2021	341.8994776
104	Caribbean	15-29 years	2022	339.7950694
104	Caribbean	15-29 years	2023	341.2146654
104	Caribbean	15-29 years	2024	342.8751886
104	Caribbean	15-29 years	2025	344.7334072
104	Caribbean	15-29 years	2026	346.7503923
104	Caribbean	15-29 years	2027	348.840796
104	Caribbean	15-29 years	2028	350.9085076
104	Caribbean	15-29 years	2029	353.081151
104	Caribbean	15-29 years	2030	355.7597836
104	Caribbean	15-29 years	2031	359.3583486
104	Caribbean	15-29 years	2032	364.3384794
104	Caribbean	15-29 years	2033	370.784092
104	Caribbean	15-29 years	2034	378.3856261
104	Caribbean	15-29 years	2035	387.2133936
104	Caribbean	15-29 years	2036	397.2614162
104	Caribbean	30-44 years	1990	1384.855845
104	Caribbean	30-44 years	1991	1395.86762
104	Caribbean	30-44 years	1992	1423.561353
104	Caribbean	30-44 years	1993	1452.475356
104	Caribbean	30-44 years	1994	1476.078783
104	Caribbean	30-44 years	1995	1498.918557
104	Caribbean	30-44 years	1996	1507.177475
104	Caribbean	30-44 years	1997	1513.837803
104	Caribbean	30-44 years	1998	1516.263301
104	Caribbean	30-44 years	1999	1511.305951
104	Caribbean	30-44 years	2000	1494.782333
104	Caribbean	30-44 years	2001	1494.154664
104	Caribbean	30-44 years	2002	1502.818763
104	Caribbean	30-44 years	2003	1540.202107
104	Caribbean	30-44 years	2004	1583.027313
104	Caribbean	30-44 years	2005	1616.408743
104	Caribbean	30-44 years	2006	1620.204879
104	Caribbean	30-44 years	2007	1617.590938
104	Caribbean	30-44 years	2008	1606.15255
104	Caribbean	30-44 years	2009	1594.330168
104	Caribbean	30-44 years	2010	1573.629887
104	Caribbean	30-44 years	2011	1561.813803
104	Caribbean	30-44 years	2012	1575.512617
104	Caribbean	30-44 years	2013	1609.246108
104	Caribbean	30-44 years	2014	1649.692208
104	Caribbean	30-44 years	2015	1688.296748
104	Caribbean	30-44 years	2016	1719.245841
104	Caribbean	30-44 years	2017	1748.963723
104	Caribbean	30-44 years	2018	1775.294217

104	Caribbean	30-44 years	2019	1804.457025
104	Caribbean	30-44 years	2020	1812.858014
104	Caribbean	30-44 years	2021	1841.231589
104	Caribbean	30-44 years	2022	1888.822704
104	Caribbean	30-44 years	2023	1919.569477
104	Caribbean	30-44 years	2024	1953.724581
104	Caribbean	30-44 years	2025	1989.292337
104	Caribbean	30-44 years	2026	2024.97571
104	Caribbean	30-44 years	2027	2058.596225
104	Caribbean	30-44 years	2028	2090.767549
104	Caribbean	30-44 years	2029	2123.50273
104	Caribbean	30-44 years	2030	2156.646537
104	Caribbean	30-44 years	2031	2190.497164
104	Caribbean	30-44 years	2032	2224.449435
104	Caribbean	30-44 years	2033	2255.965448
104	Caribbean	30-44 years	2034	2285.083536
104	Caribbean	30-44 years	2035	2315.16248
104	Caribbean	30-44 years	2036	2350.222213
104	Caribbean	45-59 years	1990	5626.64722
104	Caribbean	45-59 years	1991	5576.281489
104	Caribbean	45-59 years	1992	5621.55334
104	Caribbean	45-59 years	1993	5700.465779
104	Caribbean	45-59 years	1994	5769.249711
104	Caribbean	45-59 years	1995	5813.528141
104	Caribbean	45-59 years	1996	5871.488736
104	Caribbean	45-59 years	1997	5891.233659
104	Caribbean	45-59 years	1998	5921.700496
104	Caribbean	45-59 years	1999	5925.111006
104	Caribbean	45-59 years	2000	5868.929596
104	Caribbean	45-59 years	2001	5901.488374
104	Caribbean	45-59 years	2002	5899.965167
104	Caribbean	45-59 years	2003	6043.681262
104	Caribbean	45-59 years	2004	6205.633446
104	Caribbean	45-59 years	2005	6402.222906
104	Caribbean	45-59 years	2006	6464.229801
104	Caribbean	45-59 years	2007	6527.561576
104	Caribbean	45-59 years	2008	6581.426348
104	Caribbean	45-59 years	2009	6688.186743
104	Caribbean	45-59 years	2010	6713.048403
104	Caribbean	45-59 years	2011	6720.502504
104	Caribbean	45-59 years	2012	6879.235583
104	Caribbean	45-59 years	2013	7109.766884
104	Caribbean	45-59 years	2014	7344.995387
104	Caribbean	45-59 years	2015	7590.921115
104	Caribbean	45-59 years	2016	7810.894724
104	Caribbean	45-59 years	2017	8045.659559
104	Caribbean	45-59 years	2018	8206.840811
104	Caribbean	45-59 years	2019	8390.827127
104	Caribbean	45-59 years	2020	8535.088877
104	Caribbean	45-59 years	2021	8525.041297

104	Caribbean	45-59 years	2022	8679.889285
104	Caribbean	45-59 years	2023	8734.457451
104	Caribbean	45-59 years	2024	8763.813325
104	Caribbean	45-59 years	2025	8786.485814
104	Caribbean	45-59 years	2026	8821.368156
104	Caribbean	45-59 years	2027	8887.315399
104	Caribbean	45-59 years	2028	8981.396109
104	Caribbean	45-59 years	2029	9091.473248
104	Caribbean	45-59 years	2030	9220.535601
104	Caribbean	45-59 years	2031	9373.864755
104	Caribbean	45-59 years	2032	9556.750288
104	Caribbean	45-59 years	2033	9768.181718
104	Caribbean	45-59 years	2034	10004.39846
104	Caribbean	45-59 years	2035	10267.7814
104	Caribbean	45-59 years	2036	10564.45041
104	Caribbean	60-75 years	1990	14742.87069
104	Caribbean	60-75 years	1991	14492.9517
104	Caribbean	60-75 years	1992	14506.8968
104	Caribbean	60-75 years	1993	14703.31286
104	Caribbean	60-75 years	1994	14752.28666
104	Caribbean	60-75 years	1995	14737.37933
104	Caribbean	60-75 years	1996	14746.94806
104	Caribbean	60-75 years	1997	14610.89823
104	Caribbean	60-75 years	1998	14673.32701
104	Caribbean	60-75 years	1999	14561.83319
104	Caribbean	60-75 years	2000	14234.49855
104	Caribbean	60-75 years	2001	14244.4779
104	Caribbean	60-75 years	2002	14081.2689
104	Caribbean	60-75 years	2003	14381.49764
104	Caribbean	60-75 years	2004	14664.16477
104	Caribbean	60-75 years	2005	14998.60912
104	Caribbean	60-75 years	2006	14980.12703
104	Caribbean	60-75 years	2007	15059.52146
104	Caribbean	60-75 years	2008	15181.4268
104	Caribbean	60-75 years	2009	15345.52723
104	Caribbean	60-75 years	2010	15306.38383
104	Caribbean	60-75 years	2011	15253.34937
104	Caribbean	60-75 years	2012	15496.59958
104	Caribbean	60-75 years	2013	15972.36465
104	Caribbean	60-75 years	2014	16430.07923
104	Caribbean	60-75 years	2015	17005.58671
104	Caribbean	60-75 years	2016	17421.62552
104	Caribbean	60-75 years	2017	18034.95062
104	Caribbean	60-75 years	2018	18403.95128
104	Caribbean	60-75 years	2019	18859.80381
104	Caribbean	60-75 years	2020	19405.16449
104	Caribbean	60-75 years	2021	19372.10999
104	Caribbean	60-75 years	2022	20144.26627
104	Caribbean	60-75 years	2023	20563.57855
104	Caribbean	60-75 years	2024	21004.71206

104	Caribbean	60-75 years	2025	21453.3882
104	Caribbean	60-75 years	2026	21897.32643
104	Caribbean	60-75 years	2027	22340.56111
104	Caribbean	60-75 years	2028	22815.22925
104	Caribbean	60-75 years	2029	23338.10381
104	Caribbean	60-75 years	2030	23887.02499
104	Caribbean	60-75 years	2031	24443.80936
104	Caribbean	60-75 years	2032	25001.76743
104	Caribbean	60-75 years	2033	25591.76636
104	Caribbean	60-75 years	2034	26239.89326
104	Caribbean	60-75 years	2035	26927.17111
104	Caribbean	60-75 years	2036	27638.60895
104	Caribbean	75+ years	1990	21968.99582
104	Caribbean	75+ years	1991	22043.05206
104	Caribbean	75+ years	1992	22643.71114
104	Caribbean	75+ years	1993	23277.45977
104	Caribbean	75+ years	1994	23341.07174
104	Caribbean	75+ years	1995	23304.86805
104	Caribbean	75+ years	1996	23256.66279
104	Caribbean	75+ years	1997	22777.36124
104	Caribbean	75+ years	1998	22661.51508
104	Caribbean	75+ years	1999	22400.1663
104	Caribbean	75+ years	2000	21868.8605
104	Caribbean	75+ years	2001	21832.44788
104	Caribbean	75+ years	2002	21536.69207
104	Caribbean	75+ years	2003	22304.2263
104	Caribbean	75+ years	2004	23100.91201
104	Caribbean	75+ years	2005	23550.40483
104	Caribbean	75+ years	2006	23180.03005
104	Caribbean	75+ years	2007	23212.75717
104	Caribbean	75+ years	2008	23576.69413
104	Caribbean	75+ years	2009	23853.69969
104	Caribbean	75+ years	2010	23871.72892
104	Caribbean	75+ years	2011	23632.53306
104	Caribbean	75+ years	2012	23867.72929
104	Caribbean	75+ years	2013	24479.10609
104	Caribbean	75+ years	2014	24859.52796
104	Caribbean	75+ years	2015	25543.69169
104	Caribbean	75+ years	2016	25974.01853
104	Caribbean	75+ years	2017	26669.07633
104	Caribbean	75+ years	2018	27065.18257
104	Caribbean	75+ years	2019	27685.63811
104	Caribbean	75+ years	2020	28328.86631
104	Caribbean	75+ years	2021	28428.6086
104	Caribbean	75+ years	2022	29542.19929
104	Caribbean	75+ years	2023	30086.25639
104	Caribbean	75+ years	2024	30739.45146
104	Caribbean	75+ years	2025	31472.7827
104	Caribbean	75+ years	2026	32249.47198
104	Caribbean	75+ years	2027	33046.63584

104	Caribbean	75+ years	2028	33885.06484
104	Caribbean	75+ years	2029	34802.55523
104	Caribbean	75+ years	2030	35789.44088
104	Caribbean	75+ years	2031	36830.55136
104	Caribbean	75+ years	2032	37943.69124
104	Caribbean	75+ years	2033	39162.07769
104	Caribbean	75+ years	2034	40507.32699
104	Caribbean	75+ years	2035	41973.45303
104	Caribbean	75+ years	2036	43553.91886
32	Central Asia	15-29 years	1990	981.1072618
32	Central Asia	15-29 years	1991	1028.889115
32	Central Asia	15-29 years	1992	1122.434626
32	Central Asia	15-29 years	1993	1200.996888
32	Central Asia	15-29 years	1994	1236.272666
32	Central Asia	15-29 years	1995	1209.508881
32	Central Asia	15-29 years	1996	1178.372738
32	Central Asia	15-29 years	1997	1140.056683
32	Central Asia	15-29 years	1998	1135.073837
32	Central Asia	15-29 years	1999	1113.149041
32	Central Asia	15-29 years	2000	1091.901523
32	Central Asia	15-29 years	2001	1066.3284
32	Central Asia	15-29 years	2002	1050.418163
32	Central Asia	15-29 years	2003	1043.356634
32	Central Asia	15-29 years	2004	1036.267629
32	Central Asia	15-29 years	2005	1058.726866
32	Central Asia	15-29 years	2006	1095.603231
32	Central Asia	15-29 years	2007	1097.587276
32	Central Asia	15-29 years	2008	1060.03768
32	Central Asia	15-29 years	2009	1002.962409
32	Central Asia	15-29 years	2010	969.260987
32	Central Asia	15-29 years	2011	956.7285948
32	Central Asia	15-29 years	2012	954.4148339
32	Central Asia	15-29 years	2013	933.3801241
32	Central Asia	15-29 years	2014	912.1733461
32	Central Asia	15-29 years	2015	889.1373637
32	Central Asia	15-29 years	2016	859.8078393
32	Central Asia	15-29 years	2017	834.2448168
32	Central Asia	15-29 years	2018	805.6885547
32	Central Asia	15-29 years	2019	776.6678459
32	Central Asia	15-29 years	2020	751.1091049
32	Central Asia	15-29 years	2021	735.3555904
32	Central Asia	15-29 years	2022	723.6050536
32	Central Asia	15-29 years	2023	709.3519466
32	Central Asia	15-29 years	2024	695.6571412
32	Central Asia	15-29 years	2025	683.9214047
32	Central Asia	15-29 years	2026	675.7388187
32	Central Asia	15-29 years	2027	672.5404121
32	Central Asia	15-29 years	2028	675.6990224
32	Central Asia	15-29 years	2029	684.6711896
32	Central Asia	15-29 years	2030	698.3814922

32	Central Asia	15-29 years	2031	715.743449
32	Central Asia	15-29 years	2032	735.6688023
32	Central Asia	15-29 years	2033	760.7690503
32	Central Asia	15-29 years	2034	792.5502552
32	Central Asia	15-29 years	2035	829.3249756
32	Central Asia	15-29 years	2036	869.306289
32	Central Asia	30-44 years	1990	4803.168518
32	Central Asia	30-44 years	1991	5485.730596
32	Central Asia	30-44 years	1992	6230.499121
32	Central Asia	30-44 years	1993	6985.099322
32	Central Asia	30-44 years	1994	7450.897672
32	Central Asia	30-44 years	1995	7784.18566
32	Central Asia	30-44 years	1996	7757.056217
32	Central Asia	30-44 years	1997	7501.613281
32	Central Asia	30-44 years	1998	7453.661193
32	Central Asia	30-44 years	1999	7328.837722
32	Central Asia	30-44 years	2000	7500.091253
32	Central Asia	30-44 years	2001	7454.844369
32	Central Asia	30-44 years	2002	7320.600931
32	Central Asia	30-44 years	2003	7182.787718
32	Central Asia	30-44 years	2004	6983.589911
32	Central Asia	30-44 years	2005	7111.140951
32	Central Asia	30-44 years	2006	7128.114418
32	Central Asia	30-44 years	2007	6950.613372
32	Central Asia	30-44 years	2008	6538.766513
32	Central Asia	30-44 years	2009	6003.224954
32	Central Asia	30-44 years	2010	5752.104613
32	Central Asia	30-44 years	2011	5607.058425
32	Central Asia	30-44 years	2012	5503.524851
32	Central Asia	30-44 years	2013	5321.153227
32	Central Asia	30-44 years	2014	5113.660955
32	Central Asia	30-44 years	2015	4969.340484
32	Central Asia	30-44 years	2016	4841.331994
32	Central Asia	30-44 years	2017	4747.168273
32	Central Asia	30-44 years	2018	4738.918379
32	Central Asia	30-44 years	2019	4692.190797
32	Central Asia	30-44 years	2020	4617.049839
32	Central Asia	30-44 years	2021	4680.177034
32	Central Asia	30-44 years	2022	4691.43753
32	Central Asia	30-44 years	2023	4679.046737
32	Central Asia	30-44 years	2024	4678.035976
32	Central Asia	30-44 years	2025	4683.614346
32	Central Asia	30-44 years	2026	4691.196365
32	Central Asia	30-44 years	2027	4693.076239
32	Central Asia	30-44 years	2028	4687.784802
32	Central Asia	30-44 years	2029	4680.672959
32	Central Asia	30-44 years	2030	4676.009052
32	Central Asia	30-44 years	2031	4677.651891
32	Central Asia	30-44 years	2032	4687.43875
32	Central Asia	30-44 years	2033	4693.826794

32	Central Asia	30-44 years	2034	4693.271849
32	Central Asia	30-44 years	2035	4700.947166
32	Central Asia	30-44 years	2036	4733.641539
32	Central Asia	45-59 years	1990	18327.08736
32	Central Asia	45-59 years	1991	19014.48031
32	Central Asia	45-59 years	1992	20095.40791
32	Central Asia	45-59 years	1993	21765.50353
32	Central Asia	45-59 years	1994	22954.26607
32	Central Asia	45-59 years	1995	23896.25715
32	Central Asia	45-59 years	1996	23589.87748
32	Central Asia	45-59 years	1997	22136.65307
32	Central Asia	45-59 years	1998	20934.50312
32	Central Asia	45-59 years	1999	19742.14352
32	Central Asia	45-59 years	2000	19657.84666
32	Central Asia	45-59 years	2001	19423.10815
32	Central Asia	45-59 years	2002	19628.08133
32	Central Asia	45-59 years	2003	20385.68635
32	Central Asia	45-59 years	2004	21260.39891
32	Central Asia	45-59 years	2005	23120.53076
32	Central Asia	45-59 years	2006	24792.34449
32	Central Asia	45-59 years	2007	25462.02742
32	Central Asia	45-59 years	2008	25329.38495
32	Central Asia	45-59 years	2009	24621.78516
32	Central Asia	45-59 years	2010	24926.78465
32	Central Asia	45-59 years	2011	25020.26114
32	Central Asia	45-59 years	2012	25182.10962
32	Central Asia	45-59 years	2013	24806.62672
32	Central Asia	45-59 years	2014	24628.41228
32	Central Asia	45-59 years	2015	24618.53946
32	Central Asia	45-59 years	2016	24352.67015
32	Central Asia	45-59 years	2017	23955.54589
32	Central Asia	45-59 years	2018	23589.27932
32	Central Asia	45-59 years	2019	22845.01695
32	Central Asia	45-59 years	2020	22057.16933
32	Central Asia	45-59 years	2021	21817.69705
32	Central Asia	45-59 years	2022	21105.5947
32	Central Asia	45-59 years	2023	20794.34032
32	Central Asia	45-59 years	2024	20470.72025
32	Central Asia	45-59 years	2025	20179.37419
32	Central Asia	45-59 years	2026	19961.92389
32	Central Asia	45-59 years	2027	19843.73089
32	Central Asia	45-59 years	2028	19823.6971
32	Central Asia	45-59 years	2029	19875.01314
32	Central Asia	45-59 years	2030	19995.12219
32	Central Asia	45-59 years	2031	20188.85097
32	Central Asia	45-59 years	2032	20449.21816
32	Central Asia	45-59 years	2033	20800.66611
32	Central Asia	45-59 years	2034	21263.29297
32	Central Asia	45-59 years	2035	21838.61207
32	Central Asia	45-59 years	2036	22532.24002

32	Central Asia	60-75 years	1990	39649.52144
32	Central Asia	60-75 years	1991	44034.87849
32	Central Asia	60-75 years	1992	48766.85519
32	Central Asia	60-75 years	1993	55005.50785
32	Central Asia	60-75 years	1994	59185.93511
32	Central Asia	60-75 years	1995	62837.53647
32	Central Asia	60-75 years	1996	65279.17377
32	Central Asia	60-75 years	1997	66524.26704
32	Central Asia	60-75 years	1998	68380.41425
32	Central Asia	60-75 years	1999	69438.24121
32	Central Asia	60-75 years	2000	71276.65361
32	Central Asia	60-75 years	2001	71689.05016
32	Central Asia	60-75 years	2002	71716.44604
32	Central Asia	60-75 years	2003	70538.00971
32	Central Asia	60-75 years	2004	66918.12239
32	Central Asia	60-75 years	2005	66204.82881
32	Central Asia	60-75 years	2006	64567.20071
32	Central Asia	60-75 years	2007	63662.6242
32	Central Asia	60-75 years	2008	61655.78813
32	Central Asia	60-75 years	2009	58169.24892
32	Central Asia	60-75 years	2010	56347.08441
32	Central Asia	60-75 years	2011	55374.55885
32	Central Asia	60-75 years	2012	54302.97591
32	Central Asia	60-75 years	2013	52157.82413
32	Central Asia	60-75 years	2014	50758.78389
32	Central Asia	60-75 years	2015	50468.62767
32	Central Asia	60-75 years	2016	50701.39675
32	Central Asia	60-75 years	2017	51397.21612
32	Central Asia	60-75 years	2018	52550.10873
32	Central Asia	60-75 years	2019	54287.90485
32	Central Asia	60-75 years	2020	57722.71968
32	Central Asia	60-75 years	2021	61014.21452
32	Central Asia	60-75 years	2022	63184.41695
32	Central Asia	60-75 years	2023	65317.92423
32	Central Asia	60-75 years	2024	67243.11874
32	Central Asia	60-75 years	2025	69035.93167
32	Central Asia	60-75 years	2026	70702.22895
32	Central Asia	60-75 years	2027	72198.20961
32	Central Asia	60-75 years	2028	73545.14631
32	Central Asia	60-75 years	2029	74806.4933
32	Central Asia	60-75 years	2030	76054.01028
32	Central Asia	60-75 years	2031	77309.81628
32	Central Asia	60-75 years	2032	78539.22352
32	Central Asia	60-75 years	2033	79613.29163
32	Central Asia	60-75 years	2034	80569.77406
32	Central Asia	60-75 years	2035	81701.22669
32	Central Asia	60-75 years	2036	83259.45755
32	Central Asia	75+ years	1990	64871.20219
32	Central Asia	75+ years	1991	68236.19588
32	Central Asia	75+ years	1992	72171.1405

32	Central Asia	75+ years	1993	74749.45178
32	Central Asia	75+ years	1994	74830.98231
32	Central Asia	75+ years	1995	74277.2541
32	Central Asia	75+ years	1996	73425.11763
32	Central Asia	75+ years	1997	70706.43292
32	Central Asia	75+ years	1998	69751.35868
32	Central Asia	75+ years	1999	68849.07694
32	Central Asia	75+ years	2000	69277.65598
32	Central Asia	75+ years	2001	70110.23556
32	Central Asia	75+ years	2002	73030.53207
32	Central Asia	75+ years	2003	75955.74128
32	Central Asia	75+ years	2004	76985.41808
32	Central Asia	75+ years	2005	79542.9295
32	Central Asia	75+ years	2006	81943.01781
32	Central Asia	75+ years	2007	83853.10131
32	Central Asia	75+ years	2008	86127.99321
32	Central Asia	75+ years	2009	84027.11626
32	Central Asia	75+ years	2010	84091.17817
32	Central Asia	75+ years	2011	86410.54544
32	Central Asia	75+ years	2012	90107.7702
32	Central Asia	75+ years	2013	92863.23049
32	Central Asia	75+ years	2014	95568.93741
32	Central Asia	75+ years	2015	97195.97584
32	Central Asia	75+ years	2016	97207.44369
32	Central Asia	75+ years	2017	95860.61674
32	Central Asia	75+ years	2018	92447.4413
32	Central Asia	75+ years	2019	88996.86853
32	Central Asia	75+ years	2020	87629.59164
32	Central Asia	75+ years	2021	83751.80901
32	Central Asia	75+ years	2022	75884.59262
32	Central Asia	75+ years	2023	75069.71745
32	Central Asia	75+ years	2024	75243.4628
32	Central Asia	75+ years	2025	76165.99652
32	Central Asia	75+ years	2026	77519.9021
32	Central Asia	75+ years	2027	79103.27184
32	Central Asia	75+ years	2028	81448.20163
32	Central Asia	75+ years	2029	84971.77673
32	Central Asia	75+ years	2030	89425.08559
32	Central Asia	75+ years	2031	94455.71447
32	Central Asia	75+ years	2032	99784.77832
32	Central Asia	75+ years	2033	105953.1532
32	Central Asia	75+ years	2034	113492.3023
32	Central Asia	75+ years	2035	122228.2644
32	Central Asia	75+ years	2036	131852.4568
42	Central Europe	15-29 years	1990	696.224877
42	Central Europe	15-29 years	1991	673.7353838
42	Central Europe	15-29 years	1992	651.1124815
42	Central Europe	15-29 years	1993	630.6554502
42	Central Europe	15-29 years	1994	611.9568051
42	Central Europe	15-29 years	1995	599.6601769

42	Central Europe	15-29 years	1996	585.3649758
42	Central Europe	15-29 years	1997	564.6349866
42	Central Europe	15-29 years	1998	526.8553823
42	Central Europe	15-29 years	1999	491.0189252
42	Central Europe	15-29 years	2000	457.3965285
42	Central Europe	15-29 years	2001	433.8137833
42	Central Europe	15-29 years	2002	415.9013357
42	Central Europe	15-29 years	2003	400.0919339
42	Central Europe	15-29 years	2004	384.332202
42	Central Europe	15-29 years	2005	368.5627523
42	Central Europe	15-29 years	2006	351.5871634
42	Central Europe	15-29 years	2007	335.4153803
42	Central Europe	15-29 years	2008	320.3615291
42	Central Europe	15-29 years	2009	304.723384
42	Central Europe	15-29 years	2010	288.4029594
42	Central Europe	15-29 years	2011	272.3805904
42	Central Europe	15-29 years	2012	258.5002173
42	Central Europe	15-29 years	2013	246.345381
42	Central Europe	15-29 years	2014	235.6305948
42	Central Europe	15-29 years	2015	225.8244493
42	Central Europe	15-29 years	2016	215.509544
42	Central Europe	15-29 years	2017	207.2081988
42	Central Europe	15-29 years	2018	198.1422355
42	Central Europe	15-29 years	2019	188.044288
42	Central Europe	15-29 years	2020	177.6680155
42	Central Europe	15-29 years	2021	168.9646992
42	Central Europe	15-29 years	2022	159.4168452
42	Central Europe	15-29 years	2023	152.8003026
42	Central Europe	15-29 years	2024	146.8398401
42	Central Europe	15-29 years	2025	141.5057631
42	Central Europe	15-29 years	2026	136.7721378
42	Central Europe	15-29 years	2027	132.5916939
42	Central Europe	15-29 years	2028	129.0711995
42	Central Europe	15-29 years	2029	126.2030232
42	Central Europe	15-29 years	2030	123.8352734
42	Central Europe	15-29 years	2031	121.8194523
42	Central Europe	15-29 years	2032	119.9398272
42	Central Europe	15-29 years	2033	118.3357015
42	Central Europe	15-29 years	2034	117.1633001
42	Central Europe	15-29 years	2035	116.226781
42	Central Europe	15-29 years	2036	115.3311874
42	Central Europe	30-44 years	1990	9570.283722
42	Central Europe	30-44 years	1991	9958.857685
42	Central Europe	30-44 years	1992	9920.809848
42	Central Europe	30-44 years	1993	9619.080578
42	Central Europe	30-44 years	1994	9329.064907
42	Central Europe	30-44 years	1995	9150.873623
42	Central Europe	30-44 years	1996	8677.793865
42	Central Europe	30-44 years	1997	8404.394979
42	Central Europe	30-44 years	1998	7774.399889

42	Central Europe	30-44 years	1999	7210.082746
42	Central Europe	30-44 years	2000	6519.684178
42	Central Europe	30-44 years	2001	6016.947746
42	Central Europe	30-44 years	2002	5572.361837
42	Central Europe	30-44 years	2003	5215.696061
42	Central Europe	30-44 years	2004	4908.090224
42	Central Europe	30-44 years	2005	4648.074597
42	Central Europe	30-44 years	2006	4405.217362
42	Central Europe	30-44 years	2007	4197.24459
42	Central Europe	30-44 years	2008	4036.468047
42	Central Europe	30-44 years	2009	3907.330506
42	Central Europe	30-44 years	2010	3766.808781
42	Central Europe	30-44 years	2011	3587.821288
42	Central Europe	30-44 years	2012	3448.583711
42	Central Europe	30-44 years	2013	3249.365663
42	Central Europe	30-44 years	2014	3148.254903
42	Central Europe	30-44 years	2015	3075.250393
42	Central Europe	30-44 years	2016	2977.273457
42	Central Europe	30-44 years	2017	2922.859183
42	Central Europe	30-44 years	2018	2910.134905
42	Central Europe	30-44 years	2019	2833.566904
42	Central Europe	30-44 years	2020	2686.494308
42	Central Europe	30-44 years	2021	2617.03384
42	Central Europe	30-44 years	2022	2492.529048
42	Central Europe	30-44 years	2023	2421.737601
42	Central Europe	30-44 years	2024	2348.950483
42	Central Europe	30-44 years	2025	2274.420037
42	Central Europe	30-44 years	2026	2202.32194
42	Central Europe	30-44 years	2027	2134.499618
42	Central Europe	30-44 years	2028	2067.886104
42	Central Europe	30-44 years	2029	1999.950264
42	Central Europe	30-44 years	2030	1931.228535
42	Central Europe	30-44 years	2031	1863.641115
42	Central Europe	30-44 years	2032	1797.495168
42	Central Europe	30-44 years	2033	1729.224426
42	Central Europe	30-44 years	2034	1658.424583
42	Central Europe	30-44 years	2035	1589.635056
42	Central Europe	30-44 years	2036	1527.586457
42	Central Europe	45-59 years	1990	42861.63232
42	Central Europe	45-59 years	1991	42869.53245
42	Central Europe	45-59 years	1992	42567.84331
42	Central Europe	45-59 years	1993	41334.38011
42	Central Europe	45-59 years	1994	40430.42339
42	Central Europe	45-59 years	1995	40600.45882
42	Central Europe	45-59 years	1996	39794.64788
42	Central Europe	45-59 years	1997	39576.10814
42	Central Europe	45-59 years	1998	37586.85954
42	Central Europe	45-59 years	1999	36359.36561
42	Central Europe	45-59 years	2000	34695.14187
42	Central Europe	45-59 years	2001	34227.13347

42	Central Europe	45-59 years	2002	33969.40607
42	Central Europe	45-59 years	2003	34095.23562
42	Central Europe	45-59 years	2004	33974.76794
42	Central Europe	45-59 years	2005	34415.41804
42	Central Europe	45-59 years	2006	34229.62702
42	Central Europe	45-59 years	2007	33653.61738
42	Central Europe	45-59 years	2008	32699.17925
42	Central Europe	45-59 years	2009	31505.40906
42	Central Europe	45-59 years	2010	30015.55836
42	Central Europe	45-59 years	2011	28369.1013
42	Central Europe	45-59 years	2012	27279.3033
42	Central Europe	45-59 years	2013	25619.07439
42	Central Europe	45-59 years	2014	24617.24479
42	Central Europe	45-59 years	2015	23863.23459
42	Central Europe	45-59 years	2016	22651.76347
42	Central Europe	45-59 years	2017	21774.64605
42	Central Europe	45-59 years	2018	21114.04956
42	Central Europe	45-59 years	2019	20253.23742
42	Central Europe	45-59 years	2020	19130.01593
42	Central Europe	45-59 years	2021	18845.32202
42	Central Europe	45-59 years	2022	17464.88222
42	Central Europe	45-59 years	2023	17007.19781
42	Central Europe	45-59 years	2024	16671.1785
42	Central Europe	45-59 years	2025	16396.01095
42	Central Europe	45-59 years	2026	16138.42175
42	Central Europe	45-59 years	2027	15827.88795
42	Central Europe	45-59 years	2028	15479.98227
42	Central Europe	45-59 years	2029	15122.93383
42	Central Europe	45-59 years	2030	14761.65482
42	Central Europe	45-59 years	2031	14434.96497
42	Central Europe	45-59 years	2032	14143.38551
42	Central Europe	45-59 years	2033	13872.72123
42	Central Europe	45-59 years	2034	13599.24913
42	Central Europe	45-59 years	2035	13323.24113
42	Central Europe	45-59 years	2036	13073.26933
42	Central Europe	60-75 years	1990	111240.1684
42	Central Europe	60-75 years	1991	116598.4232
42	Central Europe	60-75 years	1992	122780.4993
42	Central Europe	60-75 years	1993	127855.3022
42	Central Europe	60-75 years	1994	131947.9651
42	Central Europe	60-75 years	1995	134843.4933
42	Central Europe	60-75 years	1996	134775.6201
42	Central Europe	60-75 years	1997	134247.1197
42	Central Europe	60-75 years	1998	126435.856
42	Central Europe	60-75 years	1999	121200.2781
42	Central Europe	60-75 years	2000	114117.8943
42	Central Europe	60-75 years	2001	109698.1705
42	Central Europe	60-75 years	2002	105762.4164
42	Central Europe	60-75 years	2003	102630.1509
42	Central Europe	60-75 years	2004	98000.55972

42	Central Europe	60-75 years	2005	95147.68482
42	Central Europe	60-75 years	2006	90932.44249
42	Central Europe	60-75 years	2007	88159.88356
42	Central Europe	60-75 years	2008	85272.13871
42	Central Europe	60-75 years	2009	83424.7484
42	Central Europe	60-75 years	2010	81329.50916
42	Central Europe	60-75 years	2011	79349.92453
42	Central Europe	60-75 years	2012	78492.23933
42	Central Europe	60-75 years	2013	76410.17742
42	Central Europe	60-75 years	2014	76055.75123
42	Central Europe	60-75 years	2015	77805.37297
42	Central Europe	60-75 years	2016	77223.21708
42	Central Europe	60-75 years	2017	78863.91593
42	Central Europe	60-75 years	2018	80363.53711
42	Central Europe	60-75 years	2019	80963.98663
42	Central Europe	60-75 years	2020	82167.36248
42	Central Europe	60-75 years	2021	82844.84215
42	Central Europe	60-75 years	2022	82937.57767
42	Central Europe	60-75 years	2023	82183.14342
42	Central Europe	60-75 years	2024	80754.5211
42	Central Europe	60-75 years	2025	78956.01929
42	Central Europe	60-75 years	2026	76947.57227
42	Central Europe	60-75 years	2027	74932.3176
42	Central Europe	60-75 years	2028	72681.37035
42	Central Europe	60-75 years	2029	70184.8418
42	Central Europe	60-75 years	2030	67627.3475
42	Central Europe	60-75 years	2031	65176.61025
42	Central Europe	60-75 years	2032	62982.76507
42	Central Europe	60-75 years	2033	61049.92401
42	Central Europe	60-75 years	2034	59356.50925
42	Central Europe	60-75 years	2035	57920.64152
42	Central Europe	60-75 years	2036	56785.61374
42	Central Europe	75+ years	1990	196318.1687
42	Central Europe	75+ years	1991	197231.8383
42	Central Europe	75+ years	1992	193817.6281
42	Central Europe	75+ years	1993	190303.1288
42	Central Europe	75+ years	1994	188823.0872
42	Central Europe	75+ years	1995	192375.21
42	Central Europe	75+ years	1996	192700.242
42	Central Europe	75+ years	1997	193928.2822
42	Central Europe	75+ years	1998	189456.406
42	Central Europe	75+ years	1999	188132.1415
42	Central Europe	75+ years	2000	184075.6231
42	Central Europe	75+ years	2001	184245.5731
42	Central Europe	75+ years	2002	186164.4586
42	Central Europe	75+ years	2003	188922.7639
42	Central Europe	75+ years	2004	188901.4789
42	Central Europe	75+ years	2005	193779.3506
42	Central Europe	75+ years	2006	195569.027
42	Central Europe	75+ years	2007	198469.7908

42	Central Europe	75+ years	2008	201736.5781
42	Central Europe	75+ years	2009	206515.4211
42	Central Europe	75+ years	2010	208271.5016
42	Central Europe	75+ years	2011	209812.1914
42	Central Europe	75+ years	2012	212605.6893
42	Central Europe	75+ years	2013	210948.8784
42	Central Europe	75+ years	2014	213212.8341
42	Central Europe	75+ years	2015	217738.5717
42	Central Europe	75+ years	2016	214568.76
42	Central Europe	75+ years	2017	218230.0148
42	Central Europe	75+ years	2018	217669.3184
42	Central Europe	75+ years	2019	214494.5406
42	Central Europe	75+ years	2020	216355.3518
42	Central Europe	75+ years	2021	210873.5012
42	Central Europe	75+ years	2022	214062.661
42	Central Europe	75+ years	2023	214793.8113
42	Central Europe	75+ years	2024	216751.1711
42	Central Europe	75+ years	2025	219935.1389
42	Central Europe	75+ years	2026	223643.9021
42	Central Europe	75+ years	2027	227251.9284
42	Central Europe	75+ years	2028	230900.8306
42	Central Europe	75+ years	2029	235768.1578
42	Central Europe	75+ years	2030	241875.9111
42	Central Europe	75+ years	2031	248452.2345
42	Central Europe	75+ years	2032	254683.4654
42	Central Europe	75+ years	2033	260594.7582
42	Central Europe	75+ years	2034	267313.2695
42	Central Europe	75+ years	2035	274767.1891
42	Central Europe	75+ years	2036	282152.4741
124	Central Latin America	15-29 years	1990	1088.23797
124	Central Latin America	15-29 years	1991	1106.766851
124	Central Latin America	15-29 years	1992	1120.824907
124	Central Latin America	15-29 years	1993	1142.274124
124	Central Latin America	15-29 years	1994	1157.39223
124	Central Latin America	15-29 years	1995	1159.702547
124	Central Latin America	15-29 years	1996	1152.127135
124	Central Latin America	15-29 years	1997	1149.706438
124	Central Latin America	15-29 years	1998	1140.140912
124	Central Latin America	15-29 years	1999	1126.955669
124	Central Latin America	15-29 years	2000	1118.750092
124	Central Latin America	15-29 years	2001	1120.512439
124	Central Latin America	15-29 years	2002	1129.959395
124	Central Latin America	15-29 years	2003	1148.564406
124	Central Latin America	15-29 years	2004	1161.430992
124	Central Latin America	15-29 years	2005	1182.084608
124	Central Latin America	15-29 years	2006	1201.049541
124	Central Latin America	15-29 years	2007	1220.784674
124	Central Latin America	15-29 years	2008	1262.67245
124	Central Latin America	15-29 years	2009	1318.379025
124	Central Latin America	15-29 years	2010	1367.259537

124	Central Latin America	15-29 years	2011	1399.170892
124	Central Latin America	15-29 years	2012	1431.163288
124	Central Latin America	15-29 years	2013	1487.704351
124	Central Latin America	15-29 years	2014	1548.84368
124	Central Latin America	15-29 years	2015	1626.462663
124	Central Latin America	15-29 years	2016	1721.398949
124	Central Latin America	15-29 years	2017	1789.307978
124	Central Latin America	15-29 years	2018	1833.556813
124	Central Latin America	15-29 years	2019	1854.236928
124	Central Latin America	15-29 years	2020	1853.445132
124	Central Latin America	15-29 years	2021	1880.704716
124	Central Latin America	15-29 years	2022	2102.759121
124	Central Latin America	15-29 years	2023	2143.474523
124	Central Latin America	15-29 years	2024	2174.849914
124	Central Latin America	15-29 years	2025	2198.588382
124	Central Latin America	15-29 years	2026	2219.980775
124	Central Latin America	15-29 years	2027	2243.46843
124	Central Latin America	15-29 years	2028	2269.703115
124	Central Latin America	15-29 years	2029	2297.967881
124	Central Latin America	15-29 years	2030	2328.999556
124	Central Latin America	15-29 years	2031	2363.932531
124	Central Latin America	15-29 years	2032	2404.073031
124	Central Latin America	15-29 years	2033	2448.71316
124	Central Latin America	15-29 years	2034	2497.389279
124	Central Latin America	15-29 years	2035	2551.045942
124	Central Latin America	15-29 years	2036	2610.774204
124	Central Latin America	30-44 years	1990	4135.323935
124	Central Latin America	30-44 years	1991	4293.05118
124	Central Latin America	30-44 years	1992	4420.670609
124	Central Latin America	30-44 years	1993	4548.874106
124	Central Latin America	30-44 years	1994	4662.599616
124	Central Latin America	30-44 years	1995	4728.420132
124	Central Latin America	30-44 years	1996	4730.64933
124	Central Latin America	30-44 years	1997	4732.985302
124	Central Latin America	30-44 years	1998	4719.168964
124	Central Latin America	30-44 years	1999	4657.402793
124	Central Latin America	30-44 years	2000	4637.856518
124	Central Latin America	30-44 years	2001	4640.15493
124	Central Latin America	30-44 years	2002	4674.4987
124	Central Latin America	30-44 years	2003	4763.030832
124	Central Latin America	30-44 years	2004	4767.01078
124	Central Latin America	30-44 years	2005	4857.557257
124	Central Latin America	30-44 years	2006	4938.003261
124	Central Latin America	30-44 years	2007	5006.073848
124	Central Latin America	30-44 years	2008	5188.616592
124	Central Latin America	30-44 years	2009	5380.45233
124	Central Latin America	30-44 years	2010	5501.028598
124	Central Latin America	30-44 years	2011	5575.632098
124	Central Latin America	30-44 years	2012	5674.393342
124	Central Latin America	30-44 years	2013	5893.896725

124	Central Latin America	30-44 years	2014	6108.368751
124	Central Latin America	30-44 years	2015	6410.780148
124	Central Latin America	30-44 years	2016	6899.060915
124	Central Latin America	30-44 years	2017	7153.431173
124	Central Latin America	30-44 years	2018	7407.500557
124	Central Latin America	30-44 years	2019	7582.984684
124	Central Latin America	30-44 years	2020	7683.927335
124	Central Latin America	30-44 years	2021	7899.572871
124	Central Latin America	30-44 years	2022	8607.80264
124	Central Latin America	30-44 years	2023	8951.147964
124	Central Latin America	30-44 years	2024	9308.358518
124	Central Latin America	30-44 years	2025	9684.085701
124	Central Latin America	30-44 years	2026	10087.97224
124	Central Latin America	30-44 years	2027	10523.01105
124	Central Latin America	30-44 years	2028	10986.96551
124	Central Latin America	30-44 years	2029	11477.55364
124	Central Latin America	30-44 years	2030	11989.94258
124	Central Latin America	30-44 years	2031	12526.88555
124	Central Latin America	30-44 years	2032	13081.06591
124	Central Latin America	30-44 years	2033	13634.7758
124	Central Latin America	30-44 years	2034	14182.76537
124	Central Latin America	30-44 years	2035	14722.5708
124	Central Latin America	30-44 years	2036	15262.14274
124	Central Latin America	45-59 years	1990	13201.15015
124	Central Latin America	45-59 years	1991	13545.82845
124	Central Latin America	45-59 years	1992	13862.10444
124	Central Latin America	45-59 years	1993	14157.39604
124	Central Latin America	45-59 years	1994	14522.79177
124	Central Latin America	45-59 years	1995	14691.63126
124	Central Latin America	45-59 years	1996	14751.25772
124	Central Latin America	45-59 years	1997	14782.26414
124	Central Latin America	45-59 years	1998	14930.01312
124	Central Latin America	45-59 years	1999	14964.50534
124	Central Latin America	45-59 years	2000	15140.18476
124	Central Latin America	45-59 years	2001	15388.31932
124	Central Latin America	45-59 years	2002	15689.33643
124	Central Latin America	45-59 years	2003	16354.93508
124	Central Latin America	45-59 years	2004	16582.73322
124	Central Latin America	45-59 years	2005	17021.95294
124	Central Latin America	45-59 years	2006	17665.54146
124	Central Latin America	45-59 years	2007	18027.43475
124	Central Latin America	45-59 years	2008	18924.14601
124	Central Latin America	45-59 years	2009	19692.24706
124	Central Latin America	45-59 years	2010	20155.80186
124	Central Latin America	45-59 years	2011	20496.46438
124	Central Latin America	45-59 years	2012	21046.31294
124	Central Latin America	45-59 years	2013	21966.93518
124	Central Latin America	45-59 years	2014	22565.55659
124	Central Latin America	45-59 years	2015	23409.29724
124	Central Latin America	45-59 years	2016	25141.22695

124	Central Latin America	45-59 years	2017	25737.21978
124	Central Latin America	45-59 years	2018	26838.68157
124	Central Latin America	45-59 years	2019	27642.55551
124	Central Latin America	45-59 years	2020	29025.87241
124	Central Latin America	45-59 years	2021	29950.13012
124	Central Latin America	45-59 years	2022	30799.40778
124	Central Latin America	45-59 years	2023	31846.06996
124	Central Latin America	45-59 years	2024	32960.63012
124	Central Latin America	45-59 years	2025	34159.72614
124	Central Latin America	45-59 years	2026	35433.28341
124	Central Latin America	45-59 years	2027	36776.92635
124	Central Latin America	45-59 years	2028	38216.49812
124	Central Latin America	45-59 years	2029	39742.23941
124	Central Latin America	45-59 years	2030	41370.07385
124	Central Latin America	45-59 years	2031	43102.63519
124	Central Latin America	45-59 years	2032	44947.72385
124	Central Latin America	45-59 years	2033	46934.71086
124	Central Latin America	45-59 years	2034	49083.78816
124	Central Latin America	45-59 years	2035	51430.31683
124	Central Latin America	45-59 years	2036	54010.37962
124	Central Latin America	60-75 years	1990	28869.15801
124	Central Latin America	60-75 years	1991	30329.9443
124	Central Latin America	60-75 years	1992	31638.4579
124	Central Latin America	60-75 years	1993	32813.05776
124	Central Latin America	60-75 years	1994	33654.79431
124	Central Latin America	60-75 years	1995	34105.74331
124	Central Latin America	60-75 years	1996	34474.77206
124	Central Latin America	60-75 years	1997	34571.21082
124	Central Latin America	60-75 years	1998	34996.83848
124	Central Latin America	60-75 years	1999	35115.23015
124	Central Latin America	60-75 years	2000	35380.12189
124	Central Latin America	60-75 years	2001	35904.52788
124	Central Latin America	60-75 years	2002	36370.93939
124	Central Latin America	60-75 years	2003	37310.8119
124	Central Latin America	60-75 years	2004	37393.72936
124	Central Latin America	60-75 years	2005	37943.32597
124	Central Latin America	60-75 years	2006	38662.7516
124	Central Latin America	60-75 years	2007	38861.00244
124	Central Latin America	60-75 years	2008	40755.71974
124	Central Latin America	60-75 years	2009	42587.30323
124	Central Latin America	60-75 years	2010	43581.57574
124	Central Latin America	60-75 years	2011	44436.32531
124	Central Latin America	60-75 years	2012	45929.21306
124	Central Latin America	60-75 years	2013	48099.95169
124	Central Latin America	60-75 years	2014	49766.59967
124	Central Latin America	60-75 years	2015	51859.4799
124	Central Latin America	60-75 years	2016	55894.32702
124	Central Latin America	60-75 years	2017	57478.05219
124	Central Latin America	60-75 years	2018	60749.22871
124	Central Latin America	60-75 years	2019	63530.29227

124	Central Latin America	60-75 years	2020	67411.48028
124	Central Latin America	60-75 years	2021	70135.88479
124	Central Latin America	60-75 years	2022	74628.55485
124	Central Latin America	60-75 years	2023	77835.14902
124	Central Latin America	60-75 years	2024	81092.22143
124	Central Latin America	60-75 years	2025	84433.13368
124	Central Latin America	60-75 years	2026	87833.74966
124	Central Latin America	60-75 years	2027	91263.97493
124	Central Latin America	60-75 years	2028	94852.74723
124	Central Latin America	60-75 years	2029	98553.31128
124	Central Latin America	60-75 years	2030	102445.8037
124	Central Latin America	60-75 years	2031	106491.1511
124	Central Latin America	60-75 years	2032	110676.121
124	Central Latin America	60-75 years	2033	115151.22
124	Central Latin America	60-75 years	2034	119866.9959
124	Central Latin America	60-75 years	2035	124945.8492
124	Central Latin America	60-75 years	2036	130372.9566
124	Central Latin America	75+ years	1990	40203.51662
124	Central Latin America	75+ years	1991	41762.58262
124	Central Latin America	75+ years	1992	43317.9811
124	Central Latin America	75+ years	1993	44901.44463
124	Central Latin America	75+ years	1994	46786.43183
124	Central Latin America	75+ years	1995	48729.90744
124	Central Latin America	75+ years	1996	50537.02158
124	Central Latin America	75+ years	1997	50518.86129
124	Central Latin America	75+ years	1998	50400.73945
124	Central Latin America	75+ years	1999	50979.62468
124	Central Latin America	75+ years	2000	51609.36542
124	Central Latin America	75+ years	2001	53162.92113
124	Central Latin America	75+ years	2002	55388.54637
124	Central Latin America	75+ years	2003	58050.63029
124	Central Latin America	75+ years	2004	59364.87351
124	Central Latin America	75+ years	2005	61740.58334
124	Central Latin America	75+ years	2006	63791.51784
124	Central Latin America	75+ years	2007	65608.62581
124	Central Latin America	75+ years	2008	69785.81059
124	Central Latin America	75+ years	2009	74270.39377
124	Central Latin America	75+ years	2010	78342.27605
124	Central Latin America	75+ years	2011	81140.40786
124	Central Latin America	75+ years	2012	84507.03398
124	Central Latin America	75+ years	2013	90271.94629
124	Central Latin America	75+ years	2014	94937.32795
124	Central Latin America	75+ years	2015	99626.48224
124	Central Latin America	75+ years	2016	106320.9078
124	Central Latin America	75+ years	2017	109478.9393
124	Central Latin America	75+ years	2018	115056.6513
124	Central Latin America	75+ years	2019	119558.3259
124	Central Latin America	75+ years	2020	120868.4868
124	Central Latin America	75+ years	2021	124421.5159
124	Central Latin America	75+ years	2022	142095.1669

124	Central Latin America	75+ years	2023	148410.5479
124	Central Latin America	75+ years	2024	155227.4111
124	Central Latin America	75+ years	2025	162448.6044
124	Central Latin America	75+ years	2026	170088.4476
124	Central Latin America	75+ years	2027	177999.7333
124	Central Latin America	75+ years	2028	186580.1142
124	Central Latin America	75+ years	2029	195867.8216
124	Central Latin America	75+ years	2030	205851.0606
124	Central Latin America	75+ years	2031	216546.6107
124	Central Latin America	75+ years	2032	227832.2734
124	Central Latin America	75+ years	2033	240233.7869
124	Central Latin America	75+ years	2034	253778.3721
124	Central Latin America	75+ years	2035	268482.4247
124	Central Latin America	75+ years	2036	284305.1185
167	Central Sub-Saharan Africa	15-29 years	1990	191.5578539
167	Central Sub-Saharan Africa	15-29 years	1991	195.6421435
167	Central Sub-Saharan Africa	15-29 years	1992	199.9802069
167	Central Sub-Saharan Africa	15-29 years	1993	204.807495
167	Central Sub-Saharan Africa	15-29 years	1994	210.3694523
167	Central Sub-Saharan Africa	15-29 years	1995	216.4234142
167	Central Sub-Saharan Africa	15-29 years	1996	222.1344176
167	Central Sub-Saharan Africa	15-29 years	1997	227.018061
167	Central Sub-Saharan Africa	15-29 years	1998	231.9247511
167	Central Sub-Saharan Africa	15-29 years	1999	236.8811577
167	Central Sub-Saharan Africa	15-29 years	2000	241.8356141
167	Central Sub-Saharan Africa	15-29 years	2001	246.5831962
167	Central Sub-Saharan Africa	15-29 years	2002	251.3709619
167	Central Sub-Saharan Africa	15-29 years	2003	256.9258783
167	Central Sub-Saharan Africa	15-29 years	2004	262.8888468
167	Central Sub-Saharan Africa	15-29 years	2005	269.4282721
167	Central Sub-Saharan Africa	15-29 years	2006	277.094817
167	Central Sub-Saharan Africa	15-29 years	2007	285.8334216
167	Central Sub-Saharan Africa	15-29 years	2008	295.4290356
167	Central Sub-Saharan Africa	15-29 years	2009	304.9384778
167	Central Sub-Saharan Africa	15-29 years	2010	314.4307095
167	Central Sub-Saharan Africa	15-29 years	2011	323.9312379
167	Central Sub-Saharan Africa	15-29 years	2012	333.198976
167	Central Sub-Saharan Africa	15-29 years	2013	342.2386402
167	Central Sub-Saharan Africa	15-29 years	2014	351.3757681
167	Central Sub-Saharan Africa	15-29 years	2015	361.4210062
167	Central Sub-Saharan Africa	15-29 years	2016	372.2064906
167	Central Sub-Saharan Africa	15-29 years	2017	384.295964
167	Central Sub-Saharan Africa	15-29 years	2018	398.1152501
167	Central Sub-Saharan Africa	15-29 years	2019	413.5867312
167	Central Sub-Saharan Africa	15-29 years	2020	430.5868589
167	Central Sub-Saharan Africa	15-29 years	2021	448.4584898
167	Central Sub-Saharan Africa	15-29 years	2022	463.3537602
167	Central Sub-Saharan Africa	15-29 years	2023	482.8789542
167	Central Sub-Saharan Africa	15-29 years	2024	503.6233312
167	Central Sub-Saharan Africa	15-29 years	2025	525.2427507

167	Central Sub-Saharan Africa	15-29 years	2026	547.2784787
167	Central Sub-Saharan Africa	15-29 years	2027	569.2335651
167	Central Sub-Saharan Africa	15-29 years	2028	591.4618254
167	Central Sub-Saharan Africa	15-29 years	2029	614.3511661
167	Central Sub-Saharan Africa	15-29 years	2030	637.6741812
167	Central Sub-Saharan Africa	15-29 years	2031	661.1057314
167	Central Sub-Saharan Africa	15-29 years	2032	684.5236431
167	Central Sub-Saharan Africa	15-29 years	2033	708.1512077
167	Central Sub-Saharan Africa	15-29 years	2034	732.4129021
167	Central Sub-Saharan Africa	15-29 years	2035	757.2900544
167	Central Sub-Saharan Africa	15-29 years	2036	782.602576
167	Central Sub-Saharan Africa	30-44 years	1990	949.9233779
167	Central Sub-Saharan Africa	30-44 years	1991	987.4047366
167	Central Sub-Saharan Africa	30-44 years	1992	1025.794361
167	Central Sub-Saharan Africa	30-44 years	1993	1066.342165
167	Central Sub-Saharan Africa	30-44 years	1994	1111.922679
167	Central Sub-Saharan Africa	30-44 years	1995	1175.156001
167	Central Sub-Saharan Africa	30-44 years	1996	1204.085275
167	Central Sub-Saharan Africa	30-44 years	1997	1228.118952
167	Central Sub-Saharan Africa	30-44 years	1998	1254.351753
167	Central Sub-Saharan Africa	30-44 years	1999	1279.874786
167	Central Sub-Saharan Africa	30-44 years	2000	1302.685551
167	Central Sub-Saharan Africa	30-44 years	2001	1322.126248
167	Central Sub-Saharan Africa	30-44 years	2002	1340.371961
167	Central Sub-Saharan Africa	30-44 years	2003	1364.980437
167	Central Sub-Saharan Africa	30-44 years	2004	1389.411173
167	Central Sub-Saharan Africa	30-44 years	2005	1415.788937
167	Central Sub-Saharan Africa	30-44 years	2006	1450.09482
167	Central Sub-Saharan Africa	30-44 years	2007	1491.113074
167	Central Sub-Saharan Africa	30-44 years	2008	1539.289426
167	Central Sub-Saharan Africa	30-44 years	2009	1586.199905
167	Central Sub-Saharan Africa	30-44 years	2010	1634.483604
167	Central Sub-Saharan Africa	30-44 years	2011	1684.971848
167	Central Sub-Saharan Africa	30-44 years	2012	1736.476307
167	Central Sub-Saharan Africa	30-44 years	2013	1788.605775
167	Central Sub-Saharan Africa	30-44 years	2014	1847.055046
167	Central Sub-Saharan Africa	30-44 years	2015	1916.679855
167	Central Sub-Saharan Africa	30-44 years	2016	1990.372372
167	Central Sub-Saharan Africa	30-44 years	2017	2072.339325
167	Central Sub-Saharan Africa	30-44 years	2018	2166.271409
167	Central Sub-Saharan Africa	30-44 years	2019	2267.493893
167	Central Sub-Saharan Africa	30-44 years	2020	2374.583636
167	Central Sub-Saharan Africa	30-44 years	2021	2484.361041
167	Central Sub-Saharan Africa	30-44 years	2022	2614.029085
167	Central Sub-Saharan Africa	30-44 years	2023	2740.885303
167	Central Sub-Saharan Africa	30-44 years	2024	2872.99293
167	Central Sub-Saharan Africa	30-44 years	2025	3009.866892
167	Central Sub-Saharan Africa	30-44 years	2026	3151.40119
167	Central Sub-Saharan Africa	30-44 years	2027	3297.513364
167	Central Sub-Saharan Africa	30-44 years	2028	3448.102623

167	Central Sub-Saharan Africa	30-44 years	2029	3603.566335
167	Central Sub-Saharan Africa	30-44 years	2030	3764.785328
167	Central Sub-Saharan Africa	30-44 years	2031	3932.570376
167	Central Sub-Saharan Africa	30-44 years	2032	4107.431524
167	Central Sub-Saharan Africa	30-44 years	2033	4288.814156
167	Central Sub-Saharan Africa	30-44 years	2034	4477.096191
167	Central Sub-Saharan Africa	30-44 years	2035	4674.818624
167	Central Sub-Saharan Africa	30-44 years	2036	4884.431863
167	Central Sub-Saharan Africa	45-59 years	1990	5128.197185
167	Central Sub-Saharan Africa	45-59 years	1991	5155.064551
167	Central Sub-Saharan Africa	45-59 years	1992	5176.568566
167	Central Sub-Saharan Africa	45-59 years	1993	5207.452223
167	Central Sub-Saharan Africa	45-59 years	1994	5262.319557
167	Central Sub-Saharan Africa	45-59 years	1995	5300.250733
167	Central Sub-Saharan Africa	45-59 years	1996	5399.822519
167	Central Sub-Saharan Africa	45-59 years	1997	5443.914107
167	Central Sub-Saharan Africa	45-59 years	1998	5510.904267
167	Central Sub-Saharan Africa	45-59 years	1999	5571.060484
167	Central Sub-Saharan Africa	45-59 years	2000	5593.736133
167	Central Sub-Saharan Africa	45-59 years	2001	5664.528468
167	Central Sub-Saharan Africa	45-59 years	2002	5723.381885
167	Central Sub-Saharan Africa	45-59 years	2003	5843.629872
167	Central Sub-Saharan Africa	45-59 years	2004	5962.763817
167	Central Sub-Saharan Africa	45-59 years	2005	6072.456401
167	Central Sub-Saharan Africa	45-59 years	2006	6300.385089
167	Central Sub-Saharan Africa	45-59 years	2007	6562.648607
167	Central Sub-Saharan Africa	45-59 years	2008	6872.771451
167	Central Sub-Saharan Africa	45-59 years	2009	7167.789998
167	Central Sub-Saharan Africa	45-59 years	2010	7542.098421
167	Central Sub-Saharan Africa	45-59 years	2011	7775.54818
167	Central Sub-Saharan Africa	45-59 years	2012	8018.88481
167	Central Sub-Saharan Africa	45-59 years	2013	8252.214396
167	Central Sub-Saharan Africa	45-59 years	2014	8518.252289
167	Central Sub-Saharan Africa	45-59 years	2015	8850.267388
167	Central Sub-Saharan Africa	45-59 years	2016	9173.168334
167	Central Sub-Saharan Africa	45-59 years	2017	9521.372606
167	Central Sub-Saharan Africa	45-59 years	2018	9938.385151
167	Central Sub-Saharan Africa	45-59 years	2019	10372.60366
167	Central Sub-Saharan Africa	45-59 years	2020	10822.08191
167	Central Sub-Saharan Africa	45-59 years	2021	11257.2737
167	Central Sub-Saharan Africa	45-59 years	2022	11843.85512
167	Central Sub-Saharan Africa	45-59 years	2023	12389.19534
167	Central Sub-Saharan Africa	45-59 years	2024	12955.92601
167	Central Sub-Saharan Africa	45-59 years	2025	13554.23399
167	Central Sub-Saharan Africa	45-59 years	2026	14194.3642
167	Central Sub-Saharan Africa	45-59 years	2027	14885.24523
167	Central Sub-Saharan Africa	45-59 years	2028	15631.0372
167	Central Sub-Saharan Africa	45-59 years	2029	16431.11177
167	Central Sub-Saharan Africa	45-59 years	2030	17286.19934
167	Central Sub-Saharan Africa	45-59 years	2031	18196.94002

167	Central Sub-Saharan Africa	45-59 years	2032	19162.73442
167	Central Sub-Saharan Africa	45-59 years	2033	20187.2946
167	Central Sub-Saharan Africa	45-59 years	2034	21276.96045
167	Central Sub-Saharan Africa	45-59 years	2035	22434.44182
167	Central Sub-Saharan Africa	45-59 years	2036	23661.90011
167	Central Sub-Saharan Africa	60-75 years	1990	11686.82319
167	Central Sub-Saharan Africa	60-75 years	1991	11928.81947
167	Central Sub-Saharan Africa	60-75 years	1992	12151.15815
167	Central Sub-Saharan Africa	60-75 years	1993	12374.13206
167	Central Sub-Saharan Africa	60-75 years	1994	12652.58073
167	Central Sub-Saharan Africa	60-75 years	1995	12936.31082
167	Central Sub-Saharan Africa	60-75 years	1996	13213.26019
167	Central Sub-Saharan Africa	60-75 years	1997	13280.47234
167	Central Sub-Saharan Africa	60-75 years	1998	13409.7149
167	Central Sub-Saharan Africa	60-75 years	1999	13498.58867
167	Central Sub-Saharan Africa	60-75 years	2000	13596.53654
167	Central Sub-Saharan Africa	60-75 years	2001	13632.60878
167	Central Sub-Saharan Africa	60-75 years	2002	13610.1454
167	Central Sub-Saharan Africa	60-75 years	2003	13698.91318
167	Central Sub-Saharan Africa	60-75 years	2004	13733.80922
167	Central Sub-Saharan Africa	60-75 years	2005	13748.44101
167	Central Sub-Saharan Africa	60-75 years	2006	13866.16068
167	Central Sub-Saharan Africa	60-75 years	2007	14030.99551
167	Central Sub-Saharan Africa	60-75 years	2008	14290.24072
167	Central Sub-Saharan Africa	60-75 years	2009	14460.82499
167	Central Sub-Saharan Africa	60-75 years	2010	14535.8786
167	Central Sub-Saharan Africa	60-75 years	2011	14908.12969
167	Central Sub-Saharan Africa	60-75 years	2012	15279.72759
167	Central Sub-Saharan Africa	60-75 years	2013	15618.55435
167	Central Sub-Saharan Africa	60-75 years	2014	16015.48846
167	Central Sub-Saharan Africa	60-75 years	2015	16534.59038
167	Central Sub-Saharan Africa	60-75 years	2016	17156.84406
167	Central Sub-Saharan Africa	60-75 years	2017	17833.11458
167	Central Sub-Saharan Africa	60-75 years	2018	18706.73155
167	Central Sub-Saharan Africa	60-75 years	2019	19656.2206
167	Central Sub-Saharan Africa	60-75 years	2020	20622.67098
167	Central Sub-Saharan Africa	60-75 years	2021	21581.56724
167	Central Sub-Saharan Africa	60-75 years	2022	23563.25154
167	Central Sub-Saharan Africa	60-75 years	2023	24882.88513
167	Central Sub-Saharan Africa	60-75 years	2024	26313.92522
167	Central Sub-Saharan Africa	60-75 years	2025	27827.77107
167	Central Sub-Saharan Africa	60-75 years	2026	29395.42497
167	Central Sub-Saharan Africa	60-75 years	2027	30990.90122
167	Central Sub-Saharan Africa	60-75 years	2028	32609.22643
167	Central Sub-Saharan Africa	60-75 years	2029	34276.23279
167	Central Sub-Saharan Africa	60-75 years	2030	36016.37643
167	Central Sub-Saharan Africa	60-75 years	2031	37851.59841
167	Central Sub-Saharan Africa	60-75 years	2032	39801.3156
167	Central Sub-Saharan Africa	60-75 years	2033	41872.77241
167	Central Sub-Saharan Africa	60-75 years	2034	44070.51637

167	Central Sub-Saharan Africa	60-75 years	2035	46410.20608
167	Central Sub-Saharan Africa	60-75 years	2036	48907.33438
167	Central Sub-Saharan Africa	75+ years	1990	5214.615564
167	Central Sub-Saharan Africa	75+ years	1991	5523.958836
167	Central Sub-Saharan Africa	75+ years	1992	5848.846913
167	Central Sub-Saharan Africa	75+ years	1993	6186.886956
167	Central Sub-Saharan Africa	75+ years	1994	6541.510526
167	Central Sub-Saharan Africa	75+ years	1995	6885.23838
167	Central Sub-Saharan Africa	75+ years	1996	7225.759446
167	Central Sub-Saharan Africa	75+ years	1997	7476.436668
167	Central Sub-Saharan Africa	75+ years	1998	7735.371649
167	Central Sub-Saharan Africa	75+ years	1999	7972.026303
167	Central Sub-Saharan Africa	75+ years	2000	8202.865943
167	Central Sub-Saharan Africa	75+ years	2001	8397.444509
167	Central Sub-Saharan Africa	75+ years	2002	8581.441161
167	Central Sub-Saharan Africa	75+ years	2003	8793.385343
167	Central Sub-Saharan Africa	75+ years	2004	8994.44901
167	Central Sub-Saharan Africa	75+ years	2005	9202.952545
167	Central Sub-Saharan Africa	75+ years	2006	9471.031445
167	Central Sub-Saharan Africa	75+ years	2007	9748.617101
167	Central Sub-Saharan Africa	75+ years	2008	10073.07775
167	Central Sub-Saharan Africa	75+ years	2009	10329.83639
167	Central Sub-Saharan Africa	75+ years	2010	10573.15974
167	Central Sub-Saharan Africa	75+ years	2011	10819.53376
167	Central Sub-Saharan Africa	75+ years	2012	11048.25508
167	Central Sub-Saharan Africa	75+ years	2013	11253.59119
167	Central Sub-Saharan Africa	75+ years	2014	11481.77399
167	Central Sub-Saharan Africa	75+ years	2015	11874.99959
167	Central Sub-Saharan Africa	75+ years	2016	12197.9372
167	Central Sub-Saharan Africa	75+ years	2017	12559.45652
167	Central Sub-Saharan Africa	75+ years	2018	13008.33055
167	Central Sub-Saharan Africa	75+ years	2019	13475.63951
167	Central Sub-Saharan Africa	75+ years	2020	13797.22327
167	Central Sub-Saharan Africa	75+ years	2021	13853.28242
167	Central Sub-Saharan Africa	75+ years	2022	15881.62872
167	Central Sub-Saharan Africa	75+ years	2023	16585.52219
167	Central Sub-Saharan Africa	75+ years	2024	17395.93876
167	Central Sub-Saharan Africa	75+ years	2025	18295.1074
167	Central Sub-Saharan Africa	75+ years	2026	19262.30052
167	Central Sub-Saharan Africa	75+ years	2027	20283.64288
167	Central Sub-Saharan Africa	75+ years	2028	21453.02894
167	Central Sub-Saharan Africa	75+ years	2029	22826.29066
167	Central Sub-Saharan Africa	75+ years	2030	24352.75441
167	Central Sub-Saharan Africa	75+ years	2031	25980.59937
167	Central Sub-Saharan Africa	75+ years	2032	27665.32291
167	Central Sub-Saharan Africa	75+ years	2033	29491.51057
167	Central Sub-Saharan Africa	75+ years	2034	31536.78372
167	Central Sub-Saharan Africa	75+ years	2035	33766.85235
167	Central Sub-Saharan Africa	75+ years	2036	36141.05023
5	East Asia	15-29 years	1990	10578.91652

5	East Asia	15-29 years	1991	10805.57326
5	East Asia	15-29 years	1992	10864.30571
5	East Asia	15-29 years	1993	10701.42194
5	East Asia	15-29 years	1994	10444.05819
5	East Asia	15-29 years	1995	10188.09869
5	East Asia	15-29 years	1996	9797.286269
5	East Asia	15-29 years	1997	9377.659198
5	East Asia	15-29 years	1998	9107.276617
5	East Asia	15-29 years	1999	8942.775742
5	East Asia	15-29 years	2000	8961.609433
5	East Asia	15-29 years	2001	8747.750317
5	East Asia	15-29 years	2002	8539.6867
5	East Asia	15-29 years	2003	8538.709731
5	East Asia	15-29 years	2004	8450.444792
5	East Asia	15-29 years	2005	8129.233997
5	East Asia	15-29 years	2006	7759.632015
5	East Asia	15-29 years	2007	7820.524194
5	East Asia	15-29 years	2008	8130.401693
5	East Asia	15-29 years	2009	8597.53044
5	East Asia	15-29 years	2010	8886.479349
5	East Asia	15-29 years	2011	8929.199151
5	East Asia	15-29 years	2012	8745.660898
5	East Asia	15-29 years	2013	8848.06072
5	East Asia	15-29 years	2014	8730.228379
5	East Asia	15-29 years	2015	8305.528172
5	East Asia	15-29 years	2016	7883.462849
5	East Asia	15-29 years	2017	7556.136616
5	East Asia	15-29 years	2018	7137.269664
5	East Asia	15-29 years	2019	6662.977821
5	East Asia	15-29 years	2020	6246.166305
5	East Asia	15-29 years	2021	5936.759212
5	East Asia	15-29 years	2022	5460.643704
5	East Asia	15-29 years	2023	5170.011469
5	East Asia	15-29 years	2024	4909.928522
5	East Asia	15-29 years	2025	4671.599521
5	East Asia	15-29 years	2026	4449.962729
5	East Asia	15-29 years	2027	4244.960882
5	East Asia	15-29 years	2028	4061.356718
5	East Asia	15-29 years	2029	3903.818706
5	East Asia	15-29 years	2030	3772.049759
5	East Asia	15-29 years	2031	3665.021763
5	East Asia	15-29 years	2032	3585.236173
5	East Asia	15-29 years	2033	3497.745954
5	East Asia	15-29 years	2034	3419.119217
5	East Asia	15-29 years	2035	3353.014002
5	East Asia	15-29 years	2036	3302.975478
5	East Asia	30-44 years	1990	36366.89904
5	East Asia	30-44 years	1991	37691.94129
5	East Asia	30-44 years	1992	39012.22526
5	East Asia	30-44 years	1993	40466.8403

5	East Asia	30-44 years	1994	40936.10898
5	East Asia	30-44 years	1995	42087.04832
5	East Asia	30-44 years	1996	42658.38035
5	East Asia	30-44 years	1997	43005.43854
5	East Asia	30-44 years	1998	43457.99863
5	East Asia	30-44 years	1999	43957.68393
5	East Asia	30-44 years	2000	44892.37186
5	East Asia	30-44 years	2001	45267.2991
5	East Asia	30-44 years	2002	46579.19752
5	East Asia	30-44 years	2003	48478.71746
5	East Asia	30-44 years	2004	50737.24593
5	East Asia	30-44 years	2005	52038.16651
5	East Asia	30-44 years	2006	53305.14517
5	East Asia	30-44 years	2007	54765.7327
5	East Asia	30-44 years	2008	54053.0231
5	East Asia	30-44 years	2009	52400.84021
5	East Asia	30-44 years	2010	51659.39437
5	East Asia	30-44 years	2011	49813.69791
5	East Asia	30-44 years	2012	46634.65993
5	East Asia	30-44 years	2013	46091.89171
5	East Asia	30-44 years	2014	46266.99536
5	East Asia	30-44 years	2015	44633.90856
5	East Asia	30-44 years	2016	43351.47737
5	East Asia	30-44 years	2017	43509.70546
5	East Asia	30-44 years	2018	43270.25735
5	East Asia	30-44 years	2019	42378.29476
5	East Asia	30-44 years	2020	41795.88207
5	East Asia	30-44 years	2021	41607.58269
5	East Asia	30-44 years	2022	42611.28908
5	East Asia	30-44 years	2023	42622.58503
5	East Asia	30-44 years	2024	42889.75317
5	East Asia	30-44 years	2025	43163.19758
5	East Asia	30-44 years	2026	43214.06809
5	East Asia	30-44 years	2027	42835.4608
5	East Asia	30-44 years	2028	42028.17967
5	East Asia	30-44 years	2029	40959.71972
5	East Asia	30-44 years	2030	39680.90296
5	East Asia	30-44 years	2031	38240.87238
5	East Asia	30-44 years	2032	36693.07534
5	East Asia	30-44 years	2033	34940.56533
5	East Asia	30-44 years	2034	32984.48557
5	East Asia	30-44 years	2035	31019.59906
5	East Asia	30-44 years	2036	29234.88702
5	East Asia	45-59 years	1990	93213.15762
5	East Asia	45-59 years	1991	94199.9267
5	East Asia	45-59 years	1992	95241.66405
5	East Asia	45-59 years	1993	95737.25269
5	East Asia	45-59 years	1994	95834.0985
5	East Asia	45-59 years	1995	96137.00025
5	East Asia	45-59 years	1996	97141.12846

5	East Asia	45-59 years	1997	98248.07054
5	East Asia	45-59 years	1998	101272.2847
5	East Asia	45-59 years	1999	105550.4095
5	East Asia	45-59 years	2000	111432.1521
5	East Asia	45-59 years	2001	116753.6969
5	East Asia	45-59 years	2002	124644.9719
5	East Asia	45-59 years	2003	131672.6321
5	East Asia	45-59 years	2004	139829.562
5	East Asia	45-59 years	2005	144947.5779
5	East Asia	45-59 years	2006	146842.5558
5	East Asia	45-59 years	2007	149182.0228
5	East Asia	45-59 years	2008	153254.4976
5	East Asia	45-59 years	2009	152633.2668
5	East Asia	45-59 years	2010	157385.8959
5	East Asia	45-59 years	2011	165937.9464
5	East Asia	45-59 years	2012	171996.4299
5	East Asia	45-59 years	2013	173990.308
5	East Asia	45-59 years	2014	170231.2694
5	East Asia	45-59 years	2015	167665.4044
5	East Asia	45-59 years	2016	165811.6297
5	East Asia	45-59 years	2017	166230.0886
5	East Asia	45-59 years	2018	168083.5701
5	East Asia	45-59 years	2019	171060.2622
5	East Asia	45-59 years	2020	174004.9255
5	East Asia	45-59 years	2021	176470.4976
5	East Asia	45-59 years	2022	190115.2039
5	East Asia	45-59 years	2023	189002.721
5	East Asia	45-59 years	2024	185866.4815
5	East Asia	45-59 years	2025	181606.7276
5	East Asia	45-59 years	2026	177094.9882
5	East Asia	45-59 years	2027	173181.358
5	East Asia	45-59 years	2028	169698.0159
5	East Asia	45-59 years	2029	166169.8552
5	East Asia	45-59 years	2030	162924.1015
5	East Asia	45-59 years	2031	160298.4312
5	East Asia	45-59 years	2032	158664.6317
5	East Asia	45-59 years	2033	158093.3778
5	East Asia	45-59 years	2034	158276.6672
5	East Asia	45-59 years	2035	159013.7726
5	East Asia	45-59 years	2036	160174.1185
5	East Asia	60-75 years	1990	208374.3311
5	East Asia	60-75 years	1991	213327.0043
5	East Asia	60-75 years	1992	217892.8715
5	East Asia	60-75 years	1993	222762.4619
5	East Asia	60-75 years	1994	227225.069
5	East Asia	60-75 years	1995	232761.8084
5	East Asia	60-75 years	1996	237080.3463
5	East Asia	60-75 years	1997	241708.9812
5	East Asia	60-75 years	1998	249927.1068
5	East Asia	60-75 years	1999	260302.7673

5	East Asia	60-75 years	2000	276519.8174
5	East Asia	60-75 years	2001	288779.4709
5	East Asia	60-75 years	2002	305155.5998
5	East Asia	60-75 years	2003	327469.3921
5	East Asia	60-75 years	2004	345045.6094
5	East Asia	60-75 years	2005	348695.5432
5	East Asia	60-75 years	2006	339824.9236
5	East Asia	60-75 years	2007	342636.2109
5	East Asia	60-75 years	2008	355091.956
5	East Asia	60-75 years	2009	372846.6275
5	East Asia	60-75 years	2010	386420.1477
5	East Asia	60-75 years	2011	404476.8091
5	East Asia	60-75 years	2012	414889.0763
5	East Asia	60-75 years	2013	430236.7901
5	East Asia	60-75 years	2014	436164.4836
5	East Asia	60-75 years	2015	442649.8303
5	East Asia	60-75 years	2016	452764.9492
5	East Asia	60-75 years	2017	464413.7092
5	East Asia	60-75 years	2018	475598.5372
5	East Asia	60-75 years	2019	488983.1605
5	East Asia	60-75 years	2020	502993.936
5	East Asia	60-75 years	2021	517683.0843
5	East Asia	60-75 years	2022	534035.4163
5	East Asia	60-75 years	2023	547558.4243
5	East Asia	60-75 years	2024	561884.3197
5	East Asia	60-75 years	2025	576232.66
5	East Asia	60-75 years	2026	589815.1882
5	East Asia	60-75 years	2027	601604.9664
5	East Asia	60-75 years	2028	610051.2874
5	East Asia	60-75 years	2029	615968.7139
5	East Asia	60-75 years	2030	621695.6016
5	East Asia	60-75 years	2031	629403.7773
5	East Asia	60-75 years	2032	640976.2818
5	East Asia	60-75 years	2033	659721.1293
5	East Asia	60-75 years	2034	684823.1571
5	East Asia	60-75 years	2035	712027.052
5	East Asia	60-75 years	2036	737191.0448
5	East Asia	75+ years	1990	218700.6973
5	East Asia	75+ years	1991	228534.7847
5	East Asia	75+ years	1992	238652.3505
5	East Asia	75+ years	1993	250557.2595
5	East Asia	75+ years	1994	260971.9847
5	East Asia	75+ years	1995	273659.3789
5	East Asia	75+ years	1996	287090.0203
5	East Asia	75+ years	1997	301757.3128
5	East Asia	75+ years	1998	321944.3251
5	East Asia	75+ years	1999	345919.2373
5	East Asia	75+ years	2000	383158.0105
5	East Asia	75+ years	2001	421328.7729
5	East Asia	75+ years	2002	459676.3919

5	East Asia	75+ years	2003	519194.4703
5	East Asia	75+ years	2004	570953.761
5	East Asia	75+ years	2005	605470.3867
5	East Asia	75+ years	2006	597477.5022
5	East Asia	75+ years	2007	625963.3161
5	East Asia	75+ years	2008	680687.9135
5	East Asia	75+ years	2009	745932.3767
5	East Asia	75+ years	2010	812996.674
5	East Asia	75+ years	2011	865459.7383
5	East Asia	75+ years	2012	884192.7469
5	East Asia	75+ years	2013	939152.1121
5	East Asia	75+ years	2014	971258.858
5	East Asia	75+ years	2015	984653.9537
5	East Asia	75+ years	2016	1018252.71
5	East Asia	75+ years	2017	1055174.662
5	East Asia	75+ years	2018	1086379.498
5	East Asia	75+ years	2019	1122530.495
5	East Asia	75+ years	2020	1163602.669
5	East Asia	75+ years	2021	1206347.323
5	East Asia	75+ years	2022	1415145.785
5	East Asia	75+ years	2023	1465432.234
5	East Asia	75+ years	2024	1523654.628
5	East Asia	75+ years	2025	1586787.171
5	East Asia	75+ years	2026	1652076.476
5	East Asia	75+ years	2027	1717601.151
5	East Asia	75+ years	2028	1789198.654
5	East Asia	75+ years	2029	1871826.71
5	East Asia	75+ years	2030	1962274.234
5	East Asia	75+ years	2031	2057040.167
5	East Asia	75+ years	2032	2152585.076
5	East Asia	75+ years	2033	2251667.947
5	East Asia	75+ years	2034	2359846.657
5	East Asia	75+ years	2035	2478196.924
5	East Asia	75+ years	2036	2607717.987
56	Eastern Europe	15-29 years	1990	1470.076378
56	Eastern Europe	15-29 years	1991	1537.609637
56	Eastern Europe	15-29 years	1992	1706.760994
56	Eastern Europe	15-29 years	1993	2026.035843
56	Eastern Europe	15-29 years	1994	2236.342011
56	Eastern Europe	15-29 years	1995	2185.609066
56	Eastern Europe	15-29 years	1996	1989.028038
56	Eastern Europe	15-29 years	1997	1817.046109
56	Eastern Europe	15-29 years	1998	1800.917202
56	Eastern Europe	15-29 years	1999	2068.842819
56	Eastern Europe	15-29 years	2000	2284.269303
56	Eastern Europe	15-29 years	2001	2217.24639
56	Eastern Europe	15-29 years	2002	2215.473843
56	Eastern Europe	15-29 years	2003	2326.056369
56	Eastern Europe	15-29 years	2004	2441.844244
56	Eastern Europe	15-29 years	2005	2583.031163

56	Eastern Europe	15-29 years	2006	2387.394806
56	Eastern Europe	15-29 years	2007	2178.101402
56	Eastern Europe	15-29 years	2008	2119.359719
56	Eastern Europe	15-29 years	2009	1905.915529
56	Eastern Europe	15-29 years	2010	1819.738978
56	Eastern Europe	15-29 years	2011	1690.11815
56	Eastern Europe	15-29 years	2012	1615.885589
56	Eastern Europe	15-29 years	2013	1569.655639
56	Eastern Europe	15-29 years	2014	1515.939572
56	Eastern Europe	15-29 years	2015	1281.148671
56	Eastern Europe	15-29 years	2016	1140.005996
56	Eastern Europe	15-29 years	2017	986.5075199
56	Eastern Europe	15-29 years	2018	899.1299409
56	Eastern Europe	15-29 years	2019	818.2258175
56	Eastern Europe	15-29 years	2020	746.3574955
56	Eastern Europe	15-29 years	2021	721.7027873
56	Eastern Europe	15-29 years	2022	700.2494326
56	Eastern Europe	15-29 years	2023	675.7836944
56	Eastern Europe	15-29 years	2024	661.4030707
56	Eastern Europe	15-29 years	2025	657.9272303
56	Eastern Europe	15-29 years	2026	666.1058143
56	Eastern Europe	15-29 years	2027	686.9069823
56	Eastern Europe	15-29 years	2028	725.1091348
56	Eastern Europe	15-29 years	2029	786.4775267
56	Eastern Europe	15-29 years	2030	875.9483342
56	Eastern Europe	15-29 years	2031	1001.777687
56	Eastern Europe	15-29 years	2032	1176.869576
56	Eastern Europe	15-29 years	2033	1429.479567
56	Eastern Europe	15-29 years	2034	1808.036025
56	Eastern Europe	15-29 years	2035	2387.922139
56	Eastern Europe	15-29 years	2036	3304.813791
56	Eastern Europe	30-44 years	1990	19153.60288
56	Eastern Europe	30-44 years	1991	21654.51385
56	Eastern Europe	30-44 years	1992	25844.8732
56	Eastern Europe	30-44 years	1993	33477.09851
56	Eastern Europe	30-44 years	1994	37918.77333
56	Eastern Europe	30-44 years	1995	35147.46767
56	Eastern Europe	30-44 years	1996	30490.96914
56	Eastern Europe	30-44 years	1997	26574.73845
56	Eastern Europe	30-44 years	1998	25446.62579
56	Eastern Europe	30-44 years	1999	27806.18947
56	Eastern Europe	30-44 years	2000	29115.84198
56	Eastern Europe	30-44 years	2001	29054.61439
56	Eastern Europe	30-44 years	2002	30027.71309
56	Eastern Europe	30-44 years	2003	30593.85836
56	Eastern Europe	30-44 years	2004	30118.76893
56	Eastern Europe	30-44 years	2005	30106.50102
56	Eastern Europe	30-44 years	2006	25267.96989
56	Eastern Europe	30-44 years	2007	22719.68506
56	Eastern Europe	30-44 years	2008	21706.60028

56	Eastern Europe	30-44 years	2009	18798.94849
56	Eastern Europe	30-44 years	2010	18452.5179
56	Eastern Europe	30-44 years	2011	17192.63371
56	Eastern Europe	30-44 years	2012	16571.35494
56	Eastern Europe	30-44 years	2013	16347.47755
56	Eastern Europe	30-44 years	2014	16325.85282
56	Eastern Europe	30-44 years	2015	15085.16159
56	Eastern Europe	30-44 years	2016	14172.08637
56	Eastern Europe	30-44 years	2017	13081.75382
56	Eastern Europe	30-44 years	2018	13249.58034
56	Eastern Europe	30-44 years	2019	12990.05083
56	Eastern Europe	30-44 years	2020	12594.88422
56	Eastern Europe	30-44 years	2021	12631.31653
56	Eastern Europe	30-44 years	2022	12846.2424
56	Eastern Europe	30-44 years	2023	12583.14208
56	Eastern Europe	30-44 years	2024	12402.97294
56	Eastern Europe	30-44 years	2025	12304.25043
56	Eastern Europe	30-44 years	2026	12299.40416
56	Eastern Europe	30-44 years	2027	12398.01945
56	Eastern Europe	30-44 years	2028	12611.72372
56	Eastern Europe	30-44 years	2029	12989.93667
56	Eastern Europe	30-44 years	2030	13620.93485
56	Eastern Europe	30-44 years	2031	14636.09095
56	Eastern Europe	30-44 years	2032	16222.38761
56	Eastern Europe	30-44 years	2033	18557.54434
56	Eastern Europe	30-44 years	2034	21933.60054
56	Eastern Europe	30-44 years	2035	27065.78916
56	Eastern Europe	30-44 years	2036	35320.92615
56	Eastern Europe	45-59 years	1990	86665.51106
56	Eastern Europe	45-59 years	1991	87295.28829
56	Eastern Europe	45-59 years	1992	97911.23455
56	Eastern Europe	45-59 years	1993	124018.1651
56	Eastern Europe	45-59 years	1994	143659.2213
56	Eastern Europe	45-59 years	1995	139260.9858
56	Eastern Europe	45-59 years	1996	125296.1411
56	Eastern Europe	45-59 years	1997	110874.5151
56	Eastern Europe	45-59 years	1998	103562.088
56	Eastern Europe	45-59 years	1999	110799.5513
56	Eastern Europe	45-59 years	2000	116201.0118
56	Eastern Europe	45-59 years	2001	119067.7815
56	Eastern Europe	45-59 years	2002	125962.2232
56	Eastern Europe	45-59 years	2003	135811.141
56	Eastern Europe	45-59 years	2004	142993.2123
56	Eastern Europe	45-59 years	2005	157280.0651
56	Eastern Europe	45-59 years	2006	145107.4144
56	Eastern Europe	45-59 years	2007	139665.7932
56	Eastern Europe	45-59 years	2008	141313.7942
56	Eastern Europe	45-59 years	2009	127755.4306
56	Eastern Europe	45-59 years	2010	125640.1082
56	Eastern Europe	45-59 years	2011	115966.0645

56	Eastern Europe	45-59 years	2012	110269.1207
56	Eastern Europe	45-59 years	2013	104720.4026
56	Eastern Europe	45-59 years	2014	101569.9558
56	Eastern Europe	45-59 years	2015	94540.75009
56	Eastern Europe	45-59 years	2016	90269.57105
56	Eastern Europe	45-59 years	2017	82984.26129
56	Eastern Europe	45-59 years	2018	81464.40848
56	Eastern Europe	45-59 years	2019	77757.29849
56	Eastern Europe	45-59 years	2020	72797.91815
56	Eastern Europe	45-59 years	2021	69635.51471
56	Eastern Europe	45-59 years	2022	64533.27561
56	Eastern Europe	45-59 years	2023	63393.03018
56	Eastern Europe	45-59 years	2024	63046.42344
56	Eastern Europe	45-59 years	2025	63471.92409
56	Eastern Europe	45-59 years	2026	64772.80545
56	Eastern Europe	45-59 years	2027	67229.31665
56	Eastern Europe	45-59 years	2028	71350.94378
56	Eastern Europe	45-59 years	2029	77572.81333
56	Eastern Europe	45-59 years	2030	86258.86635
56	Eastern Europe	45-59 years	2031	98097.25853
56	Eastern Europe	45-59 years	2032	114414.9445
56	Eastern Europe	45-59 years	2033	137701.3768
56	Eastern Europe	45-59 years	2034	171875.3673
56	Eastern Europe	45-59 years	2035	223070.8917
56	Eastern Europe	45-59 years	2036	302438.5195
56	Eastern Europe	60-75 years	1990	231667.681
56	Eastern Europe	60-75 years	1991	245302.121
56	Eastern Europe	60-75 years	1992	273909.0637
56	Eastern Europe	60-75 years	1993	334641.4764
56	Eastern Europe	60-75 years	1994	370597.165
56	Eastern Europe	60-75 years	1995	373810.0659
56	Eastern Europe	60-75 years	1996	367725.171
56	Eastern Europe	60-75 years	1997	365827.9243
56	Eastern Europe	60-75 years	1998	368268.9142
56	Eastern Europe	60-75 years	1999	402536.4249
56	Eastern Europe	60-75 years	2000	422648.5867
56	Eastern Europe	60-75 years	2001	430691.6467
56	Eastern Europe	60-75 years	2002	439484.069
56	Eastern Europe	60-75 years	2003	428952.0256
56	Eastern Europe	60-75 years	2004	402308.3599
56	Eastern Europe	60-75 years	2005	398289.163
56	Eastern Europe	60-75 years	2006	360418.8315
56	Eastern Europe	60-75 years	2007	348719.0183
56	Eastern Europe	60-75 years	2008	349356.8814
56	Eastern Europe	60-75 years	2009	335570.0838
56	Eastern Europe	60-75 years	2010	340036.4942
56	Eastern Europe	60-75 years	2011	318301.3181
56	Eastern Europe	60-75 years	2012	301185.7417
56	Eastern Europe	60-75 years	2013	283144.578
56	Eastern Europe	60-75 years	2014	269924.1773

56	Eastern Europe	60-75 years	2015	256569.7205
56	Eastern Europe	60-75 years	2016	250666.7836
56	Eastern Europe	60-75 years	2017	241854.1915
56	Eastern Europe	60-75 years	2018	252143.4682
56	Eastern Europe	60-75 years	2019	261738.6027
56	Eastern Europe	60-75 years	2020	268544.2311
56	Eastern Europe	60-75 years	2021	278261.9069
56	Eastern Europe	60-75 years	2022	285010.754
56	Eastern Europe	60-75 years	2023	289999.3854
56	Eastern Europe	60-75 years	2024	292740.4372
56	Eastern Europe	60-75 years	2025	295130.4415
56	Eastern Europe	60-75 years	2026	299492.7118
56	Eastern Europe	60-75 years	2027	308124.3229
56	Eastern Europe	60-75 years	2028	322144.6822
56	Eastern Europe	60-75 years	2029	341978.0682
56	Eastern Europe	60-75 years	2030	369509.6363
56	Eastern Europe	60-75 years	2031	408309.7903
56	Eastern Europe	60-75 years	2032	463649.6543
56	Eastern Europe	60-75 years	2033	541562.9976
56	Eastern Europe	60-75 years	2034	652752.7632
56	Eastern Europe	60-75 years	2035	819046.799
56	Eastern Europe	60-75 years	2036	1081990.331
56	Eastern Europe	75+ years	1990	434516.2795
56	Eastern Europe	75+ years	1991	440091.4619
56	Eastern Europe	75+ years	1992	451079.7689
56	Eastern Europe	75+ years	1993	493383.682
56	Eastern Europe	75+ years	1994	510256.0909
56	Eastern Europe	75+ years	1995	494659.7891
56	Eastern Europe	75+ years	1996	475773.8613
56	Eastern Europe	75+ years	1997	464131.0896
56	Eastern Europe	75+ years	1998	457705.8524
56	Eastern Europe	75+ years	1999	482001.5147
56	Eastern Europe	75+ years	2000	497406.1097
56	Eastern Europe	75+ years	2001	507894.2193
56	Eastern Europe	75+ years	2002	531017.6134
56	Eastern Europe	75+ years	2003	548609.5471
56	Eastern Europe	75+ years	2004	550696.3543
56	Eastern Europe	75+ years	2005	577538.4397
56	Eastern Europe	75+ years	2006	573128.4084
56	Eastern Europe	75+ years	2007	579135.4477
56	Eastern Europe	75+ years	2008	590669.7199
56	Eastern Europe	75+ years	2009	578266.8093
56	Eastern Europe	75+ years	2010	589179.5967
56	Eastern Europe	75+ years	2011	580277.0443
56	Eastern Europe	75+ years	2012	589491.4634
56	Eastern Europe	75+ years	2013	595022.1131
56	Eastern Europe	75+ years	2014	593855.7036
56	Eastern Europe	75+ years	2015	592149.8761
56	Eastern Europe	75+ years	2016	589296.5019
56	Eastern Europe	75+ years	2017	572726.5428

56	Eastern Europe	75+ years	2018	563977.0933
56	Eastern Europe	75+ years	2019	547100.2376
56	Eastern Europe	75+ years	2020	521130.2132
56	Eastern Europe	75+ years	2021	516412.4752
56	Eastern Europe	75+ years	2022	512034.9929
56	Eastern Europe	75+ years	2023	518813.2891
56	Eastern Europe	75+ years	2024	533780.9461
56	Eastern Europe	75+ years	2025	556661.535
56	Eastern Europe	75+ years	2026	587449.1727
56	Eastern Europe	75+ years	2027	626389.9722
56	Eastern Europe	75+ years	2028	678530.6464
56	Eastern Europe	75+ years	2029	750839.0802
56	Eastern Europe	75+ years	2030	848985.2714
56	Eastern Europe	75+ years	2031	982234.7777
56	Eastern Europe	75+ years	2032	1164782.872
56	Eastern Europe	75+ years	2033	1423481.197
56	Eastern Europe	75+ years	2034	1804517.938
56	Eastern Europe	75+ years	2035	2383415.584
56	Eastern Europe	75+ years	2036	3297869.26
174	Eastern Sub-Saharan Africa	15-29 years	1990	1154.524125
174	Eastern Sub-Saharan Africa	15-29 years	1991	1194.508908
174	Eastern Sub-Saharan Africa	15-29 years	1992	1237.232765
174	Eastern Sub-Saharan Africa	15-29 years	1993	1279.996109
174	Eastern Sub-Saharan Africa	15-29 years	1994	1315.977528
174	Eastern Sub-Saharan Africa	15-29 years	1995	1357.931932
174	Eastern Sub-Saharan Africa	15-29 years	1996	1410.89414
174	Eastern Sub-Saharan Africa	15-29 years	1997	1464.366643
174	Eastern Sub-Saharan Africa	15-29 years	1998	1514.103534
174	Eastern Sub-Saharan Africa	15-29 years	1999	1556.857851
174	Eastern Sub-Saharan Africa	15-29 years	2000	1595.770421
174	Eastern Sub-Saharan Africa	15-29 years	2001	1631.122713
174	Eastern Sub-Saharan Africa	15-29 years	2002	1667.657743
174	Eastern Sub-Saharan Africa	15-29 years	2003	1706.633264
174	Eastern Sub-Saharan Africa	15-29 years	2004	1747.43178
174	Eastern Sub-Saharan Africa	15-29 years	2005	1786.359468
174	Eastern Sub-Saharan Africa	15-29 years	2006	1823.955235
174	Eastern Sub-Saharan Africa	15-29 years	2007	1861.808483
174	Eastern Sub-Saharan Africa	15-29 years	2008	1901.190467
174	Eastern Sub-Saharan Africa	15-29 years	2009	1943.129897
174	Eastern Sub-Saharan Africa	15-29 years	2010	1987.523805
174	Eastern Sub-Saharan Africa	15-29 years	2011	2031.560267
174	Eastern Sub-Saharan Africa	15-29 years	2012	2073.604665
174	Eastern Sub-Saharan Africa	15-29 years	2013	2119.548752
174	Eastern Sub-Saharan Africa	15-29 years	2014	2171.056366
174	Eastern Sub-Saharan Africa	15-29 years	2015	2228.020549
174	Eastern Sub-Saharan Africa	15-29 years	2016	2283.393343
174	Eastern Sub-Saharan Africa	15-29 years	2017	2334.278699
174	Eastern Sub-Saharan Africa	15-29 years	2018	2390.757828
174	Eastern Sub-Saharan Africa	15-29 years	2019	2456.402254
174	Eastern Sub-Saharan Africa	15-29 years	2020	2527.73964

174	Eastern Sub-Saharan Africa	15-29 years	2021	2601.525501
174	Eastern Sub-Saharan Africa	15-29 years	2022	2775.81919
174	Eastern Sub-Saharan Africa	15-29 years	2023	2859.713011
174	Eastern Sub-Saharan Africa	15-29 years	2024	2941.469652
174	Eastern Sub-Saharan Africa	15-29 years	2025	3019.755289
174	Eastern Sub-Saharan Africa	15-29 years	2026	3094.375865
174	Eastern Sub-Saharan Africa	15-29 years	2027	3165.739427
174	Eastern Sub-Saharan Africa	15-29 years	2028	3234.840047
174	Eastern Sub-Saharan Africa	15-29 years	2029	3302.666105
174	Eastern Sub-Saharan Africa	15-29 years	2030	3369.796671
174	Eastern Sub-Saharan Africa	15-29 years	2031	3436.10212
174	Eastern Sub-Saharan Africa	15-29 years	2032	3503.963664
174	Eastern Sub-Saharan Africa	15-29 years	2033	3571.449452
174	Eastern Sub-Saharan Africa	15-29 years	2034	3636.689594
174	Eastern Sub-Saharan Africa	15-29 years	2035	3702.002613
174	Eastern Sub-Saharan Africa	15-29 years	2036	3769.026734
174	Eastern Sub-Saharan Africa	30-44 years	1990	2963.627744
174	Eastern Sub-Saharan Africa	30-44 years	1991	3088.264419
174	Eastern Sub-Saharan Africa	30-44 years	1992	3214.643235
174	Eastern Sub-Saharan Africa	30-44 years	1993	3339.08502
174	Eastern Sub-Saharan Africa	30-44 years	1994	3454.677485
174	Eastern Sub-Saharan Africa	30-44 years	1995	3597.267541
174	Eastern Sub-Saharan Africa	30-44 years	1996	3685.450305
174	Eastern Sub-Saharan Africa	30-44 years	1997	3783.243509
174	Eastern Sub-Saharan Africa	30-44 years	1998	3875.213598
174	Eastern Sub-Saharan Africa	30-44 years	1999	3944.972542
174	Eastern Sub-Saharan Africa	30-44 years	2000	4006.872974
174	Eastern Sub-Saharan Africa	30-44 years	2001	4059.125488
174	Eastern Sub-Saharan Africa	30-44 years	2002	4126.177306
174	Eastern Sub-Saharan Africa	30-44 years	2003	4207.079949
174	Eastern Sub-Saharan Africa	30-44 years	2004	4300.607233
174	Eastern Sub-Saharan Africa	30-44 years	2005	4394.108483
174	Eastern Sub-Saharan Africa	30-44 years	2006	4492.914164
174	Eastern Sub-Saharan Africa	30-44 years	2007	4600.380745
174	Eastern Sub-Saharan Africa	30-44 years	2008	4718.690112
174	Eastern Sub-Saharan Africa	30-44 years	2009	4853.558527
174	Eastern Sub-Saharan Africa	30-44 years	2010	5006.727975
174	Eastern Sub-Saharan Africa	30-44 years	2011	5173.513225
174	Eastern Sub-Saharan Africa	30-44 years	2012	5341.312621
174	Eastern Sub-Saharan Africa	30-44 years	2013	5519.681315
174	Eastern Sub-Saharan Africa	30-44 years	2014	5720.606676
174	Eastern Sub-Saharan Africa	30-44 years	2015	5952.787012
174	Eastern Sub-Saharan Africa	30-44 years	2016	6189.228782
174	Eastern Sub-Saharan Africa	30-44 years	2017	6404.624947
174	Eastern Sub-Saharan Africa	30-44 years	2018	6628.850176
174	Eastern Sub-Saharan Africa	30-44 years	2019	6865.394636
174	Eastern Sub-Saharan Africa	30-44 years	2020	7113.341491
174	Eastern Sub-Saharan Africa	30-44 years	2021	7374.853749
174	Eastern Sub-Saharan Africa	30-44 years	2022	7953.949956
174	Eastern Sub-Saharan Africa	30-44 years	2023	8271.111823

174	Eastern Sub-Saharan Africa	30-44 years	2024	8585.201238
174	Eastern Sub-Saharan Africa	30-44 years	2025	8901.659548
174	Eastern Sub-Saharan Africa	30-44 years	2026	9228.581245
174	Eastern Sub-Saharan Africa	30-44 years	2027	9573.635441
174	Eastern Sub-Saharan Africa	30-44 years	2028	9939.268068
174	Eastern Sub-Saharan Africa	30-44 years	2029	10322.79356
174	Eastern Sub-Saharan Africa	30-44 years	2030	10723.77876
174	Eastern Sub-Saharan Africa	30-44 years	2031	11141.66856
174	Eastern Sub-Saharan Africa	30-44 years	2032	11575.60977
174	Eastern Sub-Saharan Africa	30-44 years	2033	12036.33822
174	Eastern Sub-Saharan Africa	30-44 years	2034	12527.54827
174	Eastern Sub-Saharan Africa	30-44 years	2035	13039.2122
174	Eastern Sub-Saharan Africa	30-44 years	2036	13560.64618
174	Eastern Sub-Saharan Africa	45-59 years	1990	9285.768192
174	Eastern Sub-Saharan Africa	45-59 years	1991	9562.105098
174	Eastern Sub-Saharan Africa	45-59 years	1992	9831.755237
174	Eastern Sub-Saharan Africa	45-59 years	1993	10124.04558
174	Eastern Sub-Saharan Africa	45-59 years	1994	10348.55338
174	Eastern Sub-Saharan Africa	45-59 years	1995	10481.44737
174	Eastern Sub-Saharan Africa	45-59 years	1996	10751.34523
174	Eastern Sub-Saharan Africa	45-59 years	1997	11027.93925
174	Eastern Sub-Saharan Africa	45-59 years	1998	11278.26907
174	Eastern Sub-Saharan Africa	45-59 years	1999	11425.62388
174	Eastern Sub-Saharan Africa	45-59 years	2000	11522.63679
174	Eastern Sub-Saharan Africa	45-59 years	2001	11635.57978
174	Eastern Sub-Saharan Africa	45-59 years	2002	11787.19844
174	Eastern Sub-Saharan Africa	45-59 years	2003	12002.63779
174	Eastern Sub-Saharan Africa	45-59 years	2004	12227.09773
174	Eastern Sub-Saharan Africa	45-59 years	2005	12376.88562
174	Eastern Sub-Saharan Africa	45-59 years	2006	12631.71922
174	Eastern Sub-Saharan Africa	45-59 years	2007	12890.68653
174	Eastern Sub-Saharan Africa	45-59 years	2008	13163.63794
174	Eastern Sub-Saharan Africa	45-59 years	2009	13507.39674
174	Eastern Sub-Saharan Africa	45-59 years	2010	13988.9014
174	Eastern Sub-Saharan Africa	45-59 years	2011	14251.40672
174	Eastern Sub-Saharan Africa	45-59 years	2012	14543.33797
174	Eastern Sub-Saharan Africa	45-59 years	2013	14888.61921
174	Eastern Sub-Saharan Africa	45-59 years	2014	15311.28898
174	Eastern Sub-Saharan Africa	45-59 years	2015	15835.17982
174	Eastern Sub-Saharan Africa	45-59 years	2016	16359.11369
174	Eastern Sub-Saharan Africa	45-59 years	2017	16859.38783
174	Eastern Sub-Saharan Africa	45-59 years	2018	17419.07598
174	Eastern Sub-Saharan Africa	45-59 years	2019	18022.90484
174	Eastern Sub-Saharan Africa	45-59 years	2020	18684.30988
174	Eastern Sub-Saharan Africa	45-59 years	2021	19389.52019
174	Eastern Sub-Saharan Africa	45-59 years	2022	20994.37626
174	Eastern Sub-Saharan Africa	45-59 years	2023	21989.88495
174	Eastern Sub-Saharan Africa	45-59 years	2024	23066.2926
174	Eastern Sub-Saharan Africa	45-59 years	2025	24215.54747
174	Eastern Sub-Saharan Africa	45-59 years	2026	25429.59962

174	Eastern Sub-Saharan Africa	45-59 years	2027	26696.3716
174	Eastern Sub-Saharan Africa	45-59 years	2028	28031.19731
174	Eastern Sub-Saharan Africa	45-59 years	2029	29449.75594
174	Eastern Sub-Saharan Africa	45-59 years	2030	30940.63609
174	Eastern Sub-Saharan Africa	45-59 years	2031	32492.17243
174	Eastern Sub-Saharan Africa	45-59 years	2032	34087.79279
174	Eastern Sub-Saharan Africa	45-59 years	2033	35708.20656
174	Eastern Sub-Saharan Africa	45-59 years	2034	37360.23479
174	Eastern Sub-Saharan Africa	45-59 years	2035	39062.14662
174	Eastern Sub-Saharan Africa	45-59 years	2036	40831.98227
174	Eastern Sub-Saharan Africa	60-75 years	1990	19508.59653
174	Eastern Sub-Saharan Africa	60-75 years	1991	20125.04001
174	Eastern Sub-Saharan Africa	60-75 years	1992	20734.86925
174	Eastern Sub-Saharan Africa	60-75 years	1993	21366.02353
174	Eastern Sub-Saharan Africa	60-75 years	1994	21817.13008
174	Eastern Sub-Saharan Africa	60-75 years	1995	22320.10785
174	Eastern Sub-Saharan Africa	60-75 years	1996	22849.74545
174	Eastern Sub-Saharan Africa	60-75 years	1997	23434.11479
174	Eastern Sub-Saharan Africa	60-75 years	1998	23984.43364
174	Eastern Sub-Saharan Africa	60-75 years	1999	24391.87627
174	Eastern Sub-Saharan Africa	60-75 years	2000	24773.02199
174	Eastern Sub-Saharan Africa	60-75 years	2001	25080.88974
174	Eastern Sub-Saharan Africa	60-75 years	2002	25424.16466
174	Eastern Sub-Saharan Africa	60-75 years	2003	25830.67733
174	Eastern Sub-Saharan Africa	60-75 years	2004	26297.49764
174	Eastern Sub-Saharan Africa	60-75 years	2005	26719.05635
174	Eastern Sub-Saharan Africa	60-75 years	2006	27127.52706
174	Eastern Sub-Saharan Africa	60-75 years	2007	27544.1395
174	Eastern Sub-Saharan Africa	60-75 years	2008	28011.01278
174	Eastern Sub-Saharan Africa	60-75 years	2009	28526.13414
174	Eastern Sub-Saharan Africa	60-75 years	2010	28891.67894
174	Eastern Sub-Saharan Africa	60-75 years	2011	29659.51701
174	Eastern Sub-Saharan Africa	60-75 years	2012	30356.42529
174	Eastern Sub-Saharan Africa	60-75 years	2013	31089.67295
174	Eastern Sub-Saharan Africa	60-75 years	2014	31898.42234
174	Eastern Sub-Saharan Africa	60-75 years	2015	32862.88379
174	Eastern Sub-Saharan Africa	60-75 years	2016	33968.37984
174	Eastern Sub-Saharan Africa	60-75 years	2017	34948.90222
174	Eastern Sub-Saharan Africa	60-75 years	2018	36122.52852
174	Eastern Sub-Saharan Africa	60-75 years	2019	37308.74058
174	Eastern Sub-Saharan Africa	60-75 years	2020	38366.66046
174	Eastern Sub-Saharan Africa	60-75 years	2021	39384.3739
174	Eastern Sub-Saharan Africa	60-75 years	2022	41115.62291
174	Eastern Sub-Saharan Africa	60-75 years	2023	42591.34934
174	Eastern Sub-Saharan Africa	60-75 years	2024	44162.2585
174	Eastern Sub-Saharan Africa	60-75 years	2025	45835.23482
174	Eastern Sub-Saharan Africa	60-75 years	2026	47610.75422
174	Eastern Sub-Saharan Africa	60-75 years	2027	49488.99794
174	Eastern Sub-Saharan Africa	60-75 years	2028	51475.44827
174	Eastern Sub-Saharan Africa	60-75 years	2029	53590.58094

174	Eastern Sub-Saharan Africa	60-75 years	2030	55864.55648
174	Eastern Sub-Saharan Africa	60-75 years	2031	58324.62783
174	Eastern Sub-Saharan Africa	60-75 years	2032	60990.67994
174	Eastern Sub-Saharan Africa	60-75 years	2033	63912.4677
174	Eastern Sub-Saharan Africa	60-75 years	2034	67115.78021
174	Eastern Sub-Saharan Africa	60-75 years	2035	70589.1181
174	Eastern Sub-Saharan Africa	60-75 years	2036	74319.78336
174	Eastern Sub-Saharan Africa	75+ years	1990	10933.65661
174	Eastern Sub-Saharan Africa	75+ years	1991	11539.85388
174	Eastern Sub-Saharan Africa	75+ years	1992	12186.88523
174	Eastern Sub-Saharan Africa	75+ years	1993	12857.52579
174	Eastern Sub-Saharan Africa	75+ years	1994	13520.36682
174	Eastern Sub-Saharan Africa	75+ years	1995	14117.00437
174	Eastern Sub-Saharan Africa	75+ years	1996	14661.46241
174	Eastern Sub-Saharan Africa	75+ years	1997	15189.49327
174	Eastern Sub-Saharan Africa	75+ years	1998	15691.63913
174	Eastern Sub-Saharan Africa	75+ years	1999	16114.69647
174	Eastern Sub-Saharan Africa	75+ years	2000	16516.84844
174	Eastern Sub-Saharan Africa	75+ years	2001	16832.74167
174	Eastern Sub-Saharan Africa	75+ years	2002	17251.05866
174	Eastern Sub-Saharan Africa	75+ years	2003	17673.77198
174	Eastern Sub-Saharan Africa	75+ years	2004	18178.31858
174	Eastern Sub-Saharan Africa	75+ years	2005	18694.97555
174	Eastern Sub-Saharan Africa	75+ years	2006	19288.31845
174	Eastern Sub-Saharan Africa	75+ years	2007	19862.68533
174	Eastern Sub-Saharan Africa	75+ years	2008	20588.72203
174	Eastern Sub-Saharan Africa	75+ years	2009	21332.20768
174	Eastern Sub-Saharan Africa	75+ years	2010	22165.57902
174	Eastern Sub-Saharan Africa	75+ years	2011	23018.93836
174	Eastern Sub-Saharan Africa	75+ years	2012	23815.38888
174	Eastern Sub-Saharan Africa	75+ years	2013	24616.01263
174	Eastern Sub-Saharan Africa	75+ years	2014	25414.678
174	Eastern Sub-Saharan Africa	75+ years	2015	26508.00124
174	Eastern Sub-Saharan Africa	75+ years	2016	27545.2753
174	Eastern Sub-Saharan Africa	75+ years	2017	28568.32096
174	Eastern Sub-Saharan Africa	75+ years	2018	29644.17914
174	Eastern Sub-Saharan Africa	75+ years	2019	30753.63437
174	Eastern Sub-Saharan Africa	75+ years	2020	31634.92239
174	Eastern Sub-Saharan Africa	75+ years	2021	31742.01538
174	Eastern Sub-Saharan Africa	75+ years	2022	36421.68687
174	Eastern Sub-Saharan Africa	75+ years	2023	37961.63621
174	Eastern Sub-Saharan Africa	75+ years	2024	39673.04865
174	Eastern Sub-Saharan Africa	75+ years	2025	41489.03499
174	Eastern Sub-Saharan Africa	75+ years	2026	43340.60061
174	Eastern Sub-Saharan Africa	75+ years	2027	45177.65807
174	Eastern Sub-Saharan Africa	75+ years	2028	47117.09644
174	Eastern Sub-Saharan Africa	75+ years	2029	49258.25129
174	Eastern Sub-Saharan Africa	75+ years	2030	51550.5813
174	Eastern Sub-Saharan Africa	75+ years	2031	53933.40525
174	Eastern Sub-Saharan Africa	75+ years	2032	56361.81317

174	Eastern Sub-Saharan Africa	75+ years	2033	58929.86993
174	Eastern Sub-Saharan Africa	75+ years	2034	61726.6162
174	Eastern Sub-Saharan Africa	75+ years	2035	64725.74264
174	Eastern Sub-Saharan Africa	75+ years	2036	67891.91921
1	Global	15-29 years	1990	49924.12446
1	Global	15-29 years	1991	50551.09023
1	Global	15-29 years	1992	51424.75216
1	Global	15-29 years	1993	52445.97417
1	Global	15-29 years	1994	53351.75626
1	Global	15-29 years	1995	53606.91039
1	Global	15-29 years	1996	53815.93594
1	Global	15-29 years	1997	54094.48808
1	Global	15-29 years	1998	54900.80988
1	Global	15-29 years	1999	56480.76815
1	Global	15-29 years	2000	57233.54553
1	Global	15-29 years	2001	56992.75499
1	Global	15-29 years	2002	57008.28553
1	Global	15-29 years	2003	57088.84837
1	Global	15-29 years	2004	57974.87624
1	Global	15-29 years	2005	58495.99521
1	Global	15-29 years	2006	58571.91304
1	Global	15-29 years	2007	58654.22696
1	Global	15-29 years	2008	59703.55404
1	Global	15-29 years	2009	60373.95191
1	Global	15-29 years	2010	61519.86534
1	Global	15-29 years	2011	61480.98618
1	Global	15-29 years	2012	61710.87594
1	Global	15-29 years	2013	62060.51463
1	Global	15-29 years	2014	61015.8198
1	Global	15-29 years	2015	59257.48633
1	Global	15-29 years	2016	58633.51705
1	Global	15-29 years	2017	57576.00178
1	Global	15-29 years	2018	56708.50576
1	Global	15-29 years	2019	55988.88715
1	Global	15-29 years	2020	55302.32047
1	Global	15-29 years	2021	55839.88711
1	Global	15-29 years	2022	56893.12905
1	Global	15-29 years	2023	56737.03136
1	Global	15-29 years	2024	56532.08127
1	Global	15-29 years	2025	56229.58639
1	Global	15-29 years	2026	55869.02588
1	Global	15-29 years	2027	55493.31137
1	Global	15-29 years	2028	55143.53202
1	Global	15-29 years	2029	54844.74904
1	Global	15-29 years	2030	54597.87746
1	Global	15-29 years	2031	54395.83674
1	Global	15-29 years	2032	54235.10642
1	Global	15-29 years	2033	54080.58026
1	Global	15-29 years	2034	53959.93321
1	Global	15-29 years	2035	53862.80942

1	Global	15-29 years	2036	53778.43846
1	Global	30-44 years	1990	220811.248
1	Global	30-44 years	1991	229060.9951
1	Global	30-44 years	1992	238259.4365
1	Global	30-44 years	1993	250753.213
1	Global	30-44 years	1994	259353.28
1	Global	30-44 years	1995	261824.0004
1	Global	30-44 years	1996	260160.8902
1	Global	30-44 years	1997	259551.8682
1	Global	30-44 years	1998	263030.6698
1	Global	30-44 years	1999	271105.4865
1	Global	30-44 years	2000	274845.6805
1	Global	30-44 years	2001	277310.6975
1	Global	30-44 years	2002	283140.7964
1	Global	30-44 years	2003	287292.8704
1	Global	30-44 years	2004	291102.5145
1	Global	30-44 years	2005	293607.1939
1	Global	30-44 years	2006	294851.7144
1	Global	30-44 years	2007	297512.7264
1	Global	30-44 years	2008	298716.6678
1	Global	30-44 years	2009	295036.0032
1	Global	30-44 years	2010	295566.9598
1	Global	30-44 years	2011	295707.5871
1	Global	30-44 years	2012	298070.3524
1	Global	30-44 years	2013	300899.2777
1	Global	30-44 years	2014	301661.5909
1	Global	30-44 years	2015	297565.0587
1	Global	30-44 years	2016	301326.5187
1	Global	30-44 years	2017	301564.6531
1	Global	30-44 years	2018	301963.7972
1	Global	30-44 years	2019	301765.5566
1	Global	30-44 years	2020	303483.2682
1	Global	30-44 years	2021	309229.9711
1	Global	30-44 years	2022	323155.8862
1	Global	30-44 years	2023	326671.0646
1	Global	30-44 years	2024	330414.8519
1	Global	30-44 years	2025	334034.9413
1	Global	30-44 years	2026	337286.3671
1	Global	30-44 years	2027	339875.506
1	Global	30-44 years	2028	341646.0002
1	Global	30-44 years	2029	342756.5813
1	Global	30-44 years	2030	343422.8623
1	Global	30-44 years	2031	343852
1	Global	30-44 years	2032	344116.9826
1	Global	30-44 years	2033	343993.6123
1	Global	30-44 years	2034	343415.3076
1	Global	30-44 years	2035	342813.4456
1	Global	30-44 years	2036	342635.9918
1	Global	45-59 years	1990	765014.9823
1	Global	45-59 years	1991	768371.1239

1	Global	45-59 years	1992	784436.5145
1	Global	45-59 years	1993	817311.7402
1	Global	45-59 years	1994	841840.2716
1	Global	45-59 years	1995	844486.4795
1	Global	45-59 years	1996	838076.7106
1	Global	45-59 years	1997	832353.8602
1	Global	45-59 years	1998	829216.2079
1	Global	45-59 years	1999	846084.5399
1	Global	45-59 years	2000	861703.3054
1	Global	45-59 years	2001	875999.4597
1	Global	45-59 years	2002	899987.4615
1	Global	45-59 years	2003	927378.0733
1	Global	45-59 years	2004	945816.7491
1	Global	45-59 years	2005	980713.6523
1	Global	45-59 years	2006	989209.3901
1	Global	45-59 years	2007	1000590.745
1	Global	45-59 years	2008	1023363.728
1	Global	45-59 years	2009	1028336.613
1	Global	45-59 years	2010	1051023.225
1	Global	45-59 years	2011	1065925.918
1	Global	45-59 years	2012	1090559.93
1	Global	45-59 years	2013	1102015.773
1	Global	45-59 years	2014	1116071.801
1	Global	45-59 years	2015	1132844.978
1	Global	45-59 years	2016	1143575.011
1	Global	45-59 years	2017	1134860.134
1	Global	45-59 years	2018	1151954.666
1	Global	45-59 years	2019	1165298.822
1	Global	45-59 years	2020	1169869.2
1	Global	45-59 years	2021	1182636.184
1	Global	45-59 years	2022	1223217.526
1	Global	45-59 years	2023	1238980.319
1	Global	45-59 years	2024	1252874.505
1	Global	45-59 years	2025	1266170.233
1	Global	45-59 years	2026	1280436.75
1	Global	45-59 years	2027	1297135.776
1	Global	45-59 years	2028	1315405.551
1	Global	45-59 years	2029	1333391.055
1	Global	45-59 years	2030	1350994.56
1	Global	45-59 years	2031	1369317.72
1	Global	45-59 years	2032	1389699.618
1	Global	45-59 years	2033	1412234.018
1	Global	45-59 years	2034	1435649.012
1	Global	45-59 years	2035	1459011.011
1	Global	45-59 years	2036	1482126.799
1	Global	60-75 years	1990	1760489.887
1	Global	60-75 years	1991	1800647.752
1	Global	60-75 years	1992	1863643.899
1	Global	60-75 years	1993	1965736.123
1	Global	60-75 years	1994	2031442.057

1	Global	60-75 years	1995	2058489.964
1	Global	60-75 years	1996	2063739.915
1	Global	60-75 years	1997	2075551.949
1	Global	60-75 years	1998	2093908.99
1	Global	60-75 years	1999	2121948.581
1	Global	60-75 years	2000	2138661.627
1	Global	60-75 years	2001	2160851.893
1	Global	60-75 years	2002	2187881.467
1	Global	60-75 years	2003	2202725.554
1	Global	60-75 years	2004	2170351.317
1	Global	60-75 years	2005	2165070.524
1	Global	60-75 years	2006	2120746.555
1	Global	60-75 years	2007	2133110.882
1	Global	60-75 years	2008	2163938.669
1	Global	60-75 years	2009	2178292.232
1	Global	60-75 years	2010	2218711.367
1	Global	60-75 years	2011	2250961.541
1	Global	60-75 years	2012	2285235.727
1	Global	60-75 years	2013	2285404.224
1	Global	60-75 years	2014	2293592.874
1	Global	60-75 years	2015	2340114.669
1	Global	60-75 years	2016	2410014.33
1	Global	60-75 years	2017	2459091.755
1	Global	60-75 years	2018	2558958.688
1	Global	60-75 years	2019	2635655.665
1	Global	60-75 years	2020	2696757.981
1	Global	60-75 years	2021	2770853.071
1	Global	60-75 years	2022	2802897.656
1	Global	60-75 years	2023	2872740.441
1	Global	60-75 years	2024	2938588.2
1	Global	60-75 years	2025	2999341.395
1	Global	60-75 years	2026	3056844.244
1	Global	60-75 years	2027	3114480.054
1	Global	60-75 years	2028	3172741.856
1	Global	60-75 years	2029	3230029.886
1	Global	60-75 years	2030	3285967.984
1	Global	60-75 years	2031	3342709.326
1	Global	60-75 years	2032	3403673.137
1	Global	60-75 years	2033	3473810.328
1	Global	60-75 years	2034	3551670.917
1	Global	60-75 years	2035	3631964.398
1	Global	60-75 years	2036	3711911.65
1	Global	75+ years	1990	2476666.888
1	Global	75+ years	1991	2501418.179
1	Global	75+ years	1992	2537134.49
1	Global	75+ years	1993	2613499.849
1	Global	75+ years	1994	2646347.243
1	Global	75+ years	1995	2676673.47
1	Global	75+ years	1996	2684582.535
1	Global	75+ years	1997	2691641.562

1	Global	75+ years	1998	2726962.676
1	Global	75+ years	1999	2791079.457
1	Global	75+ years	2000	2832549.534
1	Global	75+ years	2001	2898194.842
1	Global	75+ years	2002	2992856.892
1	Global	75+ years	2003	3101650.789
1	Global	75+ years	2004	3149663.698
1	Global	75+ years	2005	3233904.208
1	Global	75+ years	2006	3248736.952
1	Global	75+ years	2007	3318194.655
1	Global	75+ years	2008	3425717.283
1	Global	75+ years	2009	3501914.247
1	Global	75+ years	2010	3607742.65
1	Global	75+ years	2011	3693388.962
1	Global	75+ years	2012	3780867.291
1	Global	75+ years	2013	3930868.557
1	Global	75+ years	2014	4048423.422
1	Global	75+ years	2015	4075620.425
1	Global	75+ years	2016	4121747.428
1	Global	75+ years	2017	4155360.205
1	Global	75+ years	2018	4212024.305
1	Global	75+ years	2019	4276931.983
1	Global	75+ years	2020	4326151.879
1	Global	75+ years	2021	4377912.665
1	Global	75+ years	2022	4531849.974
1	Global	75+ years	2023	4622471.868
1	Global	75+ years	2024	4734839.536
1	Global	75+ years	2025	4863204.373
1	Global	75+ years	2026	4999580.836
1	Global	75+ years	2027	5137337.376
1	Global	75+ years	2028	5287384.525
1	Global	75+ years	2029	5458974.057
1	Global	75+ years	2030	5645720.293
1	Global	75+ years	2031	5838565.929
1	Global	75+ years	2032	6030365.287
1	Global	75+ years	2033	6226982.644
1	Global	75+ years	2034	6436666.604
1	Global	75+ years	2035	6658453.556
1	Global	75+ years	2036	6890411.76
65	High-income Asia Pacific	15-29 years	1990	519.0056205
65	High-income Asia Pacific	15-29 years	1991	512.7275163
65	High-income Asia Pacific	15-29 years	1992	504.2256105
65	High-income Asia Pacific	15-29 years	1993	493.4608688
65	High-income Asia Pacific	15-29 years	1994	484.9346713
65	High-income Asia Pacific	15-29 years	1995	475.6496944
65	High-income Asia Pacific	15-29 years	1996	469.1817098
65	High-income Asia Pacific	15-29 years	1997	455.7075199
65	High-income Asia Pacific	15-29 years	1998	438.2690382
65	High-income Asia Pacific	15-29 years	1999	418.8314403
65	High-income Asia Pacific	15-29 years	2000	398.3823818

65	High-income Asia Pacific	15-29 years	2001	377.1399939
65	High-income Asia Pacific	15-29 years	2002	357.4402925
65	High-income Asia Pacific	15-29 years	2003	337.5149407
65	High-income Asia Pacific	15-29 years	2004	318.055986
65	High-income Asia Pacific	15-29 years	2005	301.472036
65	High-income Asia Pacific	15-29 years	2006	285.7177083
65	High-income Asia Pacific	15-29 years	2007	272.7755506
65	High-income Asia Pacific	15-29 years	2008	259.6902419
65	High-income Asia Pacific	15-29 years	2009	245.9501577
65	High-income Asia Pacific	15-29 years	2010	231.2653953
65	High-income Asia Pacific	15-29 years	2011	218.8049559
65	High-income Asia Pacific	15-29 years	2012	204.8223887
65	High-income Asia Pacific	15-29 years	2013	192.658421
65	High-income Asia Pacific	15-29 years	2014	180.5785958
65	High-income Asia Pacific	15-29 years	2015	170.0853142
65	High-income Asia Pacific	15-29 years	2016	160.9340383
65	High-income Asia Pacific	15-29 years	2017	152.4074206
65	High-income Asia Pacific	15-29 years	2018	144.2969013
65	High-income Asia Pacific	15-29 years	2019	137.2233323
65	High-income Asia Pacific	15-29 years	2020	131.7434944
65	High-income Asia Pacific	15-29 years	2021	125.4191155
65	High-income Asia Pacific	15-29 years	2022	120.9737279
65	High-income Asia Pacific	15-29 years	2023	115.0008898
65	High-income Asia Pacific	15-29 years	2024	109.1474864
65	High-income Asia Pacific	15-29 years	2025	103.4374743
65	High-income Asia Pacific	15-29 years	2026	97.89658422
65	High-income Asia Pacific	15-29 years	2027	92.5498228
65	High-income Asia Pacific	15-29 years	2028	87.28636978
65	High-income Asia Pacific	15-29 years	2029	82.11381656
65	High-income Asia Pacific	15-29 years	2030	77.20926609
65	High-income Asia Pacific	15-29 years	2031	72.71622558
65	High-income Asia Pacific	15-29 years	2032	68.73299413
65	High-income Asia Pacific	15-29 years	2033	65.34454816
65	High-income Asia Pacific	15-29 years	2034	62.40611562
65	High-income Asia Pacific	15-29 years	2035	59.74653438
65	High-income Asia Pacific	15-29 years	2036	57.22034116
65	High-income Asia Pacific	30-44 years	1990	2776.846649
65	High-income Asia Pacific	30-44 years	1991	2869.354788
65	High-income Asia Pacific	30-44 years	1992	2831.871679
65	High-income Asia Pacific	30-44 years	1993	2758.608222
65	High-income Asia Pacific	30-44 years	1994	2668.966411
65	High-income Asia Pacific	30-44 years	1995	2617.619034
65	High-income Asia Pacific	30-44 years	1996	2561.016233
65	High-income Asia Pacific	30-44 years	1997	2500.074609
65	High-income Asia Pacific	30-44 years	1998	2504.453083
65	High-income Asia Pacific	30-44 years	1999	2499.472156
65	High-income Asia Pacific	30-44 years	2000	2448.288199
65	High-income Asia Pacific	30-44 years	2001	2425.179732
65	High-income Asia Pacific	30-44 years	2002	2445.635435
65	High-income Asia Pacific	30-44 years	2003	2448.949526

65	High-income Asia Pacific	30-44 years	2004	2417.632378
65	High-income Asia Pacific	30-44 years	2005	2373.561507
65	High-income Asia Pacific	30-44 years	2006	2326.579443
65	High-income Asia Pacific	30-44 years	2007	2295.751651
65	High-income Asia Pacific	30-44 years	2008	2260.031882
65	High-income Asia Pacific	30-44 years	2009	2223.939491
65	High-income Asia Pacific	30-44 years	2010	2161.105393
65	High-income Asia Pacific	30-44 years	2011	2142.609421
65	High-income Asia Pacific	30-44 years	2012	2075.980782
65	High-income Asia Pacific	30-44 years	2013	2012.266541
65	High-income Asia Pacific	30-44 years	2014	1928.318457
65	High-income Asia Pacific	30-44 years	2015	1816.532319
65	High-income Asia Pacific	30-44 years	2016	1711.541206
65	High-income Asia Pacific	30-44 years	2017	1596.178956
65	High-income Asia Pacific	30-44 years	2018	1500.113973
65	High-income Asia Pacific	30-44 years	2019	1415.727825
65	High-income Asia Pacific	30-44 years	2020	1360.892656
65	High-income Asia Pacific	30-44 years	2021	1293.663308
65	High-income Asia Pacific	30-44 years	2022	1243.646011
65	High-income Asia Pacific	30-44 years	2023	1183.136869
65	High-income Asia Pacific	30-44 years	2024	1125.279345
65	High-income Asia Pacific	30-44 years	2025	1070.223846
65	High-income Asia Pacific	30-44 years	2026	1018.011557
65	High-income Asia Pacific	30-44 years	2027	968.6974606
65	High-income Asia Pacific	30-44 years	2028	922.3512597
65	High-income Asia Pacific	30-44 years	2029	878.6275757
65	High-income Asia Pacific	30-44 years	2030	837.2287179
65	High-income Asia Pacific	30-44 years	2031	797.8793263
65	High-income Asia Pacific	30-44 years	2032	760.3392511
65	High-income Asia Pacific	30-44 years	2033	725.2407554
65	High-income Asia Pacific	30-44 years	2034	692.8335662
65	High-income Asia Pacific	30-44 years	2035	662.3112153
65	High-income Asia Pacific	30-44 years	2036	632.9151655
65	High-income Asia Pacific	45-59 years	1990	11050.84389
65	High-income Asia Pacific	45-59 years	1991	10867.82364
65	High-income Asia Pacific	45-59 years	1992	10779.03636
65	High-income Asia Pacific	45-59 years	1993	10803.89827
65	High-income Asia Pacific	45-59 years	1994	10748.7122
65	High-income Asia Pacific	45-59 years	1995	10797.59685
65	High-income Asia Pacific	45-59 years	1996	10731.71773
65	High-income Asia Pacific	45-59 years	1997	10700.87114
65	High-income Asia Pacific	45-59 years	1998	10831.88797
65	High-income Asia Pacific	45-59 years	1999	10972.28876
65	High-income Asia Pacific	45-59 years	2000	10878.98836
65	High-income Asia Pacific	45-59 years	2001	10890.3882
65	High-income Asia Pacific	45-59 years	2002	10793.82356
65	High-income Asia Pacific	45-59 years	2003	10787.37673
65	High-income Asia Pacific	45-59 years	2004	10554.83033
65	High-income Asia Pacific	45-59 years	2005	10863.79484
65	High-income Asia Pacific	45-59 years	2006	11026.42345

65	High-income Asia Pacific	45-59 years	2007	10663.81223
65	High-income Asia Pacific	45-59 years	2008	10356.74695
65	High-income Asia Pacific	45-59 years	2009	10003.70047
65	High-income Asia Pacific	45-59 years	2010	9680.927901
65	High-income Asia Pacific	45-59 years	2011	9434.970341
65	High-income Asia Pacific	45-59 years	2012	9137.675688
65	High-income Asia Pacific	45-59 years	2013	8950.457143
65	High-income Asia Pacific	45-59 years	2014	8766.166896
65	High-income Asia Pacific	45-59 years	2015	8549.261226
65	High-income Asia Pacific	45-59 years	2016	8493.75141
65	High-income Asia Pacific	45-59 years	2017	8418.829486
65	High-income Asia Pacific	45-59 years	2018	8394.474809
65	High-income Asia Pacific	45-59 years	2019	8411.031848
65	High-income Asia Pacific	45-59 years	2020	8292.550448
65	High-income Asia Pacific	45-59 years	2021	8248.721627
65	High-income Asia Pacific	45-59 years	2022	8342.290418
65	High-income Asia Pacific	45-59 years	2023	8254.977272
65	High-income Asia Pacific	45-59 years	2024	8167.239081
65	High-income Asia Pacific	45-59 years	2025	8063.016322
65	High-income Asia Pacific	45-59 years	2026	7928.966774
65	High-income Asia Pacific	45-59 years	2027	7751.801
65	High-income Asia Pacific	45-59 years	2028	7525.202964
65	High-income Asia Pacific	45-59 years	2029	7262.633613
65	High-income Asia Pacific	45-59 years	2030	6979.573639
65	High-income Asia Pacific	45-59 years	2031	6690.365161
65	High-income Asia Pacific	45-59 years	2032	6408.076129
65	High-income Asia Pacific	45-59 years	2033	6128.234535
65	High-income Asia Pacific	45-59 years	2034	5845.147955
65	High-income Asia Pacific	45-59 years	2035	5568.394303
65	High-income Asia Pacific	45-59 years	2036	5306.262777
65	High-income Asia Pacific	60-75 years	1990	29838.21245
65	High-income Asia Pacific	60-75 years	1991	29818.38943
65	High-income Asia Pacific	60-75 years	1992	30286.3752
65	High-income Asia Pacific	60-75 years	1993	30914.64888
65	High-income Asia Pacific	60-75 years	1994	30920.76679
65	High-income Asia Pacific	60-75 years	1995	30872.4527
65	High-income Asia Pacific	60-75 years	1996	30685.76683
65	High-income Asia Pacific	60-75 years	1997	30683.27174
65	High-income Asia Pacific	60-75 years	1998	31148.24776
65	High-income Asia Pacific	60-75 years	1999	31542.96278
65	High-income Asia Pacific	60-75 years	2000	30773.09059
65	High-income Asia Pacific	60-75 years	2001	30455.63464
65	High-income Asia Pacific	60-75 years	2002	30228.98778
65	High-income Asia Pacific	60-75 years	2003	30355.04855
65	High-income Asia Pacific	60-75 years	2004	30037.96493
65	High-income Asia Pacific	60-75 years	2005	30244.2745
65	High-income Asia Pacific	60-75 years	2006	29454.42167
65	High-income Asia Pacific	60-75 years	2007	29083.69885
65	High-income Asia Pacific	60-75 years	2008	28933.44825
65	High-income Asia Pacific	60-75 years	2009	28682.17688

65	High-income Asia Pacific	60-75 years	2010	28788.00301
65	High-income Asia Pacific	60-75 years	2011	28799.94831
65	High-income Asia Pacific	60-75 years	2012	28365.13742
65	High-income Asia Pacific	60-75 years	2013	27755.31797
65	High-income Asia Pacific	60-75 years	2014	27458.44007
65	High-income Asia Pacific	60-75 years	2015	27012.75784
65	High-income Asia Pacific	60-75 years	2016	26720.42989
65	High-income Asia Pacific	60-75 years	2017	26205.43213
65	High-income Asia Pacific	60-75 years	2018	26153.23281
65	High-income Asia Pacific	60-75 years	2019	25746.84855
65	High-income Asia Pacific	60-75 years	2020	25485.5091
65	High-income Asia Pacific	60-75 years	2021	26306.82878
65	High-income Asia Pacific	60-75 years	2022	26955.39755
65	High-income Asia Pacific	60-75 years	2023	26707.1945
65	High-income Asia Pacific	60-75 years	2024	26234.32609
65	High-income Asia Pacific	60-75 years	2025	25687.79897
65	High-income Asia Pacific	60-75 years	2026	25222.48113
65	High-income Asia Pacific	60-75 years	2027	24970.15855
65	High-income Asia Pacific	60-75 years	2028	24942.30914
65	High-income Asia Pacific	60-75 years	2029	25029.36927
65	High-income Asia Pacific	60-75 years	2030	25170.94207
65	High-income Asia Pacific	60-75 years	2031	25316.97315
65	High-income Asia Pacific	60-75 years	2032	25410.41929
65	High-income Asia Pacific	60-75 years	2033	25441.54455
65	High-income Asia Pacific	60-75 years	2034	25435.00506
65	High-income Asia Pacific	60-75 years	2035	25386.43425
65	High-income Asia Pacific	60-75 years	2036	25300.80514
65	High-income Asia Pacific	75+ years	1990	71036.40448
65	High-income Asia Pacific	75+ years	1991	72285.24025
65	High-income Asia Pacific	75+ years	1992	73658.87637
65	High-income Asia Pacific	75+ years	1993	73823.19692
65	High-income Asia Pacific	75+ years	1994	71020.4515
65	High-income Asia Pacific	75+ years	1995	68724.21902
65	High-income Asia Pacific	75+ years	1996	66773.6286
65	High-income Asia Pacific	75+ years	1997	66540.00946
65	High-income Asia Pacific	75+ years	1998	68027.1605
65	High-income Asia Pacific	75+ years	1999	69682.41173
65	High-income Asia Pacific	75+ years	2000	68244.80847
65	High-income Asia Pacific	75+ years	2001	68226.99233
65	High-income Asia Pacific	75+ years	2002	69659.08346
65	High-income Asia Pacific	75+ years	2003	71890.4825
65	High-income Asia Pacific	75+ years	2004	73388.60112
65	High-income Asia Pacific	75+ years	2005	76751.49639
65	High-income Asia Pacific	75+ years	2006	78225.60648
65	High-income Asia Pacific	75+ years	2007	79525.78868
65	High-income Asia Pacific	75+ years	2008	81136.42039
65	High-income Asia Pacific	75+ years	2009	82139.68845
65	High-income Asia Pacific	75+ years	2010	84149.19541
65	High-income Asia Pacific	75+ years	2011	86743.45994
65	High-income Asia Pacific	75+ years	2012	88200.23076

65	High-income Asia Pacific	75+ years	2013	88489.13815
65	High-income Asia Pacific	75+ years	2014	88809.41047
65	High-income Asia Pacific	75+ years	2015	88648.24712
65	High-income Asia Pacific	75+ years	2016	90129.42638
65	High-income Asia Pacific	75+ years	2017	92094.34408
65	High-income Asia Pacific	75+ years	2018	94427.33756
65	High-income Asia Pacific	75+ years	2019	95351.7441
65	High-income Asia Pacific	75+ years	2020	94481.51281
65	High-income Asia Pacific	75+ years	2021	98641.96321
65	High-income Asia Pacific	75+ years	2022	91747.48922
65	High-income Asia Pacific	75+ years	2023	92645.19098
65	High-income Asia Pacific	75+ years	2024	93767.98108
65	High-income Asia Pacific	75+ years	2025	94975.84664
65	High-income Asia Pacific	75+ years	2026	96097.10454
65	High-income Asia Pacific	75+ years	2027	96929.06038
65	High-income Asia Pacific	75+ years	2028	97522.43766
65	High-income Asia Pacific	75+ years	2029	98074.07639
65	High-income Asia Pacific	75+ years	2030	98632.50338
65	High-income Asia Pacific	75+ years	2031	99219.03422
65	High-income Asia Pacific	75+ years	2032	99796.0749
65	High-income Asia Pacific	75+ years	2033	100412.3459
65	High-income Asia Pacific	75+ years	2034	101125.4295
65	High-income Asia Pacific	75+ years	2035	101939.1517
65	High-income Asia Pacific	75+ years	2036	102828.7872
100	High-income North America	15-29 years	1990	714.4559312
100	High-income North America	15-29 years	1991	698.3207822
100	High-income North America	15-29 years	1992	677.1495412
100	High-income North America	15-29 years	1993	662.520983
100	High-income North America	15-29 years	1994	645.7393384
100	High-income North America	15-29 years	1995	634.9870944
100	High-income North America	15-29 years	1996	626.8658402
100	High-income North America	15-29 years	1997	620.685668
100	High-income North America	15-29 years	1998	618.7563752
100	High-income North America	15-29 years	1999	629.654646
100	High-income North America	15-29 years	2000	633.9335578
100	High-income North America	15-29 years	2001	640.3655013
100	High-income North America	15-29 years	2002	638.5152476
100	High-income North America	15-29 years	2003	643.5166489
100	High-income North America	15-29 years	2004	648.1205857
100	High-income North America	15-29 years	2005	658.692492
100	High-income North America	15-29 years	2006	661.7061784
100	High-income North America	15-29 years	2007	660.7215012
100	High-income North America	15-29 years	2008	657.6844582
100	High-income North America	15-29 years	2009	646.95444
100	High-income North America	15-29 years	2010	639.2271372
100	High-income North America	15-29 years	2011	640.3493369
100	High-income North America	15-29 years	2012	632.1642253
100	High-income North America	15-29 years	2013	629.5492133
100	High-income North America	15-29 years	2014	629.3057018
100	High-income North America	15-29 years	2015	631.1186088

100	High-income North America	15-29 years	2016	626.5578897
100	High-income North America	15-29 years	2017	619.40413
100	High-income North America	15-29 years	2018	606.9798931
100	High-income North America	15-29 years	2019	590.2356346
100	High-income North America	15-29 years	2020	581.3105718
100	High-income North America	15-29 years	2021	567.1917998
100	High-income North America	15-29 years	2022	574.8946112
100	High-income North America	15-29 years	2023	567.8590498
100	High-income North America	15-29 years	2024	560.2093719
100	High-income North America	15-29 years	2025	551.4049659
100	High-income North America	15-29 years	2026	541.0634219
100	High-income North America	15-29 years	2027	529.4426221
100	High-income North America	15-29 years	2028	517.6690462
100	High-income North America	15-29 years	2029	506.4928552
100	High-income North America	15-29 years	2030	496.1516566
100	High-income North America	15-29 years	2031	486.6325419
100	High-income North America	15-29 years	2032	477.534365
100	High-income North America	15-29 years	2033	468.6578417
100	High-income North America	15-29 years	2034	460.1843293
100	High-income North America	15-29 years	2035	452.4768853
100	High-income North America	15-29 years	2036	445.8490229
100	High-income North America	30-44 years	1990	10082.38982
100	High-income North America	30-44 years	1991	10355.93372
100	High-income North America	30-44 years	1992	10401.85369
100	High-income North America	30-44 years	1993	10584.81854
100	High-income North America	30-44 years	1994	10666.30818
100	High-income North America	30-44 years	1995	10798.68695
100	High-income North America	30-44 years	1996	10609.65727
100	High-income North America	30-44 years	1997	10399.80472
100	High-income North America	30-44 years	1998	10352.45771
100	High-income North America	30-44 years	1999	10622.61809
100	High-income North America	30-44 years	2000	10572.49949
100	High-income North America	30-44 years	2001	10539.10791
100	High-income North America	30-44 years	2002	10499.68855
100	High-income North America	30-44 years	2003	10248.94391
100	High-income North America	30-44 years	2004	9791.484883
100	High-income North America	30-44 years	2005	9464.393109
100	High-income North America	30-44 years	2006	9122.322082
100	High-income North America	30-44 years	2007	8594.775972
100	High-income North America	30-44 years	2008	8178.805817
100	High-income North America	30-44 years	2009	7804.569838
100	High-income North America	30-44 years	2010	7466.804084
100	High-income North America	30-44 years	2011	7332.005127
100	High-income North America	30-44 years	2012	7194.052489
100	High-income North America	30-44 years	2013	7111.101045
100	High-income North America	30-44 years	2014	7025.568524
100	High-income North America	30-44 years	2015	7026.834806
100	High-income North America	30-44 years	2016	7047.663549
100	High-income North America	30-44 years	2017	6984.360046
100	High-income North America	30-44 years	2018	6895.818903

100	High-income North America	30-44 years	2019	6876.970269
100	High-income North America	30-44 years	2020	6965.519226
100	High-income North America	30-44 years	2021	6889.565471
100	High-income North America	30-44 years	2022	7834.545967
100	High-income North America	30-44 years	2023	7965.658209
100	High-income North America	30-44 years	2024	8069.111405
100	High-income North America	30-44 years	2025	8149.00826
100	High-income North America	30-44 years	2026	8212.191678
100	High-income North America	30-44 years	2027	8266.585292
100	High-income North America	30-44 years	2028	8316.645317
100	High-income North America	30-44 years	2029	8362.01856
100	High-income North America	30-44 years	2030	8399.287087
100	High-income North America	30-44 years	2031	8423.044981
100	High-income North America	30-44 years	2032	8426.869909
100	High-income North America	30-44 years	2033	8402.701135
100	High-income North America	30-44 years	2034	8355.786446
100	High-income North America	30-44 years	2035	8297.211031
100	High-income North America	30-44 years	2036	8237.297407
100	High-income North America	45-59 years	1990	51627.63661
100	High-income North America	45-59 years	1991	50617.71552
100	High-income North America	45-59 years	1992	50275.85882
100	High-income North America	45-59 years	1993	51034.28001
100	High-income North America	45-59 years	1994	51451.85175
100	High-income North America	45-59 years	1995	51956.28053
100	High-income North America	45-59 years	1996	51505.79075
100	High-income North America	45-59 years	1997	51140.72953
100	High-income North America	45-59 years	1998	51193.76276
100	High-income North America	45-59 years	1999	53270.09455
100	High-income North America	45-59 years	2000	54020.04684
100	High-income North America	45-59 years	2001	54649.79094
100	High-income North America	45-59 years	2002	55656.27979
100	High-income North America	45-59 years	2003	55541.2724
100	High-income North America	45-59 years	2004	54938.73326
100	High-income North America	45-59 years	2005	56005.24563
100	High-income North America	45-59 years	2006	56466.06723
100	High-income North America	45-59 years	2007	55105.18713
100	High-income North America	45-59 years	2008	54659.52262
100	High-income North America	45-59 years	2009	53978.33665
100	High-income North America	45-59 years	2010	53087.85957
100	High-income North America	45-59 years	2011	52891.87346
100	High-income North America	45-59 years	2012	52657.64901
100	High-income North America	45-59 years	2013	52485.30741
100	High-income North America	45-59 years	2014	52244.13595
100	High-income North America	45-59 years	2015	51906.80837
100	High-income North America	45-59 years	2016	52089.36237
100	High-income North America	45-59 years	2017	51143.48214
100	High-income North America	45-59 years	2018	49560.42715
100	High-income North America	45-59 years	2019	47947.87318
100	High-income North America	45-59 years	2020	46356.23286
100	High-income North America	45-59 years	2021	46501.75273

100	High-income North America	45-59 years	2022	45341.67841
100	High-income North America	45-59 years	2023	44659.85056
100	High-income North America	45-59 years	2024	44088.42461
100	High-income North America	45-59 years	2025	43663.51573
100	High-income North America	45-59 years	2026	43415.45587
100	High-income North America	45-59 years	2027	43344.56013
100	High-income North America	45-59 years	2028	43408.07553
100	High-income North America	45-59 years	2029	43595.81625
100	High-income North America	45-59 years	2030	43946.25395
100	High-income North America	45-59 years	2031	44488.64065
100	High-income North America	45-59 years	2032	45243.1854
100	High-income North America	45-59 years	2033	46241.15527
100	High-income North America	45-59 years	2034	47456.99381
100	High-income North America	45-59 years	2035	48805.89618
100	High-income North America	45-59 years	2036	50187.76264
100	High-income North America	60-75 years	1990	190903.7452
100	High-income North America	60-75 years	1991	186545.9525
100	High-income North America	60-75 years	1992	181723.711
100	High-income North America	60-75 years	1993	181074.5876
100	High-income North America	60-75 years	1994	177075.2289
100	High-income North America	60-75 years	1995	173145.0556
100	High-income North America	60-75 years	1996	166294.3829
100	High-income North America	60-75 years	1997	158741.3648
100	High-income North America	60-75 years	1998	153775.0079
100	High-income North America	60-75 years	1999	151360.1145
100	High-income North America	60-75 years	2000	144031.5876
100	High-income North America	60-75 years	2001	137779.5615
100	High-income North America	60-75 years	2002	132902.0333
100	High-income North America	60-75 years	2003	127395.4261
100	High-income North America	60-75 years	2004	120826.3532
100	High-income North America	60-75 years	2005	117406.5515
100	High-income North America	60-75 years	2006	112855.1174
100	High-income North America	60-75 years	2007	110212.732
100	High-income North America	60-75 years	2008	109992.5227
100	High-income North America	60-75 years	2009	109210.6062
100	High-income North America	60-75 years	2010	108298.7801
100	High-income North America	60-75 years	2011	109803.7716
100	High-income North America	60-75 years	2012	112060.8165
100	High-income North America	60-75 years	2013	115011.837
100	High-income North America	60-75 years	2014	118490.1653
100	High-income North America	60-75 years	2015	122889.7874
100	High-income North America	60-75 years	2016	128316.9246
100	High-income North America	60-75 years	2017	131878.7713
100	High-income North America	60-75 years	2018	133230.7593
100	High-income North America	60-75 years	2019	134697.4291
100	High-income North America	60-75 years	2020	136246.3601
100	High-income North America	60-75 years	2021	142938.1847
100	High-income North America	60-75 years	2022	137197.355
100	High-income North America	60-75 years	2023	138836.3122
100	High-income North America	60-75 years	2024	140043.4229

100	High-income North America	60-75 years	2025	140881.7622
100	High-income North America	60-75 years	2026	141452.1603
100	High-income North America	60-75 years	2027	141792.7358
100	High-income North America	60-75 years	2028	141888.9722
100	High-income North America	60-75 years	2029	141685.1847
100	High-income North America	60-75 years	2030	141202.6034
100	High-income North America	60-75 years	2031	140504.8247
100	High-income North America	60-75 years	2032	139571.1415
100	High-income North America	60-75 years	2033	138220.2798
100	High-income North America	60-75 years	2034	136492.1469
100	High-income North America	60-75 years	2035	134719.0314
100	High-income North America	60-75 years	2036	133239.8286
100	High-income North America	75+ years	1990	360617.0935
100	High-income North America	75+ years	1991	359741.0682
100	High-income North America	75+ years	1992	360593.336
100	High-income North America	75+ years	1993	371282.1285
100	High-income North America	75+ years	1994	374317.3129
100	High-income North America	75+ years	1995	377669.1286
100	High-income North America	75+ years	1996	376199.9911
100	High-income North America	75+ years	1997	375370.0534
100	High-income North America	75+ years	1998	378229.7277
100	High-income North America	75+ years	1999	388172.9178
100	High-income North America	75+ years	2000	382577.2775
100	High-income North America	75+ years	2001	378211.2799
100	High-income North America	75+ years	2002	373468.6902
100	High-income North America	75+ years	2003	364331.7445
100	High-income North America	75+ years	2004	349846.4219
100	High-income North America	75+ years	2005	343626.9911
100	High-income North America	75+ years	2006	332153.6033
100	High-income North America	75+ years	2007	320289.0236
100	High-income North America	75+ years	2008	315352.2742
100	High-income North America	75+ years	2009	305821.5841
100	High-income North America	75+ years	2010	298332.6196
100	High-income North America	75+ years	2011	296081.7359
100	High-income North America	75+ years	2012	293176.7566
100	High-income North America	75+ years	2013	291622.7899
100	High-income North America	75+ years	2014	289211.5081
100	High-income North America	75+ years	2015	286848.4153
100	High-income North America	75+ years	2016	285394.9888
100	High-income North America	75+ years	2017	284908.0353
100	High-income North America	75+ years	2018	283900.4753
100	High-income North America	75+ years	2019	282672.6942
100	High-income North America	75+ years	2020	281969.3194
100	High-income North America	75+ years	2021	289237.7639
100	High-income North America	75+ years	2022	272252.0815
100	High-income North America	75+ years	2023	273471.889
100	High-income North America	75+ years	2024	275970.0597
100	High-income North America	75+ years	2025	279621.287
100	High-income North America	75+ years	2026	284114.6639
100	High-income North America	75+ years	2027	289063.4601

100	High-income North America	75+ years	2028	295107.923
100	High-income North America	75+ years	2029	302618.9044
100	High-income North America	75+ years	2030	311134.6189
100	High-income North America	75+ years	2031	320185.8864
100	High-income North America	75+ years	2032	329275.0209
100	High-income North America	75+ years	2033	339257.2345
100	High-income North America	75+ years	2034	350561.8138
100	High-income North America	75+ years	2035	362468.3745
100	High-income North America	75+ years	2036	374313.0368
138	North Africa and Middle East	15-29 years	1990	7471.527747
138	North Africa and Middle East	15-29 years	1991	7466.675333
138	North Africa and Middle East	15-29 years	1992	7544.356907
138	North Africa and Middle East	15-29 years	1993	7718.661202
138	North Africa and Middle East	15-29 years	1994	7886.464639
138	North Africa and Middle East	15-29 years	1995	7971.303492
138	North Africa and Middle East	15-29 years	1996	8119.315983
138	North Africa and Middle East	15-29 years	1997	8241.816894
138	North Africa and Middle East	15-29 years	1998	8279.735258
138	North Africa and Middle East	15-29 years	1999	8382.412159
138	North Africa and Middle East	15-29 years	2000	8375.451441
138	North Africa and Middle East	15-29 years	2001	8478.488947
138	North Africa and Middle East	15-29 years	2002	8588.387773
138	North Africa and Middle East	15-29 years	2003	8693.244068
138	North Africa and Middle East	15-29 years	2004	8799.137375
138	North Africa and Middle East	15-29 years	2005	8920.92993
138	North Africa and Middle East	15-29 years	2006	9072.16005
138	North Africa and Middle East	15-29 years	2007	9186.20709
138	North Africa and Middle East	15-29 years	2008	9361.830312
138	North Africa and Middle East	15-29 years	2009	9501.135234
138	North Africa and Middle East	15-29 years	2010	9595.520151
138	North Africa and Middle East	15-29 years	2011	9565.399961
138	North Africa and Middle East	15-29 years	2012	9557.560685
138	North Africa and Middle East	15-29 years	2013	9499.599728
138	North Africa and Middle East	15-29 years	2014	9337.209088
138	North Africa and Middle East	15-29 years	2015	9273.10935
138	North Africa and Middle East	15-29 years	2016	8998.534608
138	North Africa and Middle East	15-29 years	2017	8753.285587
138	North Africa and Middle East	15-29 years	2018	8685.290244
138	North Africa and Middle East	15-29 years	2019	8683.119842
138	North Africa and Middle East	15-29 years	2020	8596.968353
138	North Africa and Middle East	15-29 years	2021	8556.720379
138	North Africa and Middle East	15-29 years	2022	8917.335395
138	North Africa and Middle East	15-29 years	2023	8899.278567
138	North Africa and Middle East	15-29 years	2024	8881.053851
138	North Africa and Middle East	15-29 years	2025	8861.835941
138	North Africa and Middle East	15-29 years	2026	8842.184608
138	North Africa and Middle East	15-29 years	2027	8823.503637
138	North Africa and Middle East	15-29 years	2028	8813.657312
138	North Africa and Middle East	15-29 years	2029	8813.471192
138	North Africa and Middle East	15-29 years	2030	8816.748142

138	North Africa and Middle East	15-29 years	2031	8815.681891
138	North Africa and Middle East	15-29 years	2032	8803.750875
138	North Africa and Middle East	15-29 years	2033	8793.569898
138	North Africa and Middle East	15-29 years	2034	8791.214809
138	North Africa and Middle East	15-29 years	2035	8790.968836
138	North Africa and Middle East	15-29 years	2036	8785.963479
138	North Africa and Middle East	30-44 years	1990	22406.62067
138	North Africa and Middle East	30-44 years	1991	22903.09919
138	North Africa and Middle East	30-44 years	1992	23428.27355
138	North Africa and Middle East	30-44 years	1993	24102.76808
138	North Africa and Middle East	30-44 years	1994	24752.99002
138	North Africa and Middle East	30-44 years	1995	25201.77929
138	North Africa and Middle East	30-44 years	1996	25675.4248
138	North Africa and Middle East	30-44 years	1997	26151.05016
138	North Africa and Middle East	30-44 years	1998	26249.82482
138	North Africa and Middle East	30-44 years	1999	26517.42181
138	North Africa and Middle East	30-44 years	2000	26530.90876
138	North Africa and Middle East	30-44 years	2001	26961.30399
138	North Africa and Middle East	30-44 years	2002	27402.45077
138	North Africa and Middle East	30-44 years	2003	27942.53693
138	North Africa and Middle East	30-44 years	2004	28444.20432
138	North Africa and Middle East	30-44 years	2005	28926.38415
138	North Africa and Middle East	30-44 years	2006	29595.5146
138	North Africa and Middle East	30-44 years	2007	30134.59388
138	North Africa and Middle East	30-44 years	2008	30848.66362
138	North Africa and Middle East	30-44 years	2009	31532.4217
138	North Africa and Middle East	30-44 years	2010	32015.9674
138	North Africa and Middle East	30-44 years	2011	32477.90195
138	North Africa and Middle East	30-44 years	2012	33477.7497
138	North Africa and Middle East	30-44 years	2013	34168.05854
138	North Africa and Middle East	30-44 years	2014	34519.33518
138	North Africa and Middle East	30-44 years	2015	35392.77609
138	North Africa and Middle East	30-44 years	2016	35418.57363
138	North Africa and Middle East	30-44 years	2017	35728.22411
138	North Africa and Middle East	30-44 years	2018	36459.98813
138	North Africa and Middle East	30-44 years	2019	37320.99299
138	North Africa and Middle East	30-44 years	2020	37619.68095
138	North Africa and Middle East	30-44 years	2021	38324.98633
138	North Africa and Middle East	30-44 years	2022	40663.9068
138	North Africa and Middle East	30-44 years	2023	41128.29477
138	North Africa and Middle East	30-44 years	2024	41468.12747
138	North Africa and Middle East	30-44 years	2025	41712.43009
138	North Africa and Middle East	30-44 years	2026	41891.49819
138	North Africa and Middle East	30-44 years	2027	42027.88023
138	North Africa and Middle East	30-44 years	2028	42073.92585
138	North Africa and Middle East	30-44 years	2029	42004.54467
138	North Africa and Middle East	30-44 years	2030	41865.73155
138	North Africa and Middle East	30-44 years	2031	41706.15596
138	North Africa and Middle East	30-44 years	2032	41577.66558
138	North Africa and Middle East	30-44 years	2033	41462.59805

138	North Africa and Middle East	30-44 years	2034	41327.42305
138	North Africa and Middle East	30-44 years	2035	41202.02755
138	North Africa and Middle East	30-44 years	2036	41115.13972
138	North Africa and Middle East	45-59 years	1990	76411.70184
138	North Africa and Middle East	45-59 years	1991	77284.03702
138	North Africa and Middle East	45-59 years	1992	77914.01003
138	North Africa and Middle East	45-59 years	1993	79211.83768
138	North Africa and Middle East	45-59 years	1994	80401.97408
138	North Africa and Middle East	45-59 years	1995	81076.75303
138	North Africa and Middle East	45-59 years	1996	82100.96309
138	North Africa and Middle East	45-59 years	1997	83518.10946
138	North Africa and Middle East	45-59 years	1998	84143.73922
138	North Africa and Middle East	45-59 years	1999	85428.56317
138	North Africa and Middle East	45-59 years	2000	86002.08588
138	North Africa and Middle East	45-59 years	2001	88520.7612
138	North Africa and Middle East	45-59 years	2002	90570.35844
138	North Africa and Middle East	45-59 years	2003	93259.20209
138	North Africa and Middle East	45-59 years	2004	95550.36155
138	North Africa and Middle East	45-59 years	2005	97573.63071
138	North Africa and Middle East	45-59 years	2006	99998.91534
138	North Africa and Middle East	45-59 years	2007	101592.0546
138	North Africa and Middle East	45-59 years	2008	104222.6507
138	North Africa and Middle East	45-59 years	2009	107602.5139
138	North Africa and Middle East	45-59 years	2010	109819.9061
138	North Africa and Middle East	45-59 years	2011	111135.2448
138	North Africa and Middle East	45-59 years	2012	113409.7793
138	North Africa and Middle East	45-59 years	2013	114928.4857
138	North Africa and Middle East	45-59 years	2014	117000.2482
138	North Africa and Middle East	45-59 years	2015	120955.5004
138	North Africa and Middle East	45-59 years	2016	121043.238
138	North Africa and Middle East	45-59 years	2017	122070.9746
138	North Africa and Middle East	45-59 years	2018	125569.5352
138	North Africa and Middle East	45-59 years	2019	129207.9669
138	North Africa and Middle East	45-59 years	2020	131614.2276
138	North Africa and Middle East	45-59 years	2021	135857.7523
138	North Africa and Middle East	45-59 years	2022	144610.8123
138	North Africa and Middle East	45-59 years	2023	148586.0216
138	North Africa and Middle East	45-59 years	2024	152724.8823
138	North Africa and Middle East	45-59 years	2025	156977.4432
138	North Africa and Middle East	45-59 years	2026	161321.8445
138	North Africa and Middle East	45-59 years	2027	165717.9622
138	North Africa and Middle East	45-59 years	2028	170230.5758
138	North Africa and Middle East	45-59 years	2029	174899.2672
138	North Africa and Middle East	45-59 years	2030	179635.0384
138	North Africa and Middle East	45-59 years	2031	184367.6699
138	North Africa and Middle East	45-59 years	2032	189001.3139
138	North Africa and Middle East	45-59 years	2033	193645.0856
138	North Africa and Middle East	45-59 years	2034	198356.9948
138	North Africa and Middle East	45-59 years	2035	202927.1547
138	North Africa and Middle East	45-59 years	2036	207171.4953

138	North Africa and Middle East	60-75 years	1990	146808.2297
138	North Africa and Middle East	60-75 years	1991	152562.9424
138	North Africa and Middle East	60-75 years	1992	157563.9454
138	North Africa and Middle East	60-75 years	1993	163029.1316
138	North Africa and Middle East	60-75 years	1994	167470.263
138	North Africa and Middle East	60-75 years	1995	170147.6394
138	North Africa and Middle East	60-75 years	1996	172754.5966
138	North Africa and Middle East	60-75 years	1997	176240.9676
138	North Africa and Middle East	60-75 years	1998	178422.3316
138	North Africa and Middle East	60-75 years	1999	180016.1179
138	North Africa and Middle East	60-75 years	2000	179152.0638
138	North Africa and Middle East	60-75 years	2001	181884.2008
138	North Africa and Middle East	60-75 years	2002	182927.5078
138	North Africa and Middle East	60-75 years	2003	183949.7173
138	North Africa and Middle East	60-75 years	2004	183204.4771
138	North Africa and Middle East	60-75 years	2005	182301.0304
138	North Africa and Middle East	60-75 years	2006	182810.3544
138	North Africa and Middle East	60-75 years	2007	182946.2399
138	North Africa and Middle East	60-75 years	2008	185528.5027
138	North Africa and Middle East	60-75 years	2009	189289.283
138	North Africa and Middle East	60-75 years	2010	191282.6019
138	North Africa and Middle East	60-75 years	2011	192887.7858
138	North Africa and Middle East	60-75 years	2012	198363.3677
138	North Africa and Middle East	60-75 years	2013	203413.9139
138	North Africa and Middle East	60-75 years	2014	209947.6144
138	North Africa and Middle East	60-75 years	2015	218678.0626
138	North Africa and Middle East	60-75 years	2016	221560.1578
138	North Africa and Middle East	60-75 years	2017	225888.4688
138	North Africa and Middle East	60-75 years	2018	234908.7162
138	North Africa and Middle East	60-75 years	2019	244342.6724
138	North Africa and Middle East	60-75 years	2020	251503.0167
138	North Africa and Middle East	60-75 years	2021	259150.2344
138	North Africa and Middle East	60-75 years	2022	281418.8595
138	North Africa and Middle East	60-75 years	2023	291992.5001
138	North Africa and Middle East	60-75 years	2024	302802.8293
138	North Africa and Middle East	60-75 years	2025	313636.5643
138	North Africa and Middle East	60-75 years	2026	324376.3111
138	North Africa and Middle East	60-75 years	2027	335031.0879
138	North Africa and Middle East	60-75 years	2028	345679.663
138	North Africa and Middle East	60-75 years	2029	356457.3193
138	North Africa and Middle East	60-75 years	2030	367431.2721
138	North Africa and Middle East	60-75 years	2031	378709.1887
138	North Africa and Middle East	60-75 years	2032	390401.3821
138	North Africa and Middle East	60-75 years	2033	402643.6384
138	North Africa and Middle East	60-75 years	2034	415433.3097
138	North Africa and Middle East	60-75 years	2035	428669.2089
138	North Africa and Middle East	60-75 years	2036	442359.4485
138	North Africa and Middle East	75+ years	1990	128887.65
138	North Africa and Middle East	75+ years	1991	128813.4591
138	North Africa and Middle East	75+ years	1992	130656.6694

138	North Africa and Middle East	75+ years	1993	134462.728
138	North Africa and Middle East	75+ years	1994	138606.5449
138	North Africa and Middle East	75+ years	1995	142726.6008
138	North Africa and Middle East	75+ years	1996	146690.0573
138	North Africa and Middle East	75+ years	1997	151277.0905
138	North Africa and Middle East	75+ years	1998	156181.7268
138	North Africa and Middle East	75+ years	1999	160891.5218
138	North Africa and Middle East	75+ years	2000	165051.7349
138	North Africa and Middle East	75+ years	2001	172419.337
138	North Africa and Middle East	75+ years	2002	179256.0748
138	North Africa and Middle East	75+ years	2003	185846.4135
138	North Africa and Middle East	75+ years	2004	191568.3941
138	North Africa and Middle East	75+ years	2005	197400.9281
138	North Africa and Middle East	75+ years	2006	204216.0054
138	North Africa and Middle East	75+ years	2007	212062.3501
138	North Africa and Middle East	75+ years	2008	221650.2511
138	North Africa and Middle East	75+ years	2009	231484.1628
138	North Africa and Middle East	75+ years	2010	237925.4595
138	North Africa and Middle East	75+ years	2011	243424.6976
138	North Africa and Middle East	75+ years	2012	251064.7418
138	North Africa and Middle East	75+ years	2013	261049.0017
138	North Africa and Middle East	75+ years	2014	272421.8211
138	North Africa and Middle East	75+ years	2015	284749.6978
138	North Africa and Middle East	75+ years	2016	291303.2954
138	North Africa and Middle East	75+ years	2017	295262.9744
138	North Africa and Middle East	75+ years	2018	299633.4409
138	North Africa and Middle East	75+ years	2019	310764.5064
138	North Africa and Middle East	75+ years	2020	317318.5812
138	North Africa and Middle East	75+ years	2021	314488.2073
138	North Africa and Middle East	75+ years	2022	364855.3501
138	North Africa and Middle East	75+ years	2023	376498.7825
138	North Africa and Middle East	75+ years	2024	389816.7466
138	North Africa and Middle East	75+ years	2025	404612.7051
138	North Africa and Middle East	75+ years	2026	420668.9173
138	North Africa and Middle East	75+ years	2027	437737.8991
138	North Africa and Middle East	75+ years	2028	456827.9153
138	North Africa and Middle East	75+ years	2029	478546.4715
138	North Africa and Middle East	75+ years	2030	502115.5903
138	North Africa and Middle East	75+ years	2031	526878.8449
138	North Africa and Middle East	75+ years	2032	552291.2615
138	North Africa and Middle East	75+ years	2033	579357.1148
138	North Africa and Middle East	75+ years	2034	608878.43
138	North Africa and Middle East	75+ years	2035	640113.0361
138	North Africa and Middle East	75+ years	2036	672506.3262
21	Oceania	15-29 years	1990	70.14271986
21	Oceania	15-29 years	1991	71.85069849
21	Oceania	15-29 years	1992	73.45408767
21	Oceania	15-29 years	1993	74.99034064
21	Oceania	15-29 years	1994	76.52791107
21	Oceania	15-29 years	1995	78.161586

21	Oceania	15-29 years	1996	79.92457776
21	Oceania	15-29 years	1997	81.85585387
21	Oceania	15-29 years	1998	83.97889513
21	Oceania	15-29 years	1999	86.32832356
21	Oceania	15-29 years	2000	88.84846417
21	Oceania	15-29 years	2001	91.30910189
21	Oceania	15-29 years	2002	93.66240341
21	Oceania	15-29 years	2003	96.06647124
21	Oceania	15-29 years	2004	98.69958049
21	Oceania	15-29 years	2005	101.5961345
21	Oceania	15-29 years	2006	104.6799037
21	Oceania	15-29 years	2007	107.904183
21	Oceania	15-29 years	2008	111.1301796
21	Oceania	15-29 years	2009	114.3604793
21	Oceania	15-29 years	2010	117.7009445
21	Oceania	15-29 years	2011	121.0080333
21	Oceania	15-29 years	2012	124.1795549
21	Oceania	15-29 years	2013	127.2794368
21	Oceania	15-29 years	2014	130.4005967
21	Oceania	15-29 years	2015	133.5447091
21	Oceania	15-29 years	2016	136.6002798
21	Oceania	15-29 years	2017	139.7323689
21	Oceania	15-29 years	2018	142.9546001
21	Oceania	15-29 years	2019	146.2595432
21	Oceania	15-29 years	2020	149.4136374
21	Oceania	15-29 years	2021	152.4447282
21	Oceania	15-29 years	2022	151.8004249
21	Oceania	15-29 years	2023	153.9223814
21	Oceania	15-29 years	2024	155.9393822
21	Oceania	15-29 years	2025	157.9690455
21	Oceania	15-29 years	2026	160.1349835
21	Oceania	15-29 years	2027	162.5670207
21	Oceania	15-29 years	2028	165.1885884
21	Oceania	15-29 years	2029	167.9381738
21	Oceania	15-29 years	2030	170.9586595
21	Oceania	15-29 years	2031	174.4018532
21	Oceania	15-29 years	2032	178.5601881
21	Oceania	15-29 years	2033	183.5746996
21	Oceania	15-29 years	2034	189.2863782
21	Oceania	15-29 years	2035	195.4989495
21	Oceania	15-29 years	2036	202.0370239
21	Oceania	30-44 years	1990	496.2916454
21	Oceania	30-44 years	1991	511.0194423
21	Oceania	30-44 years	1992	521.0475086
21	Oceania	30-44 years	1993	533.3761242
21	Oceania	30-44 years	1994	549.8128775
21	Oceania	30-44 years	1995	572.0719119
21	Oceania	30-44 years	1996	589.2775588
21	Oceania	30-44 years	1997	608.0036492
21	Oceania	30-44 years	1998	628.3079538

21	Oceania	30-44 years	1999	650.7446095
21	Oceania	30-44 years	2000	673.0131229
21	Oceania	30-44 years	2001	693.2842491
21	Oceania	30-44 years	2002	713.6197579
21	Oceania	30-44 years	2003	734.0219498
21	Oceania	30-44 years	2004	754.2444665
21	Oceania	30-44 years	2005	776.3019532
21	Oceania	30-44 years	2006	799.3402615
21	Oceania	30-44 years	2007	822.8681131
21	Oceania	30-44 years	2008	844.9553067
21	Oceania	30-44 years	2009	865.8489934
21	Oceania	30-44 years	2010	888.8939493
21	Oceania	30-44 years	2011	911.3358511
21	Oceania	30-44 years	2012	932.5761508
21	Oceania	30-44 years	2013	952.7085046
21	Oceania	30-44 years	2014	974.398369
21	Oceania	30-44 years	2015	997.9326159
21	Oceania	30-44 years	2016	1020.01897
21	Oceania	30-44 years	2017	1044.504331
21	Oceania	30-44 years	2018	1069.660649
21	Oceania	30-44 years	2019	1097.231782
21	Oceania	30-44 years	2020	1123.429005
21	Oceania	30-44 years	2021	1148.99875
21	Oceania	30-44 years	2022	1140.328699
21	Oceania	30-44 years	2023	1162.05542
21	Oceania	30-44 years	2024	1184.415626
21	Oceania	30-44 years	2025	1207.318257
21	Oceania	30-44 years	2026	1230.723161
21	Oceania	30-44 years	2027	1254.652287
21	Oceania	30-44 years	2028	1279.473312
21	Oceania	30-44 years	2029	1305.287537
21	Oceania	30-44 years	2030	1331.743614
21	Oceania	30-44 years	2031	1358.567373
21	Oceania	30-44 years	2032	1385.489086
21	Oceania	30-44 years	2033	1412.758381
21	Oceania	30-44 years	2034	1440.707678
21	Oceania	30-44 years	2035	1469.167464
21	Oceania	30-44 years	2036	1498.080176
21	Oceania	45-59 years	1990	1349.744915
21	Oceania	45-59 years	1991	1372.033929
21	Oceania	45-59 years	1992	1399.164627
21	Oceania	45-59 years	1993	1427.498336
21	Oceania	45-59 years	1994	1454.056512
21	Oceania	45-59 years	1995	1477.914109
21	Oceania	45-59 years	1996	1522.965017
21	Oceania	45-59 years	1997	1565.501701
21	Oceania	45-59 years	1998	1607.132888
21	Oceania	45-59 years	1999	1653.76871
21	Oceania	45-59 years	2000	1698.795124
21	Oceania	45-59 years	2001	1765.310641

21	Oceania	45-59 years	2002	1824.399021
21	Oceania	45-59 years	2003	1885.347324
21	Oceania	45-59 years	2004	1945.114906
21	Oceania	45-59 years	2005	2010.849974
21	Oceania	45-59 years	2006	2089.159189
21	Oceania	45-59 years	2007	2155.964692
21	Oceania	45-59 years	2008	2230.036374
21	Oceania	45-59 years	2009	2312.855836
21	Oceania	45-59 years	2010	2418.897553
21	Oceania	45-59 years	2011	2503.545537
21	Oceania	45-59 years	2012	2586.489457
21	Oceania	45-59 years	2013	2665.314768
21	Oceania	45-59 years	2014	2746.576695
21	Oceania	45-59 years	2015	2828.101988
21	Oceania	45-59 years	2016	2900.372649
21	Oceania	45-59 years	2017	2980.399252
21	Oceania	45-59 years	2018	3060.657786
21	Oceania	45-59 years	2019	3148.021383
21	Oceania	45-59 years	2020	3225.456013
21	Oceania	45-59 years	2021	3295.545286
21	Oceania	45-59 years	2022	3245.719289
21	Oceania	45-59 years	2023	3303.719426
21	Oceania	45-59 years	2024	3361.935837
21	Oceania	45-59 years	2025	3421.733926
21	Oceania	45-59 years	2026	3484.254651
21	Oceania	45-59 years	2027	3550.631053
21	Oceania	45-59 years	2028	3620.279137
21	Oceania	45-59 years	2029	3692.522913
21	Oceania	45-59 years	2030	3767.970163
21	Oceania	45-59 years	2031	3847.283512
21	Oceania	45-59 years	2032	3931.145049
21	Oceania	45-59 years	2033	4019.007978
21	Oceania	45-59 years	2034	4110.440102
21	Oceania	45-59 years	2035	4205.859357
21	Oceania	45-59 years	2036	4305.878048
21	Oceania	60-75 years	1990	1788.53766
21	Oceania	60-75 years	1991	1826.208286
21	Oceania	60-75 years	1992	1875.365736
21	Oceania	60-75 years	1993	1919.370412
21	Oceania	60-75 years	1994	1958.435407
21	Oceania	60-75 years	1995	1993.901554
21	Oceania	60-75 years	1996	2007.120016
21	Oceania	60-75 years	1997	2044.426217
21	Oceania	60-75 years	1998	2083.342717
21	Oceania	60-75 years	1999	2129.359106
21	Oceania	60-75 years	2000	2177.060771
21	Oceania	60-75 years	2001	2194.155801
21	Oceania	60-75 years	2002	2240.272577
21	Oceania	60-75 years	2003	2289.830657
21	Oceania	60-75 years	2004	2342.164569

21	Oceania	60-75 years	2005	2409.410316
21	Oceania	60-75 years	2006	2486.812392
21	Oceania	60-75 years	2007	2586.374459
21	Oceania	60-75 years	2008	2682.406608
21	Oceania	60-75 years	2009	2766.685007
21	Oceania	60-75 years	2010	2839.055224
21	Oceania	60-75 years	2011	2948.967787
21	Oceania	60-75 years	2012	3052.144762
21	Oceania	60-75 years	2013	3149.046306
21	Oceania	60-75 years	2014	3245.927621
21	Oceania	60-75 years	2015	3341.146294
21	Oceania	60-75 years	2016	3467.102695
21	Oceania	60-75 years	2017	3588.709402
21	Oceania	60-75 years	2018	3710.554501
21	Oceania	60-75 years	2019	3834.694889
21	Oceania	60-75 years	2020	3947.957717
21	Oceania	60-75 years	2021	4056.49669
21	Oceania	60-75 years	2022	3969.706075
21	Oceania	60-75 years	2023	4082.332221
21	Oceania	60-75 years	2024	4203.207891
21	Oceania	60-75 years	2025	4328.396745
21	Oceania	60-75 years	2026	4454.357745
21	Oceania	60-75 years	2027	4578.082148
21	Oceania	60-75 years	2028	4701.988355
21	Oceania	60-75 years	2029	4830.010154
21	Oceania	60-75 years	2030	4961.515184
21	Oceania	60-75 years	2031	5095.833971
21	Oceania	60-75 years	2032	5232.47839
21	Oceania	60-75 years	2033	5374.215972
21	Oceania	60-75 years	2034	5523.314754
21	Oceania	60-75 years	2035	5678.278054
21	Oceania	60-75 years	2036	5838.003169
21	Oceania	75+ years	1990	825.0557062
21	Oceania	75+ years	1991	867.6527132
21	Oceania	75+ years	1992	910.5771897
21	Oceania	75+ years	1993	957.1096618
21	Oceania	75+ years	1994	1007.863694
21	Oceania	75+ years	1995	1063.125903
21	Oceania	75+ years	1996	1124.740139
21	Oceania	75+ years	1997	1181.963345
21	Oceania	75+ years	1998	1240.811391
21	Oceania	75+ years	1999	1303.373464
21	Oceania	75+ years	2000	1367.926784
21	Oceania	75+ years	2001	1425.596224
21	Oceania	75+ years	2002	1484.030089
21	Oceania	75+ years	2003	1543.648226
21	Oceania	75+ years	2004	1603.613289
21	Oceania	75+ years	2005	1665.633949
21	Oceania	75+ years	2006	1704.449157
21	Oceania	75+ years	2007	1758.888456

21	Oceania	75+ years	2008	1810.864124
21	Oceania	75+ years	2009	1858.901322
21	Oceania	75+ years	2010	1905.304871
21	Oceania	75+ years	2011	1925.641852
21	Oceania	75+ years	2012	1972.122457
21	Oceania	75+ years	2013	2023.248923
21	Oceania	75+ years	2014	2076.503337
21	Oceania	75+ years	2015	2131.359485
21	Oceania	75+ years	2016	2147.158159
21	Oceania	75+ years	2017	2205.429179
21	Oceania	75+ years	2018	2269.298519
21	Oceania	75+ years	2019	2339.532738
21	Oceania	75+ years	2020	2407.462068
21	Oceania	75+ years	2021	2428.376887
21	Oceania	75+ years	2022	2021.235615
21	Oceania	75+ years	2023	2093.844264
21	Oceania	75+ years	2024	2181.364092
21	Oceania	75+ years	2025	2275.010134
21	Oceania	75+ years	2026	2368.346579
21	Oceania	75+ years	2027	2457.26758
21	Oceania	75+ years	2028	2555.857775
21	Oceania	75+ years	2029	2672.304824
21	Oceania	75+ years	2030	2797.574186
21	Oceania	75+ years	2031	2924.471851
21	Oceania	75+ years	2032	3048.073507
21	Oceania	75+ years	2033	3179.413257
21	Oceania	75+ years	2034	3327.372392
21	Oceania	75+ years	2035	3485.337189
21	Oceania	75+ years	2036	3648.128309
159	South Asia	15-29 years	1990	15533.16873
159	South Asia	15-29 years	1991	15690.00242
159	South Asia	15-29 years	1992	15965.14386
159	South Asia	15-29 years	1993	16224.84165
159	South Asia	15-29 years	1994	16739.44209
159	South Asia	15-29 years	1995	17119.94407
159	South Asia	15-29 years	1996	17752.34034
159	South Asia	15-29 years	1997	18624.09509
159	South Asia	15-29 years	1998	19761.40005
159	South Asia	15-29 years	1999	21020.49745
159	South Asia	15-29 years	2000	21494.46213
159	South Asia	15-29 years	2001	21384.46929
159	South Asia	15-29 years	2002	21300.40468
159	South Asia	15-29 years	2003	20854.3125
159	South Asia	15-29 years	2004	21497.87656
159	South Asia	15-29 years	2005	21906.45914
159	South Asia	15-29 years	2006	22190.51918
159	South Asia	15-29 years	2007	22208.02785
159	South Asia	15-29 years	2008	22687.38868
159	South Asia	15-29 years	2009	22898.85241
159	South Asia	15-29 years	2010	23797.46166

159	South Asia	15-29 years	2011	23875.16573
159	South Asia	15-29 years	2012	24304.7132
159	South Asia	15-29 years	2013	24476.46338
159	South Asia	15-29 years	2014	23503.07763
159	South Asia	15-29 years	2015	21905.89071
159	South Asia	15-29 years	2016	22048.84282
159	South Asia	15-29 years	2017	21857.80074
159	South Asia	15-29 years	2018	21542.68343
159	South Asia	15-29 years	2019	21272.39693
159	South Asia	15-29 years	2020	21657.93677
159	South Asia	15-29 years	2021	22267.69926
159	South Asia	15-29 years	2022	22372.32158
159	South Asia	15-29 years	2023	22172.24043
159	South Asia	15-29 years	2024	21965.69137
159	South Asia	15-29 years	2025	21730.99637
159	South Asia	15-29 years	2026	21447.9276
159	South Asia	15-29 years	2027	21110.09735
159	South Asia	15-29 years	2028	20721.19076
159	South Asia	15-29 years	2029	20306.16029
159	South Asia	15-29 years	2030	19893.35735
159	South Asia	15-29 years	2031	19503.61841
159	South Asia	15-29 years	2032	19151.5064
159	South Asia	15-29 years	2033	18854.59438
159	South Asia	15-29 years	2034	18598.63275
159	South Asia	15-29 years	2035	18368.34454
159	South Asia	15-29 years	2036	18150.31815
159	South Asia	30-44 years	1990	61504.2146
159	South Asia	30-44 years	1991	62656.94256
159	South Asia	30-44 years	1992	63776.58776
159	South Asia	30-44 years	1993	64747.22191
159	South Asia	30-44 years	1994	66670.74694
159	South Asia	30-44 years	1995	69128.64501
159	South Asia	30-44 years	1996	71096.63109
159	South Asia	30-44 years	1997	73762.51507
159	South Asia	30-44 years	1998	77532.78279
159	South Asia	30-44 years	1999	81788.24246
159	South Asia	30-44 years	2000	83210.76511
159	South Asia	30-44 years	2001	84885.29192
159	South Asia	30-44 years	2002	87431.68127
159	South Asia	30-44 years	2003	88048.64753
159	South Asia	30-44 years	2004	90004.5001
159	South Asia	30-44 years	2005	90929.55376
159	South Asia	30-44 years	2006	95331.76005
159	South Asia	30-44 years	2007	99319.32479
159	South Asia	30-44 years	2008	101728.4237
159	South Asia	30-44 years	2009	102351.9948
159	South Asia	30-44 years	2010	103962.4854
159	South Asia	30-44 years	2011	107296.628
159	South Asia	30-44 years	2012	112005.8236
159	South Asia	30-44 years	2013	114910.3287

159	South Asia	30-44 years	2014	114886.2746
159	South Asia	30-44 years	2015	110807.9918
159	South Asia	30-44 years	2016	115999.7202
159	South Asia	30-44 years	2017	117545.2498
159	South Asia	30-44 years	2018	116726.0356
159	South Asia	30-44 years	2019	115964.3234
159	South Asia	30-44 years	2020	119419.6101
159	South Asia	30-44 years	2021	123128.6572
159	South Asia	30-44 years	2022	127329.7795
159	South Asia	30-44 years	2023	128785.5586
159	South Asia	30-44 years	2024	130191.9616
159	South Asia	30-44 years	2025	131477.7629
159	South Asia	30-44 years	2026	132569.1978
159	South Asia	30-44 years	2027	133409.016
159	South Asia	30-44 years	2028	133962.6023
159	South Asia	30-44 years	2029	134280.1145
159	South Asia	30-44 years	2030	134452.2408
159	South Asia	30-44 years	2031	134572.6685
159	South Asia	30-44 years	2032	134722.7585
159	South Asia	30-44 years	2033	134971.0052
159	South Asia	30-44 years	2034	135319.8106
159	South Asia	30-44 years	2035	135749.1842
159	South Asia	30-44 years	2036	136239.3591
159	South Asia	45-59 years	1990	185714.0361
159	South Asia	45-59 years	1991	188317.4752
159	South Asia	45-59 years	1992	191395.4172
159	South Asia	45-59 years	1993	193947.3634
159	South Asia	45-59 years	1994	196698.9134
159	South Asia	45-59 years	1995	199728.4412
159	South Asia	45-59 years	1996	205673.7408
159	South Asia	45-59 years	1997	212846.5107
159	South Asia	45-59 years	1998	213745.6842
159	South Asia	45-59 years	1999	215918.8692
159	South Asia	45-59 years	2000	219823.2382
159	South Asia	45-59 years	2001	221905.9001
159	South Asia	45-59 years	2002	225194.5537
159	South Asia	45-59 years	2003	227881.1406
159	South Asia	45-59 years	2004	226175.8765
159	South Asia	45-59 years	2005	231732.392
159	South Asia	45-59 years	2006	241526.451
159	South Asia	45-59 years	2007	252554.2316
159	South Asia	45-59 years	2008	263637.6849
159	South Asia	45-59 years	2009	276948.9253
159	South Asia	45-59 years	2010	292790.1702
159	South Asia	45-59 years	2011	307163.495
159	South Asia	45-59 years	2012	325840.6401
159	South Asia	45-59 years	2013	336620.1463
159	South Asia	45-59 years	2014	353361.3483
159	South Asia	45-59 years	2015	367855.6172
159	South Asia	45-59 years	2016	379307.4676

159	South Asia	45-59 years	2017	377326.3737
159	South Asia	45-59 years	2018	388527.6212
159	South Asia	45-59 years	2019	398078.7729
159	South Asia	45-59 years	2020	403199.3257
159	South Asia	45-59 years	2021	406916.9509
159	South Asia	45-59 years	2022	427055.1067
159	South Asia	45-59 years	2023	438796.2225
159	South Asia	45-59 years	2024	450804.3143
159	South Asia	45-59 years	2025	463045.2168
159	South Asia	45-59 years	2026	475544.9109
159	South Asia	45-59 years	2027	488160.2935
159	South Asia	45-59 years	2028	500707.981
159	South Asia	45-59 years	2029	513138.3627
159	South Asia	45-59 years	2030	525461.6503
159	South Asia	45-59 years	2031	537869.3762
159	South Asia	45-59 years	2032	550458.3489
159	South Asia	45-59 years	2033	563166.9919
159	South Asia	45-59 years	2034	575950.2841
159	South Asia	45-59 years	2035	588879.2213
159	South Asia	45-59 years	2036	602079.0836
159	South Asia	60-75 years	1990	280674.8742
159	South Asia	60-75 years	1991	289193.6305
159	South Asia	60-75 years	1992	301261.816
159	South Asia	60-75 years	1993	310521.7666
159	South Asia	60-75 years	1994	324855.2745
159	South Asia	60-75 years	1995	338508.7484
159	South Asia	60-75 years	1996	350402.745
159	South Asia	60-75 years	1997	368214.9446
159	South Asia	60-75 years	1998	384382.6486
159	South Asia	60-75 years	1999	374643.3023
159	South Asia	60-75 years	2000	378412.9173
159	South Asia	60-75 years	2001	393409.3529
159	South Asia	60-75 years	2002	406624.5621
159	South Asia	60-75 years	2003	420258.2088
159	South Asia	60-75 years	2004	421480.3208
159	South Asia	60-75 years	2005	430259.9932
159	South Asia	60-75 years	2006	447508.8751
159	South Asia	60-75 years	2007	476461.8847
159	South Asia	60-75 years	2008	494390.5047
159	South Asia	60-75 years	2009	502342.407
159	South Asia	60-75 years	2010	524523.0785
159	South Asia	60-75 years	2011	558199.3273
159	South Asia	60-75 years	2012	586703.0253
159	South Asia	60-75 years	2013	578689.9761
159	South Asia	60-75 years	2014	579001.0299
159	South Asia	60-75 years	2015	599397.5709
159	South Asia	60-75 years	2016	640795.7891
159	South Asia	60-75 years	2017	667021.2717
159	South Asia	60-75 years	2018	715455.8981
159	South Asia	60-75 years	2019	739826.1061

159	South Asia	60-75 years	2020	751369.4786
159	South Asia	60-75 years	2021	762601.9864
159	South Asia	60-75 years	2022	741052.0615
159	South Asia	60-75 years	2023	759596.2178
159	South Asia	60-75 years	2024	778221.545
159	South Asia	60-75 years	2025	797583.9829
159	South Asia	60-75 years	2026	818353.4995
159	South Asia	60-75 years	2027	841205.7119
159	South Asia	60-75 years	2028	866214.913
159	South Asia	60-75 years	2029	893152.7
159	South Asia	60-75 years	2030	922187.6816
159	South Asia	60-75 years	2031	953653.6409
159	South Asia	60-75 years	2032	987695.5394
159	South Asia	60-75 years	2033	1024595.771
159	South Asia	60-75 years	2034	1064364.639
159	South Asia	60-75 years	2035	1106644.56
159	South Asia	60-75 years	2036	1151401.645
159	South Asia	75+ years	1990	162105.756
159	South Asia	75+ years	1991	166942.7321
159	South Asia	75+ years	1992	176924.2779
159	South Asia	75+ years	1993	181539.4393
159	South Asia	75+ years	1994	189420.8664
159	South Asia	75+ years	1995	206300.3899
159	South Asia	75+ years	1996	210960.3869
159	South Asia	75+ years	1997	211792.8687
159	South Asia	75+ years	1998	217692.7722
159	South Asia	75+ years	1999	216475.0425
159	South Asia	75+ years	2000	226197.3333
159	South Asia	75+ years	2001	239873.1337
159	South Asia	75+ years	2002	251718.9666
159	South Asia	75+ years	2003	270013.5637
159	South Asia	75+ years	2004	283598.8952
159	South Asia	75+ years	2005	297637.8311
159	South Asia	75+ years	2006	323279.0406
159	South Asia	75+ years	2007	349275.6525
159	South Asia	75+ years	2008	371302.5882
159	South Asia	75+ years	2009	381463.3197
159	South Asia	75+ years	2010	401718.4766
159	South Asia	75+ years	2011	428312.2568
159	South Asia	75+ years	2012	459989.2909
159	South Asia	75+ years	2013	528035.1954
159	South Asia	75+ years	2014	597182.4714
159	South Asia	75+ years	2015	571448.0462
159	South Asia	75+ years	2016	573966.3923
159	South Asia	75+ years	2017	577807.1661
159	South Asia	75+ years	2018	600159.2731
159	South Asia	75+ years	2019	628447.0393
159	South Asia	75+ years	2020	645677.4329
159	South Asia	75+ years	2021	657574.5467
159	South Asia	75+ years	2022	653994.4652

159	South Asia	75+ years	2023	676501.812
159	South Asia	75+ years	2024	702936.9863
159	South Asia	75+ years	2025	731519.9874
159	South Asia	75+ years	2026	760604.6121
159	South Asia	75+ years	2027	788793.6561
159	South Asia	75+ years	2028	818220.0262
159	South Asia	75+ years	2029	851104.124
159	South Asia	75+ years	2030	886477.2694
159	South Asia	75+ years	2031	923616.4361
159	South Asia	75+ years	2032	961562.6453
159	South Asia	75+ years	2033	1001212.338
159	South Asia	75+ years	2034	1044355.97
159	South Asia	75+ years	2035	1091026.431
159	South Asia	75+ years	2036	1141293.32
9	Southeast Asia	15-29 years	1990	6492.045139
9	Southeast Asia	15-29 years	1991	6578.004629
9	Southeast Asia	15-29 years	1992	6677.498977
9	Southeast Asia	15-29 years	1993	6794.087629
9	Southeast Asia	15-29 years	1994	6898.965661
9	Southeast Asia	15-29 years	1995	7010.161425
9	Southeast Asia	15-29 years	1996	7098.088451
9	Southeast Asia	15-29 years	1997	7114.23778
9	Southeast Asia	15-29 years	1998	7134.728711
9	Southeast Asia	15-29 years	1999	7221.294501
9	Southeast Asia	15-29 years	2000	7295.755105
9	Southeast Asia	15-29 years	2001	7331.613916
9	Southeast Asia	15-29 years	2002	7407.364526
9	Southeast Asia	15-29 years	2003	7480.02921
9	Southeast Asia	15-29 years	2004	7544.442035
9	Southeast Asia	15-29 years	2005	7604.449241
9	Southeast Asia	15-29 years	2006	7642.059326
9	Southeast Asia	15-29 years	2007	7662.036148
9	Southeast Asia	15-29 years	2008	7731.164721
9	Southeast Asia	15-29 years	2009	7792.802269
9	Southeast Asia	15-29 years	2010	7774.505793
9	Southeast Asia	15-29 years	2011	7697.023991
9	Southeast Asia	15-29 years	2012	7703.601469
9	Southeast Asia	15-29 years	2013	7724.750062
9	Southeast Asia	15-29 years	2014	7760.249486
9	Southeast Asia	15-29 years	2015	7873.378961
9	Southeast Asia	15-29 years	2016	7933.147131
9	Southeast Asia	15-29 years	2017	7915.202529
9	Southeast Asia	15-29 years	2018	7919.778089
9	Southeast Asia	15-29 years	2019	7951.935662
9	Southeast Asia	15-29 years	2020	7906.76344
9	Southeast Asia	15-29 years	2021	8027.659098
9	Southeast Asia	15-29 years	2022	7934.239582
9	Southeast Asia	15-29 years	2023	8001.15587
9	Southeast Asia	15-29 years	2024	8065.351677
9	Southeast Asia	15-29 years	2025	8119.121128

9	Southeast Asia	15-29 years	2026	8167.157534
9	Southeast Asia	15-29 years	2027	8213.339377
9	Southeast Asia	15-29 years	2028	8259.758528
9	Southeast Asia	15-29 years	2029	8309.327557
9	Southeast Asia	15-29 years	2030	8359.899928
9	Southeast Asia	15-29 years	2031	8408.742814
9	Southeast Asia	15-29 years	2032	8452.811166
9	Southeast Asia	15-29 years	2033	8496.309149
9	Southeast Asia	15-29 years	2034	8541.925318
9	Southeast Asia	15-29 years	2035	8587.524382
9	Southeast Asia	15-29 years	2036	8631.672932
9	Southeast Asia	30-44 years	1990	20741.0861
9	Southeast Asia	30-44 years	1991	21448.55615
9	Southeast Asia	30-44 years	1992	22331.85405
9	Southeast Asia	30-44 years	1993	23238.89258
9	Southeast Asia	30-44 years	1994	24092.03641
9	Southeast Asia	30-44 years	1995	24984.24882
9	Southeast Asia	30-44 years	1996	25778.48115
9	Southeast Asia	30-44 years	1997	26337.77167
9	Southeast Asia	30-44 years	1998	27084.87482
9	Southeast Asia	30-44 years	1999	28078.67288
9	Southeast Asia	30-44 years	2000	28839.45943
9	Southeast Asia	30-44 years	2001	29462.56214
9	Southeast Asia	30-44 years	2002	30328.43975
9	Southeast Asia	30-44 years	2003	31089.06274
9	Southeast Asia	30-44 years	2004	31706.82269
9	Southeast Asia	30-44 years	2005	32188.22944
9	Southeast Asia	30-44 years	2006	32469.50079
9	Southeast Asia	30-44 years	2007	32805.05362
9	Southeast Asia	30-44 years	2008	33447.77133
9	Southeast Asia	30-44 years	2009	34012.65825
9	Southeast Asia	30-44 years	2010	34379.47936
9	Southeast Asia	30-44 years	2011	34425.01167
9	Southeast Asia	30-44 years	2012	35009.35094
9	Southeast Asia	30-44 years	2013	35461.07155
9	Southeast Asia	30-44 years	2014	35785.00179
9	Southeast Asia	30-44 years	2015	36586.39055
9	Southeast Asia	30-44 years	2016	36886.84613
9	Southeast Asia	30-44 years	2017	36693.59297
9	Southeast Asia	30-44 years	2018	36776.51715
9	Southeast Asia	30-44 years	2019	37035.76067
9	Southeast Asia	30-44 years	2020	36863.09823
9	Southeast Asia	30-44 years	2021	37278.01666
9	Southeast Asia	30-44 years	2022	36922.43647
9	Southeast Asia	30-44 years	2023	37030.9423
9	Southeast Asia	30-44 years	2024	37087.69749
9	Southeast Asia	30-44 years	2025	37118.67848
9	Southeast Asia	30-44 years	2026	37171.18109
9	Southeast Asia	30-44 years	2027	37279.83478
9	Southeast Asia	30-44 years	2028	37448.19838

9	Southeast Asia	30-44 years	2029	37664.35332
9	Southeast Asia	30-44 years	2030	37925.94446
9	Southeast Asia	30-44 years	2031	38230.41907
9	Southeast Asia	30-44 years	2032	38565.31251
9	Southeast Asia	30-44 years	2033	38912.16094
9	Southeast Asia	30-44 years	2034	39273.81892
9	Southeast Asia	30-44 years	2035	39666.9168
9	Southeast Asia	30-44 years	2036	40108.69517
9	Southeast Asia	45-59 years	1990	53537.3427
9	Southeast Asia	45-59 years	1991	54343.81683
9	Southeast Asia	45-59 years	1992	55376.31968
9	Southeast Asia	45-59 years	1993	56446.59881
9	Southeast Asia	45-59 years	1994	57635.98675
9	Southeast Asia	45-59 years	1995	58887.89786
9	Southeast Asia	45-59 years	1996	59955.59076
9	Southeast Asia	45-59 years	1997	61012.27239
9	Southeast Asia	45-59 years	1998	62633.89641
9	Southeast Asia	45-59 years	1999	64700.06481
9	Southeast Asia	45-59 years	2000	66505.81371
9	Southeast Asia	45-59 years	2001	68294.43402
9	Southeast Asia	45-59 years	2002	70992.99657
9	Southeast Asia	45-59 years	2003	73965.58663
9	Southeast Asia	45-59 years	2004	77235.07218
9	Southeast Asia	45-59 years	2005	80660.50106
9	Southeast Asia	45-59 years	2006	83323.63188
9	Southeast Asia	45-59 years	2007	86342.08144
9	Southeast Asia	45-59 years	2008	90180.27337
9	Southeast Asia	45-59 years	2009	93892.07189
9	Southeast Asia	45-59 years	2010	97217.85062
9	Southeast Asia	45-59 years	2011	99140.58066
9	Southeast Asia	45-59 years	2012	102708.052
9	Southeast Asia	45-59 years	2013	106335.0547
9	Southeast Asia	45-59 years	2014	109255.6318
9	Southeast Asia	45-59 years	2015	113449.9568
9	Southeast Asia	45-59 years	2016	116586.7893
9	Southeast Asia	45-59 years	2017	118007.9751
9	Southeast Asia	45-59 years	2018	120426.6133
9	Southeast Asia	45-59 years	2019	122757.4504
9	Southeast Asia	45-59 years	2020	124224.3484
9	Southeast Asia	45-59 years	2021	126053.9678
9	Southeast Asia	45-59 years	2022	126140.5227
9	Southeast Asia	45-59 years	2023	128222.1025
9	Southeast Asia	45-59 years	2024	130216.4701
9	Southeast Asia	45-59 years	2025	132070.9647
9	Southeast Asia	45-59 years	2026	133759.9045
9	Southeast Asia	45-59 years	2027	135309.5207
9	Southeast Asia	45-59 years	2028	136726.6321
9	Southeast Asia	45-59 years	2029	138015.8819
9	Southeast Asia	45-59 years	2030	139203.7633
9	Southeast Asia	45-59 years	2031	140320.5414

9	Southeast Asia	45-59 years	2032	141440.2171
9	Southeast Asia	45-59 years	2033	142648.2665
9	Southeast Asia	45-59 years	2034	143931.1567
9	Southeast Asia	45-59 years	2035	145221.2088
9	Southeast Asia	45-59 years	2036	146447.9198
9	Southeast Asia	60-75 years	1990	91081.20267
9	Southeast Asia	60-75 years	1991	93834.67612
9	Southeast Asia	60-75 years	1992	97647.80825
9	Southeast Asia	60-75 years	1993	101345.5998
9	Southeast Asia	60-75 years	1994	104865.0296
9	Southeast Asia	60-75 years	1995	108001.4079
9	Southeast Asia	60-75 years	1996	111430.5847
9	Southeast Asia	60-75 years	1997	114309.8393
9	Southeast Asia	60-75 years	1998	117422.3884
9	Southeast Asia	60-75 years	1999	121433.1664
9	Southeast Asia	60-75 years	2000	124224.3245
9	Southeast Asia	60-75 years	2001	126890.7872
9	Southeast Asia	60-75 years	2002	130134.7639
9	Southeast Asia	60-75 years	2003	133136.5429
9	Southeast Asia	60-75 years	2004	135927.7741
9	Southeast Asia	60-75 years	2005	138928.6349
9	Southeast Asia	60-75 years	2006	141980.2364
9	Southeast Asia	60-75 years	2007	143914.8173
9	Southeast Asia	60-75 years	2008	147758.1096
9	Southeast Asia	60-75 years	2009	151791.1181
9	Southeast Asia	60-75 years	2010	155252.167
9	Southeast Asia	60-75 years	2011	156969.1034
9	Southeast Asia	60-75 years	2012	161438.9682
9	Southeast Asia	60-75 years	2013	166622.1162
9	Southeast Asia	60-75 years	2014	171627.164
9	Southeast Asia	60-75 years	2015	179512.0786
9	Southeast Asia	60-75 years	2016	187024.3642
9	Southeast Asia	60-75 years	2017	193201.0918
9	Southeast Asia	60-75 years	2018	202177.4083
9	Southeast Asia	60-75 years	2019	212014.4145
9	Southeast Asia	60-75 years	2020	219742.0921
9	Southeast Asia	60-75 years	2021	230605.9308
9	Southeast Asia	60-75 years	2022	231273.2286
9	Southeast Asia	60-75 years	2023	240332.1573
9	Southeast Asia	60-75 years	2024	249657.3865
9	Southeast Asia	60-75 years	2025	259039.015
9	Southeast Asia	60-75 years	2026	268327.5175
9	Southeast Asia	60-75 years	2027	277439.5626
9	Southeast Asia	60-75 years	2028	286401.7101
9	Southeast Asia	60-75 years	2029	295225.1005
9	Southeast Asia	60-75 years	2030	303857.8764
9	Southeast Asia	60-75 years	2031	312266.3911
9	Southeast Asia	60-75 years	2032	320536.1525
9	Southeast Asia	60-75 years	2033	328581.5281
9	Southeast Asia	60-75 years	2034	336285.1571

9	Southeast Asia	60-75 years	2035	343703.4724
9	Southeast Asia	60-75 years	2036	350927.9407
9	Southeast Asia	75+ years	1990	78198.46366
9	Southeast Asia	75+ years	1991	80682.60743
9	Southeast Asia	75+ years	1992	84161.81666
9	Southeast Asia	75+ years	1993	87422.65325
9	Southeast Asia	75+ years	1994	91212.48396
9	Southeast Asia	75+ years	1995	95137.96686
9	Southeast Asia	75+ years	1996	98672.90407
9	Southeast Asia	75+ years	1997	101860.5977
9	Southeast Asia	75+ years	1998	106109.743
9	Southeast Asia	75+ years	1999	110583.689
9	Southeast Asia	75+ years	2000	114162.81
9	Southeast Asia	75+ years	2001	118197.1155
9	Southeast Asia	75+ years	2002	123701.5958
9	Southeast Asia	75+ years	2003	128936.079
9	Southeast Asia	75+ years	2004	134815.0345
9	Southeast Asia	75+ years	2005	141415.9181
9	Southeast Asia	75+ years	2006	147321.9672
9	Southeast Asia	75+ years	2007	152648.7122
9	Southeast Asia	75+ years	2008	159131.6362
9	Southeast Asia	75+ years	2009	164856.9496
9	Southeast Asia	75+ years	2010	169791.5843
9	Southeast Asia	75+ years	2011	172938.025
9	Southeast Asia	75+ years	2012	178027.4525
9	Southeast Asia	75+ years	2013	183461.2079
9	Southeast Asia	75+ years	2014	187372.8369
9	Southeast Asia	75+ years	2015	193374.6713
9	Southeast Asia	75+ years	2016	199592.0674
9	Southeast Asia	75+ years	2017	202563.6494
9	Southeast Asia	75+ years	2018	208389.3312
9	Southeast Asia	75+ years	2019	214957.0297
9	Southeast Asia	75+ years	2020	218299.0177
9	Southeast Asia	75+ years	2021	226538.5075
9	Southeast Asia	75+ years	2022	244319.6533
9	Southeast Asia	75+ years	2023	252432.8055
9	Southeast Asia	75+ years	2024	261808.3839
9	Southeast Asia	75+ years	2025	272071.1274
9	Southeast Asia	75+ years	2026	282851.8423
9	Southeast Asia	75+ years	2027	294048.1792
9	Southeast Asia	75+ years	2028	306577.3273
9	Southeast Asia	75+ years	2029	320912.8445
9	Southeast Asia	75+ years	2030	336483.7006
9	Southeast Asia	75+ years	2031	352741.0382
9	Southeast Asia	75+ years	2032	369398.6793
9	Southeast Asia	75+ years	2033	387512.4482
9	Southeast Asia	75+ years	2034	407613.4908
9	Southeast Asia	75+ years	2035	428926.845
9	Southeast Asia	75+ years	2036	450763.1189
96	Southern Latin America	15-29 years	1990	248.6604788

96	Southern Latin America	15-29 years	1991	240.2499498
96	Southern Latin America	15-29 years	1992	232.6109655
96	Southern Latin America	15-29 years	1993	224.5800486
96	Southern Latin America	15-29 years	1994	217.2527029
96	Southern Latin America	15-29 years	1995	211.9731216
96	Southern Latin America	15-29 years	1996	207.310163
96	Southern Latin America	15-29 years	1997	202.5124837
96	Southern Latin America	15-29 years	1998	198.9466091
96	Southern Latin America	15-29 years	1999	194.8532311
96	Southern Latin America	15-29 years	2000	189.2914392
96	Southern Latin America	15-29 years	2001	184.6168758
96	Southern Latin America	15-29 years	2002	180.8365111
96	Southern Latin America	15-29 years	2003	177.1834409
96	Southern Latin America	15-29 years	2004	173.3415523
96	Southern Latin America	15-29 years	2005	170.6747919
96	Southern Latin America	15-29 years	2006	169.3973239
96	Southern Latin America	15-29 years	2007	169.0569407
96	Southern Latin America	15-29 years	2008	168.1673383
96	Southern Latin America	15-29 years	2009	167.6975796
96	Southern Latin America	15-29 years	2010	167.5667555
96	Southern Latin America	15-29 years	2011	167.2068566
96	Southern Latin America	15-29 years	2012	166.6991503
96	Southern Latin America	15-29 years	2013	165.2517912
96	Southern Latin America	15-29 years	2014	164.5393998
96	Southern Latin America	15-29 years	2015	164.7777397
96	Southern Latin America	15-29 years	2016	165.4005441
96	Southern Latin America	15-29 years	2017	164.542821
96	Southern Latin America	15-29 years	2018	163.2490526
96	Southern Latin America	15-29 years	2019	160.9285206
96	Southern Latin America	15-29 years	2020	156.2503963
96	Southern Latin America	15-29 years	2021	150.4232534
96	Southern Latin America	15-29 years	2022	141.5467822
96	Southern Latin America	15-29 years	2023	136.1226151
96	Southern Latin America	15-29 years	2024	130.7982909
96	Southern Latin America	15-29 years	2025	125.5858885
96	Southern Latin America	15-29 years	2026	120.4662831
96	Southern Latin America	15-29 years	2027	115.4581577
96	Southern Latin America	15-29 years	2028	110.5683625
96	Southern Latin America	15-29 years	2029	105.8678946
96	Southern Latin America	15-29 years	2030	101.5043308
96	Southern Latin America	15-29 years	2031	97.57589377
96	Southern Latin America	15-29 years	2032	94.14376336
96	Southern Latin America	15-29 years	2033	91.24647784
96	Southern Latin America	15-29 years	2034	88.78633986
96	Southern Latin America	15-29 years	2035	86.66680705
96	Southern Latin America	15-29 years	2036	84.79723739
96	Southern Latin America	30-44 years	1990	1476.362589
96	Southern Latin America	30-44 years	1991	1439.704323
96	Southern Latin America	30-44 years	1992	1420.487654
96	Southern Latin America	30-44 years	1993	1385.524859

96	Southern Latin America	30-44 years	1994	1350.025302
96	Southern Latin America	30-44 years	1995	1306.203994
96	Southern Latin America	30-44 years	1996	1273.598259
96	Southern Latin America	30-44 years	1997	1238.382136
96	Southern Latin America	30-44 years	1998	1222.917231
96	Southern Latin America	30-44 years	1999	1193.235898
96	Southern Latin America	30-44 years	2000	1149.843529
96	Southern Latin America	30-44 years	2001	1110.992297
96	Southern Latin America	30-44 years	2002	1084.676012
96	Southern Latin America	30-44 years	2003	1049.516124
96	Southern Latin America	30-44 years	2004	1019.424238
96	Southern Latin America	30-44 years	2005	1000.33114
96	Southern Latin America	30-44 years	2006	991.8414738
96	Southern Latin America	30-44 years	2007	980.7569133
96	Southern Latin America	30-44 years	2008	967.5247691
96	Southern Latin America	30-44 years	2009	960.6036159
96	Southern Latin America	30-44 years	2010	961.0525414
96	Southern Latin America	30-44 years	2011	958.9309234
96	Southern Latin America	30-44 years	2012	957.0541181
96	Southern Latin America	30-44 years	2013	953.4165234
96	Southern Latin America	30-44 years	2014	945.8520859
96	Southern Latin America	30-44 years	2015	948.5764035
96	Southern Latin America	30-44 years	2016	958.7907134
96	Southern Latin America	30-44 years	2017	955.8046375
96	Southern Latin America	30-44 years	2018	949.0332012
96	Southern Latin America	30-44 years	2019	932.3778891
96	Southern Latin America	30-44 years	2020	901.8692459
96	Southern Latin America	30-44 years	2021	874.5181393
96	Southern Latin America	30-44 years	2022	843.5362383
96	Southern Latin America	30-44 years	2023	816.7266313
96	Southern Latin America	30-44 years	2024	790.1843719
96	Southern Latin America	30-44 years	2025	764.0836428
96	Southern Latin America	30-44 years	2026	738.8627499
96	Southern Latin America	30-44 years	2027	714.9236913
96	Southern Latin America	30-44 years	2028	692.9204352
96	Southern Latin America	30-44 years	2029	672.819228
96	Southern Latin America	30-44 years	2030	653.9615976
96	Southern Latin America	30-44 years	2031	635.8163245
96	Southern Latin America	30-44 years	2032	617.6385973
96	Southern Latin America	30-44 years	2033	598.938707
96	Southern Latin America	30-44 years	2034	580.1143911
96	Southern Latin America	30-44 years	2035	561.6635733
96	Southern Latin America	30-44 years	2036	544.0972366
96	Southern Latin America	45-59 years	1990	7290.874158
96	Southern Latin America	45-59 years	1991	6974.954861
96	Southern Latin America	45-59 years	1992	6874.682976
96	Southern Latin America	45-59 years	1993	6672.430922
96	Southern Latin America	45-59 years	1994	6480.147056
96	Southern Latin America	45-59 years	1995	6397.626663
96	Southern Latin America	45-59 years	1996	6347.75474

96	Southern Latin America	45-59 years	1997	6244.702648
96	Southern Latin America	45-59 years	1998	6207.581616
96	Southern Latin America	45-59 years	1999	6196.000699
96	Southern Latin America	45-59 years	2000	6043.115593
96	Southern Latin America	45-59 years	2001	5970.143199
96	Southern Latin America	45-59 years	2002	5966.902533
96	Southern Latin America	45-59 years	2003	5876.281709
96	Southern Latin America	45-59 years	2004	5736.145417
96	Southern Latin America	45-59 years	2005	5685.909181
96	Southern Latin America	45-59 years	2006	5696.252942
96	Southern Latin America	45-59 years	2007	5749.362021
96	Southern Latin America	45-59 years	2008	5692.128855
96	Southern Latin America	45-59 years	2009	5722.688464
96	Southern Latin America	45-59 years	2010	5697.411677
96	Southern Latin America	45-59 years	2011	5708.482331
96	Southern Latin America	45-59 years	2012	5687.03353
96	Southern Latin America	45-59 years	2013	5641.845411
96	Southern Latin America	45-59 years	2014	5525.960239
96	Southern Latin America	45-59 years	2015	5532.214543
96	Southern Latin America	45-59 years	2016	5544.05744
96	Southern Latin America	45-59 years	2017	5400.45332
96	Southern Latin America	45-59 years	2018	5333.955069
96	Southern Latin America	45-59 years	2019	5234.889473
96	Southern Latin America	45-59 years	2020	5101.032356
96	Southern Latin America	45-59 years	2021	4938.574924
96	Southern Latin America	45-59 years	2022	4739.482477
96	Southern Latin America	45-59 years	2023	4586.51255
96	Southern Latin America	45-59 years	2024	4441.686772
96	Southern Latin America	45-59 years	2025	4306.038698
96	Southern Latin America	45-59 years	2026	4178.217989
96	Southern Latin America	45-59 years	2027	4055.916664
96	Southern Latin America	45-59 years	2028	3940.815232
96	Southern Latin America	45-59 years	2029	3833.497711
96	Southern Latin America	45-59 years	2030	3733.815999
96	Southern Latin America	45-59 years	2031	3641.000318
96	Southern Latin America	45-59 years	2032	3552.221361
96	Southern Latin America	45-59 years	2033	3465.494067
96	Southern Latin America	45-59 years	2034	3381.404488
96	Southern Latin America	45-59 years	2035	3301.478626
96	Southern Latin America	45-59 years	2036	3227.012133
96	Southern Latin America	60-75 years	1990	20346.78069
96	Southern Latin America	60-75 years	1991	19696.56228
96	Southern Latin America	60-75 years	1992	19639.57643
96	Southern Latin America	60-75 years	1993	19437.87079
96	Southern Latin America	60-75 years	1994	18857.09406
96	Southern Latin America	60-75 years	1995	18748.26696
96	Southern Latin America	60-75 years	1996	18414.21954
96	Southern Latin America	60-75 years	1997	17859.90383
96	Southern Latin America	60-75 years	1998	17659.72536
96	Southern Latin America	60-75 years	1999	17432.63003

96	Southern Latin America	60-75 years	2000	16478.44896
96	Southern Latin America	60-75 years	2001	16061.2391
96	Southern Latin America	60-75 years	2002	15910.62515
96	Southern Latin America	60-75 years	2003	15448.85597
96	Southern Latin America	60-75 years	2004	14800.7225
96	Southern Latin America	60-75 years	2005	14549.8816
96	Southern Latin America	60-75 years	2006	14305.82753
96	Southern Latin America	60-75 years	2007	14459.55017
96	Southern Latin America	60-75 years	2008	14121.58169
96	Southern Latin America	60-75 years	2009	14168.56751
96	Southern Latin America	60-75 years	2010	14359.81257
96	Southern Latin America	60-75 years	2011	14502.50557
96	Southern Latin America	60-75 years	2012	14711.43771
96	Southern Latin America	60-75 years	2013	14685.13343
96	Southern Latin America	60-75 years	2014	14561.19234
96	Southern Latin America	60-75 years	2015	14823.58393
96	Southern Latin America	60-75 years	2016	15191.22439
96	Southern Latin America	60-75 years	2017	15073.38863
96	Southern Latin America	60-75 years	2018	15197.27555
96	Southern Latin America	60-75 years	2019	15136.22385
96	Southern Latin America	60-75 years	2020	14955.67064
96	Southern Latin America	60-75 years	2021	14378.23252
96	Southern Latin America	60-75 years	2022	14248.52164
96	Southern Latin America	60-75 years	2023	13887.2907
96	Southern Latin America	60-75 years	2024	13519.10876
96	Southern Latin America	60-75 years	2025	13149.40181
96	Southern Latin America	60-75 years	2026	12782.8429
96	Southern Latin America	60-75 years	2027	12421.73239
96	Southern Latin America	60-75 years	2028	12064.6565
96	Southern Latin America	60-75 years	2029	11713.76747
96	Southern Latin America	60-75 years	2030	11377.6912
96	Southern Latin America	60-75 years	2031	11057.23394
96	Southern Latin America	60-75 years	2032	10752.74968
96	Southern Latin America	60-75 years	2033	10468.0009
96	Southern Latin America	60-75 years	2034	10202.75435
96	Southern Latin America	60-75 years	2035	9962.82981
96	Southern Latin America	60-75 years	2036	9746.613147
96	Southern Latin America	75+ years	1990	31971.52607
96	Southern Latin America	75+ years	1991	30881.42205
96	Southern Latin America	75+ years	1992	30743.61207
96	Southern Latin America	75+ years	1993	30495.54972
96	Southern Latin America	75+ years	1994	29402.76087
96	Southern Latin America	75+ years	1995	29665.33082
96	Southern Latin America	75+ years	1996	29395.53958
96	Southern Latin America	75+ years	1997	29048.90431
96	Southern Latin America	75+ years	1998	29851.33606
96	Southern Latin America	75+ years	1999	30174.71235
96	Southern Latin America	75+ years	2000	28511.49806
96	Southern Latin America	75+ years	2001	28198.5254
96	Southern Latin America	75+ years	2002	28202.69079

96	Southern Latin America	75+ years	2003	28319.15511
96	Southern Latin America	75+ years	2004	27833.86607
96	Southern Latin America	75+ years	2005	27715.62901
96	Southern Latin America	75+ years	2006	28049.98631
96	Southern Latin America	75+ years	2007	28869.28049
96	Southern Latin America	75+ years	2008	28170.69269
96	Southern Latin America	75+ years	2009	28349.81808
96	Southern Latin America	75+ years	2010	29084.54658
96	Southern Latin America	75+ years	2011	29191.98619
96	Southern Latin America	75+ years	2012	28987.28084
96	Southern Latin America	75+ years	2013	28626.34687
96	Southern Latin America	75+ years	2014	28060.70008
96	Southern Latin America	75+ years	2015	28415.23955
96	Southern Latin America	75+ years	2016	29046.2211
96	Southern Latin America	75+ years	2017	28482.11772
96	Southern Latin America	75+ years	2018	28256.13207
96	Southern Latin America	75+ years	2019	28177.8941
96	Southern Latin America	75+ years	2020	27250.27172
96	Southern Latin America	75+ years	2021	26361.09815
96	Southern Latin America	75+ years	2022	26068.05364
96	Southern Latin America	75+ years	2023	25484.03957
96	Southern Latin America	75+ years	2024	24977.24526
96	Southern Latin America	75+ years	2025	24559.72945
96	Southern Latin America	75+ years	2026	24216.07307
96	Southern Latin America	75+ years	2027	23910.20345
96	Southern Latin America	75+ years	2028	23632.69645
96	Southern Latin America	75+ years	2029	23411.40785
96	Southern Latin America	75+ years	2030	23240.14545
96	Southern Latin America	75+ years	2031	23103.55236
96	Southern Latin America	75+ years	2032	22975.16287
96	Southern Latin America	75+ years	2033	22849.84026
96	Southern Latin America	75+ years	2034	22748.45618
96	Southern Latin America	75+ years	2035	22664.08204
96	Southern Latin America	75+ years	2036	22588.18886
192	Southern Sub-Saharan Africa	15-29 years	1990	234.7283375
192	Southern Sub-Saharan Africa	15-29 years	1991	233.9484106
192	Southern Sub-Saharan Africa	15-29 years	1992	243.655866
192	Southern Sub-Saharan Africa	15-29 years	1993	229.2699361
192	Southern Sub-Saharan Africa	15-29 years	1994	231.2890403
192	Southern Sub-Saharan Africa	15-29 years	1995	224.9599483
192	Southern Sub-Saharan Africa	15-29 years	1996	221.0532187
192	Southern Sub-Saharan Africa	15-29 years	1997	232.8897843
192	Southern Sub-Saharan Africa	15-29 years	1998	242.8597731
192	Southern Sub-Saharan Africa	15-29 years	1999	256.1497141
192	Southern Sub-Saharan Africa	15-29 years	2000	283.0016364
192	Southern Sub-Saharan Africa	15-29 years	2001	305.5936618
192	Southern Sub-Saharan Africa	15-29 years	2002	337.2355061
192	Southern Sub-Saharan Africa	15-29 years	2003	362.0910045
192	Southern Sub-Saharan Africa	15-29 years	2004	368.8135106
192	Southern Sub-Saharan Africa	15-29 years	2005	346.6985751

192	Southern Sub-Saharan Africa	15-29 years	2006	327.4033797
192	Southern Sub-Saharan Africa	15-29 years	2007	308.7579553
192	Southern Sub-Saharan Africa	15-29 years	2008	299.4663968
192	Southern Sub-Saharan Africa	15-29 years	2009	289.2490196
192	Southern Sub-Saharan Africa	15-29 years	2010	274.6931521
192	Southern Sub-Saharan Africa	15-29 years	2011	258.1703963
192	Southern Sub-Saharan Africa	15-29 years	2012	246.0969301
192	Southern Sub-Saharan Africa	15-29 years	2013	235.6178326
192	Southern Sub-Saharan Africa	15-29 years	2014	229.7415878
192	Southern Sub-Saharan Africa	15-29 years	2015	226.7104663
192	Southern Sub-Saharan Africa	15-29 years	2016	223.2617435
192	Southern Sub-Saharan Africa	15-29 years	2017	219.2568539
192	Southern Sub-Saharan Africa	15-29 years	2018	218.6642025
192	Southern Sub-Saharan Africa	15-29 years	2019	222.2641879
192	Southern Sub-Saharan Africa	15-29 years	2020	224.6010572
192	Southern Sub-Saharan Africa	15-29 years	2021	229.1381105
192	Southern Sub-Saharan Africa	15-29 years	2022	236.6644219
192	Southern Sub-Saharan Africa	15-29 years	2023	241.7850587
192	Southern Sub-Saharan Africa	15-29 years	2024	247.4836168
192	Southern Sub-Saharan Africa	15-29 years	2025	253.5191707
192	Southern Sub-Saharan Africa	15-29 years	2026	259.9688159
192	Southern Sub-Saharan Africa	15-29 years	2027	267.3272525
192	Southern Sub-Saharan Africa	15-29 years	2028	276.3503136
192	Southern Sub-Saharan Africa	15-29 years	2029	287.5077142
192	Southern Sub-Saharan Africa	15-29 years	2030	300.9539619
192	Southern Sub-Saharan Africa	15-29 years	2031	316.7729665
192	Southern Sub-Saharan Africa	15-29 years	2032	335.0715998
192	Southern Sub-Saharan Africa	15-29 years	2033	356.733539
192	Southern Sub-Saharan Africa	15-29 years	2034	382.5431654
192	Southern Sub-Saharan Africa	15-29 years	2035	413.0109341
192	Southern Sub-Saharan Africa	15-29 years	2036	448.7820004
192	Southern Sub-Saharan Africa	30-44 years	1990	1367.919658
192	Southern Sub-Saharan Africa	30-44 years	1991	1403.795791
192	Southern Sub-Saharan Africa	30-44 years	1992	1501.060904
192	Southern Sub-Saharan Africa	30-44 years	1993	1407.471794
192	Southern Sub-Saharan Africa	30-44 years	1994	1440.077757
192	Southern Sub-Saharan Africa	30-44 years	1995	1429.298955
192	Southern Sub-Saharan Africa	30-44 years	1996	1368.840704
192	Southern Sub-Saharan Africa	30-44 years	1997	1475.434676
192	Southern Sub-Saharan Africa	30-44 years	1998	1536.237749
192	Southern Sub-Saharan Africa	30-44 years	1999	1574.821297
192	Southern Sub-Saharan Africa	30-44 years	2000	1740.79562
192	Southern Sub-Saharan Africa	30-44 years	2001	1828.457415
192	Southern Sub-Saharan Africa	30-44 years	2002	2023.404176
192	Southern Sub-Saharan Africa	30-44 years	2003	2231.94377
192	Southern Sub-Saharan Africa	30-44 years	2004	2351.046289
192	Southern Sub-Saharan Africa	30-44 years	2005	2345.864501
192	Southern Sub-Saharan Africa	30-44 years	2006	2309.072697
192	Southern Sub-Saharan Africa	30-44 years	2007	2208.095754
192	Southern Sub-Saharan Africa	30-44 years	2008	2146.659229

192	Southern Sub-Saharan Africa	30-44 years	2009	2057.442997
192	Southern Sub-Saharan Africa	30-44 years	2010	1931.384575
192	Southern Sub-Saharan Africa	30-44 years	2011	1815.764998
192	Southern Sub-Saharan Africa	30-44 years	2012	1772.523404
192	Southern Sub-Saharan Africa	30-44 years	2013	1740.041337
192	Southern Sub-Saharan Africa	30-44 years	2014	1728.055485
192	Southern Sub-Saharan Africa	30-44 years	2015	1720.966905
192	Southern Sub-Saharan Africa	30-44 years	2016	1698.992624
192	Southern Sub-Saharan Africa	30-44 years	2017	1647.747212
192	Southern Sub-Saharan Africa	30-44 years	2018	1678.939964
192	Southern Sub-Saharan Africa	30-44 years	2019	1785.717814
192	Southern Sub-Saharan Africa	30-44 years	2020	1830.782656
192	Southern Sub-Saharan Africa	30-44 years	2021	1916.680787
192	Southern Sub-Saharan Africa	30-44 years	2022	1787.974315
192	Southern Sub-Saharan Africa	30-44 years	2023	1809.417469
192	Southern Sub-Saharan Africa	30-44 years	2024	1836.846069
192	Southern Sub-Saharan Africa	30-44 years	2025	1867.756665
192	Southern Sub-Saharan Africa	30-44 years	2026	1901.19526
192	Southern Sub-Saharan Africa	30-44 years	2027	1937.13261
192	Southern Sub-Saharan Africa	30-44 years	2028	1975.67516
192	Southern Sub-Saharan Africa	30-44 years	2029	2018.477197
192	Southern Sub-Saharan Africa	30-44 years	2030	2068.077278
192	Southern Sub-Saharan Africa	30-44 years	2031	2127.048228
192	Southern Sub-Saharan Africa	30-44 years	2032	2198.322895
192	Southern Sub-Saharan Africa	30-44 years	2033	2280.635933
192	Southern Sub-Saharan Africa	30-44 years	2034	2373.592224
192	Southern Sub-Saharan Africa	30-44 years	2035	2482.924933
192	Southern Sub-Saharan Africa	30-44 years	2036	2615.597309
192	Southern Sub-Saharan Africa	45-59 years	1990	3411.94921
192	Southern Sub-Saharan Africa	45-59 years	1991	3506.952042
192	Southern Sub-Saharan Africa	45-59 years	1992	3707.211049
192	Southern Sub-Saharan Africa	45-59 years	1993	3669.521071
192	Southern Sub-Saharan Africa	45-59 years	1994	3934.669802
192	Southern Sub-Saharan Africa	45-59 years	1995	4146.446424
192	Southern Sub-Saharan Africa	45-59 years	1996	4521.474204
192	Southern Sub-Saharan Africa	45-59 years	1997	5238.87883
192	Southern Sub-Saharan Africa	45-59 years	1998	5502.943361
192	Southern Sub-Saharan Africa	45-59 years	1999	5460.365794
192	Southern Sub-Saharan Africa	45-59 years	2000	5752.613785
192	Southern Sub-Saharan Africa	45-59 years	2001	5785.80556
192	Southern Sub-Saharan Africa	45-59 years	2002	6075.034421
192	Southern Sub-Saharan Africa	45-59 years	2003	6435.281827
192	Southern Sub-Saharan Africa	45-59 years	2004	6679.400783
192	Southern Sub-Saharan Africa	45-59 years	2005	7022.674955
192	Southern Sub-Saharan Africa	45-59 years	2006	7364.154477
192	Southern Sub-Saharan Africa	45-59 years	2007	7494.495436
192	Southern Sub-Saharan Africa	45-59 years	2008	7683.030019
192	Southern Sub-Saharan Africa	45-59 years	2009	7774.571203
192	Southern Sub-Saharan Africa	45-59 years	2010	7747.237894
192	Southern Sub-Saharan Africa	45-59 years	2011	7614.376731

192	Southern Sub-Saharan Africa	45-59 years	2012	7528.995027
192	Southern Sub-Saharan Africa	45-59 years	2013	7415.307419
192	Southern Sub-Saharan Africa	45-59 years	2014	7452.594992
192	Southern Sub-Saharan Africa	45-59 years	2015	7592.623424
192	Southern Sub-Saharan Africa	45-59 years	2016	7591.99327
192	Southern Sub-Saharan Africa	45-59 years	2017	7325.114654
192	Southern Sub-Saharan Africa	45-59 years	2018	7236.930356
192	Southern Sub-Saharan Africa	45-59 years	2019	7040.988534
192	Southern Sub-Saharan Africa	45-59 years	2020	7280.46196
192	Southern Sub-Saharan Africa	45-59 years	2021	7428.941421
192	Southern Sub-Saharan Africa	45-59 years	2022	7701.130286
192	Southern Sub-Saharan Africa	45-59 years	2023	7890.271394
192	Southern Sub-Saharan Africa	45-59 years	2024	8089.969383
192	Southern Sub-Saharan Africa	45-59 years	2025	8296.153871
192	Southern Sub-Saharan Africa	45-59 years	2026	8507.833812
192	Southern Sub-Saharan Africa	45-59 years	2027	8725.832739
192	Southern Sub-Saharan Africa	45-59 years	2028	8959.076735
192	Southern Sub-Saharan Africa	45-59 years	2029	9211.024946
192	Southern Sub-Saharan Africa	45-59 years	2030	9478.090596
192	Southern Sub-Saharan Africa	45-59 years	2031	9762.615953
192	Southern Sub-Saharan Africa	45-59 years	2032	10071.37206
192	Southern Sub-Saharan Africa	45-59 years	2033	10431.23871
192	Southern Sub-Saharan Africa	45-59 years	2034	10861.16923
192	Southern Sub-Saharan Africa	45-59 years	2035	11362.88992
192	Southern Sub-Saharan Africa	45-59 years	2036	11944.75179
192	Southern Sub-Saharan Africa	60-75 years	1990	5661.476582
192	Southern Sub-Saharan Africa	60-75 years	1991	5896.187025
192	Southern Sub-Saharan Africa	60-75 years	1992	6241.742691
192	Southern Sub-Saharan Africa	60-75 years	1993	6300.320014
192	Southern Sub-Saharan Africa	60-75 years	1994	6774.522043
192	Southern Sub-Saharan Africa	60-75 years	1995	7126.640891
192	Southern Sub-Saharan Africa	60-75 years	1996	7847.876524
192	Southern Sub-Saharan Africa	60-75 years	1997	9028.182646
192	Southern Sub-Saharan Africa	60-75 years	1998	9572.495758
192	Southern Sub-Saharan Africa	60-75 years	1999	9714.096044
192	Southern Sub-Saharan Africa	60-75 years	2000	10295.72992
192	Southern Sub-Saharan Africa	60-75 years	2001	10453.39834
192	Southern Sub-Saharan Africa	60-75 years	2002	10834.00067
192	Southern Sub-Saharan Africa	60-75 years	2003	11161.85346
192	Southern Sub-Saharan Africa	60-75 years	2004	11162.93872
192	Southern Sub-Saharan Africa	60-75 years	2005	11261.90593
192	Southern Sub-Saharan Africa	60-75 years	2006	11553.66659
192	Southern Sub-Saharan Africa	60-75 years	2007	11695.19319
192	Southern Sub-Saharan Africa	60-75 years	2008	11940.43903
192	Southern Sub-Saharan Africa	60-75 years	2009	12229.84042
192	Southern Sub-Saharan Africa	60-75 years	2010	12382.45031
192	Southern Sub-Saharan Africa	60-75 years	2011	12430.59473
192	Southern Sub-Saharan Africa	60-75 years	2012	12386.61555
192	Southern Sub-Saharan Africa	60-75 years	2013	12399.69901
192	Southern Sub-Saharan Africa	60-75 years	2014	12717.04594

192	Southern Sub-Saharan Africa	60-75 years	2015	13100.79783
192	Southern Sub-Saharan Africa	60-75 years	2016	13332.36157
192	Southern Sub-Saharan Africa	60-75 years	2017	13317.73009
192	Southern Sub-Saharan Africa	60-75 years	2018	13494.20571
192	Southern Sub-Saharan Africa	60-75 years	2019	13378.60643
192	Southern Sub-Saharan Africa	60-75 years	2020	13919.52525
192	Southern Sub-Saharan Africa	60-75 years	2021	14094.49795
192	Southern Sub-Saharan Africa	60-75 years	2022	15717.55813
192	Southern Sub-Saharan Africa	60-75 years	2023	16246.9207
192	Southern Sub-Saharan Africa	60-75 years	2024	16780.55967
192	Southern Sub-Saharan Africa	60-75 years	2025	17337.14838
192	Southern Sub-Saharan Africa	60-75 years	2026	17926.08801
192	Southern Sub-Saharan Africa	60-75 years	2027	18564.937
192	Southern Sub-Saharan Africa	60-75 years	2028	19251.22527
192	Southern Sub-Saharan Africa	60-75 years	2029	19999.04915
192	Southern Sub-Saharan Africa	60-75 years	2030	20843.36834
192	Southern Sub-Saharan Africa	60-75 years	2031	21812.08972
192	Southern Sub-Saharan Africa	60-75 years	2032	22941.59474
192	Southern Sub-Saharan Africa	60-75 years	2033	24241.41995
192	Southern Sub-Saharan Africa	60-75 years	2034	25721.10572
192	Southern Sub-Saharan Africa	60-75 years	2035	27420.54018
192	Southern Sub-Saharan Africa	60-75 years	2036	29391.68578
192	Southern Sub-Saharan Africa	75+ years	1990	6991.282501
192	Southern Sub-Saharan Africa	75+ years	1991	7185.350079
192	Southern Sub-Saharan Africa	75+ years	1992	7705.751439
192	Southern Sub-Saharan Africa	75+ years	1993	7879.249617
192	Southern Sub-Saharan Africa	75+ years	1994	8517.693753
192	Southern Sub-Saharan Africa	75+ years	1995	8882.298316
192	Southern Sub-Saharan Africa	75+ years	1996	9858.251021
192	Southern Sub-Saharan Africa	75+ years	1997	11001.20409
192	Southern Sub-Saharan Africa	75+ years	1998	11440.07932
192	Southern Sub-Saharan Africa	75+ years	1999	11552.94223
192	Southern Sub-Saharan Africa	75+ years	2000	12084.6029
192	Southern Sub-Saharan Africa	75+ years	2001	12399.65348
192	Southern Sub-Saharan Africa	75+ years	2002	12864.23033
192	Southern Sub-Saharan Africa	75+ years	2003	13315.80782
192	Southern Sub-Saharan Africa	75+ years	2004	13429.03639
192	Southern Sub-Saharan Africa	75+ years	2005	13684.26914
192	Southern Sub-Saharan Africa	75+ years	2006	14077.40987
192	Southern Sub-Saharan Africa	75+ years	2007	14282.19964
192	Southern Sub-Saharan Africa	75+ years	2008	14475.59007
192	Southern Sub-Saharan Africa	75+ years	2009	14683.49431
192	Southern Sub-Saharan Africa	75+ years	2010	14763.32584
192	Southern Sub-Saharan Africa	75+ years	2011	14791.47826
192	Southern Sub-Saharan Africa	75+ years	2012	14707.91417
192	Southern Sub-Saharan Africa	75+ years	2013	14672.47769
192	Southern Sub-Saharan Africa	75+ years	2014	14963.78256
192	Southern Sub-Saharan Africa	75+ years	2015	15463.40824
192	Southern Sub-Saharan Africa	75+ years	2016	15699.76457
192	Southern Sub-Saharan Africa	75+ years	2017	15671.07267

192	Southern Sub-Saharan Africa	75+ years	2018	15845.19893
192	Southern Sub-Saharan Africa	75+ years	2019	15904.59609
192	Southern Sub-Saharan Africa	75+ years	2020	16255.45801
192	Southern Sub-Saharan Africa	75+ years	2021	15808.45723
192	Southern Sub-Saharan Africa	75+ years	2022	20579.27248
192	Southern Sub-Saharan Africa	75+ years	2023	21476.06331
192	Southern Sub-Saharan Africa	75+ years	2024	22515.29841
192	Southern Sub-Saharan Africa	75+ years	2025	23688.43043
192	Southern Sub-Saharan Africa	75+ years	2026	24973.57682
192	Southern Sub-Saharan Africa	75+ years	2027	26352.70682
192	Southern Sub-Saharan Africa	75+ years	2028	27905.52564
192	Southern Sub-Saharan Africa	75+ years	2029	29716.31499
192	Southern Sub-Saharan Africa	75+ years	2030	31777.06639
192	Southern Sub-Saharan Africa	75+ years	2031	34073.80576
192	Southern Sub-Saharan Africa	75+ years	2032	36608.94747
192	Southern Sub-Saharan Africa	75+ years	2033	39506.82417
192	Southern Sub-Saharan Africa	75+ years	2034	42934.88488
192	Southern Sub-Saharan Africa	75+ years	2035	46973.15311
192	Southern Sub-Saharan Africa	75+ years	2036	51705.38196
134	Tropical Latin America	15-29 years	1990	864.7179247
134	Tropical Latin America	15-29 years	1991	844.2918661
134	Tropical Latin America	15-29 years	1992	827.9632229
134	Tropical Latin America	15-29 years	1993	821.7100796
134	Tropical Latin America	15-29 years	1994	816.5913595
134	Tropical Latin America	15-29 years	1995	810.3028069
134	Tropical Latin America	15-29 years	1996	803.5507072
134	Tropical Latin America	15-29 years	1997	792.4175599
134	Tropical Latin America	15-29 years	1998	786.5411925
134	Tropical Latin America	15-29 years	1999	776.8901082
134	Tropical Latin America	15-29 years	2000	773.013395
134	Tropical Latin America	15-29 years	2001	771.4342726
134	Tropical Latin America	15-29 years	2002	774.8831814
134	Tropical Latin America	15-29 years	2003	781.0762509
134	Tropical Latin America	15-29 years	2004	786.8742244
134	Tropical Latin America	15-29 years	2005	790.7780304
134	Tropical Latin America	15-29 years	2006	802.1457343
134	Tropical Latin America	15-29 years	2007	812.7214126
134	Tropical Latin America	15-29 years	2008	826.5192262
134	Tropical Latin America	15-29 years	2009	839.9882226
134	Tropical Latin America	15-29 years	2010	855.6835496
134	Tropical Latin America	15-29 years	2011	877.7553108
134	Tropical Latin America	15-29 years	2012	895.8126482
134	Tropical Latin America	15-29 years	2013	921.6867945
134	Tropical Latin America	15-29 years	2014	948.9955361
134	Tropical Latin America	15-29 years	2015	968.7547394
134	Tropical Latin America	15-29 years	2016	992.7871745
134	Tropical Latin America	15-29 years	2017	995.806176
134	Tropical Latin America	15-29 years	2018	999.6167533
134	Tropical Latin America	15-29 years	2019	1019.193643
134	Tropical Latin America	15-29 years	2020	1041.525318

134	Tropical Latin America	15-29 years	2021	1063.353152
134	Tropical Latin America	15-29 years	2022	1116.123056
134	Tropical Latin America	15-29 years	2023	1137.038127
134	Tropical Latin America	15-29 years	2024	1152.936548
134	Tropical Latin America	15-29 years	2025	1162.157452
134	Tropical Latin America	15-29 years	2026	1165.360078
134	Tropical Latin America	15-29 years	2027	1165.878196
134	Tropical Latin America	15-29 years	2028	1166.123244
134	Tropical Latin America	15-29 years	2029	1167.36134
134	Tropical Latin America	15-29 years	2030	1170.433876
134	Tropical Latin America	15-29 years	2031	1175.838901
134	Tropical Latin America	15-29 years	2032	1183.782036
134	Tropical Latin America	15-29 years	2033	1194.690402
134	Tropical Latin America	15-29 years	2034	1208.163854
134	Tropical Latin America	15-29 years	2035	1223.797269
134	Tropical Latin America	15-29 years	2036	1241.474209
134	Tropical Latin America	30-44 years	1990	6275.225125
134	Tropical Latin America	30-44 years	1991	6231.787066
134	Tropical Latin America	30-44 years	1992	6287.700431
134	Tropical Latin America	30-44 years	1993	6416.471727
134	Tropical Latin America	30-44 years	1994	6468.217024
134	Tropical Latin America	30-44 years	1995	6537.608811
134	Tropical Latin America	30-44 years	1996	6620.496422
134	Tropical Latin America	30-44 years	1997	6617.036496
134	Tropical Latin America	30-44 years	1998	6639.661522
134	Tropical Latin America	30-44 years	1999	6574.413597
134	Tropical Latin America	30-44 years	2000	6485.6452
134	Tropical Latin America	30-44 years	2001	6395.810954
134	Tropical Latin America	30-44 years	2002	6306.114234
134	Tropical Latin America	30-44 years	2003	6256.096637
134	Tropical Latin America	30-44 years	2004	6249.302832
134	Tropical Latin America	30-44 years	2005	6078.510897
134	Tropical Latin America	30-44 years	2006	6098.158905
134	Tropical Latin America	30-44 years	2007	6096.706796
134	Tropical Latin America	30-44 years	2008	6146.839652
134	Tropical Latin America	30-44 years	2009	6120.20483
134	Tropical Latin America	30-44 years	2010	6126.67256
134	Tropical Latin America	30-44 years	2011	6173.520546
134	Tropical Latin America	30-44 years	2012	6156.016923
134	Tropical Latin America	30-44 years	2013	6151.38045
134	Tropical Latin America	30-44 years	2014	6166.814016
134	Tropical Latin America	30-44 years	2015	6185.773401
134	Tropical Latin America	30-44 years	2016	6287.094385
134	Tropical Latin America	30-44 years	2017	6192.111989
134	Tropical Latin America	30-44 years	2018	6148.188099
134	Tropical Latin America	30-44 years	2019	6213.419289
134	Tropical Latin America	30-44 years	2020	6315.788693
134	Tropical Latin America	30-44 years	2021	6525.116142
134	Tropical Latin America	30-44 years	2022	6512.18621
134	Tropical Latin America	30-44 years	2023	6581.970079

134	Tropical Latin America	30-44 years	2024	6648.903622
134	Tropical Latin America	30-44 years	2025	6715.728072
134	Tropical Latin America	30-44 years	2026	6793.054498
134	Tropical Latin America	30-44 years	2027	6890.192887
134	Tropical Latin America	30-44 years	2028	7006.930984
134	Tropical Latin America	30-44 years	2029	7141.656492
134	Tropical Latin America	30-44 years	2030	7298.032793
134	Tropical Latin America	30-44 years	2031	7480.034816
134	Tropical Latin America	30-44 years	2032	7688.808416
134	Tropical Latin America	30-44 years	2033	7922.102769
134	Tropical Latin America	30-44 years	2034	8173.49441
134	Tropical Latin America	30-44 years	2035	8434.758915
134	Tropical Latin America	30-44 years	2036	8698.705679
134	Tropical Latin America	45-59 years	1990	21218.4974
134	Tropical Latin America	45-59 years	1991	20611.58014
134	Tropical Latin America	45-59 years	1992	20746.51392
134	Tropical Latin America	45-59 years	1993	21304.03614
134	Tropical Latin America	45-59 years	1994	21435.23948
134	Tropical Latin America	45-59 years	1995	21767.47847
134	Tropical Latin America	45-59 years	1996	22029.23529
134	Tropical Latin America	45-59 years	1997	22046.96763
134	Tropical Latin America	45-59 years	1998	22607.1568
134	Tropical Latin America	45-59 years	1999	22898.23301
134	Tropical Latin America	45-59 years	2000	23069.40002
134	Tropical Latin America	45-59 years	2001	23333.1019
134	Tropical Latin America	45-59 years	2002	23635.84457
134	Tropical Latin America	45-59 years	2003	24056.20993
134	Tropical Latin America	45-59 years	2004	24703.15644
134	Tropical Latin America	45-59 years	2005	24611.16919
134	Tropical Latin America	45-59 years	2006	25253.77191
134	Tropical Latin America	45-59 years	2007	25690.85257
134	Tropical Latin America	45-59 years	2008	26261.97824
134	Tropical Latin America	45-59 years	2009	26626.11029
134	Tropical Latin America	45-59 years	2010	27171.99601
134	Tropical Latin America	45-59 years	2011	27509.44787
134	Tropical Latin America	45-59 years	2012	27383.22084
134	Tropical Latin America	45-59 years	2013	27560.50482
134	Tropical Latin America	45-59 years	2014	27392.91103
134	Tropical Latin America	45-59 years	2015	27598.25723
134	Tropical Latin America	45-59 years	2016	28250.45518
134	Tropical Latin America	45-59 years	2017	27602.40494
134	Tropical Latin America	45-59 years	2018	27336.69014
134	Tropical Latin America	45-59 years	2019	27430.55622
134	Tropical Latin America	45-59 years	2020	27739.56781
134	Tropical Latin America	45-59 years	2021	28359.06207
134	Tropical Latin America	45-59 years	2022	26973.4723
134	Tropical Latin America	45-59 years	2023	26880.05978
134	Tropical Latin America	45-59 years	2024	26799.48437
134	Tropical Latin America	45-59 years	2025	26747.02788
134	Tropical Latin America	45-59 years	2026	26733.58541

134	Tropical Latin America	45-59 years	2027	26752.19798
134	Tropical Latin America	45-59 years	2028	26850.48821
134	Tropical Latin America	45-59 years	2029	27037.65975
134	Tropical Latin America	45-59 years	2030	27295.44992
134	Tropical Latin America	45-59 years	2031	27596.50563
134	Tropical Latin America	45-59 years	2032	27901.63099
134	Tropical Latin America	45-59 years	2033	28246.17654
134	Tropical Latin America	45-59 years	2034	28664.84703
134	Tropical Latin America	45-59 years	2035	29150.79489
134	Tropical Latin America	45-59 years	2036	29694.50503
134	Tropical Latin America	60-75 years	1990	39421.33457
134	Tropical Latin America	60-75 years	1991	39045.76892
134	Tropical Latin America	60-75 years	1992	39541.3447
134	Tropical Latin America	60-75 years	1993	40803.10818
134	Tropical Latin America	60-75 years	1994	41104.7658
134	Tropical Latin America	60-75 years	1995	41751.48973
134	Tropical Latin America	60-75 years	1996	42566.97223
134	Tropical Latin America	60-75 years	1997	42330.74806
134	Tropical Latin America	60-75 years	1998	42903.939
134	Tropical Latin America	60-75 years	1999	43110.48336
134	Tropical Latin America	60-75 years	2000	43220.11215
134	Tropical Latin America	60-75 years	2001	43305.74533
134	Tropical Latin America	60-75 years	2002	43433.1189
134	Tropical Latin America	60-75 years	2003	44016.13992
134	Tropical Latin America	60-75 years	2004	44481.14297
134	Tropical Latin America	60-75 years	2005	43544.88392
134	Tropical Latin America	60-75 years	2006	43988.83048
134	Tropical Latin America	60-75 years	2007	44676.85806
134	Tropical Latin America	60-75 years	2008	45420.38853
134	Tropical Latin America	60-75 years	2009	46283.94919
134	Tropical Latin America	60-75 years	2010	47196.36669
134	Tropical Latin America	60-75 years	2011	48407.97313
134	Tropical Latin America	60-75 years	2012	48638.91339
134	Tropical Latin America	60-75 years	2013	49354.03657
134	Tropical Latin America	60-75 years	2014	50154.12784
134	Tropical Latin America	60-75 years	2015	51391.64932
134	Tropical Latin America	60-75 years	2016	53847.6784
134	Tropical Latin America	60-75 years	2017	53978.71409
134	Tropical Latin America	60-75 years	2018	54818.37133
134	Tropical Latin America	60-75 years	2019	56170.83887
134	Tropical Latin America	60-75 years	2020	57926.17043
134	Tropical Latin America	60-75 years	2021	58280.13637
134	Tropical Latin America	60-75 years	2022	59726.91464
134	Tropical Latin America	60-75 years	2023	60748.20991
134	Tropical Latin America	60-75 years	2024	61705.95882
134	Tropical Latin America	60-75 years	2025	62574.84409
134	Tropical Latin America	60-75 years	2026	63334.13086
134	Tropical Latin America	60-75 years	2027	63964.95043
134	Tropical Latin America	60-75 years	2028	64504.57458
134	Tropical Latin America	60-75 years	2029	64955.76095

134	Tropical Latin America	60-75 years	2030	65334.04181
134	Tropical Latin America	60-75 years	2031	65644.71264
134	Tropical Latin America	60-75 years	2032	65886.03631
134	Tropical Latin America	60-75 years	2033	66132.13222
134	Tropical Latin America	60-75 years	2034	66410.25027
134	Tropical Latin America	60-75 years	2035	66766.41497
134	Tropical Latin America	60-75 years	2036	67212.65865
134	Tropical Latin America	75+ years	1990	38425.15492
134	Tropical Latin America	75+ years	1991	37912.66543
134	Tropical Latin America	75+ years	1992	38377.90971
134	Tropical Latin America	75+ years	1993	39770.30189
134	Tropical Latin America	75+ years	1994	40078.96244
134	Tropical Latin America	75+ years	1995	40495.16775
134	Tropical Latin America	75+ years	1996	41193.39826
134	Tropical Latin America	75+ years	1997	41232.8272
134	Tropical Latin America	75+ years	1998	41903.80831
134	Tropical Latin America	75+ years	1999	42303.72358
134	Tropical Latin America	75+ years	2000	42417.13595
134	Tropical Latin America	75+ years	2001	43287.83047
134	Tropical Latin America	75+ years	2002	44639.78003
134	Tropical Latin America	75+ years	2003	45966.74519
134	Tropical Latin America	75+ years	2004	47283.29818
134	Tropical Latin America	75+ years	2005	47100.68845
134	Tropical Latin America	75+ years	2006	48537.23654
134	Tropical Latin America	75+ years	2007	49525.38137
134	Tropical Latin America	75+ years	2008	50555.60349
134	Tropical Latin America	75+ years	2009	51874.50825
134	Tropical Latin America	75+ years	2010	53400.30862
134	Tropical Latin America	75+ years	2011	55039.52052
134	Tropical Latin America	75+ years	2012	55948.21704
134	Tropical Latin America	75+ years	2013	57215.80739
134	Tropical Latin America	75+ years	2014	58394.77487
134	Tropical Latin America	75+ years	2015	60121.42813
134	Tropical Latin America	75+ years	2016	62258.19765
134	Tropical Latin America	75+ years	2017	61850.26131
134	Tropical Latin America	75+ years	2018	62306.00603
134	Tropical Latin America	75+ years	2019	63730.48983
134	Tropical Latin America	75+ years	2020	64223.63722
134	Tropical Latin America	75+ years	2021	63392.90902
134	Tropical Latin America	75+ years	2022	67518.28254
134	Tropical Latin America	75+ years	2023	69166.94137
134	Tropical Latin America	75+ years	2024	71063.8894
134	Tropical Latin America	75+ years	2025	73136.96176
134	Tropical Latin America	75+ years	2026	75333.4163
134	Tropical Latin America	75+ years	2027	77609.48409
134	Tropical Latin America	75+ years	2028	80077.57661
134	Tropical Latin America	75+ years	2029	82803.6984
134	Tropical Latin America	75+ years	2030	85709.40152
134	Tropical Latin America	75+ years	2031	88733.18236
134	Tropical Latin America	75+ years	2032	91830.32008

134	Tropical Latin America	75+ years	2033	95104.10677
134	Tropical Latin America	75+ years	2034	98593.66502
134	Tropical Latin America	75+ years	2035	102221.4015
134	Tropical Latin America	75+ years	2036	105923.0477
73	Western Europe	15-29 years	1990	1030.338448
73	Western Europe	15-29 years	1991	997.1373522
73	Western Europe	15-29 years	1992	950.3500282
73	Western Europe	15-29 years	1993	900.5424618
73	Western Europe	15-29 years	1994	848.3238466
73	Western Europe	15-29 years	1995	801.5140107
73	Western Europe	15-29 years	1996	758.4217537
73	Western Europe	15-29 years	1997	716.4580995
73	Western Europe	15-29 years	1998	678.2353946
73	Western Europe	15-29 years	1999	642.5175465
73	Western Europe	15-29 years	2000	608.4581303
73	Western Europe	15-29 years	2001	577.0873101
73	Western Europe	15-29 years	2002	550.4944172
73	Western Europe	15-29 years	2003	527.0983267
73	Western Europe	15-29 years	2004	502.764568
73	Western Europe	15-29 years	2005	479.7861675
73	Western Europe	15-29 years	2006	459.1080673
73	Western Europe	15-29 years	2007	441.7612291
73	Western Europe	15-29 years	2008	422.9118598
73	Western Europe	15-29 years	2009	403.9451781
73	Western Europe	15-29 years	2010	383.489803
73	Western Europe	15-29 years	2011	363.2340002
73	Western Europe	15-29 years	2012	345.9117552
73	Western Europe	15-29 years	2013	331.1067022
73	Western Europe	15-29 years	2014	317.4608654
73	Western Europe	15-29 years	2015	306.5581431
73	Western Europe	15-29 years	2016	295.4310999
73	Western Europe	15-29 years	2017	283.672686
73	Western Europe	15-29 years	2018	271.5163368
73	Western Europe	15-29 years	2019	258.4103076
73	Western Europe	15-29 years	2020	244.736912
73	Western Europe	15-29 years	2021	234.051559
73	Western Europe	15-29 years	2022	223.5879037
73	Western Europe	15-29 years	2023	214.0292117
73	Western Europe	15-29 years	2024	205.0011584
73	Western Europe	15-29 years	2025	196.3699505
73	Western Europe	15-29 years	2026	187.9579756
73	Western Europe	15-29 years	2027	179.6293612
73	Western Europe	15-29 years	2028	171.5491707
73	Western Europe	15-29 years	2029	163.8589163
73	Western Europe	15-29 years	2030	156.6394294
73	Western Europe	15-29 years	2031	149.8844466
73	Western Europe	15-29 years	2032	143.441171
73	Western Europe	15-29 years	2033	137.3963125
73	Western Europe	15-29 years	2034	131.7867629
73	Western Europe	15-29 years	2035	126.5247532

73	Western Europe	15-29 years	2036	121.5043254
73	Western Europe	30-44 years	1990	10642.15348
73	Western Europe	30-44 years	1991	10615.621
73	Western Europe	30-44 years	1992	10328.26177
73	Western Europe	30-44 years	1993	10018.7449
73	Western Europe	30-44 years	1994	9687.735736
73	Western Europe	30-44 years	1995	9362.864143
73	Western Europe	30-44 years	1996	9167.591555
73	Western Europe	30-44 years	1997	8948.648276
73	Western Europe	30-44 years	1998	8781.893337
73	Western Europe	30-44 years	1999	8569.225484
73	Western Europe	30-44 years	2000	8321.613644
73	Western Europe	30-44 years	2001	8130.816742
73	Western Europe	30-44 years	2002	7954.714585
73	Western Europe	30-44 years	2003	7738.351455
73	Western Europe	30-44 years	2004	7430.346146
73	Western Europe	30-44 years	2005	7124.09792
73	Western Europe	30-44 years	2006	6875.734327
73	Western Europe	30-44 years	2007	6596.702652
73	Western Europe	30-44 years	2008	6279.186325
73	Western Europe	30-44 years	2009	5968.650502
73	Western Europe	30-44 years	2010	5621.275826
73	Western Europe	30-44 years	2011	5286.848417
73	Western Europe	30-44 years	2012	5012.963649
73	Western Europe	30-44 years	2013	4758.543596
73	Western Europe	30-44 years	2014	4551.796858
73	Western Europe	30-44 years	2015	4424.350719
73	Western Europe	30-44 years	2016	4255.836077
73	Western Europe	30-44 years	2017	4097.970573
73	Western Europe	30-44 years	2018	3952.02105
73	Western Europe	30-44 years	2019	3795.888308
73	Western Europe	30-44 years	2020	3574.484428
73	Western Europe	30-44 years	2021	3508.060844
73	Western Europe	30-44 years	2022	3518.710466
73	Western Europe	30-44 years	2023	3406.614584
73	Western Europe	30-44 years	2024	3290.22679
73	Western Europe	30-44 years	2025	3168.307509
73	Western Europe	30-44 years	2026	3041.905703
73	Western Europe	30-44 years	2027	2913.73848
73	Western Europe	30-44 years	2028	2787.217843
73	Western Europe	30-44 years	2029	2664.291787
73	Western Europe	30-44 years	2030	2545.853017
73	Western Europe	30-44 years	2031	2432.186254
73	Western Europe	30-44 years	2032	2322.102719
73	Western Europe	30-44 years	2033	2213.710697
73	Western Europe	30-44 years	2034	2108.09609
73	Western Europe	30-44 years	2035	2008.076169
73	Western Europe	30-44 years	2036	1915.914945
73	Western Europe	45-59 years	1990	63787.06079
73	Western Europe	45-59 years	1991	61960.66645

73	Western Europe	45-59 years	1992	59817.9349
73	Western Europe	45-59 years	1993	58460.18991
73	Western Europe	45-59 years	1994	56192.73381
73	Western Europe	45-59 years	1995	54383.53544
73	Western Europe	45-59 years	1996	52678.28363
73	Western Europe	45-59 years	1997	51018.90127
73	Western Europe	45-59 years	1998	49862.34005
73	Western Europe	45-59 years	1999	48392.29875
73	Western Europe	45-59 years	2000	46541.49599
73	Western Europe	45-59 years	2001	45022.67202
73	Western Europe	45-59 years	2002	44251.11399
73	Western Europe	45-59 years	2003	43469.07634
73	Western Europe	45-59 years	2004	41657.69744
73	Western Europe	45-59 years	2005	40927.64479
73	Western Europe	45-59 years	2006	40302.54127
73	Western Europe	45-59 years	2007	39229.01296
73	Western Europe	45-59 years	2008	37781.91446
73	Western Europe	45-59 years	2009	36642.49031
73	Western Europe	45-59 years	2010	35623.42224
73	Western Europe	45-59 years	2011	34574.61296
73	Western Europe	45-59 years	2012	33737.51263
73	Western Europe	45-59 years	2013	33341.28963
73	Western Europe	45-59 years	2014	32505.70566
73	Western Europe	45-59 years	2015	32673.94281
73	Western Europe	45-59 years	2016	32055.77865
73	Western Europe	45-59 years	2017	31364.73772
73	Western Europe	45-59 years	2018	30839.11667
73	Western Europe	45-59 years	2019	29924.13519
73	Western Europe	45-59 years	2020	28095.61133
73	Western Europe	45-59 years	2021	27854.9929
73	Western Europe	45-59 years	2022	27436.93053
73	Western Europe	45-59 years	2023	26551.59062
73	Western Europe	45-59 years	2024	25605.83891
73	Western Europe	45-59 years	2025	24643.31942
73	Western Europe	45-59 years	2026	23710.52798
73	Western Europe	45-59 years	2027	22829.4604
73	Western Europe	45-59 years	2028	21972.30537
73	Western Europe	45-59 years	2029	21123.06556
73	Western Europe	45-59 years	2030	20311.61504
73	Western Europe	45-59 years	2031	19572.74115
73	Western Europe	45-59 years	2032	18918.40238
73	Western Europe	45-59 years	2033	18333.93806
73	Western Europe	45-59 years	2034	17796.16446
73	Western Europe	45-59 years	2035	17292.15614
73	Western Europe	45-59 years	2036	16816.08158
73	Western Europe	60-75 years	1990	238686.2134
73	Western Europe	60-75 years	1991	237161.4573
73	Western Europe	60-75 years	1992	235953.0808
73	Western Europe	60-75 years	1993	238787.6643
73	Western Europe	60-75 years	1994	235486.1348

73	Western Europe	60-75 years	1995	228978.8067
73	Western Europe	60-75 years	1996	219325.5443
73	Western Europe	60-75 years	1997	209155.2985
73	Western Europe	60-75 years	1998	202364.8305
73	Western Europe	60-75 years	1999	194117.7704
73	Western Europe	60-75 years	2000	181841.3626
73	Western Europe	60-75 years	2001	172661.6816
73	Western Europe	60-75 years	2002	165259.4209
73	Western Europe	60-75 years	2003	158085.2818
73	Western Europe	60-75 years	2004	146965.1758
73	Western Europe	60-75 years	2005	138487.7301
73	Western Europe	60-75 years	2006	130386.7336
73	Western Europe	60-75 years	2007	125465.5373
73	Western Europe	60-75 years	2008	120128.377
73	Western Europe	60-75 years	2009	115944.4037
73	Western Europe	60-75 years	2010	111648.3189
73	Western Europe	60-75 years	2011	107612.2697
73	Western Europe	60-75 years	2012	105326.4538
73	Western Europe	60-75 years	2013	103298.3806
73	Western Europe	60-75 years	2014	99767.63903
73	Western Europe	60-75 years	2015	99777.70745
73	Western Europe	60-75 years	2016	98100.92435
73	Western Europe	60-75 years	2017	97539.29291
73	Western Europe	60-75 years	2018	96889.76063
73	Western Europe	60-75 years	2019	95037.47347
73	Western Europe	60-75 years	2020	92476.91302
73	Western Europe	60-75 years	2021	93758.77049
73	Western Europe	60-75 years	2022	94105.29486
73	Western Europe	60-75 years	2023	93494.13553
73	Western Europe	60-75 years	2024	92632.70195
73	Western Europe	60-75 years	2025	91666.42806
73	Western Europe	60-75 years	2026	90715.86311
73	Western Europe	60-75 years	2027	89785.21767
73	Western Europe	60-75 years	2028	88896.94187
73	Western Europe	60-75 years	2029	87996.91562
73	Western Europe	60-75 years	2030	87051.4869
73	Western Europe	60-75 years	2031	86024.38134
73	Western Europe	60-75 years	2032	84772.25931
73	Western Europe	60-75 years	2033	83287.12382
73	Western Europe	60-75 years	2034	81654.7532
73	Western Europe	60-75 years	2035	79931.7442
73	Western Europe	60-75 years	2036	78166.90067
73	Western Europe	75+ years	1990	543172.2152
73	Western Europe	75+ years	1991	540792.3686
73	Western Europe	75+ years	1992	532094.7157
73	Western Europe	75+ years	1993	527332.8629
73	Western Europe	75+ years	1994	515216.8239
73	Western Europe	75+ years	1995	514681.628
73	Western Europe	75+ years	1996	514319.4063
73	Western Europe	75+ years	1997	510531.7857

73	Western Europe	75+ years	1998	513257.8595
73	Western Europe	75+ years	1999	508454.7278
73	Western Europe	75+ years	2000	490777.8561
73	Western Europe	75+ years	2001	482550.2485
73	Western Europe	75+ years	2002	483260.7987
73	Western Europe	75+ years	2003	479434.0353
73	Western Europe	75+ years	2004	457185.8998
73	Western Europe	75+ years	2005	445435.8753
73	Western Europe	75+ years	2006	434416.8438
73	Western Europe	75+ years	2007	430862.0453
73	Western Europe	75+ years	2008	422537.0498
73	Western Europe	75+ years	2009	416461.0075
73	Western Europe	75+ years	2010	407138.1625
73	Western Europe	75+ years	2011	403126.3144
73	Western Europe	75+ years	2012	406146.4653
73	Western Europe	75+ years	2013	403381.7152
73	Western Europe	75+ years	2014	394922.605
73	Western Europe	75+ years	2015	398233.0444
73	Western Europe	75+ years	2016	389406.5434
73	Western Europe	75+ years	2017	381665.0445
73	Western Europe	75+ years	2018	374920.7741
73	Western Europe	75+ years	2019	368645.9903
73	Western Europe	75+ years	2020	374098.599
73	Western Europe	75+ years	2021	365647.3638
73	Western Europe	75+ years	2022	342015.2246
73	Western Europe	75+ years	2023	335385.7224
73	Western Europe	75+ years	2024	329429.148
73	Western Europe	75+ years	2025	324256.6034
73	Western Europe	75+ years	2026	319726.8777
73	Western Europe	75+ years	2027	315431.6552
73	Western Europe	75+ years	2028	311446.0624
73	Western Europe	75+ years	2029	307951.0568
73	Western Europe	75+ years	2030	305238.9889
73	Western Europe	75+ years	2031	303351.5261
73	Western Europe	75+ years	2032	301955.3038
73	Western Europe	75+ years	2033	300885.0905
73	Western Europe	75+ years	2034	300124.1645
73	Western Europe	75+ years	2035	299995.0905
73	Western Europe	75+ years	2036	300572.232
199	Western Sub-Saharan Africa	15-29 years	1990	584.8423148
199	Western Sub-Saharan Africa	15-29 years	1991	593.3067673
199	Western Sub-Saharan Africa	15-29 years	1992	606.6097425
199	Western Sub-Saharan Africa	15-29 years	1993	623.1835056
199	Western Sub-Saharan Africa	15-29 years	1994	643.6904449
199	Western Sub-Saharan Africa	15-29 years	1995	670.7120875
199	Western Sub-Saharan Africa	15-29 years	1996	707.8811637
199	Western Sub-Saharan Africa	15-29 years	1997	751.4643646
199	Western Sub-Saharan Africa	15-29 years	1998	798.2745794
199	Western Sub-Saharan Africa	15-29 years	1999	847.7820948
199	Western Sub-Saharan Africa	15-29 years	2000	895.3910811

199	Western Sub-Saharan Africa	15-29 years	2001	934.1059656
199	Western Sub-Saharan Africa	15-29 years	2002	961.4771491
199	Western Sub-Saharan Africa	15-29 years	2003	978.2194991
199	Western Sub-Saharan Africa	15-29 years	2004	992.0204449
199	Western Sub-Saharan Africa	15-29 years	2005	1006.132286
199	Western Sub-Saharan Africa	15-29 years	2006	1024.929477
199	Western Sub-Saharan Africa	15-29 years	2007	1048.858434
199	Western Sub-Saharan Africa	15-29 years	2008	1084.227535
199	Western Sub-Saharan Africa	15-29 years	2009	1127.834553
199	Western Sub-Saharan Africa	15-29 years	2010	1173.38944
199	Western Sub-Saharan Africa	15-29 years	2011	1223.276103
199	Western Sub-Saharan Africa	15-29 years	2012	1268.432939
199	Western Sub-Saharan Africa	15-29 years	2013	1295.579482
199	Western Sub-Saharan Africa	15-29 years	2014	1345.259648
199	Western Sub-Saharan Africa	15-29 years	2015	1427.201834
199	Western Sub-Saharan Africa	15-29 years	2016	1465.552954
199	Western Sub-Saharan Africa	15-29 years	2017	1496.508971
199	Western Sub-Saharan Africa	15-29 years	2018	1533.619824
199	Western Sub-Saharan Africa	15-29 years	2019	1575.260465
199	Western Sub-Saharan Africa	15-29 years	2020	1619.408115
199	Western Sub-Saharan Africa	15-29 years	2021	1671.916117
199	Western Sub-Saharan Africa	15-29 years	2022	1735.432412
199	Western Sub-Saharan Africa	15-29 years	2023	1785.101354
199	Western Sub-Saharan Africa	15-29 years	2024	1831.508779
199	Western Sub-Saharan Africa	15-29 years	2025	1879.753059
199	Western Sub-Saharan Africa	15-29 years	2026	1931.323481
199	Western Sub-Saharan Africa	15-29 years	2027	1985.477936
199	Western Sub-Saharan Africa	15-29 years	2028	2042.691973
199	Western Sub-Saharan Africa	15-29 years	2029	2103.775736
199	Western Sub-Saharan Africa	15-29 years	2030	2168.66726
199	Western Sub-Saharan Africa	15-29 years	2031	2237.395574
199	Western Sub-Saharan Africa	15-29 years	2032	2312.041017
199	Western Sub-Saharan Africa	15-29 years	2033	2391.832241
199	Western Sub-Saharan Africa	15-29 years	2034	2478.309471
199	Western Sub-Saharan Africa	15-29 years	2035	2573.638996
199	Western Sub-Saharan Africa	15-29 years	2036	2680.277549
199	Western Sub-Saharan Africa	30-44 years	1990	2807.631704
199	Western Sub-Saharan Africa	30-44 years	1991	2863.506504
199	Western Sub-Saharan Africa	30-44 years	1992	2930.309573
199	Western Sub-Saharan Africa	30-44 years	1993	3012.006784
199	Western Sub-Saharan Africa	30-44 years	1994	3115.751715
199	Western Sub-Saharan Africa	30-44 years	1995	3208.104632
199	Western Sub-Saharan Africa	30-44 years	1996	3368.936041
199	Western Sub-Saharan Africa	30-44 years	1997	3555.421487
199	Western Sub-Saharan Africa	30-44 years	1998	3751.216577
199	Western Sub-Saharan Africa	30-44 years	1999	3953.528552
199	Western Sub-Saharan Africa	30-44 years	2000	4137.4785
199	Western Sub-Saharan Africa	30-44 years	2001	4278.835579
199	Western Sub-Saharan Africa	30-44 years	2002	4354.510995
199	Western Sub-Saharan Africa	30-44 years	2003	4387.680053

199	Western Sub-Saharan Africa	30-44 years	2004	4408.251306
199	Western Sub-Saharan Africa	30-44 years	2005	4433.814711
199	Western Sub-Saharan Africa	30-44 years	2006	4482.419716
199	Western Sub-Saharan Africa	30-44 years	2007	4564.724257
199	Western Sub-Saharan Africa	30-44 years	2008	4697.080206
199	Western Sub-Saharan Africa	30-44 years	2009	4874.808297
199	Western Sub-Saharan Africa	30-44 years	2010	5062.115301
199	Western Sub-Saharan Africa	30-44 years	2011	5272.341932
199	Western Sub-Saharan Africa	30-44 years	2012	5473.428578
199	Western Sub-Saharan Africa	30-44 years	2013	5590.155207
199	Western Sub-Saharan Africa	30-44 years	2014	5807.636061
199	Western Sub-Saharan Africa	30-44 years	2015	6168.638538
199	Western Sub-Saharan Africa	30-44 years	2016	6333.620637
199	Western Sub-Saharan Africa	30-44 years	2017	6471.308023
199	Western Sub-Saharan Africa	30-44 years	2018	6631.274388
199	Western Sub-Saharan Africa	30-44 years	2019	6810.39721
199	Western Sub-Saharan Africa	30-44 years	2020	6999.17843
199	Western Sub-Saharan Africa	30-44 years	2021	7228.239027
199	Western Sub-Saharan Africa	30-44 years	2022	7653.205999
199	Western Sub-Saharan Africa	30-44 years	2023	7934.776019
199	Western Sub-Saharan Africa	30-44 years	2024	8226.921321
199	Western Sub-Saharan Africa	30-44 years	2025	8537.407017
199	Western Sub-Saharan Africa	30-44 years	2026	8869.0201
199	Western Sub-Saharan Africa	30-44 years	2027	9223.771162
199	Western Sub-Saharan Africa	30-44 years	2028	9602.407986
199	Western Sub-Saharan Africa	30-44 years	2029	10007.27239
199	Western Sub-Saharan Africa	30-44 years	2030	10443.30399
199	Western Sub-Saharan Africa	30-44 years	2031	10915.83905
199	Western Sub-Saharan Africa	30-44 years	2032	11430.57009
199	Western Sub-Saharan Africa	30-44 years	2033	11996.78596
199	Western Sub-Saharan Africa	30-44 years	2034	12621.79713
199	Western Sub-Saharan Africa	30-44 years	2035	13310.07515
199	Western Sub-Saharan Africa	30-44 years	2036	14067.46955
199	Western Sub-Saharan Africa	45-59 years	1990	11848.50032
199	Western Sub-Saharan Africa	45-59 years	1991	11900.48192
199	Western Sub-Saharan Africa	45-59 years	1992	12072.527
199	Western Sub-Saharan Africa	45-59 years	1993	12268.343
199	Western Sub-Saharan Africa	45-59 years	1994	12515.91821
199	Western Sub-Saharan Africa	45-59 years	1995	12932.52552
199	Western Sub-Saharan Africa	45-59 years	1996	13487.28409
199	Western Sub-Saharan Africa	45-59 years	1997	14125.0706
199	Western Sub-Saharan Africa	45-59 years	1998	14773.05759
199	Western Sub-Saharan Africa	45-59 years	1999	15446.99305
199	Western Sub-Saharan Africa	45-59 years	2000	16055.38683
199	Western Sub-Saharan Africa	45-59 years	2001	16549.86391
199	Western Sub-Saharan Africa	45-59 years	2002	16681.03724
199	Western Sub-Saharan Africa	45-59 years	2003	16678.22473
199	Western Sub-Saharan Africa	45-59 years	2004	16610.37887
199	Western Sub-Saharan Africa	45-59 years	2005	16760.09046
199	Western Sub-Saharan Africa	45-59 years	2006	16925.05295

199	Western Sub-Saharan Africa	45-59 years	2007	17211.6293
199	Western Sub-Saharan Africa	45-59 years	2008	17683.11964
199	Western Sub-Saharan Africa	45-59 years	2009	18382.07018
199	Western Sub-Saharan Africa	45-59 years	2010	18884.00805
199	Western Sub-Saharan Africa	45-59 years	2011	19610.84414
199	Western Sub-Saharan Africa	45-59 years	2012	20332.59534
199	Western Sub-Saharan Africa	45-59 years	2013	20638.43528
199	Western Sub-Saharan Africa	45-59 years	2014	21397.15322
199	Western Sub-Saharan Africa	45-59 years	2015	22772.53591
199	Western Sub-Saharan Africa	45-59 years	2016	23220.62598
199	Western Sub-Saharan Africa	45-59 years	2017	23610.24964
199	Western Sub-Saharan Africa	45-59 years	2018	24109.47498
199	Western Sub-Saharan Africa	45-59 years	2019	24664.0496
199	Western Sub-Saharan Africa	45-59 years	2020	25246.38426
199	Western Sub-Saharan Africa	45-59 years	2021	25991.47292
199	Western Sub-Saharan Africa	45-59 years	2022	27709.60261
199	Western Sub-Saharan Africa	45-59 years	2023	28726.72619
199	Western Sub-Saharan Africa	45-59 years	2024	29819.95276
199	Western Sub-Saharan Africa	45-59 years	2025	30996.25066
199	Western Sub-Saharan Africa	45-59 years	2026	32260.18085
199	Western Sub-Saharan Africa	45-59 years	2027	33616.85633
199	Western Sub-Saharan Africa	45-59 years	2028	35073.94508
199	Western Sub-Saharan Africa	45-59 years	2029	36654.81027
199	Western Sub-Saharan Africa	45-59 years	2030	38383.80701
199	Western Sub-Saharan Africa	45-59 years	2031	40285.38027
199	Western Sub-Saharan Africa	45-59 years	2032	42385.29594
199	Western Sub-Saharan Africa	45-59 years	2033	44704.55175
199	Western Sub-Saharan Africa	45-59 years	2034	47276.83568
199	Western Sub-Saharan Africa	45-59 years	2035	50146.00216
199	Western Sub-Saharan Africa	45-59 years	2036	53362.09906
199	Western Sub-Saharan Africa	60-75 years	1990	31159.84802
199	Western Sub-Saharan Africa	60-75 years	1991	31518.81372
199	Western Sub-Saharan Africa	60-75 years	1992	32002.31439
199	Western Sub-Saharan Africa	60-75 years	1993	32564.68003
199	Western Sub-Saharan Africa	60-75 years	1994	33271.83868
199	Western Sub-Saharan Africa	60-75 years	1995	34189.03062
199	Western Sub-Saharan Africa	60-75 years	1996	35520.07422
199	Western Sub-Saharan Africa	60-75 years	1997	37017.22949
199	Western Sub-Saharan Africa	60-75 years	1998	38523.233
199	Western Sub-Saharan Africa	60-75 years	1999	40177.18248
199	Western Sub-Saharan Africa	60-75 years	2000	41639.30302
199	Western Sub-Saharan Africa	60-75 years	2001	42625.79532
199	Western Sub-Saharan Africa	60-75 years	2002	43023.66277
199	Western Sub-Saharan Africa	60-75 years	2003	43099.9167
199	Western Sub-Saharan Africa	60-75 years	2004	43062.13954
199	Western Sub-Saharan Africa	60-75 years	2005	42739.21223
199	Western Sub-Saharan Africa	60-75 years	2006	42526.62744
199	Western Sub-Saharan Africa	60-75 years	2007	42805.53681
199	Western Sub-Saharan Africa	60-75 years	2008	43408.60968
199	Western Sub-Saharan Africa	60-75 years	2009	44460.94758

199	Western Sub-Saharan Africa	60-75 years	2010	45760.95924
199	Western Sub-Saharan Africa	60-75 years	2011	47161.38904
199	Western Sub-Saharan Africa	60-75 years	2012	48562.22155
199	Western Sub-Saharan Africa	60-75 years	2013	48813.29522
199	Western Sub-Saharan Africa	60-75 years	2014	50252.44824
199	Western Sub-Saharan Africa	60-75 years	2015	53348.59948
199	Western Sub-Saharan Africa	60-75 years	2016	54310.4244
199	Western Sub-Saharan Africa	60-75 years	2017	55018.2115
199	Western Sub-Saharan Africa	60-75 years	2018	56080.37048
199	Western Sub-Saharan Africa	60-75 years	2019	57270.5983
199	Western Sub-Saharan Africa	60-75 years	2020	58954.40843
199	Western Sub-Saharan Africa	60-75 years	2021	60702.63581
199	Western Sub-Saharan Africa	60-75 years	2022	64511.32805
199	Western Sub-Saharan Africa	60-75 years	2023	67195.62942
199	Western Sub-Saharan Africa	60-75 years	2024	70135.36259
199	Western Sub-Saharan Africa	60-75 years	2025	73283.59221
199	Western Sub-Saharan Africa	60-75 years	2026	76610.3023
199	Western Sub-Saharan Africa	60-75 years	2027	80096.8142
199	Western Sub-Saharan Africa	60-75 years	2028	83805.33293
199	Western Sub-Saharan Africa	60-75 years	2029	87833.45276
199	Western Sub-Saharan Africa	60-75 years	2030	92204.55778
199	Western Sub-Saharan Africa	60-75 years	2031	96940.70023
199	Western Sub-Saharan Africa	60-75 years	2032	102080.2452
199	Western Sub-Saharan Africa	60-75 years	2033	107673.1399
199	Western Sub-Saharan Africa	60-75 years	2034	113841.8389
199	Western Sub-Saharan Africa	60-75 years	2035	120732.3622
199	Western Sub-Saharan Africa	60-75 years	2036	128502.8772
199	Western Sub-Saharan Africa	75+ years	1990	28039.06771
199	Western Sub-Saharan Africa	75+ years	1991	28450.85455
199	Western Sub-Saharan Africa	75+ years	1992	29052.00715
199	Western Sub-Saharan Africa	75+ years	1993	29734.34607
199	Western Sub-Saharan Africa	75+ years	1994	30537.06196
199	Western Sub-Saharan Africa	75+ years	1995	31545.77796
199	Western Sub-Saharan Africa	75+ years	1996	33010.36739
199	Western Sub-Saharan Africa	75+ years	1997	34617.48471
199	Western Sub-Saharan Africa	75+ years	1998	36352.75004
199	Western Sub-Saharan Africa	75+ years	1999	38119.04709
199	Western Sub-Saharan Africa	75+ years	2000	40033.59797
199	Western Sub-Saharan Africa	75+ years	2001	40942.74389
199	Western Sub-Saharan Africa	75+ years	2002	42015.14147
199	Western Sub-Saharan Africa	75+ years	2003	42003.30145
199	Western Sub-Saharan Africa	75+ years	2004	42249.63919
199	Western Sub-Saharan Africa	75+ years	2005	42557.25845
199	Western Sub-Saharan Africa	75+ years	2006	43451.96972
199	Western Sub-Saharan Africa	75+ years	2007	43809.83871
199	Western Sub-Saharan Africa	75+ years	2008	45533.79649
199	Western Sub-Saharan Africa	75+ years	2009	47112.18972
199	Western Sub-Saharan Africa	75+ years	2010	48588.15362
199	Western Sub-Saharan Africa	75+ years	2011	50193.08918
199	Western Sub-Saharan Africa	75+ years	2012	51730.17954

199	Western Sub-Saharan Africa	75+ years	2013	52215.99898
199	Western Sub-Saharan Africa	75+ years	2014	53421.43857
199	Western Sub-Saharan Africa	75+ years	2015	57691.46264
199	Western Sub-Saharan Africa	75+ years	2016	57963.55646
199	Western Sub-Saharan Africa	75+ years	2017	59347.33714
199	Western Sub-Saharan Africa	75+ years	2018	61189.62307
199	Western Sub-Saharan Africa	75+ years	2019	63223.73723
199	Western Sub-Saharan Africa	75+ years	2020	63884.98671
199	Western Sub-Saharan Africa	75+ years	2021	63591.24212
199	Western Sub-Saharan Africa	75+ years	2022	70454.27358
199	Western Sub-Saharan Africa	75+ years	2023	72507.41329
199	Western Sub-Saharan Africa	75+ years	2024	74898.87267
199	Western Sub-Saharan Africa	75+ years	2025	77599.26577
199	Western Sub-Saharan Africa	75+ years	2026	80559.3985
199	Western Sub-Saharan Africa	75+ years	2027	83749.79846
199	Western Sub-Saharan Africa	75+ years	2028	87394.616
199	Western Sub-Saharan Africa	75+ years	2029	91701.99845
199	Western Sub-Saharan Africa	75+ years	2030	96647.22616
199	Western Sub-Saharan Africa	75+ years	2031	102219.4484
199	Western Sub-Saharan Africa	75+ years	2032	108419.5866
199	Western Sub-Saharan Africa	75+ years	2033	115572.1156
199	Western Sub-Saharan Africa	75+ years	2034	123982.8159
199	Western Sub-Saharan Africa	75+ years	2035	133647.3397
199	Western Sub-Saharan Africa	75+ years	2036	144602.8862

Table S16. Bayesian APC Projections of Age-Standardized Mortality Rates (ASMR) for Ischemic Heart Disease Across Regions, 2022-2036.			
**location_id**	**location_name**	**year**	**ASR**
32	Central Asia	1990	426.7034293
32	Central Asia	1991	449.2247246
32	Central Asia	1992	478.9513783
32	Central Asia	1993	510.7515907
32	Central Asia	1994	526.5894864
32	Central Asia	1995	537.598409
32	Central Asia	1996	539.1752638
32	Central Asia	1997	527.3877989
32	Central Asia	1998	523.0443581
32	Central Asia	1999	514.8700335
32	Central Asia	2000	515.075704
32	Central Asia	2001	509.9170751
32	Central Asia	2002	512.9469846
32	Central Asia	2003	513.185019
32	Central Asia	2004	500.4399374
32	Central Asia	2005	503.0011678
32	Central Asia	2006	500.4517209
32	Central Asia	2007	497.1020668
32	Central Asia	2008	488.828262
32	Central Asia	2009	462.9016615
32	Central Asia	2010	450.0481794
32	Central Asia	2011	444.2870319
32	Central Asia	2012	443.5515694
32	Central Asia	2013	436.0895405
32	Central Asia	2014	431.4927838
32	Central Asia	2015	426.2456285
32	Central Asia	2016	416.2940697
32	Central Asia	2017	403.37528
32	Central Asia	2018	386.2736081
32	Central Asia	2019	371.3274848
32	Central Asia	2020	368.0905403
32	Central Asia	2021	360.3002392
32	Central Asia	2022	351.6632044
32	Central Asia	2023	345.7259
32	Central Asia	2024	340.069523
32	Central Asia	2025	334.7694909
32	Central Asia	2026	329.7347304
32	Central Asia	2027	325.0395048
32	Central Asia	2028	320.9369461
32	Central Asia	2029	317.5772752
32	Central Asia	2030	315.0296195
32	Central Asia	2031	313.1662839
32	Central Asia	2032	311.9812099
32	Central Asia	2033	311.676531
32	Central Asia	2034	312.4414595
32	Central Asia	2035	314.4699296

32	Central Asia	2036	317.7690463
65	High-income Asia Pacific	1990	89.1600451
65	High-income Asia Pacific	1991	86.36349331
65	High-income Asia Pacific	1992	83.88994429
65	High-income Asia Pacific	1993	80.94832957
65	High-income Asia Pacific	1994	75.74251171
65	High-income Asia Pacific	1995	71.079483
65	High-income Asia Pacific	1996	66.91516248
65	High-income Asia Pacific	1997	63.95116817
65	High-income Asia Pacific	1998	62.59650736
65	High-income Asia Pacific	1999	61.39580096
65	High-income Asia Pacific	2000	57.74247441
65	High-income Asia Pacific	2001	55.27370896
65	High-income Asia Pacific	2002	53.63571841
65	High-income Asia Pacific	2003	52.70337944
65	High-income Asia Pacific	2004	51.25634529
65	High-income Asia Pacific	2005	50.92760943
65	High-income Asia Pacific	2006	49.41735062
65	High-income Asia Pacific	2007	47.79773239
65	High-income Asia Pacific	2008	46.53557486
65	High-income Asia Pacific	2009	45.15453318
65	High-income Asia Pacific	2010	44.10086459
65	High-income Asia Pacific	2011	43.38539955
65	High-income Asia Pacific	2012	42.01114616
65	High-income Asia Pacific	2013	40.40448615
65	High-income Asia Pacific	2014	38.99199852
65	High-income Asia Pacific	2015	37.55540193
65	High-income Asia Pacific	2016	36.71180231
65	High-income Asia Pacific	2017	35.85394409
65	High-income Asia Pacific	2018	35.40214869
65	High-income Asia Pacific	2019	34.65572775
65	High-income Asia Pacific	2020	33.54944783
65	High-income Asia Pacific	2021	33.8073737
65	High-income Asia Pacific	2022	32.16501888
65	High-income Asia Pacific	2023	31.61328851
65	High-income Asia Pacific	2024	31.08355889
65	High-income Asia Pacific	2025	30.57579228
65	High-income Asia Pacific	2026	30.09187887
65	High-income Asia Pacific	2027	29.62574281
65	High-income Asia Pacific	2028	29.17852949
65	High-income Asia Pacific	2029	28.75119286
65	High-income Asia Pacific	2030	28.3459285
65	High-income Asia Pacific	2031	27.96561956
65	High-income Asia Pacific	2032	27.60339779
65	High-income Asia Pacific	2033	27.25648152
65	High-income Asia Pacific	2034	26.92421733
65	High-income Asia Pacific	2035	26.60917
65	High-income Asia Pacific	2036	26.31493905
42	Central Europe	1990	369.4700406
42	Central Europe	1991	371.7421759

42	Central Europe	1992	370.3511775
42	Central Europe	1993	367.2825234
42	Central Europe	1994	364.0046978
42	Central Europe	1995	364.1767368
42	Central Europe	1996	359.2053051
42	Central Europe	1997	356.0992022
42	Central Europe	1998	339.6253252
42	Central Europe	1999	327.5962493
42	Central Europe	2000	309.1429914
42	Central Europe	2001	299.5461594
42	Central Europe	2002	292.8436977
42	Central Europe	2003	287.6954321
42	Central Europe	2004	276.8975283
42	Central Europe	2005	271.087198
42	Central Europe	2006	262.3781427
42	Central Europe	2007	255.7629137
42	Central Europe	2008	249.3345384
42	Central Europe	2009	243.9963276
42	Central Europe	2010	235.2438425
42	Central Europe	2011	226.9475352
42	Central Europe	2012	221.5731408
42	Central Europe	2013	212.5221994
42	Central Europe	2014	208.4062236
42	Central Europe	2015	207.8495994
42	Central Europe	2016	200.7246108
42	Central Europe	2017	199.7380667
42	Central Europe	2018	196.6121504
42	Central Europe	2019	191.273586
42	Central Europe	2020	189.689828
42	Central Europe	2021	185.8498233
42	Central Europe	2022	182.7643372
42	Central Europe	2023	179.4813345
42	Central Europe	2024	176.3833174
42	Central Europe	2025	173.5646942
42	Central Europe	2026	170.8200134
42	Central Europe	2027	168.0309951
42	Central Europe	2028	165.1023825
42	Central Europe	2029	162.4577193
42	Central Europe	2030	160.1998061
42	Central Europe	2031	158.1439418
42	Central Europe	2032	156.114689
42	Central Europe	2033	154.01741
42	Central Europe	2034	152.2030129
42	Central Europe	2035	150.7551622
42	Central Europe	2036	149.5263976
9	Southeast Asia	1990	156.4645974
9	Southeast Asia	1991	156.0353691
9	Southeast Asia	1992	156.9640666
9	Southeast Asia	1993	157.5286976
9	Southeast Asia	1994	158.2741266

9	Southeast Asia	1995	158.834093
9	Southeast Asia	1996	159.0044313
9	Southeast Asia	1997	158.2191825
9	Southeast Asia	1998	158.5002978
9	Southeast Asia	1999	159.4736411
9	Southeast Asia	2000	159.1500561
9	Southeast Asia	2001	158.8011529
9	Southeast Asia	2002	159.7120833
9	Southeast Asia	2003	160.1416957
9	Southeast Asia	2004	160.806623
9	Southeast Asia	2005	161.9384428
9	Southeast Asia	2006	162.0113522
9	Southeast Asia	2007	161.2941539
9	Southeast Asia	2008	162.0020638
9	Southeast Asia	2009	162.1239088
9	Southeast Asia	2010	161.4074162
9	Southeast Asia	2011	158.4212821
9	Southeast Asia	2012	157.6381534
9	Southeast Asia	2013	157.0386539
9	Southeast Asia	2014	155.4279808
9	Southeast Asia	2015	155.7234875
9	Southeast Asia	2016	155.2869594
9	Southeast Asia	2017	152.8348923
9	Southeast Asia	2018	152.2947434
9	Southeast Asia	2019	152.106881
9	Southeast Asia	2020	150.1823249
9	Southeast Asia	2021	151.1178563
9	Southeast Asia	2022	148.849527
9	Southeast Asia	2023	148.2724383
9	Southeast Asia	2024	147.7073339
9	Southeast Asia	2025	147.1045715
9	Southeast Asia	2026	146.4526465
9	Southeast Asia	2027	145.8064429
9	Southeast Asia	2028	145.242658
9	Southeast Asia	2029	144.738972
9	Southeast Asia	2030	144.2439936
9	Southeast Asia	2031	143.7280767
9	Southeast Asia	2032	143.2358625
9	Southeast Asia	2033	142.8364338
9	Southeast Asia	2034	142.5136884
9	Southeast Asia	2035	142.2286803
9	Southeast Asia	2036	141.9574677
124	Central Latin America	1990	169.8178864
124	Central Latin America	1991	169.5518804
124	Central Latin America	1992	168.5875557
124	Central Latin America	1993	167.2965248
124	Central Latin America	1994	166.2466915
124	Central Latin America	1995	164.5044759
124	Central Latin America	1996	162.3545586
124	Central Latin America	1997	156.3603522

124	Central Latin America	1998	150.7027565
124	Central Latin America	1999	145.8936244
124	Central Latin America	2000	141.5030294
124	Central Latin America	2001	138.7820107
124	Central Latin America	2002	137.0125324
124	Central Latin America	2003	136.5877883
124	Central Latin America	2004	132.9036361
124	Central Latin America	2005	131.2242187
124	Central Latin America	2006	129.2999996
124	Central Latin America	2007	126.413679
124	Central Latin America	2008	128.2116397
124	Central Latin America	2009	130.1694954
124	Central Latin America	2010	130.6455329
124	Central Latin America	2011	129.325861
124	Central Latin America	2012	128.9799443
124	Central Latin America	2013	131.3969073
124	Central Latin America	2014	132.0951635
124	Central Latin America	2015	133.0896321
124	Central Latin America	2016	137.2208266
124	Central Latin America	2017	135.9494418
124	Central Latin America	2018	137.5783414
124	Central Latin America	2019	137.770434
124	Central Latin America	2020	137.6647672
124	Central Latin America	2021	139.3112188
124	Central Latin America	2022	139.3068791
124	Central Latin America	2023	139.90256
124	Central Latin America	2024	140.4659246
124	Central Latin America	2025	141.0565647
124	Central Latin America	2026	141.7482601
124	Central Latin America	2027	142.5226539
124	Central Latin America	2028	143.536774
124	Central Latin America	2029	144.6645662
124	Central Latin America	2030	145.9629643
124	Central Latin America	2031	147.4443062
124	Central Latin America	2032	149.0764952
124	Central Latin America	2033	151.0167281
124	Central Latin America	2034	153.174348
124	Central Latin America	2035	155.6366377
124	Central Latin America	2036	158.4168624
199	Western Sub-Saharan Africa	1990	142.7926095
199	Western Sub-Saharan Africa	1991	141.3210472
199	Western Sub-Saharan Africa	1992	140.6401218
199	Western Sub-Saharan Africa	1993	140.2436808
199	Western Sub-Saharan Africa	1994	140.4039881
199	Western Sub-Saharan Africa	1995	141.490992
199	Western Sub-Saharan Africa	1996	144.4068083
199	Western Sub-Saharan Africa	1997	147.8058505
199	Western Sub-Saharan Africa	1998	151.1975368
199	Western Sub-Saharan Africa	1999	154.7148568
199	Western Sub-Saharan Africa	2000	157.9135839

199	Western Sub-Saharan Africa	2001	158.4241417
199	Western Sub-Saharan Africa	2002	157.7017466
199	Western Sub-Saharan Africa	2003	154.3466331
199	Western Sub-Saharan Africa	2004	151.177496
199	Western Sub-Saharan Africa	2005	148.05297
199	Western Sub-Saharan Africa	2006	146.1912181
199	Western Sub-Saharan Africa	2007	144.0332117
199	Western Sub-Saharan Africa	2008	144.4763394
199	Western Sub-Saharan Africa	2009	145.2736594
199	Western Sub-Saharan Africa	2010	145.824379
199	Western Sub-Saharan Africa	2011	146.9568882
199	Western Sub-Saharan Africa	2012	147.4980184
199	Western Sub-Saharan Africa	2013	144.720563
199	Western Sub-Saharan Africa	2014	144.5101302
199	Western Sub-Saharan Africa	2015	150.183473
199	Western Sub-Saharan Africa	2016	147.7415888
199	Western Sub-Saharan Africa	2017	145.918869
199	Western Sub-Saharan Africa	2018	144.9306047
199	Western Sub-Saharan Africa	2019	144.1039648
199	Western Sub-Saharan Africa	2020	143.1716245
199	Western Sub-Saharan Africa	2021	142.9520054
199	Western Sub-Saharan Africa	2022	141.9092208
199	Western Sub-Saharan Africa	2023	141.4643504
199	Western Sub-Saharan Africa	2024	141.0984942
199	Western Sub-Saharan Africa	2025	140.8439629
199	Western Sub-Saharan Africa	2026	140.7148977
199	Western Sub-Saharan Africa	2027	140.7396755
199	Western Sub-Saharan Africa	2028	140.9536243
199	Western Sub-Saharan Africa	2029	141.4089389
199	Western Sub-Saharan Africa	2030	142.1419538
199	Western Sub-Saharan Africa	2031	143.1863233
199	Western Sub-Saharan Africa	2032	144.5837213
199	Western Sub-Saharan Africa	2033	146.3769515
199	Western Sub-Saharan Africa	2034	148.6361762
199	Western Sub-Saharan Africa	2035	151.4263292
199	Western Sub-Saharan Africa	2036	154.8279179
174	Eastern Sub-Saharan Africa	1990	94.69293102
174	Eastern Sub-Saharan Africa	1991	96.18095896
174	Eastern Sub-Saharan Africa	1992	97.73197791
174	Eastern Sub-Saharan Africa	1993	99.3082291
174	Eastern Sub-Saharan Africa	1994	100.6061026
174	Eastern Sub-Saharan Africa	1995	101.9102239
174	Eastern Sub-Saharan Africa	1996	102.8778151
174	Eastern Sub-Saharan Africa	1997	103.7062894
174	Eastern Sub-Saharan Africa	1998	104.3453085
174	Eastern Sub-Saharan Africa	1999	104.3989485
174	Eastern Sub-Saharan Africa	2000	104.2609945
174	Eastern Sub-Saharan Africa	2001	103.5933484
174	Eastern Sub-Saharan Africa	2002	103.1120508
174	Eastern Sub-Saharan Africa	2003	102.7025563

174	Eastern Sub-Saharan Africa	2004	102.495963
174	Eastern Sub-Saharan Africa	2005	102.066112
174	Eastern Sub-Saharan Africa	2006	101.5883638
174	Eastern Sub-Saharan Africa	2007	100.9302043
174	Eastern Sub-Saharan Africa	2008	100.5004567
174	Eastern Sub-Saharan Africa	2009	100.1719057
174	Eastern Sub-Saharan Africa	2010	99.93400036
174	Eastern Sub-Saharan Africa	2011	99.66875704
174	Eastern Sub-Saharan Africa	2012	99.12441027
174	Eastern Sub-Saharan Africa	2013	98.58367156
174	Eastern Sub-Saharan Africa	2014	98.12947846
174	Eastern Sub-Saharan Africa	2015	98.3697118
174	Eastern Sub-Saharan Africa	2016	98.35878268
174	Eastern Sub-Saharan Africa	2017	98.07699459
174	Eastern Sub-Saharan Africa	2018	98.04226262
174	Eastern Sub-Saharan Africa	2019	98.03172521
174	Eastern Sub-Saharan Africa	2020	98.26119354
174	Eastern Sub-Saharan Africa	2021	98.39349292
174	Eastern Sub-Saharan Africa	2022	98.07186723
174	Eastern Sub-Saharan Africa	2023	98.1148876
174	Eastern Sub-Saharan Africa	2024	98.15573746
174	Eastern Sub-Saharan Africa	2025	98.18924445
174	Eastern Sub-Saharan Africa	2026	98.21024183
174	Eastern Sub-Saharan Africa	2027	98.22349205
174	Eastern Sub-Saharan Africa	2028	98.24903845
174	Eastern Sub-Saharan Africa	2029	98.29320653
174	Eastern Sub-Saharan Africa	2030	98.35583997
174	Eastern Sub-Saharan Africa	2031	98.43173963
174	Eastern Sub-Saharan Africa	2032	98.5228587
174	Eastern Sub-Saharan Africa	2033	98.64349654
174	Eastern Sub-Saharan Africa	2034	98.79951138
174	Eastern Sub-Saharan Africa	2035	98.99162611
174	Eastern Sub-Saharan Africa	2036	99.21620373
167	Central Sub-Saharan Africa	1990	184.5315575
167	Central Sub-Saharan Africa	1991	183.7780052
167	Central Sub-Saharan Africa	1992	182.9955664
167	Central Sub-Saharan Africa	1993	182.4518362
167	Central Sub-Saharan Africa	1994	182.633051
167	Central Sub-Saharan Africa	1995	182.9060609
167	Central Sub-Saharan Africa	1996	183.1588615
167	Central Sub-Saharan Africa	1997	181.2524872
167	Central Sub-Saharan Africa	1998	179.86357
167	Central Sub-Saharan Africa	1999	178.099819
167	Central Sub-Saharan Africa	2000	176.287629
167	Central Sub-Saharan Africa	2001	173.9173082
167	Central Sub-Saharan Africa	2002	171.1871292
167	Central Sub-Saharan Africa	2003	169.5549641
167	Central Sub-Saharan Africa	2004	167.6295847
167	Central Sub-Saharan Africa	2005	165.6712395
167	Central Sub-Saharan Africa	2006	164.681453

167	Central Sub-Saharan Africa	2007	164.044509
167	Central Sub-Saharan Africa	2008	164.2168106
167	Central Sub-Saharan Africa	2009	163.3678513
167	Central Sub-Saharan Africa	2010	162.3720459
167	Central Sub-Saharan Africa	2011	161.4263679
167	Central Sub-Saharan Africa	2012	160.2711048
167	Central Sub-Saharan Africa	2013	158.713785
167	Central Sub-Saharan Africa	2014	157.4739921
167	Central Sub-Saharan Africa	2015	157.7916174
167	Central Sub-Saharan Africa	2016	157.3741004
167	Central Sub-Saharan Africa	2017	157.26484
167	Central Sub-Saharan Africa	2018	158.2779876
167	Central Sub-Saharan Africa	2019	159.4650011
167	Central Sub-Saharan Africa	2020	161.0128055
167	Central Sub-Saharan Africa	2021	162.2247396
167	Central Sub-Saharan Africa	2022	163.599094
167	Central Sub-Saharan Africa	2023	164.9690583
167	Central Sub-Saharan Africa	2024	166.3742292
167	Central Sub-Saharan Africa	2025	167.8110335
167	Central Sub-Saharan Africa	2026	169.2644548
167	Central Sub-Saharan Africa	2027	170.7459346
167	Central Sub-Saharan Africa	2028	172.2981698
167	Central Sub-Saharan Africa	2029	173.9283616
167	Central Sub-Saharan Africa	2030	175.6393727
167	Central Sub-Saharan Africa	2031	177.423826
167	Central Sub-Saharan Africa	2032	179.2946122
167	Central Sub-Saharan Africa	2033	181.2874057
167	Central Sub-Saharan Africa	2034	183.4106224
167	Central Sub-Saharan Africa	2035	185.6696733
167	Central Sub-Saharan Africa	2036	188.0618092
134	Tropical Latin America	1990	184.437163
134	Tropical Latin America	1991	174.5599705
134	Tropical Latin America	1992	169.5195458
134	Tropical Latin America	1993	168.2709497
134	Tropical Latin America	1994	163.1176477
134	Tropical Latin America	1995	158.9350372
134	Tropical Latin America	1996	155.4437241
134	Tropical Latin America	1997	149.0565591
134	Tropical Latin America	1998	145.5529639
134	Tropical Latin America	1999	141.0569711
134	Tropical Latin America	2000	136.0776811
134	Tropical Latin America	2001	132.4073762
134	Tropical Latin America	2002	129.4836018
134	Tropical Latin America	2003	127.2484483
134	Tropical Latin America	2004	125.1653096
134	Tropical Latin America	2005	119.3454013
134	Tropical Latin America	2006	117.4725935
134	Tropical Latin America	2007	115.1464937
134	Tropical Latin America	2008	113.0619441
134	Tropical Latin America	2009	111.2418657

134	Tropical Latin America	2010	109.8771224
134	Tropical Latin America	2011	108.6462481
134	Tropical Latin America	2012	105.6319248
134	Tropical Latin America	2013	103.5333266
134	Tropical Latin America	2014	101.2812066
134	Tropical Latin America	2015	100.0674621
134	Tropical Latin America	2016	100.1604794
134	Tropical Latin America	2017	96.10658811
134	Tropical Latin America	2018	93.40838744
134	Tropical Latin America	2019	91.99052839
134	Tropical Latin America	2020	90.62581053
134	Tropical Latin America	2021	88.21290994
134	Tropical Latin America	2022	87.04108271
134	Tropical Latin America	2023	85.60080942
134	Tropical Latin America	2024	84.22151037
134	Tropical Latin America	2025	82.8861422
134	Tropical Latin America	2026	81.60006126
134	Tropical Latin America	2027	80.3657997
134	Tropical Latin America	2028	79.23711486
134	Tropical Latin America	2029	78.21089061
134	Tropical Latin America	2030	77.2788707
134	Tropical Latin America	2031	76.43127094
134	Tropical Latin America	2032	75.65252162
134	Tropical Latin America	2033	74.99186746
134	Tropical Latin America	2034	74.45481065
134	Tropical Latin America	2035	74.04100267
134	Tropical Latin America	2036	73.74026879
100	High-income North America	1990	236.9459735
100	High-income North America	1991	229.3772234
100	High-income North America	1992	222.8418759
100	High-income North America	1993	221.8943683
100	High-income North America	1994	217.4533812
100	High-income North America	1995	213.0914812
100	High-income North America	1996	206.0621291
100	High-income North America	1997	199.03013
100	High-income North America	1998	194.3931435
100	High-income North America	1999	194.2702958
100	High-income North America	2000	186.8482086
100	High-income North America	2001	180.4778156
100	High-income North America	2002	174.6268045
100	High-income North America	2003	166.871329
100	High-income North America	2004	157.3076387
100	High-income North America	2005	151.3696109
100	High-income North America	2006	143.8224537
100	High-income North America	2007	136.13145
100	High-income North America	2008	131.3493563
100	High-income North America	2009	125.5703681
100	High-income North America	2010	119.9969889
100	High-income North America	2011	116.9564949
100	High-income North America	2012	113.9955777

100	High-income North America	2013	111.6559122
100	High-income North America	2014	109.6098986
100	High-income North America	2015	107.7034104
100	High-income North America	2016	106.6373013
100	High-income North America	2017	104.9571585
100	High-income North America	2018	102.3534042
100	High-income North America	2019	100.0242679
100	High-income North America	2020	98.00121784
100	High-income North America	2021	99.4482195
100	High-income North America	2022	92.9475894
100	High-income North America	2023	91.52001142
100	High-income North America	2024	90.21630485
100	High-income North America	2025	89.01879725
100	High-income North America	2026	87.91910411
100	High-income North America	2027	86.89494931
100	High-income North America	2028	85.99126727
100	High-income North America	2029	85.22922232
100	High-income North America	2030	84.60380391
100	High-income North America	2031	84.10847213
100	High-income North America	2032	83.71237477
100	High-income North America	2033	83.43567626
100	High-income North America	2034	83.29436039
100	High-income North America	2035	83.29091842
100	High-income North America	2036	83.42643864
1	Global	1990	212.8923714
1	Global	1991	209.9717291
1	Global	1992	209.0478162
1	Global	1993	211.916527
1	Global	1994	211.1159716
1	Global	1995	208.2267235
1	Global	1996	203.4273831
1	Global	1997	198.7918698
1	Global	1998	195.6436173
1	Global	1999	194.3761464
1	Global	2000	191.6128528
1	Global	2001	189.7278608
1	Global	2002	189.1816877
1	Global	2003	188.7687121
1	Global	2004	184.6294573
1	Global	2005	182.4660048
1	Global	2006	176.5549483
1	Global	2007	173.7982056
1	Global	2008	172.7329689
1	Global	2009	169.8282397
1	Global	2010	168.626666
1	Global	2011	166.4159072
1	Global	2012	164.4353099
1	Global	2013	163.0988274
1	Global	2014	161.1887607
1	Global	2015	158.1345672

1	Global	2016	155.9243435
1	Global	2017	152.6821744
1	Global	2018	151.2748362
1	Global	2019	149.509623
1	Global	2020	147.626402
1	Global	2021	146.6793823
1	Global	2022	144.3201462
1	Global	2023	143.1561377
1	Global	2024	142.030005
1	Global	2025	140.9247167
1	Global	2026	139.8604489
1	Global	2027	138.8913457
1	Global	2028	138.0492924
1	Global	2029	137.2952587
1	Global	2030	136.6044607
1	Global	2031	135.9701234
1	Global	2032	135.4296893
1	Global	2033	135.0133859
1	Global	2034	134.6949917
1	Global	2035	134.4650299
1	Global	2036	134.33817
70	Australasia	1990	235.8004001
70	Australasia	1991	224.8330439
70	Australasia	1992	218.2377377
70	Australasia	1993	209.5175339
70	Australasia	1994	202.6848898
70	Australasia	1995	194.3976381
70	Australasia	1996	186.197951
70	Australasia	1997	176.8304598
70	Australasia	1998	167.9669695
70	Australasia	1999	159.0198262
70	Australasia	2000	149.1846541
70	Australasia	2001	141.3172246
70	Australasia	2002	134.2856465
70	Australasia	2003	127.1344264
70	Australasia	2004	119.9520776
70	Australasia	2005	112.8368311
70	Australasia	2006	106.9458103
70	Australasia	2007	102.9640348
70	Australasia	2008	99.33626795
70	Australasia	2009	94.61936669
70	Australasia	2010	89.80142103
70	Australasia	2011	85.0882029
70	Australasia	2012	80.19902088
70	Australasia	2013	76.55919766
70	Australasia	2014	73.92094184
70	Australasia	2015	71.04984593
70	Australasia	2016	67.59632397
70	Australasia	2017	64.84505243
70	Australasia	2018	62.17719419

70	Australasia	2019	61.31472806
70	Australasia	2020	58.72068974
70	Australasia	2021	59.00595232
70	Australasia	2022	56.89598532
70	Australasia	2023	55.91376702
70	Australasia	2024	55.00016927
70	Australasia	2025	54.1338478
70	Australasia	2026	53.35045711
70	Australasia	2027	52.64423752
70	Australasia	2028	52.03987409
70	Australasia	2029	51.54650176
70	Australasia	2030	51.14452462
70	Australasia	2031	50.84568642
70	Australasia	2032	50.64163828
70	Australasia	2033	50.53600235
70	Australasia	2034	50.5292674
70	Australasia	2035	50.60620867
70	Australasia	2036	50.78116226
21	Oceania	1990	251.2335345
21	Oceania	1991	250.9420098
21	Oceania	1992	250.4764397
21	Oceania	1993	250.0400489
21	Oceania	1994	249.6126738
21	Oceania	1995	249.317734
21	Oceania	1996	249.291441
21	Oceania	1997	249.1649399
21	Oceania	1998	249.0441313
21	Oceania	1999	249.4384702
21	Oceania	2000	249.7450557
21	Oceania	2001	249.6200016
21	Oceania	2002	249.284088
21	Oceania	2003	248.9725555
21	Oceania	2004	248.5535098
21	Oceania	2005	248.809155
21	Oceania	2006	249.3339239
21	Oceania	2007	249.5610938
21	Oceania	2008	249.6085941
21	Oceania	2009	249.28919
21	Oceania	2010	249.1419189
21	Oceania	2011	248.7947774
21	Oceania	2012	247.8294374
21	Oceania	2013	246.5492919
21	Oceania	2014	245.2538142
21	Oceania	2015	243.8822711
21	Oceania	2016	242.2230631
21	Oceania	2017	240.6700168
21	Oceania	2018	239.1427363
21	Oceania	2019	237.8499128
21	Oceania	2020	236.1402684
21	Oceania	2021	234.3589026

21	Oceania	2022	231.6911766
21	Oceania	2023	229.9517514
21	Oceania	2024	228.2466654
21	Oceania	2025	226.5825675
21	Oceania	2026	224.9474301
21	Oceania	2027	223.3521945
21	Oceania	2028	221.8153442
21	Oceania	2029	220.354362
21	Oceania	2030	218.981323
21	Oceania	2031	217.6819518
21	Oceania	2032	216.4730386
21	Oceania	2033	215.3613874
21	Oceania	2034	214.362546
21	Oceania	2035	213.478645
21	Oceania	2036	212.6984545
120	Andean Latin America	1990	124.2433207
120	Andean Latin America	1991	121.3831644
120	Andean Latin America	1992	121.4613393
120	Andean Latin America	1993	119.8988919
120	Andean Latin America	1994	117.0846427
120	Andean Latin America	1995	111.813401
120	Andean Latin America	1996	105.8441466
120	Andean Latin America	1997	103.2462373
120	Andean Latin America	1998	100.662532
120	Andean Latin America	1999	94.86560042
120	Andean Latin America	2000	90.66612933
120	Andean Latin America	2001	87.47188576
120	Andean Latin America	2002	86.2764784
120	Andean Latin America	2003	84.29830293
120	Andean Latin America	2004	81.7167156
120	Andean Latin America	2005	79.95288798
120	Andean Latin America	2006	77.53186642
120	Andean Latin America	2007	75.01497989
120	Andean Latin America	2008	73.09466687
120	Andean Latin America	2009	71.87641655
120	Andean Latin America	2010	70.32370106
120	Andean Latin America	2011	68.68400108
120	Andean Latin America	2012	68.38122297
120	Andean Latin America	2013	68.46933995
120	Andean Latin America	2014	69.14209534
120	Andean Latin America	2015	70.21744966
120	Andean Latin America	2016	73.44318837
120	Andean Latin America	2017	77.77877874
120	Andean Latin America	2018	80.85731321
120	Andean Latin America	2019	82.81333963
120	Andean Latin America	2020	79.52738722
120	Andean Latin America	2021	77.65906713
120	Andean Latin America	2022	75.78176848
120	Andean Latin America	2023	73.93395978
120	Andean Latin America	2024	72.21661607

120	Andean Latin America	2025	70.65995967
120	Andean Latin America	2026	69.30567926
120	Andean Latin America	2027	68.1781927
120	Andean Latin America	2028	67.29569456
120	Andean Latin America	2029	66.67785944
120	Andean Latin America	2030	66.34267742
120	Andean Latin America	2031	66.32767566
120	Andean Latin America	2032	66.67317076
120	Andean Latin America	2033	67.42702452
120	Andean Latin America	2034	68.65275016
120	Andean Latin America	2035	70.42510378
120	Andean Latin America	2036	72.84720216
5	East Asia	1990	124.1612923
5	East Asia	1991	123.6963652
5	East Asia	1992	123.2454417
5	East Asia	1993	123.3246846
5	East Asia	1994	122.814956
5	East Asia	1995	123.0124722
5	East Asia	1996	122.9831983
5	East Asia	1997	123.0720169
5	East Asia	1998	125.1069309
5	East Asia	1999	128.0623114
5	East Asia	2000	134.8261939
5	East Asia	2001	140.6371208
5	East Asia	2002	146.2854296
5	East Asia	2003	157.220741
5	East Asia	2004	164.7459848
5	East Asia	2005	166.2621639
5	East Asia	2006	156.8362084
5	East Asia	2007	155.4064152
5	East Asia	2008	158.4643666
5	East Asia	2009	162.2975514
5	East Asia	2010	166.1235998
5	East Asia	2011	167.7807611
5	East Asia	2012	163.4193686
5	East Asia	2013	164.199042
5	East Asia	2014	160.5631724
5	East Asia	2015	155.0888366
5	East Asia	2016	152.305174
5	East Asia	2017	150.2042982
5	East Asia	2018	147.808869
5	East Asia	2019	145.9144556
5	East Asia	2020	144.8008598
5	East Asia	2021	143.488935
5	East Asia	2022	152.0001454
5	East Asia	2023	151.2108939
5	East Asia	2024	150.4087679
5	East Asia	2025	149.5558254
5	East Asia	2026	148.6616416
5	East Asia	2027	147.7541729

5	East Asia	2028	146.8935192
5	East Asia	2029	146.1164981
5	East Asia	2030	145.431439
5	East Asia	2031	144.8665613
5	East Asia	2032	144.4504686
5	East Asia	2033	144.2196281
5	East Asia	2034	144.2098468
5	East Asia	2035	144.4555994
5	East Asia	2036	144.9937347
192	Southern Sub-Saharan Africa	1990	103.4095699
192	Southern Sub-Saharan Africa	1991	103.1817681
192	Southern Sub-Saharan Africa	1992	106.5849818
192	Southern Sub-Saharan Africa	1993	104.3052677
192	Southern Sub-Saharan Africa	1994	108.750977
192	Southern Sub-Saharan Africa	1995	110.2267536
192	Southern Sub-Saharan Africa	1996	117.4843807
192	Southern Sub-Saharan Africa	1997	129.7783211
192	Southern Sub-Saharan Africa	1998	133.3961458
192	Southern Sub-Saharan Africa	1999	132.1682296
192	Southern Sub-Saharan Africa	2000	136.42243
192	Southern Sub-Saharan Africa	2001	136.4964807
192	Southern Sub-Saharan Africa	2002	139.4344048
192	Southern Sub-Saharan Africa	2003	142.1895922
192	Southern Sub-Saharan Africa	2004	141.0782942
192	Southern Sub-Saharan Africa	2005	140.7665387
192	Southern Sub-Saharan Africa	2006	141.7180874
192	Southern Sub-Saharan Africa	2007	140.7360346
192	Southern Sub-Saharan Africa	2008	140.0020651
192	Southern Sub-Saharan Africa	2009	139.074954
192	Southern Sub-Saharan Africa	2010	136.6718303
192	Southern Sub-Saharan Africa	2011	133.0525778
192	Southern Sub-Saharan Africa	2012	129.1700746
192	Southern Sub-Saharan Africa	2013	125.5694237
192	Southern Sub-Saharan Africa	2014	124.6171513
192	Southern Sub-Saharan Africa	2015	124.8719649
192	Southern Sub-Saharan Africa	2016	123.0108585
192	Southern Sub-Saharan Africa	2017	118.8155
192	Southern Sub-Saharan Africa	2018	116.4468418
192	Southern Sub-Saharan Africa	2019	112.7953511
192	Southern Sub-Saharan Africa	2020	114.0904611
192	Southern Sub-Saharan Africa	2021	113.3025301
192	Southern Sub-Saharan Africa	2022	117.5358701
192	Southern Sub-Saharan Africa	2023	118.305183
192	Southern Sub-Saharan Africa	2024	119.188088
192	Southern Sub-Saharan Africa	2025	120.2409984
192	Southern Sub-Saharan Africa	2026	121.487463
192	Southern Sub-Saharan Africa	2027	122.9795493
192	Southern Sub-Saharan Africa	2028	124.7542086
192	Southern Sub-Saharan Africa	2029	126.8910626
192	Southern Sub-Saharan Africa	2030	129.4734848

192	Southern Sub-Saharan Africa	2031	132.5567373
192	Southern Sub-Saharan Africa	2032	136.249957
192	Southern Sub-Saharan Africa	2033	140.6556813
192	Southern Sub-Saharan Africa	2034	145.9412925
192	Southern Sub-Saharan Africa	2035	152.3119924
192	Southern Sub-Saharan Africa	2036	159.9884788
159	South Asia	1990	187.4060729
159	South Asia	1991	186.7894331
159	South Asia	1992	189.1679494
159	South Asia	1993	188.2983668
159	South Asia	1994	189.9521644
159	South Asia	1995	195.2327546
159	South Asia	1996	195.3286335
159	South Asia	1997	195.0164242
159	South Asia	1998	194.9793783
159	South Asia	1999	187.0826541
159	South Asia	2000	185.0208336
159	South Asia	2001	185.9337148
159	South Asia	2002	185.9259202
159	South Asia	2003	187.4608682
159	South Asia	2004	185.1731123
159	South Asia	2005	184.3481072
159	South Asia	2006	188.6604818
159	South Asia	2007	194.4647158
159	South Asia	2008	196.4976562
159	South Asia	2009	194.0569108
159	South Asia	2010	196.0814382
159	South Asia	2011	199.8460247
159	South Asia	2012	204.6184022
159	South Asia	2013	212.2363585
159	South Asia	2014	221.2412227
159	South Asia	2015	212.5299299
159	South Asia	2016	210.8351715
159	South Asia	2017	205.9435281
159	South Asia	2018	207.1559019
159	South Asia	2019	206.8106045
159	South Asia	2020	205.0248018
159	South Asia	2021	203.5483796
159	South Asia	2022	199.865981
159	South Asia	2023	199.3676104
159	South Asia	2024	198.8970674
159	South Asia	2025	198.4837627
159	South Asia	2026	198.1473071
159	South Asia	2027	197.8840219
159	South Asia	2028	197.711719
159	South Asia	2029	197.654366
159	South Asia	2030	197.7322229
159	South Asia	2031	197.970724
159	South Asia	2032	198.3731448
159	South Asia	2033	198.9518913

159	South Asia	2034	199.7252528
159	South Asia	2035	200.7100794
159	South Asia	2036	201.9378369
56	Eastern Europe	1990	430.4410855
56	Eastern Europe	1991	433.9184546
56	Eastern Europe	1992	457.3186902
56	Eastern Europe	1993	524.0774303
56	Eastern Europe	1994	559.2274372
56	Eastern Europe	1995	547.938511
56	Eastern Europe	1996	523.8125853
56	Eastern Europe	1997	506.3538878
56	Eastern Europe	1998	498.2423231
56	Eastern Europe	1999	528.0247982
56	Eastern Europe	2000	547.2931989
56	Eastern Europe	2001	554.7055381
56	Eastern Europe	2002	571.8709793
56	Eastern Europe	2003	577.8271882
56	Eastern Europe	2004	559.6315216
56	Eastern Europe	2005	567.8273174
56	Eastern Europe	2006	531.8122385
56	Eastern Europe	2007	517.013713
56	Eastern Europe	2008	516.9173572
56	Eastern Europe	2009	488.942504
56	Eastern Europe	2010	487.1456632
56	Eastern Europe	2011	459.2242116
56	Eastern Europe	2012	443.1857068
56	Eastern Europe	2013	428.2077209
56	Eastern Europe	2014	415.1286432
56	Eastern Europe	2015	398.9196826
56	Eastern Europe	2016	385.5875261
56	Eastern Europe	2017	363.9109047
56	Eastern Europe	2018	359.5536225
56	Eastern Europe	2019	351.7674266
56	Eastern Europe	2020	340.552801
56	Eastern Europe	2021	340.9063756
56	Eastern Europe	2022	329.2308981
56	Eastern Europe	2023	329.2968627
56	Eastern Europe	2024	331.1853716
56	Eastern Europe	2025	335.484911
56	Eastern Europe	2026	343.1831102
56	Eastern Europe	2027	355.6331866
56	Eastern Europe	2028	374.396869
56	Eastern Europe	2029	401.1442909
56	Eastern Europe	2030	438.3497246
56	Eastern Europe	2031	490.2036397
56	Eastern Europe	2032	563.4160032
56	Eastern Europe	2033	668.3449767
56	Eastern Europe	2034	821.242178
56	Eastern Europe	2035	1050.058155
56	Eastern Europe	2036	1406.015243

96	Southern Latin America	1990	199.4829819
96	Southern Latin America	1991	187.4597712
96	Southern Latin America	1992	181.2562411
96	Southern Latin America	1993	174.1426412
96	Southern Latin America	1994	163.6183209
96	Southern Latin America	1995	159.1420299
96	Southern Latin America	1996	152.9883089
96	Southern Latin America	1997	146.3368295
96	Southern Latin America	1998	144.4391909
96	Southern Latin America	1999	141.4591969
96	Southern Latin America	2000	131.2791297
96	Southern Latin America	2001	126.2327616
96	Southern Latin America	2002	122.9299382
96	Southern Latin America	2003	119.0285802
96	Southern Latin America	2004	113.259521
96	Southern Latin America	2005	109.4313814
96	Southern Latin America	2006	106.8078249
96	Southern Latin America	2007	106.2918822
96	Southern Latin America	2008	101.467895
96	Southern Latin America	2009	99.51868759
96	Southern Latin America	2010	99.00227915
96	Southern Latin America	2011	97.44366715
96	Southern Latin America	2012	95.43771714
96	Southern Latin America	2013	92.67509206
96	Southern Latin America	2014	89.27601844
96	Southern Latin America	2015	88.49895247
96	Southern Latin America	2016	88.45157513
96	Southern Latin America	2017	85.2530009
96	Southern Latin America	2018	83.09846937
96	Southern Latin America	2019	80.73765483
96	Southern Latin America	2020	76.9353832
96	Southern Latin America	2021	72.84193736
96	Southern Latin America	2022	70.11797009
96	Southern Latin America	2023	66.88802461
96	Southern Latin America	2024	63.82133381
96	Southern Latin America	2025	60.94923917
96	Southern Latin America	2026	58.2722795
96	Southern Latin America	2027	55.75957516
96	Southern Latin America	2028	53.37298831
96	Southern Latin America	2029	51.12632099
96	Southern Latin America	2030	49.03854848
96	Southern Latin America	2031	47.10291575
96	Southern Latin America	2032	45.29249838
96	Southern Latin America	2033	43.57735212
96	Southern Latin America	2034	41.96206336
96	Southern Latin America	2035	40.46087116
96	Southern Latin America	2036	39.07234352
138	North Africa and Middle East	1990	375.1784986
138	North Africa and Middle East	1991	369.7254964
138	North Africa and Middle East	1992	365.6559481

138	North Africa and Middle East	1993	364.8011853
138	North Africa and Middle East	1994	363.2506891
138	North Africa and Middle East	1995	359.8204329
138	North Africa and Middle East	1996	355.9961223
138	North Africa and Middle East	1997	353.2364423
138	North Africa and Middle East	1998	348.9363495
138	North Africa and Middle East	1999	344.2446632
138	North Africa and Middle East	2000	337.2342375
138	North Africa and Middle East	2001	336.2326919
138	North Africa and Middle East	2002	332.9404066
138	North Africa and Middle East	2003	329.6494127
138	North Africa and Middle East	2004	323.5315996
138	North Africa and Middle East	2005	317.1869987
138	North Africa and Middle East	2006	312.7945323
138	North Africa and Middle East	2007	308.3976962
138	North Africa and Middle East	2008	306.7505259
138	North Africa and Middle East	2009	305.8938502
138	North Africa and Middle East	2010	301.3394327
138	North Africa and Middle East	2011	295.4781042
138	North Africa and Middle East	2012	293.0656448
138	North Africa and Middle East	2013	291.4986468
138	North Africa and Middle East	2014	291.4852322
138	North Africa and Middle East	2015	293.7906289
138	North Africa and Middle East	2016	288.8581421
138	North Africa and Middle East	2017	283.3826777
138	North Africa and Middle East	2018	280.1142926
138	North Africa and Middle East	2019	280.2960456
138	North Africa and Middle East	2020	278.6714022
138	North Africa and Middle East	2021	275.1865845
138	North Africa and Middle East	2022	269.030793
138	North Africa and Middle East	2023	266.6224248
138	North Africa and Middle East	2024	264.2540868
138	North Africa and Middle East	2025	261.8771866
138	North Africa and Middle East	2026	259.5129138
138	North Africa and Middle East	2027	257.197336
138	North Africa and Middle East	2028	254.961124
138	North Africa and Middle East	2029	252.7871118
138	North Africa and Middle East	2030	250.6425317
138	North Africa and Middle East	2031	248.5522248
138	North Africa and Middle East	2032	246.5477584
138	North Africa and Middle East	2033	244.6326549
138	North Africa and Middle East	2034	242.7818115
138	North Africa and Middle East	2035	240.9713914
138	North Africa and Middle East	2036	239.2358228
104	Caribbean	1990	255.5096516
104	Caribbean	1991	245.5102279
104	Caribbean	1992	241.5007819
104	Caribbean	1993	239.3214211
104	Caribbean	1994	233.3003671
104	Caribbean	1995	226.542468

104	Caribbean	1996	220.0247594
104	Caribbean	1997	210.9481528
104	Caribbean	1998	204.9928549
104	Caribbean	1999	198.0013522
104	Caribbean	2000	189.0054291
104	Caribbean	2001	184.3065324
104	Caribbean	2002	177.7519742
104	Caribbean	2003	178.3940303
104	Caribbean	2004	179.3879789
104	Caribbean	2005	179.2299946
104	Caribbean	2006	173.6088796
104	Caribbean	2007	169.8732394
104	Caribbean	2008	167.5251339
104	Caribbean	2009	165.2427136
104	Caribbean	2010	161.4358232
104	Caribbean	2011	156.6562684
104	Caribbean	2012	154.8251162
104	Caribbean	2013	155.2512309
104	Caribbean	2014	154.8699229
104	Caribbean	2015	155.7613554
104	Caribbean	2016	155.1301827
104	Caribbean	2017	155.9570029
104	Caribbean	2018	154.9544575
104	Caribbean	2019	154.791485
104	Caribbean	2020	155.0539455
104	Caribbean	2021	153.1769382
104	Caribbean	2022	151.4931462
104	Caribbean	2023	150.2712902
104	Caribbean	2024	149.1652357
104	Caribbean	2025	148.1681712
104	Caribbean	2026	147.2629099
104	Caribbean	2027	146.5138851
104	Caribbean	2028	145.9519544
104	Caribbean	2029	145.5896145
104	Caribbean	2030	145.4267554
104	Caribbean	2031	145.4605121
104	Caribbean	2032	145.7528541
104	Caribbean	2033	146.3238125
104	Caribbean	2034	147.18623
104	Caribbean	2035	148.3488315
104	Caribbean	2036	149.8291065
73	Western Europe	1990	198.5092594
73	Western Europe	1991	193.6380725
73	Western Europe	1992	187.6094052
73	Western Europe	1993	183.6531145
73	Western Europe	1994	176.3461193
73	Western Europe	1995	170.9996753
73	Western Europe	1996	165.5115862
73	Western Europe	1997	159.6892019
73	Western Europe	1998	156.0362248

73	Western Europe	1999	150.2435407
73	Western Europe	2000	141.234311
73	Western Europe	2001	134.9950466
73	Western Europe	2002	131.3514962
73	Western Europe	2003	127.0657662
73	Western Europe	2004	118.1723005
73	Western Europe	2005	111.8112616
73	Western Europe	2006	105.8992819
73	Western Europe	2007	101.9932696
73	Western Europe	2008	97.27751725
73	Western Europe	2009	93.01399773
73	Western Europe	2010	88.29670667
73	Western Europe	2011	84.46828476
73	Western Europe	2012	82.06719234
73	Western Europe	2013	79.2145206
73	Western Europe	2014	75.42453607
73	Western Europe	2015	74.34003252
73	Western Europe	2016	71.58170199
73	Western Europe	2017	69.20011422
73	Western Europe	2018	66.99605511
73	Western Europe	2019	64.47600573
73	Western Europe	2020	63.10892013
73	Western Europe	2021	61.47662207
73	Western Europe	2022	57.78725357
73	Western Europe	2023	55.90757844
73	Western Europe	2024	54.08521412
73	Western Europe	2025	52.36004592
73	Western Europe	2026	50.75410177
73	Western Europe	2027	49.24134343
73	Western Europe	2028	47.80801847
73	Western Europe	2029	46.43869248
73	Western Europe	2030	45.16504354
73	Western Europe	2031	43.99719185
73	Western Europe	2032	42.89380146
73	Western Europe	2033	41.82549289
73	Western Europe	2034	40.78817067
73	Western Europe	2035	39.81886865
73	Western Europe	2036	38.9342534

Supplemental Tables

Table S1. Definitions of metrics and measures used in the Global Burden of Disease (GBD) 2021 Study.

Table S2. 2021 Socio-demographic index (SDI) classification criteria and corresponding quintiles.

Table S3. Socio-demographic Index values for 204 countries and territories, 1990-2021.

Table S4. Trends in Global, SDI Regions, and GBD regions ischemic heart disease mortality: total number of deaths and age-standardized mortality rate (ASMR), 1990-2021.

Table S5. Trends in age-standardized mortality rate (ASMR) for ischemic heart disease across 204 countries and territories: EAPC and net drift analysis, 1990-2021.

Table S6. Shifts in age distribution of ischemic heart disease (IHD) deaths by SDI region: Total number and proportion of deaths across age groups, 1990-2021.

Table S7. Trends in sex-specific age-standardized mortality rates (ASMR) for ischemic heart disease across global and SDI Regions: Male-to-female ratio analysis, 1990-2021.

Table S8. Local drift analysis of ischemic heart disease mortality: age-specific percent changes in mortality rates by sex across SDI quintiles, 1990-2021.

Table S9. Age effects on ischemic heart disease mortality rates by sex across SDI regions: Analysis of rate increases with age, 1990-2021.

Table S10. Period effects on ischemic heart disease mortality rates: Rate ratios by sex and SDI regions, 1990-2021.

Table S11. Cohort effects on ischemic heart disease mortality rates by sex across SDI regions: Relative risks across birth cohorts, 1902-2002.

Table S12. Frontier analysis of ischemic heart disease mortality and socio-demographic index (SDI): Effective differences and trends, 1990-2021.

Table S13. Decomposition analysis of ischemic heart disease mortality: Contributions of Aging, population growth, and epidemiological changes across SDI and GBD regions, 1990-2021.

Table S14. Risk factor analysis of ischemic heart disease mortality: Contributions of top 8 risk factors by region in 1990 and 2021.

Table S15. Bayesian APC Projections of Ischemic heart disease mortality: Projected number of deaths across regions, 2022-2036.

Table S16. Bayesian APC projections of age-standardized mortality rates (ASMR) for ischemic heart disease across regions, 2022-2036.

Supplemental Figures

Figure S1. (A) Trends in death numbers (in thousands) from 1990 to 2021, categorized by SDI regions among specific age groups (15−19 years to 95+ years). **(B)** Contributions of age stratification to total deaths (in percentages) across different global regions in 1990 and 2021, highlighting the distribution of deaths among various age groups (75+ years, 60−75 years, 45−59 years, 30−44 years, 15−29 years).

Figure S2. (A) Trends in death numbers (in thousands) for males and females from 1990 to 2020, presented for each SDI region and globally. **(B)** Age-standardized mortality rates (ASMR, per 100,000 population) for males and females from 1990 to 2020, categorized by SDI regions and globally. **(C)** Ratio of male to female deaths from 1990 to 2020 across different SDI regions and globally, highlighting the differences in mortality between genders.

Checklist of information that should be included in new reports of global health estimates

Item #	Checklist item	Reported on page #
**Objectives and funding**		
**1**	Define the indicator(s), populations (including age, sex, and geographic entities), and time period(s) for which estimates were made.	Page7-8
**2**	List the funding sources for the work.	Page25
**Data Inputs**		
For all data inputs from multiple sources that are synthesized as part of the study:		
**3**	Describe how the data were identified and how the data were accessed.	Page8-9
**4**	Specify the inclusion and exclusion criteria. Identify all ad-hoc exclusions.	Page8-10
**5**	Provide information on all included data sources and their main characteristics. For each data source used, report reference information or contact name/institution, population represented, data collection method, year(s) of data collection, sex and age range, diagnostic criteria or measurement method, and sample size, as relevant.	Page8-10
**6**	Identify and describe any categories of input data that have potentially important biases (e.g., based on characteristics listed in item 5).	Not applicable
For data inputs that contribute to the analysis but were not synthesized as part of the study:		
**7**	Describe and give sources for any other data inputs.	Not applicable
For all data inputs:		
**8**	Provide all data inputs in a file format from which data can be efficiently extracted (e.g., a spreadsheet rather than a PDF), including all relevant meta-data listed in item 5. For any data inputs that cannot be shared because of ethical or legal reasons, such as third-party ownership, provide a contact name or the name of the institution that retains the right to the data.	Supplementary Tables
**Data analysis**		
**9**	Provide a conceptual overview of the data analysis method. A diagram may be helpful.	Page10-11
**10**	Provide a detailed description of all steps of the analysis, including mathematical formulae. This description should cover, as relevant, data cleaning, data pre-processing, data adjustments and weighting of data sources, and mathematical or statistical model(s).	Supplementary Methods
**11**	Describe how candidate models were evaluated and how the final model(s) were selected.	Supplementary Methods
**12**	Provide the results of an evaluation of model performance, if done, as well as the results of any relevant sensitivity analysis.	Not applicable
**13**	Describe methods for calculating uncertainty of the estimates. State which sources of uncertainty were, and were not, accounted for in the uncertainty analysis.	Supplementary Methods
**14**	State how analytic or statistical source code used to generate estimates can be accessed.	Not applicable
**Results and Discussion**		
**15**	Provide published estimates in a file format from which data can be efficiently extracted.	Page11-18
**16**	Report a quantitative measure of the uncertainty of the estimates (e.g. uncertainty intervals).	Page11-18
**17**	Interpret results in light of existing evidence. If updating a previous set of estimates, describe the reasons for changes in estimates.	Page18-23
**18**	Discuss limitations of the estimates. Include a discussion of any modelling assumptions or data limitations that affect interpretation of the estimates.	Page23-24

This checklist should be used in conjunction with the GATHER statement and Explanation and Elaboration document, found on gather-statement.org
